# Neuritin reverses deficits in murine novel object associative recognition memory caused by exposure to extremely low-frequency (50 Hz) electromagnetic fields

**DOI:** 10.1038/srep11768

**Published:** 2015-07-03

**Authors:** Qian-Ru Zhao, Jun-Mei Lu, Jin-Jing Yao, Zheng-Yu Zhang, Chen Ling, Yan-Ai Mei

**Affiliations:** 1 Institutes of Brain Science, School of Life Sciences and State Key Laboratory of Medical Neurobiology, Fudan University, Shanghai 200433, China; 2 Division of Cellular and Molecular Therapy, Department of Pediatrics, University of Florida College of Medicine, Gainesville, FL 32611, USA

## Abstract

Animal studies have shown that electromagnetic field exposure may interfere with the activity of brain cells, thereby generating behavioral and cognitive disturbances. However, the underlying mechanisms and possible preventions are still unknown. In this study, we used a mouse model to examine the effects of exposure to extremely low-frequency (50 Hz) electromagnetic fields (ELF MFs) on a recognition memory task and morphological changes of hippocampal neurons. The data showed that ELF MFs exposure (1 mT, 12 h/day) induced a time-dependent deficit in novel object associative recognition memory and also decreased hippocampal dendritic spine density. This effect was observed without corresponding changes in spontaneous locomotor activity and was transient, which has only been seen after exposing mice to ELF MFs for 7-10 days. The over-expression of hippocampal neuritin, an activity-dependent neurotrophic factor, using an adeno-associated virus (AAV) vector significantly increased the neuritin level and dendritic spine density. This increase was paralleled with ELF MFs exposure-induced deficits in recognition memory and reductions of dendritic spine density. Collectively, our study provides evidence for the association between ELF MFs exposure, impairment of recognition memory, and resulting changes in hippocampal dendritic spine density. Neuritin prevented this ELF MFs-exposure-induced effect by increasing the hippocampal spine density.

Extremely low-frequency electromagnetic fields (ELF MFs) are ubiquitously present in various environments in everyday life. The major sources of 50 Hz magnetic fields (ELF MFs) pertaining to the general public are in-house installations, household appliances and power lines^1^. Mounting studies have noted that exposure to ELF MFs may both positively and negatively influence the learning and memory of rodents and engender the increases in behavioral anxiety and stress^2^. Studies have continued to investigate potential interaction mechanisms, and a number of possible mechanisms have been suggested, including effects on neural plasticity and changes in oxidative stress^3^,^4^. A recent study by Xiong *et al*. (2013) indicated that exposing rats to ELF MFs for 14 or 28 days induced a decrease in spine density in the superficial layers of the medial entorhinal cortex^5^. Since the structure and density of dendritic spines are crucial determinants of neuronal input-output transformations and resulting synaptic plasticity, these data imply that ELF MFs exposure might induce impairment in cognitive functions via reduction of spine number. However, whether this association is causative requires further investigation.

Rats and mice have a tendency to interact more with a novel object than with a familiar object. This tendency has been exploited as the novel object recognition test (NORT) and is used by behavioral pharmacologists and neuroscientists to study learning and memory^6^,^7^. Previous studies have shown that NORT is associated with various hippocampal functions. For example, Trimper *et al*. (2014) reported that hippocampal synchrony (defined as CA3-CA1 coherence) markedly increased in the low gamma range when rats were exploring novel objects^8^. This effect was particularly evident for those objects for which the rat subsequently showed good memory. Furthermore, Zisopoulou *et al*. (2013) recently presented the first direct evidence that PKCε activation in rat hippocampus is an essential molecular component of object recognition memory^9^.

Although NORT has been used to study differences in mutant mice, aging deficits, and developmental consequences, and as a tool to evaluate the effects of drugs on memory and recognition^10^,^11^,^12^, direct evidence for whether exposure to ELF MFs induces impairments in NORT is still lacking. Moreover, whether exposure to ELF MFs results in a decrement of dendritic spine density and whether this is concurrent with deficits in NORT are also unclear.

Neuritin, also known as CPG15, was discovered in a novel gene screen to identify new candidates involved in activity-dependent synaptic plasticity in the neocortex^13^,^14^. Neuritin was subsequently identified as an important neurotrophin that is expressed in the developing nervous system^15^. It plays multiple roles in the process of neural development, such as during synaptic plasticity and synaptic maturation^16^,^17^. To this end, experimental studies have reported that exposure of either primary embryonic hippocampal or cortical neurons to purified recombinant neuritin promotes neurite outgrowth and arborization^13^,^14^. Hyeon Son’s recent study (2012) indicated that neuritin is decreased by chronic, unpredictable stress and that hippocampal, viral-mediated expression of neuritin produces antidepressant actions and prevents the atrophy of dendrites and spines^18^. Finally, the ability of neuritin to increase neuroplasticity was confirmed in two hippocampal-dependent learning tasks, object recognition and contextual fear conditioning. However, whether neuritin is capable of reversing ELF MFs exposure-induced decreases in dendritic spine density is still not clear.

The aim of the present study was to determine the concurrent effects of ELF MFs on the density of dendritic spines and novel object recognition, and whether the introduction of neuritin would be able to reverse any losses. We found that exposure of ICR mice to ELF MFs significantly decreased the density of hippocampal dendritic spines and resulted in deficits in recognition memory. Importantly, over-expression of neuritin by using an adeno-associated virus (AAV) vector reversed both ELF MFs exposure-induced reduction of dendritic spine density and the resulting deficits in recognition memory.

## Results

### Mice exposed to ELF MFs had deficits in hippocampus-dependent recognition memory but no concurrent changes in their locomotor activity

**Figure 1:**
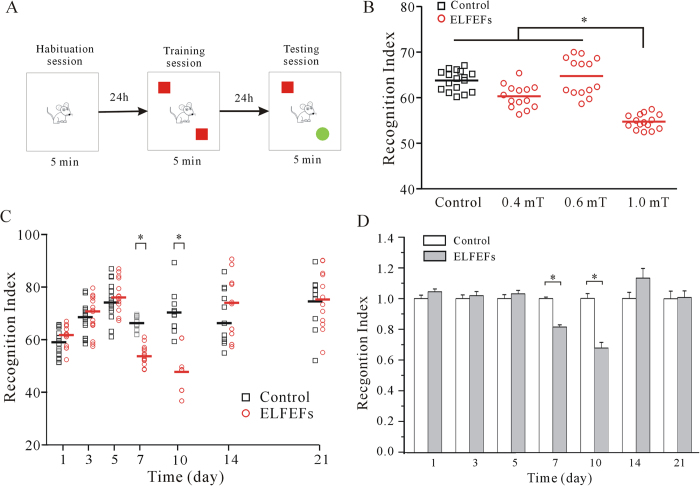
Effect of exposure to ELF MFs on recognition memory of mice using NORT. (**A**) Training and testing scheme for NORT. (**B**) Intensity-dependent influence on recognition index of mice. *P < 0.05, for the groups connected by a straight line using one-way Kruskal-Wallis test with Dunn’s post-hoc. (**C**) Original data showing the time-dependent effect of ELF MFs exposure. *P < 0.05, for two groups connected by a straight line using one-way Kruskal-Wallis test with Dunn’s post-hoc. (**D**) The data normalized to non-ELF MFs-exposed mice in the same day. *P < 0.05, for two groups connected by a straight line using Student’s t test.

To address whether ELF MFs exposure affects the recognition memory ability of mice, NORT was used. A schematic of the NORT procedure is shown in Fig. 1A. The abilities of mice to recognize novel object were determined by dividing the mean time spent exploring the novel object by the total mean time exploring both novel and familiar objects during the test session. This value was multiplied by 100 to obtain a percentage preference for the novel object. This is termed as recognition index, with the standard formula: (T_novel_/[T_novel _+ T_familiar_] × 100).

We then assessed the effect of ELF MFs exposure on long-term recognition memory by comparing the recognition index between the non-ELF MFs and ELF MFs exposure groups. When the mice were continuously exposed for 12 h/day for 7 days with a field intensity of 1.0 mT (n = 15), the recognition index significantly decreased compared to the non-ELF MFs control mice (n = 16, *P* < 0.05, Fig. 1B). The decrease in the recognition index resulting from ELF MFs exposure was time–dependent, which was only observed after exposing mice to ELF MFs for 7–10 days.

Figure 1C shows the original data obtained from mice, which were exposed to 1 mT ELF MFs for 1 to 21 days. We found that the recognition index increased slightly over the first five days before plateauing (Fig. 1C). The results indicated that exposure to 1.0 mT ELF MFs for 12 h/day for 7 and 10 days led to a significant decrease in recognition index by 18.5% (n = 15–16, *P* < 0.05) and 32.3% (n = 12, *P* < 0.05), respectively when normalized to the corresponding non-ELF MFs controls (Fig. 1D). Moreover, after a 14-day exposure to ELF MFs, a further 21 days ELF MFs exposure did not induce any further decreases in recognition index (Fig. 1D).

**Figure 2:**
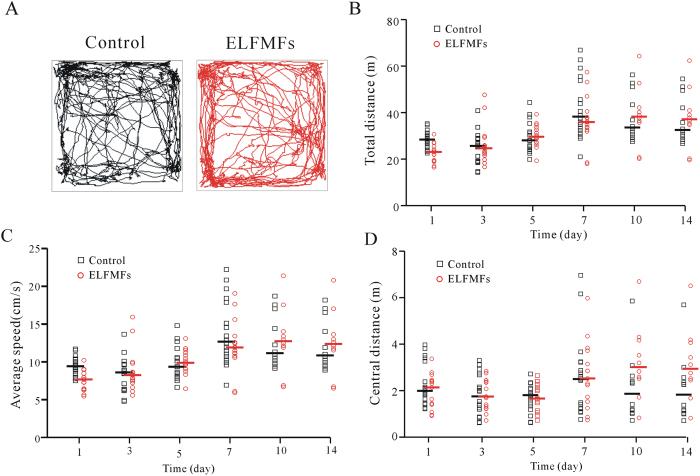
Effect of ELF MFs exposure on locomotor activity of mice was evaluated with open field test. (**A**) Sample traces of non-ELF MFs and ELF MFs exposure mice in the open field test. (**B**–**D**) Total distance, average speed and central distance that subjects moved in the horizontal plane. One way Kruskal-Wallis test with Dunn’s post-hoc indicated that ELF MFs exposure groups were not statistically significant with the corresponding non-ELF MFs exposed controls.

It is possible that the decrease in recognition index caused by ELF MFs exposure might be due to the changes in motor ability or anxiety-like behavior of mice. To explore these possibilities, locomotor activity was evaluated in both ELF MFs and non ELF MFs-exposed mice using open field test (Fig. 2A). The results showed that there was no statistically significant difference between non-ELF MFs (n = 12–18) and ELF MFs-exposed groups (n = 10–15) in the total horizontal distance traveled in each 5 min time period (Fig. 2B). The average speed during these 5 min intervals was also not significantly different between the control and ELF MFs-exposed groups (Fig. 2C). These data indicated that continuous exposure to ELF MFs did not affect the motor ability of the mice. Previous studies have suggested that the center distance and average speed can be used as indices of anxiety-related responses^19^. In this study, mice exposed to 1 mT ELF MFs for 12 h per day for 1–14 days did not differ from controls in measures of center distance (Fig. 2D). These results suggest that the ELF MFs exposure-induced decrement in recognition scores was not due to the general anxiety-like behavior.

### ELF MFs exposure reduced dendritic spine densities of hippocampal CA1 pyramidal cells

**Figure 3:**
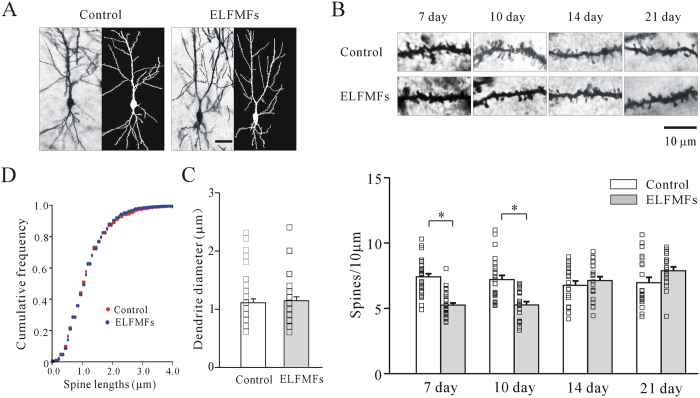
Effect of ELF MFs exposure on hippocampal neurons morphology as observed with Golgi staining. (**A**) The hippocampal neurons visualized with Golgi staining (left) and reconstructed using imaging analysis software (right) under ELF MFs exposure and non-ELF MFs exposure. Scale bar is 10 μm. (**B**) Representative images and statistical analyses of spine density on second-order dendrites of hippocampus neurons after ELF MFs exposure from 10 to 21 days. Scale bar is 10 μm. *P < 0.05, for two groups connected by a straight line using with Student’s t test. (**C** and **D**) Effect of ELF MFs exposure on dendritic diameter and spine length of 10 days in hippocampal neurons.

Recent work by Xiong *et al*. (2013) demonstrated that ELF MFs exposure can decrease dendritic spine densities^5^. We thus used Golgi staining to investigate the effect of ELF MFs exposure on dendritic spine densities in hippocampal CA1 pyramidal cells. This region was chosen because of its strong link to recognition memory^8^,^20^. Spine density on secondary apical dendrite of CA1 pyramidal cells from three mice of each group was measured (Fig. 3A).

Dendritic diameter was first measured before comparison of spine densities between the non-ELF MFs and ELF MFs exposure groups to avoid differences caused by diameter variation. No significant differences in dendritic diameter were observed between the two groups (Fig. 3C). The results showed that spine density was significantly decreased by 29% (from 7.4 ± 0.2 to 5.3 ± 0.2 per 10 μm, n = 29 and 31 in non-ELF MFs and ELF MFs exposure groups respectively, p < 0.05) and 27% (from 7.2 ± 0.3 to 5.3 ± 0.2 per 10 μm, n = 25 and 21 in non-ELF MFs and ELF MFs exposure groups respectively, p < 0.05) in mice exposed to ELF MFs (1.0 mT,12 h/day) for 7 days or 10 days, respectively. However, the spine densities in mice exposed to ELF MFs for 14 or 21 days were similar to those in corresponding control mice (Fig. 3B). These results were consistent with the results of the NORT recognition indices (Fig. 1D).

In addition, we also tested the effect of ELF MFs on spine length. The cumulative frequency distributions of spine length in non-ELF MFs and ELF MFs-exposed mice were compared using a Kolmogorov-Smirnov test. The results indicated that there were no significant differences in spine length between the two groups (Fig. 3D).

### Viral neuritin over-expression in hippocampus reversed ELF MFs exposure-induced decrease in spine densities and the corresponding deficits in recognition memory

**Figure 4:**
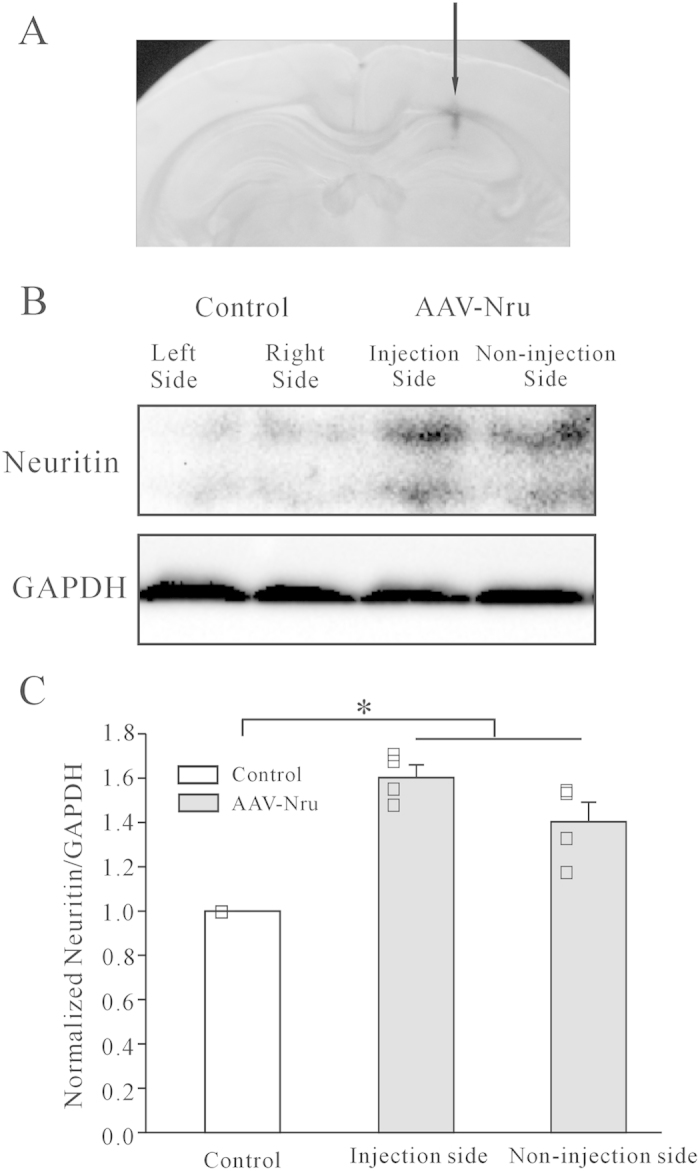
Effect of AAV-mediated neuritin expression on hippocampal neuritin protein levels. (**A**) Representative images of the sites with microinjection of AAV-neuritin cDNA into the hippocampus. (**B**, **C**) Western blot and statistical analyses showing expression levels of neuritin in hippocampus tissues on either AAV-infected or non-infected side (n = 4). *P < 0.05, for the groups connected by a straight line using one-way ANOVA with Turkey’s post-hoc.

Neuritin is an important neurotrophin, and previous work has shown that over-expression of neuritin could increase the spine density of hippocampal pyramidal neurons^21^. Given this, we next examined whether over-expression of neuritin could reverse the decrease in spine densities and the subsequent deficits in recognition memory in mice exposed to ELF MFs. Adeno-associated virus serotype 9 (AAV9) vectors carrying human cpg15 cDNA were produced and stereotaxically injected into the CA1 region of the hippocampus (0.2 μl each mouse) 7 days before exposure to ELF MFs (Fig. 4A).

To confirm the efficiency of neuritin expression mediated by the AAV expression system, the hippocampal neuritin protein levels were assessed using western blotting. We injected unilaterally, keeping the hemisphere untouched to act as an internal control. The results indicated that the expression of neuritin was increased by 60.2 ± 5.8% in the injection side of the hippocampus. Moreover, the expression of neuritin in the non-injected control side of the hippocampus was also increased (40.3 ± 8.9%; Fig. 4C), suggesting that the overall neuritin levels in the whole hippocampus were increased. The increase of neuritin expression in the non-injected side is likely due to spreading of the AAV virus after infection, and/or secretion of neuritin.

**Figure 5:**
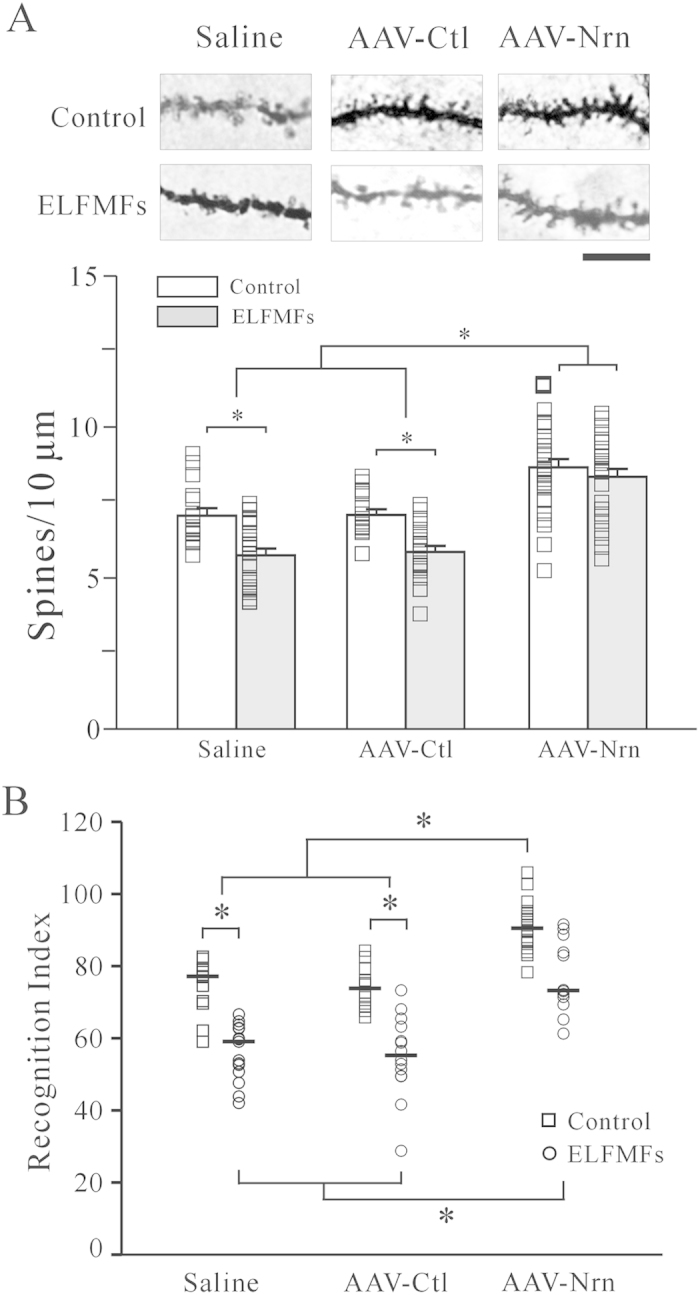
Effect of AAV-mediated neuritin expression on dendritic spine density and recognition memory of mice after ELF MFs exposure. (**A**) Representative images and statistical analyses of spine density on dendrites of hippocampus neurons after saline, AAV-control (AAV-Ctl) or AAV-neuritin (AAV-Nrn) infection under ELF MFs exposure. Scale bar is 10 μm. *P < 0.05, for the groups connected by a straight line using one-way ANOVA with Turkey’s post-hoc. (**B**) The recognition indices of mice obtained after saline, AAV-Ctl or AAV-Nrn infection with ELF MFs exposure. *P < 0.05, for the groups connected by a straight line using one-way Kruskal-Wallis test with Dunn’s post-hoc.

We then quantified the effects of AAV9-mediated neuritin over-expression on hippocampal dendritic spine number and subsequent recognition memory in mice of both non-ELF MFs and ELF MFs exposure groups. The experimental group and two control groups were injected with the same volume of AAV-neuritin, AAV-control and saline in bilateral hippocampus, respectively. After a 2.5-week infection period, a significant increase in spine density was observed in the AAV-neuritin group. While there is no significant difference in spine density between the AAV-control and saline control groups (Fig. 5A). The pyramidal neurons from mice transfected with saline or AAV-control had an average spine density of 7.1 ± 0.3 and 7.1 ± 0.2 per 10 μm of dendritic length, respectively (n = 16 and 16). Conversely, hippocampus neurons transduced with AAV-neuritin had an average spine density of 8.7 ± 0.3 per 10 μm dendritic length (n = 29). Thus, over-expression of neuritin led to a 22.3% increase in dendritic spine density (Fig. 5A).

Additionally, after ELF MFs exposure (1 mT) for 12 h/day for 10 days, the spine density of pyramidal neurons obtained from neuritin-expression mice was no longer reduced. Sampled neurons showed an increase of 17.9% in spine density. Furthermore, the recognition memory test of AAV-neuritin mice showed a significant increase in recognition index when compared to AAV-control mice. This result is consistent with the neuron spine density values obtained in AAV-neuritin mice. Importantly, the recognition index was not reduced by ELF MFs exposure while hippocampal neuritin was over-expressed (Fig. 5B).

**Figure 6:**
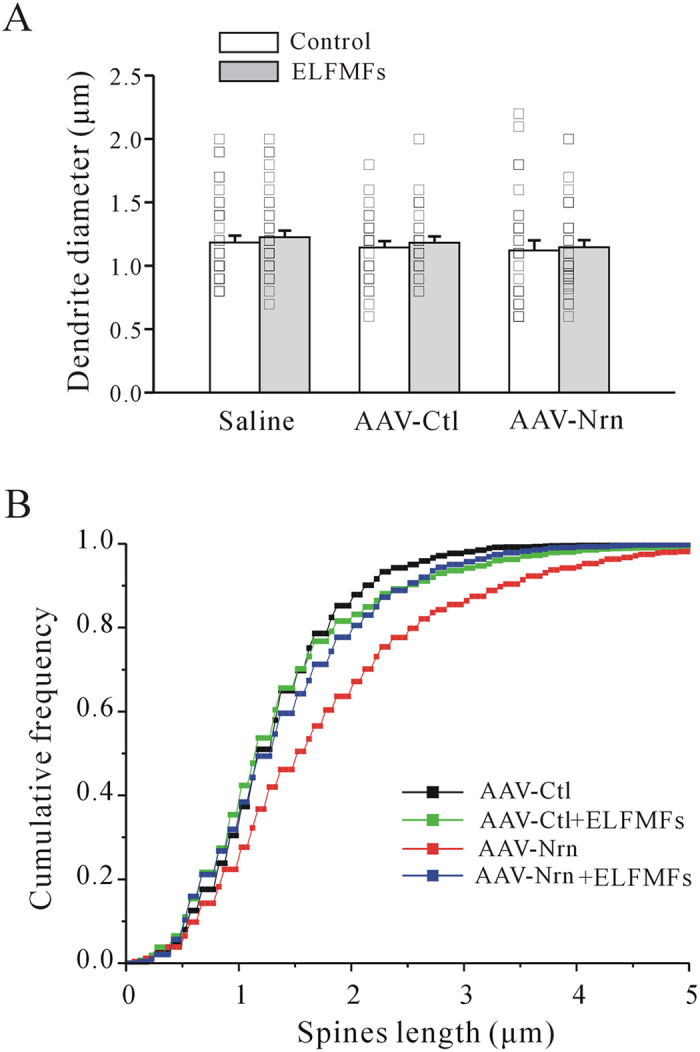
Effect of AAV-mediated neuritin expression on dendritic diameter and spine length after ELF MFs exposure. (**A**) Statistical analyses of dendritic diameter of hippocampal neurons after saline, AAV-Ctl or AAV-Nrn infection in ELF MFs-exposed hippocampus. (**B**) Effect of ELF MFs exposure on spine length of hippocampal neurons after saline, AAV-Ctl or AAV-Nrn infection in hippocampus.

Finally, we quantified the effect of AAV-neuritin alone or AAV-neuritin with ELF MFs exposure on dendritic diameter and dendritic spine length. As shown in Fig. 6A, there was no significant difference in dendritic diameter between AAV-neuritin, AAV-control, and saline control groups. Moreover, ELF MFs exposure (1 mT) for 12 h/day for 10 days did not change the dendritic diameter of mice in any of the three groups (Fig. 6A). The results of cumulative frequency distributions of spine length indicated that AAV-neuritin expression significantly increased dendritic spine length, which was reduced by ELF MFs exposure (Fig. 6B).

## Discussion

Although the effect of chronic exposure to ELF MFs on rodent learning and memory has been previously studied, the results were inconsistent. Using the Morris water maze test, Liu (2008) and He *et al*. (2011) found that ELF MFs exposure (1–2 mT; 50 Hz; 1 or 4 h/day for 4 weeks) improved long-term memory for platform location without affecting either short-term memory or motor activity^22^,^23^. Leone *et al*. (2014) reported that the ELF MFs-exposed mice (1 mT; 50 Hz; 3.5 h/day for 12 days) showed a higher discrimination ability compared with controls, and exposure to ELF MFs modulated adult hippocampal neurogenesis by epigenetic mechanisms leading to pro-neuronal gene expression^24^. ELF MFs exposure (1 mT; 50 Hz; 3.5 h/day for 6 days) was also reported to enhance the survival of newborn neurons in mouse hippocampus^25^. With a huntington’s disease rat mode, Tasset *et al*. (2012) found that behavioral changes of OFT tests and loss of neurons in rat striatum induced by 3-nitropropionic acid (3NP) administration were neutralized by application of ELF MFs (60 Hz and 0.7 mT, 2 h in the morning and 2 h in the afternoon for 21 days)^26^. On the other hand, Liu (2013) and Cui *et al*. (2012) reported significant cognitive dysfunction in rodents that received prolonged ELF MFs exposure for 4 h/day for 12 consecutive weeks or 60 days^27^,^28^. In our study, ELF MFs exposure reversibly reduced the recognition memory after 7 to 10 days. The discrepancies between our data and previous reports suggest that the given frequency, intensity and duration of exposures to ELF MFs are critical for their effects. Considering many people are exposed to ELF MFs almost continuously in their daily lives, in our experiment, we chose an exposure model with mice receiving ELF MFs for 12 h/day to mimic these circumstances. However, since the 1 mT ELF MFs level, which induced the impairment of recognition memory and potential changes in hippocampal dendritic spine density in this study, is not within the general range in daily human lives, our study is more significant for elucidation of biological mechanisms than for human health risk assessment.

Although previous research on the role of the hippocampus in object recognition memory has produced conflicting results, this variability may be due to methodological considerations such as the diversity of animal type and age, object shape and color, testing environment, and so on^6^. Moreover, the hippocampus is still considered an important neuroanatomical substrate for novel object recognition^29^, particularly in the maintenance of strong, novel object preference after long delays^30^.

In our study, wild-type mice spent more time exploring a novel object than they spent exploring a familiar object, thus reflecting their memory of the familiar object. ELF MFs exposure reduced the time spent exploring a novel object after 7 to 10 days, thus showing a decrease in recognition memory for the familiar object. Considering previous studies have reported that ELF MFs exposure has anxiogenic effect in rats^22^, and that a failure to discriminate between the objects may be a function of compromised motor ability or ataxia, locomotor activity was monitored in our study. The results indicated that spontaneous locomotor activity had no difference between groups, suggesting that ELF MFs exposure did not produce ataxia or motor impairments in the mice under the experimental parameters used in this study, and the ELF MFs exposure-induced decrement in recognition scores was not due to the general anxiety-like behavior. Dendritic spines are the primary site of excitatory input on most principal neurons, and are essential for functional synaptic plasticity and cognition functioning. Accordingly, alterations in spine shape, size, and number induce long-lasting changes in synaptic activity and cognition function^31^,^32^. In the present study, chronic exposure to ELF MFs also appeared to affect spine density in hippocampal neurons. These results are similar to what Xiong *et al*. (2013) found in the entorhinal cortex^5^. Moreover, the timing of appearance and the reversible pattern in impairment of recognition memory paralleled the reduction of spine density. These results provide evidence for the association between the impairment of recognition memory induced by ELF MFs exposure and the reduction of hippocampal spine density. We noted that dendritic spines are classified into three morphological categories: mushroom spine, thin spine and stubby spine according to the ratio of spine head width to spine neck width and the number of spine heads^33^. The study by Xiong *et al*. (2013) found that ELF MFs-induced changes in the density of spines were different among the three types of spines in entorhinal cortical neurons^5^. We did not find a similar difference among morphologically distinct spines after ELF MFs exposure due to the resolution limit of Golgi staining. This limitation may partly explain why the overall percentage of spine reduction in our study was slightly lower than that found in the report of Xiong *et al*. (2013)^5^. To this end, Xiong *et al*. (2013) found that there was no significant change in the density of thin spines after a 14-day ELF MFs exposure, but there was a significant reduction in thin spines (26%) after a 28-day ELF MFs exposure^5^. The difference between the findings of Xiong *et al*.^5^ and ours could be attributed to different brain regions and/or to variation in the dosage and duration of ELF MFs exposure. It is also worth noting that in our study, the ELF MFs-exposure-induced reduction in spine density and the subsequent deficits in recognition memory are transient and reversible. Similarly, Liu *et al*. (2013) also reported recovery of ELF MFs-exposure-induced effects on rat cognitive function and hippocampal morphology, while the treatment parameters and recovery time window are different. In their study, the ELF MFs treatment was applied at 0.4 mT for 24 h/day for 60 days^28^. The ELF MFs-exposure-induced effects on rat cognitive function and hippocampal morphology were recovered when the ELF MFs exposure was stopped after 7 or 15 days treatment. Our results showed that the reduction of spine density and deficits in recognition memory occurred after 7 days of ELF MFs exposure, with the most significant effect appearing after 10 days of ELF MFs exposure, followed by a gradual recovery. From day 14, the initial reduction in spine density and recognition memory caused by ELF MFs-exposure was recovered to the control levels. Moreover, the recognition indices of mice in both ELF MFs treatment and control groups were increased similarly over the first few days of exposure. Cui *et al*.’s (2012) study indicated that ELF MFs exposure impaired hippocampal-dependent spatial learning and striatum-dependent habit learning, and that this impairment was associated with oxidative stress in the hippocampus and striatum induced by ELF MFs exposure^27^. However, an increase in the transcription of pro-neuronal genes (Mash1, NeuroD2 and Hes1) and genes encoding Cav1.2 channel α1C subunits after ELF MFs exposure (1 to 7 h/ day for 7 days) was observed by Cuccurazzu *et al*. (2010)^34^. Meanwhile, significantly increased neurotrophic factor levels (GDNF and BDNF) were observed in brain tissue after a 21-day ELF MFs treatment^26^. Therefore, we speculated that in the early period of chronic ELF MFs exposure, there was a neuronal oxidative stress response, which induced the reduction of spine density and deficits in recognition memory. The subsequently occurred transcription of genes, increase of neurotrophic factor levels and changes of ion channel activity aroused the self-protective and self-compensatory processes, which might eliminate the effect of ELF MFs exposure. Finally, the increasing age of mice might explain the similar increases in recognition indices of both control and ELF MFs-exposed mice over the first five days.

Neuritin is a critical protein for dendritic outgrowth, maturation, and axonal regeneration^13^,^21^. Neuritin exerts non-cell autonomous function by binding to receptors^14^,^16^, which have been identified as insulin-like receptors by our recent study^35^. Neuritin can be released to extracellular space in a soluble-secreted form *in vivo*^36^,^37^,^38^, and several studies have indicated that this soluble form had neurotrophic effects on mammalian neurons^36^,^37^,^38^. In our study, bilaterally injection of AAV-neuritin in hippocampus was used for testing the effect of over-express neuritin on spine number and the recognition index. However, we also observed that the overall neuritin levels in the whole hippocampus including the non-injected control side of the hippocampus were increased after the injection of AAV-neuritin in one side of hippocampus. The increase of neuritin in the non-injected side is likely due to the spreading of the AAV virus after infection, and/or secretion of neuritin.

Son *et al*. (2012) recently reported that neuritin is decreased by chronic, unpredictable stress and that viral-mediated expression of neuritin in the hippocampus produces antidepressant actions and prevents the atrophy of dendrites and spines^18^. In their study, AAV-neuritin (Nrn) infusion significantly increased spine density and head diameter of mushroom-like spines. The ability of neuritin to promote synaptic plasticity was supported in two hippocampal-dependent learning tasks: novel object recognition and contextual fear conditioning. Our results showed that viral-mediated expression of neuritin in the hippocampus increased the number of spines, both in control and ELF MFs-exposed groups. Importantly, the expression of neuritin in ELF MFs-exposed mice reversed the reduction of the spine number as well as the decrease of the recognition index. These data further clarified the importance of the spine number changes induced by neuritin in recognition memory.

Finally, we showed that besides spine number, neuritin also significantly increased the length of spines in non-ELF MFs exposed mice, but not in ELF MFs-exposed mice. It is highly likely that ELF MFs exposure did not affect the original dendritic spine length, but did attenuate the increase of dendritic spine length induced by AAV-neuritin expression. We also noticed that although ELF MFs exposure modestly decreased the spine length increased by neuritin, it did not affect either the recognition index or the number of spines, suggesting that the number of spines may be more important than the length of the spines in hippocampus-dependent recognition memory. Qin *et al*. (2011) reported that the largest percentage of spines with increased length were filopodial-type spines, which were unlikely to have formed synaptic contacts^19^. Since the effect of ELF MFs exposure on spines was known to be dependent on the type of spines, future studies will be required to characterize which type of spines was increased by neuritin and whether the increased filopodial-type spines were reduced by ELF MFs exposure.

## Conclusion

In the present study, we have determined that 1 mT ELF MFs exposure for 12 h per day for 7–10 days resulted in a deficit in recognition memory via decreasing density of hippocampal dendritic spines. However, this effect was transient and reversible in the three-week time frame of this study. Over-expression of neuritin in hippocampus could reverse ELF MFs-exposure-induced reduction of dendritic spine density and deficit in recognition memory by increasing spine density. Our study not only associates ELF MFs exposure to impaired learning and memory, but also reveals that neuritin-mediated synaptic plasticity counteracts neuronal changes induced by ELF MFs exposure.

## Materials and Methods

The methods were carried out in accordance with the approved guidelines.

### Ethics and Animal Use Statement

This study was conducted in strict accordance to the recommendations in the Guide for the Care and Use of Laboratory Animals put forth by the National Institutes of Health. The protocol was approved by the Committee on the Ethics of Animal Experimentation at Fudan University (Permit No. 20090614-001). All surgery was performed under sodium pentobarbital anesthesia (50 mg/kg, i.p.), and all efforts were made to minimize animal suffering.

### Animals

Experimental subjects were 3 to 4 weeks old female ICR mice (SLC Co., Ltd., Shanghai, China). The mice were housed in plastic cages at room temperature (23–25 °C) with *ad libitum* access to standard food pellets and water. No specific dietary supplements were provided. The light–dark cycle was set at 12 h (lights on from 8:00 AM to 8:00 PM).

### Electromagnetic field production

The system used to expose mice to electromagnetic fields was the same as that used in previous studies (I-ONE, Shanghai, China)^39^,^40^. Briefly, a pair of Helmholtz coils that were placed in opposition to each other generated a 50 Hz electromagnetic field. The coils were powered by an AC generator, which produced a sinusoidal input voltage. The resulting magnetic flux densities could be regulated within a range of 0 to 1.0 mT.

ELF MFs frequency and density were monitored by an ELF MFs sensor that was connected to a digital multimeter. The orientation of the system ensured a uniform field for each glass cage, which contained 4 to 5 experimental mice. The surfaces of the glass cage were parallel to the force lines of the alternating magnetic field in the solenoid. Air temperature was continuously monitored during all the experiments. When compared to non-exposed cages, the maximum temperature variation recorded in an ELF MFs-exposed glass cage was 0.4 ± 0.1 °C. Controls were placed in the same glass cages and submitted to identical conditions as those used for the experimental groups, but without exposure to ELF MFs.

### Open-field test (OFT)

Locomotor activity was evaluated in each different group, including control (no ELF-MFs exposure) and ELF MFs exposure groups. The open field apparatus consisted of a clear Plexiglas box (23 × 23 × 35 cm) with a black floor. Mice were placed in the open field in the dark. Activity was detected by a computer-operated tracking system that consisted of 16 light beams per side (Mobiledatum Information Technology Co., Ltd., Shanghai, China) and recorded continuously over 5 min. The subject’s total horizontal distance moved, center distance and moving speed were measured. The central and peripheral regions were generated automatically by the tracking system software (Mobiledatum Information Technology Co., Ltd., Shanghai, China).

### Novel object recognition test (NORT)

The protocol for NORT was adapted from Ennaceur and Bevins (1988) and utilized an open field consisting of a clear Plexiglas box (45 × 45 × 45 cm)^7^,^41^. NORT was conducted in this open field apparatus for three consecutive days, with 5 min daily trials. On the first day, mice were allowed to explore and habituate to an empty open field box. On the second day, mice were presented with two of the same objects, indicated as A and B. Twenty four hours later, the mice were exposed to two different objects: A and C, with C indicating a novel object. All objects were rinsed with ethanol and allowed to dry between trials and before the first trial. All testing and training sessions were videotaped and analyzed by an experimenter blind to the treatment group of the animals. Object exploration was defined as each instance in which a mouse’s nose touched the object or was oriented toward and within 2 cm of the object. Exploratory activity within the experimental arena was measured over each five minutes trial with a computer timer.

### AAV vectors production

Highly purified stocks of AAV vectors were generated by the triple-plasmid transfection protocol as previously described^42^. The physical genomic titers of recombinant vector stocks were determined by quantitative DNA slot-blot and Southern blot analyses^43^.

### Animal injection procedures

Mice were injected with either saline, AAV-control, or AAV-neuritin virus as previously described^44^. Virus (0.1 μL) was injected into the one side of hippocampus at the following stereotaxic coordinates using bregma as reference: (x) 1.5–2 mm, (y) −2 mm, and (z) 1.5 mm. All injected animals were kept for a one-week recovery prior to either ELF MFs or non-ELF MFs exposure.

### Golgi staining

A FD Rapid GolgiStain kit (FD Neurotechnologies, Baltimore, MD, USA) was used for dendritic spine histology analysis by following the instructions of the kit as previously described^45^. In brief, mouse brains were removed, placed in appropriate impregnation solution, and stored in the dark for 14 days at room temperature. The brains were then transferred into a solution containing sucrose and incubated at 4 °C until the residual water was driven from the tissues (2–7 days). Finally, a vibratome (DTK-1000, Leica, Germany) was used to obtain 100 μm thick, semi-horizontal sections that included the hippocampus. Slices were then mounted onto gelatin-coated slides and allowed to air dry at room temperature for about two days. Once sufficiently dry, the sections were rinsed with distilled water and incubated for ten minutes in a solution containing silver nitrate. Slides were then rinsed with distilled water before being dehydrated in absolute alcohol, cleared with xylene, and covered with non-acidic synthetic balsam and cover slips.

### Western blot analyses

Mice were anesthetized with pentobarbital sodium (50 mg/kg) and decapitated. Brains were removed immediately. Coronal sections (400 μm) were made at the level of hippocampus by vibratome (DTK-1000, Leica, Germany). Then the CA1 region was subdissected with a dissecting microscope. Ice-cold 1.5 ml Eppendorf tubes with NP-40 reagent and 1% protease inhibitor cocktail (sigma, USA) were prepared in advance. After subdissection, the CA1 tissues were moved into the Eppendorf tubes and homogenated with grinding rod on ice. The CA1 homogenate was vortexed for 30 min and centrifugate for 15 min at 4 °C. Proteins were fractionated by 12% SDS gel and transferred to PVDF membrane. Then the membranes were blocked in 10% milk for 1 h and then incubated with the following primary antibodies overnight at 4 °C: anti-neuritin (1:200, R&D systems Inc., USA) and anti-GAPDH (1:5000, KANGCHEN, China) antibodies. After washing with 0.3‰ PBST for three times (15 min/time), the membranes were incubated with the following secondary antibodies for 1 h at room temperature: donkey anti-goat antibody (1:5000, beyotime, China) for neuritin, goat anti-mouse antibody (1:5000, KANGCHEN, China) for GAPDH. After washing with 0.3‰ PBST for three times (15 min/time), the membranes were taken to the molecular imager (Chemi Doc XRS+, BIO-RAD, USA) to detect the protein bands. The chemiluminescence enhanced chemiluminescent (ECL) reagent (Thermo Scientific, USA) was used for detection of protein bands.

### Data acquisition

All image acquisition and analyses were performed by an experimenter who was blind to treatment group. When comparing our slices to reference sections^46^, we selected those slices obtained from −1.5 to 3.0 mm of Bregma. The images for spine analysis were obtained using a laser confocal scanning microscope (LSM 710, Zeiss, Germany) with 1000× magnification. To ensure a homogenous neuronal population, neurons that were selected for analysis had to fulfill the following two criteria: (1) the cell body and dendrites were completely impregnated and (2) the neuron(s) were isolated from the surrounding neurons. We reconstructed intact cell morphology and calculated dendritic length using image analysis software (Neurolucida V9.0, Williston, VT, USA). Dendritic spines number, spine length and dendrite diameter were counted/measured on 20 μm lengths of beginning of the second-order, apical dendrites of pyramidal neurons in the CA1 of the hippocampus. The spine density was expressed as the average number of spines per 10 micron of dendritic length.

### Statistical analysis

Data were presented as mean ± SEM or Median. Statistical analyses were performed using GraphPad Prism software (GraphPad Software, Inc., USA). All statistical assessments were two-sided and the corresponding P values were calculated. A value of P < 0.05 was considered statistically significant. One-way Kruskal-Wallis test followed by Dunn’s test was used for multiple comparisons of the recognition indices and locomotor activity. The normalized recognition indices, dendrite diameters and spine densities for different exposure time were analyzed by an unpaired, two-tailed *t*-test between control and ELF MFs exposure groups. Cumulative frequency distributions of spine length were analyzed using a one-way Kruskal–Wallis test followed by Kolmogorov-Smirnov test. One-way analysis of variance (ANOVA) was used for multiple comparisons of data in western blot, dendrite diameters and spine densities after animal injection, and Tukey’s post-hoc was used for analysis of differences between two groups.

## Additional Information

**How to cite this article**: Zhao, Q.-R. *et al*. Neuritin reverses deficits in murine novel object associative recognition memory caused by exposure to extremely low-frequency (50 Hz) electromagnetic fields. *Sci. Rep*. **5**, 11768; doi: 10.1038/srep11768 (2015).

## Supplementary Material

Supplementary Information
