# Supplementary material for: Neuritin reverses deficits in murine novel object associative recognition memory caused by exposure to extremely low-frequency (50 Hz) electromagnetic fields
Source: Sci Rep. 2015 Jul 3;5:11768. doi: 10.1038/srep11768 (PMC4650637; doi:10.1038/srep11768)
Supplement: Supplementary Information [file srep11768-s1.pdf]

Scientific Committee on Emerging and Newly Identified Health Risks

SCENIHR

Opinion on

Potential health effects of exposure to electromagnetic fields (EMF)

SCENIHR adopted this Opinion at the 9th plenary meeting on 27 January 2015

About the Scientific Committees

Three independent non-food Scientific Committees provide the Commission with the scientific advice it needs when preparing policy and proposals relating to consumer safety, public health and the environment. The Committees also draw the Commission's attention to the new or emerging problems which may pose an actual or potential threat.

They are: the Scientific Committee on Consumer Safety (SCCS), the Scientific Committee on Health and Environmental Risks (SCHER) and the Scientific Committee on Emerging and Newly Identified Health Risks (SCENIHR).

In addition, the Commission relies upon the work of the European Food Safety Authority (EFSA), the European Medicines Agency (EMA), the European Centre for Disease prevention and Control (ECDC) and the European Chemicals Agency (ECHA).

SCENIHR

This Committee deals with questions related to emerging or newly identified health and environmental risks and on broad, complex or multidisciplinary issues requiring a comprehensive assessment of risks to consumer safety or public health and related issues not covered by other Community risk assessment bodies. Examples of potential areas of activity include potential risks associated with interaction of risk factors, synergic effects, cumulative effects, antimicrobial resistance, new technologies such as nanotechnologies, medical devices including those incorporating substances of animal and/or human origin, tissue engineering, blood products, fertility reduction, cancer of endocrine organs, physical hazards such as noise and electromagnetic fields (from mobile phones, transmitters and electronically controlled home environments), and methodologies for assessing new risks. It may also be invited to address risks related to public health determinants and non-transmissible diseases.

Scientific Committee members

Michelle Epstein, Igor Emri, Philippe Hartemann, Peter Hoet, Norbert Leitgeb, Luis Martínez Martínez, Ana Proykova, Luigi Rizzo, Eduardo Rodriguez-Farré, Lesley Rushton, Konrad Rydzynski, Theodoros Samaras, Emanuela Testai, Theo Vermeire

Contact:

European Commission
DG Health and Food Safety
Directorate C: Public Health
Unit C2 – Health information and Scientific Committees
Office: HTC 03/073 L-2920 Luxembourg

SANTE-C2-SCENIHR@ec.europa.eu

© European Commission 2015

ISSN 1831-4783 ISBN 978-92-79-30134-6

Doi: 10.2772/75635 ND-AS-13-004-EN-N

The Opinions of the Scientific Committees present the views of the independent scientists who are members of the Committees. They do not necessarily reflect the views of the European Commission. The Opinions are published by the European Commission in their original language only.

http://ec.europa.eu/health/scientific_committees/policy/index_en.htm

ACKNOWLEDGMENTS

Members of the Working Group are acknowledged for their valuable contribution to different parts of this Opinion. They are:

SCENIHR members:

Prof. Theodoros Samaras, (Chair and co-rapporteur from April 2013) University of Thessaloniki, GR

Prof. Norbert Leitgeb, retired, Graz University of Technology, AT

External experts:

Prof. Anssi Auvinen, University of Tampere and STUK – Radiation and Nuclear Safety Authority, FI

Prof. Dr. Heidi Danker-Hopfe, Charité University of Medicine, Berlin, DE

Dr. Kjell Hansson Mild, Umeå University, SE

Prof. Mats-Olof Mattsson, (Chair of the working group until March 2013 and co-rapporteur) Austrian Institute of Technology, AU

Dr. Hannu Norppa, Finnish Institute of Occupational Health, FI

Dr. G. James Rubin, King's College London, UK

Dr. Maria Rosaria Scarfí, CNR-IREA, IT

Dr Joachim Schüz, International Agency for Research on Cancer, FR

Dr. Zenon Sienkiewicz, Public Health England, UK

Dr. Olga Zeni, CNR-IREA, IT

All Declarations of Working Group members and supporting experts are available at the following webpage:
http://ec.europa.eu/health/scientific_committees/emerging/members_wg/index_en.htm

ABSTRACT

The purpose of this Opinion is to update the SCENIHR Opinions of 19 January 2009 'Health effects of exposure to EMF' and 6 July 2009 'Research needs and methodology to address the remaining knowledge gaps on the potential health effects of EMF' in the light of newly available information since then, and to give special consideration to areas where important knowledge gaps were identified in the previous Opinion. In addition, biophysical interaction mechanisms and the potential role of co-exposures to environmental stressors are discussed.

Exposure

Human exposure to electromagnetic fields (EMF) comes from many different sources and occurs in various situations in everyday life. Man-made static fields are mainly found in occupational settings, such as close to MRI scanners, although DC high-voltage overhead transmission lines are being constructed, which are expected to expose larger parts of the population to static electric and magnetic fields.

EMF in the extremely low frequency (ELF) range are ubiquitous. The main sources of these fields pertaining to the general public are in-house installations, household appliances and powerlines. In recent years, attention has also been directed towards people living next to electric power transformers installed inside residential buildings. It appears that long-term exposure to ELF magnetic field of these people can extent to several tenths of μT.

Today, for power regulation most modern electrical equipment uses electronics instead of transformers. Examples include the switched power supplies to laptops, drilling tools, chargers of mobile phones and similar devices. As a consequence, the frequency content of the daily magnetic field exposure has changed mainly by adding odd harmonics. In particular, the third harmonic (150 Hz) has become another dominating frequency in our environment.

In the household, more appliances have appeared in the intermediate frequencies (IF) range. An important source of exposure in this frequency range is induction hobs, which have become popular in recent years. These can expose their users (both members of the general public and professionals) to IF magnetic fields higher than the reference levels of exposure guidelines.

In the radio frequency (RF range), by far the most applications which emit EMF are in the frequency range above 100 kHz up to some GHz. Multiple sources exist that contribute to an individual’s exposure. However, transmitters in close vicinity to or on the body have become the main sources of exposure for the general population and professionals. Distance to the source is the main determinant of exposure, together with emitted power and duty factor.

In particular for brain tissues, the mobile phone used at the ear remains the main source of exposure. However, since the first generation of mobile telephony, the technology aimed at reducing the emitted power of mobile handsets. Digital Enhanced Cordless Telecommunications (DECT) phones are another source of everyday exposure.

Smart-phones, which operate within networks of different technologies, as well as other portable wireless devices, like tablets and laptop computers, increased the complexity of the user’s exposure and changed the exposed body region. Due to the different sources used next to the body, it is important to take into account multiple exposures for risk assessment, which may also require organ-specific dosimetry. This issue is also important for occupational exposure, since there may be situations, such as working in an MRI suite, where professionals are exposed simultaneously to EMF of multiple frequencies ranges, different temporal variations and field strengths.

The environmental exposure from sources is dominated by broadcasting antennas, antennas from private and governmental telecommunication services and mobile communications base stations. Historical data from spot measurement campaigns and

continuous radiation monitoring systems indicate that the introduction of new mobile telecommunication technologies after the deployment of the GSM and UMTS systems did not substantially change the average levels of EMF in the environment. At the same time, other technologies, like digital broadcasting, have in some regions contributed to the reduction of EMF exposure from far field sources.

The number of sources has increased indoors. The installation of access points and short range base stations, such as 3G femtocells, WiFi hotspots and DECT devices, has given rise to exposure at very close distances (within 1 m), whereas farther away the emitted EMF does not exceed the common background levels. Consequently, the emitted EMF from these devices, even when combined, still results in a marginal exposure compared to reference levels of European and international guidelines. In general, it appears that, with respect to telecommunication applications, the technological trend is to use low-power emitters, closer to or on the human body, and at higher frequencies.

Millimetre wave and THz applications are expected to be available soon in various industrial environments, such as for imaging systems used for non-destructive quality control, as well as for short-range broadband telecommunications. Currently, they do not significantly affect the average exposure of the general public. These applications will operate with low power and, due to the small penetration depth of the radiation, expose only superficial tissues.

Interaction mechanisms

Several interaction mechanisms are well established. These enable extrapolation of scientific results to the entire frequency range and wide-band health risk assessment. They have been used to formulate guidelines limiting exposures to EMF in the entire frequency range from static fields to 300GHz. A number of studies proposed other candidate mechanisms. However, none that operates in humans at levels of exposure found in the everyday environment has been firmly identified and experimentally validated nor do they enable concluding on potential health risks at other exposure conditions both with regard to amplitude and/or frequency.

Health effects from THz fields

The number of studies investigating potential biological, non-thermal effects of THz fields is small, but has been increasing over recent years due to the availability of adequate sources and detectors.

In vivo studies indicate mainly beneficial effects on disorders of intravascular components of microcirculation in rats under immobilization stress, but do not address acute and chronic toxicity or carcinogenesis. *In vitro* studies on mammalian cells differ greatly with respect to irradiation conditions and endpoints under investigation. There are studies suggesting health effects of exposure, but these have not been replicated. Some theoretical mechanisms have been proposed, but there is no experimental evidence for them. Considering the expected increase in use of THz technologies, more research focusing on the effects on skin (long-term, low-level exposure) and cornea (high-intensity, short-term exposure) is recommended.

Health effects from Radiofrequency (RF) EMF

Overall, the epidemiological studies on mobile phone RF EMF exposure do not show an increased risk of brain tumours. Furthermore, they do not indicate an increased risk for other cancers of the head and neck region. Some studies raised questions regarding an increased risk of glioma and acoustic neuroma in heavy users of mobile phones. The results of cohort and incidence time trend studies do not support an increased risk for glioma while the possibility of an association with acoustic neuroma remains open. Epidemiological studies do not indicate increased risk for other malignant diseases, including childhood cancer.

The earlier described evidence that mobile phone RF EMF exposure may affect brain activities as reflected by EEG studies during wake and sleep is further substantiated by

the more recent studies. With regard to these findings, studies which aim at investigating the role of pulse modulation and which use more experimental signals, indicate that although effects on the sleep EEG are neither restricted to NREM sleep (one study also indicates effects in REM sleep) nor to the spindle frequency range. It seems that depending on the EMF signal, the theta and delta frequency range in NREM sleep can also be affected. Furthermore, half of the experimental studies looking at the macrostructure of sleep (especially those with a longer duration of exposure) also found effects, which, however, are not consistent with regard to the affected sleep parameters. Therefore, given the variety of applied fields, duration of exposure, number of considered leads, and statistical methods it is presently not possible to derive more firm conclusions.

For event-related potentials and slow brain oscillations, results are inconsistent. Furthermore, there is a lack of data for specific age groups. One study indicates that children and adolescents seem to be less affected. The previous evidence that RF exposure may affect brain activity as reported by EEG studies during both wake and sleep appears also in recent studies. However, the relevance of the small physiological changes remains unclear and mechanistic explanation is still lacking.

Overall, there is a lack of evidence that mobile phone RF EMF affects cognitive functions in humans. Studies looking at possible effects of RF fields on cognitive function have often included multiple outcome measures. While effects have been found in individual studies, these have typically been observed only in a small number of endpoints, with little consistency between studies.

Symptoms that are attributed by some people to various RF EMF exposure can sometimes cause serious impairments to a person's quality of life. However, research conducted since the previous SCENIHR Opinion adds weight to the conclusion that RF EMF exposure is not causally linked to these symptoms. This applies to the general public, children and adolescents, and to people with idiopathic environmental intolerance attributed to electromagnetic fields (IEI-EMF). Recent meta-analyses of observational and provocation data support this conclusion.

For symptoms triggered by short-term exposure to RF fields (measured in minutes to hours), the consistent results from multiple double-blind experiments give a strong overall weight of evidence that such effects are not caused by RF exposure.

For symptoms associated with longer-term exposures (measured in days to months), the evidence from observational studies is broadly consistent and weighs against a causal effect. However, it has gaps, most notably in terms of the objective monitoring of exposure.

Human studies on neurological diseases and symptoms show no clear effect, but the evidence is limited.

The previous SCENIHR Opinion concluded that there were no adverse effects on reproduction and development from RF fields at non-thermal exposure levels. The inclusion of more recent human and animal data does not change this assessment. Human studies on child development and behavioural problems have conflicting results and methodological limitations. Therefore, the evidence of an effect is weak. Effects of exposure on foetuses from mother's mobile phone use during pregnancy are not plausible owing to extremely low foetal exposure.

Studies on male fertility are of poor quality and provide little evidence.

Health effects from Intermediate Frequency (IF) EMF

There are few new studies on health effects from IF exposures in general, and no epidemiological studies have been conducted in particular. Some *in vivo* studies report on the absence of effects on reproduction and development of IF fields up to 0.2 mT in a frequency range of 20-60 kHz.

As in the previous SCENIHR Opinion, there are still too few studies available, and furthermore no epidemiological studies have been conducted. In view of the expected increase of occupational exposure to IF, studies on biomarkers and health outcomes in workers are recommended. This could be supplemented with experimental studies.

Health effects from Extremely Low Frequency (ELF) EF and MF

Overall, existing studies do not provide convincing evidence for a causal relationship between ELF MF exposure and self-reported symptoms.

The new epidemiological studies are consistent with earlier findings of an increased risk of childhood leukaemia with estimated daily average exposures above 0.3 to 0.4 µT. As stated in the previous Opinions, no mechanisms have been identified and no support is existing from experimental studies that could explain these findings, which, together with shortcomings of the epidemiological studies prevent a causal interpretation.

Studies investigating possible effects of ELF exposure on the power spectra of the waking EEG are too heterogeneous with regard to applied fields, duration of exposure, and number of considered leads, and statistical methods to draw a sound conclusion. The same is true for behavioural outcomes and cortical excitability.

Epidemiological studies do not provide convincing evidence of an increased risk of neurodegenerative diseases, including dementia, related to power frequency MF exposure. Furthermore, they show no evidence for adverse pregnancy outcomes in relation to ELF MF. The studies concerning childhood health outcomes in relation to maternal residential ELF MF exposure during pregnancy involve some methodological issues that need to be addressed. They suggest implausible effects and need to be replicated independently before they can be used for risk assessment.

Recent results do not show an effect of the ELF fields on the reproductive function in humans.

Health effects from static magnetic fields (SMF)

In most of the available *in vitro* studies, SMF above 30 µT induced effects in the cellular endpoints investigated, although in some cases the effects were transient. Gene expression was affected in all studies, with predominantly up-regulated outcomes. These new studies are consistent with the results of previous studies.

A number of studies are reporting that effects of SMF exposures occur in animals, at levels ranging from mT to T. However, since many of the findings are limited to single studies, they do not provide any firm foundation for risk assessment.

Observational studies have shown that movement in strong SMF may cause effects such as vertigo and nausea. These can be explained by established interaction mechanisms and are more likely to occur in fields above 2 T. The relevance of these effects for the health of personnel remains unclear.

Health effects from combined EMF exposure

The few available studies on combined exposure to different EMFs do not provide sufficient evidence for risk assessment.

The studies reporting on effects on DNA integrity after an MRI investigation are clearly of interest to follow up. However, it is not clear which component of the complex EMF exposure during scanning may cause the effect: SMF, switched gradient MF or the pulsed RF EMF. Further studies on DNA integrity and MRI exposure are needed, and the feasibility of cohort studies of MRI patients and occupationally exposed personnel should be discussed.

Health effects from co-exposure to environmental stressors

Experimental results reported since the previous opinion indicate that co-exposures to environmental stressors (such as physical or chemical agents) with ELF or RF lack consistency. Under the same conditions, effects might be increased, decreased or not

influenced at all and are not linked to specific experimental protocols. Due to the small number of available investigations and the large variety of protocols used (different chemical or physical treatments and different EMF exposure conditions), it is not possible to draw definitive conclusions. Therefore, the relevance of co-exposures to environmental stressors (such as physical or chemical agents) with ELF or RF to human health under real-life exposure conditions remains unclear.

Research recommendations and methodological guidance

The SCENIHR has developed a set of prioritized research recommendations and methodological guidance on the experimental design and minimum requirements to ensure data quality and usability for risk assessment. These are provided in chapters 3.14 and 3.15 of the Opinion.

Keywords: Electromagnetic fields, EMF, RF, IF, ELF, static fields, millimetre wave, THz, health effects.

Opinion to be cited as:

SCENIHR (Scientific Committee on Emerging and Newly Identified Health Risks), **Potential health effects of exposure to electromagnetic fields (EMF),** 27 January 2015.

TABLE OF CONTENTS

ABSTRACT 4

EXECUTIVE SUMMARY.. 12

1. BACKGROUND 18

2. TERMS OF REFERENCE... 20

3. SCIENTIFIC RATIONALE 21

3.1. Introduction and scope 21

3.2. Methodology 21

3.3. Exposure to EMF... 27

3.3.1. Wireless communication technologies (incl. dosimetry) 29
3.3.2. Industrial applications 38
3.3.3. Medical applications 43
3.3.4. Security applications 47
3.3.5. Power generation and transmission 48
3.3.6. Transportation 49
3.3.7. Household appliances... 50
3.3.8. THz technologies 52
3.3.9. Discussion on exposure to EMF.. 53
3.3.10. Conclusions on exposure to EMF 55
3.4. Interaction mechanisms.. 56

3.4.1. Static fields 57
3.4.2. ELF fields 57
3.4.3. RF Fields 58
3.4.4. Other mechanisms 59
3.4.5. Conclusion 63
3.5. Health effects from THz fields 63

3.5.1. *In vivo* studies 63
3.5.2. *In vitro* studies 66
3.4.3 Discussion on health effects from THz fields 71
3.4.4 Conclusion on health effects from THz fields 71
3.6. Health effects from RF fields 72

3.6.1. Neoplastic diseases.. 72
3.6.1.1. Epidemiological studies 72
3.6.1.2. *In vivo* studies 84
3.6.1.3. *In vitro* studies 86
3.6.1.4. Conclusions on neoplastic diseases from RF exposure 101
3.6.2. Nervous system effects and neurobehavioural disorders..................... 101
3.6.2.1. Epidemiological studies 101
3.6.2.2. Neurophysiological studies... 104
3.6.2.3. *In vivo* studies 127
3.6.2.4. *In vitro* studies 133

3.6.2.5. Conclusions on nervous system effects and neurobehavioral disorders ... 135
3.6.3. Symptoms 135
3.6.3.1. Provocation studies ... 135
3.6.3.2. Observational studies ... 139
3.6.3.3. Conclusions on symptoms ... 143
3.6.4. Other effects of RF exposure ... 144
3.6.4.1. Reproductive effects ... 144
3.6.4.2. Developmental effects ... 147
3.6.5. Conclusions on the health effects of exposure to RF fields ... 152
3.7. Health effects from IF fields ... 153
3.7.1. What was already known on this subject ... 153
3.7.2. What has been achieved since then ... 154
3.7.3. Conclusions on health effects from IF fields ... 155
3.8. Health effects from ELF fields ... 155
3.8.1. Neoplastic diseases ... 155
3.8.1.1. Epidemiological studies ... 155
3.8.1.2. *In vivo* studies ... 159
3.8.1.3. *In vitro studies* ... 161
3.8.1.4. Conclusions on neoplastic diseases ... 164
3.8.2. Nervous system effects and neurobehavioral disorders ... 164
3.8.2.1. Epidemiological studies ... 164
3.8.2.2. Neurophysiological studies ... 166
3.8.2.3. *In vivo* studies ... 169
3.8.2.4. *In vitro* studies ... 179
3.8.2.5. Conclusions on nervous system effects and neurobehavioral disorders ... 180
3.8.3. Other effects of ELF fields exposure ... 181
3.8.3.1. Symptoms ... 181
3.8.3.2. Reproductive effects ... 184
3.8.3.3. Effects of foetal exposure to ELF on children's health ... 185
3.8.4. Conclusions on health effects from ELF fields ... 186
3.9. Health effects from Static Fields including MRI exposure ... 187
3.9.1. Human studies ... 187
3.9.2. Animal studies ... 190
3.9.3. *In vitro* studies ... 194
3.9.4. Conclusion on health effects from SMF exposure ... 199
3.10. Health effects from combined exposure to different EMF ... 199
3.10.1. *Combined exposure in Magnetic Resonance Imaging (MRI) environment* 199
3.10.2. *Combined exposure to RF* ... 201
3.10.3. *Combined exposures to different EMFs* ... 203
3.11. Health effects from co-exposure to other agents ... 204
3.11.1. Animal studies ... 204

3.11.2. *In vitro* studies 209
3.11.3. Conclusions on health effects from co-exposure to EMF and other agents 217
3.12. EMF effects on implanted medical devices 217
3.13. Research recommendations 218
3.13.1. Static fields including MRI exposure 219
3.13.2. ELF fields 219
3.13.3. IF fields 220
3.13.4. RF fields 220
3.13.5. THz technologies 221
3.13.6. Combined exposures to EMF 222
3.13.7. Co-exposure with other agents... 222
3.13.8. Exposure assessment.. 222
3.14. Guidance on research methods... 223
4. OPINION.. 225
5. MINORITY OPINION.. 228
6. CONSIDERATION OF THE RESPONSES RECEIVED DURING THE CONSULTATION PROCESS 229
7. GLOSSARY 230
8. REFERENCES 233

EXECUTIVE SUMMARY

Introduction

The purpose of this Opinion is to update the SCENIHR Opinions of 19 January 2009 'Health effects of exposure to EMF' and 6 July 2009 'Research needs and methodology to address the remaining knowledge gaps on the potential health effects of EMF' in the light of newly available information, and to give special consideration to areas where important knowledge gaps were identified in the previous Opinions. In addition, biophysical interaction mechanisms and the potential role of co-exposures to environmental stressors are discussed.

Information has primarily been obtained from reports published in international peer-reviewed scientific journals in the English language. Additional sources of information have also been considered, including web-based information retrieval and documents from governmental bodies and authorities. SCHENIR 'Memorandum on the use of the scientific literature for human health risk assessment purposes – weighing of evidence and expression of uncertainty' 2012, was followed.

Not all identified studies are necessarily included in the Opinion. On the contrary, a main task is to evaluate and assess the articles and the scientific weight that is to be given to each of them. Only studies that are considered relevant for the task are commented upon in the Opinion. Nevertheless, all identified studies are listed in the annex.

A specific concern in the assessment of many studies is the description of the exposure. This applies to experimental as well as to epidemiological studies. Over time, many studies have reported biological effects as a result of EMF exposure. However, in many cases the description of the exposure is insufficient for reproducing the experiment. Papers with poor description of essential data, such as the exposure, are therefore of little or no value in risk evaluation and do not provide adequate knowledge about modes of actions. In the last few years there have been a number of *in vivo* and *in vitro* studies dealing with exposure directly from a commercial mobile phone or other wireless device. In almost all cases these experiments are without relevance, since they do not quantify the factual exposure.

An epidemiological study should ideally capture all major sources of exposure as a function of time during the relevant time period (considering latency) prior to occurrence of the outcome. The minimum requirement for exposure assessment for an epidemiological study to be informative is to include reasonably accurate individual exposure characterization over a relevant period of time capturing all major sources of exposure for the pertinent part of the body. Valid exposure assessment makes it possible to distinguish between sub-groups with contrasting exposure levels. As EMF exposure is ubiquitous, it is difficult to find an unexposed reference group, and instead, a quantitative contrast is chosen by comparing low versus high exposure levels.

In general, personal exposimetry is regarded as the gold standard for assessment of current short-term exposure, because spot measurements may not adequately reflect long-term exposure. For studies on health risks from EMF, depending on the investigated endpoint, the relevant time period for which exposure data would be needed is a sufficiently long period, such as several years preceding the diagnosis of cancer. As a rule, retrospective exposure assessment is more challenging and prone to errors than estimation of concurrent exposures. Estimates from study subjects are rarely a reliable source of information, due to potential errors in recall, particularly for case-control studies. More objective sources of information should be used wherever possible.

In research on health effects of EMF, the lack of clearly focused working hypotheses for chosen biological endpoints is accentuated by the lack of an established biological or biophysical mechanism of action at environmental exposure levels. This does not allow researchers to conclude on the most relevant exposure parameter, and usually several alternative measures of exposure are evaluated (for instance field strength, exposure

frequency, cumulative exposure, time since first exposure etc.). In addition, some studies use multiple end-points which are equally prone to false positive results, without adequate statistical corrections. Good research practice requires that all hypotheses evaluated are clearly stated and that all results pertaining to them are reported. Selective reporting, with emphasis on significant findings that were not specified in advance, can mislead the assessment by ignoring the issue of multiple testing.

Exposure

Human exposure to EMF comes from many different sources and occurs in various situations in everyday life. Man-made static fields are mainly found in occupational settings, such as close to MRI scanners, although DC high-voltage overhead transmission lines are being constructed, which are expected to expose larger parts of the population to static electric and magnetic fields.

EMF in the ELF range are ubiquitous. The main sources of these fields pertaining to the general public are in-house installations, household appliances and powerlines. In recent years, attention has also been directed towards people living next to electric power transformers installed inside residential buildings. It appears that long-term exposure to ELF magnetic field of these people can extent to several tenths of μT.

Today, for power regulation most modern electrical equipment uses electronics instead of transformers. Examples include the switched power supplies to laptops, drilling tools, chargers of mobile phones and similar devices. As a consequence, the frequency content of the daily magnetic field exposure has changed mainly by adding odd harmonics (150 Hz, 250 Hz, 750 Hz, etc.). In particular, the third harmonic (150 Hz) has become another dominating frequency in our environment.

In the household, more appliances have appeared in the intermediate frequencies (IF) range. It was found that at close range, some of them, including playthings, can exceed the reference levels set by exposure guidelines. An important source of exposure in this frequency range is induction hobs, which have become popular in recent years. These can expose their users (both members of the general public and professionals) to IF magnetic fields higher than the reference levels of exposure guidelines, mainly due to the fact that their safety standard requires conformity at a distance of 0.3 m only, and does not account for all the different modes and (worst case) use conditions.

By far the most applications which emit EMF are in the frequency range above 100 kHz up to some GHz. Multiple sources exist that contribute to an individual's exposure. However, transmitters in close vicinity to or on the body have become the main sources of exposure for the general population and professionals. Distance to the source is the main determinant of exposure, together with emitted power and duty factor.

In particular for brain tissues, the mobile phone used at the ear remains the main source of exposure. However, since the first generation of mobile telephony, the technology aimed at reducing the emitted power of mobile handsets. In particular, for GSM systems, already the introduction of dynamic power control reduced the average output power to about 50% of its rated value during calls, whereas the use of discontinuous transmission (DTX) during voice calls gave a further 30% reduction in average emitted power. Adaptive power control became faster and more effective in the third-generation (3G) of mobile telephony systems leading to a further reduction (by about two orders of magnitude) in the specific absorption Specific energy Absorption Rate (SAR) compared to GSM phones. In addition, hands-free kits reduce the energy absorbed by the head drastically. DECT phones are another source of everyday exposure.

Smart-phones, which operate within networks of different technologies, as well as other portable wireless devices, like tablets and laptop computers, have added complexity to the user's exposure and changed the exposed body region. Due to the different sources used next to the body, it is important to take into account multiple exposure for risk assessment, which may also require organ-specific dosimetry. This issue is also important for occupational exposure, since there may be situations, such as working in

an MRI suite, where professionals are exposed simultaneously to EMF of multiple frequencies ranges, different temporal variations and field strengths.

The exposure from environmental sources is dominated by broadcasting antennas, antennas from private and governmental telecommunication services and mobile communications base stations. It has been shown that such systems have significantly increased the EMF levels in the urban environment compared to the levels measured during the 1980's, when only analogue radio and television broadcasting were present. However, historical data from spot measurement campaigns and continuous radiation monitoring systems indicate that the introduction of new mobile telecommunication technologies after the deployment of the GSM and UMTS systems did not substantially change the average levels of EMF in the environment. At the same time, other technologies, like digital broadcasting, have in some regions contributed to the reduction of EMF exposure from far field sources.

The number of sources has increased indoors. The installation of access points and short range base stations, such as 3G femtocells, WiFi hotspots and DECT devices, has given rise to exposure at very close distances (within 1 m), whereas farther away the emitted EMF does not exceed the common background levels. Consequently, the emitted EMF from these devices, even when combined, still results in a marginal exposure compared to reference levels of European and international guidelines. In general, it appears that, with respect to telecommunication applications, the technological trend is to use low-power emitters, closer to or on the human body, and at higher frequencies.

Millimetre wave and THz applications are expected to be available soon in various industrial environments, such as for imaging systems used for non-destructive quality control, as well as for short-range broadband telecommunications. Currently, they do not significantly affect the average exposure of the general public. These applications will operate with low power and, due to the small penetration depth of the radiation, expose only superficial tissues.

Interaction mechanisms

Several interactions mechanisms are well established. They allow extrapolation of scientific results to the entire frequency range and wide-band health risk assessment. They have been used to formulate guidelines limiting exposures to EMF in the entire frequency range from static fields to 300GHz. A number of studies reported other candidate mechanisms. However, none that operates in humans at levels of exposure found in the everyday environment has been firmly identified and experimentally validated nor do they allow concluding on potential health risks at other exposure conditions both with regard to amplitude and/or frequency.

Health effects from THz fields

The number of studies investigating potential biological, non-thermal effects of THz fields is small, but has been increasing over recent years, due to the availability of adequate sources and detectors.

In vivo studies indicate mainly beneficial effects on disorders of intravascular components of microcirculation in rats under immobilization stress, but do not address acute and chronic toxicity or carcinogenesis. *In vitro* studies on mammalian cells differ greatly with respect to irradiation conditions and endpoints under investigation. Studies suggesting effects of exposure have not been replicated in independent laboratories. Some theoretical mechanisms have been proposed, but no conclusive experimental support is available.

Considering the expected increase in use of THz technologies, more research focusing on the effects on skin (long-term, low-level exposure) and cornea (high-intensity, short-term exposure) is recommended.

Health effects from RF fields

Overall, the epidemiological studies on mobile phone RF EMF exposure do not show an increased risk of brain tumours. Furthermore, they do not indicate an increased risk for other cancers of the head and neck region. Some studies raised questions regarding an increased risk of glioma and acoustic neuroma in heavy users of mobile phones. The results of cohort and incidence time trend studies do not support an increased risk for glioma while the possibility of an association with acoustic neuroma remains open. Epidemiological studies do not indicate increased risk for other malignant diseases including childhood cancer.

A considerable number of well-performed *in vivo* studies using a wide variety of animal models have been mostly negative in outcome.

A large number of *in vitro* studies pertaining to genotoxic as well as non-genotoxic end-points have been published since the last Opinion. In most of the studies, no effects of exposure at non-thermal levels were reported, although in some cases DNA strand breaks and mitotic spindle disturbances were observed.

The earlier described evidence that RF exposure may affect brain activities as reflected by EEG studies during wake and sleep is further substantiated by the more recent studies. With regard to these findings, studies which aim at investigating the role of pulse modulation and which use more experimental signals, indicate that although effects on the sleep EEG are neither restricted to NREM sleep (one study indicates effects also in REM sleep) nor to the spindle frequency range. It seems that depending on the EMF signal the theta and delta frequency range in NREM sleep can also be affected. Furthermore, half of the experimental studies looking at the macrostructure of sleep (especially those with a longer duration of exposure) also found effects, which, however, are not consistent with regard to the affected sleep parameters. Therefore, given the variety of applied fields, duration of exposure, number of considered leads, and statistical methods it is presently not possible to derive more firm conclusions.

For event-related potentials and slow brain oscillations results are inconsistent. Furthermore, there is a lack of data for specific age groups. One study indicates that children and adolescents seem to be less affected. Therefore, the previous evidence that RF exposure may affect brain activity as reported by EEG studies during both wake and sleep appears also in recent studies. However, the relevance of the small physiological changes remains unclear and mechanistic explanation is still lacking.

Overall, there is a lack of evidence that RF EMF affects cognitive functions in humans. Studies looking at possible effects of RF fields on cognitive function have often included multiple outcome measures. While effects have been found by individual studies, these have typically been observed only in a small number of endpoints, with little consistency between studies.

Symptoms that are attributed by some people to RF EMF exposure can sometimes cause serious impairments to a person’s quality of life. However, research conducted since the previous SCENIHR Opinion adds weight to the conclusion that RF EMF exposure is not causally linked to these symptoms. This applies to the general public, children and adolescents, and to people with idiopathic environmental intolerance attributed to electromagnetic fields (IEI-EMF). Recent meta-analyses of observational and provocation data support this conclusion.

For symptoms triggered by short-term exposure to RF fields (measured in minutes to hours), the consistent results from multiple double-blind experiments give a strong overall weight of evidence that such effects are not caused by RF exposure.

For symptoms associated with longer-term exposures (measured in days to months), the evidence from observational studies is broadly consistent and weighs against a causal effect. However, it has gaps, most notably in terms of the objective monitoring of exposure.

Human studies on neurological diseases and symptoms show no clear effect, but the evidence is limited.

The previous SCENIHR Opinion concluded that there were no adverse effects on reproduction and development from RF fields at non-thermal exposure levels. The inclusion of more recent human and animal data does not change this assessment. Human studies on child development and behavioural problems had conflicting results and methodological limitations. Therefore, the evidence of an effect is weak. Effects of exposure on foetuses from mother's mobile phone use during pregnancy are not plausible owing to extremely low foetal exposure.

Studies on male fertility are of poor quality and provide little evidence.

Health effects from IF fields

There are few new studies on health effects from IF exposures in general, and no epidemiological studies have been conducted in particular. Some *in vivo* studies report on the absence of effects on reproduction and development of IF fields up to 0.2 mT in a frequency range of 20-60 kHz. In view of the expected increase of occupational exposure to IF EMF, studies on biomarkers and health outcomes in workers, which are based on reasonably sized groups with well-characterized exposure, would be informative. This could be supplemented with experimental studies.

Health effects from ELF fields

Overall, existing studies do not provide convincing evidence for a causal relationship between ELF MF exposure and self-reported symptoms.

The new epidemiological studies are consistent with earlier findings of an increased risk of childhood leukaemia with estimated daily average exposures above 0.3 to 0.4 µT. As stated in the previous Opinions, no mechanisms have been identified and no support is existing from experimental studies that could explain these findings, which, together with shortcomings of the epidemiological studies prevent a causal interpretation.

As concluded in the previous SCENIHR Opinion, data suggest that ELF MF may induce both genotoxic and other biological effects *in vitro* at magnetic flux densities of about 100 µT and higher. The mechanisms are not established and the relevance for a connection between ELF MF exposure and childhood leukaemia is unclear.

Studies investigating possible effects of ELF exposure on the power spectra of the waking EEG are too heterogeneous with regard to applied fields, duration of exposure, number of considered leads and statistical methods to draw a sound conclusion. The same is true for behavioural outcomes and cortical excitability.

Epidemiological studies do not provide convincing evidence of an increased risk of neurodegenerative diseases, including dementia, related to ELF MF exposure. Furthermore, they show no evidence for adverse pregnancy outcomes in relation to ELF MF. The studies concerning childhood health outcomes in relation to maternal residential ELF MF exposure during pregnancy involve some methodological issues that need to be addressed. They suggest implausible effects and need to be replicated independently before they can be used for risk assessment.

Recent results do not show that ELF fields have any effect on the reproductive function in humans.

Health effects from static magnetic fields (SMF)

In most of the available *in vitro* studies, SMF above 30 µT induced effects in the cellular endpoints investigated, although in some cases the effects were transient. Gene expression was affected in all studies, with predominantly up-regulated outcomes. These new studies are consistent with the results of previous studies.

A number of studies report that effects of SMF exposures occur in animals, at levels ranging from mT to T. However, since many of the findings are limited to single studies, they do not provide any firm foundation for risk assessment.

Observational studies have shown that movement in strong SMF may cause effects, such as vertigo and nausea. These can be explained by established interaction mechanisms and are more likely to occur in fields above 2 T. The relevance of these effects for the health of personnel remains unclear.

Health effects from combined exposure to different EMFs

The few available studies on combined exposure to different EMFs do not provide sufficient evidence for risk assessment.

It is clearly of interest to follow up on studies concerning the effects on DNA integrity after an MRI investigation. However, it is not clear which component of the complex EMF exposure during scanning may cause the effect: SMF, switched gradient MF or the pulsed RF EMF. Further studies on DNA integrity and MRI exposure are needed and the feasibility of cohort studies of MRI patients and occupationally exposed personnel should be discussed.

Health effects from co-exposure to environmental stressors

Experimental results reported since the previous opinion indicate that co-exposures of environmental stressors (such as physical or chemical agents) with ELF or RF lack consistency. Under the same conditions, effects might be increased, decreased or not influenced at all and are not linked to specific experimental protocols. Due to the small number of available investigations and the large variety of protocols used (different chemical or physical treatments and different EMF exposure conditions), it is not possible to draw definitive conclusions. Therefore, the relevance of co-exposures of environmental stressors (such as physical or chemical agents) with ELF or RF to human health under real-life exposure conditions remains unclear.

1.BACKGROUND

Council Recommendation of 12 July 1999[1] on the limitation of exposure of the general public to electromagnetic fields (0 Hz to 300 GHz) fixes basic restrictions and reference levels for the exposure of the general public to electromagnetic fields (EMFs). These restrictions and reference levels are based on the guidelines published by the International Commission on Non Ionizing Radiation Protection in 1998 (ICNIRP)[2]. In response to the Council Recommendation, all Member States have implemented measures to limit the exposure of the public to EMF, either by implementing the provisions proposed by the Council Recommendation, or by implementing more stringent provisions[3].

For workers, the Council and the Parliament have adopted Directive 2004/40/EC of 29 April 2004[4] on the minimum health and safety requirements regarding the exposure of workers to the risks arising from physical agents (EMFs). However, in October 2007, the European Commission announced the postponement of the implementation of this Directive in order to allow enough time to prepare a modified text to better take into account research findings on the possible impact of the exposure limits on magnetic resonance imaging (MRI). The new Directive on the minimum health and safety requirements regarding the exposure of workers to the risks arising from physical agents (electromagnetic fields) and repealing Directive 2004/40/EC was issued on 26 June 2013 (Directive 2013/35/EU)[5].The Council Recommendation also invites the Commission to "*keep the matters covered by this recommendation under review, with a view to its revision and updating, taking into account possible effects, which are currently the object of research, including relevant aspects of precaution*". The ICNIRP guidelines were endorsed by the Scientific Steering Committee (SSC)[6] in its Opinion on health effects of EMFs of 25–26 June 1998. The Scientific Committee on Toxicity, Ecotoxicity and the Environment (CSTEE) prepared an update of the Scientific Steering Committee's Opinion and concluded in its Opinion on "Possible effects of Electromagnetic Fields (EMF), Radio Frequency Fields (RF) and Microwave Radiation on human health", of 30 October 2001, that the information that had become available since the SSC Opinion of June 1999 did not justify revision of the exposure limits recommended by the Council[7]. The Opinions delivered by the SCENIHR in March 2007[8], January 2009[9] and July 2009[10] confirmed the earlier conclusion of the CSTEE and again highlighted the need for additional data and research on this issue and recommended that specific research areas be addressed.

The Commission relies on the SCENIHR to periodically review new information that may influence the assessment of risks to human health in this area and to provide regular updates on the scientific evidence base to the Commission.

---

[1] (OJ. L 199/59, 30.7.1999)

[2] http://www.icnirp.de/

[3] http://ec.europa.eu/health/electromagnetic_fields/role_eu_ms/index_en.htm

[4] (OJ. L 184/1, 24.5.2004)

[5] http://eur-lex.europa.eu/LexUriServ/LexUriServ.do?uri=OJ:L:2013:179:0001:0021:EN:PDF

[6] http://europa.eu.int/comm/food/fs/sc/ssc/index_en.html

[7] The main frequencies in the ELF frequency range are 50 Hz in Europe and 60 Hz in North America. The RF and lower microwave frequencies are of particular interest for broadcasting, mobile telephony. The 2.45 GHz frequency is mainly used in domestic and industrial microwave ovens.

[8] http://ec.europa.eu/health/ph_risk/committees/04_scenihr/docs/scenihr_o_007.pdf

[9] http://ec.europa.eu/health/ph_risk/committees/04_scenihr/docs/scenihr_o_022.pdf

[10] http://ec.europa.eu/health/ph_risk/committees/04_scenihr/docs/scenihr_o_024.pdf

Since September 2008, the cut-off date for the previous review by the SCENIHR, a sufficient number of new scientific publications have appeared to warrant a new analysis of the scientific evidence on possible effects on human health of exposure to EMF. In addition, the development of new technologies using EMF in the THz range, especially imaging techniques such as security scanners for passenger screening, calls for new assessments.

On 16-17 November 2011, the International Conference on EMF and Health, organized by the European Commission under the auspices of the SCENIHR, provided an overview of the most recent scientific developments in this area as the initial preparation for a future Scientific Opinion.

Consequently, the SCENIHR is being asked to examine this new scientific evidence and to address in particular the questions listed in the Terms of Reference.

2. TERMS OF REFERENCE

The Committee is requested:

1. To update its Opinions of 2009[9, 10] in the light of newly available information.

2. To give particular attention to issues affected by important gaps in knowledge in the previous Opinions, especially:

- the potential adverse effects of EMF on the nervous system, including neuro-behavioural disorders, and on the risk of neo-plastic diseases;
- the understanding of biophysical mechanisms that could explain observed biological effects and epidemiological associations; and
- the potential role of co-exposures with other environmental agents in biological effects attributed to EMF.

3. To review the scientific evidence available to understand the potential adverse health effects of EMF in the THz range.

4. To develop a set of prioritized research recommendations updating previous efforts in this area (in particular by the SCENIHR and the WHO). These recommendations should include methodological guidance on the experimental design and minimum requirements to ensure data quality and usability for risk assessment.

3.SCIENTIFIC RATIONALE

3.1. Introduction and scope

The purpose of this Opinion is to update the SCENIHR Opinion of 19 January 2009 in the light of newly available information and to give special consideration to areas where important knowledge gaps were identified in the previous Opinion. In addition, biophysical interaction mechanisms and the potential role of co-exposures to environmental stressors are discussed. In order to update the Opinion, this section establishes the scientific rationale that is needed to provide the requested Opinion. Relevant scientific knowledge from the physical, engineering, medical and biological sciences is critically evaluated and summarised. When appropriate, gaps in knowledge are highlighted and suggestions for future important areas of research are included.

As in the previous Opinions, the section is divided into separate sub-sections based on frequency bands: (radio frequency (RF) (100 kHz < f ≤ 300 GHz), intermediate frequency (IF) (300 Hz < f ≤ 100 kHz), extremely low frequency (ELF) (0 < f ≤ 300 Hz), and static (0 Hz) (only static magnetic fields are considered in this Opinion). These frequency ranges are discussed in order of decreasing frequency: RF, IF, ELF, and static fields, respectively. For each frequency range, the review begins with a summary of the findings in the previous Opinion. This is followed, for each frequency range, by a discussion that is organised according to outcome. For each outcome, relevant human, *in vivo* and *in vitro* data are covered.

This Opinion also discusses a part of the radio frequency spectrum that is the lower Terahertz (THz) range. Terahertz applications operate between the optical spectrum on the short wavelength side and the radio frequency fields on the longer wavelength side. Applications are mainly imaging and spectroscopy.

There are also frequency bands that are not covered in this Opinion since relevant data regarding possible effects on human health are not available, or not directly mentioned in the mandate. Parts of the electromagnetic spectrum that are not discussed include the infrared and ultraviolet frequency bands.

Throughout this Opinion, the terms "positive", "negative", and "uninformative" are used to describe studies. A "positive" study refers to a study where an effect of EMF is shown, with valid methods described in enough detail to constitute evidence supporting the study hypothesis. If a well-conducted and appropriately reported study shows no clear effect despite proper methods and statistical power, its results provide evidence against the study hypothesis (but support the null hypothesis), and the study is considered "negative". Studies with insufficient information on the methodology or inadequate statistical power or flawed study design (or methods) are regarded as "uninformative". Furthermore, SI-units are consistently used throughout the Opinion.

3.2. Methodology

Information has been obtained primarily from original research papers published in international peer-reviewed scientific journals in the English language. This includes meta-analyses but not reviews. Additional sources of information have also been considered, including web-based information retrieval, and documents from Governmental bodies and authorities.

For most of the sections in the Scientific Rationale, scientific reports published after the publication of the previous SCENIHR Opinion (SCENIHR 2009) have been considered. In practice, the present Opinion thus covers studies that were published between 2009 and June 2014. Certain sections in the Scientific Rationale were not covered in our previous SCENIHR Opinions. In such cases, publications published before 2009 have also been included in the assessment.

Not all identified studies are necessarily included in the Opinion. On the contrary, a main task is to evaluate and assess the articles and the scientific weight that is to be given to

each of them. Detailed criteria for selecting these studies have been published in the SCENIHR Memorandum "Use of the scientific literature for risk assessment purposes – a weight of evidence approach" (SCENIHR 2012). Additional criteria specifically for studies of EMF health effects were also listed in a previous SCENIHR Opinion (SCENIHR 2009). Although anecdotal evidence can be valuable for highlighting an area of concern and thus initiating scientific studies, this kind of evidence has not been considered in the assessments performed in this Opinion.

In some areas where the literature is particularly scarce, it has been considered important to explain why the results of certain studies do not add useful information to the database. Identified reports that have not been considered in the Opinion are listed under the subheading "Literature identified but not cited" in the References section.

Exposure considerations

A specific concern in the assessment of many studies is the description of the exposure. This is true for experimental as well as for epidemiological studies. Over time, many studies have reported biological effects after EMF exposure. However, the description of the exposure is in many cases not sufficient even for scientists with relevant knowledge and the proper equipment to reproduce the experiment. Papers with poor descriptions of the exposure are therefore of little or no value in risk evaluation and do not provide knowledge about modes of actions. Valberg (1995) and Kaune (1995) have listed up to 18 parameters that need to be considered in ELF MF *in vivo* and *in vitro* experiments, which fall into five major categories: a) exposure intensity and timing, b) frequency-domain characteristics, c) spatial (geometric) descriptors, d) combined EMF exposure, and e) characteristics of the exposure system. The same considerations are also valid for experimental work in other frequency areas. Omission of many EMF exposure parameters causes considerable difficulty for others to replicate the experiment and interpret the reported EMF bioeffects.

An example where important exposure details are commonly missing is an *in vitro* experiment with cells in a Petri dish. If a magnetic field is applied vertically it will induce an electric field that is strongest at the periphery of the dish, and approaching zero in the centre of the dish. On the other hand, if the field is applied horizontally the induced E field will in most cases be much smaller and also uneven in a different way. It is important to know these details in order to tell if any effect is due to the magnetic field itself or to an induced E field.

Another factor of importance in *in vitro* experiments is the background magnetic field in cell culture incubators. It has been shown by Hansson Mild et al. (2009) and Portelli et al. (2013) that values up to some tens of μT are common and the distribution within the incubator is very inhomogeneous. Needless to say, if the performed experiments are investigating MF-effects at similar flux densities, the relevance of the experiment is doubtful.

Recently, Zeni and Scarfi (2012) discussed the requirements for *in vitro* studies with RF exposure. Just as in the ELF situation, there are many parameters to take into consideration, and experiments without proper dosimetry are not useful in risk evaluation or other interpretations.

In the last few years there have been a number of *in vivo* and *in vitro* studies dealing with exposure directly from a mobile phone. In almost all cases these experiments are without relevance since they do not mention anything about the factual exposure. They are also impossible to reproduce in another laboratory. Thus, there are studies where a mobile phone is placed next to or under a Petri dish, or under a cage of animals, and connected to another phone. Such a set-up does not allow for proper dosimetry as many unknown factors can influence the exposure that is produced. These include the distance to the phone's base station, the output power, the SAR distribution of the phone, whether the DTX function was activated, and the frequency used by the phone. These experiments are therefore best carried out with a special exposure set-up. More detailed

advice for proper procedures regarding *in vitro* studies of EMF effects are given in Zeni and Scarfi (2012) and Paffi et al. (2010).

Considerations for epidemiology

An epidemiological study should ideally capture all major sources of exposure as a function of time during the relevant time period (considering latency) prior to occurrence of the outcome. For exposures from environmental and occupational sources, as well as from personal use of devices, comprehensive construction of exposure history requires evaluation of exposure as a function of time. For RF, personal use of mobile phones and DECT is the predominant source of exposure for the vast majority of the population, followed by occupational exposure for certain subgroups and by presence of wireless devices, base stations and similar sources in residential and other daily settings. For ELF, consideration of residential exposure from nearby power lines, wiring within the home and some occupational exposures are essential.

In general, personal measurements are regarded as the gold standard for assessment of current short-term exposure, though spot measurements may not reflect long-term exposure. For studies on health risks from EMF, the relevant time period for which exposure data would be needed is a period of perhaps several years preceding the diagnosis. Typically, exposure assessment only encompasses either a short-term measurement of a maximum of 48 hours with personal monitoring, or a spot measurement providing only a snapshot of instantaneous exposure levels at a single location (while the former can more widely cover the places where exposure occurs, such as work or school, and hence provide a more realistic picture of typical exposures). As a rule, retrospective exposure assessment is more challenging and prone to errors than estimation of concurrent exposures. Long-term exposure from some key sources such as power lines, TV/radio transmitters or base stations can also be retrospectively reconstructed if adequate information on the system is available (voltages for power lines, power levels, directions and shielding for transmitters and base stations). Study subjects are rarely an optimal source of information due to potential errors in recall, particularly for case-control studies. More objective sources of information include records such as monitoring reports, e.g. operator records for call time in mobile phone studies (provided that both in-coming and out-going calls are registered). Various proxy measures as indirect indicators of exposure are commonly employed, such as job title for occupational exposure. Their validity depends on variability of exposure for subjects with similar occupations – the wider the exposure distribution, the higher the misclassification.

Exposure assessment should provide adequate temporal and spatial resolution. The focus should be on the relevant part of the body (target tissue). Mobile phone use is important for local exposure in the head and neck area, but far-field exposures are (likely to be) more important for other parts of the body. For instance, maternal mobile phone use is likely to be inappropriate as an indicator of RF-EMF exposure to the foetus in studies on developmental outcomes or the testis in sperm quality studies. Estimation of SAR from mobile phones in various parts of the brain (at an individual level) based on self-reported usage history is already approaching/extending the limits of the resolution achievable from such data.

The minimum requirements for exposure assessment for an epidemiological study to be informative include reasonably accurate individual exposure characterization over a relevant period of time capturing all major sources of exposure for the pertinent part of the body. Valid exposure assessment allows a researcher to distinguish sub-groups of the population with contrasting exposure levels. As EMF exposure is ubiquitous, it is difficult to find an unexposed reference group and instead, a quantitative contrast is used with comparison of low versus high exposure levels.

Whatever exposure metric is used, it is important to demonstrate its adequacy for the specific study hypothesis, for instance with the help of validation studies, comparison of different metrics aimed at predicting the same exposure, or sensitivity analysis using different error scenarios. Firstly, sometimes the seemingly most appropriate or

comprehensive metric is not the best one; for example, personal dosimetry in case-control studies on cancer in children captures all exposures over a typical day, but is unlikely to be appropriate for estimating past exposure conditions, as children's daily activities change dramatically with age and daily activities of the case children are definitely influenced by having had the disease. Secondly, depending on exposure prevalence, it might be that small misclassification errors have a big impact and large misclassification errors have a small impact in the risk estimation, as the bias related to misclassification depends on the sensitivity and specificity of the metric in predicting the true exposure. For example, childhood cancer studies using calculated fields as exposure metric suffer from extra loss of statistical power; however, there is little bias in the risk estimation, because the method has very high specificity (unlikely that truly non-exposed children are classified as exposed) but has low sensitivity (likely that truly exposed children are classified as non-exposed) that hardly matters due to the low exposure prevalence. These examples clearly show the reason why, for exposure assessment in epidemiological studies, experts in epidemiology and dosimetry should team up to jointly develop the most appropriate method.

Dose

Even if the exposure assessment is carried out in a valid fashion, the problem of combining the exposure intensity with the duration of exposure into a dose measure still remains. However, the problem of dose assessment in epidemiological studies has mostly not been taken into account because no interaction mechanism(s) is (are) known regarding potential non-thermal effects of weak fields. Depending on the type of disease studied, the exposure assessment in the epidemiological studies need to be very different. For effects depending on just short term exposure – effects of more acute character – the SAR values might be the useful measures to obtain. This can be exemplified by subjective symptoms and mobile phone use. However, regarding diseases with long latencies like cancer and Alzheimer's disease, it becomes much more difficult, since then it is the exposure a number of years ago that is of interest, which may not be easy or at all possible to estimate today with any reasonable accuracy. In particular, questions about how the exposure is accumulated over many years need to be answered before the ultimate exposure assessment can be made. When calculating accumulated exposure over time, an important question is if there is a threshold under which no effect occurs, i.e. how low values should be taken into account. Intermittent exposures also provide difficult problems, such as the spacing of the repeated exposures and its relation to a possible biological reset time, i.e. when the system is fully recuperated. There are studies suggesting that repeated exposure in the minute to hour scale can be much more efficient than continuous exposure in experimental settings, but much remains to be investigated before this can be taken into account in epidemiological studies.

Issues in data analysis and reporting: Multiple comparisons and statistical significance

Statistical significance is used as a means of summarising the findings in various fields where statistical analysis is used in drawing inference from the data. Fundamentally, it indicates the agreement between the null hypothesis and the observations (empirical data). Statistical significance (p-value) is defined as the probability of observing an effect (of observed size or larger) in the absence of any true effect (called type 1 or alpha (α) error in statistics). The p-value indicates the frequency of comparable results (similar or larger effect than observed) that would occur by chance alone, i.e. under the null hypothesis. Statistical significance is calculated based on tests that pertain to the distribution of the outcome variable (e.g. a t-test for comparing two normally distributed variables, a chi-square test for frequencies etc.). A p-value is always calculated by contrasting the null hypothesis (claiming no effect) in relation to a specific finding, reflecting both sample size (number of observations), and magnitude of effect. The study hypothesis (alternative hypothesis) is a statement that assumes the existence of the proposed effect (claiming the presence of an effect of undefined size). A critical value of 0.05 for the p-value is commonly used as a threshold, with values <0.05 taken to indicate the presence of an effect (which means accepting a 5% probability of error in

case the null hypothesis is true, i.e. α or type I error). This selection is based on convention alone and can be regarded as completely arbitrary. When an important decision is to be made and erroneously accepting a chance finding would have important bearing, lower values such as 0.01 or 0.001 can be used. An appropriate interpretation of the p-value is the smaller the value, the more support the data lend for the study hypothesis. Yet the p-value alone is not a sufficient description of the study results, because it depends on both the amount of information and the size of the effect. Therefore, a study that is too small would fail to reach statistical significance even when the effect size is large enough to be meaningful. This would be termed a 'false negative' result due to insufficient statistical power. Statistical power is defined as the probability of detecting a true effect. It is usually defined in terms of type II error, β, which is the probability of not reaching statistical significance with a given effect size. Statistical power is then 1-β. Statistical significance testing is an issue in studies aimed at evaluating hypotheses. This is not the goal in all research, but estimation, i.e. quantification of the magnitude of effect (such as assessment of dose-response curve), is pursued in some studies (though typically in a situation, where the presence of an effect has already been established, for instance risk of cancer from ionising radiation).

Statistical power depends on the magnitude of the effect, probability of end-point occurrence, and sample size (or the combined effect of the three, which can be expressed as the number of excess events in the exposed group). An example of probability of detecting an effect of a given size as a function of sample size is illustrated in the Figure 1 below. The smaller the study, the larger the effect needs to be to reach statistical significance – even a substantial difference may fail to be detected. Conversely, in a very large study, even an effect of trivial size can be statistically significant. Hence, the effect size and number of events need to be indicated to allow a meaningful interpretation of the p-value, and some journals discourage use of significance tests placing more emphasis on confidence intervals as indicators of random error.

Figure 1. Required sample size to detect an effect of a given size

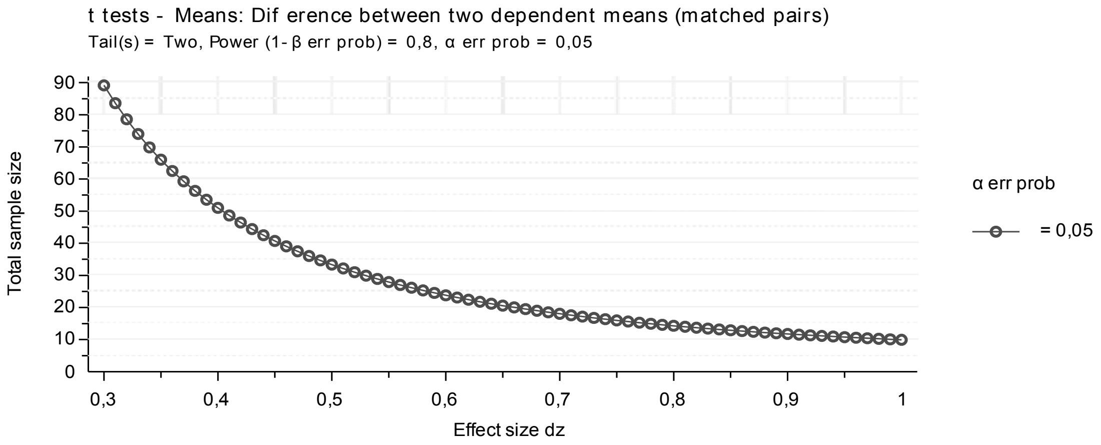

In research on health effects of EMF, the lack of clearly focused hypotheses is accentuated by the lack of an established biological or biophysical mechanism of action. This does not allow the researchers to specify mechanistically the most relevant exposure indices, but commonly several alternative measures of exposure are evaluated (for instance field strength, exposure frequency, cumulative exposure, time since first exposure etc.). In addition, some studies use multiple end-points, which are equally prone to false positive results. Neurophysiological studies also generate diverse outcome data with various aspects of brain function (with unclear pathophysiological relevance). For example, high through-put methods used in analysis of gene expression (e.g. genome-wide association studies) and other analyses are a good example of approaches that generate a wealth of data that are commonly analysed in an exploratory fashion (data mining or association mapping). In such contexts, a proportion of tests are

expected to show statistically significant results even in the absence of any true effect. For instance, when using a cut-off of 0.05 for the p-value, one out of 20 significance tests can be anticipated to be below that level and the probability of finding at least one $p<0.05$ for 10 comparisons is 40% (provided that they are based on uncorrelated data).

When a large number of significance tests are performed, avoiding false positive results (apparently significant findings arising due to chance) is an issue. For example, several neurophysiological studies have reported effects of EMF on various aspects of EEG. Especially with regard to power spectra, several tests are commonly performed (e.g. testing 0.25 Hz bin frequency bands for a range from 0.25 to 20 Hz implies 80 tests, with four test results expected to be statistically significant just by chance, given type I error frequency of 0.05). Similar difficulties are also commonly encountered in epidemiological studies of occupational exposures, for example where a wide range of job titles are evaluated. Such comprehensive evaluations are called hypothesis generating or hypothesis screening studies, as opposed to hypothesis testing, and can be regarded as exploratory studies.

Several methods have been developed for adjusting the significance level used for multiple comparisons (Kooperberg et al. 2005, Rice et al. 2008). The simplest and most commonly used is the Bonferroni correction, which is based on defining the alpha error over the entire material by setting the criterion of statistical significance as the standard (0.05), divided by the number of tests. Hence, for an analysis with 10 tests (without *a priori* defined main results), a significance level of 0.005 could be applied. Other approaches are also available, some with more refined definitions for a positive finding (for instance the Benjamini-Hochberg method incorporating also the false discovery rate, Wacholder et al. 2004). Others use a resampling procedure, such as boot strapping or Monte-Carlo simulation, or first test the overall result for evidence of heterogeneity across the hypotheses. More empirical approaches include dividing the material into a test set and a separate validation set, where only those findings supported by the initial analysis are evaluated. The inherent problem in adjusting significance levels is that a true effect is of course unaffected by the number of tests and missing an effect due to correction (false negative or type II error) is a possibility that has prompted several researchers to abandon such correction methods.

Study design can help minimise false positive findings. A key issue is selection of study size based on careful power calculation, with realistic estimates of effect size and background risk. Small studies that only have adequate statistical power for detection of extreme effects are most prone to serendipitous findings.

Good research practice requires that all hypotheses evaluated are stated and that all results pertaining to them are reported. Selective reporting, with emphasis placed on significant findings that were not specified in advance, can mislead the reader by ignoring the issue of multiple testing. In the worst cases, only the significant results are reported, and non-significant ones ignored – this would misguide the interpretation of statistical significance by obscuring the need for considering multiple testing. This inappropriate practice is called the 'Texas sharp shooter effect' ("if you want to hit the bull's eye, the best method is to shoot first and call whatever you hit the intended target"). To avoid such conscious or unconscious selection of results, detailed study protocols and analysis plans with pre-specified exposure indicators and primary outcomes are needed. Registration of randomised trials is nowadays required by several journals for this same reason. Publication of study protocols for non-randomised studies has also been suggested as a remedy to this problem (Swaen 2011, Lancet 2010).

Publication bias is a related distortion of the results reported in the literature (Dwan et al. 2008). It refers to a phenomenon whereby research in which the study hypothesis is supported by the findings is more likely to be formally reported in the peer-reviewed literature (Hopewell et al. 2009). The selective publication of results that appear to provide most support for the study hypothesis is enhanced by editorial policies focusing on the most striking findings which are likely to attain the most attention (and citations). Frequently, initial reports of effects turn out to be smaller in subsequent assessment

(known as 'winner's curse'), which reflects the role of serendipity in reporting and publication (Zollner & Pritchard 2007, Ioannidis 2008). Publication bias tends to be strongest for small studies: large, costly studies are more likely to be published regardless of their findings. Publication bias should always be evaluated in meta-analyses to assess the possibility that small studies in particular are skewed toward positive results.

Weight of evidence

A weight of evidence approach is used to assess the scientific support for a specific outcome. This is based on data from human, animal and mechanistic studies (the primary evidence) along with exposure. For each line of evidence, the overall quality of the studies is taken into account, as well as the relevance of the studies for the issue in question. The weighting also considers if causality is shown or not in the relevant studies. In the present Opinion (i.e. the Opinion text proper, the Executive Summary, and the Abstract) the following categories are used to assign the relevant weight of evidence for the specific outcomes.

Strong overall weight of evidence
- Coherent evidence from human and one or more other lines of evidence (except for symptoms where only human evidence is available); no important data gaps

Moderate overall weight of evidence
- Good evidence from a primary line of evidence (human experimental or epidemiological, animal and mechanistic studies together with exposure), but evidence from several other lines is missing (important data gaps)

Weak overall weight of evidence
- Weak evidence from primary lines of evidence, severe data gaps

Discordant overall weight of evidence
- Conflicting information from different lines of evidence

Weighing of evidence not possible
- No suitable evidence available

3.3. Exposure to EMF

Basic restrictions and reference levels

The 1999/519/EC European Council Recommendation (EC, 1999) defines, in its Annex I, the basic restrictions and reference levels for limiting exposure of the general public. This had been added by the directive 2013/35/EU on occupational exposure to EMF.

In accordance to EC (1999) and ICNIRP (1998) restrictions on exposure to time-varying electric, magnetic, and electromagnetic fields that are based directly on established health effects and biological considerations are termed 'basic restrictions'. Depending upon the frequency of the field, the physical quantities used to specify these restrictions are magnetic flux density, current density, specific energy absorption rate, and power density. Magnetic flux density and power density can be readily measured. In the latest guidelines issued by ICNIRP (2010) for limiting exposure in the frequency range of 1 Hz - 100 kHz, the internal electric field strength (the electric field inside the tissues) has been introduced to replace the electric current density as a quantity to restrict the excitation of nerve and other electrically sensitive cells.

Since many of the physical quantities used for setting the basic limits cannot be readily measured, *reference levels* are provided for practical exposure-assessment purposes to determine whether the basic restrictions are likely to be exceeded. Some reference levels are derived from relevant basic restrictions using measurements and/or computational techniques and some reference levels address perception and adverse indirect effects of exposure to EMF. The derived quantities are electric field strength, magnetic field

strength, magnetic flux density, power density, and contact current. Quantities that address perception and other indirect effects are (contact) current and, for pulsed fields, specific energy absorption. In any particular exposure situation, measured or calculated values of any of these quantities can be compared with the appropriate reference level. It should be noted that the derived reference levels (or action values) may also include additional reduction factors or be highly conservative in nature.

The field induced inside the body further depends on physical properties of the exposure configuration, such as frequency, polarization, direction of incidence, as well as on the anatomy of the exposed person, including height, posture, body mass index (BMI). Finally, the dielectric properties of tissues that change with water content and age are also important. The distribution of the field induced inside the human body at high frequencies is highly non-uniform, therefore compliance with both local and whole-body energy absorption needs to be demonstrated.

Respect of the reference level will ensure respect of the relevant basic restriction. If the measured value exceeds the reference level, it does not necessarily follow that the basic restriction will be exceeded. Under such circumstances, however, there is a need to establish whether there is respect of the basic restriction. Some quantities such as magnetic flux density and power density serve both as basic restrictions and reference levels.

Despite certain question marks regarding the potential health effects of EMF on humans in general, and on workers in particular, there is a rapid and steady development of new techniques, technologies and work practices exposing the workers and the population to a-priori advantageous electrical, electronic, wireless or wired appliances such as telephony, WiFi, electrical distribution, RFID, welding systems, galvanization, microwave applications, non-ionizing medical imaging (MRI), surgery (surgical diathermy), etc.

Much of our daily exposure to EMF, both in the workplace and for the general public, is complex and no longer consists of a single frequency, but is rather a multi-frequency exposure with different characteristics. An example is the use of wireless telephony where the phone may operate in several different modes depending on location; for instance switching between 3G and GSM modes. Welding is another example where multiple frequencies are present during the process. Workers are increasingly wearing medical implantable systems (pacemakers, insulin pumps, etc.) that are susceptible to influences from electromagnetic emitting appliances. Some interactions / interferences between bodily systems and the mentioned appliances are known, described and scientifically documented. In certain cases some of them are avoidable; other interactions with living materials remain unknown or unexplained.

The novel EU directive on occupational exposure (Directive 2013/35/EU) was initiated in 2004, but concerns about possible negative impact on the use of MRI caused some delays.

The exposure limit values for low frequency fields that are now being discussed are based as before on stimulatory effects on central and peripheral nervous systems. The values are given as limits of the internal electrical field strength, and this is then transformed into action levels given as external electric field strength and magnetic field induction. If an action value is exceeded, it does not necessarily follow that the exposure limit value is also exceeded. Under such circumstances, however, there is a need to establish whether there the exposure limit value is respected.

For the radiofrequency range the limits are given in Specific energy Absorption Rate (SAR) and follow the ICNIRP guidelines from 1998. These values are then transformed into the measurable quantities of electric and magnetic field strengths.

3.3.1. Wireless communication technologies (incl. dosimetry)

Broadcasting

Transmitters operating in the medium frequency range (300 kHz – 3 MHz) typically use monopoles as antennas, whereas in the high frequency range (HF, 3 MHz – 30 MHz) they use curtain antennas. In this lower band used for broadcasting, the transmitter power is rather large resulting in electric field strength values that are high with respect to the fields generated by other applications, even at a distance of a few hundred meters. In their measurement campaign, Mantiply et al. (1997) measured electric field strengths that varied from 2.5 to 20 V/m (magnetic field strengths from 7.7 to 76 mA/m) at 100 m away from the antenna tower of AM radio stations operating in medium frequency with powers between 1 and 50 kW. At the same distance in front of a conventional curtain antenna operating at 9.57 MHz (HF) and with 100 kW of input power, the electric and magnetic field strengths varied from 4.2 to 9.2 V/m and from 18 to 72 mA/m along the traverse respectively. As a consequence, a control zone is usually defined around such installations in which access for the general public is prohibited.

In the case of FM radio and TV broadcasting antennas, which operate in the frequency range of 80 – 800 MHz, the people exposed most are the professionals who work in the area around the antennas. The antennas in this frequency range typically have output powers of 10 – 50 kW and they take the form of dipole arrays (either horizontal or vertical) on the sides of the installation tower. Hansson Mild (1981) measured the fields at places where it is not possible to avoid RF exposure of the hands and feet while climbing the ladder of the antenna tower in an FM and TV broadcasting facility. The highest values registered were 600 V/m for the electric field strength and 3.0 A/m for the magnetic field strength; the lowest were 275 V/m and 0.9 A/m, respectively.

In most European countries analogue broadcasting systems are being replaced by digital ones, namely digital video and audio broadcasting (DVB and DAB). Although the power transmitted from digital broadcasters is lower than their analogue counterparts, a study carried out by Schubert et al. (2007) statistically analysed the electric field strength at the same locations before and after switchover from analogue to digital broadcasting. The analysis revealed an increase in mean exposure in the TV broadcasting frequency band, mostly in the central parts of Nuremberg and Munich. The maximum power density for TV broadcasting increased from 0.9 mW/m^2 to 6.5 mW/m^2 after the transition. According to the authors the main reason for this mean exposure change was the increase in the radiated power at the transmitter stations with the introduction of DVB-T. A closer examination of the results revealed that the change of the radiated power at the transmitter covering the respective regions was nearly the same as the measured exposure change and could therefore be taken as a coarse indicator for the mean change of exposure. On the contrary, the transition from analogue (FM) broadcasting to DAB led to a mean exposure reduction of 10 times in the corresponding frequency band.

In a recent study, Joseph et al. (2010a) compared the public exposure to sources in various frequency bands of the spectrum, using the data collected by personal exposure meters across five European countries (Belgium, Hungary, The Netherlands, Slovenia, Switzerland). The highest mean exposure from broadcasting was registered in office environments in Belgium for the FM frequency band and was 0.096 mW/m^2 (0.2 V/m).

Mobile phones

Table 1 lists the various mobile phone systems that have been used by the participants of the INTERPHONE study (Cardis et al., 2001). The next generations of mobile phones were expected to operate at frequency bands higher than 2 GHz. However, the transition from analogue to digital broadcasting will free a significant part of the spectrum (digital dividend), which may be reallocated to newer systems. The fourth generation (4G) of mobile phone systems in Europe is Long Term Evolution (LTE). Its main feature is fast data transmission with rates reaching up to 100 Mbps (megabits per second) downlink (from the base station to the mobile unit) and 50 Mbps uplink (from the mobile unit to the base station). Although current frequency and transmission powers of LTE mobile

phones are comparable to those for 2G and 3G handsets, in the future use may be made of higher frequency bands (beyond 2 GHz) for this technology. Furthermore, coding and modulation schemes are different in the LTE system to allow for higher data rates. The data flows into several narrow frequency bands called subcarriers, which can be switched on and off. Another important aspect of LTE is the use of MIMO (Multiple Input Multiple Output) antennas, i.e. the presence of more than one antenna on the device, so that the signal can reach the latter following different routes and thus improving the quality of service. Following the tradition in the field of mobile telephony, where about every 10 years a next generation of systems is introduced, it is expected that 5G systems will be developed by 2020 to accommodate the demand for faster communications with higher data transfer rates. In this direction, EU has funded a flagship project within FP7, the METIS project, with the objective of laying the foundation for 5G systems and building consensus prior to standardization. In order to fulfil the requirements of the test cases examined within METIS, the communicating devices must be equipped with radio access technologies at higher frequency ranges with large bandwidths. In the METIS project the highest priority for frequencies above 6 GHz was placed on frequencies between 40 and 90 GHz (Osseiran et al., 2014).

Concerning the values in Table 1, it is useful to note that the signal from most 2G terminals is pulsed. If a phone uses a TDMA (Time Division Multiple Access) technology, it transmits at regular intervals. The fraction of time that the phone transmits is given by the duty factor, i.e., a duty factor of 0.12 denotes that the phone transmits 12% of the time. The average power is calculated as the product of the maximum power with the duty factor. In the case of 3G phones (continuous transmission) the power can be up to 125 mW. This is, however, the maximum value, since in reality the output power of a mobile phone is considerably lower and is determined by the signal quality (strength). The use of Adaptive Power Control (APC) with which mobile phones reduce their output powers to allow for good signal quality gives longer life to their batteries. The network continually monitors signal quality and may reduce the emitted power of a mobile phone, by up to a factor of 1,000 for GSM and about 100,000,000 for UMTS (SCENIHR, 2009).

Table 1. Historical development of mobile telephony systems (adapted from HPA (2012) and Cardis et al. (2011)).

Generation	Start of commercial use in the region of next column	Region	System	Handset Band MHz	Base Station Band MHz	Burst duration (μs)	TDMA duty factor	Maximum emitted power (W) from handset	Average power (mW)
1	1981	Nordic countries, France, Germany	NMT-450	453.5 – 457.5	463.5 – 467.5	-	1.0	0.9 (handsets) 15 (car phone version)	900
	1986	Nordic countries	NMT-900	890 – 915	935 – 960	-	1.0	0.6 (handsets) 6 (car phone version)	600
	1985	Italy, UK	ETACS	872 – 905	917 – 950	-	1.0	0.6	600
	1989	Japan	JTACS/ NTACS	915 – 925	860 – 870	-	1.0	0.6	600
				898 – 901	843 846	-	1.0	0.6	600
				918.5 – 922	863.5 867	-	1.0	0.6	600
	1987	Japan	NTT	925 – 940	870 – 885	-	-	0.6	600
				915 – 918.5	860 – 863.5				
				922 – 925	867 – 870				
	1985	Australia, Canada, Israel and New Zealand, USA	AMPS (N-AMPS)	824 – 849	869 - 894	-	1.0	0.6	600
2	1992	Canada, Israel, New Zealand	D-AMPS / TDMA-800	824 – 849	869 – 894	6666	1/3	0.6	200
	1993	Japan	PDC-800	940 – 956	810 – 826	3333 or 6666	1/3 or 1/6	0.8	133 or 266
	1994		PDC-1500	1429 – 1465	1477 – 1513				
	2003	Canada	GSM-850	824 – 849	869 – 894	576.9	0.12	2	240
	1992	All European countries and Australia, Israel, New Zealand	GSM-900	890 – 915	935 – 960	576.9	0.12	2	240
	1993	All European countries and Australia, Israel, New Zealand	GSM-1800	1710 – 1785	1805 – 1880	576.9	0.12	1	120
	2001	Canada	PCS (GSM-1900)	1850 – 1910	1930 – 1990	576.9	0.12	1	120
	1998	Canada	TDMA-1900	1850 – 1910	1930 – 1990	6666	1/3	0.6	200
	1998	Australia, Canada, Israel, New Zealand	CDMA-800	824 – 849	869 – 894	-	1.0	0.2	200
	1998	Japan	CDMAone	830 – 840	875 – 885	-	1.0	0.2	200
	1997	Canada	CDMA-1900	1850 – 1910	1930 – 1990	-	1.0	0.2	200
3	2001	Japan and rest of the world	W-CDMA	1920 – 1980	2110 – 2170	-	1.0	0.125	125

In a multinational study (Vrijheid et al., 2009), software-modified GSM phones were distributed to more than 500 volunteers in 12 countries for 1 month each. The average output power of over 60,000 phone calls was approximately 50% of the maximum. The maximum power was used 39% of the time (on average) and was higher for rural areas. The fact that output power from mobile phones is higher in rural environments was confirmed by Persson et al. (2012), who studied the uplink power of devices in a 3G network. In an urban environment they measured an average output power of 0.4 mW (median 0.02 mW) for voice calls and 2.0 mW (median 0.2 mW) for video upload. These results are in agreement with an older study by Gati et al. (2009) who had noticed, however, that there is also a differentiation between indoor and outdoor environments, with the average output powers for voice calls in 3G systems being less than 5 mW for the former and less than 1 mW for the latter.

Mobile phones in standby mode are only active in periodic location updates, and this occurs with a frequency set by the network operator. Typical updates occur with 2 – 5 h in between. During these time intervals the phone is to be considered as a passive radio receiver with no microwave emission (Hansson Mild et al., 2012). However, modern smart phones, which can operate in several modes other than voice and SMS transmission (e.g., by staying connected to the internet for data transmission), seem to require location updates more often, thus contributing to the exposure of their users and the persons around them (Urbinello and Röösli, 2012).

In order to assess the exposure of users to mobile phones the quantity of Specific energy Absorption Rate (SAR) is used and not the electric field directly next to its antenna, because it is not possible to measure so close to the antenna without perturbing the electric field to be measured and the operation of the phone itself. SAR is measured in W/kg and is the rate of specific absorption (SA), measured in J/kg, i.e. the rate at which energy is deposited in tissue. It is assessed with measurements in human body phantoms filled with appropriate liquids, which bear dielectric properties similar to those of human tissues. Another way of estimating the SAR is to use computational techniques and numerical phantoms derived from real humans with high resolution medical imaging techniques (Christ et al., 2010; Zradziński, 2013).

During the INTERPHONE study 1,233 maximum SAR values averaged over a 10 g cube of tissue were registered (Cardis et al., 2011). They ranged from 0.01 W/kg, which is actually the sensitivity limit for measurement equipment, to 1.7 W/kg. The vast majority of values, however, were below 1 W/kg. Although not statistically significant, a trend of decreasing SAR over a period of years was clear from this study. This trend was confirmed by Kühn et al. (2013).

In epidemiological studies, cumulative specific absorption is also referred to as total cumulative specific energy and is commonly used as an exposure proxy, equivalent to dose. It is clear from the INTERPHONE study (Cardis et al., 2011) that cumulative specific absorption for the early analogue systems were manifold higher than for the next generations of handsets.

During operation, GSM mobile phones are the sources of magnetic fields at the ELF range. Perentos et al. (2007) have measured a magnetic flux density value of less than 100 μT at 217 Hz, which is the main spectral component associated with the GSM pulses, and confirmed the presence of spectral components at 2.1 and 8.3 Hz. The maximum current density induced in the head of the mobile phone user was not larger than 28% of the ICNIRP (1998) limit, according to Jokela et al. (2004) who measured the battery current pulses for seven GSM phones and calculated the exposure quotient in a simplified spherical head model. Ilvonen et al. (2005) calculated lower values of the induced current density in a realistic human head phantom in the range of some μA/m^2, i.e., about three orders of magnitude below the ICNIRP (1998) limit of 2 mA/m^2 at 217 Hz.

There are some differences in energy absorption from mobile phones between children and adults. Children's heads are smaller and, therefore, mobile phones expose a larger part of their brains. Moreover, their tissues, like bone marrow, have a higher electrical conductivity due to larger water content; therefore, local energy absorption can become higher in these tissues. Nevertheless, the peak spatial (SAR) assessed with the standardized specific anthropometric mannequin (SAM) head phantom has been shown to yield a conservative exposure estimate for both adults and children using mobile phones (Christ et al., 2010). Moreover, the value of the maximum local peak SAR in the SAM was always higher than in the adult and children models (Hadjem et al., 2010).

Mobile phone base stations

Modern communication systems are based on the division of space in 'cells' to allow for full coverage of subscribers. The coverage in each cell is provided by a base station, also called a 'relay' station in some countries, which is a transceiver serving the subscribers that are within that cell. The size of the cells can vary from several kilometres in the countryside (macrocells) to some metres inside a home (femtocells), with the respective

output power from the antennas ranging from tens of watts to as low as 5 mW. It has been shown that for macrocells distance from the base station is a bad proxy for exposure (Schüz and Mann, 2000), whereas latest studies show that for femtocells the electric field radiated by them rapidly falls off with distance to reach background radiation levels at about 1m (Boursianis et al., 2012). Moreover, the use of femtocells in homes leads to a reduction of the exposure to the mobile phone user (Zarikoff and Malone, 2013; Aerts et al., 2013).

In a recent study Rowley and Joyner (2012) analysed the data from surveys of radio base stations in 23 countries across five continents from the year 2000 onward (figure 2). They reported the immission level as a function of time (figure 3), as well as in terms of the technology (figure 4).

These figures are reproduced with permission of the Journal of Exposure Science and Environmental Epidemiology.

Figure 2. Minimum (•), maximum (•) and narrowband average (□), broadband average (□) or mixed narrowband/broadband average (□) of all survey data for each country with the number of measurement points for the country in brackets. For comparison, the global weighted average marked with dot--dashed line through (□) and the ICNIRP reference levels for the public at 900 and 1800 MHz are also plotted. (Rowley and Joyner, 2012)

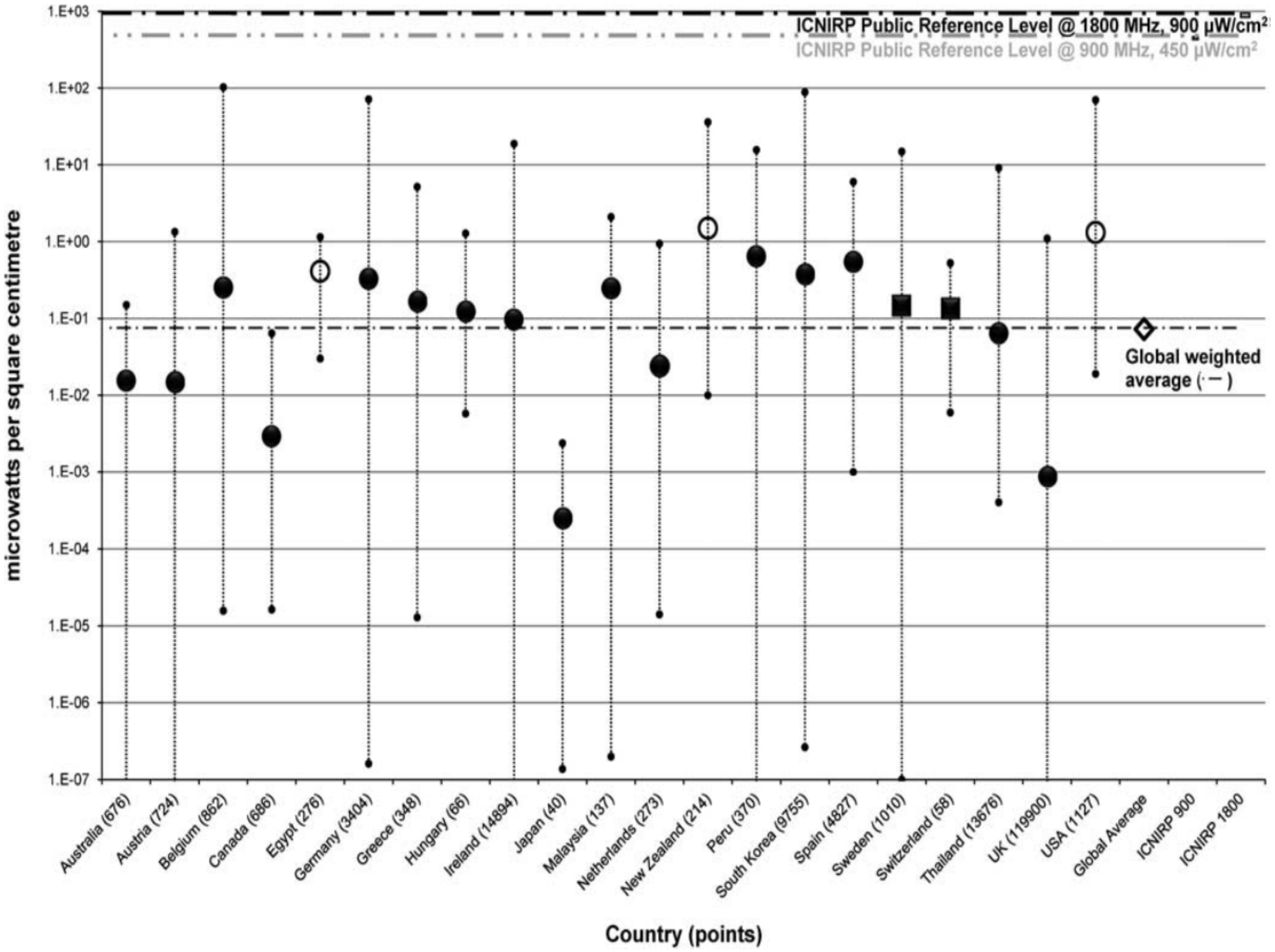

Figure 2 shows that despite the increasing number of base stations and the deployment of additional mobile technologies, the electromagnetic radiation levels have essentially remained the same in each country. Nevertheless, the results also show that the environmental level of radiation from mobile communication base stations is at least one order of magnitude higher than the median exposure level of 0.05 mW/m^2 reported more than 30 years ago by Tell and Mantiply (1980) for measurements of VHF and UHF broadcast services from 486 locations distributed throughout 15 large cities in the USA.

In a recent study by Tell and Kavet (2014) measurements were taken at a total of 94 sites across four cities in the USA. Although a direct comparison of the two studies is not possible due to design limitations, it is clear from the reported results that for the FM radio broadcast band there was a three-fold increase in the median value of the power density.

Figure 3. Minimum, maximum and average of the narrowband measurements for the UK, Spain, Greece and Ireland; and the broadband measurements for the US, with the year of measurement data on the horizontal axis. Note that not all years were available in all countries. For comparison, the ICNIRP reference level for the public at 900 and 1800 MHz are included. (Rowley and Joyner, 2012)

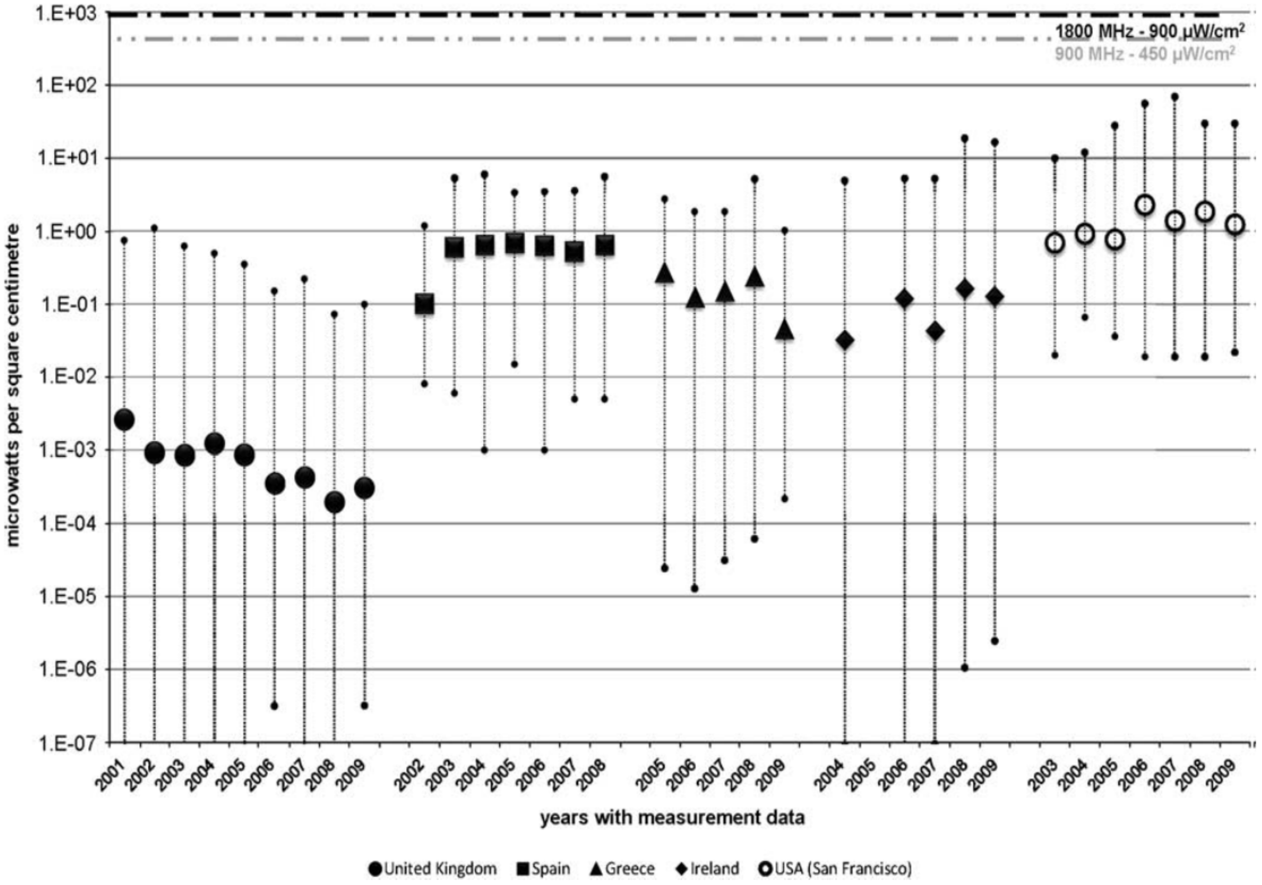

Figure 4. Minimum, maximum and average for each wireless technology. For comparison, ICNIRP reference levels for the public at 900 and 1800 MHz are also plotted. Mobile Other refers to mobile technologies either not identified in the source survey or not included (e.g., PDC) in one of the other mobile technologies categories. All Mobile is the result of averaging over all mobile technologies. Only narrowband measurements (from 16 countries) could be used. The weighted averages for all available measurement years for each country were then averaged over the number of countries with measurements for each mobile technology. The figure in brackets on the horizontal axis label is the number of countries for which measurements were available for each technology. (Rowley and Joyner, 2012).

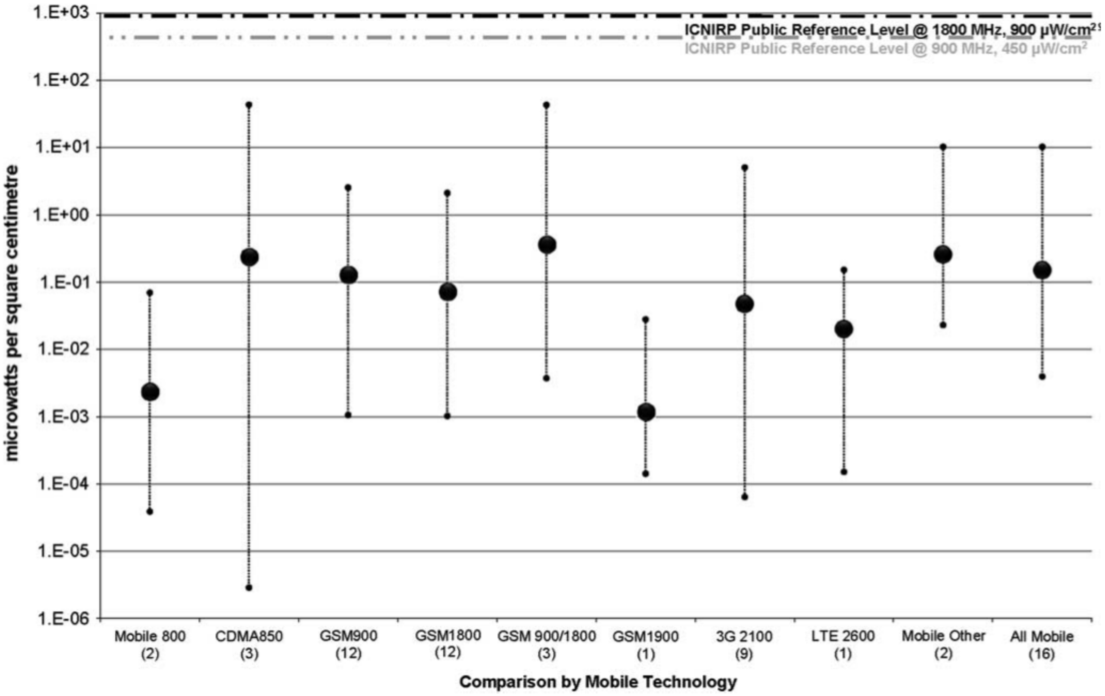

With respect to emerging mobile communication technologies, the measurement campaign in Stockholm, Sweden, has shown that the average contribution of LTE (Long Term Evolution) to the total radiofrequency exposure was less than 5% (Joseph et al., 2010b).

The results from the comparison of personal exposure data across five European countries (Joseph et al., 2010) have shown that exposure in all countries was of the same order of magnitude and that in the outdoor urban environment, mobile phone base stations and mobile phone handsets dominated the exposure. The exposure from the downlink frequency bands of mobile communication systems ranged in the outdoor urban environment of the five countries between 0.08 and 0.35 mW/m^2. These values are considerably lower than the value of 1 mW/m^2 derived from measurement campaigns around base stations (figures 2 and 4), but this difference can be explained by the way the measurement points were selected in the latter case, i.e., mainly in the vicinity of base stations and in some cases within their line of sight (LOS).

Microwave links

On the masts of mobile phone base stations very often drum-like antennas are mounted; usually more than one. These antennas serve to wirelessly link two points with a microwave communications link in the GHz frequency range and it is very unlikely that a member of the general public gets in the main lobe of the antennas, especially since they are mounted at a significant height. In their majority, these antennas are parabolic dish reflectors similar to the antennas used for receiving satellite broadcasting signals. However, the size of parabolic antennas and the emitted power of microwave links may differ according to the application. For satellite uplink broadcasting, several hundreds of W are used with dishes that can reach 5 meters in diameter. In this case the antenna is directed at a satellite avoiding all obstacles in-between, therefore exposure to the main lobe is unlikely to happen.

Apart from fixed installations VSAT (Very Small Aperture Terminal) transportable stations also exist. They use antennas less than 3 meters in diameter (most of them are about 0.75 m to 1.2 m tall) and a power of some Watt. The transmission rates of VSAT stations usually range from very low up to 4 Mbps. These VSAT usually access the satellites in the geosynchronous orbit and relay data (e.g., TV signal) from terminals on earth to other terminals and hubs.

Cordless phones

There are both analogue and digital cordless phones marketed, although the latter have dominated in recent years, due to their technological advantages and quality of communication. The average transmitted power of cordless phones is about 10 mW. Analogue cordless phones continuously emit during operation, whereas digital cordless phones can involve timesharing and pulse modulation. Therefore, the peak power of the latter can be higher than 10 mW. Digital Enhanced Cordless Telecommunication (DECT) phones, for example, have a peak power of 250 mW. However, they operate with 400 μs bursts every 10 ms, resulting in a 4% duty factor (the percentage of the time that they emit), which if multiplied with the burst peak power gives an average value of 10 mW. DECT phones operate at 1880 – 1900 MHz and offer voice communication. Although there is no adaptive power control for the cordless phones, it is clear from the above that their average power is smaller than that from mobile phones operating at their highest power level. As far as DECT base stations (the fixed part of the device) are concerned, it must be noted that when in standby mode they transmit an 80 μs burst every 10 ms, i.e. they have a duty factor of 0.8%, and, thus, an average power level of 2 mW. With the ECO DECT technology, transmission power is turned off when the handset is docked and charging and is adjusted according to the handset's distance to the base station.

Two studies (Kühn et al., 2007; Schmid et al., 2007) that measured DECT devices, reported that at a distance of 1 m the maximum power density from the base station was less than 40 mW/m^2, which is less than 1% of the ICNIRP reference levels (ICNIRP, 1998). The reported worst-case 10 g averaged spatial peak SAR was less than 0.06 W/kg (Kühn et al., 2007), a value which is also several times below the ICNIRP basic restriction for local exposure of 2 W/kg.

In a similar way to mobile phones, the operation of cordless phones with 10 ms frames leads to the presence of an ELF MF magnetic field of 100 Hz.

Terrestrial trunked radio

Terrestrial trunked radio (TETRA) is a digital technology mainly used for the mobile communications of emergency services. It uses the frequency range of 380 – 470 MHz. The system works in a time-division multiple-access way, similarly to GSM but only with four time-slots per frequency channel and 17 frames per second. In normal two-way voice communication only one of these four time-slots is used, resulting in a 25% duty factor (percentage of time when there is transmission) for the hand-portable equipment. Since the maximum power of portable devices is 1 and 3 W, the above duty factor leads to average powers of 0.25 and 0.75 W respectively. If the device is used for both voice and data transmission, i.e. more than one of the four available slots are occupied, the average power can increase accordingly. Commercially available TETRA handsets come with either helical or monopole antennas. Several numerical dosimetry studies (Dimbylow et al., 2003; Schmid et al., 2007; Wainwright, 2007) have investigated the operation of TETRA devices against the ICNIRP exposure guidelines (ICNIRP, 1998). They have shown that the 10 g averaged SAR values were always below the occupational basic restriction but could exceed the general public basic restriction by up to 50%, such as in the case of a 3 W device with a helical antenna (Dimbylow et al., 2003).

In a similar manner to the GSM system there is a location update signal sent from a TETRA mobile device to the base station. The rate of the location update can be set in a wide range and largely depends on the network operator. The maximum rate defined by the standard is every 10 seconds.

Bluetooth devices

Bluetooth devices operate at the license free ISM band of 2.45 GHz. They are used to connect devices within a short range wirelessly. They come at three different power classes of 1, 2.5 and 100 mW, with a range of about 1, 10 and 100 m. Hands-free kits that are connected to mobile phones operate usually at 1 mW (class 3) or 2.5 mW (class 2), such as in the case of car-kits. In a simulation of a realistic case with a class 2 device Martínez-Búrdalo et al. (2009) calculated 10 g averaged SAR values that were about 1/1000th of the ICNIRP basic restriction of 2 W/kg (ICNIRP, 1998), which is consistent with the measurements of Kühn et al. (2009) who reported peak spatial 10 g SAR values lower than the sensitivity of the measuring equipment (5 mW/kg). In an earlier study, Kühn et al. (2007) had measured the maximum 10 g averaged SAR of a class 1 (100 mW) Bluetooth device to be less than 0.5 W/kg and the electric field strength at 1 m distance at 1 V/m.

Baby monitors

Baby monitors are one- or two-way communication devices that that are used to transmit the sound or the picture of an infant, or to transmit the voice of an adult for calming an infant. Baby monitor operate at 40, 446, 864, 1900 and 2450 MHz and can have peak transmit powers up to 500 mW. Schmid et al. (2007) reported maximum electric field strength of 1.1 V/m at a distance of 1 m, whereas Kühn et al. (2007) reported a higher value at the same distance of 3.2 V/m. In the latter study the 10 g averaged SAR was measured to be lower than 0.1 W/kg, therefore several times below the 2 W/kg basic restriction of ICNIRP for the general population (ICNIRP, 1998).

Wireless local area networks

Wireless local area networks (WLAN) are formed by devices which connect directly with each other or via an entry point to a wired network, known as the access point (or "hot spot"). In order to establish the connection with these devices, which can be a laptop, a peripheral computer (e.g., printer, digital camera, and video projector), a game console and so on, an antenna and a transmitter have to be included. The most common WLANs operate at the license free frequency bands of 2.4 and 5 GHz. The technical standards for WLANs are produced by the Institute of Electrical and Electronic Engineers (IEEE) and have evolved to provide for data rates up to 72 Mbps in a single channel. In Europe, the European Telecommunications Standards Institute (ETSI) standard EN 300 328 limits the maximum power for any system operating in the 2.4 GHz band to 100 mW.

Several studies have assessed exposure to devices operating in a WLAN. In a dosimetric measurement of access points touching a flat phantom filled with tissue simulating liquid, Kühn et al. (2007) reported that the maximum 10 g averaged SAR was less than 1 W/kg. They also reported a maximum power density of approximately 3 mW/m^2 at a distance of 1 m and 40 mW/m^2 at a distance of 0.2 m from an access point. At the same distances Foster (2007) and Schmid et al. (2007) reported 1 mW/m^2 and approximately 180 mW/m^2 respectively. It should be stressed that all the values given above are far below the reference level of 10 W/m^2 specified in the ICNIRP guidelines (ICNIRP, 1998). The numerical dosimetric studies of Martínez-Búrdalo et al. (2009) and Findlay and Dimbylow (2010) have also confirmed that the maximum local SAR values are within the ICNIRP basic restrictions for the general public. At 2.4 GHz, using a power of 100 mW and a duty factor of one (100%), the highest local SAR value in the head was calculated as 5.7 mW/kg (Findlay and Dimbylow, 2010). However, in reality, the duty factor is much less. In fact, for 146 individual laptops and the access points from 7 networks investigated in UK schools, the maximum duty factors were 0.91% and 11.7% respectively (Khalid et al., 2011). Applying these duty factors to the numerical dosimetric results from the previous studies would result in a peak 10 g averaged SAR value of some μW/kg in the torso of a 10-year-old child.

Another WLAN technology known as Worldwide Interoperability for Microwave Access (WiMax) has emerged in recent years to provide connectivity at a larger range, similar to that of cellular networks (up to 50 km for fixed stations). Joseph et al. (2012) have

reported values up to 0.3 V/m (0.24 mW/m^2) for the electric field strength from WiMax applications in various indoor and outdoor environments.

Recently, the Wireless Gigabit Alliance (WiGig) was formed, which envisions seamless connectivity between digital devices at multi-gigabit-speed data rates that will drive industry convergence to a single radio using the license free 60 GHz band. The typical application for the new WLAN technology will be multimedia streaming for high definition video and audio, as well as latency free gaming.

Smart meters

Smart meters are devices that allow the remote monitoring of energy consumption (usually electricity and gas) by allowing data, such as location, consumption units and time of usage to be wirelessly transmitted to the utility company at regular intervals.

Recently, a report (EPRI, 2010) and several papers (Tell et al., 2012; Zhou and Schneider, 2012; Foster and Tell, 2013) have been published regarding the exposure associated with smart meter use. The devices investigated were both an end point meter, as well as cell relays. The former includes two transmitters, of which one connects the end meter to the local area network (LAN) at a license free or licensed frequency band, while the other operates at the 2.4 GHz ISM band to interact with other devices in the home constituting the home area network. The second type of smart meter includes a third type of transmitter operating usually at a cellular communications frequency (e.g., 900 or 1900 MHz) to form a wireless wide area network (WWAN), which collects the data from all the end meters and forwards them to the utility company (relay function). The percentage of time that a smart meter is active transmitting data (duty factor) depends on the technology used. In the paper by Tell et al. (2012) the maximum duty factor for end point smart meters was only 4.74% and for cell relays approximately 0.088% (due to the high data rate provided by the specific wireless technology used). Although the nominal maximum transmitted equivalent isotropy radiated power (EIRP) of the examined meters was 2.3W, the measured value for the same cell relay meter was a lot smaller (0.3 W). Given the above, Tell et al. (2012) concluded that under virtually any realistic condition of deployment with the meters operating as designed, the RF power densities of their emissions will remain, in most cases, two orders of magnitude or more below FCC's maximum permissible exposure (MPE) levels for the general public (6 W/m^2 at 900 MHz) both in front of and behind the meters.

Wireless smart meters are not the only type used in practice. Power line communications (PLC), which allows the transmission of broadband signals through power line cables, is also employed for the implementation of remotely reading the utility meters. Zhou and Schneider (2012) conducted a dosimetric study of a smart meter with the help of the Virtual Family computational phantoms models (Christ et al., 2010). They concluded that if the estimated SAR values were reduced to take into account the 0.088% duty factor the exposure guidelines would be met, even when the human placed his or her head against the meter. They also concluded that vertical displacements of the meter relative to the body never produced greater peak spatial SAR values than when the smart meter antenna was aligned with the nose.

In their effort to assess exposure form a specific type of a smart meter, Foster and Tell (2013) reported that it was not always possible to clearly distinguish emissions from the meters, since smart meters operate at similar power levels and in similar frequency ranges as many other digital communications devices in common use. They reported a duty factor of 0.05-0.1% over the course of a day and concluded that smart meters "would make only minor contributions to the total background RF radiation level inside a home, which is in any event tiny in comparison to accepted safety limits".

3.3.2. Industrial applications

Occupational exposure has been discussed in several publications and perhaps the most comprehensive text can be found in the fact sheets produced in the EU project EMF-NET:

Effects of the Exposure to Electromagnetic Fields: From Science to Public Health and Safer Workplace (see also Table 2).

These fact sheets are available at:
http://www.ciop.pl/CIOPPortalWAR/appmanager/ciop/pl?_nfpb=true&_pageLabel=P620059861340178661073&html_tresc_root_id=32277&html_tresc_id=300003145&html_klucz=32274&html_klucz_spis

accessed January 30, 2015.

In this chapter we therefore only briefly discuss the various sources and the exposure that can occur in industrial application.

Table 2. Sources and types of occupational exposure to EMF. From EMF-NET Main Task MT2 WORKEN - Deliverable D49[11] (Hansson Mild et al. 2009)

TABLE 2. EMF Exposure at the Workplace—Common Applications Resulting in EMF Emission

	EMF Frequency Related To Application				Workers' EMF Exposure		
EMF Source	Static	ELF	IF	RF/ MW	Probably Low-Level*	Possibly High-Level**	Probably High-Level***
Induction heating		oo	o			xx	x
Surgical and physiotherapeutic use of diathermy			oo	oo		xx	x
Dielectric heating (RF: glue drying and plastic welding & MW: heating and vulcanization applications)				oo		xx	x
Arc-welding (MIG, MAG, TIG, etc.)	oo	oo	o			xx	xx
Spot welding	o	oo	o			xx	x
Electrochemical installations or other ones using microwaves (e.g., chemical activation of processes)				oo	NAD		
Electrolytic installations	o	oo			xx	x	
Industrial microwave ovens				oo	xx	x	
MRI medical diagnostic equipment	oo	oo		oo		xx	o
NMR spectrometers	oo			oo	x	x	x
Electric vehicles (trains, trams, metro)	o	o			xx	x	
Plasma discharge equipment				o	NAD		
Plasma polymerization at RF				o	NAD		
Radar and other systems				oo			xx
Broadcasting systems and devices (radio and TV: AM, VHF, UHF)		o	o	o	xx	x	x
Mobile telephony base stations				oo	xx	x	x
Military and research RF systems			o	oo	x	xx	x
RFID, EAS and other security equipment	o	o	o	o	xx	x	x
WLANs				oo	xx		
Cordless phones				o	xx		x
Bluetooth devices and hand-free kits				oo	xx		x
Electricity supplying networks and electricity distribution and transmission equipment	o	oo			xx	x	
Electric handheld tools		o			xx	x	x
Industrial magnetizers demagnetizers	o	oo				x	

Notes. EMF—electromagnetic fields, ELF—extremely low frequency, IF—intermediate frequency, RF—radiofrequency, MW—microwave, MIG—metal inert gas, MAG—metal active gas, TIG—tungsten inert gas, NMR—nuclear magnetic resonance, MRI—magnetic resonance imaging, AM—amplitude modulation, VHF—very high frequency, UHF—ultra high frequency, RFID—radio-frequency identification, EAS—electronic article surveillance, WLAN—wireless local area network; NAD—no available data; oo—basic frequency range, which is in the most common use for specific applications; o—other frequencies, which can be used for specific applications; xx—the most common situation in the work environment; x—a possible situation in the work environment; *—detailed exposure assessment not necessary; **— assessment with external measures, using environmental measurements; ***— assessment with internal measures, computational assessment may be needed.

Static and ELF fields

Strong static magnetic fields are uncommon in industrial applications, with some exceptions. In aluminium production the current used can reach hundreds of kA with static fields of the order of some mT close to the conductors, and the general level in the factory is up to 1 mT. The current is rather smooth and the ELF component from the ripple is of the order of some μT only.

In electrolytic processes, the static magnetic field levels at the operator's locations can be approximately 8-15 mT, but here the ELF component from the ripple from the AC

11 http://www.portaleagentifisici.it/DOCUMENTI/NIR_DOCUMENTAZIONE/Final_technical_report_D49_FactSheet.pdf?lg=IT

rectification is perhaps the interesting part. The ELF MF can reach some hundreds of µT at basic frequencies of 50-300 Hz.

Magnetic resonance imaging systems use magnets typically from 0.05 T to about 3 T. Also static magnetic fields, RF fields (10-100 MHz) and rapidly changing gradient magnetic fields occur in pulse sequences within MRI equipment. The maximum level is about 1 T in front of the magnet, and nurses/technicians staying with patients can be exposed to up to 0.2 T, approaching the protection guideline. However, it must be borne in mind that ICNIRP employs reduction factors from effect thresholds, when setting exposure limits, so exposure to well-characterised EMF some way above the exposure limits is possible before effects take place.

Strong static magnetic fields are used in MRI and NMR application and this is dealt with in section 3.8.

RF

The use of RF fields in our workplaces has increased rapidly during the last decade, mainly due to the increased use of wireless communication techniques, security devices and in medical applications. However, although workers' exposure in these cases is in general low and not in conflict with the EU directive, there are exceptions.

In the office as well as in the industry and transportation environment, wireless communications are frequently used. The indoor base stations as well as different blue tooth equipment and WLAN used for man to machine or machine to machine communication have a low output power and therefore the possible exposure of workers is not in conflict with the regulations.

Low exposure can also be expected when the sources are enclosed. Examples in the industry are plasma metallization and polymerization, plasma deposition and etching and microwave heating, for instance vulcanization of rubber. These processes are normally performed in closed chambers, but there might be leakages especially after reconstructions or changes in process and therefore a simple recurrent check might be a part of the assessment.

The number of devices used for security purposes, as anti-theft and personal access control have increased rapidly in shops, libraries, airports and restricted areas. These devices operate at different frequencies depending on which technique is used. Several work below 100 kHz, but the RFID equipment (Radiofrequency Identification Device) works at 120-154 kHz and there are also devices working up to 4.9 GHz. The first calculations with a numerical model of a pregnant woman exposed to an RFID reader operating in the 900 MHz band have shown that the peak spatial SAR remains below the basic restrictions (ICNIRP, 1998) for 1 W radiated power and 100% duty cycle (Fiocchi et al., 2013).

Electronic Article Surveillance (EAS) systems works usually in the MHz range both in continuous swept frequency and at fixed pulsed frequency at the detector. Normally, the personnel only pass through these areas and are therefore only exposed during a short period and not in conflict with the regulations. However, there might be devices situated near a permanent working place, for instance a cashier. In such cases actions must be taken to insure that the regulations are fulfilled. In some workplaces it will be necessary to take measurement for showing compliance with the EU directive. Examples of such workplaces are given below.

Dielectric heaters

RF sealers and glue dryers are two examples of dielectric heaters frequently used in the industry to seal plastic objects and to glue wood details. The output powers range from 1 to 200 kW. Most sealers are operated manually and require the presence of the operator close to the RF electrodes. In some applications, pieces of plastic materials to be heated must be held by hand, and the operator's hands will be highly exposed to RF fields.

Electric field strengths range in areas of operators typically from 1 to 300 Vm^{-1}, and magnetic fields range from 0.1 to 20 Am^{-1} respectively.

In workplaces where these devices are used it is necessary to perform detailed measurements of both the electric and the magnetic fields as well as contact and induced currents. These measurements often need to be done on a regular basis, perhaps yearly, since the radiation pattern from the machinery changes with use.

Induction heating

Operators of induction furnaces and heaters are highly exposed; at 1 meter from a 1-10 kHz heating equipment, flux densities typically range from 0.03 to 0.5 mT, and may reach 5 mT at 10 cm. Similarly, devices working at a frequency of 50 Hz, may produce 5 mT fields at 20 cm, and over 0.1 mT at a distance of several meters, and the guidelines (27 µT for 3 kHz -to 10 MHz and 1 mT for 50 Hz;, ICNIRP, 2010))) are exceeded manifold during work procedures close to furnaces.

Industrial microwave ovens and microwave drying

These ovens are often closed and no access is given to areas where high intensity microwave ovens can be encountered. However, there may be leakage in some cabinets and connections, and a regular maintenance program is recommended.

Microwaves are also used for drying of water damage in buildings. These applications are usually high powered devices with an applicator that has some potential leakage. Due to the high intensity microwave energy used it is also possible to get exposure on the other side of the wall or floor where the applicator is located. Great care when using these devices is needed, and in some countries there is a demand for licensing for the use of these machines.

Radar

In general it would be exceptionally to find cases of staff being exposed to direct emissions of radar signals from the antennas. Often measurement is not needed and the exposure assessment can be done by numerical calculations. However, during manufacturing, service and repair it may happen that staff accidentally can be exposed.

Some of the radars used by the military can have a very high output power and therefore are restricted in use at close range. As an example we can mention a destroyer that was equipped with so called SPY radar. This is mounted on four places around the ship and consists of phase controlled small antennas. The radar beam can be formed into a so called pencil beam and it is randomly searching the area. The power is of the order of 6 MW and with an antenna gain of 10,000 the power density at 100 m distance can reach several hundreds of kW/m^2 with a peak electric field exceeding 10 kV/m. This can cause permanent damage to electronics.

It has been confirmed that in a quiet environment humans can hear radar pulses (Chou et al., 1982; Elder and Chou 2003). Experimental studies have shown that the hearing is caused by thermoelastic pressure waves generated in the head due to the inhomogeneous absorption of the radar pulses. Other effects on man from a short term exposure, besides feeling heat, are not known.

Broadcasting and other communications

Radio and TV broadcasting installations are usually safe workplaces. However, there is a potential for involuntary, accidental intense exposure of staff. In most of the cases, technical staff working at radio/TV broadcasting equipment, are technically well informed and trained. However, when working near antennas with repair or adjustment during broadcasting, occupational exposure is likely to be in conflict with the EU directive. These situations should be avoided. Rooftop workers near base stations antennas might be exposed to RF fields about 900– 2000 MHz. Examples of such workers are sheet metal workers, chimney-sweeper and painter. In these cases the emission properties are well defined and simple instructions are more relevant than measurements.

ELF

In arc welding, electric currents up to 1 kA can be used. The cable carrying the welding current can touch the welder or even be wrapped around a shoulder of the welder. Magnetic flux densities are approximately 1-2 mT at the surface of the welding cable and power supply, exposing the welders to strong ELF fields.

Handheld electric tools
We are not aware of any new publications dealing with the exposure from handheld tools, but there is a need to clarify these questions with a more systematic measurement of different tools.

It is not straight forward to measure EMF from handheld tools. It is clear that they are surrounded by a magnetic field when used; the machines can use up to a kilowatt of power which leads to currents in the wiring of the order of a few amperes. B fields in the range of a few hundreds of μT are not uncommon measured at close distance, and as such they do not exceed international guidelines. The problem in exposure assessment arises when we start looking at the average time of the exposure. Hansson Mild et al. (2009) brought up the example of a handheld electric drill. The machine usually draws 10 times more current during the first few periods and the corresponding magnetic field is also strongest then. Standard No. EN 62233:2008 states that the measurement should be taken at a certain distance from the machine, and for the first 200 ms from the start-up the machine should be neglected. But since the limits for exposure to ELF fields are set to protect against nerve excitation, which can happen even within a half-period of the power frequency alternating current (AC), i.e. during exposure of <10 ms (Reilly, 1998), this then becomes very questionable.

The question of average time needs also to be discussed in connection with exposure assessment of for instance a spot welding machine. Usually the limits are set in root-mean-square (rms) values for field strengths, but should averaging be over one second or a shorter time period? Various standards give different answers, but since most commercially available instruments use one second as averaging time, this is the most commonly used period. In contrast, Directive 2013/35/EU 2013/35/EU does not specify any averaging time for frequencies <100 kHz. IEEE Standard No. C.95.6:2002 gives the rms averaging time as the longer of 0.2 s or 5 cycles (up to 10 s) ((IEEE, 2002).2010). However, even the use of this standard might be problematic. An assessment of exposure produced by a spot welding machine is an example. The total welding time, i.e. the time when the current is on, is typically shorter than one second, even only a few periods of 50 Hz (i.e. the order of tens or hundreds of 1 ms) (see further Hansson Mild et al., 2009). The whole weld is over before the averaging time is up.

3.3.3. Medical applications

Diathermy

Diathermy is a technique used in physiotherapy for the treatment of acute or chronic orthopaedic and inflammatory conditions. Its therapeutic effect derives from the heat produced in the tissues, due to the absorption of electromagnetic energy at high frequencies, and from the influence of transmembrane ionic activity at low frequencies (Maccà et al., 2008). Short-wave diathermy devices operate at 13.56 or 27.12 MHz in a continuous or pulsed mode. Microwave diathermy is applied mainly at 2.45 GHz, although there are devices working at 434 MHz, as well. The studies for the evaluation of exposure due to diathermy have mainly focused on the occupational exposure of physiotherapists.

A measurement campaign in 20 physiotherapy departments across the UK operating 36 diathermy units has shown that at distances of 0.15 - 0.2 m the electric field strength for continuous wave operation was generally over 500 V/m and sometimes as high as 5000 V/m for capacitive equipment; the magnetic field strength at the same distances was 0.5 - 2.0 A/m (Martin et al., 1990), leading the authors to propose that the operator should keep a distance of at least 1 m from the unit, cables and electrodes when talking to a

patient during continuous wave treatments. However, in a more recent survey of 10 short-wave diathermy units operating at 27.12 MHZ, it was found that stray fields fell below the reference levels for occupational exposure given in the ICNIRP guidelines (ICNIRP, 1998) at 2 m for continuous wave capacitive and at 1 m for inductive equipment; another 0.5 was required before the fields fell below guidelines for other personnel (Shields et al., 2004). For microwave diathermy, measurements of approximately 11 devices have shown that if operators stand at 1 m away from the 2.45 GHz and 434 MHz applicators and not in the vicinity of large metallic objects, which could reflect radiation, they should not be exposed to fields above the reference levels for occupational exposure (Maccà et al., 2008).

A numerical study has shown that overexposure of tissues, such as the eye lenses, central nervous system and the gonads, can occur in a patient receiving short-wave diathermy at 27.12 MHz, if certain output power levels are exceeded for specific applicators, during the treatment of the head, the shoulder or the hip (Leitgeb et al., 2010).

Electrosurgery

Radiofrequency energy is used in several surgical procedures. In most cases the setup used entails a small active electrode as the applicator of high current density and a flat electrode (known also as the 'ground' or 'dispersive' electrode) from which the current returns to the generator (monopolar configuration). The active electrode acts as a cutting or coagulation instrument by applying sinusoidal or pulsed waveforms in the current in the frequency range of 0.3-5 MHz. Currently, a widely used minimally invasive electrosurgical procedure is radiofrequency ablation, which is routinely applied in oncology, cardiology and otorhinolaryngology.

In one study 6 electrosurgical devices were measured (De Marco and Maggi, 2006). It was found that near the equipment the measured fields were rather high, but at a distance of 0.5 m from the device the electric field strength fell to 32 - 57 V/m and the magnetic field strength to 0.2 - 0.8 A/m. According to the authors, in the worst case (maximum reading obtained) a surgeon's hands are exposed to an RF wave with magnetic field strength of 0.75 A/m and electric field strength of 400 V/m. However, it should be noted that stray radiation is produced not only by the electrosurgical unit but also by the cables (Liljestrand et al., 2003).

Active medical devices in and on the human body

Active medical devices operating inside or on the human body can be classified into two categories, namely diagnostic and therapeutic.

The first category includes the devices used for physiological monitoring, which find the most applications in medicine. Such devices are inserted into the patient's body for the *in vivo* monitoring of critical physiological information, such as heart function (electrocardiograph ECG), hemodynamics (venous oxygen saturation SvO2, blood pressure), body thermoregulation (temperature), and metabolic dysfunction (blood glucose level) (Kjellström et al., 2004; Paradiso et al., 2008; Klueha et al., 2005). This category also includes the miniaturized medical image capturing devices, such as the capsule endoscope, which are transiently inserted into the body (Liao et al., 2010; Cohen and Klevens, 2011). The second category of devices includes those which are used for the treatment of a disease, a dysfunction or an impairment, such as various neuromuscular microstimulators (Ghovanloo and Najafi, 2007; Kane et al., 2011), drug infusion pumps (Meng and Hoang, 2012) and other microelectromechanical systems (MEMS) based devices, as well as cochlear implants (Eshraghi et al., 2012) and visual prostheses (Ong and Cruz, 2011).

Many active medical devices inside or on the human body (such as cardiac pacemakers or breathing stimulators) communicate with other implants or external control units), in order to exchange commands, transfer data or, even, receive power. This process is called telemetry. So far, a wide range of radio frequency bands have been used by

medical device manufacturers for this purpose. However, the two frequency bands, which are most often used for medical systems are the Medical Implant Communication Service (MICS) bands (401-406 MHz) and the Industrial, Scientific, Medical (ISM) bands (e.g., devices with the protocols of Bluetooth in the 2.4 GHz ISM band and ZigBee in the 868 MHz and 2.4 GHz ISM bands for Europe). In telemetry, both inductive coupling and radiofrequency radiation are employed for implementing telemetry.

Unfortunately, despite the increased use of active medical devices inside or on the body, the Specific energy Absorption Rate (SAR), the current density, or the fields inside the tissues are not always reported, although they should form a design consideration (Q Fang, 2010). However, there are also reports of implanted devices either for biotelemetry (Scanlon et al., 1999; Shiba et al., 2008; Singh et al., 2009; Xu et al., 2009) or for wireless power transmission (O'Handley et al., 2008; Shiba et al., 2002; Zan et al., 2010), which mention the SAR and current induced in the patient tissues. They also give an indication of the maximum power or duty factor values that need to be obeyed to comply with ICNIRP guidelines (ICNIRP, 1998).

Cosmetic medicine

Radiofrequency energy is used in several applications of cosmetic (aesthetic) medicine, which include skin tightening and rejuvenation, cellulite reduction, acne scars treatment and hair removal (Sadick et al., 2004; Belenky et al., 2012; Lolis and Goldberg, 2012). The frequency of operation of the various devices used in this area is up to 10 MHz (Belenky et al., 2012). When RF energy is used alone (not in conjunction with light), the main mechanism of action is the heating of dermis. Partial collagen denaturation is caused because of the heat, which results in collagen contraction and thickening. The natural inflammatory wound healing response triggers neocollagenesis and further skin contraction (Lolis and Goldberg, 2012).

Unfortunately, there is not much information about the exposure of the operator of devices used in clinical dermatology. As far as the patients are concerned, the energy fluences can reach up to 144 J/cm^2 over 1 cm^2 of area (Lolis and Goldberg, 2012).

Transcranial magnetic stimulation

Transcranial magnetic stimulation (TMS) is a technique, based on the induction of an electric field inside the brain by the application of an external magnetic field. This field can depolarize neurons or modulate cortical excitability, by choosing the appropriate parameters of stimulation, even beyond the duration of the treatment session. This has behavioural consequences and therapeutic potential (Rossi et al., 2009).

One experimental study has assessed the exposure of the operator during a TMS treatment session: With a figure-8 coil, a pulse repetition frequency of 5 pulses/s and stimulus intensity of 60–80% of the stimulator's maximum output, the worker's reference levels for the magnetic field were transgressed at a distance of about 0.7 m from the surface of the coil (Karlström et al., 2006).

In a second numerical study, it was confirmed that the staff working with TMS treatments can become exposed to magnetic field levels exceeding the ICNIRP restrictions (ICNIRP, 1998). It was concluded that the figure-8 coil results in a smaller stray magnetic field and lower induced current density in the TMS operator compared with the round coil. The authors suggest that the operating staff should stand at least 1.1 m away from TMS coil and propose the use of robot controlled TMS systems instead of handheld devices (Lu and Ueno, 2010).

Electromagnetic Fields used in MRI

The electromagnetic fields used in MRI scanners have been thoroughly investigated by for instance Capstick et al. (2008), and have been discussed in length in a review by McRobbie (2012); therefore only a brief summary is given here.

Static field

MRI scanners in clinical use have superconducting magnets generally with cylindrical bores and provide static fields with magnetic flux density of 1.5 -3 T. A smaller number of ultra-high field MR systems are in use in research institutions worldwide and these use static fields up to 9.4 T. Due to the active shielding of the static field, especially for scanners with higher field strengths, the field drops quickly with a distance from the scanner, producing a large gradient of the static field so that the field may only become significant within 0.5 m from the bore opening. There is a requirement that the 0.5 mT contour around the magnet is marked, or access to it restricted, to prevent interference with implanted cardiac pacemakers and cardioverter defibrillators and to avoid accidental release of iron containing objects into the magnetic field. This contour is usually contained within the MRI scanner room. Static fields may interact directly with tissues via magnetic susceptibility causing differential forces on tissues, or by interaction with nuclear spins. Furthermore, motion of tissue (a conducting medium) in a gradient of the static field or rotation in a field will induce electric currents in the body. So-called open systems provide much greater access to the patient, facilitating, for example, interventional procedures. Such systems use static fields typically around 0.2 – 1 T.

The static magnetic field is always on, independent of whether an MRI procedure is being performed or not. That means that everyone moving around the scanner will effectively be exposed to a space- varying magnetic field (cause by motion in the static field and its gradient).

Switched gradient field

The switched gradient fields used for image encoding come from three different coils used to create linear gradients of the magnetic field in three directions within the scanner. Switched gradient fields (time varying magnetic fields), are deliberately created which must be distinguished from the inevitable time-independent gradients of the static field that exist where the magnetic field falls away around the scanner. These switched gradient fields are switched on and off to select the region of diagnostic interest and to spatially encode the MR signals. The faster the imaging sequence, the greater the rate of change of the gradient fields required. The amplitude of this is of the order of mT with fast rise and fall times of tens to hundreds of µs. Typically, the gradient field strengths in the region can be 25-50 mT/m and maximum slew rates (the peak amplitude divided by the rise time) can be 100 - 200 T/m/s within the imaging field of view. Gradient fields in modern systems can be as high as 100 mT/m with slew rates of 800 T/m/s. The gradient waveform is complex and not periodic but can be characterized by primary frequencies in the kHz range. The limiting factor for the patient's exposure is peripheral nerve stimulation (PNS) due to electric potentials induced across the nerve fibres. A limit has been set at about 50 T/s to avoid nerve excitation in the patient. The occupational exposure to the switched gradient field will be significant especially close to the bore. In Wilén et al. (2010) the rms value of the field was measured to be up to 0.1 mT at 0.3 m distance from the centre of the bore. From their data dB/dt values of 70 T/s could be calculated at the same position.

The magnitude of the magnetic field gradient and its time derivate depends on which pulse sequence is used.

Radiofrequency field

The RF field is usually created with a body coil integrated into the scanner that produces a circularly polarised B_1 field. For cylindrical bore systems at 1.5 or 3 T, this is usually a birdcage coil in order to provide a region around the iso-centre of the scanner where the B_1-field is spatially uniform. For open MR scanners with the static field vertical, the RF B_1 field is often produced by a pair of planar coils placed above and below the patient. Only the magnetic field component is required for the MRI. The E field is generally small except in the vicinity of the coil windings. The occupational exposure to the RF B_1 field will in general be low since the field falls off rapidly outside the transmit coil. An

exception will be staff carrying out interventional procedures, particularly in open scanners, where hands and arms, and possibly the head may be exposed to levels similar to those for the patients.

The RF field has a frequency of around 42 MHz/T, which means that for a 3 T scanner the frequency is around 126 MHz. There are limit values for SAR for patients (ref) and in normal operation mode the whole body SAR should be below 2 W/kg, whilst for the 1st controlled level the SAR should be below 4 W/kg. Different RF pulse sequences are used depending on what contrast is required. This leads to different SAR values for each pulse sequence, and typically during a clinical scan many different sequences are used to get the appropriate information. However, the intensity of each pulse can be substantial. Measurements show that the peak values for the RF B1 field can reach 10 A/m and more, and with a duty cycle on about 1%, the SAR values in the pulses are rather high. This is an area where very little information is available since almost all research on RF has been dealing with the average values and thermal effects.

The RF field and the switched gradient fields are only turned on during the MRI procedure. Only professionals that stay in the room during the procedure will be exposed to these fields.

The problem of conducting an exposure assessment for epidemiological studies has been discussed in a recent publication by a COST BM0704 group, see further Hansson Mild et al. (2012).

Exposure near MRI machines

Several new papers have been looking into the occupational exposure of persons working with MRI. De Vocht et al. (2009a) measured personal exposure to both static and time-varying magnetic fields, and they found that while the time-weighted exposure levels are below the ICNIRP guidelines, the peak exposure limits were exceeded during certain procedures.

Karpowicz et al. (2011) and Karpowicz and Gryz (2012) studied the exposure to static magnetic field (SMF) during operations of MRI scanners. Measurements near a 1.5 T MRI magnets showed that the SMF exposure from various scanners depends on both SMF of magnets and scanners design, as well as on work organization. A routine examination of one patient the radiographer was exposed to SMF exceeding 0.5 mT for approximately 1.5-7 min, and up to 1.3 min to SMF exceeding 70 mT. The mean values (B mean) of exposure to SMF are 5.6-85 mT, with a mean of 30 mT.

One of the main problems with the risk assessment of work near an MRI scanner is the induction of an electric field in the body when moving near the bore. Chiampi and Zilberti (2011) have addressed this problem and developed a computational procedure to evaluate the internal E field. For further details see Wang et al. (2012).

3.3.4. Security applications

Electronic article surveillance systems

Electronic article surveillance systems (EAS) are widely used in shops and libraries to prevent theft. However, reports on the magnetic fields around the EAS gates are very few in the literature. There are three components in an EAS, i.e. a detection unit (e.g., in the form of walk-through gates), a tag to be detected, and a tag deactivator. The main categories of EAS are also three, namely, electromagnetic systems (10 Hz - 20 kHz), acousto-magnetic systems (20 - 135 kHz), and radiofrequency systems (1 - 20 MHz) (Joseph et al., 2012).

Trulsson et al. (2007) measured the magnetic fields around 11 EAS in Swedish shops and found values of up to 536 A/m (673 μT) and 118 A/m (148 μT) next to an electromagnetic and an acousto-magnetic system, respectively. Both values were above the reference levels from the ICNIRP (1998) guidelines for the general public at the frequency of operation of the EAS. Joseph et al. (2012) measured the magnetic field at

several points near six EAS - two from each category - and also concluded that the maximum values were up to 13, 8 and 1.8 times higher than the reference values from the ICNIRP guidelines (1998; 2004) for the electromagnetic, acousto-magnetic and radiofrequency systems respectively. In particular, they measured rms (root-mean-square) values of up to 148 A/m (186 μT), 42.4 A/m (53.3 μT), and 0.14 A/m for the three EAS categories.

In a simulation study of Martínez-Búrdalo et al. (2010) it was shown that SAR and induced current density were kept below the basic restrictions (ICNRIP, 1998) even when the radiofrequency EAS operating at 10 MHz had a maximum magnetic field close to the maximum value measured by Trulsson et al. (2007), which exceeded the reference levels (ICNIRP, 1998).

Conducted electrical weapons

Conducted electrical weapons (CEW), also called neuromuscular incapacitation devices, ('tasers') use electrical currents to disrupt the voluntary control of muscles by stimulating involuntary muscle contractions. Such devices can be used in a pain compliance mode, whereby they are held against the target, so as to cause pain but not incapacitate it. The amplitude and time course of pulses delivered by the CEW may vary considerably; the net charge delivered may be in the order of some tens of μC (Reilly et al., 2009).

In a recent review of the literature on the acute pathophysiological influences of CEW, Kunz et al. (2012) concluded that the majority of current medical research could not find any acute clinical relevant effects during or after professional use of such devices on human subjects. However, they also note that in most of the current literature on CEW, tests were done on subjects with no significant medical history and the CEW devices were applied as indicated by the manufacturer. Furthermore, no testing has been performed on persons intoxicated by illegal substances. Therefore, possible CEW-related injuries or pathophysiological changes cannot be excluded in the field, where the targets often receive multiple shocks in extreme situations.

A numerical study of Leitgeb et al. (2010) has shown that the maximum cardiac rms current density amounted to 7.7 kA/m^2. This is higher than the values published so far and by far outweighs the reduced stimulatory efficiency of the short pulses compared to the sinusoidal fibrillation threshold. Therefore, the authors concluded that ventricular fibrillation risk from CEW cannot generally be excluded.

3.3.5. Power generation and transmission

Photovoltaic arrays

Public concerns about the potential health effects from magnetic fields emanating from installations of photovoltaic arrays for power generation had already appeared in the early 90's. The measurements performed in large scale installations of DC photovoltaic modules in the USA (Jennings et al., 1993) have resulted in magnetic field values of up to 18.3 μT at the closest distance to transformers and inverters for 60 Hz. This value became larger (27.4 μT) for a broader frequency range (40 - 800 Hz). The measurement of magnetic fields in the above frequency range at a distance of less than 2.5 cm from the inverter case of an AC photovoltaic module gave a value of over 0.2 mT (Jennings et al., 1997). Unfortunately, the literature on the magnetic fields from the components of roof-mounted photovoltaic modules is scarce and no conclusions can be drawn regarding their contribution to personal exposure to ELF MF.

Wind turbines

Limited research has been performed on the EMF generated by wind turbines, although their proliferation has been fast in the recent years. In a pilot study conducted in Bulgaria, Israel et al. (2011) measured both electric and magnetic fields in a wind energy farm comprising 55 wind turbines of the Vestas V90 3 MW type. Measurements were made on the border of the populated areas/settlements at 1.80 m above the ground and it was found that electromagnetic fields are not emitted during operation of wind turbines

or they are so small that they are insignificant compared to the values to be found in other measurements in residential areas and homes. This conclusion was confirmed by McCallum et al. (2014) who had performed measurements in a wind energy farm around 15 Vestas 1.8 MW wind turbines. The authors of the study mention that the magnetic field levels detected at the base of the turbines under both the 'high wind' (turbine generating power) and 'low wind' (turbine drawing power from the grid for maintenance) conditions were low (mean = 0.9 mG) and rapidly diminished with distance, becoming indistinguishable from background within 2 m of the base.

Transformers and power substations

Public concern on the exposure to ELF electric and magnetic fields (EMF) has mainly focused on power transmission lines. However, the exposure to EMF from transformers installed inside residential buildings has attracted the interest of many researchers in recent years. Keikko et al. (2006) investigated magnetic fields, especially the harmonic components, in electric distribution (20 to 0.4 kV) substations installed indoors. They extrapolated their measurements to calculate residential exposure immediately above the transformer room and reported a large contribution of the harmonics in the exposure.

In a survey of residential exposure at 50 Hz from 10 to 0.4 kV transformers in Hungary (Szabó et al., 2007) the mean magnetic field value in the apartments just above the transformer rooms was 0.66 μT, when spot measurements were taken in a grid of 0.5 m step. In a similar study in Finland (Ilonen et al., 2008) spot measurements were performed in 30 residential buildings with transformer stations installed in them. In the apartments exactly above the installation a mean value of 0.62 μT was measured, whereas the mean value was 0.21 μT in flats on the same floor but not exactly above the transformer. The measurements conducted in 41 apartments within 10 buildings in Israel (Hareuveny et al., 2011) resulted in an average magnetic field at the height of 0.5 m of 0.40 μT for the apartments above the transformer station and 0.06–0.12 μT in all other locations of apartments. In Switzerland (Röösli et al., 2011), the mean magnetic field in 8 apartments directly above or neighbouring wall-to-wall with the transformer station room was 0.59 μT. In another 10 apartments which touched the transformer room at a corner or edge the average magnetic field was 0.14 μT. In Bulgaria (Zaryabova et al., 2013), the average magnetic field measured at a height of 0.5 m, in 23 apartments that have rooms directly above and next to transformers ("exposed apartments") was 0.37 μT. In other apartments (in the same building) the average value was 0.24 μT on the same floor like the exposed apartments, and 0.03 μT on higher floors.

Joseph et al. (2009) performed magnetic and electric field measurements at positions accessible to the general public around two 150-36/11 kV substations. They measured momentary magnetic field values within the range of 0.05 to 13 μT and electric field values within the range 0.1 to 270 V/m.

3.3.6. Transportation

Exposure to electromagnetic field can be encountered when using different modes of transportation. Many studies have addressed the ELF magnetic field in trains. Nordenson et al. (2001) looked at railroad engine drivers and found that they are exposed to relatively high ELF magnetic fields (MF), ranging from a few to over a hundred μT instantaneous values, and with mean values over the working day from 2-15 μT depending on the type of engine. Röösli et al. (2007) found that for Swiss railway drivers mean exposure could be as high as 21 μT.

Much lower values were found in an Italian study by Contessa et al. (2010). The average exposure to ELF MF was in the order of 1-2 μT, with higher levels (few μT) only for one engine; occasionally in hot spots, close to wiring or specific equipment, the field values could reach several tens of μT.

Halgamuge et al. (2010) investigated the exposure values at the floor level and seat level in Australian trams and trains in urban and suburban areas, and in a hybrid car. The MF

strength was measured at different points inside and near the moving vehicles. The results are far lower than the ICNIRP recommended levels.

A large comprehensive summary report on low frequency EMFs encountered in different modes of transport has recently been presented by the Swedish Radiation Safety Agency, authored by Anger (2010). The agency has – as a part of the environmental monitoring - measured EMF in buses, cars, long-distance and commuter trains, trams, underground trains, marine vessels and aircrafts. The measurements were performed at randomly chosen places where passengers are present. All of the levels measured are well below the limits for general public exposure. The highest levels were measured in trains, where the mean MF strength ranged from 2 to 27 μT, depending on the type of train and coach. On single occasions, measurements in commuter trains showed a magnetic field strength of up to 80 μT.

Following the work by Vedholm and Hamnerius (1997) who showed for the first time that steel-belted tires in cars could produce an ELF MF inside the car, Milham et al. (1999) looked into this in more detail. They found that the magnetic fields emanate from radial tires due to the presence of reinforcing belts which are made of magnetized steel wire. When the tires spin, they generate ELF MF, usually below 20 Hz. The fundamental frequency of these fields is determined by the tire rotation rate and has a sinusoidal waveform with a high harmonic content. The field strength can exceed 2.0 μT at seat level in the passenger compartment of vehicles.

Tell et al. (2012) measured the magnetic field in electric and gasoline-powered cars. For seven electric cars, the geometric mean (GM) of all measurements was 0.095 μT with a geometric standard deviation (GSD) of 2.66, compared to 0.051 μT for four gasoline-powered cars (GSD=2.11).

3.3.7. Household appliances

Microwave ovens

Microwave ovens are among the most widespread devices at home. They work with ultra-high frequency (UHF) radiation in the frequency range of microwaves (0.3 – 300 GHz), hence the device name. Almost all of microwave ovens work at 2.45 GHz. The radiation is absorbed by food and heats it. However, the food itself does not radiate when it is inside the oven or after it is removed from it. By design and construction, although the radiation is confined inside the metal casing of the microwave oven with the help of a metal-wired glass door, there is still some radiation leakage from it. This is higher close to the casing, but falls off rapidly with distance, except in the case when the door seals are defective or dirty. In an early systematic study (Matthes, 1992) 130 microwave ovens from 20 different manufacturers were measured to determine their leakage radiation at 5 cm distance. Depending on their maximum operating power (300-1200 W) the measured values ranged between 0.2 and 1 W/m^2. In a more recent study (Alhekail, 2001), which included 106 devices, both in households and restaurants, the device power reached up to 4.4 kW. However, it was not the powerful devices that gave the highest leakage radiation of 60 W/m^2, but a 10-year-old device. In general it was found that older devices leaked more radiation. Nevertheless, the median value for leakage radiation was only 1.6 W/m^2 and, in agreement with theory, the power intensity of the radiation fell in both studies fast with distance following the inverse square law. An interesting aspect of microwave ovens is that, apart from the microwave radiation they work with, they are a source of static (a permanent magnet is used to power the magnetron) and low frequency magnetic fields. The latter were measured at several distances from 34 microwave ovens and amounted to some tens of microtesla (27±17 μT) at 5 cm, but dropped to some microtesla (1.7±0.6 μT) at 50 cm (Preece et al., 1997).

Induction hobs

Another household appliance used for preparing food is the induction cooker, known also as an induction hob. Induction cookers have been used by professionals in restaurants and other industrial environments for a long time due to their advantages, which include

shorter cooking times, energy saving and lower risk of burns and fire. Their environmentally friendly profile has increased their popularity as domestic appliances. They operate with magnetic fields between 20 kHz and 100 kHz, mainly in the intermediate frequency (IF) range which induce currents in special cooking vessels for heating them and the contained food. If the cooking zone is not completely covered by the cooking vessel, the possibility of stray magnetic field reaching the position of a person standing near the appliance exists. Moreover, if the vessel is touched by a person during the cooking process, a small current (leakage current) may flow through the body of that person. In some cases output is regulated by on-off modulation at a typical frequency of 0.5 Hz (one pulse every two seconds).

The technical standard for induction cookers (EN 62233) by IEC (2005) specifies that the reference value of 6.25 μT recommended by ICNIRP (1998) should be met at a distance of 30 cm from the cooking field when one cooking zone is operated with a cooking vessel large enough and centred on the cooking zone. However, it is not always possible to keep this distance from the appliance, particularly when pregnant women, children and people of small stature are standing next to the cooker. Therefore, measurements have also been performed at closer distances and have shown (Christ et al., 2012) that directly in front of the device cabinet the magnetic field can even exceed the occupational limit of 30.7 μT at the frequency of 20 kHz. Induction hobs are at the top of the list in generated magnetic fields, despite the fact that the highest magnetic fields are usually emitted by motor-driven appliances, tools and kitchenware (Leitgeb et al., 2008).

Electric heating systems

Electric floor heating systems comprise an arrangement of heating cables or films incorporated in the thickness of the floor below a covering. Heat is produced by the flow of electric current through the incorporated heating elements. This current may generate low-frequency magnetic fields around the heating elements, the field strength varying according to the type of heating cable used. State-of-the-art electric floor heating systems produce only negligible magnetic fields. These systems employ two-core heating cables in which the magnetic fields of the adjacent supply and return conductors cancel each other out. On the contrary, single-core heating cables carry a single heating conductor and the supply and return conductors in this type of system may lie far apart. As the magnetic fields of the two conductors cannot fully offset each other, a residual magnetic field persists.

Storage heating systems use the thermal mass of the floor to store heat energy. The heating cables are laid in the bottom section of an approximately 10 cm thick cement layer. The thermal store is normally heated up during the night using off-peak electricity. The stored energy is then passively released to the space as radiant heat during the daytime. Low-frequency magnetic fields occur during the heat-up phase, i.e. normally during the night.

Direct systems, which employ a thin screed as a short-term thermal store, respond more immediately to temperature fluctuations. Energy is passively released as radiant heat with only a short time lag, the short-term thermal store being replenished throughout the daytime as required. Low-frequency magnetic fields occur during the heat-up cycles, i.e. in most cases throughout the day.

Electric floor heating systems are the reason for higher magnetic field values at the level of the floor in Swedish residences, according to a recent study (Hamnerius et al., 2011).

Mobile electrical radiators start operating when their temperature falls below the pre-set temperature of a thermostat by storing heat in the water or oil they contain. During their operation a low frequency (50/60 Hz) magnetic field is produced in their immediate vicinity with a value of less than 1 μT. The magnetic field rapidly falls with distance from the appliance.

Toys and playthings

Radio-controlled toys include cars, boats, planes, helicopters and scale railway locomotives. Radio-controlled devices often have a transmitter that is the controller and have control sticks, triggers, switches and dials at the user's fingertips. The receiver is mounted in the toy itself and receives and processes the signal from the transmitter, translating it into signals that are sent to the servos. High-end toys use pulse-code modulation (PCM) to provide a computerised digital bit-stream signal to the receiving device, instead of analogue-type pulse modulation. There is a large range of operating frequencies and output powers for the radio-controlled toys available in the market. In terms of exposure assessment, each device needs to be considered on the basis of its own output power and frequency of operation.

Certain playthings, such as plasma balls, emit the highest electric fields found in our living environment in the intermediate frequency (IF) range. These products, are devices that use high voltage to create ionized light discharges. Measurements have shown (Alanko et al., 2011) that the recommended reference levels for the general public are exceeded at distances <1.2 m, and that the contact currents in the hand may be two times higher than recommended by the general public guidelines.

3.3.8. THz technologies

In the literature, there are various definitions of the THz frequency range, depending on the application under consideration. For telecommunication engineers this frequency range spans from 0.3 to 3 THz (1 THz = 10^{12} Hz) and is also known as the Tremendously High Frequency (THF) range (Tanenbaum 2002); frequencies above this range are considered in the optical radiation spectrum. In the field of biomedical engineering the THz frequency range may include up to 30 THz (Shumyatsky and Alfano, 2011). For the purposes of this Opinion, we shall define the THz radiation as covering 0.3 to 20 THz, i.e. having a wavelength between 1 mm and 15 µm, spanning the spectral interval between the millimetre wave and the infrared regions.

From a spectroscopic point of view, biologically important collective modes of protein, RNA and DNA vibrate at THz frequencies, whereas polar liquids like water strongly absorb THz frequencies due to rotation and collective modes. These features make THz imaging very attractive for medical applications. As a matter of fact, many organic substances have characteristic absorption spectra in the THz frequency range, while the high water absorption coefficient, although limiting penetration depth in biological tissues, allows for extreme contrast between less or high hydrated tissues to be employed for medical imaging.

Another valuable property of such fields is their ability to pass through a wide range of materials, like plastics and cardboard, making it possible to inspect packaged goods and opening the way to non-destructive and non-invasive inspection of packages like mail envelopes and laggings for manufacturing, quality control, and process monitoring (Jansen et al., 2010).

Radiation at this frequency range has been a subject of study for astrophysicists for many years, because approximately one-half of the total luminosity and 98% of the photons emitted since the Big Bang fall into the submillimeter and far-infrared (Mueller, 2003). It has also been used by scientists in the laser fusion community for the diagnostics of plasmas. However, for many years, THz sources were not generally available, and this gap has only recently begun to be filled by a variety of high quality sources and detectors of THz field. This has provided great advances in research and continues to enable further applications to be investigated. The power of THz sources ranges from a few nW to a few W (Shumyatsky and Alfano, 2011).

The opportunities for THz science in chemistry and biology are wide ranging from label-free sensors to cell signalling, cell and tissue imaging (Ramundo Orlando and Gallerano, 2009). Furthermore, THz technologies are recently being increasingly integrated into a host of practical medical, military and security applications. For instance, THz imaging

and sensing techniques are presently being tested at major airports for security screening purposes (Luukanen et al., 2013), at major medical centres for cancer and burn diagnosis (Taylor et al., 2008; Woodward et al., 2003), and at border patrol checkpoints for identification of drugs, explosives and weapons (Federici et al., 2005; Dobroiu et al., 2006; Luukanen et al., 2013).

Moreover, THz radiation is being considered in telecommunications due to several advantages of THz communication links (Federici and Moeller, 2010):

- THz communications have the potential for increased bandwidth capacity compared to microwave systems. They are inherently more directional than microwave or millimetre (MMW) links due to less free-space diffraction of the waves.

- Compared to infrared (IR), there is lower attenuation of THz radiation under certain atmospheric conditions (e.g., fog). Time-varying fluctuations in the real refractive index of the atmospheric path lead to scintillation effects in wireless communications. For THz radiation, these scintillation effects are smaller than for IR radiation, allowing THz to provide longer links compared to wireless IR. Therefore, THz communication links are a viable solution for the last mile and first mile problem (The last and first mile problem refers to establishing broadband, multiuser local wireless connections to high speed networks, such as fibre-optical). As an example, THz wireless links could be used as part of the last mile transmission of multiple channel high definition television (HDTV) signals.

Overall, although THz applications are in their early stage of development, it is expected that general public exposure will take place in the near future, mainly due to security and telecommunications applications. Occupational exposure will also increase as THz imaging systems will be developed and deployed in manufacturing chains for non-destructive quality control. This has raised concerns about health risks and biological effects associated with this type of radiation. Furthermore, the current recommendation of safety limits has been determined using extrapolated estimates from neighbouring spectral regions of millimetre wave on the lower frequency side, and optical radiation on the upper frequency side (ICNIRP, 1996, 1998). There are no specific guidelines generated for this frequency range. In addition, only a few studies have collected experimental data to support these standards.

3.3.9. Discussion on exposure to EMF

Human exposure to EMF occurs from many different sources and in various everyday or exceptional situations. Man-made static fields are mainly found in occupational settings, such as close to MRI scanners, although DC high-voltage transmission lines are being constructed which will expose larger parts of the population to static EMF.

EMF in the ELF range are ubiquitous. The main sources of these fields pertaining to the general public are household appliances and power lines. The electrical appliances that generate higher magnetic fields around them are those that use a motor for their operation. However, in recent years attention has been directed towards people living next to power transformers installed inside residential buildings. It appears that long-term exposure to ELF magnetic field of these people (assessed with spot measurements) can exceed several tenths of µT.

Today practically all electrical equipment uses modern electronics instead of transformers, although the latter are still being used because of galvanic insulation. Examples include all the switched power supplies to laptops and similar devices, chargers to mobile phones etc. In new welding machines there is also a shift to modern electronics with the introduction of IGBT transistors. Welding machines are using more and more a power factor correction that allows to rectify the shape of the current closer to a sinusoid. This, in turn, leads to a ripple current in the tens of kHz range instead of the earlier 50 Hz and harmonic frequencies.

The use of switched power supplies has also led to a change of the frequency content of our daily magnetic field exposure. Since these devices utilize only a small portion of the

50 Hz current, this leads to large harmonics with 150 Hz and higher. With the present electrical wiring with three phases and a neutral, the 150 Hz harmonic is now the dominating frequency in the stray currents in buildings.

In the household, more appliances have appeared in the IF range. It was found that some of them, including playthings, can exceed the limits set by exposure guidelines at close range. An important source of exposure in this frequency range is induction hobs, which have become popular in recent years. These can expose their users (both members of the general public and professionals) to IF magnetic fields higher than those suggested in exposure guidelines, mainly due to the fact that their safety standard requires conformity at a distance of 0.3m only, and does not account for all the different modes and (worst case) use conditions.

By far most applications that emit EMF are in the frequency range above 100 kHz and up to some GHz. Multiple sources exist that contribute to an individual's exposure and under various circumstances. However, transmitters in the close vicinity to or on the body have become the main sources of exposure for the general population and professionals. Distance to the main beam of the source is the main determinant of exposure, given that the emitted power and duty factor remain the same. Especially for brain tissues, the mobile phone used at the ear remains the main source of exposure. However, since the first generation of mobile telephony, there is a trend in the technology of mobile terminals for lower time-averaged emitted power. In particular, for GSM systems, the introduction of power control reduced the output power to about 50% of its maximum during calls, whereas the use of discontinuous transmission (DTX) during voice calls gave a further 30% reduction in average emitted power. Adaptive power control became faster and more effective in the third-generation (3G) of mobile telephony systems leading to a further reduction (by about two orders of magnitude) in the SAR compared to GSM phones. In addition, hands-free kits can lower the energy absorbed by the head drastically. DECT phones which are another source of everyday exposure lead to an SAR which is several times lower than that of GSM phones, although their peak spatial SAR may be smaller by one order of magnitude.

Smart-phones, which operate within networks of different technologies, as well as other portable wireless devices, like tablets and laptop computers, have added complexity to the user's exposure; therefore, combined exposure should be considered for exposure assessment.

The exposure from environmental sources is dominated by broadcasting antennas, antennas from private and official governmental telecommunication services and mobile communications base stations. It has been shown that such systems have significantly increased the EMF levels in the urban environment compared to the levels measured during the 1980's, when only analogue radio and television broadcasting was present. However, historical data from spot measurement campaigns and continuous radiation monitoring systems indicate that the introduction of new technologies after 2G systems, even the emerging 4G systems, did not significantly increase the measured fields in the environment. Indoors, the installation of access points and short-range base stations, such as 3G femtocells, WiFi hotspots and DECT devices, has given rise to exposure at distances within 1 m from them, whereas farther away the emitted EMF does not exceed the common background levels. The emitted power from these devices, even combined, still gives a very low exposure compared with reference levels of European and international guidelines.

Occupational exposure to RF sources at work may lead to a cumulative whole-body exposure of professionals much greater than from their mobile phone use, although the exposure in their head tissues is still expected to be higher from their mobile phone.

In the higher frequencies of the RF range and beyond, i.e. millimetre and submillimetre waves, there are only a few applications currently, but these applications will become more widespread, especially for broadband telecommunications. However, such systems

will operate with low power and, due to the small penetration depth of radiation, only superficial tissues are of concern.

Terahertz applications are also in their early stage of development. General public exposure will be mainly due to security and telecommunications applications, whereas occupational exposure will originate from the introduction of THz imaging systems in manufacturing chains for non-destructive quality control.

3.3.10. Conclusions on exposure to EMF

Human exposure to EMF comes from many different sources and occurs in various situations in everyday life. Man-made static fields are mainly found in occupational settings, such as close to MRI scanners, although DC high-voltage overhead transmission lines are being constructed, which are expected to expose larger parts of the population to static electric and magnetic fields.

EMF in the ELF range are ubiquitous. The main sources of these fields pertaining to the general public are in-house installations, household appliances and powerlines. In recent years, attention has also been directed towards people living next to electric power transformers installed inside residential buildings. It appears that long-term exposure to ELF magnetic field of these people can extent to several tenths of μT.

Today, for power regulation most modern electrical equipment uses electronics instead of transformers. Examples include the switched power supplies to laptops, drilling tools, chargers of mobile phones and similar devices. As a consequence, the frequency content of the daily magnetic field exposure has changed mainly by adding odd harmonics (150 Hz, 250 Hz, 750 Hz, etc.). In particular, the third harmonic (150 Hz) has become another dominating frequency in our environment.

In the household, more appliances have appeared in the IF range. It was found that at close range, some of them, including playthings, can exceed the reference levels set by exposure guidelines. An important source of exposure in this frequency range is induction hobs, which have become popular in recent years. These can expose their users (both members of the general public and professionals) to IF magnetic fields higher than the reference levels of exposure guidelines, mainly due to the fact that their safety standard requires conformity at a distance of 0.3 m only, and does not account for all the different modes and (worst case) use conditions.

By far the most applications which emit EMF are in the frequency range above 100 kHz up to some GHz. Multiple sources exist that contribute to an individual’s exposure. However, transmitters in close vicinity to or on the body have become the main sources of exposure for the general population and professionals. Distance to the source is the main determinant of exposure, together with emitted power and duty factor.

In particular for brain tissues, the mobile phone used at the ear remains the main source of exposure. However, since the first generation of mobile telephony, the technology aimed at reducing the emitted power of mobile handsets. In particular, for GSM systems, already the introduction of dynamic power control reduced the average output power to about 50% of its rated value during calls, whereas the use of discontinuous transmission (DTX) during voice calls gave a further 30% reduction in average emitted power. Adaptive power control became faster and more effective in the third-generation (3G) of mobile telephony systems leading to a further reduction (by about two orders of magnitude) in the specific absorption Specific energy Absorption Rate (SAR) compared to GSM phones. In addition, hands-free kits reduce the energy absorbed by the head drastically. DECT phones are another source of everyday exposure.

Smart-phones, which operate within networks of different technologies, as well as other portable wireless devices, like tablets and laptop computers, have added complexity to the user’s exposure and changed the exposed body region. Due to the different sources used next to the body, it is important to take into account multiple exposure for risk assessment, which may also require organ-specific dosimetry. This issue is also

important for occupational exposure, since there may be situations, such as working in an MRI suite, where professionals are exposed simultaneously to EMF of multiple frequencies ranges, different temporal variations and field strengths.

The exposure from environmental sources is dominated by broadcasting antennas, antennas from private and governmental telecommunication services and mobile communications base stations. It has been shown that such systems have significantly increased the EMF levels in the urban environment compared to the levels measured during the 1980's, when only analogue radio and television broadcasting were present. However, historical data from spot measurement campaigns and continuous radiation monitoring systems indicate that the introduction of new mobile telecommunication technologies after the deployment of the GSM and UMTS systems did not substantially change the average levels of EMF in the environment. At the same time, other technologies, like digital broadcasting, have in some regions contributed to the reduction of EMF exposure from far field sources.

The number of sources has increased indoors. The installation of access points and short range base stations, such as 3G femtocells, WiFi hotspots and DECT devices, has given rise to exposure at very close distances (within 1 m), whereas farther away the emitted EMF does not exceed the common background levels. Consequently, the emitted EMF from these devices, even when combined, still results in a marginal exposure compared to reference levels of European and international guidelines. In general, it appears that, with respect to telecommunication applications, the technological trend is to use low-power emitters, closer to or on the human body, and at higher frequencies.

Millimetre wave and THz applications are expected to be available soon in various industrial environments, such as for imaging systems used for non-destructive quality control, as well as for short-range broadband telecommunications. Currently, they do not significantly affect the average exposure of the general public. These applications will operate with low power and, due to the small penetration depth of the radiation, expose only superficial tissues.

3.4. Interaction mechanisms

Today, well established knowledge allows explaining all scientifically proven biological effects of non-ionizing electric, magnetic and electromagnetic fields and justifies the clear-cut separation between low frequency and radio frequency range. At static fields and in the LF range induced intracorporeal electric fields may cause biologic effects such as cellular stimulation. However, due to basic biologic reasons stimulation ends above about 100 kHz. Consequently, in the RF range EMF energy absorption and subsequent tissue heating becomes the major mechanism. This does not imply that in the LF range energy absorption and heating is not occurring anymore. It just means that their effects simply become negligible compared to the stimulatory effects of the induced electric fields.

The several established biophysical mechanisms through which EMF can interact with living matter help to provide the rationale for limiting human exposures to EMF such as in guidelines from the International Commission on Non-Ionizing Radiation Protection (ICNIRP, 1998; 2010; 2014) and the standards of the Institute of Electrical and Electronics Engineers (IEEE, 2005; 2007). EMF may also have an indirect impact on health such as by causing interference with the functioning of active electronic implants (e.g. pacemakers or cardioverter defibrillators) or other body-worn medical devices and prostheses.

Controversial reports on suggested health risks are explained by various hypothetical mechanisms that have not been verified under permitted exposure conditions and in particular of daily life exposure. As an example, the established mechanisms at low frequencies cannot explain the increased risk for childhood leukaemia that has been reported in epidemiologic studies with much higher than average magnetic field exposure at homes. Similarly at high frequencies, the established mechanisms cannot explain why

there might be an increased risk for glioma and acoustic neuroma as reported in epidemiological studies in heavy users of mobile phones after 10 years or more of use.

Much laboratory research has attempted to determine the chain of molecular and biological events that could lead to the development of neoplastic (or other) disease following exposure to EMF. However, changes in the specific or global expression of genes, proteins or metabolites, or temporal changes in particular hormones are not considered a mechanism *per se* but are considered to reflect a biological response or potential biomarker of exposure. These responses (and similar studies related to other diseases) are described in the relevant sections of this Opinion devoted to describing experimental work.

3.4.1. Static fields

Static electric fields due to the electric conductivity of body tissue cannot induce intracorporeal electric fields. However, static electric fields cause redistribution of electric charges within initially uncharged objects, and in particular charging of the body surface. This leads to electromechanic forces acting upon and between charged hair and may lead to perception or even annoyance. In addition, electrostatic charging may cause discharging at the body surface or to grounded objects. These (micro-) electroshocks can be repetitive and may also lead to perception or even annoyance.

Static magnetic fields interact with living tissues via electrodynamic and magnetomechanical interactions (WHO, 2006).

Electrodynamic interaction occurs at moving charged particles (electrons, ions or dipoles) within the blood flow, within moving tissue such as the beating heart muscle or within moving persons by generating a force (Lorentz force) that separates positively and negatively charged particles and hence induces a movement-dependent electric field.

Magnetomechanical interaction may cause forces which align atoms and particles or with inherent magnetic moments (magnetic dipoles) or at material with diamagnetic, paramagnetic or ferromagnetic properties. In inhomogeneous MF, forces may cause acceleration of ferromagnetic parts and, in strong MF a projectile effect. In daily life, geomagnetic fields are too weak to generate relevant effects. However, the much stronger magnetic fields in and around a MRI scanner at present operating with 0.3 – 9.4 T are strong enough to produce relevant effects which may need protective measures such as access control by detector gates to avoid ferromagnetic devices brought in the vicinity of MRI scanners, to prevent from adverse interference with implanted cardiac pacemakers and – at MF at or above 4T - to prevent from dangerous stimulation of nerves and muscles (and from excess tissue heating by the associated RF EMF) (ICNIRP, 2009).

3.4.2. ELF fields

Time-varying fields (with frequencies up to 10 MHz) result in the induction of alternating electric fields in biological objects. If of sufficient magnitude, these fields can modulate or initiate ongoing activity in the nervous system. Above dedicated thresholds they may also cause stimulation of nerves and, above higher thresholds contraction of muscles.

ELF electric fields similar to static electric fields cause redistribution of electric charges within initially uncharged objects, and consequently charging of the body surface, however, this time oscillating. This leads to periodic electromechanic forces acting upon charged hair and may lead to perception or even annoyance. In addition, alternating electric charging may cause periodic discharges at the body surface or to grounded objects. These repetitive (micro-) electroshocks may also lead to perception or even annoyance.

In contrast to static fields periodic recharging induces intracorporeal electric currents and electric fields strengths which, if strong enough may stimulate nerve and muscle cells.

ELF magnetic fields induce intracorporeal electric field strengths that according to the induction law increase with frequency and cross sectional area. Consequently, inside the body, the strength of induced electric fields increases from zero at the centre to the maximum at the body surface.

3.4.3. RF Fields

In the RF range, which means beyond frequencies that are high enough that cellular stimulation is no longer possible, the EMF energy absorption and subsequent tissue heating becomes the major mechanism. In this frequency range in the far field electric and magnetic field strengths are tightly coupled like the links of a chain.

RF EMF energy absorption is based on oscillating mechanical forces on electric charged particles or electric dipoles generated by the electric component, and, generated by the magnetic field component on particles with an inherent magnetic moment. Depending on the particle's mass and mobility, these forces may cause translatory, oscillatory and/or rotatory movement and hence conversion of field energy to particle's kinetic energy. In the following step, inside the material particle's kinetic energy is distributed by collision with other particles, hence causing irregular particle movement that on a macroscopic scale is named heat (Brown's molecular movement) and quantified by the physical term temperature.

It must be stressed that on a molecular level by its principle nature the interaction of RF EMF is non-thermal. Therefore, the terms *thermal* and *heating* apply to the entire ensemble of particles and have no meaning for individual particles. Consequently, the discussion about thermal and non-thermal effects is misleading. Irrespective of the field amplitude the basic physical interaction mechanism is non-thermal. However the (macroscopic) biochemical and physiological responses depend on temperature. Most chemical properties, chemical reaction kinetics and cellular processes are temperature-dependent. Therefore, any claimed borderline between thermal and non-thermal effects necessarily needs to be defined with regard to specific effects such as triggering the onset of thermoregulatory reactions. Therefore, to generally claim that effects observed below exposure limits would necessarily be non-thermal is misleading and ignores this basic relationship.

If strong enough, time-varying fields (with frequencies above 100 kHz) result in measurable heating of exposed tissues (WHO, 1993, ICNIRP, 1998). If the exposure is sufficiently prolonged or intense, the capabilities of the various elements of the thermoregulatory system to dissipate the absorbed power may be compromised, resulting in increases in (whole-body or local) temperature. Rises of core body temperatures of up to a few degrees stay within the body's intrinsic thermoregulatory bandwidth as demonstrated by the circadian or women's monthly temperature rhythms. Some temperature increase is usually well tolerated, although it might become health relevant at higher values.

In addition, short transient heating such as from microwave radar pulses with high peak power (of a few kW/m^2 or more) can result in thermomechanic effects such as auditory perception described as a hissing, popping or clicking sound: the microwave hearing phenomenon (WHO, 1993; Lin and Wang, 2007). These effects are attributed to the pulses causing a transient localised heating, resulting in an associated tissue expansion, particularly of the liquid within the labyrinth of the inner ear. This generates an acoustic wave that stimulates the auditory receptors.

As indirect effects electric shocks or contact burns can result from touching large, conducting ungrounded objects in strong RF EMF.

Public exposures guidelines for EMF are set to avoid adverse effects on the nervous system and stimulation of nerve and muscle cells and to avoid excess heating of either the whole body or parts of it by limiting exposures to stay sufficiently below the thresholds for these effects with a reduction factor that accounts for uncertainty and interpersonal variability.

3.4.4. Other mechanisms

In addition to the established interaction mechanisms, a number of other mechanisms have been postulated. Many of these candidates are not biophysically plausible, although a few do enjoy limited experimental support. However, none has been firmly demonstrated in humans and their relevance to health remains unclear.

To a large extent, the investigation of potential new relevant mechanisms that operate at low levels of exposure has been hampered by the absence of a robust, reliable and repeatable effect that occurs in any biological model as a consequence of exposure. The search for a new relevant mechanism, if existent, would be greatly improved if such an effect could be established. At present, reports of low-level effects by exogenous fields remain highly controversial and subject to much scientific debate and scrutiny.

In a careful review of potential mechanisms, Sheppard et al. (2008) concluded that the dominant mechanism at RF frequencies was dielectric heating, and that most other possible mechanisms, many based upon direct coupling to specific modes in molecules, cells or tissues, are implausible as means for independent energy deposition. Most potential candidates were excluded because, to be biologically effective, they would be accompanied by such temperature rises that would overwhelm any other biological response. Resonant molecular or submolecular vibrational modes were also excluded because movements are too heavily damped, whilst other suggested mechanisms involve energy that would be far weaker than thermal background. It was also concluded that conditions where an RF field interacts directly with charges or dipoles to cause molecular transformation or damage would require field strengths that are greatly in excess of those that would cause dielectric heating.

Radical pair mechanism

Free radicals are highly reactive and short-lived molecules or ions with unpaired electrons, and they are formed when radical pairs dissociate. Certain metabolic reactions involve an intermediate state comprising a radical pair, usually in a singlet state with the spin of one unpaired electron anti-parallel to the spin of the other (WHO, 2006). These spin-correlated radical pairs recombine to form reaction products. It is well known that an applied magnetic field (with a frequency of less than about 100 MHz) can affect the rate and the extent to which the radical pair converts to the triplet state (parallel spins) in which recombination is no longer possible, and thereby change the reaction yield. Although there is theoretical and experimental evidence for such effects in chemical systems (Challis, 2005; Georgiou, 2010), the dependence on reaction rates is complicated and sizeable effects are usually only seen in special circumstances (Timmel et al., 1998). While extending the lifetime of the radical pair could have serious consequences by affecting the integrity of DNA or other subcellular components and processes (Finkel, 2003), the extent to which these effects could produce oxidative stress-induced tissue damage is not known.

This mechanism has been suggested (Ritz et al. 2000) as a means by which some species of birds and other animals may use the Earth's magnetic field as a source of navigational information during migration, and there is experimental support for this view (Ritz et al. 2004). More recently, Ritz et al. (2009) investigated the behavioural effects of exposure of robins to time-varying magnetic fields of 470–480 nT at frequencies ranging from 0.01 MHz to 7 MHz, and identified a radical pair with a long lifetime involved in the birds' magnetic compass. It was found that fields of around 0.6 MHz and above caused the robins to be disoriented. Ritz and co-workers concluded that a strong resonance at a Larmor frequency of 1.315 MHz (with a geomagnetic field of 46 µT) would be expected for a radical pair in which a radical has a magnetically-isolated spin. This resonance may arise from the interaction of the unpaired electron with the external magnetic field to produce a unique energy-level splitting (Zeeman interaction).

Georgiou (2010) reported some studies with RF fields that provide evidence for the induction of oxidative stress via the free-radical pair mechanism in biological systems. These effects included increased production of reactive oxygen species, an increase in

DNA single-strand breaks, increased lipid peroxidation, and alterations in the activities of enzymes associated with antioxidative defence. Many of these changes were reversed by pre-treatment with antioxidants.

Sheppard et al. (2008) emphasized that despite the number of biochemical reactions involving free radicals, there are many restrictive conditions that would make the effect of RF magnetic fields very unlikely in most systems. These include the frequency constraints based on hyperfine coupling strength, and radical pair interactions restricted by the necessity for creation of spin-correlated radical pairs that remain in close proximity. It is also essential that radical lifetimes are long enough to be affected by a time-varying magnetic field, and relaxation processes are slow enough to allow adequate radical lifetime. In addition, the static magnetic field must have an appropriate field strength.

Magnetite particles

Most biological materials are either diamagnetic of paramagnetic in nature and interact only very weakly with a magnetic field. In contrast, magnetite (Fe_3O_4), a naturally occurring oxide of iron, is ferrimagnetic and interacts with magnetic fields more strongly than any other biological material. Magnetosomes containing biogenic magnetite crystals have been found in certain bacteria and in the cells of many animals, including brain cells in humans (Kirschvink et al., 1992a). Magnetite may play a role in navigation in some fish, birds and turtles although its function in humans remain unclear, and there is very little evidence that humans possess an endogenous magnetic compass. Magnetite has also been found in mouse tumours, suggesting a possible role in cellular functions and implicated in iron transport and storage.

The pathway(s) through which magnetic information could be transduced into relevant biological signals remains largely elusive. However, it has been suggested that magnetosomes may rotate or oscillate in low frequency magnetic fields of sufficient intensity (Kirschvink et al., 1992b, Kirschvink, 1996). If these are coupled to mechanically-sensitive ion channels, this torque may activate the channels and cause them to open or close. The resulting change in ion flux could influence a variety of cellular processes. In addition to power frequency fields, effects could be produced by low frequency fields associated with mobile phone signals.

The ferrimagnetic transduction hypothesis provides a tentative biophysical mechanism to begin to understand how magnetic fields might cause significant biological effects. However, it has been calculated that exposure to time-varying fields of around 50 μT or more are required to overcome constraints imposed by thermal noise (Adair, 1994). Fields of this magnitude may be encountered in the environment but will be limited to parts of the body in close proximity to wiring or electric appliances. Thus it cannot provide an obvious explanation for the increased risk of childhood leukaemia with above average time-weighted fields in the home.

Non-equilibrium and nonlinear effects

Because living, biological systems do not exist at thermal equilibrium, theories on interactions between EMFs and biological tissues must consider the non-equilibrium and nonlinearity of these systems (Georgiou, 2010). Non-linear processes such as rectification could transduce low frequency- modulated RF signals into the frequency range where physiological systems operate (Sheppard et al., 2008).

It has been proposed that biochemical effects may be induced by weak fields in biological systems that are in non-equilibrium states in which the time to transition from an intermediate metastable state to a final active or inactive state may be less than the thermalization time of the induced field (Binhi and Rubin, 2007).

After considering whether protein conformation might be affected by RF fields if amplitudes of specific vibrational modes are altered, Prohofsky (2004) concluded that the biological effects of RF fields on DNA, proteins and similar macromolecules can only be due to temperature changes because the absorbed energy associated with inter-

molecular vibrations is too rapidly converted to heat and coupling to the surrounding water (damping) occurs before the energy can be transferred to intra-molecular resonant modes. A non-thermal effect might still exist, however, if there was a very strong energy coupling between the inter- and intra-molecular modes, and if the damping effect of water can be ignored. These conditions may occur for proteins such as myoglobin or haemoglobin, in which the haem group can oscillate in the protein pocket at lower frequencies.

Another suggestion has been that cells may have the potential to cause non-linear demodulation of a modulated or pulsing frequency to create low frequency fields that then have an effect on tissues. However, it has been concluded that the power of the demodulated signal would be infinitesimally small, at most 77 dB below the carrier, and more probably 90-100 dB below (AGNIR, 2012). Thus the demodulated power at 16 Hz from an incident field of 100 V/m at 900 MHz has been estimated to only about 1 pW. This suggests that any demodulated signal of biological significance would need to be accompanied by a carrier frequency of thermally-destructive power.

Balzano and co-workers designed a doubly resonant cavity to search for nonlinear RF energy conversion in biological material (Balzano et al., 2008). The loaded cavity was resonant at about 890 MHz and at the second harmonic of about 1780 MHz. The sensitivity of the system was considered sufficient to reveal any nonlinearity that could have any potential biological significance, and allowed detection of second harmonic signals above a noise floor as low as -169dBm (Davis and Balzano, 2010). Biological material was exposed to a low-level continuous wave field at input powers of 0.1 or 1□mW at the resonant frequency while monitored for evidence of the generation of the second harmonic (Kowalczuk et al., 2010). A wide range of both cancer and non-cancer cell lines were used, as were cells and tissues previously reported to exhibit responses to weak RF fields. These cells included high-density cell suspensions of human lymphocytes and mouse bone marrow cells, and adherent layers of IMR-32 human neuroblastoma, HF-19 human fibroblasts, N2a murine neuroblastoma and Chinese hamster ovary cells. Also examined were thin slices of mouse tissues, including brain, kidney, muscle, liver, and diaphragm. The cavity was placed within an incubator to ensure optimum conditions for the samples. Over 500 samples were tested but no evidence of non-linear energy conversion by any of the samples was found Overall, since second-harmonic generation is considered to be a necessary and sufficient condition for demodulation, these results do not support the possibility that living cells possess the non-linear properties necessary to demodulate RF energy.

Ca^{2+} ion homeostasis

The possibility that changes in Ca^{2+} flux may occur in cells in response to exposure has long been considered (see reviews by Adey, 1981; WHO, 1993. 2007). Some, but not all studies have reported effects using low frequency electric fields or pulse modulated RF fields, often with effects restricted to specific amplitude or frequency windows (see Pall, (2013) for a review of studies suggesting effects through voltage-gated calcium channels).

One recent study tested this hypothesis using a highly sensitive assay and state-of-the-art techniques. Using an open transverse electromagnetic (TEM) cell housed within a custom-made incubator, O'Connor et al. (2010) exposed cover slides with monolayers of human EA.hy926 endothelial cells, cultured PC-12 neuroblastoma cells or rodent primary hippocampal neurons to CW or GSM 900 MHz signals at 0.012 - 2 W/kg for 30 min. An automated high-throughput imaging technology was used to monitor Ca2+ ion concentrations in the cells using florescence indicators (Fura-2, or Fura-PE3 for hippocampal neurons); real-time images were made every 30s before, during and after exposure. No significant effects on resting or spontaneous Ca2+concentrations were seen for any cell line with either CW or pulsed signals. A second phase of the experiment studied the effects of exposure on endothelial cells following treatment with histamine (to stimulate the release of Ca2+ ions from intracellular stores) or with thapsigargin (to stimulate passive Ca2+ store depletion and Ca2+ entry). It was found that exposure had

no influence on the responses of the endothelial cells to either treatment. Overall, the authors concluded that this highly-sensitive assay did not detect any consequences of RF exposure.

Pearl chain formation

The pearl-chain formation may occur when molecules and cells can move under the influence of an electric field and rearrange to form chains along the direction of the field (Sher et al., 1970; Schwan, 1982; Takashima and Schwan, 1985).

Under the influence of an electric field, electrical charges tend to accumulate on opposite cell surfaces to form induced dipoles, whose orientation changes with oscillations of the field. A dipole–dipole attraction occurs in the process. The attractive forces between the dipoles are enhanced when the objects are in close proximity to each other.

The dipoles then align in the direction of the applied electric field and form chains of many cells or molecules. The chains are mostly single-stranded, although it is possible to form multi-stranded chains. Pearl-chains have been observed within suspensions of erythrocytes or bacteria that allowed sufficient mobility.

At frequencies up to about 100 MHz, the threshold of the electric field strength needed to produce the pearl-chain effect depends on frequency, cell or particle size, the amplitude and pulsation of the applied field. At higher frequencies, the induced dipoles have insufficient time to follow the oscillating field to change their directions. Both (single or multiple) pulses and CW fields are known to produce the pearl-chain effect. Pulsed fields appear no more effective than CW fields in producing the pearl-chain effect.

While these effects have been observed using cell suspensions *in vitro*, they seem unlikely to occur *in vivo*. Blood might be regarded as a possible biological candidate to show this effect, but its complex, dynamic motion means that the erythrocytes and other cells are unlikely to become aligned with the field for appreciable periods.

Reactive oxygen and nitrogen species

Many free radicals are the result of naturally occurring processes such as oxygen metabolism and inflammatory processes, although environmental stimuli such as ionizing radiation and toxins can also increase the levels of free radicals (see Dröge 2002 for a comprehensive review). Reactive Oxygen Species (ROS) along with Reactive Nitrogen Species (RNS) are terms collectively describing radicals and other non-radical reactive oxygen and/or nitrogen derivatives. ROS/RNS are formed in all living organisms as by-products of normal metabolism and as a consequence of exposure to environmental compounds. In biological systems, there are a multitude of reactions that act to reduce the induced increased levels of ROS/RNS, collectively called antioxidants. The antioxidants are substances that are able to regulate oxidative reactions in the way that they compete with other oxidizable substrates; they prevent, delay or inhibit the oxidation of the substances. During normal conditions there is a balance between oxidants and antioxidants (a radical homeostasis is established).

Whereas the reactions of ROS/RNS are fast, the responses of the cell/organism are much slower. Short periods of elevated ROS/RNS levels, less than a few minutes, can manage to initiate protein expression and other regulations, yet they cannot be completed within these short times. Thus, the functional responses, acting on detoxification as well as on the pro-oxidant side, require longer time, over a period of hours or days (Jezek and Hlavata 2005).

A number of experimental studies have investigated whether EMF can influence the radical homeostasis of the cell, and thus act as an initiator of events that can lead to changes in the cell´s status that in turn may be involved in development of disease processes (reviewed e.g. by Simkó and Mattsson, 2004; Consales et al. 2012). Recently, a grouping approach was performed by Mattsson and Simkó (2014) that tested the hypothesis that ELF MF exposure *in vitro* changes the oxidative balance in the cell. The conclusion from the work is that ELF MF (modulated or unmodulated) consistently can

influence the oxidative status, at or above 1 mT, in a broad range of cell types and independent of exposure duration.

3.4.5. Conclusion

Several interactions mechanisms are well established. They allow extrapolation of scientific results to the entire frequency range and wide-band health risk assessment. They have been used to help formulate guidelines limiting exposures to EMF in the entire frequency range from static fields to 300GHz. A number of studies reported other candidate mechanisms, however none that operates in humans at levels of exposure found in the everyday environment has been firmly identified and experimentally validated nor do they allow concluding on potential health risks at other exposure conditions both with regard to amplitude and/or frequency.

3.5. Health effects from THz fields

The previous SCENIHR Opinion did not include health effects from THz technologies, so a brief introduction of this part of the electromagnetic (EM spectrum) is in order.

THz-induced biological effects are strictly related to THz exposure parameters (frequency, power, exposure duration, etc.) and the composition and properties of the biological target (index of refraction, absorption and scattering properties, etc.). These elements can impact the propagation, energy spatial distribution and thermal effects of THz irradiation. For instance, the largest and primary targets are the skin and cornea (since the penetration depth is in the order of 100µm), and many biological macromolecules like DNA, tryptophan, protein and carbohydrates contribute to tissue absorption although water is the main tissue chromophore at THz frequency. Due to water absorption, high power THz field is assumed to cause thermal effects in biological materials, although non-thermal effects have also been proposed (Alexandrov et al., 2011).

The number of studies investigating the biological effects of weak THz field is small, but has increased during the last 10 years due to the availability of reliable sources and detectors. In the following, a review of the main publications dealing with health effects of THz field is provided. Experiments have been described by including THz frequency, exposure duration, power density when applicable, biological systems, investigated endpoint and main results. The main studies addressing the interaction mechanisms of THz field on biological systems have also been included. The *in vivo* and *in vitro* studies that are referred are summarized in Tables 3 and 4 in the following text.

3.5.1. *In vivo* studies

To date the only human study was carried out by Ostrovskiy et al. (2005) and published in the Proceedings of IRMMW-THz. They demonstrated that THz fields could represent a useful tool to induce burn repair and reduce microbial dissemination. They treated a group of 14 and a group of 21 patients suffering from superficial and deep burns respectively, while 2 groups of 15 patients each were employed as controls. Seven to ten 15 min treatments were provided per day in CW mode at the frequency of 0.15 THz, 0.3 W/m2. This resulted in acceleration of the epithelialization process and reduced the microbial dissemination in deep burns by 100 to 1000 fold (Ostrovskiy et al., 2005). Although empirical dosimetric data were not provided by the authors, post-publication measurements performed by Wilmink and Grundt (2011b), demonstrated that the THz-induced temperature rise was roughly 0.1°C, thus corroborating the authors' suggestion that the observed effects are due to the strong absorption of nitric oxide (NO) molecules at THz frequencies and not to thermal mechanisms.

The majority of the *in vivo* experiments on the Albino rat model were carried out, by the Kirichuck group. In the first paper (Kirichuck et al., 2008), by using a microwave generator, they exposed male and female rats (n=180; 60 males and 120 females) for 15 or 30 min to 0.15 THz, 0.7 mW, 2 W/m² after inducing disorders of intravascular components of microcirculation by immobilization stress (a single 3 h fixation of animals

in the supine posture). Platelet aggregation was studied in platelet-rich plasma samples by using a platelet aggregation analyser. Results indicated that both male and females exhibited complete recovery of platelet aggregation, although female rats were more sensitive (15 min treatment was effective in female with respect to 30 min in male rats). In a second paper (Kirichuck et al., 2009), the authors did not confirm their previous observations on platelet aggregation, and as a matter of fact in this study they found the aggregation parameters to be elevated in Albino rats after immobilization and after exposure to 0.15 THz, 30 W/m² for 15, 30 and 60 min. The discrepancy between the papers was not commented on by the authors. In the same paper they found that immobilization stress weakened the animals' orientation abilities (maze designed to test for depression) and the irradiation even increased this weakening.

In a third paper (Kirichuck and Tsymbal, 2009), these authors employed 75 male albino rats divided into 4 groups (control; rats immobilized and not irradiated; rats immobilized and subjected to a single irradiation session for 15 min; rats immobilized and subjected to a single irradiation session for 30 min) to test the effects of terahertz irradiation at the nitric oxide frequencies 150.176-150.664 GHz (0.7 mW radiation power and 2 W/m^2 power density) on the intensity of lipoperoxidation (LPO) and antioxidant properties of the blood subjected to immobilization stress by a supine fixation technique for 3 h to activate lipoperoxidation. They found that 30 min terahertz irradiation completely normalized LPO processes and functional activity of antioxidants in stressed rats. In a fifth group of rats subjected to immobilization stress and irradiated for 30 min at the frequency of 53.54 GHz no reduction of stress parameters was observed, thus confirming the putative role of nitrogen monoxide as a mediator. Subsequently (Kirichuck and Tsymbal, 2010), they demonstrated the efficacy of 30 min terahertz radiation at 129.0 GHz (1 W/m^2) (frequency of the molecular spectrum of radiation and absorption of atmospheric oxygen) on normalizing the hypercoagulation and the suppression of fibrinolysis of blood induced in mongrel white rats by experimental stress as in the previous paper. In a fifth paper, they investigated the effects of electromagnetic radiation at the frequency of NO emission and absorption spectrum 150.176-150.664 GHz (0.7mW radiation power and 2 W/m^2 power density) on peripheral perfusion in albino rats under conditions of acute immobilization stress (rigid fixation in the supine position for 3 h). Laser Doppler Flowmetry (LDF) was performed using a laser blood flow analyser, whose transducer was fixed on the dorsal surface of the right paw using a-traumatic patch and LDF software. 30 min THz exposure resulted in correcting disturbance in peripheral circulation (Kirichuk et al., 2011).

The possibility to treat hemodynamic disorders accompanying some of pathologic diseases has also been demonstrated (Kirichuk et al., 2012). Albino rats, in which immobilization stress once again caused hemodynamic disorders, were exposed by using Orbita, an extremely high frequency therapy apparatus for hemodynamic, fibrinolytic and peripheral perfusion disorders treatment, to continuous terahertz radiation with frequencies equal to absorption and emission frequencies of nitrogen oxide (150.176-150.664 GHz) and atmospheric oxygen (129.0 ± 0.75 GHz), and 1 W/m^2 power density for 3 cm^2 skin area. Exposures of 5, 10 and 15 min in both conditions allow for reverting the post-stress hemodynamic changes in great vessels.

In the latest study from the same group (Kirichuk and Tsymball, 2012), they found that the positive effects of the THz field, at atmospheric oxygen frequency of 129 GHz on blood nitrite concentration of exposed male white rats under acute and chronic immobilization stress, were negated upon preliminary treatment with L-NAME, a non-selective inhibitor of NO-synthase, thus demonstrating the involvement of constitutive NO-synthase in the mechanisms of positive effects.

The effects of THz waves on the behaviour of mice were investigated by Bondar and co-workers (2008). Male adult C57BI/6J mice were kept in a metal cage divided into 2 compartments with a transparent barrier with holes. By means of a hole in the metal cage, at the level of mouse body and at a distance of 3 cm from the barrier, the radiation beam entered the cage and was reflected inside the cage by another hole with a mirror in

the opposite wall, to expose mice at 3.6 THz, (about 50 W/m^2) for different time periods from 5 to 30 min. There were no changes in behaviour of animals with respect to the barrier or to the mouse into the adjacent compartment, while significant reduction in sniffing the hole allowing entry of radiation and time spent in its proximity were recorded as compared to the controls. Delayed effects of 30 min THz irradiation were also detected one day after exposure by the anxiety of experimental mice with respect to control by means of the orientation test in a maze, thus the authors concluded that mice exposed to radiation show anxiety.

The effect of THz waves on the inflammatory response in skin has been investigated recently by Hwang et al. (2014). They employed a genetically engineered Tie2-eGFP mouse model to analyse cellular level inflammatory response after pulsed THz wave irradiation. In particular, ear skin of a live anaesthised mouse was irradiated for 30 min with pulsed THz wave (2.7 THz, 4 μs pulse width, 61.4 μJ per pulse, 3 Hz repetition) generated from a compact free electron laser. Average power density was 260 mW/cm^2, and the number and distribution of neutrophils, which are the first immune cells to rapidly migrate to an inflammation site, in the ear skin were monitored before and after THz irradiation by using a custom-built intravital laser-scanning confocal microscopy system. A massive recruitment of newly infiltrated neutrophils was observed in the irradiated skin 6 hours after exposure. Histological analysis also confirmed an accumulation of inflammatory cells in the dermis of the irradiated skin in absence of observable changes in the skin structure. Overall the results suggest that THz irradiation is capable of initiating an acute inflammatory response without structural disruption of the irradiated skin.

In conclusion, taken together, the *in vivo* studies mainly showed beneficial effects of THz field on disorders of intravascular components of microcirculation in rats under immobilization stress, while an indication of negative effects was recorded on behaviour of experimental animals which showed increased anxiety compared to control animals. In all cases, further experiments are needed to support these findings. Studies so far also suffer from a lack of adequate dosimetry. Moreover, *in vivo* investigations on acute and chronic toxicity and carcinogenesis are mandatory in evaluating health risk related to THz frequencies.

Table 3. ***In vivo*** **studies on THz technologies**

Reference	Sample/Model	Exposure conditions	Results
Ostrovsky et al., 2005	14 patients with superficial burns<br>21 patients with deep burns	0.15 THz (CW),<br>0.3 W/m^2,<br>7 to 10 treatments of 15 min	Acceleration of epithelialization process and reduced microbial dissemination
Kirichuk et al., 2008	Albino rats	0.15 THz,<br>2 W/m^2, 15 min	Recovery of platelet aggregation induced by immobilization stress
Kirichuk et al., 2009	Albino rats	0.15 THz,<br>30 W/m^2,<br>15-60 min	Increase of platelet aggregation parameters. Increased weakness in orientation abilities.
Kirichuk and Tsymbal, 2009	Albino rats	0.15 THz (nitric oxide frequencies);<br>53.54 GHz,<br>2 W/m^2, 30 min	Reduction of stress parameters induced by immobilization stress at nitric oxide frequencies, no effects at 53.54 GHz.
Kirichuk and Tsymbal, 2010	Mongrel white rats	0.13 THz, 1 W/m^2, 30 min	Recovery of hypercoagulation and suppression of fibrinolysis induced by immobilization stress.
Kirichuk et al., 2011	Albino rats	0.15 THz 2W/m^2,<br>30 min	Recovery of disturbance in peripheral perfusion induced by acute immobilization stress.

Kirichuk et al., 2012	Albino rats	0.15 THz;0.13 THz, 1 W/m^2,<br>5, 10, 15 min.	Reversion of post immobilization stress hemodynamic changes.
Kirichuk and Tsymbal, 2012	White rats	0.13 THz exposure +/- L-NAME, an inhibitor of NO sintase.	Positive effects of exposure on blood nitrite concentration negated by L-NAME.
Bondar et al., 2008	C57B1/6J mice	3.6 THz, 50 W/m^2, 5-30 min.	Mice recognize radiation showing anxiety.
Hwang et al., 2014	A genetically engineered Tie2-eGFP mouse model	2.7 THz, 4 µs pulse-width, 61.4 µJ per pulse, 3 Hz repetition;<br>260 mW/cm^2 ; 30 min	Massive recruitment of newly infiltrated neutrophils 6 hours post exposure.

3.5.2. *In vitro* studies

Human cell types

Some investigations deal with cells from human skin since THz field cannot penetrate deep into the human body but can likely affect the skin.

The research group of Clothier (Clothier et al., 2003; Bourne et al., 2008), focusing on human primary keratinocytes (NHKs) and neural cell cultures, ND7/23 cell line, investigated the effects of THz field in the range 0.1-2.7 THz (240-620 W/m^2) for time periods varying from 10 min to 24 h. The differentiation was monitored via the incorporation of fluorescein cadaverine into the cornified envelops. This differentiation assay was combined with the assessment of cell viability by resazurin assay. Primary cultures of NKS express adhesion molecules that comprise part of the natural barrier function of the skin, and the effects of exogenous agents on this barrier function can be measured. Absence of effects on cell differentiation and barrier forming and viability following THz exposure was found. Furthermore human corneal epithelial cells were also investigated which would also be likely exposed to the THz field *in vivo*. Their ability to differentiate in a normal way is important as the eye is potentially less protected than the skin. Again, after two cycles of 24 h exposure, with a 48 h interval between the exposures, no adverse effects were found on cell viability and barrier function. Authors also evaluated effects of 24 h exposure on glutathione (GSH) and heat shock protein 70 levels in NHKs before and after differentiation and no stress response was detected.

Human dermal fibroblasts were employed by Wilmink and co-workers (2011a) to investigate cellular and molecular response to THz field exposure. *In vitro* exposures of 5, 10, 20, 40, or 80 min were performed in a temperature-controlled chamber using a molecular gas THz laser (2.52 THz, 848 W/m^2). Both computational and empirical dosimetric techniques were conducted using finite-difference time-domain (FDTD) modelling approaches, infrared cameras, and thermocouples. Cellular viability was assessed using conventional MTT assays. In addition, to determine if protein and/or DNA damage occurred, qPCR was employed to quantify the transcriptional activation of genes involved in protein and DNA sensing and repair pathways. Comparable analyses were also conducted for hyperthermic (40°C for 5, 10, 20, 40, or 80 min) and genotoxic (3 min UV lamp exposure, 254 nm and 38 W) positive controls. They found that cellular temperatures increased by 3°C during all THz exposures, and equivalent levels of cell survival (≥90%) and heat shock protein expression (3.5-fold increases) in the THz and hyperthermic exposure groups for each exposure duration. In addition, the expression of DNA sensing and repair genes was unchanged in both groups; however, appreciable increases were observed in the genotoxic controls. In this paper, computational modelling techniques to simulate the thermal history of cells exposed to THz field were employed, and authors concluded that 2.52 THz bioeffects may be accurately predicted with conventional thermal damage models (Wilmink et al., 2011a).

In two more recent papers from Hintzsche and co-workers, human primary dermal fibroblasts (HDF cells) and a keratinocyte cell line (HaCaT) were exposed to THz field in

different conditions to evaluate primary DNA damage (comet assay) and chromosomal damage (micronucleus assay). In the first paper (Hintzsche et al., 2012), cell cultures were exposed from below with a collimated Gaussian beam at 0.106 THz in a modified incubator at defined environmental conditions for 2 h, 8 h, and 24 h with different power density ranging from 0.4 W/m^2 to 20 W/m^2, representing levels below, at, and above current safety limits. Neither DNA strand breaks nor alkali-labile sites, in the comet assay, or chromosomal damage in the form of micronucleus induction were detected. In the second paper (Hintzsche et al., 2013), human skin cells (HDF and HaCaT) were exposed *in vitro* to terahertz radiation for 2 and 8 h at the specific frequencies of 0.380 and 2.520 THz, with power density ranging from 0.3-9 W/m^2. Chromosomal damage was not detected in the different cell types after exposure to radiation of both frequencies. In addition, cell proliferation was quantified and found to be unaffected by the exposure, and there was no increase in DNA damage measured in the comet assay for both frequencies.

In two recent papers from the same research group, an artificial human skin tissue model (EpiDermFT, MatTek), resembling normal tissue structure, was employed to address the effects of THz wave on human skin tissue. The model consists of normal, human derived epidermal keratinocytes and dermal fibroblasts that form a multi-layered, highly differentiated model of human dermis and epidermis. It is mitotically and metabolically active and preserves the arrangement and communication of cells in skin tissue *in vivo*. Titova et al.(2013a) exposed tissue samples at room temperature (21°C) for 10 min at the focus of a 1 kHz pulsed THz beam with high and low THz energy of 1 μJ and 0.1 μJ, respectively. The time averaged-THz power density was 570 W/m2 at the highest THz pulse energy, and the temperature increase was estimated to be less than 0.7 °C. The presence of phosphorylated H2AX (γH2AX) was measured. A significant induction of γH2AX phosphorylation was detected, which that is one of the earliest and most characterized cellular responses to DSBs, thus indicating DNA damage. At the same time, THz exposure resulted in the increase of the expression level of various proteins that take part in cell cycle control and DSB repair processes. Titova et al., (2013b) exposed the same artificial human skin tissue to picoseconds-duration broadband (0.2-2.5 THz) THz pulses with 1 KHz repetition rate, 1/e2 spot-size diameter of 1.5 mm and pulse energy of 1.0 μJ or 0.1 μJ. Tissue samples in single well plates were placed at the focus of the pulsed THz beam for 10 min 30 min after irradiation, excised 2 mm-diameter exposed portions of the tissues were analysed using an Illumina HumanHT-12 v4 Espression BeadChip. Exposure to THz pulses profoundly affected gene expression in directly exposed human skin. The levels of 442 genes and the levels of 397 genes resulted affected after 10 min exposure to THz pulses with energy of 1.0 μJ and 0.1 μJ, respectively, when compared to unexposed controls. 219 differential expressed genes were common for both THz pulse exposure regimes; among them 164 were down regulated, and 55 were up regulated and include genes involved in the etiology of dermatological diseases and cancer, but also genes with key functions in apoptotic signal pathways. Since the induced changes in transcription levels are opposite to disease-related changes, authors hypothesized potential therapeutic applications of intense THz pulses.

Human epithelial cells and embryonic stem cells were studied by Williams et al. (2013). They exposed human corneal epithelial (HCE-T), human retinal pigment epithelial (ARPE-19) and human embryonic stem (hES07) cells, at frequencies up to 0.5 THz in different conditions to evaluate cell morphology and proliferation (phase contrast microscopy and BrdU uptake), attachment (cytoskeleton staining), and differentiation (immunostaining). Confluent ARPE-19 cell cultures were irradiated for 3 h (1.8 W/m^2 average power density) and their morphology and growth observed immediately after exposure and for various time up to several days. Subconfluent cultures of both the ARPE-19 and HCE-T epithelial cells were exposed (1.4 to 3.7 W/m^2 average power density) for periods of the order of 3 h, to test the effects of exposure time, the influence of multiple exposures and the influence of irradiation on longer term cell behaviour, such as the subsequent cell proliferation after sub-culturing. hES07 cells were exposed (0.2 to 2.9 W/m^2) for variable

duration (2-6 h) to evaluate the effects on attachment, proliferation and differentiation. It was found that epithelial cell cultures did not show any effects in terms of cell morphology or proliferation, irrespective of the specific cell type, exposure time and multiple exposures. Similar results were observed in embryonic stem cells that also demonstrated that they maintain their undifferentiated phenotype after THz irradiation.

Human blood cells have also been investigated, mainly in the framework of the EU funded THz-BRIDGE project (http://www.frascati.enea.it/THz-BRIDGE/).

Zeni et al. (2007), using a Free Electron Laser and a specific THz delivery system to irradiate whole blood samples, exposed human blood samples from 17 healthy donors for 20 min to Terahertz radiation, and different electromagnetic conditions were considered. In particular, the frequencies of 120 and 130 GHz were chosen: the first one was tested at 0.5 W/m^2, while the second one was tested at 0.3-2.3 W/m^2. In this paper, Specific energy Absorption Rate (SAR) values were also calculated that resulted in 0.4 mW/g and 0.24, 1.4, and 2 mW/g for 120 and 130 GHz respectively. Chromosomal damage was evaluated in PHA stimulated whole blood cultures established after irradiation, by means of the cytokinesis block micronucleus technique, which also gives information on cell cycle kinetics. Moreover, human whole blood samples exposed to 130 GHz at SAR levels of 1.4 and 2 mW/g were also tested for primary DNA damage by applying the alkaline comet assay immediately after exposure. The results obtained indicated that THz exposure, in the explored electromagnetic conditions, was not able to induce chromosomal damage or alteration of cell cycle kinetics in PHA stimulated human blood lymphocytes, and primary DNA damage in human leukocytes from healthy subjects.

Korenstein–Ilan et al. (2008), applied continuous-wave (CW) 0.1 THz field (0.31 W/m^2) to PHA stimulated human lymphocytes isolated from whole blood samples from healthy volunteers and cultured according to standard protocol. After 1, 2 and 24 h exposure, they examined the changes in chromosome number of chromosomes 1, 10, 11 and 17 and changes in the replication timing of their centromeres using interphase fluorescence in situ hybridization (FISH). Chromosomes 11 and 17 were shown to be the most vulnerable (about 30% increase in aneuploidy after 2 and 24 h of exposure), while chromosomes 1 and 10 were not affected. Changes were also observed in the asynchronous mode of replication of centromeres 11, 17 and 1 (by 40%) after 2 h of exposure and of all four centromeres after 24 h of exposure (by 50%). Authors speculated that the induced genomic instability was likely caused by radiation-induced low-frequency collective vibrational modes of proteins and DNA (Korenstein-Ilan et al., 2008).

Rodent cell types

Berns and Bewley (1987) investigated the effects of pulsed 1.5 THz field on a rat kangaroo kidney cell line (PTK2). They used Free Electron Laser to expose cells at room temperature to 10, 20 or 100 pulses of 100 W/cm^2 for 1-10 min; 1 W/m^2 average power density. They examined cell morphology by means of standard light microscopy and did not observe any changes either immediately after irradiation and 3 h post exposure. Small changes were observed only 20 h post exposure. DNA synthesis, measured by means of ^{3}H thymidine isotopes and autoradiographic analysis was found to be inhibited after long exposure. The same group also found DNA synthesis inhibition in either synchronized S phase or unsynchronized Chinese Hamster Ovary (CHO) cells under 5-10 min exposure to 1.5 THz field, 1 W/m^2 average (Berns et al., 1990, 1994).

Bock et al. (2010) exposed mouse mesenchymal stem cells (MSC) to a broadband THz field (~ 10 THz), average power density of 10 W/m^2 for 2, 4, 6 and 9 h. By looking at morphological changes, a significant accumulation of lipid-like droplets in the cytoplasm was evident after 9 h THz irradiation. By looking at global gene expression (Affymetrix mouse genome microarray), many of the MSC genes did not respond at all (89%), certain genes were activated (6%), while still other genes were repressed (5%) significantly after 9 h irradiation. In the group of activated genes, confirmed by mRNA level quantification by using RT-PCR, the over-expression of transcription factor

peroxisome proliferator-activated receptor gamma (PPARG) that is known to be required for adipocyte differentiation, suggested that a THz field, in the specific exposure conditions, enhanced the differentiation process towards an adipocyte-like phenotype in MSC. Authors proposed that a THz field could represent a potential tool for activation of cellular differentiation.

More recently, the same research group in a follow up of the previous study (Alexandrov et al., 2011), investigated the effects of both pulsed and CW THz field on hyperthermic genes (i.e. genes that usually respond to temperature increases in the cell) in MSCs. Low-power radiation from both a pulsed broad-band (centred at 10 THz) source (10 W/m^2) and from a CW laser (2.52 THz) source (~30 W/m^2) was applied for 2 and 9 h. Modelling, empirical characterization, and monitoring techniques were applied to minimize the impact of radiation-induced increases in temperature. qRT-PCR was used to evaluate changes in the transcriptional activity of selected hyperthermic genes. Temperature increases were minimal, and the differential expression of the investigated heat shock proteins (HSP105, HSP90, and CPR) resulted unaffected, while the expression of certain other genes (Adiponectin, GLUT4, and PPARG) showed clear effects of the THz irradiation after prolonged, broad-band exposure.

Hintzsche et al. (2011), investigated and quantified the production of spindle disturbances in A(L) cells, a human-hamster hybrid cell line, by a 0.106 THz field (CW). Monolayer cultures in petri dishes were exposed for 0.5 h to a 0.106 THz field with power densities ranging from 0.43 W/m^2 to 43 W/m^2 or were kept under sham conditions (negative control) for the same period. As a positive control, 100 µg/ml of the insecticide trichlorfon, which is an aneuploidy-inducing agent, was used for an exposure period of 6 h. During exposure, the sample containers were kept at defined environmental conditions in a modified incubator as required by the cells. Based on a total of 6,365 analysed mitotic cells, the results of two replicate experiments suggest that 0.106 THz field is a spindle-acting agent as predominately indicated by the appearance of spindle disturbances at the anaphase and telophase (especially lagging and non-disjunction of single chromosomes) of cell divisions. The authors claimed that their findings do not necessarily imply disease or injury but may be important for evaluating possible underlying mechanisms.

In conclusion, taken together, the *in vitro* studies differ greatly for exposure characteristics and duration, cell type, biological endpoint and do not allow for any conclusion. Concerning genotoxicity, due to the close correlation between DNA damage and cancer occurrence, and the importance of genomic instability in assessing the potential health effects of radiation, the conflicting results presented here deserve future attention.

Table 4. *In vitro* studies on THz technologies

Reference	Cell type	Exposure conditions	Results
Clothier et al., 2003;<br>Bourne et al., 2008	Human primary keratinocytes (NHKs); neural cell cultures (ND7/23);<br>human corneal epithelial cells	0.1-2.7 THz (CW),<br>240-620 W/m^2,<br>10 min – 24 h	No effect on cell differentiation, barrier forming and cell viability.<br>No stress response (glutathione and heat shock protein level)
Wilmink et al., 2011a	Human dermal fibroblasts	2.52 THz, 848 W/m^2,<br>5-80 min	3.5 fold increase in heat shock protein expression as a result of 3°C temperature increase during THz exposure.<br>No effect on cell viability and on DNA sensing and repair gene.
Hintzsche et al., 2012	Human primary dermal fibroblasts (HDF); keratinocytes cell line	0.106 THz,<br>0.4-20 W/m^2, 2-24 h	Neither DNA damage nor chromosomal damage.

	(HaCaT)		
Hintzsche et al., 2013	HDF and HaCaT	0.38 and 2.52 THz,<br>0.3-9 W/m^2, 2 and 8 h	No effect on DNA and chromosomal damage; no effect on cell proliferation
Titova et al., 2013 a	Human skin tissue model	THz pulses peaked at 0.5 THz,<br>570 W/m2, 10 min	Increased γH2AX phosphorylation.<br>Increased expression level of cell cycle and DNA repair related proteins.
Titova et al., 2013 b	Human skin tissue model	THz pulses (0.2 -2.5 THz),<br>1.0 or 0.1 μJ, 10 min	Changes in transcription levels of genes involved in the etiology of skin diseases and cancer, but also genes with key functions in apoptotic signal pathways.
Williams et al., 2013	Human epithelial cells (HCE-T, corneal and ARPE 19, retinal) and human embryonic stem cells (hES07)	Up to 0.5 THz,<br>0.2-3.7 W/m^2, 2-6 h	No effect on cell morphology and proliferation irrespective of cell type, stage of cell growth before exposure, exposure time and schedule (multiple exposure).
Zeni et al., 2007	Human blood samples	0.12 THz (0.5 W/m^2); 0.13 THz (0.3-2.3 W/m^2); 20 min	Neither genotoxic effects (DNA and chromosomal damage) nor alteration of proliferation in human peripheral blood lymphocytes.
Korenstein-Ilan et al., 2008	Isolated human peripheral blood lymphocytes	0.1 THz, 0.31 W/m^2,<br>1, 2 and 24 h.	30% increase in aneuploidy of chromosomes 11 and 17 after 2 and 24 h exposure.
Berns and Bewley, 1987	Rat kangaroo kidney cell line (PTK2)	Pulsed 1.5 THz field,<br>1 W/m^2, 1-10 min	No change in cell morphology immediately post exposure; small change 20 h later.
Berns et al., 1990, 1994	CHO cells	Pulsed 1.5 THz field,<br>1 W/m^2, 5-10 min	DNA synthesis inhibition in S phase synchronized or unsynchronized cells.
Bock et al., 2010	Mouse mesenchymal stem cells (MSC)	10 THz, 10 W/m^2,<br>2, 4, 6, 9 h	Accumulation of lipid-like droplets in the cytoplasm and 6% activated genes after 9 h exposure. Over-expression of a transcription factor (PPARG) related to adipocyte differentiation.
Alexandrov et al., 2011	Mouse mesenchymal stem cells (MSC)	10 THz, (10 W/m^2) and 2.52 THz (30 W/m^2),<br>2 and 9 h	Over-expression of Adiponectin, GLUT4 and PPARG after 9 h exposure at 10 THz
Hintzsche et al., 2011	Human hamster hybrid cell line	0.106 THz (CW),<br>0.43-43 W/m^2, 30 min	Spindle disturbances at anaphase and telophase.

Studies on mechanisms

The most relevant studies on possible mechanisms of effects of THz fields on biological systems are quoted in this section. They mainly originate from the Frohlich studies (1968, 1975) that postulated that the homeostasis of living systems is assured by the flow of free energy through a coherent exited state maintained by metabolic processes, and predicted that biological objects are able to support, under defined conditions, coherent excitations in the range 10^9-10^{12} Hz. As a matter of fact, based on this assumption, THz field exposure might be expected to affect biological processes and

living systems. Theoretical models have been developed to support the onset of non-thermal effects of THz fields. They are mainly based on the fact that the energy scale of a THz field is within the range of hydrogen bonds, van der Waals interactions, and charge-transfer reactions and thus, through nonlinear resonance mechanisms, such fields may have a significant effect on biomolecules and cells (Chitanvis, 2006). Some theoretical works have addressed this possibility. Recently, a fascinating approach has been proposed by Alexandrov et al. (2010). It predicts that high electric fields can generate localized modes of vibration in DNA molecule and that THz excitation could induce and drive conformational changes. They showed that THz field could cause dynamic separations of the DNA double strands, and claimed that the nonlinear resonance mechanism is active even for small amplitudes of the THz field, but it is probabilistic and therefore requires extended exposure. The conformations generated through this mechanism can subsequently affect molecular processes involved in gene expression and DNA replication. The observation on the influence of THz fields on the natural dynamics of DNA was confirmed in the study by Swanson (2011); furthermore, he showed that parameter variation can eliminate breather modes entirely or make them unrealistically strong, that thermal noise completely dominates the external influences of the system, and that it is extremely unlikely that double stranded DNA denaturation can be induced by experimentally accessible THz fields.

Overall, the relevance of these mechanisms is questionable, since the postulated effects have not been experimentally verified at permissible exposure levels.

3.5.3. *Discussion on health effects from THz fields*

A proper risk assessment on health effects from THz exposures is difficult to perform since little pertinent evidence is available due to the small number of investigations carried out so far. Most of the studies have been performed in the last decade, mainly in the frequency range of 0.1-1 THz. Only very few investigations are available on higher frequencies. *In vivo* studies mainly indicate beneficial effects on disorders of intravascular components of microcirculation in rats under immobilization stress, but do not address acute and chronic toxicity or carcinogenesis. *In vitro* studies on mammalian cells differ greatly with respect to irradiation conditions and endpoints under investigation. Studies suggesting effects of exposure have not been replicated in independent laboratories. Some theoretical mechanisms have been proposed, but they are difficult to accept since no conclusive experimental evidence is available.

More systematic research is needed for any firm conclusions to be drawn on the health effects from exposure to a THz field. In particular, broader frequency ranges are to be investigated. Human and animal studies should address specific endpoints related to possible toxic effects on the skin and the cornea. Positive studies need to be replicated in independent laboratories.

Considering the expected increase in use of THz technologies, more research focusing on the effects on skin (long-term, low-level exposure) and cornea (high-intensity, short-term exposure) is recommended. In addition, monitoring of occupationally exposed groups for skin and eye changes and disorders would be useful.

3.5.4. *Conclusion on health effects from THz fields*

The number of studies investigating potential biological, non-thermal effects of THz fields is small, but has been increasing over recent years, due to the availability of adequate sources and detectors. However, scientific studies on potential health effects from long-term exposure are still lacking.

In vivo studies indicate mainly beneficial effects on disorders of intravascular components of microcirculation in rats under immobilization stress, but do not address acute and chronic toxicity or carcinogenesis. *In vitro* studies on mammalian cells differ greatly with respect to irradiation conditions and endpoints under investigation. Studies suggesting effects of exposure have not been replicated in independent laboratories. Some

theoretical mechanisms have been proposed, but no conclusive experimental support is available.

Considering the expected increase in use of THz technologies, more research focusing on the effects on skin (long-term, low-level exposure) and cornea (high-intensity, short-term exposure) is recommended.

3.6. Health effects from RF fields

3.6.1. *Neoplastic diseases*

3.6.1.1. Epidemiological studies

Brain tumours and other tumours of the head and neck area

What was already known on this subject?

In the previous SCENIHR Opinion adopted in 2009, it was concluded that the evidence from epidemiological studies indicates that the use of mobile phones for less than ten years was not associated with an increased risk of developing a brain tumour. A major limitation however was that only few longer term users were included in those studies, circumventing firm conclusions related to long-term mobile phone use. In addition, it was noted that any conclusions of risk after induction periods of more than 20 years were not possible due to the short lifetime of the technology.

What has been achieved since then?

Exposure considerations for mobile telephony

Exposure assessment in epidemiological studies of mobile phone (MP) users is complicated due to the complex and varying use patterns and power control as well as the rapid changes of technologies and devices. As a first proxy the exposure has been assessed comparing users versus non-users. The next step has been to use the cumulative call time. . However, a long-term user has often used more than one phone model, and sometimes also more than one mobile phone system (analogue and digital systems). It is particularly complicated to combine the use of different phones with different power outputs, systems, frequencies and intracorporeal distributions of the Specific energy Absorption Rate (SAR) into one exposure measure.

Different mobile phones have different output power and the change is quite large when first generation phones are compared with today's models. The old analogue phones had an output power of 1 W and it was seldom down-regulated due to the long distance between base stations. The 2nd generation GSM phones operate with a peak power of 2 or 1 W for the 900 and 1800 MHz band, respectively. This is then down-regulated depending on to the quality of the connection to the base station. Lauer et al. (2013) give the average output power as 133 mW for GSM 900 and 62 mW for GSM 1800. Persson et al. (2012) report that the average terminal output power for 3G voice calls was below 1 mW for any environment including rural, urban, and dedicated indoor networks. The median value was of the order of 10 µW. For DECT phones the rated output power is 10 mW.

Hansson Mild et al. (2005) used the average output as a weight factor for the call time on the various phone types, NMT, GSM and DECT. However, since the NMT operate at a much higher output power, the weighting made their use dominant. In another attempt Cardis et al. (2011a) tried to estimate the radio frequency (RF) dose as the amount of mobile phone RF energy absorbed at the location of a brain tumour. They quantified all the main parameters thought to influence the amount of the total cumulative specific RF energy from mobile telephone use (in joules per kilogram), or dose, absorbed at a particular location in the brain. This algorithm was then applied to Interphone Study subjects in five countries (Cardis et al. 2011b).

With regard to the dynamic changes in technology, exposed body regions and use patterns, exposure assessment in epidemiological studies of users of mobile telecommunication devices such as smart phones, tablets etc. faces severe problems. In view of the lack of verification of any proposed non-thermal interaction mechanism, established knowledge does not suggest effects accumulating with time. Beyond that, there is no sound scientific basis for defining additional dose-dependent exposure parameters.

Brain tumours

A working group at the International Agency for Research on Cancer (IARC) within the Monograph program on the evaluation of carcinogenic risks to humans classified the epidemiological evidence for glioma due to mobile phone use as limited, but extended the conclusion to the entire RF range and classified it as possibly carcinogenic to humans (IARC 2013, Baan et al., 2011).

Whether the use of mobile phones is associated with an increased risk of brain tumours has been the research question of numerous small and a handful of large-scale epidemiological studies. Attention has focused on the possibility of tumours of the head and neck region because these tissues are primarily exposed to the RF fields emitted by hand-sets.

Several studies were completed just between the last SCENIHR Opinion (SCENIHR, 2009) and today, allowing now a more thorough assessment especially regarding longer-term use over more than a decade. The association between mobile phone use and brain tumour risk was investigated with three different study designs, including ecological studies, i.e. age- and sex-specific time trend analyses of brain tumour incidence rates; case-control studies; and cohort studies. Due to the inherent strengths and weaknesses related to each of the approaches, their results complement each other and contribute to the overall picture.

Case-control studies are common in cancer epidemiology because cancer subtypes are rare diseases, and this approach involves comparing exposure patterns in persons with the disease of interest to a random sample of non-diseased from the same source population. In case-control studies, exposure is often assessed by personal interviews that, since collecting detailed personal information is possible, allows for a rather detailed modelling of exposure. Limitations of case-control studies include i) the challenge of establishing a truly representative control group, given that many countries lack a good framework for random sampling and, since active participation is required, that selection bias may result if participation is related to the exposure of interest; ii) the challenge of recruiting the cases especially for a disease with poor prognosis and, with regard to brain tumours, that symptoms of the disease may include memory difficulties; and iii) exposure estimation mainly based on recall of study subjects, which may give rise to recall bias generally overestimating a possible effect. Self-reported exposure also suffers from substantial misclassification as detailed information on everyday activities in the distant past is sought, such as the amount of mobile phone use more than 5-10 years earlier.

Cohort studies follow the direction of aetiology by assessing exposure prior to occurrence of disease, but when investigating a rare disease very large numbers of participants are needed. With such large numbers, exposure assessment is often crude. In addition, a system for tracing study subjects to collect information on disease occurrence needs to be in place. Unsurprisingly, given these demands, no prospective study with detailed exposure information has been completed, although one study has been underway in Europe since 2007 (Schüz et al., 2010). The only cohorts that provided results are a Danish cohort study of mobile phone subscribers and the UK Million Women Study, both described below. Cohort studies are not free of bias; once again, selection bias (comarability of study groups) may be of some concern, although not to the same extent as in case-control studies if within-cohort comparisons are made; exposure assessment is often a weakness as it is often crude and sometimes based on self-reported

information with uncertain accuracy. However, an advantage compared to case-control studies is that exposure information is collected before occurrence of the disease, and therefore the reporting of exposure information is unrelated to disease status avoiding recall bias.

Ecological studies are prone to ecological fallacy; due to lack of data at the individual level, findings may reflect cases that occur in the unexposed segments of the population. With regard to mobile phone use, ecological studies based on high-quality cancer registry information (nearly complete coverage of the cancer cases) have some value if one assumes an effect with already modest mobile phone use, as then exposure prevalence has increased sharply to cover the vast majority of the population and would affect the incidence time trends; however, if an effect were restricted to, for example a small proportion of very heavy users in the population, such an effect may be missed in the trends unless heavy users can be identified. An example of such a method was a study exploring links between brain cancers and various environmental factors in 165 countries for generating hypotheses (de Vocht et al. 2013). They reported higher incidence rates of brain cancers in countries with the most frequent mobile phone subscriptions. The study is not informative for causal inference, as popular use of mobile phones can also reflect standard of living, which is also associated with, for example, availability of diagnostic medical services.

Ecological studies on the other hand can be used for consistency checks that extrapolate the risk estimates from case-control or cohort studies to surveillance data and compare the expected with the observed changes in time trends. This approach is strong as it is based on objective and comprehensive data, when the predictions would result in a major increase in the disease burden of the population in particular in case of mobile phones with their very high prevalence.

In the following paragraphs, case-control, cohort and ecological studies will first be described separately. The last part will summarize the findings of all three designs and an interpretation of the overall evidence is given.

Case-control studies

Interphone was a multinational case-control study conducted in 16 centres in 13 countries; several country-specific results were already available for the previous SCENIHR Opinion (SCENIHR, 2009). The final report of Interphone included 2708 cases of glioma with 2792 matched controls, and 2409 meningioma cases with 2662 matched controls (Interphone Study Group, 2010). A reduced risk related to ever having been a regular mobile phone user (using a mobile phone at least once a week over a period of 6 months or more) was seen for glioma (odds ratio (OR) 0.81; 95% confidence interval (CI) 0.70–0.94) and meningioma (OR 0.79; CI 0.68–0.91). No elevated OR was observed beyond 10 years after first phone use (glioma: OR 0.98; CI 0.76–1.26; meningioma: OR 0.83; 95% CI 0.61–1.14). ORs were below 1.0 for all deciles of lifetime number of phone calls compared with non-regular users and for nine deciles of cumulative call-time, with several ORs in the intermediate categories being statistically significantly decreased. In the top decile of recalled cumulative call time, beyond 1640 hours of use, the OR was 1.40 (CI 1.03–1.89) for glioma, and 1.15 (CI 0.81–1.62) for meningioma. An analysis with the lightest users (less than 5 hours of use) as a reference gave respective ORs of 1.82 from glioma and 1.10 for meningioma. ORs for glioma tended to be higher in the temporal lobe than in other lobes of the brain, but the CIs around the lobe-specific estimates were wide. ORs for glioma were higher in subjects who reported phone use mostly on the same side of the head (ipsilateral) as their tumour than for use on the opposite side (contralateral). For meningioma, ORs for temporal lobe tumours were slightly lower than for other locations, while a similar pattern as for glioma of higher ipsilateral ORs compared to contralateral ORs was seen. Years since first use by cumulative call time showed the highest ORs for heavy use in the shortest-term users of 1-4 years for in both glioma and meningioma.

Several factors may have had an impact on the results: i) evidence of an overestimation of mobile phone users among controls contributed to the overall decrease in risk in overall use; ii) prodromal symptoms of the tumour, particularly glioma, may have added to this effect if due to those symptoms patients refrain from becoming mobile phone users or use it less as they would otherwise; iii) evidence of general difficulties in remembering past mobile phone use accurately, introducing non-differential random error, that would lead to an underestimation of an association, if it exists; iv) evidence of systematic reporting errors with underestimation of use by light users and overestimation of use by heavy users, that could inflate an association; v) some evidence of stronger over-reporting of past use in cases than in controls, and of more commonly reported implausible values in cases that could lead to a spurious positive association. Due to the nature of various biases with some leading to under- and some to overestimation of associations, firm conclusions are difficult to draw.

Two novel approaches were used in subsets of the Interphone data to further explore the relationship between RF and location of the brain tumour (Larjavaara et al., 2011; Cardis et al., 2011b). Larjavaara et al. (2011) used two approaches: In a case-case analysis, tumour locations were compared with varying exposure levels; in a case-specular analysis, a hypothetical reference location was assigned for each glioma, and the distances from the actual and hypothetical locations to the mobile phone were compared. The study included 888 gliomas from 7 European countries. Overall, the results did not suggest that gliomas in mobile phone users are preferentially located in the parts of the brain with the highest RF exposure. Based on small numbers, however, the OR from the case-specular analysis in mobile phone users of 10+ years was 2.0 (CI: 0.68-5.85) and was 1.0 (CI: 0.59-1.69) in the category of highest cumulative call time (>339 hours). Cardis et al. (2011b) used a RF modelling algorithm developed based on mobile phone characteristics such as frequency, type of phone, etc. and location of the brain tumour based on images (Cardis et al. 2011a), and applied it to 553 glioma and 676 meningioma cases with 1762 and 1911 controls, not over-lapping with the study population from Larjavaara et al. RF dose was estimated as total cumulative specific energy (TCSE; J/kg) absorbed at the tumour's estimated centre. The ORs for glioma increased with increasing TCSE 7 or more years before diagnosis, with an OR of 1.91 (CI: 1.05-3.47) in the highest quintile, other ORs varied around 1.0. Patterns for meningioma were similar, but ORs were usually lower, many below 1.0, except for TCSE 7 or more years before diagnosis where the OR was 2.01 (CI: 1.03-3.93). A complementary analysis in which 44 glioma and 135 meningioma cases in the most exposed area of the brain were compared with gliomas and meningiomas located elsewhere in the brain showed increased ORs mainly for glioma in the most exposed part of the brain in those with 10+ years of mobile phone use (OR 2.80, CI 1.13 to 6.94 for glioma; OR 1.34, CI 0.55-3.25 for meningioma) but not in those in the highest category of cumulative mobile phone use, 1147+ hours (OR 0.99, CI: 0.30-3.27 for glioma; OR 1.41, CI 0.66-3.02 for meningioma). Comparing the two sets of results with the original Interphone results shows consistency; while the approach by Larjavaara et al. (2010) is rather conservative and attempts to remove sources of recall bias, it strengthens the overall finding of no association, whereas the approach by Cardis et al. (2011a) offers a refinement of the exposure metric emphasizing the association in heavy users; however, it is still based on recall and cannot therefore exclude that the observed association might be due to bias.

Another case-control study in several parts was done in Sweden. A pooled analysis covered two case-control studies on patients with malignant brain tumours diagnosed during 1997-2003 and matched controls alive at the time of study inclusion, as well as one case-control study on patients and controls deceased during the same time period (Hardell et al., 2011). The analysis included 1,251 cases and 2,438 controls. ORs increased with latency being 1.1 (CI 0.9-1.4) for 1-5 years, 1.2 (CI 0.9-1.5) for >5-10 years and 2.5 (CI 1.8-3.3) for 10+ years of mobile phone use. For cordless phone use the respective figures are 1.1 (CI 0.9-1.4), 1.4 (1.1-1.8) and 1.6 (CI 1.03-2.5). Risks were highest when use started before the age of 20 years, especially for astrocytoma. Risks increased by 1-2% per 100 hours of cordless phone or mobile phone use. No

validation studies to assess the possible impact of bias and errors were carried out for this study, but most of those identified in Interphone would likely apply to this study, too. While response rates for the Hardell studies were reported to be higher than for Interphone, the mixture of self-administered questionnaire and telephone interviews not described in detail allowed less standardized guidance through complicated questions.

In a commentary, Hardell et al. (2011) made an attempt to allow better comparison between the results of the Interphone study and the Swedish case-control studies, by restriction to the same age group of 30-59 years and applying the Interphone definition of a non-regular mobile phone user (regular user was defined as at least one call per week over a period of six months or more and disregarding cordless phone use) and the cut-offs of different user categories to their data. The ORs in the two studies became more similar for the heavy users (as defined by Interphone, 1640+ hours of lifetime cumulative use), being 1.75 (1.02-3.00) for the Swedish studies compared to 1.40 (1.03-1.89) for Interphone, but for most other comparisons remained different (e.g. for time since first use of >10 years: 1.79 (1.19-2.70) vs 0.98 (0.76-1.26; Interphone). Afterwards, in an attempt to quantify the relationship, Interphone and the Hardell studies were analysed in a meta-analytical approach (Hardell et al., 2013a), an OR of 1.71 (CI: 1.04-2.81) was found for temporal glioma among ipsilateral mobile phone users of 10+ years of use; however, the above-mentioned caveats of combining those studies remain.

Hardell and Carlberg (2013) analysed the survival of patients after glioma diagnosis in relation to the use of wireless phones. All cases diagnosed between 1997 and 2003 with a malignant brain tumour (n = 1,251) in the authors case-control studies were included. For glioma, the use of wireless phones (mobile and cordless phones) gave a hazard ratio (HR) = 1.1 (95% CI =0.9–1.2), with >10-year latency HR = 1.2 (95% CI = 1.002–1.5, p trend = 0.02). For astrocytoma grade IV (glioblastoma), HR was 1.1 (95% CI = 0.95–1.4), with >10 year latency HR = 1.3 (95% CI = 1.03–1.7). In the highest tertile (>426 h) of cumulative use, HR = 1.2 (95% CI = 0.95–1.5) was found for glioblastoma. A decreased survival of glioma cases with long-term and high cumulative use of wireless phones (mobile and DECT) was found.

Three further case-control studies were published recently. Hardell et al. (2013b) extended their case-control study series with glioma cases aged 18-75 years and diagnosed between 2007-2009, with overall 593 participating cases and 1368 controls, using the methodology of their previous studies. ORs for use of different types of wireless phones were increased, being 1.8 (CI: 1.04-3.3) for analogue phones, 1.6 (CI: 0.996-2.7) for digital (2G) phones, and 1.7 (CI: 1.1-2.9) for cordless phones; all associations were stronger when latency periods between 15-25 years were taken into account. For analogue phones and >25 years of latency, the OR was 3.3 (CI: 1.6-6.9), and for all digital (2G, 3G, cordless) phones combined and >20 years latency it was 1.5 (CI: 0.5-4.6). They also extended their set of meningioma cases, also by those aged 18-75 years and diagnosed between 2007-2009 (Carlberg et al., 2013), involving 709 cases and the same 1368 combined controls of the glioma and meningioma study as used for the glioma analyses. ORs for use of different types of wireless phones were not increased, being 0.9 (CI: 0.6-1.5) for analogue phones, 1.0 (CI: 0.7-1.4) for digital (2G) phones, and 1.1 (CI: 0.8-1.5) for cordless phones. ORs varied around 1.0 for different phone types also when taking long latencies into account. With >25 years latency, the OR for all wireless phones combined (analogue and all digital) was 1.2 (0.6-2.4), based on 16 exposed cases and 33 exposed controls. In the highest category of use (≥2376 hours), for all phone types combined, the OR was 1.4 (CI: 0.9-2.0). Coureau et al. (2013) published results from a French multi-centre case-control study of cases of glioma and meningioma diagnosed between 2004-2006, with a total of 253 gliomas, 194 meningiomas and 892 controls, following a questionnaire-based approach for exposure assessment. No association was seen when comparing ever regular users with non-users (OR 1.24, CI: 0.86-1.77 for glioma; OR 0.90, CI: 0.61-1.34 for meningioma). When specifically looking at the highest category of lifetime cumulative use (≥896 hours), however, ORs increased, and were 2.89 (CI: 1.41-5.93) for glioma and 2.57 (CI: 1.02-6.44) for meningioma.

The results on glioma from the most recent Swedish study are in line with the previous results from the same group, namely showing increased risks with ever use of mobile phones for all wireless phone types, that even increase further with longer latencies. The French study finds an increased risk only in "heavy" users and is therefore often interpreted as being compatible with Interphone (Interphone, 2010). However, it needs to be noted that the strengths of associations are very different and so is the definition of a "heavy" user, with a much lower cut-off in the French study. For glioma, the French study (Coureau et al., 2013) shows an OR of 2.89 for ≥896 hours, while the comparable categories in Interphone show ORs of 0.71 (CI: 0.53-0.96) for 735-1640 hours and 1.40 (CI: 1.03-1.89) for ≥1640 hours.

The only available study on mobile phone use and brain tumours in children and adolescents is the Cefalo study conducted in four European countries, involving face-to-face interviews with 352 families of brain tumour patients in 7-19 year olds and 646 matched controls (Aydin et al., 2011a). Regular use (again at least one call per week over a period of 6 months or more) showed a statistically non-significantly increased OR of 1.36 (CI 0.92-2.02), but there was no trend by either time since first use, cumulative number of calls, or cumulative call time. Use of cordless phones showed no increased OR (1.09; CI 0.81-1.45), not even in the group of highest cumulative use. For a subsample of participants it was possible to obtain traffic records from mobile phone operators: while the OR significantly increased in the time since first use category of longest latency of >2.8 years (2.15; CI 1.07 to 4.29), there was no trend by cumulative call time with ORs being 1.24, 1.95 and 1.38 (none statistically significantly elevated). No clear patterns were seen when comparing ipsilateral and contralateral use. Validation studies in the context of Cefalo confirm observations from Interphone, namely the difficulty of participants to accurately recall past mobile phone use (Aydin et al., 2011b).

Cohort studies

Follow-up of all private Danish subscribers of mobile phones starting in 1982-1995 for brain tumour risk until 2006 was included in the previous Opinion (SCENIHR, 2009). In the meantime, an update of this cohort was published (Frei et al., 2011). In this update, 358,403 subscription holders accrued about 3.8 million person-years. Relative risks (RR) for all central nervous system tumours was 1.02 (CI 0.94-1.10) in men and 1.02 (CI 0.86-1.22) in women, based on 714 cases in men and 132 in women. By type of brain tumours, no associations were seen for glioma (1.08 in men and 0.98 in women) or for meningioma (0.78 in men and 1.02 in women). In the longest term subscribers, of 13+ years, RR of glioma for men was 0.98 (CI 0.70-1.36), based on 37 cases. Analysis by lobe showed no clear pattern, the RR for temporal lobe glioma in men being 1.13 (CI 0.86-1.48); due to small numbers no subanalyses were possible for women. Exposure misclassification is of concern in this cohort study, as information was only available on subscriptions in the name of an individual (no subscriptions that were in the name of a company) and no data were obtained on the amount of use; cordless phone use was not included. An advantage, however, is that subscriber status was ascertained before occurrence of disease. . No analysis by amount of use was possible. Therefore, heavy users could not be analysed separately. This could lead to an underestimation of the association if risk was restricted to heavy use, depending however on the proportion of heavy users within the overall user category.

Recently, the results of the Million Women Study conducted in the UK pertaining to mobile phone use were released (Benson et al. 2013), with prospective data on years of mobile phone use and never, less than daily, or daily use for approximately 800,000 middle-aged women. The mobile phone use was assessed by questionnaire and did not include the use of cordless phones. No indications of increased risks of glioma were found in relation to duration or frequency of mobile phone use (rate ratios for the highest exposed groups of 10+ years of mobile phone use or daily use, respectively, based on 40 cases, group 0.8-0.9 with upper confidence limits around 1.1). Rate ratios were close to one also for meningioma for all exposure indices. In summary, the relative risks for 10+ years of mobile phone use were 0.78 (CI 0.55-1.10) for glioma and 1.10 (CI 0.66-1.84)

for meningioma. The follow-up was relatively short, on average seven years, and numbers of cases for specific tumour types rather small, especially for long/term users.

Time trend analyses

Analyses of recent time trends of brain tumours and its subtypes were published based on incidence data from the UK (de Vocht et al., 2010), from the Nordic countries (Deltour et al., 2012), and from the US (Little et al., 2012). They consistently show little indication of an increase in the age groups of most active mobile phone users and steady weak increases only in the elderly. Such analyses of incidence trends provide evidence which is too weak to rule out an association between mobile phone use and brain tumour risk but may be suitable to check the plausibility of reports on higher risk. In two of these studies (Deltour et al., 2012, and Little et al., 2012), simulations were used to evaluate the risk estimates of the case-control studies by calculating expected time trends under various risk scenarios and comparing them with the observed time trends. The simulation study in the Nordic countries virtually rules out a doubling in risk even after 15+ years since first mobile phone use as well as a 50% risk increase after 10+ years and 20% after 5+ years; increases of 50% after 15+ years or 20% after 10+ years would be highly unlikely as well as 10% after 5+ years (Deltour et al., 2012). When assuming risk only among heavy users, the possibilities of detecting such effects decrease. However, a doubling of risk with 10+ years latency or 50% with 5+ years latency are very unlikely, given the observed trends. The overall decreased risk in Interphone, an OR of 0.8 for overall use would also be highly unlikely, even assuming 10+ years latency (Deltour et al., 2012). The US results confirm the observations made on the basis of the Nordic countries (Little et al., 2012). No increase was seen in the UK study (De Vocht et al., 2011), with the exception of a small one in temporal tumours; how much this is attributable to a decline in tumours with missing information on location is unclear. With respect to teenagers and adolescents, Aydin et al. (2012) provide incidence rates from Sweden in their Cefalo report to compare with the ORs observed in the study; rates were stable or even slightly declining, providing little support for a 36% risk increase with overall mobile phone use as seen in the case-control analysis and some evidence against the two-fold risk increase after 2.8+ years latency as observed in the operator-records based case-control analysis.

The relationship between risks observed in analytical studies and the associated absolute excess in the incidence is shown in Figure 5 for one specific scenario. The figure shows the observed glioma incidence rate in the Nordic countries, as reported by Deltour et al. (2012), reflecting how many cases occurred in the Nordic countries. In addition, three predicted incidence rates are shown which are based on an increased risk of 1.2, 1.5 or 2 respectively, after regular mobile phone use of 10 years or more. The predicted steep increase shows that increased risks of these magnitudes are in conflict with the population data. That renders all studies reporting increased risks of such magnitude implausible. Consequently, the most plausible reason for the reported increased risks are methodological artefacts.

Figure 5. Observed glioma incidence rate in the Nordic countries and expected rates assuming mobile phone (regular mobile phone use of 10 years or more) related relative risk increases of 1.2, 1.5 and 2, respectively [Based on data from Deltour et al. 2012]

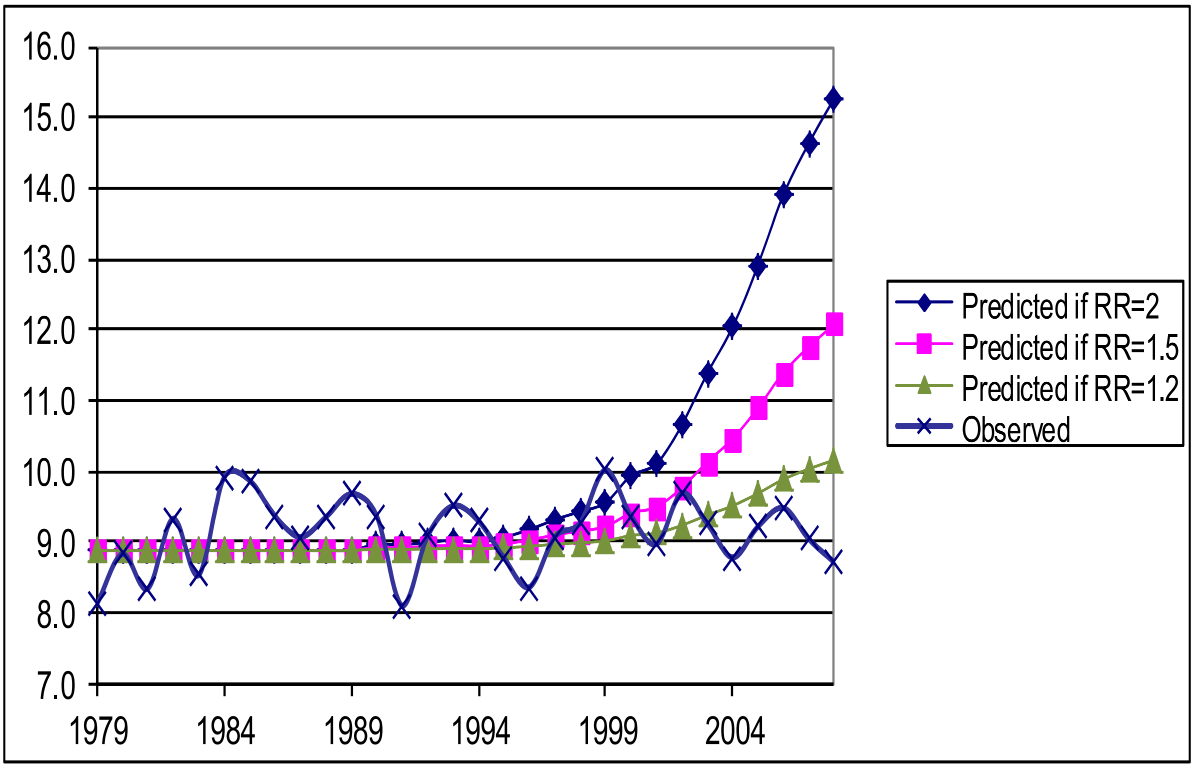

Brain tumour results in context

The fact that incidence rates of glioma and meningioma do not rise in the age groups of highest mobile phone prevalence provides evidence that common use of mobile phones is unlikely to be associated with an increased risk of those brain tumours. This is confirmed by the Danish cohort study that rules out risks that would affect large segments of the population. Evidence against an association also arises from the large-scale UK million women study with prospective exposure information. Case-control studies already show associations for moderate mobile phone use, with decreased risk estimates in Interphone and increased risk estimates in the Hardell studies, both incompatible with the observed incidence rate time trends and demonstrating the vulnerability of case-control studies with self-reported mobile phone use to bias. With such a material impact already in the overall results, the findings restricted to heavy mobile phone users become difficult to interpret. Increase of risk in heavy users, such as 1.5-fold incidence after 10 years of use, are incompatible with observed incidence trends. The two major studies differ in some methodological aspects including different comparison groups (different definition of the unexposed reference). However, while this may explain some of the heterogeneity, the fundamental difference in risk observed remains in the moderate users that also influences the association seen in heavy users. The incidence time trends do not contradict a modest increase in heavy users because numbers of excess cases would remain too small to be detectable in the time period analysed.

<u>Acoustic neurinoma</u>

Acoustic neurinoma, also termed vestibular schwannoma, is a tumour that arises on the eighth cranial nerve leading from the inner ear to the brainstem and accounts for about 5% of all intracranial tumours. The Interphone study also included 1105 patients with newly diagnosed acoustic neuroma and 2145 controls (Interphone Study Group, 2011). OR with ever having been a regular mobile phone user was 0.85 (CI 0.69–1.04). The OR

for 10+ years after first regular mobile phone use was 0.76 (CI 0.52–1.11). There was no trend of increasing ORs with increasing cumulative call time or cumulative number of calls, with the lowest OR (0.48; CI 0.30–0.78) observed in the 9th decile of cumulative call time. In the 10th decile (1640+ hours of cumulative call time), the OR was 1.32 (CI 0.88–1.97). With censoring at 5 years before the reference date the OR for 10+ years after first regular mobile phone use was 0.83 (CI 0.58–1.19) and for 1640+ hours of cumulative call time it was 2.79 (CI 1.51–5.16), but again with no trend in the lower nine deciles and with the lowest OR in the 9th decile. In general, ORs were not greater in subjects with ipsilateral use.

Acoustic neuroma was also analysed in the Danish subscriber cohort, and follow up through 2006 identified 404 cases in men and 402 cases in women among approximately 2.8 million Danes (Schüz et al., 2011). Among subscribers of 11+ years since the first subscription, 15 cases occurred in men yielding an RR of 0.87 (CI 0.52-1.46) and no case versus 1.6 expected in women. Additional clinical data showed that acoustic neuroma sizes in long-term mobile phone subscribers were not larger than among nonsubscribers and tended not to be more often on the right side of the brain, the side of the head preferred during mobile phone use by the majority of the Danish population.

The two studies align well in providing additional evidence against a positive association between common mobile phone use and risk of acoustic neuroma. In the case-control study an increased risk in the group of heaviest users was observed; patterns, however, were difficult to interpret as in the second highest group of heavy use the risk was statistically significantly decreased. As the group of heavy users comprised only 77 of the 1105 cases (about 7%), the absolute number of excess cases would be small in populations and therefore difficult to detect in the cohort study or in incidence time trend analyses, as done by Larjavaara et al. (2011) where no increase was observed in time trends compatible with a mobile phone-related hypothesis. In the UK Million Women study (Benson et al., 2013), an excess of acoustic neuroma was seen among those using mobile phones the longest. Increased risks were noted for more than 10 years of use (relative risk of 2.46 (CI 1.07-5.64)), though the finding was based on less than 10 cases. In the same paper, an analysis of incidence rates of acoustic neuroma in England showed no increasing trend in the period 1998-2008. An update was recently published in a letter (Benson et al., 2013b); no increase in risk in acoustic neuroma was observed anymore (the relative risk for long-term users of 10+ years decreased from 2.46 to 1.17 (CI: 0.60-2.27)), suggesting that the previously reported increase in risk was a chance finding.

Two further case-control studies were recently reported from Sweden. In a study of 451 cases and 710 controls, the OR for ever using a mobile phone was reported to be 1.18 (CI: 0.88-1.59) (Pettersson et al., 2014). The OR for the highest quartile of cumulative mobile phone use (≥680 hours) was initially 1.46 (CI: 0.98-2.17) and 1.14 (CI: 0.63-2.07) when only histologically confirmed cases were included. Hardell et al. (2013c) added a further case-control dataset of cases diagnosed between 2007-2009 to their previous case-control dataset of acoustic neuroma cases, and the pooled analyses based on overall 316 cases and 3530 controls. This showed increased risks for different wireless phone types, but strongest for analogue phones. For use of analogue phones, ORs were 2.9 (CI: 2.0-4.3) and 7.7 (CI: 2.8-21) in those with >20 years latency. The respective ORs for all digital wireless phones combined (2G, 3G, cordless) were 1.5 (CI: 1.1-2.0) and 8.1 (CI: 2.0-32) with >20 years latency. For total wireless use, in the >20 years latency group, the OR was 4.4 (CI: 2.2-9.0).

<u>Other tumours of the head and neck region</u>

Salivary gland tumours represent about 3% of the head and neck tumours, and among them, parotid gland tumours occur in about 70-80%. No additional analytical studies on mobile phone use and the risk of parotid or specifically salivary gland tumours were published since the last Opinion (SCENIHR, 2009). In addition, researchers investigated time trends in incidence rates or numbers of cases. In Israel, the total number of parotid gland cancers in Israel increased 4-fold from 1970 to 2006 (from 16 to 64 cases per

year). The steepest increase occurred after 2001; however, no incidence rates were presented (Czerninsky et al., 2011). In the UK, numbers of new cases of parotid gland tumours more than doubled from 112 new cases in 1986 to 247 in 2007 in men, and 116 to 199 cases in women (de Vocht, 2011), corresponding to an increase in age-standardized incidence rates from 0.5 to 0.8 (1986-2008) per 100,000 in men and 0.4 to 0.6 in women. More recently, data of around 8500 patients in the Nordic countries were analysed (Shu et al., 2012). The age-standardized incidence rate of salivary gland tumours between 1970 and 2009 was stable, with annual percent changes of -0.1% (CI: -0.4 to 0.2) for men and -0.2% (CI: -0.5%-0.1%) for women, providing no evidence of any increase.

Söderqvist et al. (2012a) studied the risk of salivary gland tumours and use of wireless phones. Sixty-nine patients with salivary gland tumours and 262 randomly recruited controls were included. The use of wireless phones was not associated with an overall increased risk of salivary gland tumours, odds ratio 0.8, 95% confidence interval 0.4-1.5.

A UK population-based case-control study of the risk of pituitary tumours in relation to mobile phone use enrolled 291 cases and 630 controls (Schoemaker and Swerdlow, 2009). Following the Interphone design and interview, tumour risk was not associated with overall mobile phone use, and did not increase 10+ years after first use (OR 1.0; CI: 0.5-1.9), or for users in the highest quartile of cumulative number call time (OR 1.1; CI 0.7-1.7). A hospital based case-control study of mobile phone use and parotid gland malignancies carried out in China (Duan et al. 2011) was based on 136 epithelial cancers and 64 mucoepidermoid carcinomas with 2051 hospital controls. However, the results are not internally consistent showing both increases and decreases across the exposure range.

<u>Melanoma (skin, eye) and other skin cancer</u>

A German hospital-based case-control study of uveal melanoma of the eye found no increased risk related to mobile phone use (Stang et al. 2009). The material consisted of 459 cases (participation 94%) and 827 population-based controls (with additional sets of hospital and sibling controls). Regular use, long duration of use or cumulative call time did not show any increased risks (point estimates below unity, with most upper confidence bounds below 1.5). These findings contradict those of an earlier report (Stang et al. 2001) by the same group (related only to mobile phone use at work), but the current study is based on a larger material and more extensive exposure assessment.

In a Swedish case-control study the use of mobile phones and cordless phones was assessed for 347 cases with malignant melanoma in the head and neck region and for 1,184 controls (Hardell et al., 2011). Overall no increased risk was found. In the most exposed area, namely temporal, cheek and ear, cumulative call time of >365 hours of cordless phone use showed an OR of 2.1 (CI: 1.1-3.8) and mobile phone use of 2.1 (CI: 0.7-6.1) in the group of 1-5 years after first use, but no association was seen for longer latencies.

Using the Danish subscriber cohort study described above, no increased risks were seen for malignant melanoma, squamous cell carcinoma or basal cell carcinoma of the head (Poulsen et al., 2012). Among men with ≥13 years of subscription, the RRs were close to unity for basal and squamous cell carcinomas of the head. For melanoma, although a slightly elevated RR was found (RR=1.20, CI: 0.65-2.22), a similar RR was observed for melanoma of torso or legs (RR=1.16, CI: 0.94-1.47), yielding a ratio of the two RRs of 1.04 (CI: 0.54-2.00). The risk pattern was similar among women, although it was based on smaller numbers.

Discussion of brain tumours and other tumours of the head and neck area

Overall, there is little evidence that moderate mobile phone use is associated with risk of any cancer in the head and neck region. This is supported by large-scale epidemiological studies of three different designs. Only one case-control study shows risk increases at

moderate usage levels, but the results are incompatible with observed time trends in incidence rates in reality checks and can therefore not be used for hazard assessment.

Evidence is more controversial for heavy users of mobile phones; "heavy use" is a qualitative characterisation and difficult to quantify as the users with the highest life-long use are compared to those with lesser use (combining years of use and amount of daily use), with various definitions and cut-points. For instance, in Interphone, "heavy users" were approximately 10% of life-long heaviest regular users (or about 5% of all study subjects). It corresponds to, for example, half an hour of daily use over 10 years or more (in the communication of the outcome of the IARC Monograph (IARC 2013)), but this figure must not be interpreted as any suggestion of a threshold of an effect. For the heaviest users, the largest case-control study in particular observed about 40% increased risks for glioma and for acoustic neuroma. It cannot be concluded from the available studies whether this reflects a causal association. Limitations of the case-control studies, including selection bias and recall bias, raise concern that the observed association in small subgroups could be attributable to methodological shortcomings. Time trend analysis in incidence rates and the two cohort studies show no evidence of any risk. They are useful to check the plausibility of elevated risk estimates but would not detect small risk increases after longer latencies in heavy users only.

A major limitation of most studies is that mobile phone use is used as a crude proxy for RF exposure, with the latter also depending on many technological features, but very strongly – as described in the chapter on exposure – on the generation of mobile technology. RF exposure from NMT handsets were manifold higher than GSM technology or today's exposure and RF exposure during the roll out of GSM technology, when networks were not fully optimized, was also substantially higher than current exposure levels. Therefore, the increased risk estimates seen in heavy users in case-control studies, mainly driven by former technologies may not be replicable anymore due to changed technology.

For meningioma, the evidence for increased risks of long-term heavy users is weaker than for glioma, but some case-control studies do show increased risks as well. For uveal melanoma, there is no evidence for any association, including heavy users. For salivary gland tumours and melanoma of the cheek or ear the evidence is somewhat controversial as for glioma but based on much fewer studies.

Too few of the published cancer studies have sufficient statistical power and observation time to identify a potential small risk after use periods of 15 years or more. Most reported elevated risks did not pass the plausibility check by time-trend analyses. Although, overall, the evidence of any positive association is weak, given the widespread use of mobile phones, more research with improved quality of exposure assessment is needed. There is currently only one recent study, overcoming the limitations mentioned above by oversampling light and heavy users from the population and basing exposure assessment on traffic records from network operators (Schüz et al., 2011).

Cancer other than head and neck region

What was already known on this subject?

The previous SCENIHR Report concluded that evidence weighed against an association between RF-EMF exposure from broadcast transmitters and the risk of childhood leukaemia.

What has been achieved since then?

<u>Childhood cancers in relation to RF exposure</u>

A nation-wide case-control study of RF EMF exposure from base stations and childhood cancers was conducted in the UK (Elliott et al. 2010). It covered all childhood malignancies diagnosed at ages 0-4 years during 1999-2001, with four controls per case identified from national birth register, with matching on sex and date of birth. The electromagnetic field from base stations was estimated based on coordinates of residence

at birth (obtained for 93% of the cases and 90% of the controls) and comprehensive data on all base stations by the four nationwide network operators. For central nervous system cancers (251 cases), no increased risks were found for the highest exposure tertile in terms of distance from the nearest base station, its power output or calculated power density (adjusted odds ratios 0.76-0.95, with upper confidence limits 1.12-1.38). No indication of increased risks was found for leukaemia and lymphoma either (odds ratios 1.03-1.08, with upper confidence limits 1.34-1.42, 527 cases). Analyses of continuous exposure metrics did not reveal any indication of exposure-response effects.

Hauri et al. investigated the association between exposure to RF EMF from broadcast transmitters – with field strengths in V/m estimated from prediction models – and the risk of childhood cancer in children under the age of 16 years at diagnosis in Switzerland (Hauri et al., 2014). They used two approaches: one using time-to-event analysis of children included in the Swiss National Cohort in the 2000 census followed up for cancer in the time period 2000-2008, and one approach using incidence density cohort analysis by using all cases registered in the Swiss Childhood Cancer Registry 1985-2008 and person-years under risk from censuses (1990, 2000) and interpolation between or extrapolation before or after the respective censuses. Hazard ratios (HR) from the time-to-event analysis based on overall 830 cancers (40 in the high exposure group >0.2 V/m) did not show an association (HR=1.02, CI: 0.96-1.08) per 0.1 V/m increase in exposure or in the >0.2 V/m exposure category (HR=1.03, CI: 0.74-1.43), but some variation by cancer type, with a small decrease in HR for leukaemia (0.82, CI: 0.67-1.01) and a small increase in HR for CNS tumours (1.05, CI: 1.00-1.10). In comparison, the incidence density analysis, based on larger numbers (3591 cases overall and 144 in the high exposure category), showed RRs of 0.90 (CI: 0.76-1.06) for all cancers combined, 0.76 (CI: 0.55-1.05) for leukaemia, and 1.03 (CI: 0.73-1.43) for brain tumours for >0.2 V/m compared to <0.05 V/m, for the entire time period. When splitting the time period into 1985-1995 and 1996-2008, with the former one possibly having less “contamination” from RF EMF from the mobile telephony networks, RRs for leukaemia were 1.13 (CI: 0.74-1.71) and 0.52 (CI: 0.32-0.85), and for CNS tumours 1.60 (CI: 0.98-2.61) and 0.75 (CI: 0.45-1.23), respectively. Hence, no clear patterns were identified, with mainly seeing no associations, but in few subgroup analyses both elevated risks (CNS tumours) and reduced risks (leukaemia), possibly due to chance.

A large case-control study of childhood cancer and environmental RF from base stations in Taiwan reported odds ratios slightly and non-significantly above unity for brain tumours, but not leukaemias (Li et al. 2012). The main shortcoming of the study was crude exposure assessment, as information was available on annual power of base stations but residential data related only to the township of residence at the time of diagnosis and no information on address, residential history or other sources of RF was available. No validation study of the exposure indices used was conducted.

<u>Adult cancers in relation to RF exposure</u>

In the nationwide Danish cohort study of mobile phone subscribers described above (Frei et al., 2011), a deficit of all cancers was observed among subscribers combined in men but not women, corresponding to RRs of 0.96 (CI: 0.95-0.98) and of 1.02 (CI: 0.97-1.06) respectively. The reduced risk for men was mainly seen in tobacco-related cancers, suggesting lower tobacco consumption in the group of early mobile phone subscribers compared to the general population.

The above-mentioned prospective UK Million Women Study (Benson et al., 2013) also shows a slight deficit in cancers in mobile phone users, with a RR of 0.97 (CI: 0.95-0.99), again mainly due to fewer tobacco-related cancers.

Leukaemia was suggested to be of interest because it may have a shorter induction period than solid cancers. In a UK case-control study, the relation of acute lymphocytic and non-lymphocytic leukaemia risk to mobile phone use was investigated, including 806 cases and 585 non-blood relatives as controls (Cooke et al., 2009). No association was found between regular mobile phone use (Interphone definition) and risk of leukaemia

(OR=1.06, CI: 0.76-1.46). Analyses of risk in relation to years since first use or cumulative call time showed no significantly raised risks, and there was no evidence of any trends. A non-significantly raised risk was found in people who first used a phone 15 or more years ago (OR=1.87, CI: 0.96-3.63). Another study from Thailand with 180 cases and 756 age- and sex-matched hospital controls covered only short durations of mobile phone use (median 24-26 months), rendering an observed association with digital mobile phone use difficult to interpret (Kaufman et al., 2009).

Conclusions on epidemiology of neoplastic diseases

Overall, the epidemiological studies on RF EMF exposure do not indicate an increased risk of brain tumours, and do not indicate an increased risk for other cancers of the head and neck region, or other malignant diseases including childhood cancer.

Two large prospective cohort studies do not show increased risks of brain tumours or other malignancies and large-scale time series analyses of incidence trends are consistent with their results. Some case-control studies have reported odds ratios around 1.5 to 3 for the highest exposed groups of cumulative use time, but recall bias cannot be excluded as a possible explanation. Case-case analyses of the highest exposed parts of the brain have not shown increased risk when exposure indices independent of self-reported use have been employed. The only study of mobile phone use and brain tumours in children did not show an increased risk, but more studies are needed especially for those starting to use mobile phones as children and their cancer risk later in life.

The totality of evidence of epidemiological studies weighs against cancer risks from base stations and broadcast antennas. In particular, large case-control studies modelling RF exposure and investigating the risks of childhood cancers have not shown any association.

A working group at the International Agency for Research on Cancer (IARC) within the Monograph programme on the evaluation of carcinogenic risks to humans classified the epidemiological evidence for glioma and acoustic neuroma as limited and therefore evaluated RF fields as a possible human carcinogen (IARC, 2013). Based on studies published since that assessment (update of the Danish cohort study, the UK cohort study, further case-control studies, the case-control study on mobile phones and brain tumours in children and adolescents, the consistency checks of brain tumour incidence rates using data from the Nordic countries and the US), the evidence for glioma has become weaker.

3.6.1.2. *In vivo* studies

What was known on this subject?

A number of studies have investigated the possible carcinogenicity of RF fields using animal models. These have used both normal strains and those with a genetic predisposition to one or more types of cancer. Other studies have tested possible co-carcinogenicity with known chemical or physical carcinogens. While a few of these studies have reported positive results (most notably, Repacholi et al. (1997) found an increased lymphoma incidence in the transgenic *Eμ-Pim1* mouse model) the majority of studies have produced no evidence that exposure to mobile phone signals is associated with an increased incidence, latency or severity of neoplasms, nor does exposure have a significant effect on survival time or increase the occurrence of other adverse responses. The previous Opinion concluded that the newer studies were consistent with earlier results, and the few differences that had been observed for some endpoints were possibly false positives. Overall, it was concluded that RF fields such as those emitted by mobile phones were not carcinogenic in laboratory rodents.

What has been achieved since then?

Bartsch et al. (2010) examined the effects of near-continuous, long-term exposure to low intensity GSM signals on health and survival in female SD rats. Groups of 12 freely

moving animals were exposed in their home cages to 900 MHz GSM signals at average whole-body SARs of 0.08 W/kg (when young) to 0.038 W/kg (when old). Weight was monitored at regular intervals and an extensive post-mortem examination was carried out on most animals. No significant changes in weight gain or on the incidence of mammary or pituitary tumours were seen in two groups of 12 animals exposed for up to 24 months. No significant effects on weight gain were seen in two groups of 30 animals given exposure until death (at about 36 months of age), but their lifespan was significantly shortened. The incidence of mammary tumours was also reduced, possibly due to a relative increase in pituitary tumours in these animals. It was suggested that previous rodent studies had not used a sufficiently long exposure period to enable the effects of the RF field to be seen. Significant differences in survival were also noted between groups (including the sham-exposed animals) that were attributed to differences in the time of year the animals were born: those animals born in the spring had a significantly longer survival compared with those born in the autumn.

Jin et al. (2011) exposed young rats to combined 849 MHz CDMA and 1950 MHz WCDMA signals at a combined SAR of 4 W/kg, for 45 min/day, 5 day/week for a year. Animals were exposed alternately in the morning or afternoon. No significant effects on weight or on spontaneous tumour rates were found, and post-mortem analysis did not show any significant pathological differences that could be related to exposure. In addition, analysis of blood and urine did not reveal any significant field-related effects except a significant increase in mean corpuscular haemoglobin level, and alkaline phosphatase in males; and a significant decrease in total bilirubin, and lactate dehydrogenase in females.

Lee et al. (2011) exposed young AKJ/R mice (which spontaneously develop lymphoma) to combined CDMA and WCDMA signals for 45 min/day, 5 day/week for 42 weeks using a reverberation chamber; the SAR at each frequency was calculated to be 2 W/kg. Compared to sham-exposed controls, exposure had no significant effect on weight, survival time or incidence of lymphoma. The latter was assessed by histopathological analysis of the thymus. Blood counts remained unaffected by exposure and there were no consistent effects on metastatic infiltration in the spleen or other organs (changes in infiltration were seen in the brain but these was attributed to factors other than exposure).

Some studies have investigated the effects of long-term exposure to RF fields on the promotion of CNS tumours in rats initiated by prenatal (maternal) administration of n-ethylnitrosourea (ENU) and have generally found negative results (SCENHIHR, 2007). However, Tillmann et al. (2010) found that life-time exposure to 1.966 GHz UMTS signals (for 20 h/day, beginning on gestational day 6 and continuing for up to 24 months) increased incidence and multiplicity of lung carcinomas in female mice compared with animals treated with ENU alone. Peak SARs were calculated to be 5 W/kg and a pre-study showed that this exposure did not induce measurable increases in body temperature. Significant effects were also seen on liver tumours, but these were discounted due to possible confounding caused by bacterial infection. UMTS exposure on its own had no tumourigenic effect. Due to limitations in the design of the study, the authors considered this a pilot, so more extensive studies using this model would be informative.

Finally, the results of a National Toxicology Program (NTP) project entitled "Studies to Evaluate the Toxic and Carcinogenic Potential of Cell Phone Radio Frequency Radiation in Laboratory Animals" are expected to be published in late 2014 (http://ntp.niehs.nih.gov). This large and important project was initiated in 2003 at the Illinois Institute of Technology Research Institute. It uses well-characterised reverberation chambers to expose animals to intermittent fields (10 min-on, 10 min-off) for 18.50 hours per day, 5 days per week, without the need for restraint. Following studies exploring thermal effects, and a pre-chronic study investigating effects on *in utero* and post-weaning exposures, a chronic toxicity/carcinogenicity study will be undertaken. It is planned to expose rats and mice for two years to GSM or CDMA signals at 900 and 1900 MHz at three SARs, the highest of which is expected to induce an increase in body temperature

of 1°C. Long-term absorption of RF energy at that level will have a considerable impact on thermoregulation, and induce compensatory changes in metabolism, as well as reducing food consumption and spontaneous activity. Nevertheless, the results of the project are eagerly awaited and will inform future research in this area.

Repacholi et al. (2012) conducted a systematic review of animal laboratory studies that investigated the risks of exposure to RF fields associated with mobile phones on brain cancers or other tumours of the head. Twelve animal studies were identified that have been published since 2000. No statistically significant relationship was found between exposure to RF fields and genotoxic damage to the brain or the incidence of brain cancers or other neoplasms of the head. However, a significant increase in spontaneous pituitary tumours was found in female rats and mice at SARs below 2 W/kg (OR 1.6, 95% CI 1.2-2.2). This excess was not found in male rats and mice exposed below 2 W/kg, and exposure at higher SARs did not result in a significant change from unity in either males or females. The authors attributed the excess to under-representation of tumours in the sham-exposed groups in two out of the three studies considered, resulting in a spurious increase in overall tumour incidence.

Discussion on *in vivo* studies

Consistent with many earlier studies, recent animal studies have not produced any compelling evidence that RF fields are carcinogenic or have other adverse effects. The recent data are not completely negative, however: one study found that long-term low level exposure of rats to GSM signals may shorten their life-span; and a pilot study using UMTS signals indicated an increased risk of lung tumours in female mice treated with a chemical carcinogen during gestation and after weaning. Neither study is definitive and the results require independent confirmation. The results of a large NTP study are expected in the next year or so, which should help to clarify the remaining uncertainties.

Based upon an analysis of animal studies published since the early 1980s, IARC (2013) considered that the evidence in experimental animals for carcinogenicity of RF fields was limited (for making a definitive evaluation): although some positive studies were noted, there were unresolved questions regarding the adequacy of the design, conduct or interpretation of these studies.

Conclusions on *in vivo* studies

Overall, because a considerable number of well-performed studies using a wide variety of animal models have been mostly negative in outcome, the animal studies are considered to provide strong evidence for the absence of an effect.

3.6.1.3. *In vitro* studies

What was already known?

In the previous Opinion several *in vitro* studies were reviewed. Due to the inconsistent findings and a lack of a dose-response relationship, it was concluded that there was no evidence to explain carcinogenesis of RF fields.

What has been achieved since then?

A large number of studies have been carried out on different cell types. They deal with genotoxic as well as non-genotoxic cancer-relevant endpoints, as reported below.

Genotoxic effects

The induction of genotoxicity after RF exposure has been evaluated by applying several cytogenetic tests that measure chromosomal damage (chromosomal aberrations, micronuclei), spindle damage or changes in DNA conformation and DNA repair (comet assay, formation of foci). The results obtained are summarized in table 5.

Concerning the induction of chromosomal damage, several authors failed to find effects in a frequency range from 900 MHz to 18 GHz. No significant increase in chromosome aberrations was detected by Hansteen and co-workers in human peripheral blood

lymphocytes exposed for 53 h to 2.3 GHz, continuous wave (CW) or pulsed waves (PW, 200 Hz pulse frequency, 50% duty cycle), 10 W/m^2 power density (no SAR value is given), respect to unexposed controls, although a slight increase was detected in PW respect to CW exposed samples (Hansteen et al., 2009a). The authors also confirmed their results at higher frequencies (18 GHz CW, 1 W/m^2 and 16.5 GHz PW, 10 W/m^2) (Hansteen et al., 2009b). Similar findings were also reported for shorter exposure duration (24 h) at lower frequency (1950 MHz) at SAR values of 0.5 and 2 W/kg (Manti et al., 2008). In another investigation, absence of chromosomal rearrangements, either numerical or structural, was found after 24 h exposure of human amniotic cells to 900 MHz, GSM (0.25 W/kg SAR), evaluated soon after and 24 h after RF exposure, by using complete R-banded karyotyping (Bourthoumieu et al., 2010). These results were confirmed by further investigations where the authors found no significant changes in the rate of aneuploidy of chromosome 11 and 17 (Bourthoumieu et al., 2011) and in the expression and activation of the p53 protein at average SARs up to 4 W/kg (Bourthoumieu et al., 2013).

Absence of chromosomal damage was also reported by applying the cytokinesis-block micronucleus (MN) assay under several experimental conditions. Vijayalaxmi and co-workers failed to detect chromosomal damage (MN formation) in PHA stimulated human peripheral blood lymphocytes from four donors after exposure for 2 h to 2450 MHz radiofrequency field, SAR of 10.9 W/kg, at both CW and wideband code division multiple access (WCDMA) modulated field. Melatonin treatment did not change the MN frequency in RF-exposed/sham-exposed cells (Vijayalaxmi et al., 2013). Absence of effects was also confirmed by Waldmann and co-workers. Peripheral blood lymphocyte cultures from 20 healthy donors were intermittently (5 min on/10 min off cycles) exposed for 28 h at the frequency of 1800 MHz, GSM modulation, at SAR values of 0.2, 2 and 10 W/kg. Neither primary DNA damage (comet assay), nor chromosomal damage (MN, CA and SCE) was detected in exposed samples compared to sham exposed ones when slides were analysed in blind mode in three independent laboratories (Waldmann et al., 2013). No increase in MN frequency was detected in human peripheral blood lymphocytes exposed to 900 MHz, GSM (1.25 W/kg mean SAR) given for 20 h in several stages of the cell cycle (Sannino et al., 2009a; 2011). Similar results were obtained by the same research group when 20 h exposures were carried out in the S phase of the cell cycle at 1950 MHz (UMTS) and SAR values of 0.15, 0.3, 0.6 and 1.25 W/kg (Zeni et al., 2012). Moreover, they also reposted absence of effects on DNA integrity (MN assay) and DNA migration (alkaline comet assay) in human fibroblasts from healthy and Turner's syndrome donors after 24 h exposure to 900 MHz, GSM, 1 W/kg SAR (Sannino et al., 2009b). Liu and co-workers failed to detect DNA strand breakage by alkaline comet assay in mouse spermatocyte-derived GC-2 cells exposed to 1800 MHz (GSM-talk mode) for 24 h (5 min on/10 min off cycles) at SAR values of 1, 2 and 4 W/kg. Interestingly, when the FPG modified comet assay was applied, an increase in the extent of DNA migration was found at 4 W/kg SAR, indicating oxidative damage caused by reactive oxygen species at the level of DNA molecule. Oxidative damage was also confirmed by an increase of DNA adducts at 4 W/kg SAR and of ROS production at 2 and 4 W/kg SAR (Liu et al., 2013).

In four investigations, the effect of RF exposure was evaluated in terms of mitotic spindle disturbances.

Shrader and co-workers found a statistically significant increase in the number of mitotic figures with spindle alterations in Human-Hamster hybrid cells (FC2 cells) exposed from 0.5 to 2 h to 835 MHz (calculated SAR of 0.6 W/kg) with a field strength of 90 V/m (Shrader et al., 2008). In a further study they confirmed this result by exposing FC-2 cells to 900 MHz for 30 min (calculated SARs of 0.01 and 0.017 W/kg) and found that the E-field component of the transversal electromagnetic field (E-field strengths of 45 and 90 V/m), but not the magnetic component, is responsible for the observed effect (Schrader et al., 2011).

Defects of spindle assembly were detected in Chinese Hamster V79 fibroblasts exposed for 15 min to 2.45 GHz, CW, at power densities of 50 and 100 W/m^2 (Ballardin et al.,

2011). Moreover, the authors also observed an increase in the number of apoptotic cells. However, they stated that, since most of the literature reports a lack of RF-induced genotoxicity, it is reasonable to speculate that the observed spindle alterations belong to a non-permanent effect.

Zimmerman et al. (2012) showed that very low levels of 27.12 MHz (0.05-1 W/kg) RF given for 21 h (3 h/day for a week) inhibit cancer cell proliferation at specific modulation frequencies by destroying the mitotic spindle. Moreover, alteration of gene expression was also detected. Since the effect was observed in hepatocarcinoma and breast cancer cells, but not in cells from healthy tissues, the authors concluded that their results may have broad implications for the treatment of cancer.

A large number of experiments have been carried out by employing the comet assay to assess the effect of RF on DNA migration. Kumar et al. (2011) exposed rat long bones to 900 MHz, CW, at 2 W/kg SAR for 30 min. After exposure, the bone marrow cells were extracted and analysed. No differences in DNA migration pattern were detected between RF- and sham-exposed cells. Moreover, no differences were found in terms of proliferation and erythrocyte maturation.

Zhijian and co-workers evaluated the effect of intermittent (5 min on/10 min off) RF exposure at 1800 MHz, GSM (2 W/kg), on human white blood cells and human lymphoblastoid B-cell lines (24 h and 2h exposure duration, respectively). In both cases DNA migration was unaffected (Zhijian et al., 2009; 2010).

DNA integrity also resulted unaffected in human neuroblastoma cell lines (SH-SY5Y) after 1 and 3 h exposure to 872 MHz, CW and GSM, 5 W/kg, compared to their respective sham-exposed controls (Luukkonen et al., 2009; 2010).

A transient increase in DNA migration was measured in the human trophoblast HTR-8/SV neo cell line exposed to 1800 MHz at 2 W/kg for 16 or 24 h (5 min on/10 min off cycles). The effect was detected either in GSM basic and GSM talk signal modulation, but it was recovered after 2 h. No effect was found for shorter exposure duration (4 h) and when the field was applied without modulation (CW) (Franzellitti et al., 2010).

Other authors reported an increase in DNA migration induced by RF exposure. Thus, Campisi et al. (2010) exposed primary rat astrocytes for 5, 10 or 20 min to 900 MHz, CW or amplitude modulated at 50 Hz at the same power density of 0.26 W/m^2 (no SAR reported). A significant increase in DNA fragmentation, together with ROS formation, was found after modulated exposure for 20 min. No effects were detected when shorter exposure duration or CW were used (Campisi et al., 2010). Gajski and Garaj-Vrhovac (2009) also found induction of DNA damage, as assessed by the alkaline comet assay and the Fpg-modified comet assay, in rat blood lymphocytes exposed for 30 min to 915 MHz, GSM, at power density of 2.4 W/m^2 (calculated SAR of 0.6 W/kg). An increased DNA fragmentation, together with increased ROS formation and decreased viability and mobility was found in human spermatozoa exposed for 16 h to 1800 MHz at SAR ranging from 0 to 30 W/kg. The effect resulted depending on the SAR value (De Iuliis et al., 2009).

In three investigations, detection of γ-H2AX phosphorylated histone (foci formation) was employed as a measure of RF-induced DNA damage. This technique is capable of detecting DNA damage at levels 100-fold below the detection limit of the alkaline comet assay and foci formation is an early marker of DNA damage.

Xu and co-workers exposed six different cell types to 1800 MHz, GSM (3 W/kg SAR), for 1 or 24 h (5 min on/10 min off cycles). No changes in the average number of foci per cell was detected after 1 h exposure in each of the six cell types examined, while 24 h exposure resulted in a significant increase of foci formation in two cell types. However, the elevated number of foci was not associated with DNA fragmentation (comet assay), cell cycle arrest, cell proliferation or viability changes, although a slight but not statistically significant increase in ROS formation was detected. The authors concluded that RF is able to induce foci formation in a cell-type dependent manner, but the induced

DNA damage may be reversible or compensated by DNA repair pathways (Xu et al., 2013).

Two more studies, carried out by the research group of Dr. Belyaev, evaluated the inhibition of endogenous foci formation by RF exposure. In a first investigation it was demonstrated that 1 h exposure to 915 MHz, GSM (0.037 W/kg SAR) and to 1947.4 MHz, UMTS (0.039 W/kg SAR) of human peripheral blood lymphocytes from normal and hypersensitive donors resulted in a significant inhibition of 53BP1/γ-H2AX DNA-repair foci formation, while no consistent response was observed at 905 MHz (Belyaev et al., 2009). In a further study the authors extended the results obtained on human lymphocytes to human primary fibroblasts and mesenchymal stem cells. Since steam cells exhibited the strongest effect, they suggest that the latter are the most relevant cellular model for validating safe mobile communication signals (Markova et al., 2010).

The effect of RF exposure on the DNA molecule was investigated in a study by Hekmat et al. (2013). They exposed DNA extracted from calf thymus to 940 MHz, 40 mW/kg SAR, for 45 min and different spectroscopic measurements were carried out immediately after RF exposure and 2 h post exposure. The results indicated that RF exposure induced irreversible conformational changes in DNA structure, as assessed by UV-vis spectroscopy and circular dichroism analysis.

A meta-analysis pooled 88 *in vivo* and *in vitro* studies published during 1990-2011 assessing genetic damage in human cells exposed to RF. The authors concluded that the magnitude of difference between RF- exposed and sham-exposed controls was small with some exceptions. Of the six end-points analysed, no effect was found for micronuclei, sister chromatid exchange or SCE foci, while studies using COMET assay showed higher frequencies of changes overall in the exposed than control group, but no exposure gradient in terms of SAR. Results concerning the induction of CA, MN and SCE indicated that, overall, the genotoxicity indices in RF-exposed samples were within the spontaneous values reported in a large database. For the result obtained with the comet assay, although the meta-analysis indicated significant increases in several exposure conditions, the authors stated that some of the increases could be due to the modification of the comet analysis and interpretation of the results (Vijayalaxmi and Prihoda, 2012).

Furthermore, the authors found strong evidence of publication bias in the studies. A skewed (asymmetric) distribution of results in a funnel plot, i.e. substantially larger effect size in small than large studies, suggests that small studies have also been conducted with small or no effect, but they were not published. Small studies with positive results are more likely to be published than those with null or negative results.

As reported in the previous Opinion, Schwarz et al. (2008) found that 24 h exposure of human fibroblasts, but not of lymphocytes, to 1950 MHz, UMTS, at SAR values of 0.05 and 0.1 W/kg, induced a statistically significant increase in DNA damage both in terms of MN frequency and DNA migration (Comet assay). There are several areas of concern about the reported results, including non-credible low standard deviation of reported data, suspiciously low inter-individual differences, indications of data fabrication, inappropriate statistical analysis, and undermined blinding (Lerchl 2009, 2010) This makes the study by Schwarz et al inappropriate for risk assessment. Moreover, in 2013 Speit and co-workers attempted to replicate the induction of micronuclei and DNA migration (alkaline comet assay) in HL-60 cells exposed for 24 h (5 min on/10 min off cycles) to 1800 MHz, CW, at a SAR of 1.3 W/kg. By using the same exposure system and the same experimental protocols as the authors of the original study, they failed to confirm the results. They did not find any explanation for these conflicting results (Speit et al, 2013).

Table 5. ***In vitro*** **studies on genotoxic effects of Radiofrequencies**

Reference	Cell type	Exposure conditions	Results
Hansteen et al., 2009 a)	Human peripheral blood lymphocytes	2.3 GHz CW and PW (200 Hz, 50% dc) 10 W/m^2; 53 h	No significant increase in CA. Slight increase with PW than with CW
Hansteen et al., 2009 b)	Human peripheral blood lymphocytes	18 GHz CW 1W/m^2 16.5 GHz PW (1 kHz, 50% dc) 10 W/m^2; 53 h	No significant increase in CA
Manti et al., 2008	Human peripheral blood lymphocytes	1950 MHz, UMTS, 0.5 & 2 W/kg; 24 h	No effects on CA
Bourthoumieu et al., 2010	Human Amniotic cells	900 MHz, GSM-217, 0.25 W/kg; 24 h	No cytogenetic effects (R-banded caryotyping), evaluated immediately after exposure and after 24 h.
Bourthoumieu et al., 2011	Human Amniotic cells	900 MHz, GSM-217, 0.25, 1, 2, 4 W/kg; 24 h	No aneuploidy of chromosome 11 & 17
Bourthoumieu et al., 2013	Human Amniotic cells	900 MHz, GSM-217, 0.25, 1, 2, 4 W/kg; 24 h	No changes in expression and activation of p53
Vijayalaxmi et al., 2013	Human peripheral blood lymphocytes	2450 MHz (CW, WCDMA) 10.9 W/kg, 2 h	No effects on MN frequency.
Waldmann et al., 2013	Human peripheral blood lymphocytes	1800 MHz (GSM) (5 min on/10 min off cycles) 0.2, 2 and 10 W/kg, 28 h	No effects on DNA breakage; No chromosomal damage (MN, SCE, SCE)
Sannino et al., 2009a	Human peripheral blood lymphocytes	900 MHz, GSM, 1.25 W/kg mean SAR 20 h (from 24 to 44h after PHA)	No effect on DNA damage (MN)
Sannino et al., 2011	Human peripheral blood lymphocytes	900 MHz, GSM, 1.25 W/kg mean SAR 20 h in several stages of the cell cycle	No effect on DNA damage (MN)
Zeni et al., 2012	Human peripheral blood lymphocytes	1950 MHz, UMTS, 1.25, 0.6, 0.3 and 0.15 W/kg; 20 h (from 24 to 44h after PHA)	No effect on DNA damage (MN)
Sannino et al., 2009b	Human fibroblasts from healthy (ES-1) and Turner's syndrome donors	900 MHz, GSM, 1 W/kg mean SAR; 24 h	No effect on DNA integrity (MN) and DNA migration(comet)
Liu et al., 2013	Mouse spermatocyte-derived GC-2 cells	1800 MHz (GSM-talk mode) (5 min on/10 min off cycles) 1, 2 and 4 W/kg, 24 h	No effects on DNA breakage; oxidative DNA damage at 4 W/kg SAR.
Schrader et al., 2008	Human –Hamster hybrid (ALCells) (FC2)	835 MHz E field: 90 V/m; calculated SAR: 0.6 W/kg; 0.5 – 2 h	Spindle disturbances in anaphase and telophase
Schrader et al., 2011	Human –Hamster hybrid (ALCells) (FC2)	900 MHz H & E field separated E: 45 and 90 V/m; calculated SAR: 0.01-0.017 W/kg; 0.5 h	Spindle disturbances in anaphase and telophase in cultures exposed to the E component of the EMF
Ballardin et al., 2011	Chinese Hamster V79 cells	2.45 GHz, CW 50, 100 W/m^2; 15 min	decrease in mitotic index and increase in apoptosis; reversible increase of aberrant spindles as a function of the power density
Zimmerman et al., 2012	Human hepatocellular carcinoma cells (HepG2), breast cancer cells, hepatocytes & breast epithelial cells	27.12 MHz Tumour-specific modulation; 21 h 0.05 – 1 W/kg	Decrease in cell proliferation and mitotic spindle disruption and alteration of gene expression by specific modulation frequencies only in cancer cells

Kumar et al., 2010	Rat bone marrow cells, erythrocytes and lymphocytes	900 MHz CW 2 W/kg; 0.5 h	No effect on proliferation, erythrocyte maturation and DNA damage (comet)
Zhijian et al., 2009	Human white blood cells	1800 MHz, GSM, 2W/kg; 24 h (5 min on, 10 min off)	No effect on DNA migration (comet)
Zhijian et al., 2010	Human lymphoblastoid B-cells (HMy2.CIR)	1800 MHz, GSM, 2W/kg; 2 h (5 min on, 10 min off) with several exposure schedules	No effect on DNA migration (comet)
Luukkonen et al., 2009	Human neuroblastoma (SH-SY5Y)	872 MHz, CW and GSM, 5 W/kg; 1 h	No effect on DNA migration (comet assay) and ROS production
Luukkonen et al., 2010	Human neuroblastoma (SH-SY5Y)	872 MHz, CW and GSM, 5 W/kg 1 h (ROS) or 3 h (DNA migration)	No effects in terms of ROS production, DNA damage and cell viability for all the experimental conditions tested
Franzellitti et al., 2010	Human Trophoblasts (HTR-8/SV neo cells)	1800 MHz; GSM 217, GSM talk, CW 2 W/kg; 4, 16, 24 h (5 min on/10 min off)	Increase in DNA migration (GSM-217, GSM-Talk, 16 and 24 h). Recovery in 2 h No effect of CW
Campisi et al., 2010	Primary rat astrocytes	900 MHz, CW and amplitude modulated (50 Hz) 0.26 W/m^2; 5, 10, 20 min	Increased ROS formation and DNA fragmentation after 20 min modulated exposure. No effects for CW exposures
Gajski and Garaj-Vrhovac, 2009	rat blood lymphocytes	915 MHz, GSM, 2.4 W/m^2 (calculated SAR 0.6 W/kg); 30 min	Induction of DNA damage, assessed by the alkaline comet assay and Fpg-modified comet assay
De Iuliis et al., 2009	Human spermatozoa	1800MHz 0-30 W/kg (mean SAR 27 W/kg); 16 h	Decreased viability and mobility. Increased ROS formation and DNA fragmentation as a function of the SAR
Xu et al., 2013	Chinese hamster lung cells; lung rat astrocytes; Human amniotic epithelial cells; human lens epithelial cells; human skin fibroblasts; umbilical vein endothelial cells	1800 MHz GSM 3 W/kg; 1, 24 h (5 min on/10 min off)	Cell type-dependent increase in foci, without alteration in DNA fragmentation, cell cycle progression, cell proliferation, ROS formation.
Belyaev et al., 2009	Human peripheral blood lymphocytes from normal and hypersensitive donors	905 or 915 MHz, GSM 0.037 W/kg; 1 h; 1947,4 MHz, UMTS 0.039 W/kg; 1 h	Inhibition of DSB (foci) by 915 MHz, GSM and UMTS exposure. Differences not statistically significant for 905 MHz.
Markova et al., 2010	Human diploid fibroblasts (VH-10), human mesenchymal stem cells (HMSc)	905 or 915 MHz, GSM 0.037 W/kg; 1-3 h; 1947,4 MHz, UMTS 0.039 W/kg; 1-3 h	Inhibition of DSB (foci). Effect cell-type dependent after 1 h exposure. No increase for longer exposure duration
Hekmat et al. (2013)	DNA extracted from calf thymus	940 MHz, 40 mW/kg SAR 45 min	conformational changes in DNA structure
Speit et al., 2013	Human promyelocytic leukaemia cells (HL60)	1800 HMz, CW 1.3 W/kg SAR; 24 h (5 min on/10 min off)	No effect on DNA integrity (MN) and DNA migration (comet) Repetition study of Schwarz et al, 2008

CA: chromosome aberration; CW: continuous wave; DSB: double strand breaks; E: electric; M: magnetic; MN: micronuclei; PHA: phytohemagglutinin; PW: pulsed wave; ROS: reactive oxygen species.

Non genotoxic effects

In vitro investigations have been carried out on different cell processes related to non-genotoxic carcinogenesis, such as cell death (apoptosis), cell cycle progression, oxidative stress, gene and protein expression as well as other metabolic and molecular changes.

In several studies the induction of apoptosis has been investigated after exposure to RF ranging from 900 to 2450 MHz, as reported in Table 6.

Exposures to 900 MHz, GSM, 1 W/kg SAR for 24 to 144 h did not induce apoptosis in rat primary cortical neurons as well as in murine SN56 cholinergic neurons. The exposure also failed to induce effects on viability and proliferation (Del Vecchio et al., 2009a). Similar results were found in human spermatozoa exposed for 1 h to 900 MHz, GSM, at SAR of 2 and 5.7 W/kg. At various times after exposure no differences with respect to un-exposed controls were detected in terms of caspase-3 activity, externalization of phosphatidylserine, DNA strand breaks and generation of ROS (Falzone et al., 2010).

One hour exposure of human lymphoblastoma (Jurkat) cells and peripheral blood lymphocytes, either proliferating or quiescent, to 900 MHz, GSM, at a mean SAR of 1.35 W/kg also provided no evidence for induction of apoptosis, although a slight but statistically significant increase in caspase 3 activity was detected in proliferating but not in quiescent cells. Since several studies detected an involvement of caspases in processes other than apoptosis, the authors also evaluated viability and cell cycle in proliferating lymphocytes exposed to RF. However, no effects were detected in cell cycle kinetics at 6, 24 and 48 h after 1 h exposure (Palumbo et al., 2008).

Moquet et al. (2008) confirmed the lack of apoptosis in proliferating as well as in differentiated murine neuroblastoma N2a cell line exposed for 24 h to 935 MHz at 2 W/kg SAR. These findings were obtained by testing three signal types (CW, GSM basic and GSM talk) and by employing several tests to measure apoptosis.

Simon et al. (2013), exposed reconstruct epidermis from human skin biopsy to 900 MHz, GSM basic for 6 h at an SAR level of 2 W/kg. After RF exposure, growth medium was replaced with fresh medium, and reconstructed epidermis was incubated at 37 °C and 5% CO2 for 2, 6, 18 or 24 h. Results indicated no significant variation of p53 and caspase 3 in exposed samples compared to sham exposed ones. In this study global protein oxidation, proliferation and differentiation were also evaluated in the same experimental conditions. No effects were detected, except for a slight alteration of differentiation markers level.

In contrast, Joubert and co-workers, using a 900 MHz CW signal exposed rat primary cortical neurons for 24 h with an SAR of 2 W/kg and detected a significant difference in the apoptosis frequency with respect to sham-exposed cells, as assessed by DAPI staining and TUNEL. During these RF exposures, a temperature rise of 2°C was noted and therefore control experiments with cells exposed to 37 and 39°C were also performed. Overall, the results suggested that the induction of apoptosis is independent of changes in temperature. As the apoptosis rate in the RF-exposed cells was significantly different from these controls, they concluded that they may have seen an effect of RF fields (Joubert et al., 2008). In a further study, the same research group exposed rat cerebral cortical cells for 24 h to 900 MHz, GSM, but to a lower SAR (0.25 W/kg). No induction of apoptosis was detected, but an increase in HSC70 and a decrease in HSP90 expression was observed. Since comparable effects were also observed in cells incubated at 37.5°C, the authors concluded that the induced changes are most likely linked to temperature increase (Terro et al., 2012).

Ballardin et al. (2011) detected an increase in apoptosis when V79 cells were exposed for 15 min to 2450 MHz, CW, at power density of 50 and 100 W/m^2. The frequency of apoptotic cells increased with the increase of the applied power density of the incident field. The authors excluded thermal effects since treatments with thermostatic baths induced apoptosis only when the temperature exceeded 40°C.

Kahya et al. (2014) exposed a breast cancer cell line derived from metastatic site (MDA-MB-231) to 900 MHz modulated at 217 Hz for 1 h at an average calculated SAR of 0.36±0.02 W/kg. They found induction of apoptosis, including increased caspases 3 and 9 activities, and increased mitochondrial membrane depolarization in compared to control cells. Moreover, oxidative stress was also induced (increased ROS formation). These levels decreased towards controls when treatment of 1h with sodium selenite (200 nM), a well-known antioxidant agent, was carried out before RF exposure. In this study no sham

exposures were carried out and the results obtained in exposed samples were compared to their respective controls.

In another study the ability of RF to induce apoptosis and to act as a tumour-promoting agent in rat astrocytes and C6 glioma cells was investigated. For this purpose, cell cultures were exposed for 12, 24 or 48 h to 1950 MHz at 5.36 W/kg by employing the Time Division Synchronous Code Division Multiple Access (TD-SCDMA), a 3-G standard currently employed in UMTS mobile telecommunication networks in China. A significant increase in apoptotic cells (annexin-V assay and caspase 3 activation), together with down-regulation of bcl-2 and up-regulation of bax mRNA levels and inhibition of cell growth was detected after 48 h exposure of astrocytes. No effects were found for shorter exposure times. C6 glioma cells resulted unaffected for all the experimental conditions tested. Moreover, when exposed cells were injected into mice no tumour induction was produced (Liu et al., 2012).

Table 6. ***In vitro*** **studies on effects of RF exposure on apoptosis**

Reference	Cell type	Exposure conditions	Results
Del Vecchio et al., 2009b	Rat primary cortical neurons<br>Murine SN56 cholinergic neurons	900 MHz GSM<br>1 W/kg; 24-144 h	No effect on viability, proliferation, apoptosis
Falzone et al., Rad Res, 2010	Human spermatozoa	900 MHz, GSM<br>2 and 5.7 W/kg; 1 h	No effects on apoptosis, DNA strand breaks and ROS
Palumbo et al., 2008	Human peripheral blood lymphocytes; Jurkat cells	900 MHz, GSM<br>1.35 W/kg mean SAR; 1h	Increase in caspase 3 activity in proliferating but not in quiescent cells.<br>No effect on apoptosis and cell cycle distribution
Moquet et al., 2008	Murine Neuroblastoma (N2a)	935 MHz; CW, GSM basic, GSM talk<br>2 W/kg; 24 h	No apoptosis using three different assays
Simon et al., 2013	Reconstruct epidermis from human skin biopsy	900 MHz, GSM basic<br>2 W/kg<br>6 h.<br>Analysis after 2, 6, 18 and 24 h post exposure.	No significant variation of p53 and caspase 3.<br>No effect on global protein oxidation.<br>Slight alteration in differentiation markers.
Joubert et al., 2008	Rat primary cortical neurons	900 MHz CW<br>2 W/kg; 24 h	Induction of apoptosis, no caspase-3 activation, increase in AIF-positive cells
Terro et al., 2012	Rat Cerebral cortical cells	900 MHz, GSM<br>0.25 W/kg; 24 h	No induction of apoptosis and protein degradation.<br>Increased expression of HSC70; decreased expression of HSP90
Ballardin et al., 2011	Chinese Hamster V79 cells	2.45 GHz, CW<br>50, 100 W/m^2;<br>15 min	decrease in mitotic index and increase in apoptosis; reversible increase of aberrant spindles as a function of the power density
Kahya et al., 2014	Breast cancer metastatic cell line (MDA-MB-231)	900 MHz (217 Hz)<br>0.36±0.02 W/kg<br>1 h	Increased apoptosis (caspase-3 and caspase-9 activities), mitochondrial membrane depolarization and ROS levels.<br>Effect suppressed by pre-treatment with selenium.
Liu et al., 2012	Rat astrocytes and C6 glioma cells	1950 MHz, TD-SCDMA, 5.36 W/kg; 12, 24, 48 h	Damage of mitochondria and induction of apoptosis after 48 h exposure in astrocytes but not in C6 cells. No tumour formation in mice after injection of exposed cells.

AIF: apoptosis-inducing factor; CW: continuous wave; HSC: Heat shock cognate; HSP: Heat shock proteins; ROS: reactive oxygen species; TD-SCDMA: time division-synchronous code division multiple access.

Most of the studies devoted to assess the capability of RF exposure to modify the oxidation state of the cells have been carried out by measuring reactive oxygen species (ROS) formation, although in some cases other targets related to oxidative stress have

been evaluated, such as antioxidant enzyme activity, glutathione (GSH) depletion, mitochondrial RNA. The details of the reviewed studies are presented in table 7.

Luukkonen et al. investigated ROS formation in SH-SY5Y human neuroblastoma cells exposed for 1 h to 872 MHz, CW and GSM signal, 5 W/kg SAR. The results did not show evidence of differences when comparing RF and sham-exposed cultures (Luukkonen et al., 2009). Under similar exposure conditions (900 MHz, GSM, 2 or 5.7 W/kg SAR) Falzone and co-workers confirmed that 1 h RF exposure was not able to induce ROS formation in human spermatozoa, a cell model particularly susceptible to oxidative stress (Falzone et al., 2010). 2 h exposure of human breast epithelial cells (MCF10A) to 837 MHz (CDMA) or to 1950 MHz (WCDMA) at SAR of 4 W/kg also did not induce oxidative stress in terms of ROS formation, GSH depletion and Superoxide Dismutase (SOD) activity (Hong et al., 2012). In another study Brescia et al. also provided no evidence for ROS increase in human lymphoblastoid T cells (Jurkat) exposed to 1950 MHz, UMTS, at SAR of 0.5 and 2 W/kg for short (5-60 min) or long (24 h) exposure duration (Brescia et al., 2009). Similar results were obtained by Poulletier de Gannes et al. (2011), who investigated the effect of the Enhanced Data rate for GSM Evolution (EDGE) signal on three brain human cell lines (SH-SY5Y, U87 and CHME5) and primary cortical neuron cultures. Exposures to 1800 MHz were carried out and four conditions were tested: 2 and 10 W/kg for 1 and 24 h. For all the experimental conditions tested, RF exposure was not able to increase ROS production.

In several investigations an increase in ROS formation was reported under different exposure conditions.

Liu et al. (2013) exposed mouse spermatocyte-derived GC-2 cells exposed to 1800 MHz (GSM-talk mode) for 24 h (5 min on/10 min off cycles) at SAR values of 2 and 4 W/kg and measured ROS levels and oxidative DNA base damage by measuring the levels of 8-oxoG. RF exposure increased the generation of ROS in a SAR-dependent manner, and 4 W/kg exposure also increased the levels of the DNA base damage. Pre-treatments with the antioxidant tocopherol blocked RF exposure-increased ROS production and 8-oxoG levels at a SAR value of 4 W/kg.

Another research group (Liu et al., 2014) by employing the same cell model and the same experimental conditions demonstrated a dose dependent increase in ROS levels at 2 and 4 W/kg SAR and an increased autophagy.

Sefidbakht et al. (2014) exposed human embryonic kidney cells (HEK293T) for 15, 30, 45, 60 and 90 min to 940 MHz RF field at an average SAR of 0.09 W/kg. They detected an increase in ROS formation after 30 min exposure, followed by a sharp rise in catalase and superoxide dismutase activities and elevation of glutathione content during the 45 min exposure with a concomitant decrease in lipid peroxidation. The authors concluded that RF exposure is capable of activate the stress response by an immediate increase in ROS levels.

Ni et al. (2013) investigated the induction of oxidative stress in human lens epithelial B3 (HLE-B3) cells intermittently exposed (5 min on/10 min off cycles) to a 1800 MHz RF EMF, GSM signal (average SAR=2, 3 and 4 W/Kg). The ROS levels were measured (DCFH-DA assay) in cells exposed for 0.5, 1, and 1.5 h. Lipid peroxidation was detected by a Malondialdehyde test (MDA, a member of a family of final products of lipid peroxidation) in cells exposed for 6, 12, and 24 h. The mRNA expression of SOD1, SOD2, CAT, and GPx1genes and the expression of SOD1, SOD2, CAT, and GPx1 proteins were measured by qRT-PCR and Western blot assays in the cells exposed for 1 h. For all the experimental conditions tested, in the RF exposed cultures ROS and MDA levels significantly increased and mRNA and protein expression significantly decreased in comparison to sham-exposed ones; cell viability also decreased (three independent experiments for each exposure condition/endpoint examined).

Xu et al. reported that intermittent exposures (5 min on/ 10 min off) of rat primary neurons for 24 h at 1800 MHz, GSM, 2 W/kg SAR, induced an increase in ROS production and in the levels of 8-hydroxyguanine (8-OHdG), a common biomarker of DNA oxidative

damage in mitochondrial DNA (mtDNA), and a concomitant reduction in the copy number of mtDNA and the levels of mtRNA transcripts. However, such effects were demonstrated to reverse by pre-treatment with melatonin, an efficient antioxidant in the brain (Xu et al., 2010). In a more recent investigation, the same research group evaluated ROS formation on six different cell types after 1 and 24 h intermittent exposure (5 min on/10 min off) at 1800 MHz, GSM, 3 W/kg SAR. No differences were detected when comparing exposed and sham-exposed cultures. The study also provided no indication of alteration in cell proliferation and cell cycle progression (Xu et al., 2013).

An increase in ROS formation, together with enhanced DNA fragmentation, was reported by Campisi et al. on primary rat astrocytes exposed for 20 min to 900 MHz amplitude modulated at 50 Hz, 0.26 W/m^2 power density (no SAR value is given). No effects were detected when shorter exposure duration (5 or 10 min) or CW exposures were performed (Campisi et al., 2010). In this study the absence of dosimetric details makes the results difficult to comment.

De Iuliis et al., after 16 h exposure at 1800 MHz, SAR from 0.4 up to 27.5 W/kg also found an increase in ROS generation by the whole cell and mitochondria in a SAR-dependent manner, together with oxidative DNA damage (8-OHdG) and DNA fragmentation. Such effects translated to reduction in sperms motility and vitality. The authors claimed that their results clearly demonstrated that RF exposure can damage sperm function via mechanisms involving the leakage of electrons from the mitochondria and the induction of oxidative stress, but the employed SAR values are very high and not relevant to cell phone users.

Table 7. ***In vitro* studies on effects of RF exposure on oxidative stress**

Reference	Cell type	Exposure conditions	Results
Luukkonen et al., 2009	Human neuroblastoma cells (SH-SY5Y)	872 MHz, CW and GSM, 5W/kg; 1 h	No effect on DNA migration (comet assay) and ROS production
Luukkonen et al., 2010	Human neuroblastoma (SH-SY5Y)	872 MHz, CW and GSM, 5W/kg 1 h (ROS) or 3 h (DNA migration)	No effects in terms of ROS production, DNA damage and cell viability for all the experimental conditions tested
Falzone et al., 2010	Human spermatozoa	900 MHz, GSM 2 and 5.7 W/kg; 1 h	No effects on apoptosis, DNA strand breaks and ROS
Hong et al., 2012	Human breast epithelial cells (MCF10A)	837 MHz; CDMA, 4 W/kg; 1950 MHz; WCDMA, 4 W/kg; 2h	No induction of oxidative stress (ROS formation, SOD activity and GSH depletion)
Brescia et al., 2009	Human lymphoblastoid T cells (Jurkat)	1950 MHz, UMTS, 0.5 and 2 W/kg 5-60 min, 24 h	No effects on ROS production and cell viability for all the experimental conditions tested
Poulletier de Gannes et al., 2011	Brain human cell lines (SH-SY5Y; U87; CHME5)	1800 MHz, EDGE 2 and 10 W/kg; 1 and 24 h	No increase in ROS production
Liu et al., 2013	Mouse spermatocyte-derived GC-2 cells	1800 MHz (GSM-talk mode) (5 min on/10 min off cycles) 1, 2 and 4 W/kg, 24 h	Increased ROS levels at 2 and 4 W/kg SAR.
Liu et al., 2014	Mouse spermatocyte-derived GC-2 cells	1800 MHz (GSM-talk mode) (5 min on/10 min off cycles) 1, 2 and 4 W/kg, 24 h	Dose-dependent increase in ROS levels, increased autophagy
Sefidbakht et al., 2014	Human embryonic kidney cells (HEK293T)	940 MHz 0.09 W/kg, 15-90 min	Increased ROS levels after 30 min, Increase in CAT and SOD activity and Glutathione content.
Ni et al., 2013	Human lens epithelial cells (HLE-B3)	1800 MHz, GSM (5 min on/10 min off) 2, 3 and 4 W/kg; 0.5-24 h	For all the condition tested: Increase in ROS and MDA levels; Decrease in mRNA and SOD, CAT and GPx1 proteins; Decrease in cell viability
Xu et al., 2010	Rat cortical neurons	1800 MHz, GSM (5 min on/10 min off) 2 W/kg; 24 h	Decrease in 8-OHdG levels in mitochondria; reduced levels of mtDNA and mtRNA, reverted by pre-treatment with melatonin
Xu et al., 2013	Chinese hamster lung rat astrocytes; Human amniotic epithelial cells; human lens epithelial cells	1800 MHz GSM 3 W/kg; 1, 24 h (5 min on/10 min off)	Cell type-dependent increase in foci, without alteration in DNA fragmentation, cell cycle progression, cell proliferation, ROS formation.
Campisi et al., 2010	Primary rat astrocytes	900 MHz, CW and amplitude modulated (50 Hz); 0.26 W/m^2; 5, 10, 20 min	Increased ROS formation and DNA fragmentation after 20 min exposure. No effects for CW exposures
De Iuliis et al., 2009	Human spermatozoa	1800 MHz 0-30 W/kg (mean SAR 27 W/kg); 53 h	Decreased viability and mobility. Increased ROS formation and DNA fragmentation as a function of the SAR

8-OHdG: 8-hydroxyguanine; CAT: catalase; CW: continuous wave; EDGE: Enhanced Data rate for GSM Evolution; GPx: Glutathione Peroxidase; GSH: Reduced Glutathione; MDA: Malondialdehyde; mtDNA: mitochondrial DNA; mtRNA: mitochondrial RNA; ROS: reactive oxygen species; SOD: Superoxide dismutase.

Several studies have been carried out to investigate the effects of RF exposure on cell proliferation, cell cycle progression and other cancer-related endpoints. They are summarized in table 8.

No effects on cell cycle progression were detected in several cell types exposed intermittently (5 min of/ 10 min off) to 1800 MHz, GSM, 3 W/kg SAR for 1 or 24 h (Xu et al., 2013). Similar results were obtained by Lee et al. on human breast MCF7 cancer cells exposed for 1 h to 837 MHz (CDMA, 4 W/kg SAR). The authors found no effects on cell cycle distribution and on cell cycle regulatory protein expression (Lee et al., 2011a).

Beneduci and co-workers also reported no effects on cell proliferation and cell cycle kinetics after 1 h or 4 days exposure of human skin melanoma cells at 42.2 and 53.57 GHz, CW (1.4 and 3.7 W/m^2, respectively) (Beneduci et al., 2009).

In a study carried out to investigate the response of two human cancer cell lines to a 24 h exposure to 2200 MHz pulse-modulated (5 μs pulse duration, 100 Hz repetition rate) at an average SAR of 0.023 W/kg, a consistent reduction in cell number together with an increased proportion of cells in G0/G1 and G2/M phase was found. The effect was detected in neuroblastoma but not in hepatocarcinoma cells. The authors stated that the cytostatic response observed is cell-type specific (Trillo et al., 2011).

Different results on the basis of the exposure conditions were reported by Ozgur and co-workers who employed human hepatocellular carcinoma cells to evaluate the effects of GSM EDGE signals at 900 and 1800 MHz. Exposures were carried out for 1, 2, 3 and 4 h in intermittent mode (15 min on/15 min off) at an average SAR of 2 W/kg. When compared with their respective sham exposed cells, an increase in cell proliferation was observed after 1h exposure to 1800 MHz while a significant decrease was observed in both the 900 and 1800 MHz exposed cells after 4 h exposure. No effects in the other conditions were detected. Moreover, significant increase in LDH and glucose levels released into the medium were observed after 4 h exposure to either the 900 and 1800 MHz RFR, associated with the presence of morphological changes characteristic of apoptosis (Ozgur et al., 2014).

The enzyme Ornithine Decarboxylase (ODC) acts in cell cycle regulation and its activity after RF exposure has been investigated in the past by several research groups with conflicting results. Two investigations conducted by Billaudel and co-workers reported negative effects of RF exposure on different cell types and under different exposure conditions. In particular, ODC activity resulted unaffected in L929 cells exposed to a) 825 MHz and 872 MHz, Digital Advanced Mobile Phone System (DAMPS) standard for 8 h at 0.5-2.5 W/kg, b) 900 MHz, GSM, for 2 h at 0.5-2.5 W/kg and c) 1800 MHz, GSM for 2 or 24 h at 2.5 W/kg (Billaudel et al., 2009a). The results were confirmed on human neuroblastoma SH-SY5Y cells exposed for 8 or 24 h to 835 MHz (DAMPS) or 1800 MHz (GSM) at 1 or 2.5 W/kg SAR (Billaudel et al., 2009b).

In two papers the capability of RF exposure to induce cellular neoplastic transformation was investigated. Yang et al. exposed NIH 3T3 cells to 916 MHz, CW, at 10, 50 or 90 W/m^2 power density for 2 h/day up to 8 weeks (no SAR value given). They detected a changed morphology of exposed cells. Moreover, when exposed cells were inoculated into mice, the development of lumps was induced. The authors concluded that RF exposure can promote neoplastic transformation of NIH 3T3 cells (Yang et al., 2012). The results reported are very interesting, but the study lacks rigorous dosimetry. Different results were reported by Hirose et al. They exposed embryonic mouse fibroblasts to 2142 MHz, W-CDMA, at SAR of 0.08 or 0.8 W/kg for 6 weeks. The number of transformed foci resulted in similar exposed and sham-exposed cultures, suggesting that RF is not capable of inducing cell transformation (Hirose et al., 2008). In a further study the same research group also reported lack of activation of rat microglial cells after 2 h exposure at 1950 MHz (IMT-2000), W-CDMA, 0.2, 0.8 or 2 W/kg SAR. Furthermore, no differences in the production of tumour necrosis factor-α (TNF-α), interleukin-1β and interleukin-6 between exposed and sham-exposed cultures was detected (Hirose et al., 2010).

Rao et al. evaluated the effect of RF exposure on cell differentiation. Mouse embryonic carcinoma cells were exposed for 1 h at a frequency ranging from 700 to 1100 MHz. Intracellular Ca^{++} spikes , which trigger proliferation and differentiation, resulted increased in retinoic-acid differentiated cells as a function of frequency (at 0.05 W/kg) and SAR (at 800 MHz) (Rao et al., 2008).

Del Vecchio et al. showed that long duration exposure to 900 MHz, GSM, at 1 W/kg decreased neurite number and increased β-thymosine gene expression in rat primary cortical neurons (5 days exposure) and in murine SN56 cholinergic neurons (3 days exposure). However, both cell types recovered after 6 days (Del Vecchio et al., 2009a).

Table 8 *In vitro* studies on effects of RF exposure on cell proliferation, cell cycle and other cancer-related endpoints

Reference	Cell type	Exposure conditions	Results
Xu et al., 2013	Chinese hamster lung rat astrocytes; Human amniotic epithelial cells; human lens epithelial cells	1800 MHz GSM 3 W/kg; 1 or 24 h 5 min on/10 min off	Cell type-dependent increase in foci, without alteration in DNA fragmentation, cell cycle progression, cell proliferation, ROS formation.
Lee et al., 2011a	Human breast cancer cells (MCF7)	CDMA (837 MHz) 4 W/kg; 1 h	No effects on DNA synthesis, cell cycle distribution and cell cycle regulatory proteins.
Beneduci et al. 2009	Human skin melanoma cells	42.2 and 53.57 GHz, CW 1.4 and 3.7 W/m^2; 1h day/4 dd	No effects on cell proliferation and cell cycle
Trillo et al., 2011	Human hepatocarcinoma (HepG2) and neuroblastoma (NB69) cells	2200 MHz pulse modulated; 0.023 W/kg; 24 h	Cytostatic effect cell-type specific
Ozgur et al., 2014	Human hepatocarcinoma cells (Hep G2).	900 MHz, 1800 MHz 15 min on/15 min off cycle 2 W/kg 1, 2, 3, or 4 h	Increased cell proliferation after 1h exposure to 1800 MHz. Decreased cell proliferation in 900 and 1800 MHz exposed cells after 4 h exposure. Increased LDH and glucose levels and presence of morphological changes characteristic of apoptosis.
Billaudel et al., 2009a	Mouse fibrosarcoma cells (L929)	835 MHz, DAMPS (0.5-2.5 W/kg; 8h) 900 MHz, GSM (0.5-2.0 W/kg; 2h) 1800 MHz, GSM (2.5 W/kg; 2-24h)	No effects on ODC activity
Billaudel et al., 2009b	Human neuroblastoma cells (SH-SY5Y)	835 MHz, DAMPS 1800 MHz, GSM 1 or 2.5 W/kg; 8-24h	No effects on ODC activity
Yang et al., 2012	NIH-3T3	916 MHz, CW 10, 50, 90 W/m^2; 2 h/day up to 8 weeks	Morphological transformation. Lumps formation in mice inoculated with exposed cells
Hirose et al., 2008	Embryonic mouse fibroblasts BALB/3T3	2142 MHz W-CDMA 0.08 or 0.8 W/kg; 6 weeks	Neither malignant cell transformation nor tumour promotion
Hirose et al., 2010	Rat primary microglial cells	1950 MHz IMT-2000, W-CDMA 0.2, 0.8, 2 W/kg; 2 h	No activation of microglial cells. No production of TNF-α, IL-1ß, IL-6
Rao et al., 2008	Mouse Embryonic carcinoma cells (P19)	700-1100 MHz 0.5 W/kg; 1 h 800 MHz 0.5, 1.61, 5, 50 W/kg; 1 h	No effects on cell viability. Increase in Ca^{2+} spiking in retinoid-acid differentiated cells as a function of frequency at 0.5 W/kg and SAR (at 800 MHz)
Del Vecchio et al., 2009a	Rat primary cortical neurons	900 MHz GSM 1 W/kg	Decrease in neurite number, increase in ß-thymosine gene

	Murine SN56 cholinergic neurons	3 (SN56) and 5 (primary neurons) days	expression in both cell types. Recovery after 6 days

DAMPS: Digital Advanced Mobile Phone System; IL-1β: Interleukin-1β; IL-6: Interleukin-6; IMT-2000: International Mobile Telecommunications-2000; ODC: Ornithine Decarboxylase; ROS: reactive oxygen species; TNF: Tumour Necrosis Factor; W-CDMA: wideband-Code Division Multiple Access.

A large number of studies have been carried out to evaluate the effect of RF on gene and protein expression. They are reported in table 9.

In six investigations the expression of heat shock proteins (HSPs) has been evaluated, since they are regarded as cellular stress markers and have been reported to be affected by several environmental stressors, including RF. Exposure of human endothelial cells (EA.hy926) for 1 h to 1800 MHz, GSM, 2W/kg SAR provided no evidence for increase of HSP27 expression (Nylund et al., 2009). In a further study the authors confirmed their results on human umbilical vein (HUVEC) and brain endothelial (HBMRC) cells exposed in the same experimental conditions (Nylund et al., 2010). In both investigations the authors found altered expression of several not identified proteins but these findings were not confirmed by western blotting or resulted as artifacts.

By applying intermittent exposures (5 min on/10 min off cycles) Franzellitti et al. also reported lack of effects on HSP expression in human trophoblast cells exposed for 4, 16 or 24 h to 1800 MHz, GSM, 2 W/kg SAR, although changes in one (HSP70C) over 4 transcript isoforms was detected (Franzellitti et al., 2008).

Lack of effects on HSP expression and phosphorylation was also reported by Kim et al. (2012) on human breast epithelial (MCF10A) cells exposed to 837 MHz, CDMA, 4W/kg SAR, for 4h or 2h on three consecutive days. On the contrary, an increased expression of HSP70 and a decreased expression of HSP90 was found in rat cerebral cortical cells exposed for 24h to 900 MHz, GSM, 0.25 W/kg SAR (Terro et al., 2012). Nevertheless, it must be noted that the authors reported a 0.5°C difference between sham and RF exposed samples, which could be responsible for the observed differences.

Gerner et al. intermittently exposed (5 min on/10 min off) Jurkat cells, human fibroblasts and mononuclear cells to 1800 MHz, 2 W/kg SAR, for 8 h. They detected no effects on protein expression, but a higher level of $^{35-}$S incorporated proteins, including HSPs (Gerner et al., 2010).

In one study the effect of millimetre waves was assessed on human astrocytoma-derived cells (U-251) exposed to 59-61 GHz (2.64-3.30 W/kg). After 24 h exposure no variation in the expression of HSP70 and on endoplasmic reticulum stress-responsive chaperon proteins was measured (Nicolaz et al., 2009).

Sun et al. (2012) reported an increased epidermal growth factor (EGF) receptor clustering and phosphorylation in human amniotic cells exposed to 1800 MHz, GSM, from 0.5 to 4 W/kg for 15 minutes.

In another paper an increased transcript expression of IGF-1, increased phosphorylation of MAPK1 and protein expression of BCL-2 and cyclin D1, together with a decreased expression of BX was detected by Yoon et al. (2011) in human dermal cells exposed to 1763 MHz, CDMA, at 2 or 10 W/kg SAR for 3 h.

Cervellati and co-workers also reported an increased expression of genes for connexions, together with changes in cellular localization when human trophoblasts were exposed for 1 h to 1817 MHz, GSM (2 W/kg SAR). However, no variation in terms of expression of these membrane proteins was detected (Cervellati et al., 2009).

Genomic and proteomic techniques have been applied by some researchers to evaluate the effects of RF exposure. None of these studies have reported any significant difference between exposed and unexposed samples.

Roux et al. failed to find differences in gene expression of normal human keratinocytes exposed to 900 MHz, CW, for 10 min (2.6 W/kg) or 30 min (0.73 W/kg) compared to

sham-exposed cultures. As a matter of fact, some genes had a different expression but this result was not confirmed by RT-PCR (Roux et al., 2010).

Sakurai et al. (2011) also found altered gene expression not confirmed by RT-PCR in human-derived glial cells exposed for 1, 4 or 24 h to 2450 MHz, CW, at 1, 5 or 10 W/kg SAR.

In another study, Sekijima et al. (2010) exposed three different cell lines (Human glioblastoma A172, neuroglioma H4 and lung fibroblast IMR-90 cells) to 2145 MHz, CW or W-CDMA, 0.08-0.8 W/kg SAR, for up to 96 h. Differential expression in a small number of genes was observed in each cell line. However, the results again were not validated by RT-PCR.

Le Quement et al. (2012) also reported no effects of 60.4 GHz millimetre waves (42.4 W/kg average SAR) given for 1, 6 or 24 h to primary human keratinocytes. Only few transcripts resulted to be affected by RF after PCR validation and the effect was transient (disappeared after 6 h).

In one investigation, the effect of RF exposure on protein expression of human breast cancer cells (MCF-7) was evaluated after RF exposure given 1h/day for 3 days at 849 MHz, at 837 MHz, CDMA, 2 or 10 W/kg SAR. No significant differences were recorded in exposed vs. sham exposed samples (Kim et al., 2010).

Table 9. ***In vitro*** **studies on effects of RF on gene and protein expression**

Reference	Cell type	Exposure conditions	Results
Nylund et al., 2009	Human endothelial cells (EA.hy926)	1800 MHz, GSM<br>2 W/kg; 1 h	Altered expression of several not identified proteins. No effect on HSP27 expression
Nylund et al., 2010	Human umbilical vein (HUVEC) and brain (HBMEC) endothelial cells	1800 MHz, GSM<br>2 W/kg; 1 h	Altered expression of several not identified proteins. No effect on HSP27 expression
Franzellitti et al., 2008	Human Trophoblasts (HTR-8/SV neo cells)	1800 MHz; GSM-217, GSM talk<br>2 W/kg; 4, 16, 24 h<br>(5 min on/10 min off)	No effect on HSP expression. Changes in one (HSP70C) over 4 transcript isoforms with different effect of GSM signals
Kim et al., 2012	Human breast epithelial cells (MCF10A)	837 MHz CDMA<br>4 W/kg; 4 h or 2 h on three consecutive days	No variation in the expression level of HSPs and MAPKs
Terro et al., 2012	Rat Cerebral cortical cells	900 MHz, GSM<br>0.25 W/kg; 24 h	No induction of apoptosis and protein degradation.<br>Increased expression of HSC70; decreased expression of HSP90
Gerner et al., 2010	Human leukaemic cells (Jurkat); Human fibroblasts (ES-1); mononuclear cells	1800 MHz, GSM<br>2 W/Kg; 8h<br>(5 min on/10 min off)	No effect on protein expression; Higher level of ^{35}S-incorparated proteins
Nicolaz et al., 2009	Human astrocytoma-derived cells (U-251)	59-61 GHz;<br>2.64-3.3 W/kg<br>24 h	No effects on endoplasmic reticulum stress-responsive chaperon proteins and HSP70
Sun et al., 2012	Human amniotic cells (FL)	1800 MHz, GSM<br>0.1 to 4 W/kg; 15 min	Increased EGF receptor clustering and phosphorylation from 0.5 to 4 W/kg
Yoon et al., 2011	Human dermal cells (hDPC)	1763 MHz, CDMA<br>2 or 10 W/kg<br>1-3 h	Increased IGF-1 expression, MAPK1 phosphorylation, BCL-2 and cyclin D1 expression; decreased BAX expression after 3h at 10 W/kg
Cervellati et al., 2009	Human Trophoblasts (HTR-8/SV neo cells)	1817 MHz, GSM 217<br>2 W/kg; 1 h	Increase in Cx40 and Cx43 gene expression.<br>No effect on proteins expression.<br>Change in proteins cellular localization.

Roux et al., 2010	Normal human keratinocytes	900 MHz CW<br>8 V/m (2.6 W/kg); 10 min<br>41 V/m (0.73 W/kg); 30 min	No significant expression modulation of about 47000 genes
Sakurai et al., 2011	Human-derived glial cells (SVGp12)	2450 MHz, CW<br>1, 5, 10 W/kg<br>1, 4, 24 h	Altered gene expression, not confirmed by RT-PCR
Sekijima et l., 2010	Human glioblastoma A172, neuroglioma H4 and lung fibroblast IMR-90 cells	2142.5 MHz (CW, W-CDMA)<br>0.08, 0.25 or 0.8 W/kg; 96 h	Altered expression of a small number of genes in each cell line
Le Quement et al., 2012	Primary human keratinocytes	60.4 GHz<br>average SAR<br>42.4 W/kg; 1,6, 24 h	No effect on gene expression.
Kim et al., 2010	Human breast cancer cells (MCF-7)	849 MHz CDMA<br>2 or 10 W/kg<br>1 h/day for 3 days	No effects on protein expression

EGF: Epidermal Growth Factor; HSC: Heat Shock cognate; HSP: Heat Shock proteins; IGF: Insuline-like Growth Factor; MAPK: mitogen activated protein kinase; RT-PCR: Real Time-Polymerase Chain Reaction.

Conclusions on *in vitro* studies

DNA damage has not been detected in a large number of *in vitro* studies, although DNA integrity was affected in some investigations. In some of these cases, the effect seemed to be dependent on the cell type investigated and by the electromagnetic parameters applied (frequency, modulation). Most of the studies reporting a lack of effects refer to chromosome aberration and micronuclei, which are indicators of fixed DNA damage, i.e., unrepairable damage, while most of the investigations reporting effects refer to DNA migration, spindle disturbances and foci formation, which are indicators of non-fixed DNA damage, i.e., transient and repairable damage. Concerning the other endpoints not related to genotoxicity, most of the studies did not find any effects. A few studies reported positive findings, which sometimes were reversible.

3.6.1.4. Conclusions on neoplastic diseases from RF exposure

Overall, the epidemiological studies on RF EMF exposure do not show an increased risk of brain tumours. Furthermore, they do not indicate an increased risk for other cancers of the head and neck region. Some studies raised questions regarding an increased risk of glioma and acoustic neuroma in heavy users of mobile phones. The results of cohort and incidence time trend studies do not support an increased risk for glioma while the possibility of an association with acoustic neuroma remains open. Epidemiological studies do not indicate increased risk for other malignant diseases including childhood cancer.

A considerable number of well-performed *in vivo* studies using a wide variety of animal models have been mostly negative in outcome. These studies are considered to provide strong evidence for the absence of an effect.

A large number of *in vitro* studies pertaining to genotoxic as well as non-genotoxic end-points have been published since the last Opinion. In most of the studies, no effects of exposure at permissible levels were recorded, although in some cases DNA strand breaks and spindle disturbances were observed.

3.6.2. Nervous system effects and neurobehavioural disorders

3.6.2.1. Epidemiological studies

What was already known on this subject?

The previous SCENIHR report concluded that there was no evidence that acute exposures to RF fields at the levels relevant for mobile telephony had effects on hearing or vision. Furthermore, there was is no evidence that this kind of exposure had direct

neurotoxicological effects. Most studies showed lack of effects on supporting structures like the blood-brain-barrier. The positive finding was lacking dose-response relationships and needed independent replication in studies with improved methodology.

What has been achieved since then?

Neurodevelopment and behavioural outcomes

To further elucidate earlier findings showing an association between mobile phone use and behavioural problems, an extension of the first analysis within the Danish Birth Cohort was conducted based on more than 28,000 children born in 1998-2002 (Divan 2012). Similar to the earlier report, a 25-item Strengths and Difficulties Questionnaire was used to assess behavioural problems (disruptive behaviour including temper tantrums and disobedience, with attention deficit hyperactivity disorder as the most common diagnosis) at age 7 years. Mobile phone use of the mother during pregnancy and child's own mobile phone use were assessed by interview when the child was aged seven. The findings were largely consistent with the earlier report, with slightly but significantly elevated risk of behavioural problems associated with both maternal and own mobile phone use. The adjusted odds ratio for mother's mobile phone use during pregnancy was 1.2 (95% CI 1.0-1.4), for child's own use 1.3 (1.1-1.5) and for both exposures combined 1.5 (1.3-1.7). Mobile phone exposure was associated with lower socioeconomic status, maternal smoking and mother's younger age as well as higher prenatal stress scores. Adjustment for these potential confounders weakened the association but did not remove it. The overall prevalence of behavioural problems was 3%, which is similar to reports from earlier studies (and suggests that the assessment method gives credible results).

The relation of maternal mobile phone use and child development was analysed in the Danish National Birth Cohort, with 41,000 singletons born in 1996-2002 (Divan et al. 2011). Information on mothers' mobile phone use during pregnancy was assessed retrospectively and child development was evaluated using telephone interviews at ages 6 and 18 months. No clear associations between mobile phone use and cognitive development (language skills) or motor development were observed (odds ratios 0.8-1.1 for mothers with 4 or more relative to 0-1 calls per day and mobile phone on all day versus not at all). The assessment of development was based on maternal reports instead of direct observation. In addition, mobile phone use was asked retrospectively.

A Dutch study on behavioural problems in relation to mobile phone exposure found no increases related to maternal mobile phone use during pregnancy (Guxens et al. 2013). The analysis was based on a birth cohort study of 2618 children and behavioural problems assessed using the Strengths and Difficulties Questionnaire at age 5 with both mothers and teachers as informants. The major weakness of the study was the fact that information on phone use during pregnancy was obtained retrospectively when the children were aged 7 years.

In a Spanish study of 530 children, neurodevelopment was assessed at age 14 months by psychologists using well-established instruments, and information on mothers' frequency of mobile phone use was collected with an interview during pregnancy (Vrijheid et al. 2010). No significant association was found between the number of daily calls and mental or psychomotor scores, although the average scores were slightly higher for mental and lower for psychomotor development even after adjustment for mother's education, IQ and smoking. A strength was the careful assessment of outcome, weakness scanty information on mobile phone use.

Interview data collected from parents of 2422 schoolchildren (age distribution not given) in 2008 and 2010 were used to analyse the relationship between mobile phone use, blood lead and ADHD (Byun et al., 2013). The data were analysed as two cross-sectional studies as well longitudinal analysis. Adjustment for age, gender, number of siblings, child's and parental psychiatric diagnoses and maternal smoking was used. ADHD was assessed using ADHD rating scale and prevalence of ADHD was 8-10%. Overall, children with mobile phones did not have increased prevalence of ADHD symptoms (OR 0.74-0.93

in three analyses), age at start of mobile phone use or cumulative call-time were not associated with ADHD symptoms either. Only use of mobile phone for playing games >3 minutes per day was associated with ADHD symptoms, and internet browsing with mobile phone in 2008 data but not in the other two analyses. Results for possible association between blood lead and ADHD symptoms were not given. Analyses of the interaction (joint effect) of blood lead level (dichotomized into below versus above 2.35 μg/dl) and mobile phone usage were also presented. Of the eight exposure indices reported, only age at first obtaining an own mobile phone was associated with ADHD symptoms, though no systematic pattern by age was observed though highest prevalences were reported for older ages. The results show little evidence for an association between mobile phone use and ADHD symptoms. The behavioural patterns in ADHD may however affect also mobile phone use and therefore interpretation of a relationship would need to be cautious.

In a cross-sectional survey conducted in Germany, a higher prevalence of conduct problems was found among children and adolescents with the highest RF exposure from mobile phones (Thomas 2010). The study population was recruited as a sample of the population aged 8-17 years in four Bavarian towns in 2006-2008, with 52% participation. Maschek exposimeter worn during one day (recording once per second, no measurements during night time) was used for exposure assessment. The exposure levels were low, with the highest measurements <1% of the ICNIRP reference level. The 25-item Strengths and Weaknesses Questionnaire was used to evaluate behavioural problems. It was filled in by the subjects themselves, with exception for children aged 8-12 where parents made the assessment. The prevalence of four categories of behavioural problems ranged from 3-7%. When the subjects were divided into deciles based on the electromagnetic field strength, those in the highest exposure category had a higher prevalence of conduct disorders (OR=3.7, 95% CI 1.6-8.4 for those teenagers and 2.9, 1.4-5.9 for those aged 8-12 years). The analysis used adjustment for age, sex, own or parental education, town.

Neurological disease

An analysis of the risk of multiple sclerosis in relation to mobile phone use was analysed in the Danish cohort study of 420,000 private mobile phone subscribers (Poulsen et al. 2012). The cohort was established from network operator records in 1982-1995 and followed up through 2004. During a 10-year follow-up, a total of 406 multiple sclerosis cases occurred among the subscribers with incidence comparable to the rest of the population (RR 1.06, 95% CI 0.96-1.18). No clear relation to duration of subscription was found, although the point estimate for 13 or more years was slightly above unity (RR 1.26, 95% CI 0.65-2.43).

Incidence of neurological disease has also been reported in the Danish cohort study (Schüz et al. 2009). The cases were defined as first hospital contacts (hospitalization or outpatient visit). The standardized hospitalisation rates (SHR) relative to the entire population were slightly increased for migraine and vertigo (SHR 1.1-1.2), but decreased for dementias and Parkinson's disease (SHR 0.7-0.8). Among men, lower rates of hospitalization were also seen for epilepsy. For migraine, vertigo and Parkinson's disease, no difference was observed any more after allowing for a 10-year latency. No difference in hospitalisations was found in amyotrophic lateral sclerosis or multiple sclerosis.

Discussion and conclusion on epidemiological studies

The large Danish National Birth Cohort study has reported results that suggest higher prevalence of some behavioural and health disorders in children, but these have not been confirmed in other studies. The published studies have methodological weaknesses including information on mobile phone use during pregnancy obtained only years after the birth of the child and concerns about residual confounding. A fundamental issue is whether the exposure indicators such as frequency of mother's mobile phone use are at all relevant for foetal RF exposure in utero. Attention deficit disorders have a clear hereditary component and hence it is possible that the findings could be due to reverse

causality, i.e. mother's mobile phone use reflecting her hyperactive features rather than phone use causing child's behavioural problems. In conclusion, there is weak evidence for an association between behavioural disorders and RF exposure of the foetus, because of conflicting results and methodological limitations.

Recent epidemiological studies have not shown increased risks of neurological disease related to RF exposure.

3.6.2.2. Neurophysiological studies

What was already known on this subject?

SCENIHR concluded in the previous Opinion that, with the exception of a few findings on otherwise negative studies, there is no evidence that acute or long-term RF exposure at SAR levels relevant for mobile telephony can influence cognitive functions in humans or animals. There is some evidence that RF exposure influences brain activity as seen by EEG studies in humans. Human studies also indicate the possibility of effects on sleep and sleep EEG parameters. However, certain findings are contradictory and are furthermore not substantiated by cellular studies into mechanisms. There is a need for further studies into mechanisms that can explain possible effects on sleep and EEG.

There is no evidence that acute exposures to RF-EMF fields at SAR levels relevant for mobile telephony have effects on hearing or vision. The positive finding is lacking dose-response relationships and needs independent replication in studies with improved methodology. The findings of activated glial cells at relatively high SAR-values could indicate gliosis and thus subsequent neurodegeneration after exposure, although exposures at lower levels did not reveal any such effects.

What has been achieved since then?

A number of studies on human volunteers as well as on various animal species (section 3.6.2.3) have been published since the previous Opinion. The experimental human studies focus on the macrostructure (sleep variables derived from polysomnography) and microstructure (EEG power) of sleep, other electrophysiological measurements (resting-stake waking EEG and event related potentials), brain activity assessed by functional magnetic resonance tomography (fMRI), cortical excitability assessed by transcranial magnetic stimulation (TMS), behaviour and cognition, sensory related functions, and other physiological outcomes, e.g. heart rate variability, skin conductance, blood flow and oxygenation parameters. Exposures have mostly been to GSM-related signals and UMTS-signals, one study each considered LTE and TETRA exposure.

Human studies - sleep:

Studies on possible effects of electromagnetic fields on the central nervous system (CNS) can be distinguished into those which focus on a resting and those which focus on an active brain. In the former case a further distinction can be made between a state in which exogenous factors can largely be neglected (sleep) and one in which the brain is awake but relaxed (usually waking EEG with eyes closed). Studies investigating a possible impact on the active brain among others comprise endpoints like event related potentials and cognitive performance. With regard to sleep it has to be distinguished between studies, which assess sleep at a physiological basis, i.e. based on sleep EEG, and those which rely on subjectively reported sleep quality. The latter assessments can deviate substantially from EEG based indicators of sleep quality. Studies referring to subjectively assessed sleep quality are discussed separately in the section symptoms (see 3.6.3).

Since the last Opinion eight studies covering EEG-based macrostructure of sleep as primary or secondary endpoint (see Table 10) and six studies on EEG-power during sleep (see Table 11) have been published. In a double-blind, randomized, sham-controlled cross-over study, Danker-Hopfe et al. (2011) investigated whether a GSM (900 MHz, pulsed with 217 Hz) and/or a UMTS (1966 MHz) exposure applied by a specially developed antenna (Bahr et al. 2006, 2007) for 8h during time in bed has an effect on

the macrostructure of sleep. A cell-phone usage at maximum RF output power was simulated and the transmitted power was adjusted in order to approach, but not to exceed a SAR_{10g} of 2 W/kg. To avoid electromagnetic interference with the recording device additional filters and a shielding were applied. The sample comprised 30 healthy males (age range 18 – 30 years). In order not to miss any possible effect 177 variables characterizing the initiation and maintenance of sleep were investigated. In the GSM exposure condition six REM sleep related variables indicated significantly more REM sleep as compared to sham, while four NREM stage 2 related variables showed a statistically significant decrease. The number of stage shifts from slow wave sleep to the light NREM stage 1 sleep was lower in the exposure condition and movement time was slightly higher. In the UMTS exposure condition only three sleep variables showed a statistically significant effect. The duration of the REM sleep period was longer while the one for NREM sleep was shorter. Furthermore, there was less NREM stage 2 sleep in the middle of sleep cycles. Although for GSM the number of statistically significant variables exceeds those expected by chance at the 5% significance level (9) the results do not indicate a negative impact of RF exposure on sleep macrostructure.

In a second study by this group, a possible effect of EMF exposure from mobile phone base stations on the sleep of residents (< 500 m distance from base station) was investigated in an experimental field study (Danker-Hopfe et al. 2010, see Table 10). Whole night exposure comparable to real-world scenarios for the general public living in areas with mobile phone service was realized by an experimental mobile phone base station, originally used for disaster recovery, containing GSM 900 MHz and GSM 1800 MHz base transceiver stations, a mast, cables, antennas and a power supply system. The sum signal simulated a base station transmitting near full capacity. For more than 90% of the study participants the field strength resulting from the experimental base station was between 0.01-0.9 V/m. The seven EEG-based sleep parameters obtained from 335 subjects (mean age ± SD: 45.0 ± 14.2 years; range 18-81 years) did not differ between sham and exposure nights. This study also analysed subjective sleep quality (see 3.8.3).

Lowden et al. (2011) studied possible effects of RF EMF exposure prior to sleep (duration: 3 h). They used a double-blind exposure to either an 884 MHz GSM signalling standard including the low frequency amplitude modulation components of an uplink GSM signal: 2, 8, 217 and 1736 Hz with a 10 g peak spatial-averaged SAR of 1.4 W/kg or sham. The sample comprised 48 subjects (23 with mobile phone-related symptoms and 25 without symptoms, overall 27 females, age range 18-44 years). An ANOVA revealed that there were no differences between the sensitive and the non-sensitive group and also a lack of a significant group*exposure interaction, hence the groups were pooled for further analyses. The results of full night polysomnography (7h) revealed that under exposure slow wave sleep was significantly decreased (and this was mainly due to a reduction of NREM stage 4), while the latency to NREM stage 3 and the amount of NREM stage 2 (min) were significantly increased (see Table 10).

In a study aimed at analysing possible mechanisms by which RF EMF could affect cortical excitability during sleep and sleep dependent performance changes in memory, Lustenberger et al. (2013) also looked at changes in the macrostructure resulting from an all-night exposure (see Table 10). The sample consists of 16 healthy males in the age range 18-21 years. Subjects' head was exposed using a circular-polarized antenna facing down to the subject's forehead. They used a 900 MHz signal pulsed with 7 consecutive 7.1 ms pulses forming one 500 ms burst. "These 500 ms bursts were repeated every 4 s (Intermittent-1 phase, 0.25 Hz, corresponding approximately to occurrence of sleep spindles), and every 1.25 s (Intermittent-2 phase, 0.8 Hz, corresponding approximately to frequency of slow oscillations) respectively. Exposure of 5 min Intermittent-1 was followed by 1 min with no exposure (OFF phase=, then 5 min Intermittent-2 was followed by a 7 min OFF phase. This 18 min sequence was repeated throughout the night. The peak spatial specific absorption rate averaged over any 10g tissue (psSAR10 g) during the 7.1 ms pulses was set to 10W/kg. This resulted in a burst average of 1.0 W/Kg. The whole night $psSAR_{10\ g}$ averaged to 0.15 W/kg." (Lustenberger et al., page 2). The exposed subjects showed a reduced total sleep time (p = 0.04) and consequently a

reduced sleep efficiency ($p = 0.04$). This was mainly due to increase of wake after sleep onset ($p = 0.03$), while NREM and REM sleep duration was not affected.

Nakatani-Enomoto et al. (2013) investigated possible effects of a 3 h W-CDMA exposure starting 5 h prior to sleep on subjective perception of sleep quality, macrostructure of and the EEG power spectrum during sleep. Their sample comprised 19 subjects (12 males and 7 females) aged 22-39 years. Assessments were made on three consecutive nights (adaptation, sham and real exposure). Exposure was applied in a double-blind, counterbalanced design in three 1h-blocks separated by 5 min breaks, in which batteries were replaced. A not further specified phone controlled by a base station generator was used for exposure. Peak spatial SAR_{10g} values were calculated for a Japanese human head model resulting in 1.52 W/kg for the head and 0.13 W/kg for the brain. The location of the maximum was not specified. In this study none of the investigated endpoints: sleep stages and derived variables, EEG power spectra (calculated for six EEG derivations and analysed for frequency bands nor narrow frequency bids) of stages wake, stage NREM2 and spindles as well as subjective ratings on sleep quality was affected.

Table 10. RF-EMF effects and macrostructure of sleep

Authors	Signal type	Exposure side [1)] ; Antenna [2)] ; Design [3)]	Sample	Exposure Duration (ED); EEG/Exposure [4)]; EEG electrodes (E)	Changes with exposure
Danker-Hopfe et al. (2010)	GSM 900 + GSM 1800: generic GSM signals, 2 channels per sector 8/8 slots + 1 channel 6/8 slots (each for 900 + 1800) (modified base station)	S: n.a.<br>A: bs<br>D: db, co	335 subjects (162f / 173m); 18-81 years	ED: 8 hours / night 5 nights with and 5 nights without exposure;<br>EEG/E: 2;<br>E: frontopolar	No effect of exposure on:<br>Sleep efficiency index, stage NREM1 latency, stage NREM2 latency, wake after sleep onset (min and % time in bed), total sleep time and time in bed
Danker-Hopfe et al. (2011)	1) 900 MHz: pulse mod. 217 Hz, width 533 µs<br>2) WCDMA: 1966 MHz, QPSK, incl. power control & fading simulation<br>SAR_{10g} = 2 W/kg	S: R<br>A: s<br>D: db, co	30 males; 18-30 years	ED: 8 hours during bed time;<br>EEG/E: 3;<br>E: 19 + M1 + M2	GSM exposure: 13 significant variables out of 177 variables analysed; increased stage REM sleep (6 variables), reduced NREM stage 2 sleep (4 var.), increased movement time (2 var.), less stage shifts from SWS to NREM stage 1<br>WCDMA exposure: 3 significant variables out of 177 variables analysed; duration of REM periods increased, duration of NREM periods decreased, less NREM stage 2 in the middle sleep cycles
Lowden et al. (2011)	GSM 884 MHz test signal incl. DTX (periods of 5s mean duration, SAR reduced to 12%) and non-DTX (11s mean dur.), modulation components: 2, 8, 217, 1736 Hz;<br>SAR_{10g} = 1.4 W/kg	S: L<br>A: s/l<br>D: db, co	48 (27f / 21m) 23 with and 25 without mobile phone attributed symptoms; 18-44 years	ED: 3 hours, prior to sleep, till 1 h before lights off;<br>EEG/E: 1;<br>E: 8 bipolar signals	No group*exposure effect<br>Total sample:<br>Increase in stage NREM2 sleep and decrease in stage NREM4 sleep (min) & slow wave sleep (SWS); increase in stage NREM3 latency;<br>No effects: total sleep time (TST) wake, stage NREM1, stage NREM3, and stage REM (all min), latencies to stage NREM1, NREM2, stage REM, sleep efficiency index, arousals/h TST;
Loughran et al. (2012)	894.6 MHz: carrier, pulse mod. 217 Hz, width 576 [µs]; (mp controlled by manufacturer software)<br>SAR_{10g} = 0.674 W/kg (hemispheric mean: 0.11 W/kg)	S: R<br>A: mp<br>D: db, co	20 (13f / 7m); 20-51 years	ED: 30 min, prior to sleep, till 20 min before lights off;<br>EEG/E: 1;<br>E: C3-A2, C4-A1	No effect: total sleep time sleep latency, REM sleep latency, arousal index/h, sleep efficiency index
Schmid et al (2012a)	1) 900 MHz, pulse mod. 14 Hz, width 2.3 ms (crest factor 31)<br>2) 900 MHz, pulse mod. 217 Hz, width 0.577 ms (crest factor 8); both active cond.: SAR_{10g} = 2 W/kg	S: L<br>A: l<br>D: db, co	30 males; 20-26 years	ED: 30 min, prior to sleep, till 10 min before lights off;<br>EEG/E: 1;<br>E: C3-A2	No effect: total sleep time, sleep latency, stage REM latency, wake after sleep onset, stage NREM2 sleep, slow wave sleep, stage REM sleep , and movement time(all min), sleep efficiency index; NREM sleep, REM sleep and NREM stage 2 sleep in cycles 1, 2, 3, and 4
Schmid et al. (2012b)	magnetic field (MF) or 900 MHz RF (amplitude modulated); MF or modulation: 2 Hz, 8 Hz and harmonics up to 20 Hz;<br>RF: SAR_{10g} = 2 W/kg;<br>MF: 0.7 mT	S:<br>MF: LR<br>RF: L<br>A: l<br>D: db, co	25 males; 20-26 years	ED: 30 min, prior to sleep, till 10 min before lights off;<br>EEG/E: 1;<br>E: C3-A2	RF exposure: less stage REM sleep in the second sleep cycle;<br>No effect of RF and MF: total sleep time, sleep latency, stage REM latency, wake after sleep onset, stage NREM2 sleep, slow wave sleep, stage REM sleep, and movement time (all min), sleep efficiency index; NREM sleep and NREM stage 2 sleep in cycles 1, 2, 3, and 4; REM sleep in cycles 2, 3, and 4
Lusten-berger et al. (2013)	900 MHz: carrier, 500 ms bursts: 7 pulses of 7.1 ms each; exposure sequence (repeated whole night):<br>5 min 1 burst every 4 s<br>1 min off<br>5 min 1 burst every 1.25 s<br>7 min off<br>SAR_{10g} = 10 W/kg (pulse)<br>SAR_{10g} = 1 W/kg (for burst)<br>SAR_{10g} = 0.15 W/kg (average for sequence or whole night)	S: n.a.<br>A: l<br>D: db, co	16 males; 19.9 ± 0.2 years	ED: whole night;<br>EEG/E: 2;<br>E: C4-A1	RF exposure: decrease of total sleep time (p = 0.04), reduced sleep efficiency (p = 0.04), increase of wake after sleep onset (p = 0.03).<br>RF exposure: reduced (p = 0.03) sleep-dependent performance improvement in a motor-tapping task.
Nakatani-Enomoto et al. (2013)	WCDMA 1950 MHz;<br>SAR_{10g} = 1.52 W/kg (head)<br>SAR_{10g} = 0.13 W/kg (brain)	S: L<br>A: mp<br>D: db, co	19 subjects; 22-39 years<br>12 males; 30.1 ± 5.9 years<br>7 females; 32.4 ± 6.5 years	ED: 3 h starting 5 h prior to sleep<br>EEG/E: 1<br>E: F3-A2, F4-A1, C3-A2, C4-A1, O1-A2, O2-A1	No effect on sleep latency, REM sleep latency, sleep efficiency index, stages W, N1, N2, N3 and R (%), stage N2 latency

1) L = left, R = right, LR = both sides; n.a. = not applicable
2) mp = mobile phone, s = similar to mobile phone, l = larger head area, bs = base station
3) db = double-blind, sb = single-blind, co = cross-over, pg = parallel group
4) 1 = not simultaneously; 2 = simultaneously without or without information on electromagnetic interference tests, 3 = simultaneously with information on electromagnetic interference tests

In a study, which aimed at analysing the effect of pulse modulation on sleep EEG power Schmid et al. (2012a) used a GSM 900 MHz signal pulsed with 217 Hz and 14 Hz. The 14 Hz signal was selected since this is in the EEG frequency range (11-15 Hz) where previous studies have shown a significant effect of pulsed exposure. They used a double-blind randomized three-way cross-over design (exposure conditions: GSM 900 MHz pulsed with 217 Hz, GSM 900 MHz pulsed with 14 Hz and sham; active conditions: peak spatial SAR_{10g} 2 W/kg). Schmid et al. (2012a) did not find differences in the macrostructure of sleep following a 30 min exposure prior to sleep (see Table 10). The results are based on data from 30 young healthy men (20 - 26 years). EEG power in the spindle frequency range was increased during NREM sleep in the second sleep episode following the 14 Hz pulse modulation. For the 217 Hz pulse-modulated condition the increase was not statistically significant (see Table 11). The observation that pulse modulated RF-EMF alters brain physiology is consistent with previous research. The authors underline the considerable individual variability.

Schmid et al. (2012b) investigated the effect of a 2 Hz pulse modulation of an RF EMF exposure on sleep EEG and whether the same effects occur after magnetic field exposure with the same 2 Hz pulse sequence. The sample comprised 25 healthy young males (20 to 26 years) of which two had to be excluded due to bad signal quality or long periods of wakefulness. Exposure was delivered for a duration of 30 min prior to sleep in a three way cross-over double-blind design. For both the amplitude modulation of the 900 MHz carrier and the time course of the magnetic field a low frequency signal containing components up to 20 Hz was used. These components (2 Hz, 8 Hz and harmonics) had higher amplitudes compared to those in GSM uplink signals. For 900 MHz the peak spatial SAR_{10g} was 2 W/kg. The amplitude (temporal peak value) of the magnetic field was 0.7 mT in the brain. This corresponds to 86% of the ICNIRP limit. ELF magnetic fields from mobile phones are weaker. Neither of the exposure conditions had a significant effect on sleep macrostructure as compared to sham except for a reduced amount of REM sleep in the second sleep cycle under RF exposure (see Table 10). A statistically significant increase in EEG power in the spindle frequency range (13.75 – 15.25 Hz) was only seen following RF exposure in NREM sleep and in NREM stage 2 sleep for the whole night, the first, third and fourth sleep cycle. Additionally, for both exposure conditions increased spectral power was observed for NREM sleep as well as for NREM stage 2 sleep for frequencies in the delta and theta frequency ranges (1.25 – 9.0 Hz). With regard to sleep cycles the differences occurred in cycles 3 and 4 of the night. The REM sleep EEG showed an increased power in the alpha range frequencies (7.75 – 12.25 Hz) following RF exposure only and in the lower delta range (0.75 – 1.5 Hz) in both exposure conditions (see Table 11). The authors concluded that both the pulse-modulated RF field and the pulsed magnetic field affect brain physiology; with higher frequency pulse modulation components not being necessary for the effect to occur. Furthermore, the results do not support the hypothesis that previously observed effects of RF fields are based on demodulation of the signal only.

In the study by Lowden et al. (2011), sample size for power spectra analyses was reduced from 48 to 32 due to artefacts. They observed an increased power after exposure in the frequency ranges 0.5 – 1.5 Hz and 5.75 – 10.5 Hz during the first 30 min of NREM stage 2, an increased power for 7.5 – 11.75 Hz in the first hour of NREM stage 2 sleep and finally in the 4.75 – 8.25 Hz bands in the second hour of NREM stage 2 sleep. The corresponding figure shows that for the second and third hour of NREM stage 2 sleep there were also single statistically significant results for lower and higher frequency bands. There were no differences between subjects with and without mobile phone attributed symptoms.

Table 11. RF-EMF effects and sleep EEG power

Authors	Signal type	Exposure side [1]; Antenna [2]; Design [3]	Sample	Exposure Duration (ED); EEG/Exposure [4]; EEG electrodes (E)	Changes with exposure
Lowden et al. (2011)	GSM 884 MHz test signal incl. DTX (periods of 5 s mean duration, SAR reduced to 12%) and non-DTX (periods of 11s mean dur.) SAR_{10g} = 1.4 W/kg	S: L<br>A: s/l<br>D: db, co	32<br>14 with and 18 without mobile phone attributed symptoms;<br>18-44 years	ED: 3 hours, prior to sleep, till 1 h before lights off;<br>EEG/E: 1;<br>E: 8 bipolar signals3 hours	No effect first 30 min and first h of slow wave sleep;<br>First 30 min stage NREM 2 sleep: EEG power increase 0.5-1.5 Hz, 5.75-10.5 Hz;<br>First 60 min stage NREM2 sleep: EEG power increase 7.5-11.75 Hz;<br>2nd h of stage NREM2: 4.75-8.25 Hz; 3rd h of stage NREM2 sleep: sporadic elevated single 0.25 Hz bands.
Schmid et al (2012a)	1) 900 MHz, pulse mod. 14 Hz, width 2.3 ms (crest factor 31)<br><br>2) 900 MHz, pulse mod. 217 Hz, width 0.577 ms (crest factor 8);<br>both active cond.:<br>SAR_{10g} = 2 W/kg	S: L<br>A: l<br>D: db, co	30 males;<br>20-26 years	ED: 30 min, prior to sleep, till 10 min before lights off;<br>EEG/E: 1;<br>E: C3-A2	14 Hz pulse: Increased power:<br>during NREM in the 2nd sleep episode (spindle frequency range); post hoc: 2nd sleep episode NREM: 12.75-13.25 Hz; 2nd sleep episode NREM stage 2: 11.25, 12.75-13 Hz;<br><br>217 Hz: No significant effects
Schmid et al. (2012b)	magnetic field (MF) or 900 MHz RF (amplitude modulated); MF or modulation: 2 Hz, 8 Hz and harmonics up to 20 Hz;<br>RF: SAR_{10g} = 2 W/kg;<br>MF: 0.7 mT (amplitude, temporal peak) nearly all over the brain	S: L<br>MF: LR<br>RF: R<br><br>A: l<br>D: db, co	25 males;<br>20-26 years	ED: 30 min, prior to sleep, till 10 min before lights off;<br>EEG/E: 1;<br>E: C3-A2	RF:<br>EEG power increase in the spindle frequency range (13.75-15.25 Hz) for NREM and NREM stage 2 sleep for the whole night, the 1st, 3rd and 4th sleep cycles;<br>EEG power increase in alpha range frequencies (7.75-12.25 Hz) for REM sleep;<br><br>RF and MF:<br>EEG power increase in the delta and theta frequency ranges (1.25-9 Hz) for NREM and NREM stage 2 sleep for the whole night, the differences occurred in the 3rd and 4th sleep cycles;<br>EEG power increase in lower the delta range frequencies (0.75-1.5 Hz) for REM sleep;
Loughran et al. (2012)	894.6 MHz: carrier, pulse mod. 217 Hz, width 576 [μs];<br>(mp controlled by manufacturer software)<br>SAR_{10g} = 0.674 W/kg (hemispheric mean: 0.11 W/kg)	S: R<br>A: mp<br>D: db, co	20<br>(13f / 7m);<br>20-51 years	ED: 30 min, prior to sleep, till 20 min before lights off;<br>EEG/E: 1;<br>E: C3-A2, C4-A1	1st 30 min of 1st NREM sleep episode: increased power: 11.5-12.25 Hz, females more affected than males;<br>no changes in 12.25-13.5 Hz and 13.5-14 Hz frequency ranges (where previously effects were observed)
Lusten-berger et al. (2013)	900 MHz: carrier, 500 ms bursts: 7 pulses of 7.1 ms each; exposure sequence (repeated whole night):<br>5 min 1 burst every 4 s<br>1 min off<br>5 min 1 burst every 1.25 s<br>7 min off<br>SAR_{10g} = 10 W/kg (pulse)<br>SAR_{10g} = 1 W/kg (for burst)<br>SAR_{10g} = 0.15 W/kg (average for sequence or whole night)	S: n.a.<br>A: l<br>D: db, co	16 males;<br>19.9 ± 0.2 years	ED: whole night;<br>EEG/E: 2;<br>E: C4-A1	RF Exposure: statistically significant ($p < 0.05$) increase in NREM power due to exposure alone: 8.5 Hz, statistically significant ($p < 0.05$) interaction effect for 13 out of 38 frequency bands (width 0.25 Hz) up to 10 Hz, and for 5 out of 16 frequency bands (width 0.25 Hz) in the slow wave activity range up to 4.5 Hz.
Nakatani-Enomoto et al. (2013)	WCDMA 1950 MHz;<br>SAR_{10g} = 1.52 W/kg (head)<br>SAR_{10g} = 0.13 W/kg (brain)	S: L<br>A: mp<br>D: db, co	19 subjects;<br>22-39 years<br><br>12 males;<br>30.1 ± 5.9 years<br><br>7 females;<br>32.4 ± 6.5 years	ED: 3 h starting 5 h prior to sleep<br>EEG/E: 1<br>E: F3-A2, F4-A1, C3-A2, C4-A1, O1-A2, O2-A1	No effect on EEG-power in frequency bands (delta, theta, alpha and beta) in stages W, N1, N2, N3 and R (during sleep period time) at F3, F4, C3, C4, O1 and O2<br><br>No effects on other microstructures of sleep: number of arousal; number of spindles in the first 30 min of NREM sleep

1) L = left, R = right, LR = both sides; n.a. = not applicable
2) mp = mobile phone, s = similar to mobile phone, l = larger head area, bs = base station
3) db = double-blind, sb = single-blind, co = cross-over, pg = parallel group
4) 1 = not simultaneously; 2 = simultaneously without or without information on electromagnetic interference tests, 3 = simultaneously with information on electromagnetic interference tests

In an attempt to investigate individual differences in effects of mobile phone exposure, Loughran et al. (2012) retested 20 healthy subjects (13 females, 27.9 ± 6.5 years) who participated in an earlier study (Loughran et al. 2005) with altogether 50 subjects. As in the previous study a double-blind, counterbalanced cross-over design was used to investigate a possible effect of a 30 min GSM exposure (894.6 MHz, pulsed with 217 Hz;

hemispheric mean SAR10g = 0.11 W/kg, peak SAR10g = 0.674 W/kg) prior to sleep. Based on the results of the previous study participants were divided into "increasers" (n=8) and "decreasers" (n=12) according to an increase or decrease of spectral power of the NREM sleep EEG in the 11.5-12.25 Hz frequency range. Overall verum exposure was associated with a significant ($p = 0.046$) increase in power in the 11.5 -12.25 Hz frequency range in the first 30 min of NREM sleep. This effect was more pronounced in the "increasers" than in the "decreasers" ($p = 0.038$). No other significant changes were observed in frequency ranges, which were previously reported to be affected (12.25 – 13.5 Hz and 13.5 – 14 Hz). Furthermore, females were more affected than males ($p = 0.035$) in this study. The authors claim that their results underline EEG effects to be sensitive to individual variability and that previous negative results are not strong evidence for a lack of an effect. Macrostructure of sleep was not affected in this study (see Table 10).

In the study by Lustenberger et al. (2013) described with regard to exposure in more detail above, eight EEG channels were recorded (F3, F4, C3, C4, P3, P4, O1 and O2) which were referenced to the contralateral mastoid. The sample consists of 16 healthy males in the age range of 18-21 years. Spectral power was computed for C4A1 for the first 4 NREM and REM episodes. An increase in spectral power for frequencies up to 10 Hz was seen during NREM sleep episodes. Spindle frequency ranges and REM sleep were not affected. Exposure as a factor showed a statistically significant ($p < 0.05$) effect in just one frequency band (centred at 8.5 Hz), while the interaction between exposure and sleep episode was significant ($p < 0.05$) in 13 of 38 frequency bands (width 0.25 Hz) considered up to 10 Hz, and for 5 of the 16 frequency bands (width 0.25 Hz) up to 4.5 Hz. A more detailed analysis of slow wave activity (SWA) which was calculated as spectral power between 0.75 and 4.5 Hz, revealed that in contrast to the usual decline of SWA during the night, there was a statistically significant deviation in SWA in the 4th NREM episode ($p < 0.05$), indicating a less pronounced SWA decrease under exposure (Table 2). Additionally, for NREM episode 4 two parameters based on the time-course of short time spectra were calculated: event-related spectra power (ERSP) and inter-trial coherence (ITC). They were time-locked to either the real EMF pulses or to corresponding times during sham. Under exposure an increased ERSP and ITC changes were observed.

The study by Lustenberger et al. (2013) for the first time looked at a possible RF EMF effect of sleep related performance improvement. They found a statistically significant ($p = 0.03$) reduced sleep-related performance improvement as assessed by the variance of performance in a motor sequence tapping (Table 10).

Human studies – Resting state waking EEG

Table 12. RF-EMF effects and waking EEG (resting state and related to cognitive tasks)

Authors	*Signal type*	*Exposure side [1]; Antenna [2]; Design [3]*	*Sample*	*Exposure Duration (ED); EEG/Exposure [4]; EEG electrodes (E)*	*Changes with exposure*
Croft et al. (2010)	GSM: 894.6 MHz, pulse mod. 217 Hz, duty cycle 0.125 (mp set via manufacturer software), SAR_{10g} = 0.7 W/kg;<br>WCDMA: 1900 MHz simulated WCDMA signal (external source), SAR_{10g} = 1.7 W/kg	S: LR counter-balanced<br>A: GSM: mp WCDMA: s<br>D: db, co	41: 13-15 years<br>42: 19-40 years<br>20: 55-70 years	ED: 51 min; EEG/E: 2; E: 61 scalp sites	GSM exposure only: Increased alpha power only in the 19-40 year old subjects
Vecchio et al. (2010)	GSM 902.4 MHz (incl. mod. components 217 Hz & 8.33 Hz) (mp set by a test card); SAR_{10g} = 0.5 W/kg	S: L<br>A: mp<br>D: db, co	16: 47-84 years<br>15: 20-37 years	ED: 45 min; EEG/E: 1; E: 19+M1+M2	Increased interhemispheric coherence of frontal alpha activity after exposure; statistically significant in the ederly and not in the younger subjects
Vecchio et al. (2012a)	GSM 902.4 MHz (incl. mod. components 217 Hz & 8.33 Hz) (mp set by a test card); SAR_{10g} = 0.5 W/kg	S: L<br>A: mp<br>D: db, co	10 patients with epilepsy and 15 age matched controls; 19-43 years	ED: 45 min; EEG/E: 1; E: 19+M1+M2	Increased interhemisperic coherence of temporal and frontal alpha rhythms after exposure in patients as compared to controls
Vecchio et al. (2012b)	GSM 902.4 MHz (incl. mod. components 217 Hz & 8.33 Hz) (mp set by a test card); SAR_{10g} = 0.5 W/kg	S: L<br>A: mp<br>D: db, co	11; 24-63 years	ED: 45 min; EEG/E: 1; E: 56 sites	Go/no-go task: alpha event-related desynchronisation (ERD): High-frequency alpha band: significantly lower amplitude change (ERD) after exposure as compared to pre exposure in the GSM condition, no effect for sham condition; Not effect for the low-frequency alpha band
Trunk et al (2013)	WCDMA 1947 MHz (mp, controlled by service software + RF amplifier); SAR_{1g} < 1.75 W/kg	S: R<br>A: s<br>D: db, co	17 (9f / 8m); 21.8 ± 3.5 years	ED: 30 min; EEG/E: 1; E: Fz, Cz, Pz	No exposure effect on spectral power for 6 frequency bands: delta, theta,alpha I, alpha II, beta I, and beta II)
Loughran et al. (2013)	900 MHz carrier, GSM mobile phone like modulation, Sham or SAR_{10g} = 0.35 W/kg or SAR_{10g} = 1.4 W/kg	S: L<br>A: l<br>D: db, co	22 adolescents (10f / 12m) 11 – 13 years two of them had to be excluded for EEG analysis	ED: 30 min; EEG/E: 1; E: C3, C4, O1, O2, M1+M2	No clear exposure effects on power spectra of the waking EEG
Perentos et al. (2013)	GSM900, 4 different exposure conditions:<br>1.Sham<br>2.RF continous wave<br>3.RF DTX-like pulsing<br>4.DTX-like ELF magnetic field<br>All exposure conditions were applied consecutively in a randomized order in one 2h session	S: R<br>A: s<br>D: db, co	72 (35 f / 3 m) 24.5 ± 5.4 years	ED: 20 min EEG/E: 3 E: 19 electrodes (cap 10/20 system), reference: M1	Outcome parameter: alpha power; During pulsed RF exposure: significant reduction of alpha-activity as compared to sham but not different from continous wave signal;<br>No effect of DTX-like ELF exposure<br>There were no paiwise differences between exposure conditions in the alpha power of the 5 min post exposure periods
Suhhova et al. (2013)	450 MHz carrier frequency, 40 Hz rectangular modulation; duty cycle 50%; quarter-wave antenna at 10 cm distance<br>SAR_{1g} = 0.303 W/kg or SAR_{1g} = 0.003 W/kg	S: L<br>A: l<br>D: sb, co	15 (6f / 9m) 32 – 32 years	ED: 10 min EEG/E: 3 E: 19 electrodes recorded, 8 considered for analysis: Fp1, Fp2, T3, T4, P3, P4, O1, O2, reference Cz.	Significant increase in power of the alpha, beta1 and beta2 EEG frequency bands for the higher exposure level, and beta2 for the lower exposure level.

1) L = left, R = right, LR = both sides; n.a. = not applicable
2) mp = mobile phone, s = similar to mobile phone, l = larger head area, bs = base station
3) db = double-blind, sb = single-blind, co = cross-over, pg = parallel group
4) 1 = not simultaneously; 2 = simultaneously without or without information on electromagnetic interference tests, 3 = simultaneously with information on electromagnetic interference tests

The literature has described effects of exposure to electromagnetic fields on EEG power not only for sleep but also for waking EEG. Here, the alpha frequency band (the basic rhythm of the resting EEG in approximately 85% of the population) seems to be affected. Many older studies must be criticized for methodological reasons (one reason being a single-blind exposure design), and recent studies are in some cases contradictory. Since the last Opinion seven studies on RF effects on resting state waking EEG were published (Table 12).

Croft et al. (2010) investigated age-related exposure effects on EEGs (eyes open) in the alpha band for GSM (894.6 MHZ, pulse modulated at 217 Hz; peak spatial SAR_{10g} = 0.7 W/kg) and UMTS (1900 MHz; peak spatial SAR^{10g} = 1.7 W/kg) in adolescents (13 – 15 years, n=41), young adults (19 – 40 years, n=42) and the elderly (55 – 70 years, n=20). Within each group of subjects they used a double-blind, counterbalanced, cross-over design. Effects were analysed for frontal and posterior electrodes. Results showed an increased alpha power only in the GSM exposure condition and here only for young adults.

A study by Vecchio et al. (2010) analysed age-dependent EMF effects on alpha activity in waking EEGs in 16 older (47-84 years) and 15 younger subjects (20-37 years). Participants were exposed to a GSM signal (902.40 MHz, modulation frequencies: 8.33 and 217 Hz) for 45 min with a maximum SAR of 0.5 W/kg emitted by a commercially available mobile phone which was set using a test card in a double-blind cross-over paradigm. EEG was recorded for 5 min prior to and following exposure at 19 electrodes. The authors found an increased inter-hemispheric coherence of frontal alpha EEG activity after GSM exposure which was statistically significant for the elderly subjects but not for the young ones. This might point to a GSM-EMF related inter-hemispheric synchronization of alpha rhythms as a function of physiological aging.

Vecchio et al. (2012a) used the same study design to investigate an exposure effect in patients with epilepsy. Data from 10 patients were compared to results from 15 age-matched controls from previous studies. Patients showed a statistically significant higher inter-hemispheric coherence of temporal and frontal alpha-rhythms under exposure as compared to control subjects. According to the authors, these results might indicate a GSM exposure effect on inter-hemispheric synchronization of the dominant (alpha) EEG rhythms in epileptic patients.

The effects of a 30 min UMTS mobile-phone like (1947 MHz with SAR1g slightly less than 1.75 W/kg) exposure was investigated in a randomized double-blind cross-over study by Trunk et al. (2013) in 17 young subjects (9 females, 21.8 ± 3.5 years). EEG was recorded at 3 sites 10 min prior and 10 min following exposure (sham and UMTS), while the subjects were watching a silent documentary. Repeated measures ANOVAs were conducted for the mean log-transformed spectral power for 6 frequency bands (delta, theta, alpha I, alpha II, beta I and beta II). None of the frequency bands showed a statistically significant exposure effect (see Table 12). Furthermore in a second experiment performed at another test session event-related potentials (ERPs) and mismatch negativity (MMN) were investigated. There was no effect on amplitude and latency of the auditory ERP components (see Table 13).

Loughran et al. (2013) presented the results of a study on GSM 900 MHz mobile-phone like exposure on the waking EEG in 22 adolescents (12 males) aged between 11 and 13 years (12.3 ± 0.8 years). Two of them had to be excluded from the EEG analyses due to high frequency noise in the signal. They applied three exposure conditions in a double-blind, randomized, and counter-balanced crossover design with a planar antenna at the left side of the participant's head: Sham, "low SAR" (psSAR 0.35 W/kg) and "high SAR" (psSAR 1.4 W/kg). EEG was recorded at C3, C4, O1 and O2 (referenced to the linked mastoids) prior to (baseline recording) and immediately as well as 30 and 60 minutes after an exposure session of 30 min duration. Time of day was kept constant within individuals. The authors summarize that there were no clear significant effects of exposure on the waking EEG. Moreover "results suggest that contrary to popular belief,

adolescents are not more sensitive to mobile phone emissions" (Loughran et al. 2013, p.1).

The impact of pulsing of GSM900-like RF signals on the resting EEG was investigated by Perentos et al. (2013) in 72 healthy subjects (35 females and 37 males, mean age 24.5 ± 5.4 years). In one single recording session participants received four 30 min intervals with 5 exposure free min at the beginning and at the end and 20 min of exposure: sham, continuous RF, DTX pulsed RF, and DTX pulsed low-frequency magnetic fields with 4 min breaks between exposure intervals. The order of exposures was counterbalanced and randomly assigned in a double-blind cross-over design. The EEG was recorded continuously during the 2-h session. The recording device was protected against RF interference by shielding and filters. Exposure was applied by a specially constructed handset mimicking the spatial exposure characteristics of GSM phones. Peak spatial SAR10g was measured in a phantom: 1.95 W/kg for the continuous wave condition. The DTX signal used the same amplitude resulting in much lower SAR values. The peak magnetic flux density was 25 μT at the surface of the handset. The authors exclusively looked at the alpha-band (8 – 12.75 Hz) and observed a significant decrease in the power spectra during pulse modulated RF exposure, which is not in line with previous observations indicating an increase in alpha power under RF exposure. Since the EEG spectral power with pulse modulated RF exposure was not different from continuous wave RF exposure the authors concluded that their results do not support the hypothesis that "pulsed" RF is required to produce EEG effects. DTX-like ELF magnetic exposure did not affect alpha power. No significant pairwise differences in alpha power between exposure conditions have been observed in the post exposure period.

Suhhova et al. (2013) investigated effects of a 450 MHz exposure modulated at 40 Hz on the resting state waking EEG with eyes closed in a sample of 15 subjects (6 females and 9 males, 23 – 32 years). Following a baseline assessment two randomly assigned exposure levels were considered in a single-blind design: psSAR1g = 0.303 W/kg (field strength 24.5 V/m) and SAR1g = 0.003 W/kg (field strength 2.45 V/m. This set-up (session) was repeated on the same day after a 15 min break with a reversed order of the two real exposures. Exposure was delivered by a signal generator, a modulator, an amplifier and a quarter-wave antenna located 10 cm from the left side of the head. Each exposure condition consisted of five 2 min cycles with 1 min exposure off and 1 min exposure on. The first 30s of the 1 min segments were used for calculating deviations between on/off in percent. The same segmentation was done for the reference condition. (Note: This methodological approach leads to an increase between on and off also in the reference condition amounting to a "change" of at least 10%). Results showed a statistically significant increase in the EEG power in three of four EEG frequency bands: beta2, beta1, and alpha frequency bands at the higher SAR level, and in the beta2 frequency band at the lower SAR level. In a phantom measurement artifacts from RF exposure were identified in the EEG signal. Although the disturbing 40 Hz component was removed from the EEG by filtering it is not clear whether parameter shifts or other interferences with the EEG recording system may account for at least a part of the observed differences.

Human studies - brain activity assessed by fMRI

There are two studies investigating RF-EMF effects on brain activity with functional magnetic resonance tomography (fMRI). One (LV et al. 2014) investigated resting state brain activity while the other (Curcio et al. 2012) investigated brain activity during a cognitive task.

Lv et al. (2014) used resting state BOLD fMRI signals to analyse possible effects of a 30-min RF-EMF exposure to a LTE signal (2.573 GHz) in a double-blind, cross-over, randomized and counter-balanced study. Assessments of spontaneous low frequency oscillations were made prior to and after a 30-min exposure. The sample comprised 18 subjects (6 females and 12 males) from 19 to 35 years of age (mean 24.9 ± 3.9 years). Subjects had test sessions on two consecutive days with the following assessments: structural MRI, pre-exposure resting state fMRI, RF-EMF exposure (real or sham) and

post-exposure resting state fMRI. Exposure was delivered by a signal generator, amplifier, and a dipole antenna located on the right hand side of the head. The delivered power of the antenna was measured and adjusted to produce a mean spatial peak SAR_{10g} of approximately 1W/kg. Outcome parameters were the ALFF, which indicates the absolute strength of spontaneous fluctuations within a specific frequency range (typically 0.01 – 0.1 Hz), and fALFF, which represents the relative contribution of this specific frequency range to the whole detectable frequency range (0 – 0.25 Hz). Both parameters were individually standardized to reduce variability between subjects. A comparison of pre-post differences did not reveal significant differences in the sham condition. In the real exposure condition three brain regions showed significantly decreased ALFF values (after correction of the alpha level for multiple testing): cluster 1 located in the junction of the left superior temporal gyrus and the left middle temporal gyrus, cluster 2 located in the posterior part of the right superior temporal gyrus and cluster 3 located in the junction of right medial frontal gyrus and the right paracentral lobule. fALFF was significantly reduced close to the junction of the medial frontal gyrus and the right paracentral lobule. No region showed an increased ALFF or fALFF after correction. The study provided other than EEG-based evidence that RF-EMF may modulate resting state neural activity. However, deviating from EEG, it has to be kept in mind that the MRI investigations involve stronger electromagnetic fields than the experimental ones.

Curcio et al. (2012) investigated whether GSM exposure affects the brain BOLD response in a somatosensory Go-NoGo task in 12 healthy young males between 19 and 25 years (mean ± SD: 21.4 ± 2.0 years). Female subjects were not included to avoid variation in the outcome parameters due to hormonal changes during the menstrual cycle. Paired electrical pulses separated by 150 ms (Go) and single pulses (No-Do) were presented on either the left or the right hand in a balanced design for sequence. The Go stimuli had to be responded by pressing a button with the respective hand. The study was performed double-blind cross-over in a counterbalanced design, the two sessions (sham and real exposure) were separated by one week, and exposure lasted 45 min. Each session consisted of a pre- and a post-exposure assessment with 3 runs each lasting approximately 10 min. During the task the BOLD signal and reaction times were recorded. Exposure was delivered by a commercial mobile phone controlled by a test card to transmit a basic GSM signal (carrier frequency 902.4 MHz, modulation 217 Hz and 8.33 Hz) at a peak power of 2 W (0.25 W average). The phone was fixed to a helmet with a distance of 1.5 cm from the tragus. A non-operational second phone was fixed at the other side for blindness and symmetry. BOLD responses revealed significant activations in some brain areas with Go responses, after both, real and sham exposure. Brain activity changes due to GSM exposure were not observed in this study. A General Estimating Equation with three main factors (exposure, trial, and hand) did not reveal significant exposure effects on reaction time, which might be expected given the small sample size. An analysis of pre- post assessment by exposure yielded a significant decrease of reaction time after real exposure, which was not observed after sham exposure.

Human studies – waking EEG related to cognitive tasks

Three studies looked at effects of RF exposure on the waking EEG related to cognitive tasks. One of these studies (Hountala et al. 2008), however, provides insufficient information on exposure for its assessment. Another one (Leung et al. 2011), is listed in Table 13 and discussed in the context of event related potentials. Using the same exposure setup Vecchio et al. (2012b, Table 12) investigated whether the EEG effects observed in a previous study are related to alterations in cognitive-motor functions. In a double-blind, placebo-controlled cross-over design EEG was recorded continuously at 56 sites in 11 subjects (24-63 years) during a go/no-go task before and after GSM and sham exposure. At the behavioural level, faster reaction times were observed in the post GSM exposure condition than in the pre GSM exposure condition (see Table 14). No statistically significant difference was observed in the sham session. To analyse task related EEG changes the alpha event-related desynchronization (ERD) was computed at the individual level for low- and high-frequency alpha sub-bands. There was less power

decrease of widely distributed high-frequency alpha rhythms in the post- than in the pre-exposure period of the GSM session while no effect was found in the sham session. The results indicate an exposure effect both at the EEG and the behavioural level.

Human studies – event-related potentials (ERP) and slow brain potentials

Since the last Opinion eleven studies were published which investigated RF effects on event related potentials or slow brain potentials (Table 13). In three of these studies (Maganioti et al. 2010, Colletti et al. 2011, Mandala et al. 2014) there is insufficient information on exposure to be considered in more detail in this review.

Table 13. RF-EMF effects and event related potentials / slow brain potentials

Authors	Signal type	Exposure side [1]; Antenna [2]; Design [3]	Sample	Exposure Duration (ED); EEG/Exposure [4]; EEG electrodes (E)	Changes with exposure
Kwon et al. (2009)	902 MHz, „pulsed" 217 Hz, width 0.58 [ms] modulation? (SMIQ generator, RF amplifier) SAR_{10g} = 0.82 W/kg	S: L, R<br>A: mp<br>D: sb?, co	17 (12f / 5m); 23.1 ± 4.5 years	ED: 6 min per block;<br>EEG/E: 3;<br>E: 7+M1+M2	No effect of exposure on auditory ERP components evoked by mismatch negativity (MMN) for stimuli deviant in frequency, duration, intensity and gap
Kwon et al. (2010a)	902 MHz, „pulsed" 217 Hz, width 0.58 [ms] modulation? (SMIQ generator, RF amplifier) SAR_{10g} = 0.82 W/kg	S: L, R<br>A: mp<br>D: sb, co	17 (13f / 4m); 11-12 years	ED: 6 min per block;<br>EEG/E: 3 (= Kwon 2009);<br>E: 7+M1+M2	No effect of exposure on auditory ERP components evoked by mismatch negativity (MMN) for stimuli deviant in frequency, duration, intensity and gap
Kwon et al.(2010b)	902 MHz, „pulsed" 217 Hz, width 0.58 [ms] modulation? (SMIQ generator, RF amplifier) SAR_{10g} = 0.82 W/kg	S: L, R<br>A: mp<br>D: sb, co	17 (11f / 6m) 25.9 ± 4.3 years	ED: 5 min per block;<br>EEG/E: 3;<br>E: ear chan. (ABR) + Fp1,Fp2	No effect on latency and amplitudes of auditory brainstem responses (ABR)
Tommaso et al. (2009)	900 MHz test signal, probably pulse mod. 217 Hz, width 577 μs (mp test mode / externally controlled by software);<br>a) SAR_{10g} = 0.5 W/kg<br>b) RF dissipated internally	S: L<br>A: mp<br>D: db, co	10 (5f / 5m); 20-31 years	ED: <10 min;<br>EEG/E: 2/3;<br>E: 30 + mastoids	Amplitude reduction of the initial contingent negative variation (iCNV) for both conditions: a) active mp transmitter with real RF emission and b) active mp transmitter without RF emission, compared to condition c) mp inactive
Parazzini et al. (2010)	1947 MHz WCDMA signal (mobile phone, controlled by software, amplifier, small patch antenna);<br>SAR_{1g} = 1.57W/kg (roughly at the cochlear)	S: L or R<br>A: s<br>D: db; co	73 (38 f / 35 m) 22.8 ± 3.8 years	ED: 20 min<br>EEG/E: 1<br>E: Pz, Cz, Fz, ref. nose	No effect of exposure on latencies and amplitides: N1, P2, N2, P3
Leung et al. (2011)	GSM: 894.6 MHz duty cycle 0.125 (phone in test mode)<br>SAR_{10g} = 0.7 W/kg;<br><br>WCDMA: 1900 MHz simulated WCDMA signal (external source);<br>SAR_{10g} = 1.7 W/kg	S: L, R counter-balanced<br><br>A:<br>GSM: mp<br>WCDMA: s<br><br>D: db, co	41 (20f / 21m): 13-15 years<br>42 (21f / 21m): 19-40 years<br>20 (10f / 10m): 55-70 years;<br><br>7 subjects excluded	ED: 51 min;<br>EEG/E: 2;<br>E: 61 scalp sites	GSM:<br>Acoustically evoked potentials: Larger N1 amplitiude, no effects for amplitudes of P3a and P3b and no effect for all three latencies;<br><br>WCDMA:<br>No effects
Papageorgiou et al. (2011)	Wi-Fi 2.45 GHz access point, OFDM 1.5m distance (0.5 V/m)	S: n.a.<br><br>A: dual dipole<br><br>D: sb, co	30 (15 f / 15m); 23.8 ± 1.7 years	ED: <10 min;<br>EEG/E: 3 (checked elsewhere);<br>E: 30 + ears	EEG potentials evoked by three different conditions (inhibition, initiation and baseline) of a modified Hayling Sentence Completion task: statistically significant exposure*gender interaction in the inhibition condition (in 15 out of 30 electrodes): higher amplitude under exposure for females; no other significant EMF effect
Trunk et al. (2013)	WCDMA 1947 MHz (mp, controlled by service software + RF amplifier); SAR_{1g} < 1.75 W/kg	S: R<br>A: s<br>D: db, co	26 (12f / 14m); 24.1 ± 6.7 years	ED: 30 min;<br>EEG/E: 1;<br>E: Fz, Cz, Pz	No effect on amplitude and latency on auditory ERP components (MMN experiment)
Trunk et al. (2014)	WCDMA: 1947 MHz; simulated power control scheme;<br>(mp, controlled by service software + RF amplifier, patch antenna);<br>SAR_{10g} = 0.73 W/kg<br>Four conditions:<br><br>1.No caffeine, no RF<br>2.Caffeine, no RF<br>3.No caffeine, RF<br>4.Caffeine and RF	S: R<br>A: s<br>D: db, 4-way co	25 (9f /16 m) 21.1 ± 3.6 years	ED: 15 min<br>EEG/E:1<br>E:19 electrodes (cap 10/20 system) with nose as reference	Outcome: ERP in an oddball paradigm:<br>Caffeine: reaction time and area under the curve of the ERP were significantly decreased under caffeine exposure; no EMF effects (neither alone nor when co-exposed wit caffeine) on the ERP parameters and reaction time.

1) L = left, R = right, LR = both sides; n.a. = not applicable
2) mp = mobile phone, s = similar to mobile phone, l = larger head area, bs = base station
3) db = double-blind, sb = single-blind, co = cross-over, pg = parallel group
4) 1 = not simultaneously; 2 = simultaneously without or without information on electromagnetic interference tests, 3 = simultaneously with information on electromagnetic interference tests

Kwon et al. (2009) used the Mismatch Negativity (MMN) task, which is an auditory ERP elicited by infrequent stimuli deviant in frequency, duration, intensity or gap from the repetitive frequent standard stimuli in a sound sequence. The sample comprised 17 (12 females) young healthy adults (23.1 ± 4.5 years). EEG was recorded at 9 sites (there was no radiofrequency interference) in three conditions: two with verum exposure and one with sham exposure at each side of the head. Each exposure condition was applied for 6 min, and all exposures were applied consecutively in one session in an order counterbalanced across subjects. An externally generated signal (902 MHz, pulsed with 217 Hz; SAR_{10g} = 0.82 W/kg) fed to the antenna of a mobile phone was used for exposure. In this study precautions were taken to prevent possible sensations of audible noise caused by equipment. It is not clear whether the study was double-blind. All types of deviants in stimuli resulted in a MMN, however, there was no effect of GSM exposure on the results.

The same exposure setup and experimental design was used to analyse the MMN in 17 children (13 girls), aged 11-12 years (Kwon et al. 2010a). In this single-blind study a short exposure did not result in significant exposure effects. The authors themselves claimed that this study only had enough power to detect large effect sizes.

To investigate whether GSM exposure has an effect on brainstem auditory processing the same exposure setup was used again in a sample of 17 young healthy subjects (11 females, 25.9 ± 4.3 years) (Kwon et al. 2010b). To eliminate GSM artefacts, which were identified during the recording of auditory brainstem responses (ABR), the position of the phone was adjusted. Hence, the experiments were not double-blind. The results did not show an effect on the ABR suggesting that a short-term exposure to mobile phones EMF does not affect the transmission of sensory stimuli from the cochlea up to the midbrain.

Parazzini et al. (2010) investigated possible effects of UMTS exposure on the auditory system in 73 volunteers (38 females and 35 males; 22.8 ± 3.8 years). The ear with the better auditory functioning outcomes was selected for exposure and assessments, which were made pre and post a 20 min exposure. The study was double-blind and cross-over. Sessions were scheduled at different days. WCDMA exposure at 1957 MHz was delivered by a mobile phone controlled by software and connected to an amplifier and a patch antenna positioned close to the ear. Peak spatial SAR_{1g} was measured in a phantom to be 1.75 W/kg at 2 cm depth from the phantom shell surface, which roughly corresponds to the position of the cochlear. The authors prevented any interaction between the exposure and the audiometric transducer. None of the outcomes reflecting auditory function (hearing threshold level, distortion product otoacoustic emissions, contralateral suppression of transiently evoked otoacoustic emissions, and auditory evoked potentials, for which sample sizes varies between 25 and 57) showed statistically significant exposure effects after correction for multiple testing.

Possible effects of a 30 min UMTS exposure (simulated mobile phone use, 1947 MHz, SAR1g slightly less than 1.75 W/kg) on auditory event related potentials (ERP) in a mismatch negativity (MMN) experiment with 10% frequency deviant tones were investigated by Trunk et al. (2013) in 26 young subjects (12 females, 24.1 ± 6.7 years). The test was run prior and following a 30 min exposure. No EMF effects on amplitude and latency of any ERP component were observed.

The same group also investigated the effect of electromagnetic fields (duration of exposure 15 min) when (co-)exposed with caffeine (3mg/kg) on event related potentials (ERP) in an oddball paradigm with 80% frequent and 20% rare target stimuli (Trunk et al. 2014). The sample consisted of 25 healthy subjects (9 females and 16 males, mean age: 21.1 ±3.6 years) UMTS signals were delivered by a mobile phone controlled by a test software provided by the phone manufacturer. For exposure a RF amplifier and a patch antenna mounted on a plastic headset were used. SAR values were measured in a phantom to be peak spatial SAR_{1g} = 1.75 W/kg and peak spatial SAR_{10g} = 0.73 W/kg, respectively. The study was performed in a double-blind 4-way cross-over design, with the following experimental conditions: 1) no caffeine and no mobile phone, 2) caffeine only, 3) mobile phone only, and 4) caffeine and mobile phone. While the area under the

curve of the ERP and the reaction time were significantly decreased under caffeine exposure, no effects of mobile phone exposure alone or in combination with caffeine were observed for the four parameters characterizing the P300 of the ERP and the reaction time.

In a sample of 10 subjects (5 females, 20-31 years) Tommaso et al. (2009) analysed a possible exposure effect on the initial contingent negative variation (iCNV) during exposure to a) a GMS phone (900 MHz, SAR_{10g} = 0.5 W/kg) by a transmitting mobile phone and b) by a modified mobile phone with the RF power dissipated internally (SAR approximately 30dB less than in condition a); called sham in this paper) compared to c) a condition with the phone completely switched off. All three tests were done on the same day in a double-blind cross-over design. Electromagnetic interference of the EEG device was tested, but not in the experimental setting. A decreased amplitude of the initial contingent negative variation (iCNV), diffusely distributed over the scalp was observed for conditions a) and b). The authors interpreted their results as the consequence of reduced arousal and expectation of warning stimuli, explainable in terms of effects by both the GSM signal and the ELF magnetic fields produced by currents in the internal circuits.

Leung et al. (2011) used the same sample, exposure and study design as described by Croft et al. (2010) for the analysis of the waking EEG to investigate possible effects of 2nd (2G) and 3rd (3G) generation mobile phones on EEG and behavioural outcomes in an auditory 3-stimulus oddball paradigm and an N-back task on working memory. The sample comprised 41 adolescents (13-15 years, 14.1 ± 0.9 years), 42 young adults (19-40 years, 24.5 ± 4.5 years) and 20 elderly subjects (55-70 years, 62.2 ± 3.9 years). EEG was recorded at 61 sites, 7 participants had to be excluded. Out of the six variables considered for the event related potentials (ERP) resulting from the auditory task (peak amplitude and latency of N1 P3a and P3b), the only one showing an exposure effect was the N1 amplitude. It was larger in the 2G exposure condition than under sham (no age effects). The EEG analysis for the N-back task revealed delayed ERD/ERS responses of the alpha power in both exposure conditions as compared to sham.

Since the last Opinion one study (Papageorgiou et al. 2011) was published, which analysed the effect of a Wi-Fi signal (2.45 GHz, 0.5 V/m) on event related potentials (ERPs) evoked in three different conditions (inhibition, initiation and baseline) of a modified version of the Hayling Sentence Completion task. In a single-blind cross-over design with randomized exposure (Wi-Fi or sham) 30 subjects (15 females, 23.8 ± 1.7 years) performed the test. EEG was recorded from 30 electrodes during exposure while performing the task. The only statistically significant effect seen for the P300 amplitude was one for exposure*gender interaction in the inhibition condition (at 15 out of the 30 electrodes). In the absence of the Wi-Fi signal the amplitudes in males were greater than in females (not statistically significant), while under exposure this was reversed: females had significantly higher amplitudes.

Human studies – cortical excitability assessed by transcranial magnetic stimulation (TMS)

One study investigated the effect of a 45 min GSM-exposure on cortical excitability in 10 patients (5 females and 5 males, age range 19 – 67 years, mean: 39.9 ± 18.1 years, two women were in menopause, for the other three assessments were made during the early follicular phase) with cryptogenic focal epilepsy originating outside the primary motor area (M1) by means of paired-pulse transcranial magnetic stimulation (TMS) (Tombini et al. 2013). In a double-blind counterbalanced cross-over study three exposures (ipsilateral exposure (IH), contralateral exposure (CH) and sham) were applied 1 week apart. TMS was applied immediately before and following exposure. Exposure was delivered by a commercially available mobile phone equipped with a test card. The typical GSM signal (carrier frequency 902.4 MHz, average power of 0.25 W) was delivered resulting in a peak spatial SAR of 0.5 W/kg according to measurements in a phantom (Ferreri et al. 2006). For weight balance and blinding an identical phone was placed at the other side of the head. Resting motor threshold (RMT) and amplitude of the motor-evoked potential (MEP) prior to exposure did not show interhemispheric

differences. The MEP ratio for paired TMS showed the well-known inhibition-facilitation curve with inhibition at interstimulus intervals (ISI) of 1 and 3 ms, lack of inhibition/facilitation at ISIs 7, 9 and 11 ms and facilitation at ISI 13 ms. Differences between hemispheres were only observed for ISI 1 ms where inhibition at the ipsilateral hemisphere was lower than at the contralateral side. Following exposure there was no effect on RMT. The MEP amplitude revealed a significant hemisphere*condition*time interaction, which was due to a significant reduction of amplitude in the same hemisphere after EMF exposure of the contralateral side. The MEP ratio resulting from paired-pulses showed a significant increase at the contralateral hemisphere after exposure at the contralateral hemisphere as compared to the pre-exposure situation. These results indicate that EMF modulates cortical excitability in epileptic patients only when exposure was applied contralateral to the epileptic focus.

Human studies – cognition

Since the last Opinion 10 papers investigating RF-EMF effects on cognition (as primary focus of research or as a minor additional result) have been published (Eltiti et al. 2009, Luria et al. 2009, Sauter et al. 2011, Hareuveny et al. 2011, Leung et al. 2011, and Schmid et al. 2012a, 2012b, Vecchio et al. (2012b), Wallace et al. (2012), Loughran et al. (2013), see Table 14) as well as a systematic review and two meta analyses on the topic (Barth et al. 2008, 2012, Valentini et al. 2010 and 2011). Furthermore, Regel and Achermann (2011) published a paper with recommendations concerning methodological standards in this research area.

Eltiti et al. (2009) investigated 114 subjects (54.0 ± 15.4 years, no information concerning males and females in the sample) in a three-way double-blind cross-over design. Exposures were combined GSM 900 and GSM 1800 signals, total: 100 W/m^2; UMTS 2020 MHz, 100 W/m^2; and sham exposure. Power flux densities roughly correspond to the maximum an individual is exposed to by real base stations. Repeated measures ANOVA revealed no statistically significant differences for the outcome variables of three cognitive tests performed during exposure: forward digit span (DS), digit symbol substitution test (DSST) and mental arithmetic task (MA). Testing was done in test sessions at least one week apart at approximately the same time of the day. 44 (20 females) out of the 114 subjects were used as an age-matched control sample for 44 self-reported sensitive individuals (18 females, 46.1 ± 13.2 years). The authors claim that overall cognitive functioning was not affected by short-term exposure (50 min) to either GSM or UMTS. The sensitive group had an impaired performance on the DS task under both exposure conditions as compared to sham, which was not present after Bonferroni correction for multiple testing.

Using a single-blind three parallel-group design Luria et al. (2009) investigated effects of a transmitting mobile phone on cognition in a spatial working memory task in 48 male subjects (age not reported). For exposure, a head-worn frame holding two standard mobile phones equipped with test SIM cards and controlled by a GSM test system was used. Either no transmission at all or one phone at maximum output power (2 W) at 890.2 MHz, pulsed at 217 Hz, pulse duration 577 µs was set (max. SAR_{10g} values of 0.54 to 1.09 W/kg are reported.) Each of the 16 subjects per group was exposed on the left or right side of the head or by sham during the cognitive test, which was divided into 12 blocks of 50 trials each. 15 additional trials before the start of exposure served for practising. The whole duration per subject was approx. 1 hour. Average RT of the right-hand responses under left-side exposure condition was significantly longer than those of the right-side and sham-exposure groups averaged together during the first two time blocks. Authors conclude that experiment duration, exposure side and responding hand may influence the outcome of experiments for detection of EMF effects.

In a follow-up study (Hareuveny et al. 2011) the question was investigated, whether the results found by Luria et al. (2009) and previous studies represent an effect of EMF or whether they are due to other causes. The same single-blind design, but with 29 male subjects (age not reported) in two groups for left and right exposure (no sham) was used while the phones were equipped with external antennas placed far from the subjects.

This setup was chosen to prevent any significant radio frequency exposure from the mobile phones. The weak emission from the external antennas was measured, but an investigation of possible residual exposure from the phones is not reported. A longer reaction time for right-hand responses under left side exposure compared to right side exposure was found as a trend. The authors claim that the results obtained without EMF are similar to those with EMF. This suggests that effects of mobile phones previously attributed to EMF could be the result of, for example, low frequency magnetic fields or warming caused by the phones' electronics.

Table 14. RF-EMF effects and cognition

Authors	Signal type	Exposure side 1) ; Antenna 2) ; Design 3)	Sample	Exposure Duration	Changes with exposure
Eltiti et al. (2009)	GSM:<br>Combined GSM 900 and GSM 1800 signal (10 mW/m²);<br><br>UMTS:<br>2020 MHz signal (10 mW/m²)<br><br>(technical info in 2007 publication)	S: n.a.<br>A: bs<br>D: db, co	114:<br>54.0 ± 15.4 years<br><br>44 (18f / 26m) with and 44 (20f / 24m) age-matched without MPRS 4);<br>46.1 ± 13.2 years	50 min	Forward digit span (DS): no exposure effect<br>Digit symbol substitution test (DSST):no exposure effect<br>Mental arithmetic task (MA): no exposure effect<br><br>Forward digit span (DS): no exposure effect for the controls; subjects with MPRS: impaired performanace under both exposure conditions as compared to sham – no effect after Bonferroni correction;<br>Digit symbol substitution test (DSST): no exposure effect for both groups<br>Mental arithmetic task (MA): no exposure effect for both groups
Luria et al (2009)	890.2 MHz, pulse mod. 217 Hz, width 0.577 ms (mp,test sim cards, test system);<br>SAR_{10g} = 0.54 – 1.09 W/kg	S: LR<br>A: mp<br>D: sb, pg	48 males;<br>3 groups with 16 males each;<br>age not reported	ca. 60 min	Working memory task:<br>Reaction time increased only for match responses for left-side exposure and first two of twelve test blocks, compared to averaged sham and right-side exposure groups
Hareuveny et al. (2011)	890.2 MHz, pulse mod. 217 Hz, width 0.577 ms - but no RF emission (mp,test sim cards, test system, RF output to far away antennas)	S: L, R (pg)<br><br>A: mp, no RF<br><br>D: sb, pg	29 males;<br>age not reported;<br>2 groups,<br>no sham	?	Working memory task: no significant differences for exposure sides
Sauter et al. (2011)	1) 900 MHz pulse modulated,<br>2) WCDMA: 1966 MHz;<br>SAR_{10g} = 2 W/kg<br><br>(technical info in other publications)	S: R<br>A: s<br>D: db, co	30 males;<br>18-30 years	7 h 15 min	Divided attention:<br>GSM: No effect on reaction time and accuracy in the visual and acoustic task;<br>WCDMA: significantly increased reation time in the visual task in the morning – not in the acoustic task, effects not seen in the afternoon.<br>Selective attention:<br>GSM, WCDMA: No exposure effect on reaction time and accuracy<br>Vigilance:<br>GSM: statistically significant higher number of correct reactions in the morning – not in the afternoon; mean reaction time in the second 5 min of the test is significantly shorter in the morning; WCDMA: significantly shorter reaction time in the first part of the test in the morning.<br>Working memory:<br>GSM, WCDMA: No exposure effect on reaction time and accuracy
Leung et al. (2011)	GSM: 894.6 MHz duty cycle 0.125 (phone in test mode)<br>SAR_{10g} = 0.7 W/kg;<br><br>WCDMA: 1900 MHz simulated WCDMA signal (external source)<br>SAR_{10g} = 1.7 W/kg	S: LR counter-balanced<br><br>A:<br>GSM: mp<br>WCDMA: s<br><br>D: db, co	41 (20f):<br>13-15 years<br><br>42 (21f):<br>19-40 years<br><br>20 (10f):<br>55-70 years	51 min	3-stimulus oddball paradigm: no exposure effect (total sample and by age group) on accuracy and reaction time;<br>N-back task: reaction time not affected by exposure; accuracy worse under UMTS exposure in the group of adolescents.
Schmid et al. (2012a)	1) 900 MHz, pulse mod. 14 Hz, width 2.3 ms (crest factor 31)<br>2) 900 MHz, pulse mod. 217 Hz, width 0.577 ms (crest factor 8);<br>both active cond.:<br>SAR_{10g} = 2 W/kg	S: R<br>A: I<br>D: db, co	30 males;<br>20-26 years	30 min	Simple reaction time task (STR): no exposure effect;<br>Choice reaction time tals (CRT): no exposure effect;<br>N-back task: no exposure effect.
Schmid et al. (2012b)	magnetic field (MF) or 900 MHz RF (amplitude modulated); MF or modulation: 2 Hz, 8 Hz and harmonics up to 20 Hz;<br>RF: SAR_{10g} = 2 W/kg;<br>MF: 0.7 mT (amplitude, temporal peak) nearly all over the brain	S:<br>MF: LR<br>RF: R<br><br>A: I<br>D: db, co	25 males;<br>20-26 years	30 min	Simple reaction time task (STR): increase in reaction time after MF exposure; no effect on accuracy:<br>Choice reaction time tals (CRT): no exposure effect;<br>N-back task: no exposure effect.
Vecchio et al. (2012b)	GSM 902.4 MHz (incl. mod. components 217 Hz & 8.33 Hz) (mp set by a test card);<br>SAR_{10g} = 0.5 W/kg	S: L<br>A: mp<br>D: db, co	11;<br>24-63 years	45 min	Go/no-go task: faster reaction times following exposure in a pre-post exposure design; no effect in the sham condition
Wallace et al. (2012)	TETRA base station signal (420 MHz); realistic time slot occupancy variation – average 50%;<br>power flux density: 10 mW/m²);<br>estimated whole body SAR = 0.3 mW/kg	S: n.a.<br>A: bs<br>D: a) open<br>b) db, co	48 sensitive subjects (29f / 19m)<br>18 – 80 years<br><br>132 controls (67f / 65m)<br>18 – 80 years	50 min	Results for the double-blind condition are reported:<br><br>Backwards digit span test and Operation Span: Both groups: no exposure effects
Loughran et al (2013)	900 MHz carrier, GSM mobile phone like modulation, Sham or<br>SAR_{10g} = 0.35 W/kg or<br>SAR_{10g} = 1.4 W/kg	S: L<br>A: I<br>D: db, co	22 adolescents (12 m/ 10 f)<br>11 – 13 years	ED: 30 min;<br>EEG/E: 1;<br>E: C3, C4, O1, O2, M1+M2	No significant exposure effects on speed and accuracy in a simple and complex reaction time task and in a 1- and 2-back working memory task.

1) L = left, R = right, LR = both sides; n.a. = not applicable
2) mp = mobile phone, s = similar to mobile phone, l = larger head area, bs = base station
3) db = double-blind, sb = single-blind, co = cross-over, pg = parallel group
4) 1 = not simultaneously; 2 = simultaneously without or without information on electromagnetic interference tests, 3 = simultaneously with information on electromagnetic interference tests

Using a double-blind cross-over design, Sauter et al. (2011) investigated a possible effect of RF-EMF exposure as compared to sham on outcomes of tests on attention (divided attention, selective attention, and vigilance) and working memory. The sample comprised 30 healthy male subjects (25.3 ± 2.6 years) who were tested on nine study days in which they were exposed to three exposure conditions (sham, GSM 900 MHz pulsed with 217 Hz and WCDMA 1966 MHz in a randomly assigned and balanced order). Exposure was delivered by a specially developed antenna, and simulated a cell phone use at maximum RF output power. The transmitted power was adjusted in order to approach but not to exceed a localised SAR_{10g} = 2.0 W/kg. Each test session comprised a morning and an afternoon session within a fixed timeframe. Subjects were constantly exposed for 7 hours and 15 min during the day. Reaction time in the divided attention task was significantly increased during WCDMA exposure in the morning session but not in the afternoon session, and only with regard to the optic part of the test. A better performance in the vigilance task was seen under GSM exposure in the morning – not in the afternoon. Overall, time-of-day effects were more pronounced. The results do not support that RF EMF exposure has a negative effect on cognitive performance. Control for time-of-day in studies of cognitive performance has to be added to the list of issues that need consideration when designing bioelectromagnetic studies on cognitive performance summarized by Regel and Achermann (2011).

The study by Leung et al. (2011) described in more detail under the heading Human studies – event-related potentials (ERP) and slow brain potentials also investigated the effect of 2G and 3G mobile phone signals on behavioural outcomes of the auditory 3-stimulus oddball and the N-back test. For the oddball test, the behavioural outcomes (accuracy and reaction time) were not affected by exposure in the total sample as well as in age groups analysed separately. The behavioural data of the N-back task showed that reaction time was not affected by exposure while accuracy showed an effect in the 3G exposure condition with better accuracy in the sham condition and a significant effect of age. The exposure related reduced accuracy was only observed in the group of adolescents.

The studies by Schmid et al. (2012a and 2012b) mentioned above, which primarily aimed at investigating different pulse-modulations of RF-EMF and a pulsed magnetic field on sleep EEG, also looked at cognitive performance during the 30 min of exposure prior to sleep in the evening. No exposure effects were seen on reaction time in a simple (SRT) and 2-choice reaction time task (CRT) as well as in an N-back working memory test paradigm with the 14 Hz and 217 Hz exposure (Schmid et al. 2012a). Following exposure to the 2 Hz magnetic field exposure a significant increase in the SRT was seen while performance accuracy was not affected (Schmid et al. 2012b).

The study by Loughran et al. (2013) which looked at effects of a GSM 900 MHz mobile-phone like exposure on the waking EEG in 22 adolescents (12 males) aged between 11 and 13 years (12.3 ± 0.8 years) also looked at cognitive performance. They applied three exposure conditions in a double-blind, randomized, and counter-balanced crossover design with a planar antenna at the left side of the participant's head: Sham, "low SAR" (psSAR 0.35 W/kg) and "high SAR" (psSAR 1.4 W/kg). Time of day for the investigation was kept constant within individuals. Participants performed the same three cognitive tasks as described above (Schmid et al. (2012a and 2012b)). No significant differences between exposure conditions were observed for any of the three different tasks.

Wallace et al. (2012) investigated acute effects of exposure to a TETRA base station signal (420 MHz; power flux density: 10 mW/m^2 resulting in an estimated whole body SAR of roughly 0.3 mW/kg) on short-term memory, working memory and attention in subjects between 18 and 80 years of age. They had three exposure conditions, ≥ 1 week apart at approximately the same time of the day: an open provocation test session with both real and sham exposure was followed by two double-blind test sessions with either

sham or real exposure (duration 50 min). 48 sensitive (29 females, 19 males) and 132 controls (67 females and 65 males) completed all three sessions. After applying performance exclusion criteria the number of subjects varied test specifically between 36 and 48 for the sensitives and 107 to 129 for the controls. The authors found no evidence for a negative impact of TETRA base station signals on memory or processing capacity in either the control or the sensitive group in the double-blind condition. They also considered physiological measures recorded while the subjects performed the Backwards Digit Span and the Ospan Test. Neither in the control group nor in the group of sensitive subjects a significant exposure effect was observed for the means and standard deviations of heart rate, skin conductance and blood volumetric pressure.

Human studies – regional blood flow, blood concentration and oxygenation changes

Out of the four papers published since the last Opinion, one (single-blind) study (Volkow et al. 2011) among others lacks dosimetry, distance between phone and head, as well as information about the anatomical distribution of SAR and hence is not discussed in detail here. In a small study on 9 healthy male volunteers (age not reported) Mizuno et al. (2009, see Table 15) investigated, in a single-blind randomized cross-over design, whether a 30 min exposure to WCDMA (SAR_{10g} = 2.0 W/kg) delivered by a microstrip patch antenna has an effect on blood flow as assessed with positron emission tomography (PET) with two scans during and two scans after exposure. Electromagnetic interference to PET was tested. The results indicate that EMF emitted by 3G WCDMA-type mobile phones do not significantly change rCBF during or after 30 min exposure. The reason for choosing a single-blind design was "because it was expected to disclose EMF effects whereas double blind studies tend to highlight null effects" (Mizuno et al. 2009, p 537).

Table 15. RF-EMF effects and regional blood flow, blood concentration and oxygenation

Authors	Signal type	Exposure side [1]; Antenna [2]; Design [3]	Sample	Exposure Duration (ED); PET/NIRS/tDS and Exposure [4]	Changes with exposure
Mizuno et al. (2009)	WCDMA 1950 MHz (signal generator, amplifier) SAR_{10g} = 2 W/kg	S: R<br>A: s<br>D: sb, co	9 males	ED: 30 min; PET/Exposure: 3	No effect of exposure on regional cerebral blood flow
Spichtig et al. (2011)	WCDMA 1900 MHz downlink (bs) signal (signal generator, amplifier)<br>low: SAR_{10g} = 0.18 W/kg<br>high: SAR_{10g} = 1.8 W/kg	S: L<br>A: l<br>D: db, co	16 males; 26.8 ± 3.9 years	ED: 30 min, intermittent, 20s on, 60s off; NIRS/Exposure: 3	Significant short-term increase from baseline for Δ[O2Hb] and Δ[tHb] at 0.18 W/kg exposure<br>Significant decrease of Δ[HHb] at 0.18 W/kg and at 1.8 W/kg
Lindholm et al. (2011)	GSM 902.4 MHz test signal (mp antenna fed by a remote amplifier and a mp controlled using test software); SAR_{10g} = 2 W/kg	S: R<br>A: mp<br>D: db, co	26 males; 14-15 years	ED: 15 min; NIRS/Exposure: 3	No significant exposure effects for local cerebral blood flow
Ghosn et al. (2012)	GSM 900 MHz; duty cycle 0.125 (mobile phone test software) SAR_{10g} = 0.49 W/kg	S: L<br>A: mp<br>D: db, co	29 (19f / 10m) 21-35 years	ED: 20 min Transcranial Doppler sonography/Exposure: 2	Cerebral arterial blood flow velocity, pulsatility index and resistance index as assessed by transcranial Doppler sonography did not show any exposure effects.

1) L = left, R = right, LR = both sides; n.a. = not applicable
2) mp = mobile phone, s = similar to mobile phone, l = larger head area, bs = base station
3) db = double-blind, sb = single-blind, co = cross-over, pg = parallel group
4) 1 = not simultaneously; 2 = simultaneously without or without information on electromagnetic interference tests, 3 = simultaneously with information on electromagnetic interference tests

A potential effect of intermittent UMTS-EMF exposure (peak SAR_{10g} 1.8 W/kg, peak SAR_{10g} 0.18 W/kg and sham; exposure: 20 s on/60 s off) on blood circulation in the head (auditory region) was investigated by Spichtig et al. (2012) in a double-blind, randomized cross-over design. They used near-infrared spectroscopy (NIRS) and considered a short-term (occurring within 80s) and medium-term (occurring from 80 s to 30 min) effects in a study sample of 16 healthy young males (26.8 ± 3.9 years) looking at changes in oxy- [O2Hb], deoxy- [HHB] and total haemoglobin [tHb] as well as at heart

rate (HR). Furthermore, subjective well-being, tiredness and counting speed in the task, which was used to control concentration, were considered. These parameters did not vary with exposure. During exposure to 0.18 W/kg, a significant short-term increase in Δ[O2Hb] and Δ[tHb] was found, which is small (≈17%) compared to functional brain activation. Δ[HHb] showed a significant decrease at 0.18 W/kg and at 1.8 W/kg in the range of physiological fluctuations. The change in heart rate from baseline was significantly higher at 1.8 W/kg than for sham with regard to medium-term effects.

Possible effects of a short term exposure (15 min) to a RF EMF produced by a GSM mobile phone on thermal responses (ear canal and facial skin), local blood flow in the head, and the autonomous nervous system (ECG and continuous blood pressure) was investigated by Lindholm et al. (2011) in a double-blind sham-controlled cross-over design. Subjects (26 boys aged 14-15 years) were exposed to a mobile phone GSM test signal (SAR_{10g} = 2 W/kg) in a climatic chamber. Blood flow was measured using near-infrared spectroscopy (NIRS). No significant exposure effects were observed for local cerebral blood flow, ear canal temperature, and autonomic nervous system responses.

Ghosn et al. (2012) investigated whether an acute 20 min exposure to a commercial mobile phone controlled by manufacturer’s test software to transmit a GSM signal at 900 MHz, 250 mW average had an effect on cerebral blood flow. Peak spatial SAR_{10g} was measured in a phantom to be 0.49W/kg. In a double-blind cross-over study sham and real exposure were applied in two separate test sessions. The sample comprised 29 subjects (10 males and 19 females) between 21 and 35 years. Possible effects were investigated by transcranial Doppler sonography with middle cerebral arterial blood flow velocity, pulsatility index and resistance index as outcome parameters. None of these parameters showed significant effects during and after exposure.

Human studies – others

Besides Spichtig et al. (2012) and Wallace et al. (2012), which have already been mentioned, Havas et al. (2010), Havas and Marrongelle (2013), Parazzini et al. (2013), and Choi et al. (2014) investigated RF-EMF effects in heart rate (HR) and its variability (HRV). Due to problems with the exposure setup, the studies by Havas et al. (2010) and Havas and Marrongelle (2013) are not further discussed.

Parazzini et al. (2013) analysed the effect of a GSM exposure on nonlinear dynamics of the heart rate variability in 26 subjects (12 females and 14 males, mean age: 25.5 ± 1.5 years) without cardiac or nervous system disorders between 21 and 28 years. In a double-blind, randomly assigned cross-over design subjects were exposed by a commercial mobile phone controlled by manufacturer’s test software to generate a GSM signal at 900 MHz (peak power 2W which corresponds to 0.25 W average). Subjects had two test sessions (sham and real exposure) at least one day apart. Exposure is described in more detail in a previous paper (Parazzani et al. 2007). The sham condition was realized by a resistive load on the external antenna connector of the phone resulting in no transmission. For blinding an identically looking device with no effect was used in the real exposure condition. Local SAR was measured in a head phantom, the only information reported is that local max SAR in the area of interest (hypothalamus and brainstem) was lower than 0.02 W/kg. All assessments were done in the morning to minimize circadian variation. No effect of exposure on nonlinear dynamics of heart rate was observed in this study.

In a double-blind randomized cross-over study Choi et al. (2014) investigated possible effects of a 32 min exposure to WCDMA on perception, eight subjective symptoms, heart rate, respiration rate and heart rate variability. The sample comprised two groups: a) 26 adults (13 females and 13 males, 28.4 ± 5.1 years), and b) 26 teenagers (13 females and 13 males, 15.3 ± 0.7 years). Exposure was delivered by a WCDMA module placed in a dummy phone, and controlled by a laptop. Double-blinding was assured by remote control of the laptop. Peak spatial SAR1g was measured to be 1.57 W/kg at the left cheek. Assessment of physiological parameters was performed for a duration of 5 min at four times: pre-exposure, after 11 and 27 min of exposure, and post exposure. Data

analysis is based on a comparison of changes from baseline (pre-exposure measure) by exposure separately for the two age groups. WCDMA exposure did not significantly affect the autonomous system, subjective symptoms and perception neither in adults nor in teenagers.

There is one group of researchers (Söderqvist, Hardell and co-workers) who looked at effects of RF EMF exposure on serum levels of various proteins (S100B, ß-trace and transthyretin (TTR)) discussed among others as putative indicators of a dysfunction of the blood brain barrier (BBB: S100b) and the blood cerebrospinal-fluid barrier (BCSFB: TTR) or as key enzyme in the synthesis of prostaglandin D2, which for example is involved in sleep regulation (ß-trace).

Söderqvist et al. (2009a) performed a descriptive cross-sectional study (n=314) to investigate whether S100B protein levels were higher among frequent than non-frequent users of mobile and cordless desktop phones. Blood serum was analysed and set against self-reported mobile phones use. The study failed to show that long- or short-term use of wireless telephones was associated with elevated levels of serum S100B. Logistic regression of dichotomized serum transthyretin (TTR) levels (a less brain-specific marker) derived from the same observational sample yielded increased odds ratios that were statistically not significant (Söderqvist et al. 2009b). Further explorative (hypothesis-generating) data analyses yielded inconsistent results (Söderqvist et al. 2009b).

In an experimental provocation study, Söderqvist et al. (2009c) investigated the effect of a 30 min mobile phone exposure to an 890 MHz GSM signal with an average SAR_{1g} distribution of 1.0 W/kg in the temporal area of the head in 41 subjects (18-30 years, 24 females) using an indoor base station antenna. Repeated blood sampling before and after the provocation showed no statistically significant increase in the serum levels of S100B, while for transthyretin a statistically significant increase was seen in the final blood sample 60 min after the end of the provocation as compared to the prior sample taken immediately after provocation (p=0.02). Analysis of the ß-trace protein revealed no significant exposure related changes (Hardell et al. 2010). The volunteers who participated in this study plus 22 additional not exposed subjects were used for an observational epidemiological study showing that the concentration of ß-trace protein decreased with increasing number of years of use.

Söderqvist et al. (2012b) have also looked at the data from the earlier descriptive cross-sectional study (Söderqvist et al. 2009a) to see whether use of wireless phones was associated with lower concentrations of ß-trace protein. Overall, no statistically significant association between use of wireless phones and the serum concentration of ß-trace protein was found, neither with respect to short-term nor long-term use.

Given that mobile phone contact increases skin blood flow (SkBF) by heating Loos et al. (2013) investigated whether there is an additional RF-EMF specific effect on SkBF. The sample consisted of 20 healthy, young Caucasian subjects (12 females and 8 males, mean age: 25 ± 3.9 years). In two test sessions, performed at the same time of the day, under constant ambient conditions SkBF and skin temperature (Tsk) were measured by a laser Doppler system. Exposures were randomly assigned and the study was double-blind. Exposure was delivered by a commercial phone controlled by manufacturer's test software to deliver a peak 2 W (mean 0.25 W) 900 MHz GSM signal. The SAR was measured in a phantom resulting in a peak spatial SAR_{10g} = 0.49 W/kg. The sham condition was achieved by a resistive load as described above for the Parazzini et al. (2013) study. Following a 30 min rest period to stabilize Tsk, a five min baseline assessment was made. During the 20 min exposure (real or sham) measurements were made after 1, 5, 10, 15 and 20 min as well as 1, 5, 10, 15 and 20 after the exposure had been stopped and system has been removed. Furthermore a heat challenge was applied 25 min after the end of exposure by locally heating both sides of the face to 44°C for one minute. During real exposure the SkBF on the exposed side was significantly higher than during sham exposure, while temperature was not significantly different between conditions. The heat challenge led to a significantly greater early peak value for SkBF at

the exposed side following real exposure as compared to sham. These differences were not observed at the unexposed (contralateral) side of the face. The authors conclude that they identified specific, athermal modifications of the skin blood flow during mobile phone radiofrequency exposure.

Kwon et al (2011) investigated how exposure to GSM-type RF fields (902.45 MHz; 33 min; double-blinded conditions; SAR_{max} in head 0.74 W/kg; SAR_{10g} in brain 0.23 W/kg) influenced cerebral glucose metabolism in male volunteers. The subjects were investigated with positron emission scanning (PET) post-exposure and performed then also a simple vigilance test. Exposure caused a local suppression of glucose metabolism in the ipsilateral cortex, compared to sham conditions. This effect was not correlated to skin temperature and had no effect on the outcome of the vigilance test. In a second study from the same group (Kwon et al. 2012), the male subjects were exposed and scanned simultaneously, with the exposure emanating from three different anatomical locations. Exposure or sham exposure lasted for 5 minutes, and was concomitant with the vigilance test. No effects were noted on the cerebral blood flow or on the task performance. A slight temperature increase was noted in the ear canals during exposure.

In a double-blind, sham-controlled cross-over study Vecsei et al. (2013) investigated potential effects of a WCDMA exposure on pain threshold perception in response to thermal stimuli applied to the finger surface in 22 healthy young subjects (10 females and 12 males, 20-29 years). They used topical capsaicin as a positive control to validate the protocol (capsaicin lowers the thermal pain threshold - TPT). EMF exposure was delivered for 30 min by a commercial mobile phone controlled by manufacturer's test software to generate a WCDMA signal (1947 MHz carrier frequency, constant power) connected to a RF amplifier and a patch antenna placed close to the head. SAR was measured in a phantom resulting in a peak spatial SAR_{10g} of 0.73 W/kg. While the TPT was not affected by the UMTS-like EMF exposure, results indicate a slightly stronger desensitization effect across repeated trials under exposure for the contralateral side. The biological relevance of this observation, which needs to be confirmed by other studies, however, is not clear.

Discussion on neurophysiological studies

Overall, neurophysiological studies on possible effects of RF exposure on brain function in humans (macrostructure of sleep, power of the sleep EEG, resting state waking EEG, event-related potentials, slow brain potentials, cognition, as well as regional blood flow and oxygenation changes) yielded variable results. Reasons for this are, among others, different exposure conditions and set-ups, the great number of investigated outcome measures, missing replication studies in a strict sense, different levels of control of the vigilance state, and varying statistical properties. Effects sizes are usually not reported. Furthermore, it is rarely stated that measures were taken to avoid interference between the recording system and the exposure when assessments are made during exposure. RF interference can lead to artefacts as shown by Fouquet et al. (2013). On the other hand electrodes and cables of an EEG recording system change the RF field distribution (Murbach et al. 2014). In spite of the repeatedly stated "consistency" of results showing that pulsed RF EMF exposure leads to sleep EEG effects (SSM 2013), power spectra differences are observed 1) in varying EEG frequency bands (not only in the spindle frequency range), 2) with regard to different reference sleep stages (NREM stage 2, NREM including all NREM stages, and REM), and 3) concerning different time frames (whole night, first 20 or 30 min of NREM sleep or NREM stage 2 sleep, first or later sleep cycles, 4th NREM episode). This variation is underlined by more recent studies. These results of single studies have not been confirmed by exact replication studies performed by other laboratories.

Most of the human studies have been performed in young subjects and predominantly in males. Since neurophysiological parameters might change with age, it is not known whether CNS effects might differently affect elderly or younger (children and adolescents) subjects. There are some studies indicating that effects might vary with age and gender. In studies, which cover males and females separate analyses should be

performed in order not to miss gender specific effects. Very few of the studies, which include females, state that the menstrual cycle was controlled, a factor, which affects most of the neurophysiological outcome parameters. This requires adequate sample sizes for both males and females. Another aspect is that studies with a parallel group design should be avoided in studies investigating EEG effects. Especially the power spectra of the sleep EEG is known to show a high inter-individual variation and high intra-individual stability (Buckelmüller et al. 2006)) Furthermore it is not known whether subjects with pre-existing medical conditions may be affected differently.

Moreover, most of the provocation studies investigating possible RF-EMF effects on brain activity have used either a commercial mobile phone for exposure or tried to mimic the exposure pattern of typical handheld devices. Almost all of them have reported the maximum SAR over 1 or 10 g but not a detailed SAR distribution. In some cases more detailed information is available in earlier publications of the group. It is well known that different phone models give raise to very different anatomical distributions (Wilén et al. 2003, Deltour et al. 2011). To ensure reproducibility of such studies simulating the use of handset and its effects at least a detailed description of the hard ware and its use (e.g. distance from the head position according to standards etc.) should be provided. A brain region specific SAR distribution would be helpful for the discussion of results.

Conclusions on neurophysiological studies

Most of the recent studies have reported an effect of RF exposure on the spectral power of sleep and the waking resting state EEG. The effects on sleep EEG, however, are not restricted to the spindle frequency range. Furthermore, half of the experimental studies looking at the macrostructure of sleep (especially those with a longer duration of exposure) also found effects, which, however, are not consistent with regard to the affected sleep parameters. It seems that with regard to event-related potentials and slow brain oscillations, results are inconsistent.

There is a lack of data for specific age groups. One study indicates that children and adolescents seem to be less affected.

Overall there is a lack of evidence that RF affects cognitive functions in humans. Studies looking at possible effects of RF fields on cognitive functions have often included multiple outcome measures. Where effects have been found by individual studies, these have typically only been observed in a small number of these outcomes, with little consistency between studies as to which exact outcomes are affected.

The earlier described evidence that RF exposure may affect brain activities as reflected by EEG studies during wake and sleep is further substantiated by the more recent studies. However, the relevance of the small physiological changes remains unclear and mechanistic explanation is still lacking.

3.6.2.3. *In vivo* studies

What was already known on this subject?

The previous Opinion concluded that there were few studies on animals that investigated possible effects of RF exposure on cognitive functions and behaviour, and that there is no evidence from these studies that cognitive functions in animals are influenced by exposure. It was also stated that there is no evidence of direct neurotoxic effects at SAR levels relevant for mobile telephony. At higher SAR levels, activated glial cells were seen in a couple of studies.

What has been achieved since then?

A number of studies on animals have been published since the last Opinion. They range from focus on learning and memory, on behaviour, biochemical brain responses, neurogenesis and cytotoxicity, to neurodegenerative diseases.

<u>Blood brain barrier</u>

Studies of blood brain barrier (BBB) permeability after exposures to permissible RF EMF levels have previously received some interest after findings reported by a Swedish group that suggested increased permeability to albumin in the rat brain during some treatment combinations (Salford et al. 2003; Eberhardt et al. 2008). The change was observed after a 2 h exposure to whole body SARs from 0.01 mW/kg to 0.12 W/kg, and remaining two but not four weeks after exposure. These findings were previously not supported by results from other research groups. Since the last SCENIHR Opinion, three independent studies have been published that were designed to reproduce the conditions employed by the Swedish group. In these "replication" studies (Masuda et al. 2009; McQuade et al. 2009; Poulletier de Gannes et al. 2009) animals (male Fischer 344 rats) were exposed to a 915 MHz GSM signal at whole body SARs between 0.0018 to 20 W/kg, which gave head SARs of 0.14-2 W/kg, for 30 min or 2 h. Assessment was done immediately after exposure or after 2-7 weeks. None of the studies could find any effect of RF exposure on albumin extravasation, number of "dark neurons", or other neurodegenerative markers, whereas the used positive controls caused increased BBB permeability.

In contrast, positive findings were reported by Sirav and Seyhan (2009, 2011) who exposed anaesthetised albino Wistar rats to CW 900 or 1800 MHz radiowaves (at 20 min; SAR-values in the single mW/kg range). In both studies, male rats responded with increased BBB permeability (as shown by Evans blue measurements), whereas female rats were unaffected. Both the gender difference in response and effects at very low SAR-values raise questions regarding the validity of the results. A weakness in the studies is also the use of anaesthesia which brings about relevance issues.

Taken together, the recent studies on BBB integrity do not lend support to that exposure to mobile phone-like RF at SAR-values below or equal to 2 W/kg causes impairment of the BBB. Several of the studies are furthermore done in such a way that their relevance for risk assessment is questionable.

Learning, memory and behaviour

There are some studies addressing RF effects on spatial learning, memory, and behaviour published since the last Opinion. However, several of these studies are not possible to evaluate, or not performed in such a way that they can be considered to be of sufficient quality for risk assessment.

A study with some relevance was published by Hao et al. (2012) where male Wistar rats experienced a transient negative effect of exposure on a spatial memory task. The exposure was to a 916 MHz CW RF field, 10 W/m^2 (no SAR values are given) (six h exposure per day; five days a week; ten weeks). Compared to controls (no sham exposure), exposed animals displayed impairment in completion of a spatial memory task in the middle of the exposure period, whereas values were comparable between the two groups at the end of the trial. Implanted micro-electrode arrays (into the hippocampus) in one control and one exposed animal indicated changes in electrophysiological parameters in the exposed brain.

An interesting study was published by Hirata and co-workers (Hirata et al. 2010). Their aim was to determine at what whole-body SAR value thermal stress-related behaviour was induced in rabbits exposed to 2.45 GHz in a range of ca 100-1000 W/m^2. The rabbit is highly susceptible to heat stress and an appropriate model organism for these kinds of studies. A core body temperature increase of ca 1°C was sufficient to induce thermal stress behaviour in some, but not all animals. The threshold for onset of behavioural thermal stress was at approximately 110 W/m^2, which corresponds to a whole body average SAR of 1.3 W/kg.

A study on Wistar rats exposed to UMTS signals (0, 2 and 10 W/kg SAR) for a period of 120 minutes showed no differences at an exposure of 2 W/kg from the sham-exposed group in hippocampal derived synaptic long-term potentiation (LTP) and long-term depression (LTD), indicators of memory storage and memory consolidation. In contrast, at 10 W/kg, significant reductions of LTP and LTD were observed (Prochnow et al. 2011). The authors conclude that UMTS exposure at a rate of 2 W/kg is not harmful to markers

for memory storage and memory consolidation. At higher exposures, however, effects occur that can be distinguished from the stress-derived background.

Maaroufi et al. (2014) studied whether combined 900 MHz exposure (probably CW) for 1 h/day during 21 consecutive days and iron overload (which is neurotoxic and can contribute to learning deficits etc.) influenced the outcome of spatial cognitive tasks, neurochemistry, and oxidative stress. Calculated SAR-values in the male one-month-old Wistar rats were 0.05-0.18 W/kg (depending on position within the field). The testing took place after that EMF exposures and/or iron administration was completed. A proper sham conditions was not in place. It is unclear if any blinding procedures were adopted. In summary, the EMF treatment caused impairment of the object exploration task but not in the other behaviour tests. Some changes in dopamine levels in certain brain regions were noted, but not in all parts of the brain. There were no consistent effects on parameters related to oxidative balance in the brain. The iron overload did not exacerbate the effects of EMF exposure.

Hao et al. (2012) investigated the effects of a CW 916 MHz RF EMF on spatial learning and memory in Wistar rats. The animals were exposed for 6 h/day, 5 day/week, 100 weeks, to a 10 W/cm^2 field (in the middle of the cage). Once per week during the exposure period, the completion time, number of total errors, and neuron discharge signal (implanted microelectrode arrays in the hippocampus) were recorded while the rats were searching for food in a radial maze. A transient negative effect on performance was seen during weeks 4-5. The authors speculated in an adaptation to the long-term exposure. Unfortunately, this interesting study lacks proper dosimetry. Another study on memory and behaviour in Wistar rats was published by Junior et al. (2014). No effects on anxiety patterns or working memory were observed in study where male rats were exposed to a 1.8 GHz GSM-like signal for 3 days. Once again, this study is lacking proper dosimetry, so its relevance for risk assessment is not possible to ascertain.

Another possible transient effect on memory was reported by Ntzouni et al. (2013). Unrestrained mice (C57BI/6J) were exposed for 66 or 148 days (90 min/day) to a 1.8 GHz signal (SAR 0.11 W/kg). The animals displayed impairment in an object recognition and an object location task immediately at the end of exposure. Four weeks later, without exposure, the exposure effect was absent.

Memory impairment in Wistar rats was reported by Wang et al. (2013) together with morphological effects on neuronal structures in the hippocampus. The exposure was to a 2.856 GHz RF signal at 0, 5, 10, and 50 mW/cm^2 (for 6 min). The two higher exposure levels were associated with effects on spatial learning and memory at 6 h, 1 day, and 3 days after exposure.

Sharma et al. (2014) reported a negative effect on spatial memory performance after a 2 day training period in Swiss albino mice. The exposure (10 GHz; 0.25 mW/cm^2; 0.18 W/kg) lasted 2 h/day for 30 days. The authors furthermore reported a concurrent decrease in protein synthesis in the animal´s brains.

Improvement in motor function was seen in a study from Odaci et al. (2013) on female Sprague-Dawley rats. Pups were exposed in utero (days 13-21 of pregnancy) and investigated on postnatal day 32. Exposure was to a 900 MHz signal, at 10 V/m. No SAR-values were reported and the number of animals was very low (3 in each group, control and exposed).

Cognitive impairment (as well as oxidative stress and inflammatory markers) were reported in a study on male Fisher rats (900/1800 MHz; 0.6 mW/kg; 2 h exposure/day; 30 days) (Megha et al. 2012). Lu et al. (2012) reported negative effects on spatial learning and memory in a study on Wistar rats (2.45 GHz; 1 mW/cm^2; 3 h/day; 30 days). The authors reported that effects were reversed by systemic glucose treatment.

In summary, although some of the studies reported here suggest an effect at non-thermal levels on learning, memory or behaviour, any conclusive evidence cannot be drawn at present. Results are to some extent contradictory, and there remain significant

question marks regarding exposure, blinding, proper controls, and dosimetry in many studies.

Neurogenesis and cytotoxicity

There are some recent studies that suggest cell loss in certain brain areas after RF exposure at levels below the exposure guidelines. Thus, Bas et al. (2009) and Sonmez et al. (2010) exposed female Wistar Albino rats during weeks 12-16 (1 h/day for 28 days) to a 900 MHz continuously modulated RF field. The authors report that the output power from the signal generator was 2 W (peak), causing 10 W/m^2 in power density. During exposure, animals were restrained in a cylindrical tube, where the modelled SAR amounted to 0.016 (whole body) and 2 W/kg (head) respectively. Sham exposed animals were kept in a similar contraption, without RF exposure. The SAR-values in the investigated parts of the brain were not calculated. The total pyramidal cell number in the hippocampus (Bas et al. 2009) and the Purkinje cell number in the cerebellum (Sonmez et al. 2010) were significantly decreased in the exposed animals. The same animals (n=6 for both sham and exposed groups) were used in both these studies, that furthermore did not find any exposure-related effects on body or brain weight.

Newborn (postnatal day 7, P7) and young adult (P28) Wistar rats were used in a study by Oredacova et al. (2011). The animals were exposed to a 2.45 GHz (average power density 20 – 67 W/m^2) for 2 h, followed by a 2 h post-exposure period before euthanasia. Markers for proliferation were investigated by immunohistochemistry (semiquantitative evaluation) for the immediate-early response gene c-fos and for NADPH-diaphorase. This short exposure duration resulted in increased c-fos levels in the subventricular zone in P7 rats and increased NADPH-diaphorase staining in the rostral migratory stream in P7 rats. Based on morphology, exposed rats displayed a younger phenotype at P28 than controls. The results are contradictory and the methodology including exposure description render the study unsuitable for any further conclusions.

Caballo-Quintas et al. (2011) analysed expression of c-fos and the glial marker GFAP in several brain regions in normal and picrotoxin-treated (prone to undergo seizures) adult male Sprague-Dawley rats. Animals were i.p. injected with sub-convulsive doses of picrotoxin immediately prior to exposure of immobilized (plastic tubes) rats. The exposure was to a 900 MHz RF for 2 h, yielding an estimated peak SAR in the brain of 1.5-1.6 W/kg. Animals were sacrificed at different time periods after exposure (90 min, 24 h, 72 h) followed by immunohistochemical staining of several brain regions. The results show immediate (90 min post exposure) increase in the number of c-fos positive cells in neocortex and paleocortex in exposed and picrotoxin-treated animals, which persisted until three days after exposure. The levels of GFAP increased with time in exposed and picrotoxin-treated animals. The study suggests that the epileptic brain could be more sensitive to RF exposure, leading to glial cell activation.

Neurodevelopment from a functional point of view was studied by Aldad et al. (2012) who exposed mice in utero and investigated them as adults for certain behavioural traits and electrophysiological characteristics. Exposure is poorly described but is reported to be to a muted telephone (900-1800 MHz) during the entire gestation period. After blinded investigations, the authors concluded that exposed animals displayed hyperactivity, memory deficiencies, decreased anxiety, and impaired glutamatergic transmission. Although the study employs relevant biological end-points, it cannot be used for any conclusions regarding pre-natal mobile phone exposure and functional development of the brain.

These studies indicate some neurotoxic effects (reduced neuronal cell number, glial cell activation) after exposure for several days to RF fields at SAR-levels below 2 W/kg. Additional studies with better dosimetry are needed before any firm conclusions can be drawn. Additional studies on early development as well as the effects on the pathologic brain are also justified.

Neurodegeneration

Ammari et al. (2010) have documented increased GFAP expression, and thus glial cell activation after exposures at 1.5 and 6 W/kg in rats. Male Sprague-Dawley rats were exposed to a 900 MHz EMF, modulated at 217 Hz (five days/week; eight weeks). Animals were then sacrificed three or ten days after exposure and brain sections analysed for GFAP expression by means of immunohistochemistry. Performed SAR calculations (phantom modelling) showed that animals were exposed to either 1.5 W/kg (45 min/day) or 6 W/kg (15 min/day). Both exposure regimes caused significantly increased levels of GFAP in the investigated regions after three and ten- days post exposure. In almost all cases, the effects were more pronounced in animals exposed to 6 W/kg. The conclusion of this study is that RF exposure may activate glial cells, in particular astrocytes. This is a typical marker for damage to the CNS and appears independent of injury agent.

In contrast, studies from the Arendash group (Arendash et al. 2009, 2010), suggest that RF exposure (GSM-like signal, 918 MHz, SAR 0.25 – 1.05 W/kg) of mice (normal or transgenic; mixed strain background) provided a protective effect against Alzheimer´s disease (AD) development. The transgenic mice (Tg mice) were engineered to over-express the proteins Aβ and PS1 and thus easily develop the neurodegeneration typical for AD. In Arendash et al. (2009), both normal and transgenic litter mates were daily exposed (2 h) for up to more than six months to the RF. For both types of mice, beneficial cognitive effects were noted after exposure, and in the case of Tg mice, the disease process was reversed to some extent. These animals were exposed for various time periods from the age of five months up to 13.5 months of age. A more recent study (Arendash et al. 2012) employed older animals (21-27 moths) that were exposed for two months. Also here, improved memory capacity (in the Y-maze test) was noted, in both normal and transgenic diseased animals. The authors showed that the treatment did not cause increased brain temperature, slightly increased body temperature, and reduced the blood-flow in the cerebral cortex.

Despite the commendable approach in using Tg mice and the overall good quality in the biological parts of the study, it is necessary to replicate these results using an improved design and larger groups. The studies by Arendash et al. suffer gravely from their complete lack of dosimetry. The authors have erroneously calculated the SAR values for the exposed animals by directly using the measured values of the external electric field. In the formula for SAR calculation it is the internal electric field that should be used and this is not easily obtained from just a value of the external field.

The mentioned studies show results that are contradictory in terms of RF effects on neurodegeneration. Increased GFAP staining would indicate activated glial cells and thus increased risks for neurodegenerative processes, whereas the other studies suggest that a disease process can be reversed. Additional studies conducted by independent laboratories that try to replicate and extend these findings are necessary to reconcile the different outcomes.

Oxidative stress

End-points related to stress and oxidative stress have been investigated in several recent studies, with different outcomes. Ait-Aissa and co-workers (2013) analysed several stress markers (3-nitrotyrosine, HSP25, HSP70) in brains of young rats exposed to a 2450 MHz Wi-Fi signal in utero (day 6-21 of gestation) or as newborns (up to 5 weeks). Exposures were done 2 h daily (blinded conditions) at 0, 0.08, 0.4 and 4 W/kg. None of the exposure conditions yielded results different from the sham conditions.

Sasdag et al. (2012) studied long-term effects of 900 MHz in adult male Wistar rats. The animals were exposed 2 h/day (7 days/week) for 10 months in a carousel configuration. Levels of amyloidβ, malondialdehyde, and protein carbonyl were analysed, and only the latter was affected (higher levels in exposed than in sham controls). Oxidative stress effects were also seen in a study from Dashmuk et al. (2013) on Fisher rats exposed to 900 MHz (80 μW/kg SAR; 2 h/day; 5 days/week). Both malondialdehyde and protein carbonyl levels were increased in exposed animals. Eser et al. (2013) employed adult

male Wistar rats in their study on effects of 900, 1800, and 2450 MHz (1 h exposure/day; 2 months; 1.04 W/kg whole body SAR). The total antioxidative capacity and oxidative stress index levels were affected (decreased and increased respectively) in several structures of the brain.

In utero exposure (0.9 W/kg; 10 min exposure/day during the entire gestation period) of foetal rat brain (restraining conditions) caused decreased antioxidant (SOD and GSH-Px) and increased malondialdehyde levels in a study by Jing et al. (2012). Also Naziroglu et al. (2012) observed oxidative stress in rat brains exposed to 2.45 GHz, which was counteracted by administration of the antioxidant melatonin.

Taken together, several studies suggest that RF exposure in in rodents can cause oxidative stress effects. The studies are however often lacking in proper dosimetry and do not include proper positive controls. The magnitude of the changes are modest, and their biological significance unclear.

Other effects

Maskey and co-workers (Maskey et al. 2010, 2012) have focused on RF-exposure effects on Ca^{2+}-binding proteins in the mouse hippocampus. In both these studies, animals were exposed to an 835 MHz signal (whole body average SAR 1.6 or 4 W/kg) for various time periods. During exposure, animals were non-restrained. Three hours after the last exposure, animals were sacrificed and the brains prepared for immunohistochemical staining for calbindin, calretinin, or GFAP (only in Maskey et al. 2012). In the first study, six week old male ICR mice were exposed for 1 h (5 days), 5 h (1 day), or 1 h for 28 days (only at 1.6 W/kg). Compared to controls (it is unclear if real sham conditions were employed), several significant changes in immunoreactivity in different hippocampal regions were seen. However, the changes followed no consistent pattern, and no dose-response pattern was seen. The more recent work (Maskey et al. 2012) used a similar experimental approach, with the modification that GFAP was also investigated, and a more specific cell type analysis in specific hippocampal regions was made. In addition the exposure was for 8 h/day, one month. Calbindin and calretinin immunoreactivity decreased at both SAR-levels in the CA1, CA3, and dentate gyrus regions. Effects on GFAP levels were more equivocal, increasing only at 1.4 W/kg in CA1 and CA3 and at only 4 W/kg in dentate gyrus. The papers thus report changes in levels of certain Ca^{2+}-binding proteins, but in an inconsistent way. There is furthermore no consistent effect on GFAP expression. A more recent work (Maskey et al. 2014) found decreased levels of the neurotrophins BDNF and GDNF in several cerebral nuclei.

Possible effects on stress hormones (ACTH, corticosterone) and hippocampal memory storage and consolidation (LTP and LDP) on male Wistar rats were investigated by Prochnow et al. (2011). Six restrained rats inside a spherical sector waveguide were simultaneously exposed (2 h) to either 0 W/kg, 2 W/kg or 10 W/kg (which does not cause a temperature increase >0.1 ^{o}C in the rat brain). Blinded conditions were applied and measures were taken to minimize stress to the animals. All exposure conditions (including sham) significantly increased ACTH and corticosterone levels compared to the cage control. The only significant difference to sham was noted for corticosterone in the animals exposed to 10 W/kg. Also regarding LTP and LDP, all exposures were different from cage control values. Exposure to 10 W/kg was also significantly different from sham and 2 W/kg for both LTP (decrease), and LDP (increase) suggesting a possibility that high SAR-values impair hippocampal memory capacity.

Also, Bouji et al. (2012) focused on a single short exposure (15 min to 900 MHz GSM-signal, 6 W/kg) of rats. Markers for glial activation (GFAP), inflammation (IL-1β, IL-6), stress (corticosterone) and emotional memory in six-week-old and 12 month old male Sprague Dawley rats were investigated. The only noted effects were increased corticosterone levels in young rats, and enhanced emotional memory and increased IL-1β levels in the olfactory bulb in the older animals.

In two recent studies in rats, Pelletier et al. have investigated the effects of constant RF exposure (900 MHz continuous wave, 1 V/m, 5 weeks) on thermal related behaviour and

sleep pattern. In the first study (Pelletier et al. 2013) control of body energy homeostasis (feeding behaviour, sleep, thermoregulation) was studied in juvenile male Wistar rats. The obtained results suggested that the exposure caused increased constriction of blood vessels, and increased food intake, but without any effects on sleep pattern. In the second study from the same group (Pelletier et al. 2014), the same rat model and exposure conditions were used for thermal preference and sleep stage distribution studies. Exposed animals shifted their thermal preference after the exposure period towards higher temperatures. Also certain sleep parameters (duration, SWS frequency) were increased in the exposed animals, whereas the PS was unaffected.

Effects on body mass (decrease) in rats was observed by Sokolovic et al. (2012), whereas Kim et al. (2013) did not observed any effects in brain glucose metabolism (915 MHz RFID signal; 4 W/kg; 8 h/day; 5 days/week; 2 or 16 weeks).

No consistent pattern in monoamine transmitter levels in several brain regions could be seen in a study by Abould Ezz et al. (2013). Their exposure of adult male Wistar rats (1800 MH; 0.8 W/kg; 1, 2, 4 months of 1 h daily exposure) displayed various levels of dopamine, norepinephrine, and serotonin in the hippocampus, hypothalamus, midbrain, and medulla oblongata in exposed compared to control animals. However, no specific pattern can be obtained from the data.

In a study from Razavinos et al. (2014) electrophysiological properties of CA1 hippocampal neurons were investigated in Wistar rats exposed as foetuses (900 MH; 6 h/day). A decrease in neuronal excitability was seen.

A gene expression analysis based on a cDNA microarray was performed by Yang et al. (2012). Adult male Sprague-Dawley rats (restrained) were exposed to a 2.45 GHz RF field (0 W/kg, 6 W/kg). MRNA from the hippocampus showed 23 up- and 18 down-regulated genes after the 6 W/kg exposure. This included the stress response genes for hsp27 and hsp70, which was further confirmed by RT-PCR, immunohistochemistry, and Western blot analysis.

Conclusions on *in vivo* effects

A number of different end-points have been studied at various SAR-levels in both mice and rats. Although some positive findings are noted, they are inconsistent and appear mostly at levels well above guideline values. There is however a need to replicate certain of the studies, and also to perform studies at more stringent conditions (exposure and dosimetry, blinding, controls).

3.6.2.4. *In vitro* studies

What was already known on this subject?

There was no specific reference to any relevant *in vitro* studies on this subject in the previous Opinion.

What has been achieved since then?

There are only few *in vitro* studies published in this area, and their relevance for an assessment of effects on the nervous system is limited. Some studies related to neurodegenerative diseases (NDD) have nevertheless been published. The rationale behind these papers has been that one feature often involved in NDD is activation of microglia and/or astrocytes, which will cause changes in radical homeostasis and subsequent cellular stress. Also, different viability related end-points in both neurons and glial cells have been investigated.

Del Vecchio et al. (2009) exposed a cholinergic cell line and primary cultures of rat cortical neurons to a 900 MHz signal (1 W/kg; up to 144 h). There were no effects on cell proliferation or viability from this exposure. A co-exposure of RF with H_2O_2 potentiated H_2O_2 induced cell death in the cell line, but not in the primary cultures. Co-exposures to RF and amyloid-β or glutamate did not exert any additive or synergistic effect to exposures to the chemicals. Viability was also investigated by Campisi et al. (2010) who

exposed primary rat astrocytes to 900 MHz CW or 900 MHz amplitude modulated at 50 Hz. Exposures were for 5, 10, or 20 minutes, at 10 V/m. None of the exposure conditions had any effects on viability. The only noted effect was that a 20 min modulated RF exposure caused ROS and DNA fragmentation (Comet assay) increases.

Endpoints related to survival and cell death was also investigated in studies by Liu et al. (2012) and Zeni and co-workers (2012). The former study found that primary rat astrocytes, but not C6 glioma cells, were induced to undergo Caspase-3-dependent apoptosis after exposure to a 1950 MHz TD-SCDMA EMF at a SAR-value of 5.36 W/kg for 48 h. The study by Zeni et al. used a similar exposure protocol (1950 MHz UMTS signal; 10 W/kg; 24 h) where PC12 rat phaeochromocytoma cells were exposed. End-points studied included DNA integrity, cell viability and apoptosis, directly after the exposure or after 24 h post exposure. None of the end-points at none of the investigated time points were affected due to the exposure.

Signs of oxidative stress due to RF exposure at 1800 MHz (modulated at 217 Hz; 2 W/kg; 24 h exposure) were seen in a study by Xu et al. (2010) who noted increased levels of 8-hydroxyguanine (8-OHdG) in primary rat cortical neurons. The effect level of RF was comparable to the effects of the positive control H_2O_2, and counteracted by melatonin, suggesting that the exposure is causing DNA damage via oxygen radical production.

A paper from Sakurai et al. (2011) adopted a microarray gene expression analysis approach, where human SVGp21 glial cells were exposed to a 2.45 GHz CW signal (1, 5, 10 W/kg; 1, 4, 24 h). The microarray analysis yielded 23 assigned gene spots, but subsequent qRT-PCR could not confirm any effects on gene expression.

Possible microglia activation by RF exposure has been studied in a few papers. Work from Hao et al. (2010) and Yang et al. (2010) employed the N9 mouse glia cell line and exposed the cells to a 2.45 GHz pulsed EMF (2 µs pulse width; 500 pps pulse rate; 20 min exposure; 6 W/kg). The results consistently show indicators of microglia activation (including CD11b activation, NO release; induction of iNOS and TNF-α; JAK1/JAK2 expression; phosphorylation of STAT3 and JAK1/JAK2). Any possible microglia activation was not studied at lower SAR-values. A similar line of investigation was published by Hirose et al. (2010), who exposed primary rat microglia to a 1950 MHz W-CDMA signal (0.2, 0.8, 2.0 W/kg; 2 h). There were no signs of microglia activation (inflammatory cytokines) after exposure.

A study on mouse retinal ganglion cells (Ahlers and Ammermüller 2013) focused on responses of the cells to light stimuli. Cells were investigated before, during, and after exposure (GSM 900, GSM 1800; UMTS) and up to 35 min after exposure. No consistent effect of RF exposure on light responses was seen.

Moretti et al. (2013) used cortical neuronal cultures on microelectrode arrays to investigate possible effects on neurotransmission. Exposure was to a GSM-1800 signal (3 min) at 3.2 W/kg. This pilot study indicated some effects of the exposure in the form of decreased firing rate and burst rate in the cultures.

Primary rat cortical neurons were studied by Zhang et al. (2013). Cultures were obtained from newborn Sprague-Dawley rats and exposed to 2.45 GHz (10 min). Cell viability was decreased in exposed cells (4 W/kg), with signs of apoptosis.

Stress protein expression and apoptosis markers were investigated by Calabro et al. (2012) in the human neuroblastoma cell line SH-SY5Y. Cells were exposed for 2 and 4 h to 1800 MHz. Results did not indicate any consistent pattern, with levels increasing, decreasing, or remaining unchanged.

Conclusions on *in vitro* studies

The few available *in vitro* studies are not providing data useful for assessment of possible effects on the nervous system function or on disease processes in the nervous system.

3.6.2.5. Conclusions on nervous system effects and neurobehavioral disorders

Although the Danish National Birth Cohort study has reported results that suggest higher prevalence of some behavioural and health disorders in children whose mothers have been mobile phone users, these findings have not been confirmed in other studies. In general, the published epidemiological studies have methodological weaknesses.

Recent epidemiological studies have not shown increased risks of neurological disease related to RF exposure.

Regarding neurophysiological studies, the conclusions from the previous SCENIHR Opinion that RF exposure may affect brain activities as reflected by EEG studies during wake and sleep is confirmed by the more recent studies. The relevance of the small physiological changes remains unclear and mechanistic explanation is still lacking.

Overall there is a lack of evidence that RF affects cognitive functions in humans.

A number of different end-points have been studied at various SAR-levels in both mice and rats. Although some positive findings are noted, they are inconsistent and appear mostly at levels well above guideline values. There is however a need to replicate certain of the studies, and also to perform studies at more stringent conditions (exposure and dosimetry, blinding, controls).

The few available *in vitro* studies are not providing data useful for assessment of possible effects on the nervous system function or on disease processes in the nervous system.

3.6.3. Symptoms

What was already known on this subject?

One of the more common health concerns associated with RF exposure is the onset of short-term symptoms such as headaches, fatigue and dizziness. Identifying whether RF exposure can cause these symptoms has attracted a substantial amount of research. As well as assessing these effects in the general population, the existence of a group of people who report being particularly sensitive to various forms of electromagnetic fields including RF fields has also been of special interest. Due to the lack of evidence for a causal relationship this phenomenon is called ‘idiopathic environmental intolerance attributed to electromagnetic fields’ (IEI-EMF) (Hillert, 2004). However it is also more commonly referred to as ‘electromagnetic hypersensitivity’ or ‘electrosensitivity.

The 2009 Opinion noted that several studies had tested the association between RF exposure and the onset of symptoms. These included studies relating to both the general public and to people with IEI-EMF. Although some studies had reported an association between individual symptoms and RF EMF exposure, there was no consistency in these findings. In addition, although multiple studies were found which tested whether participants could tell when they were being exposed to RF EMF, none had found that participants were reliably able to do this. The Opinion therefore noted that “the conclusion that scientific studies have failed to provide support for an effect of RF on symptoms still holds.”

What has been achieved since then?

3.6.3.1. Provocation studies

Since the last Opinion was published, an additional paper has appeared (Lowden et al., 2011) which contains more data from a study included in the 2009 Opinion (Hillert et al., 2008). This double-blind experimental provocation study exposed participants with and without IEI-EMF to an 884 MHz GSM signal (time averaged 10g psSAR of 1.4 W/kg) for three hours on one day and to a sham condition for three hours on another day. The new paper reports the effects of these exposures on the quality of the participants’ subsequent sleep, including measures of subjective fatigue, arousal, sleepiness and sleep quality. No effects of exposure were observed for any subjective outcome.

Fifteen new experimental provocation studies have also been published since the last Opinion. These are summarised in Table 16. Five of these included participants with IEI-EMF, and all but two of them (Nam et al., 2009; Leitgeb et al., 2008) described using a double blind protocol. Twelve of the studies assessed exposures that were designed to emulate those that might be received from a mobile phone or radio handset during a relatively long call (30 minutes to 3 hours) (Choi et al., 2014; Croft et al., 2010; Curcio et al., 2009; Kwon et al., 2012; Loughran et al., 2012; Nakatani-Enomoto et al. 2013; Nam et al., 2009; Nieto-Hernandez et al., 2011; Riddervold et al., 2010; Schid, Murbach et al., 2012; Schmid, Loughran et al. 2012; Spichtig et al., 2012). Two studies observed a significant effect of their exposures. First, Curcio et al. (2009) asked fifteen participants to score each of ten symptoms before and after exposure to a sham condition and a GSM 902.4 MHz signal generated by a mobile phone positioned near to the participant's head. After discarding data from four participants because of "technical problems," a marginally significant ($p=0.04$) increase in headache ratings was observed, but in the sham condition rather than the GSM condition. Second, Nieto-Hernandez et al. (2011) exposed 60 police officers with IEI-EMF and 60 without the condition to 50 minutes of sham exposure, 50 minutes of exposure to a signal emulating that produced by a TETRA handset and 50 minutes of exposure to a continuous wave signal. Unexpectedly, the continuous wave signal was associated with a decrease in itching sensations, an effect which was observed only among the IEI-EMF group. Despite testing a range of subjective sensations, none of the other handset-related studies identified any significant effects of exposure.

Two provocation studies assessed the effect of exposures associated with mobile phone or radio base stations. Wallace et al. (2010), exposed participants with IEI-EMF and healthy control participants to TETRA base station and sham exposure conditions. After being exposed to both conditions in an initial non-blind session, 48 participants with IEI-EMF and 132 without IEI-EMF were exposed under double-blind conditions to four brief exposures (two 'on' and two 'off') and two 50 minute exposures (one 'on' and one 'off'). Sixty-three symptoms were assessed at the end of each exposure. Under non-blind conditions, the participants and particularly those with IEI-EMF reported significantly greater symptoms during the TETRA exposure than during the sham exposure. When tested under double-blind conditions, however, these effects were no longer apparent.

In an attempt to assess longer-term exposure to base station signals, Danker-Hopfe et al. (2010) travelled to 10 villages in Germany where there was no mobile phone service, only weak fields from other RF sources and no on-going discussion about the potential health risks of EMF. In each village, all adult members of every household were invited to participate in their study. Over the course of ten nights, participants recorded their sleep quality while at home, using a standardised questionnaire (other outcomes are summarised in section 3.6.2.2). During five of these nights, the research team used their own experimental base station to transmit combined GSM 900 MHz and 1800 MHz signals in the village. The base station was set to a test mode to ensure that the signal did not register on any mobile phones in the village. The other 5-night period was used as the control condition. 365 participants completed the study, under double blind conditions. No effects of exposure were observed for any subjective measure of sleep quality.

Finally, one additional study by Leitgeb et al. (2008) assessed whether shielding people from electromagnetic fields during the night would have any beneficial effects on their sleep. 43 volunteers who regularly experienced sleep problems which they attributed to RF-EMF were asked to sleep at home for three 3-night periods. During one of these periods, participants slept within a Faraday cage designed to protect them from RF-EMF exposure. During another period, participants slept within a placebo cage which looked similar but lacked the shielding properties. The third period involved no cage and acted as a control condition. Objective and subjective measures of sleep quality were recorded in this single-blind experiment. Although three of the volunteers did display positive effects as a result of sleeping within the genuine cage, the authors subsequently discovered that all three had broken their blinding by checking which condition blocked

RF-EMF and cautioned that "no reliable conclusion can be drawn from… these three volunteers."

The results of these individual studies, which have typically not found any effect of exposure to radiofrequency fields on self-reported symptoms, are supported by a series of meta-analyses conducted by Augner, Gnambs, Winker and Barth (2012). These authors identified nine single- or double-blind provocation studies which assessed the effects of GSM exposure on five self-reported symptoms (headache, nausea, dizziness, fatigue and skin irritation) and which were suitable for inclusion in a meta-analysis. No evidence was found in the meta-analyses that any of these end-points were affected by exposure.

One additional double-blind experiment used a different paradigm from those detailed above to assess whether exposure to radiofrequency fields affects pain tolerance thresholds (Vecsei et al. 2013). Twenty two students (mean age 22, standard deviation 2.65, 10 female) were exposed to a UMTS 1947MHz signal (maximum SAR average over 1g=1.75 W/kg and over 10g=0.73W/kg) or a sham condition for 30 minutes on separate days. Participants were exposed to thermal stimuli of their index fingers over four blocks of trials (two blocks of trials during each exposure and two blocks after each exposure, with each block consisting of six trials). For each trial, participants were instructed to move the relevant finger away from a heating pad as soon as the heating stimulus was perceived as painful. The time taken to move the finger was used as an objective measure of pain threshold. After each block of trials, participants also provided a subjective assessment of how painful the stimuli were. For the blocks of trials that occurred during the exposure, a non-significant ($p=0.09$) 'device x trial' interaction was observed, with the expected desensitisation of fingers to heat stimuli over time being slightly stronger in the UMTS condition than the sham condition. A significant three-way 'side x device x trial' interaction was also observed, suggesting that the desensitization interaction only occurred for the index finger that was contralateral to the exposure. Analysis of the subjective pain data also suggested a significant effect, with pain ratings increasing between blocks (i.e. over time) during sham exposure, but remaining steady during UMTS exposure. The use of a behavioural indicator of pain threshold in this study represents a novel step in this field. Whether the findings of this study, which had a good methodological design, can be replicated remains to be seen.

Eleven of the studies described in Table 16 have also tested whether people are able to tell whether or not they are being exposed to RF (Choi et al., 2014; Kwon et al., 2012; Nakatani-Enomoto et al. 2013; Nieto-Hernandez et al., 2011; Wallace et al., 2010; Nam et al., 2009; Croft et al., 2010; Riddervold et al., 2010; Schmid, Murbach et al., 2012; Schmid, Loughran et al. 2012; Spichtig et al., 2012). In addition, one further study from Iran tested this ability in 20 students who reported symptoms which they attributed to their mobile phone (Mortazavi et al., 2011). None of these studies has found any evidence that participants are able to make this discrimination, a result which holds true both for people with IEI-EMF and for those without it. Additionally, the meta-analyses conducted by Augner and co-workers (2012) pooled the results from seven double-blind studies which assessed people's abilities to detect radiofrequency fields, but without finding any evidence of such an effect. A second meta-analysis by Röösli, et al. 2010) pooled the results of four double-blind provocation studies, and also observed no evidence that people with or without IEI-EMF were able to correctly discriminate between conditions.

Table 16. Provocation studies with symptom outcomes

Authors	Sample	Signal type	Exposure duration	Effects of exposure
Choi et al., 2014 (HS)	26 adults (mean age 28.4 plus or minus 5.1yr; 13 female) and 26 teenagers (mean age 15.3 plus or minus 0.7yr; 13 female)	1950MHz WCDMA, averaged peak spatial SAR over 1g = 1.57W/kg.	Two 32 min exposures (WCDMA and sham).	No effects on eight symptoms.
Croft et al., 2010 (HS)	41 adolescents (mean age (sd) 14.1 (0.87), 20 female); 42 young adults (24.5 (4.51), 21 female) and 20 elderly (62.2 (3.94), 10 female)	895MHz GSM, 1900MHz GSM. Maximum peak SAR averaged over 10g=0.7 W/kg (895 MHZ) and 1.7 W/kg (1900 MHz).	Three 50 min exposures to 895 MHz 1900 MHz and sham.	No effect of 3G exposure on mood in adolescents or the elderly. Activation (psychological arousal) greater during 3G exposure in young adults (t[41]=2.06, p=0.046), though this did not reach the Bonferroni-corrected critical value for significance. No effect of 2G exposure on mood in any group
Curcio et al., 2009 (HS)	11 healthy participants (mean age 20.9, 20 to 23, all female)	902 MHz GSM. Maximum SAR averaged over 10g = 0.5 W/kg	Two 40 min exposures (GSM and sham).	No effect of exposure on any subjective outcome except for headache (F1,10=5.46, p=0.04) which was increased in the sham condition.
Kwon et al., 2012 (HS)	20 healthy participants (mean age29.4, plus or minus 5.2, 9 female) and 17 participants with IEI-EMF (mean age 30.1 plus or minus 7.6, 9 female)	1950 MHz<br>WCDMA exposure (1950 MHz). Peak SAR 1g=1.57 W/kg.	Two 32 min exposures (WCDMA and sham).	No effect of exposure on symptoms in either group, and no evidence that participants in either group could detect the exposure.
Loughran et al. 2012 (HS)	20 healthy volunteers (mean age 27.9, range 20 to 51, 13 female)	894.6 MHz GSM. SAR averaged over 10g=0.67 W/kg	Two 30 min exposures (GSM and sham).	No evidence of any effect of exposure on self-reported sleepiness, or any differential response when participants were categorised as "increasers" or "decreasers" based on EEG responses to exposure
Nakatani-Enomoto et al. 2013 (HS)	19 healthy volunteers (mean age 30.6, 22 to 39: 7 female)	1950MHz WCDMA, maximum 10g SAR in head: 1.52 W/kg, in brain: 0.13 W/kg.	Two 3hr exposures prior to sleep (W-CDMA and sham)	No effect of exposure on sleepiness measured the following morning. No evidence of ability to discriminate between conditions.
Nam et al., 2009 (HS)	18 participants with IEI-EMF (mean age 26.1 (3.4), 10 female) and 19 healthy participants (mean age 25.0 (2.3), 9 female)	835 MHz CDMA. Spatial peak SAR averaged over 1 g=1.22 W/kg, based on manufacturer's data.	Two 30 min exposures (CDMA and sham).	No effect of exposure on symptoms
Nieto-Hernandez et al.,	60 healthy participants (mean age 38.2 (8.0), 10	385 MHz TETRA, CW. Maximum SAR averaged over 10 g	Three 50 min exposure (TETRA, CW and	Reduced sensations of itching in the IEI-EMF participants in response to the continuous

2011 (HS)	female), and 60 participants with IEI-EMF (mean age 35.6 (7.4), 7 female)	= 1.3 W/kg	sham)	wave exposure (p=0.03). No other effects were found for symptoms.
Riddervold et al., 2010 (HS)	53 healthy emergency service personnel (mean age 36.4, 25 to 49, all male)	420 MHz TETRA. Peak SAR averaged over 10 g = 2 W/kg	Two 45 min exposures (TETRA and sham).	No significant effects on any self-reported symptoms.
Spichtig et al., 2012 (HS)	16 healthy participants (mean age (sd): 26.8 (3.9), all male)	UMTS with maximum peak averaged over 10 g SAR of 1.8 W/kg, UMTS with maximum peak SAR of 0.18 W/kg.	Three 31 min exposures (1.8 W/kg, 0.18 W/kg and sham)	No effect of exposure on subjective tiredness or well-being.
Schmid, Murbach et al., 2012 (HS)	25 healthy volunteers (mean age 23.2, range 20 to 26, all male)	GSM 900 MHz (SAR 10 g: 2 W / kg)	Three 30 min exposures (2 W/kg, pulsed magnetic field and sham)	No effects of exposure on mood, well-being or sleep quality
Schmid, Loughran et al. 2012 (HS)	30 healthy volunteers (mean age 23.0, range 20 to 26, all male)	GSM 900 MHz (SAR 10g: 2 W / kg) pulse modulated at 14 Hz or at 217 Hz	Three 30 min exposures (14 Hz, 217 Hz and sham)	No effects of exposure on mood, well-being or sleep quality
Danker-Hopfe et al., 2010 (BS)	365 healthy participants recruited from 10 villages with no pre-existing mobile phone coverage (mean age 45.0, range 18 to 81, 186 female)	900 and 1800 MHz GSM 0.01 to 0.9 V/m.	Ten nights of exposure to either real or sham conditions	No effects on self-reported sleep quality.
Wallace et al., 2010 (BS)	51 participants with IEI-EMF (mean (SD) 42 (16); 61% female) and 132 healthy controls (41 (19); 51% female)	420 MHz TETRA. Approximated SAR = µ271 W/kg	Four 5 minute exposure (two sham and two TETRA) and two 50 minute exposure (sham and TETRA).	No effects on well-being or symptoms.
Leitgeb et al., 2008 (S)	43 participants with IEI-EMF (26 female)	Protective netting over bed to screen out EMF. Unshielded RF-EMF levels were typically 0.5% of ICNIRP reference levels	Three nights under protective netting and three nights under sham netting.	No evidence of a specific effect of shielding on subjective sleep quality

HS – Exposure analogous to a handset, BS – Exposure analogous to a base station, S – Shielding study

3.6.3.2. Observational studies

In addition to experimental provocation studies, several observational studies have recently been published which assess the possible impact of longer-term exposure to RF on symptoms, well-being and other subjective outcomes. Several of these have relied on participants to self-report their level of exposure to RF. In the largest of these studies, Korpinen and Paakkonen (2009) tested whether self-reported use of various electrical devices were associated with six psychological symptoms experienced in the past 12 months among a random sample of 6121 Finns. Only one statistically significant association was found out of the 32 analyses that were conducted using these data.

Redmayne et al. (2013) obtained data from 373 children (mean age 12.3 years (10.4 to 13.7 years)) and their parents with respect to cordless and mobile phone use, use of and type of mobile phone headset, cordless phone frequency, and presence of Wi-Fi at home.

The authors controlled for the following in their analyses: age, sex, the socioeconomic rating of the school (SES), having recently had a cold or flu, usual bedtime, exercise levels, weekend viewing/electronic gaming hours, having a television in the bedroom, the number of times woken weekly by the cell phone, and cell phone storage and carrying habits. Associations were found between headaches and having long or frequent mobile phone calls (compared to not, or hardly ever using a mobile phone) or using a wireless headset. Associations were also found between tinnitus and having a cordless phone at home with a frequency less than or equal to 900 MHz or 1.8 to 1.9GHz, and using a wired mobile phone headset. Feeling down or depressed was associated with using a wireless headset or having a cordless phone with a frequency of 900MHz or less. Waking in the night was associated with using a wireless headset or not having Wi-Fi at home. Being tired at school was associated with having a digital spread spectrum cordless phone at home. Finally, having a painful texting thumb was associated with making more or longer cordless or mobile phone calls, and sending more texts.

Hutter et al. (2010) used a case-control design to compare 100 patients attending an ear nose and throat clinic with tinnitus against 100 patients attending the same clinic but for other reasons and matched for age and sex. Both groups were asked to complete a questionnaire relating to their mobile phone usage. There was a significant association between having tinnitus and using a mobile phone on the same side of the head for four years or more prior to the onset of the tinnitus (OR 1.95, 95% CI 1.00 to 3.80). Khan (2008) compared self-reported mobile phone use and symptoms among 286 medical students. Significant associations were found between higher use of mobile phones and higher rates of eight symptoms. Similarly, Kucer et al. (2014) assessed the association between self-reported mobile phone use and symptoms in a sample of 350 people from Turkey, finding associations between higher usage and headache, joint pain and hearing loss. Szyjkowska et al. (2014) analysed questionnaire data from 587 people in Poland and identified associations between factors relating to self-reported mobile phone use and symptoms including headache, fatigue and heat sensations.

In a cross-sectional survey of a sample of 250 people living near to a base station in Iran, Shahbazi-Gahrouei et al. (2014) identified several significant associations between self-reported distance of residence from the base station and reporting of symptoms.

In a sample of 57 participants, recruited for a provocation study, Augner and Hacker (2009) looked at the association between how far participants believed they lived from a mobile phone base station, their self-reported daily mobile phone use and various measures of symptoms, anxiety and well-being. Self-reported mobile phone use was not associated with any outcome, but lower self-reported distance from a base station was associated with higher levels of symptoms and anxiety.

A survey of 251 citizens of a Bavarian town identified an association between symptoms and distance of residence from a mobile phone base station, with participants divided into four groups for the analysis based on distance (Eger & Jahn, 2010). Although exposure was assessed at an aggregate level for each of the four distance categories in this study, no attempt was made to test whether objectively measured exposure within each residence was associated with the symptoms reported by each participant.

An observational study using data from the Danish National Birth Cohort assessed the association between pre and postnatal exposure to mobile phone signals and migraine-type or other headaches in seven year old children (Sudan et al. 2012). Both types of exposure were assessed through the mother’s reports as to whether she had used a mobile phone while pregnant and whether her child currently used a mobile phone. Both migraine-type (prevalence roughly 1%) and other headaches (19%) were more common among children whose mother reported mobile phone use during pregnancy. The effect was small but statistically significant (ORs 1.2-1.3 for prenatal and postnatal exposure about 1.5 for both combined). Adjustment for other factors associated with headache diminished the effect, suggesting that residual confounding is likely to have inflated the results. Frequency of calls and amount of hands-free device use as well proportion of time the phone was on were related to other headaches (only frequency of calls showed

an association with migraine-type headache). In these analyses too, the effect was reduced after taking into account other factors.

Although several significant associations have been found in studies relying on self-reported exposure, caution is required in interpreting the associations suggested by these various studies. First, it is possible that confounders explain some of the associations. For example, a recent study by Thomee et al. (2011) assessed the association between self-reported mobile phone use and symptoms of stress, sleep disturbances and depression. While several associations were found, these related more to lifestyle factors such as the self-reported stress associated with being easy to contact than to any bioelectromagnetic mechanism. Similarly, reduced depression in older adults as a result of cell phone use has been observed but attributed to greater ability to interact socially with relatives (Minagawa and Saito, 2014), while the availability of electronic gadgets after bedtime has been associated with poor sleep and obesity (Chahal et al., 2013). With respect to the study by Redmayne et al. (2013), unmeasured differences between 12yr old children who are and are not regular users of mobile phones, such as personality or social stress, may account for some of the associations that were observed. Second, self-reports of mobile phone use or of the distance to the nearest base station are known to be inaccurate and have a poor association with actual levels of RF exposure. In particular, for the study by Sudan et al. (2012), it is difficult to quantify what level of exposure to the foetus might have occurred during maternal use of a mobile phone, except that it was presumably very low. Third, a participant's description of their previous exposure to RF may itself be influenced by their knowledge about their current health status, resulting in spurious associations being reported. Again, this seems particularly problematic for the study by Sudan et al. (2012) where maternal use during pregnancy was not assessed until seven years later. Finally, even when a participant's self-report of their exposure to RF is accurate, it is still difficult to know whether any association with symptoms is the result of RF exposure per se or whether the association is the result of a 'nocebo' effect, whereby the participant's belief that they are being exposed is sufficient to their trigger their symptoms (Rubin et al., 2010; Baliatsas et al., 2012).

Several recent studies support this last suggestion. For example, Baliatsas and co-workers (2011) sent symptom questionnaires to a random sample of 3611 participants in the Netherlands. While the perceived proximity of a mobile phone base station to the participant's home was associated with their level of symptoms, actual proximity (as determined using a comprehensive database of base station locations) showed no such associations. Similarly, although a survey of 30047 participants in Germany (Blettner et al., 2009) found a small association between the objective distance from a respondent's house and the nearest base station and their level of symptoms, subsequent RF measurements made in the homes of 1500 of the participants found no association between symptoms and objective levels of exposure (Berg-Beckhoff et al., 2009). A survey of 500 participants in Poland (Bortkiewicz et al., 2012) also observed an association between symptom reports and distance to the nearest base station, but not between symptom reports and the electric field strength recorded within the house.

In an attempt to move away from reliance on self-reported exposure and to control for possible worry as a confounder, Gómez-Perretta et al. (2013) reported findings from a re-analysis of an earlier paper (Navarro et al. 2003). Out of 215 symptom questionnaires that were distributed to residents of one Spanish town in 2001, 150 were returned and useable RF measurements were made in the bedrooms of 88 of these participants. Sixty-six of these participants were recontacted in 2012 and asked whether, in 2001, they had been worried about the presence of two local base stations and whether they believed the base stations might damage their health. Thirty-nine (59.1%) reported 'yes' to both questions. Many of the symptoms enquired about showed significant associations with exposure levels in the bedroom, which remained after controlling for covariates including age, mobile phone usage and concern about the base stations. Using two questions eleven years after the event seems unlikely to provide a good measure of attitudes and perceptions towards the presence of mobile phone base stations, however.

Given the problems of finding an adequate way to assess exposure, a welcome advance in this area has been the development of personal exposure meters which can be worn by participants during their day to day lives. The MobilEe-study has made use of these meters by asking 1484 children (aged 8 to 12yrs) and 1508 adolescents (aged 13 to 17yrs) to wear a personal exposure meter for 24 hours and to return various self-report and parent-report measures of symptoms, behaviour and mental health. The possible associations with behavioural disorders observed by this study (Thomas et al., 2010) are discussed in Section 3.5.2.1. Additional papers using the MobilEe data have assessed the associations between exposure and physical symptoms (Kuhnlein et al., 2009; Heinrich et al., 2010; Heinrich et al., 2011; Milde-Busch et al., 2010), but have not observed any consistent effects.

An alternative approach to assessing RF exposure was applied by the Swiss Qualifex team, who used a questionnaire to assess a range of 'surrogate' measures that had previously been shown in a separate study to predict RF exposure as measured using personal exposure meters. Exposure assessment covered both far fields in residential setting and use of appliances such as mobile phones, DECTs and WLAN. Information on mobile phone use was collected both from the participants and network providers. The participants were classified into three exposure groups with cut-points at the 50th and 90th percentile. The questionnaire, which also measured a range of symptom outcomes, was completed at two time-points one year apart by 1124 participants aged 30 to 60. No consistent associations were identified between exposure and non-specific symptoms, tinnitus or sleep quality (Frei et al., 2012; Mohler et al., 2010). Perceived exposure at baseline, however, (evaluated with a question about self-rated exposure compared with average population levels) was associated with symptom score and increase in self-rated exposure with headache. A more detailed analysis of sleep quality was subsequently performed for 120 of the participants who wore an actigraph on their wrist for two weeks and completed a detailed sleep diary (Mohler et al., 2012). Supplementary information on their exposure was also collected using an exposimeter in the bedroom and during a working day. Radiofrequency exposure was not associated with increases in daytime sleepiness score or sleep problems. Sleep duration and sleep efficiency showed no association with any measure of EMF exposure in the sub-study.

A systematic review of observational studies by Baliatsas and co-workers (2012) identified two to four cross-sectional studies (depending on the specific outcome) which assessed the impact of objectively assessed exposure to base station signals on subjective symptoms, which were suitable for inclusion in a meta-analysis and which were not judged to have a high risk of bias due to exposure misclassification, selective participation or confounding. In each meta-analysis "highly exposed" participants (based on the highest exposure category used by a study) were compared with the lowest exposure reference category. No significant effects of exposure were found for any acute or chronic symptoms.

Discussion on symptoms

The quality of the provocation studies which have included subjective outcomes can be evaluated against the standard criteria for randomised controlled trials, including the level of blinding, the use of randomisation and counterbalancing, the use of a sample size calculation, the reporting of participant drop-outs and the registration of a study protocol in a publically-accessible registry prior to recruitment beginning. When assessed against these criteria, the provocation studies described in this update were of reasonably good quality, with double-blinding, randomisation and counterbalancing being the norm. Room for improvement still exists with respect to some elements, however. In particular, it is rare for studies in this field to describe an a priori sample size calculation, to register a trial protocol or to report how many drop-outs occurred during the experiment.

Other quality criteria are also relevant in the particular context of IEI-EMF. One issue that is sometimes raised concerns the appropriateness of measuring subjective endpoints at all. Given that IEI-EMF is defined by the occurrence of symptoms which are attributed to electromagnetic fields, it is appropriate to test for the condition by studying the onset of

these symptoms. This does not preclude other endpoints also being studied. However, with some exceptions (e.g. Vecsei et al. 2013), the use of objective endpoints as 'surrogates' for subjective symptoms is not always valid (Fleming and DeMets, 1996).

A second general issue concerns the possibility that some people may be genuinely sensitive to radiofrequency fields, but may be hidden amongst a larger number who believe they are sensitive, but who are mistaken. In this hypothetical scenario, studies which test the effects of exposure by assessing the average response of a group of participants with IEI-EMF may be unable to detect an effect unless a large sample size is used. Studies that were published before the cut-off date for this Opinion have previously explored this possibility by testing individual participants with multiple exposures in an attempt to identify any individual who can reliably detect or react to radiofrequency fields (Rubin et al. 2005; Rubin et al. 2010). These studies have not found convincing evidence of this phenomenon, a finding supported in this update by Wallace et al. (2010). While it cannot be ruled out that some people experience symptoms as a result of exposure to RF, if this phenomenon exists at all it appears to affect only a small minority of all those who believe that they are affected.

A third general issue relates to the level of detail given regarding SAR distribution. As noted for neurophysiological studies (see Section 3.6.2.2), the details provided are often limited.

More specific issues relating to the quality of IEI-EMF provocation studies concern whether the experiments are a fair test of the sensitivity reported by their participants. To be considered a fair test, studies should ensure that: the follow-up period after any exposure is long enough to allow a participant's symptoms to develop and be recorded; the ambient levels of electromagnetic fields within the testing room are not themselves sufficient to trigger symptoms; the interval between exposures is long enough to prevent carry-over effects from occurring; and that the exposure used in the study is similar to that reported as problematic by the participants. Poor reporting by some studies included in this Opinion limited an assessment of them against these criteria. However, measures taken to ensure fairness have included: restricting participation to those people who report a short latency for their symptoms (Nieto-Hernandez et al., 2011; Leitgeb 2008); checking that ambient electromagnetic fields are not problematic for participants or using shielding or other techniques to minimise levels (Nam et al., 2009; Nieto-Hernandez et al., 2011; Kwon et al., 2012; Leitgeb et al., 2008; Wallace et al. 2010); ensuring that the interval between testing sessions was tailored to each participant (Nieto-Hernandez et al., 2011); and ensuring that only participants who reported sensitivity to the type of exposure being used in the study were recruited (Leitgeb et al., 2008; Nam et al., 2009; Nieto-Hernandez et al. 2011; Kwon et al. 2012). Wallace et al. (2010) also demonstrated that their laboratory conditions were not a barrier to symptoms developing in their IEI-EMF participants during a set of preliminary, non-blind exposures.

The most recent observational studies that have been published since the 2009 Opinion represent a substantial move forward in quality for studies assessing the relationship between long-term RF exposure and symptoms. Early studies that were suggestive of a link suffered from substantial methodological weaknesses due to their reliance on self-reported measures of exposure and their often poor control of confounding variables. Studies which have used objective measures of exposure have typically found no association between exposure and symptoms. While further work using this paradigm would be beneficial, at present these studies suggest there is no causal link between exposure and symptoms.

3.6.3.3. Conclusions on symptoms

The symptoms that are attributed by people to RF EMF exposure can sometimes cause serious impairments to a person's wellbeing. However, research conducted since the previous Opinion adds weight to the conclusion that RF EMF exposure is not the cause of these symptoms. This applies to the general public, children and adolescents, and to

people with IEI-EMF. Recent meta-analyses of observational and provocation data support this conclusion.

For symptoms triggered by short-term exposure to RF fields (measured in minutes to hours), the consistent results from multiple double-blind experiments lead to a strong overall weight of evidence that RF EMFs do not cause such effects.

For symptoms associated with longer-term exposures (days to months), the evidence from observational studies is broadly consistent but has gaps, most notably in terms of the objective monitoring of exposure. Current evidence weighs towards an absence of effects due to RF EMF exposure.

3.6.4. Other effects of RF exposure

The previous SCENIHR report concluded that there was no evidence for adverse health effects at levels below existing exposure limits on prenatal development and insufficient evidence concerning male fertility due to methodological limitations of published studies. The overall assessment found no indication of an effect of RF fields on reproduction and development

3.6.4.1. Reproductive effects

The possibility that human sperm could be particularly vulnerable to the use of mobile phones, and other sources of RF fields, has received interest and attention. The previous SCENIHR report concluded that studies on male fertility were inadequate due to low statistical power and/or methodological problems.

What has been achieved since then?

Two main approaches have been used to investigate the effects of RF fields on male fertility in humans: either phone use has been estimated in men attending infertility clinics, or samples of sperm from healthy donors have been exposed to RF fields *ex vivo*. Some studies have used a mobile phone as exposure source, but these have not been included in this assessment. In addition, one study examined reproductive outcomes in naval personnel who had been exposed to RF fields aboard a ship.

Gutschi et al. (2011) examined 2100 men attending an infertility clinic from 1993 to 2007 and reported reductions in semen quality in men using mobile phones. Samples of semen were collected from patients and analysed for sperm count and morphology, and concentrations of testosterone, FSH, LH and PRL. Patients with a history of smoking or alcohol consumption were excluded as were those with systemic disease, orchitis and varicocele. Self-reported information was also gathered on phone use, and patients were placed in either use (n = 991) or no use (n = 1119) groups: the basis for this attribution was not described. Significant differences were found between groups in sperm motility, and in abnormal sperm morphology, although no difference in sperm count was seen. Users also showed significantly higher testosterone level and lower LH levels than no users. There are a number of limitations with the study, including lack of assessment of RF exposures from other sources of RF in the home and at work, exposures to other factors that might influence fertility (cofounding) and problems associated with recall bias regarding phone use.

Reproductive outcomes were evaluated in a Norwegian study of navy personnel occupationally exposed to RF fields from radar and high-frequency antennas aboard speed boats (Baste et al. 2012). A total of more than 28,000 navy servicemen were included in the study, of whom half were land-based personnel and of those in the fleet a third had served aboard fast patrol boats. Spot measurements of electric fields were conducted in several locations aboard speed boats in 1998 and 2005. A measure of cumulative exposure was calculated based on job title, vessel type and duration of service. Average exposure level was 0.4-2.3% of the ICNIRP guideline values in 1950-1994 and 3.3-7.9% from 1995 for the rest of the crew, but roughly 90% for the captains of two of the boat types. Exposures during the three-month period preceding conception were analysed separately. Information on seven reproductive outcomes was obtained

from the comprehensive national medical birth registry. Nearly 38,000 singleton pregnancies were included in the analysis. Low birth weight was associated with work on vessels, but no such relation was found for other measures of RF field exposure. Pre-eclampsia was associated with work aboard fast patrol boats and an increased risk was found in all categories of RF exposure among men on such boats. Similar results were also found for perinatal mortality. The study used an exploratory approach with multiple comparisons involving seven outcomes and five exposure classifications which suggest that some significant results are expected just by chance. The contribution of paternal factors is likely to be small for several of the outcomes in comparison with maternal factors and events during pregnancy. Uncontrolled confounding by lifestyle factors such as paternal smoking and alcohol consumption is also a concern, and no information on maternal exposures was available. It appears that each pregnancy was regarded as an independent event, while children born to the same couple have dependence in terms of risks (this is likely to inflate the significance, but would not be expected to bias the risk estimates).

De luliis et al. (2009) exposed purified human sperm to CW 1.8 GHz fields at a range of power densities for 16 h. The SARs were determined by calorimetry to be 0.4 - 27.5 W/kg. Significant decreases in motility and vitality were reported at 1 W/kg and above, as well as significant increases in mitochondrial generation of reactive oxygen species (ROS) and DNA fragmentation at 2.8 W/kg and above. The magnitudes of these changes increased with increasing SAR. The samples were placed in 35 mm Petri dishes and exposed using a cylindrical waveguide, but the temperature in the waveguide does not appear to have been regulated using an incubator, but only controlled through the ambient temperature which was maintained at 21°C. Although the effects of increasing bulk temperature on ROS production in sperm samples were investigated, there is a strong possibility that localised hot spots would occur in the exposed samples, and numerical dosimetry is required to describe the pattern of energy absorption.

Using computer-assisted sperm analysis, Falzone et al. (2008) reported that exposure for 1 h to GSM-like pulsed signals at 900 MHz at 2 or 5.7 W/kg had no effect on progressive sperm motility. There was also no effect on sperm mitochondrial membrane potential. Samples of sperm from 12 healthy donors were exposed to RF fields using a specially-constructed irradiation chamber that was held in a humidified incubator to ensure consistency of temperature; controls were kept next to the chamber. Numerical dosimetry was used to determine the SAR distribution in the samples, which was validated using physical dosimetry. Using a similar protocol, Falzone et al. (2010a) reported that exposure of sperm to pulsed 900 MHz fields for 1 h at 2 W/kg significantly reduced the size of the head of the sperm and the acrosome percentage of the head area. Exposure also caused a significant decrease in the numbers of sperm binding to oocytes in the hemizona assay, but had no effect on the ability of the sperm to initiate the acrosome reaction. The authors suggested that the changes in sperm morphology could have been artefactual, and possibly a consequence of air-drying the semen samples (Cooper, 2012). Nevertheless, it was concluded that RF fields might affect male fertility and impair fertilization rates. Falzone et al. (2010b) examined the effects of exposure on four markers of apoptosis. Sperm samples from 12 donors were exposed to pulsed 900 MHz field at 2 or 5.7 W/kg for 1 h. and flow cytometry was used to measure caspase 3 activity, externalization of phosphatidylserine, induction of DNA strand breaks, and generation of ROS up to 24 h after exposure. No significant field-dependent effects were seen, suggesting exposure had not had any impact on pro-apoptosis events.

Rago et al. (2013) reported that, compared to men who did not use a mobile phone or used one for less than 4 h per day, DNA fragmentation was significantly increased in men who used a phone for more than 4 h a day, particularly in men who carried their phones in a trouser pocket (as opposed to a shirt pocket). Another eight indices of semen quality were not affected, nor were any changes seen in morphology of the testes. Unlike some other studies, only healthy and fertile men were assessed in this study, men with andrological disease or other conditions that might affect the outcome pf the study were excluded, and all subjects used the same mobile phone. Phone use was assessed by

questionnaire but use of cordless phones or other sources of potential exposure do not appear to have been considered.

As part of a study exploring the relationship between lifestyle factors and fertility in a population of 344 men, Jurewicz et al. (2014) reported that longer-term mobile phone use (and many other factors) adversely affected semen quality. After correction for multiple comparisons, it was found that, compared to men who had used a mobile phone for 0-5 years, the percentage of motile sperm decreased, and the percentages of atypical sperm and sperm with an abnormal head increased in men who had used a mobile phone for 11-25 years. The authors acknowledged that the study participants were drawn from those attending an infertility clinic, so may not be representative of the general population.

Adams et al. (2014) performed a systematic review and meta-analysis of 10 studies that had investigated effects of exposure to fields associated with mobile phones on sperm quality. It was concluded that overall there were negative associations between exposure and sperm viability and motility, although an effect on sperm concentration was less clear. However, all except two of the studies in the pooled analysis had used a commercial phone as an exposure source with little or no dosimetry to calculate the absorbed energy in the samples. As explained elsewhere, a mobile phone is not acceptable as an exposure source without detailed dosimetry, and studies with such methodological shortcomings should not have been included in the pooled analysis.

Liu et al. (2014) also performed a systematic review and meta-analysis on mobile phone use and semen quality using data from humans exposed *in vivo* or *ex vivo*, and *in vivo* studies using rats. While the studies were evaluated for quality, and studies failing to meet inclusion criteria were excluded, several studies using a mobile phone as an exposure source were included. Four out of six human cross-sectional studies examined in the systematic review reported negative effects, although the meta-analysis (conducted using four studies) indicated no adverse effects on sperm concentration, motility, viability or morphology. The authors listed the limitations with this type of study, which included potential publication bias for studies reporting positive results, large heterogeneity between different studies, and recall bias.

Although not concerned with male fertility per se, the effects of mobile phone use on erectile dysfunction (ED) were investigated in a pilot study by Al-Ali et al. (2014). Using questionnaires to assess phone use, it was found that the total time spent on the phone per week was not different between 20 men who had been complaining of ED for six months or more and 10 healthy men with no complaints, although those with ED carried their phones switched on for significantly longer than men without ED. The authors concluded that total time of exposure from mobile phones was more important than exposures occurring during actual use, and recommended larger studies to confirm these results. Information on where on the body the subjects carried their phones was not given.

As part of a study into factors affecting perinatal morbidity, the effects of using mobile phones by 500 pregnant women on birth weight and birth time was investigated by Col-Azaz (2013). It was found that mothers who used a mobile phone during pregnancy had significantly shorter pregnancies with a greater number of pre-term births compared to mothers who did not use a phone. In addition, talking on a mobile phone for more than an hour per day was also associated with a shorter pregnancy compared with talking on the phone for less than an hour per day. Phone usage and pregnancy duration were assessed using retrospective questionnaire and may be subject to recall or other bias; no validation of reported phone use was attempted. The mechanism for these changes remains elusive and unknown.

Discussion on reproductive effects

Studies have continued to investigate the possibility that exposure to low level RF fields from mobile phones and other sources can affect male fertility, but none of the recent studies are particularly informative. Most of the *ex vivo* studies have reported at least

one positive effect, but all these studies are subject to a variety of methodological limitations, and at least one study reporting changes in sperm morphology may be attributable to artefact. A Norwegian study examining paternal RF field exposures aboard patrol boats was large, but confounding by uncontrolled lifestyle factors cannot be excluded. Similarly, a study examining men attending an infertility clinic is also subject to possible confounding and recall bias regarding phone use. Two meta-analyses of the available data produced no clear evidence of consistent adverse effects on semen quality. Whether mobile phone use by expectant mothers during pregnancy is associated with adverse outcomes remains largely unexplored.

It is not possible to weigh the evidence on male fertility due to a lack of informative studies.

3.6.4.2. Developmental effects

What was known on this subject?

Numerous studies have shown that RF fields are teratogenic in animals at exposure levels that are sufficiently high to cause a significant elevation in core maternal temperature (>1°C); there is no consistent evidence of adverse effects at non thermal levels. The previous Opinion described two studies investigating male fertility in rats, one negative and one positive, but the dosimetry of the testes were not sufficiently characterised in either; one study also used a mobile phone as exposure source. There was a lack of proper dosimetry in two studies describing effects on development.

What has been achieved since then?

Many animal studies have investigated effects of RF fields on male fertility and on pregnancy outcome and development. Some of these studies used a commercial mobile phone, sometimes in standby mode, as the source of exposure in their experiments. Unfortunately, such studies are of no use for health risk assessment, as the exposures would have been highly complex and very variable, especially if the animals were unrestrained and free to move in their cages. In addition, the emissions from a mobile phone in standby mode would be negligible (Hansson Mild et al., 2012). These, and other studies with inadequate dosimetry, have not been included in this assessment.

<u>Male fertility</u>

Using a reverberation chamber to expose the animals, Lee et al. (2010) reported that daily exposure of SD rats to CDMA signals at a whole body SAR of 2 W/kg twice a day for 45 min, 5 days/week for 12 weeks, had no significant effect on direct and other measures of spermatogenesis. Assessments included sperm counts and histological evaluation of the testes, as well as apoptosis measured using the TUNEL assay. In addition, here was also no change in the expression of p53, bcl-2, caspase-3, key proteins related to apoptosis. In a further study, Lee et al. (2012) exposed rats to a combined CDMA and WCDMA signal at 4 W/kg for 45 min/day, 5 days/week for 12 weeks. No effects were found on testicular function, including sperm count and stage of sperm cycle, testosterone concentration in blood, or on malondialdehyde concentration and appearance of apoptotic cells in the testes. In both studies, exposure had no effect on rectal temperature.

Imai et al. (2011) investigated the effects of 1.95 GHz WCDMA fields associated with IMT-2000 phones on testicular function in Sprague-Dawley rats. Animals were exposed 5 h/day for 5 weeks at a whole body SAR of 0.08 or 0.4 W/kg: the local SARs (1 g average) in the testes were calculated to be 0.2 and 1 W/kg. There were no significant differences in the absolute or relative weights of the testes, epididymis, seminal vesicles or prostate, compared to values in sham exposed rats. There were also no changes in sperm count, mobility or in the appearance of the sperm (except for a significantly higher sperm count in the testes, but not the epididymis, of the animals exposed at 0.4 W/kg). The stage of the sperm cycle was unaffected by exposure.

No significant effects on testicular function were found by Trošić et al. (2013) following short-term, intermittent exposure of 9 male Wistar rats to 915 MHz GSM signals. Restrained animals were exposed using a GTEM cell for 1 h/day for 2 weeks at a whole-body SAR of 0.6 W/kg. The animals were examined immediately at the end of the last exposure for testis weight and morphology, and for the number, mobility or structure of epididymal free sperm.

Tas et al. (2014) investigated the effects of long-term exposure to 900 MHz fields on male fertility in rats. A group of seven animals were individually restrained and exposed using a carousel system to GSM 900 signals for 3 h/day, every day for 12 months; control animal were sham-exposed. The average SAR in the testes and prostate were calculated to be 0.04 W/kg, average whole-body SAR was similar, with a maximum whole-body SAR of 2 W/kg. No significant effects were seen on epididymal sperm concentration or motility, or on testes weight or general morphology, although the percentage of normal sperm was lower, and the thickness of the tunica albuginea was reduced in the exposed animals. In addition, spermatogenesis (assayed using the Johnsen biopsy score) was significantly reduced by exposure. The authors suggested that chronic exposure may accelerate degenerative changes in the testis, but this is based on data from relatively modest numbers of animals.

Chaturvedi et al. (2011) reported that whole-body exposure of mice to CW 2.45 GHz fields at 0.04 W/kg for 2 h/day for 30 days had no significant effect on epididymal sperm count or motility. Treatment groups were very modest, however, consisting of 5 animals, which limit the usefulness of this study. A later study by the same group reported that sperm count and viability were decreased in mice that had been exposed to CW 2.45 GHz at an averaged, whole-body SAR of 0.018 W/kg for 2 h/day for 30 days (Shalin et al., 2014). In addition, exposure produced significant changes in testicular morphology, decreased plasma testosterone levels, and increased levels of reactive oxygen species (ROS) and reactive nitrogen species (RNS) in the testis, liver and other tissues; exposure also resulted in a reduction in the activity of antioxidant enzymes. Although 20 animals were exposed in total, most parameters were measured in 15 animals, although some endpoints, including testes morphology, were examined in only five animals. Overall, it was suggested that infertility may result from chronic, low level irradiation via a ROS/RNS-mediated pathway. The local SAR in the testes was not calculated.

In a series of studies, Behari and co-workers have examined the effects of long-term, low level exposure to various RF fields on fertility and testicular function in Wistar rats. In these studies, the observed changes are attributed to a field-induced increase in reactive oxygen species. However, the size of the treatment groups is mostly very small (n = 9 or less); comparable results are found irrespective of applied frequency; and the whole body SARs have been provided using simple models with no attempt made to calculate the local SAR in the testes using computational dosimetric models. Kesari and Behari (2010) reported changes in the activities of antioxidant enzymes in epididymal sperm as well as effects on apoptosis and the spermatogenesis cycle using 10 GHz fields. The activities of glutathione peroxidase, superoxide dismutase and histone kinase both decreased, while the activity of catalase increased; apoptosis significantly increased and the percentages of sperm in S and G_2/M phase, assessed by flow cytometry, significantly decreased. In this study, freely-moving animals were exposed to CW fields for 2 h/day for 45 days, at a whole-body SAR of 0.8 mW/kg. Similar results were also reported by these authors using CW 10 GHz fields at 0.014 W/kg (Kumar et al. 2011a, 2012, 2013). Kumar et al. (2011b) reported that exposure to 50 Hz-modulated 2.45 GHz fields at 0.014 W/kg for 2 h/day for 60 days resulted in significant increases in caspase-3 and creatine kinase activity in sperm. Serum concentrations of testosterone and melatonin were also significantly decreased in the exposed animals. Ghanbari et al. (2013) exposed rats for 8 h/day for 2 weeks to signals representing either GSM phone handsets (pulsed 915 MHz) or base stations (pulsed 950 MHz). Both signals had no effect on either sperm count or testes morphology but both caused a significant decrease in sperm viability and motility. In addition, exposure to handset signals for 3 weeks resulted in further significant decreases in sperm viability and motility. Total antioxidant capacity in sperm

was also reduced by exposure, but longer exposures did not increase the change significantly. Caveats exist with this study: only the average field strength was given (1.6 mW/cm^2), and there was no indication of the actual SAR in the animals. Further, six freely-moving animals appear to have been exposed as a group at any time, so the absorbed dose to any individual or their testes would be uncontrolled and variable.

Qin et al. (2014) reported changes on numerous measures of male fertility in rats after repeated, low level exposure to 1800 MHz fields. Beginning at various times throughout the light-dark cycle, groups of restrained animals were exposed using a GTEM chamber to a CW field at a whole-body SAR of 0.04 W/kg for 2 h/day for 32 days. Exposure was associated with significant reductions in testosterone, daily sperm production and sperm motility, with concomitant changes in the activities of two testes marker enzymes and in the expression of two genes encoding enzymes involved in sperm production and maturation. However, the largest effects were seen when exposures coincided with peak time of plasma testosterone concentration (as observed in sham-exposed animals) which was towards the end of the dark phase and beginning of the light phase. The circadian rhythms for most parameters were either altered in exposed animals, or in the case of testosterone, abolished.

The effects of long-term exposure to Wi-Fi signals on male fertility in rats were investigated by Dasdag et al. (2014). In this study, a group of eight adult males were housed together in a cage held 50 cm from the antenna of a generator producing 2.4 GHz fields at 50 mW, and exposed continuously for 24 h/day, 7 days/week for 12 months. Compared to another group of eight similarly-housed animals not exposed to the field, exposure significantly increased defects of the head of the sperm and the weights of the epididymis and seminal vesicles, diameter of the seminiferous tubules, and thickness of the tunica albuginea were all decrease. There were no significant effects on sperm motility or concentration, or on the weight of the testes. The SAR used is quoted at 2.42 mW/kg (1g average) but the text presents the results of theoretical calculations suggesting the local SAR in the testes was in the region of around 0.5-1 mW/kg (10 g average). However, absorbed power would depend on the behaviour of the animals and be highly variable, making it impossible to replicate the exact exposure conditions used in this study.

<u>Pregnancy outcome and development</u>

Ogawa et al. (2009) examined the effects of head-only exposure of Sprague-Dawley rats to a 1.9 GHz W-CDMA signal during pregnancy. Mothers were exposed using a head-mainly system for 90 min each day at 0.67 or 2 W/kg on gestational day 7 to 17. Mothers and foetuses were examined on gestational day 20 for implantation and foetal losses, internal abnormalities and external malformations. No significant changes were seen in either the mothers or foetuses.

Bas et al. (2009b) reported that exposure of Wistar rats to CW 900 MHz fields for 90 min/day from conception until birth resulted in significant losses in pyramidal cell numbers in area CA1 at 4 weeks of age as measured using optical fractionator techniques. Mothers were exposed using a head-only system. The low numbers of animals used (results were obtained from 3 litters per treatment) means no conclusions can be drawn.

Lee et al. (2009) reported no significant effects on mouse foetuses following daily, combined exposure to 849 MHz CDMA and 1.95 GHz W-CDMA signals throughout pregnancy, at a whole-body SAR of 4 W/kg or to CDMA signals at 2 W/kg. In a follow-up study, Jin et al. (2011) reported that exposures of young rats to these signals for a year had no adverse impact on health: no significant changes were seen except for some altered parameters of the complete blood count and serum chemistry.

Takahashi et al. (2010) reported a lack of teratological effects following whole-body exposure of pregnant rats to a 2.14 GHz W-CDMA base station signals. Freely-moving animals were exposed for 20 h per day from day 7 of gestation to weaning; SARs used were 0.028-0.040 and 0.066-0.093 W/kg in mothers which corresponded to 0.029 or

0.068 W/kg in the foetus, and 0.061-0.067 and 0.143-0.156 W/kg in offspring. Offspring were scored for visceral and skeletal abnormalities, external malformations, growth, and physical and reflex development. From 5 weeks of age, offspring were also assessed for functional development by measuring behaviour in an open field arena and spatial learning in a water maze. In addition, the fertility and reproductive ability of the offspring at 10 weeks was assessed. A few significant effects were reported but these were discounted as being transient or inconsistent. However, in the probe trial in a water maze task, the exposed males spent a small but significant increase of time in the target quadrant compared with the sham-exposed animals, suggesting a modest improvement in learning had occurred.

Sambucci et al. (2010) examined the early and late effects of acute, daily exposure to a WiFi signal during pregnancy with particular emphasis on the immune system. Pregnant C59BL/6 mice were exposed to a pulsed 2.45 GHz signal at 4 W/kg from day 5 of gestation for 2 h each day. Animals were restrained during exposure. No effects on pregnancy outcome were seen, and there were no consistent effects on immune parameters including B-cell compartment and antibody production in offspring at 5 or 26 weeks of age. Sporadic differences were noted, but these were attributed to the effects of confinement stress during exposure, or to sex- or age-related changes. In a follow-up study examining the effects of exposure on the T-cell compartment, no consistent field-related effects were seen at either time point on T cell counts, phenotype, or on thymocyte proliferation, and no effects were seen on peripheral (spleen) T cells (Laudisi et al., 2012). A companion study examined the effects of early postnatal exposure to WiFi signals on the maturation of the immune system in mice (Sambucci et al. 2011) and no consistent field-dependent effects were found. Newborn animals were exposed for 2 h/day, 5 days/week for 5 weeks at a whole body SAR of 0.08 or 4 W/kg.

Aït-Aïssa et al. (2012) reported that prenatal and postnatal exposure of free-running rats had no significant effects on immunological markers or on growth. Animals were exposed using a reverberatory chamber to a 2.45 GHz Wi-Fi signal for 2 h/day, 5 days/week from day 6 to 21 of gestation, and then from birth to postnatal day 35 at a whole-body SAR of up to 4 W/kg for pregnant animals, and up to 9 W/kg for offspring. Blood samples were collected on postnatal day 35 and analysed by ELISA for antibodies related to 15 antigens related damage or pathology.

Poulletier de Gannes et al. (2012) have also investigated the effects of prenatal exposure to 2.45 GHz WiFi signals on the development of rats. Pregnant animals were exposed using a reverberation chamber for 2h/day. 6 days/week for 18 days at a whole body SAR of 0.08, 0.4 and 4 W/kg. There were no significant effects on pregnancy outcomes, or on the weight and postnatal development of the offspring. Exposure was also without significant effect on the health or behaviour of the pregnant animals. A second study investigated the effects of WiFi signals in rats exposed before and during pregnancy (Poulletier de Gannes et al., 2013). Six week-old male rats were exposed for 1 h/day, 6 days/week for 3 weeks, and seven week-old female rats were similarly exposed for 2 weeks; mating pairs of animals were then exposed for a further 3 weeks. Freely-moving animals were exposed using reverberation chambers at a whole-body SAR of 0.08 or 4 W/kg; control animals were sham-exposed. At the end of exposure, males were examined for testes histopathology, and pregnant females were examined the day before delivery for pre- and post-implantations losses, numbers of foetuses and external abnormalities. No field-dependent effects were seen at either SAR, In contrast, results of a study by Nazıroğlu and co-workers (Özorak et al., 2103; Çetin et al., 2014) suggest prenatal and early postnatal exposure of male rats may increase oxidative stress in testis and other tissues. Restrained rats were exposed to either pulsed 900, 1800 or 2.45 GHz fields using a carousel-type system for 60 min/day, 5 days/week during gestation and until six weeks old. The average whole body SAR was calculated to be 0.18-0.15 W/kg, with a reported range of 0.001 to 1.2 W/kg (presumably the lower values represent absorption during early embryofoetal development). Effects were found that appeared to depend on the length of exposure (or age of animals) but not on frequency of the applied field. Özorak et al. (2103) found that lipid peroxidation levels in the kidney and testis

decreased at 4 weeks of age, were not changed at 5 weeks, and decreased in the kidney and increased in the testis at 6 weeks; total antioxidant status decreased throughout, and iron (Fe) concentration in kidney tissues increased. Glutathione peroxidase levels decreased in the kidney and increased in the testis at 4 weeks of age and were unaffected thereafter. Exploring effects of 900 and 1800 MHz only, Çetin et al. (2014) found lipid peroxidation levels in the liver increased at all times, but were unaffected in the brain. Glutathione peroxidase levels decreased in both the brain and liver at 4 and 5 weeks and were unaffected at 6 weeks; brain iron levels were increased throughout exposure. Overall, these effects are suggestive of the possibility of some increased risk to normal development from oxidative stress, but they are far from conclusive.

Using a similar protocol to the above study, Dundar and co-workers examined the effects of prenatal and early postnatal exposure to a 2.45 GHz field pulsed at 217 Hz on growth and development in female rats (Sangun et al., 2014). Restrained animals were exposed using a carousel-type system in an EMF-shielded room for 60 min/day at 0.1 W/kg: one group of 4 pregnant females was exposed during gestation and then female offspring were exposed from postnatal day 21 days until puberty (prenatal group); another group of female offspring from 4 pregnant animals was exposed from day 21 until puberty (postnatal group); other animals were sham-exposed. Exposure was found to cause some significant effects that appeared to depend on the exposure period. Neither exposure period had an effect on birth weight of offspring, but the rate of weight gain by the prenatal group was lower than that of the other groups, although daily food and water consumption of both exposed groups were greater than those of the control group. Onset of puberty was also significantly delayed in the prenatal group (by about 14 days compared to controls). Total oxidant status in the brain and ovary were significantly increased in the prenatal group, and the oxidative stress index was increased in both exposed groups. IGF-1 levels were also significantly lower in the postnatal group However, exposure had no significant effect on the histology of the hypothalamus or on ovarian follicular count. Overall, it was concluded that the field had induced chronic stress in the exposed animals, particularly during the prenatal period, resulting in restriction of postnatal growth and delayed onset of puberty.

The effects of early postnatal exposure to GSM 1800 signals in the developing brain were investigated by Watilliaux et al. (2011). Young Wistar rats were exposed for a single 2 h period on postnatal day 2, 15 or 35 at whole body SAR of 0.13-1.2 W/kg, corresponding to a local SAR in the brain of 1.7-2.5 W/kg. No evidence of early neural cell damage in any brain region was seen 24 h after exposure, as measured by expression of HSP60 or HSP90 or for markers for glial development or activation. There was also no significant effect on the proteins involved in astroglial modulation of glutamate neurotransmission.

Ozlem Nisbet et al. (2012) reported that early exposure to RF fields increased the maturity of male rats. Young animals (2 days old) were exposed to 900 or 1800 MHz for 2 h/day for 90 days. The whole body SAR varied with age, and was between 3 and 1.2 mW/kg with 900 MHz, and 0.05 and 0.01 mW/kg with 1800 MHz. Exposure at both frequencies was associated with higher levels of testosterone, and an increased motility of epididymal sperm which had fewer abnormalities.

The haematological effects on long-term, low level exposure of rats to RF fields were investigated by Adang et al. (2009). Groups of free-moving animals were exposed every day for 2 h for up to 21months to either CW or pulsed (at 1 Hz) 970 MHz fields or to 9.70 GHz CW fields. The SAR in the animals was not calculated, but the power density was measured at 2.06 W/m at 970 MHz and 3.2 W/m at 9.70 GHz. Blood samples were regularly taken during exposure. Small but significant increases were observed in all groups of exposed animals for most variables examined, especially after the first 3 months of exposure, with pulsed fields generally causing a reduced response compared with CW fields. Survival was also significantly decreased in animals exposed to 9.70 MHz CW fields. This result is intriguing, as it is not consistent with the results of earlier long-term studies. The absence of dosimetry here is of concern, but exposure did not induce

increases in rectal temperature nor induce behavioural signs of thermal stress in the exposed animals.

Sommer et al. (2009) examined the effects of lifetime exposure to 1.966 GHz UMTS signals over four generations of mice. Freely-moving animals were exposed for 23.5 h/day, in groups of 2 or 3 adults, 2 adults and 6 pups or 4 young mice, at 1.35, 6.8 or 22 W/m^2 (corresponding to whole body SARs for adult mice of 0.08, 0.4 or 1.3 W/kg[1]). No significant changes were seen on testicular function or female fertility, rates of malformations and abnormalities or on early development of offspring. Exposure was associated with a trend towards lower food consumption in exposed males, possibly due to a decrease in metabolism caused by the absorption of RF energy. This effect was independent of exposure level and occurred in all four generations of mice.

Discussion on developmental effects

Animal studies allow the effects of long-term exposure to RF fields on testicular function and development to be examined in detail. Unlike the situation with humans, it is possible for animals to be exposed to controlled and well-characterised fields without possible confounding from other RF sources in the environment. The timescale of *in utero* and post-natal development in rodents is also amenable to investigation in laboratory studies. Recent well-conducted studies indicate that long-term, repeated exposures to WCDMA and/or CDMA signals at whole body SARs of up to 4 W/kg are not associated with adverse effects on testicular function in rats. Such results are consistent with a number of other studies reporting a lack of effects in the absence of significant testicular heating. In contrast, one laboratory has reported that long-term, low-level exposure at 2.45 or 10 GHz may cause adverse effects in sperm through a field-induced increase in reactive oxygen species. However, these and similar studies are of modest size, and confirmatory studies with larger numbers of animals would be useful. For acute exposures, it is possible that the time of day when exposure occurs could be a significant influence on outcome. Most recent studies investigating effects on pregnancy outcome and development of the offspring have been large and well conducted, and so can provide very useful information. These studies found that low level prenatal and early postnatal exposure to a variety of RF signals was not associated with any adverse outcome, although one study suggested early postnatal exposure increased maturity in male rats, and another suggested long-term, low level exposure of rats may reduce survival. In addition, no significant effects were seen following almost continuous, lifetime exposure of mice over four generations.

Conclusions on reproduction and developmental effects

The previous SCENIHR Opinion concluded that there were no adverse effects on reproduction and development from RF fields at non-thermal exposure levels. The inclusion of more recent human and animal data does not change that assessment. Therefore, it is concluded that there is strong overall weight of evidence against an effect of low level RF fields on reproduction or development.

3.6.5. Conclusions on the health effects of exposure to RF fields

Neoplastic diseases

Overall, the epidemiological studies on RF EMF exposure do not show an increased risk of brain tumours. Furthermore, they do not indicate an increased risk for other cancers of the head and neck region. Some studies raised questions regarding an increased risk of glioma and acoustic neuroma in heavy users of mobile phones. The results of cohort and incidence time trend studies do not support an increased risk for glioma while the possibility of an association with acoustic neuroma remains open. Epidemiological studies do not indicate increased risk for other malignant diseases including childhood cancer.

A considerable number of well-performed *in vivo* studies using a wide variety of animal models have been mostly negative in outcome.

A large number of *in vitro* studies pertaining to genotoxic as well as non-genotoxic end-points have been published since the last Opinion. In most of the studies, no effects of exposure at non-thermal levels were reported, although in some cases DNA strand breaks and mitotic spindle disturbances were observed.

Nervous system

The earlier described evidence that RF exposure may affect brain activities as reflected by EEG studies during wake and sleep is further substantiated by the more recent studies. With regard to these findings, studies which aim at investigating the role of pulse modulation and which use more experimental signals indicate that effects on the sleep EEG are neither restricted to NREM sleep (one study indicates effects also in REM sleep) nor to the spindle frequency range. It seems that depending on the EMF signal the theta and delta frequency range in NREM sleep can also be affected. Furthermore, half of the experimental studies looking at the macrostructure of sleep (especially those with a longer duration of exposure) also found effects, which, however, are not consistent with regard to the affected sleep parameters. Therefore, given the variety of applied fields, duration of exposure, number of considered leads, and statistical methods it is presently not possible to derive more firm conclusions.

Symptoms

Symptoms that are attributed by some people to RF EMF exposure can sometimes cause serious impairments to a person's quality of life. However, research conducted since the previous SCENIHR Opinion adds weight to the conclusion that RF EMF exposure is not causally linked to these symptoms. This applies to the general public, children and adolescents, and to people with idiopathic environmental intolerance attributed to electromagnetic fields (IEI-EMF). Recent meta-analyses of observational and provocation data support this conclusion.

For symptoms triggered by short-term exposure to RF fields (measured in minutes to hours), the consistent results from multiple double-blind experiments give a strong overall weight of evidence that such effects are not caused by RF exposure.

For symptoms associated with longer-term exposures (measured in days to months), the evidence from observational studies is broadly consistent and weighs against a causal effect. However, it has gaps, most notably in terms of the objective monitoring of exposure.

Other effects

Human studies on neurological diseases and symptoms show no clear effect, but the evidence is limited.

Human studies on child development and behavioural problems suffer from conflicting results and methodological limitations. Therefore, the evidence of an effect is weak. Effects of exposure on foetuses from mother's mobile phone use during pregnancy are not plausible owing to extremely low foetal exposure.

Studies on male fertility are of poor quality and provide little evidence.

3.7. Health effects from IF fields

3.7.1. What was already known on this subject

The previous Opinion outlined that "very little research on IF in occupational settings or for the general public has been presented since the previous Opinion, and no epidemiological studies have appeared. Consequently, the data are still too limited for an appropriate risk assessment". It was also recommended that research into health effects from IF fields should be given a priority.

3.7.2. What has been achieved since then

Despite the wide range of sources of IF MFs, there are still very few studies that address possible health effects of IF exposures. A case in point is that no epidemiological studies have been published since the last SCENIHR Opinion. The few relevant studies that have been identified include both *in vivo* and *in vitro* approaches.

Weinberg et al. (2012) provided experimental data on PNS thresholds to oscillating magnetic field stimulation from 2 to 183 kHz in 26 adults human. It was found that for 0.4 T stimulations, the probability of sensation was markedly reduced at frequencies of 101 and 183 kHz when compared to the probability of sensation at 2 and 25 kHz. This is of interest in designs of human and preclinical MRI systems.

In line with studies performed mainly in the 1990´s, possible teratological effects of 20 kHz, triangular shaped, MF were investigated on ICR mice foetuses (Lee et al. 2009). This signal is emitted by video display terminals and inconsistent effects of exposure on embryo development in several species have been documented (see Juutilainen 2005 for a review). The work by Lee et al. employed a 20 kHz vertical MF, 30 µT peak-to-peak, which was applied for 8 h per day from gestational day 2.5 to 15.5 as whole-body exposure. This flux density was chosen since it is the occupational exposure limit for 20 kHz MF in Korea. Exposed and sham-exposed animals were placed during treatment in separate rooms. A background 60 Hz MF was reported only for the exposure situation (ca 0.11 µT). Animals were sacrificed on gestation day 18, whereafter dams and foetuses were investigated for a number of end-points. No exposure-related effects were noted in the dams, including clinical signs, body weight and body weight gain. The foetuses were investigated for viability, malformations, weight and length, and gender. In addition, the investigation included observations of implantation end-points. In no single case, was any effect of exposure noted. This study extends the work by the same group where the effects of the 20 kHz signal at 6.25 µT peak intensity were investigated. That study also came out as negative, i.e. revealing no exposure-related effects (Kim et al. 2004). The relevance of these low MF exposure levels in studies on mice for the human situation is difficult to evaluate.

A series of studies of IF exposure effects on embryonic development has been published by Nishimura and co-workers (Nishimura et al., 2009; 2011; 2012). Their work has its rationale in the increasing domestic use of induction ovens or cookers in Japan. Consequently, 20 and 60 kHz sinusoidal MF effects were investigated in these studies.

In Nishimura et al. (2009) White Leghorn chick embryos were exposed during the first 2, 7, or 11 days of embryogenesis. A 20 kHz vertical sinusoidal B-field (0.011, 0.11, or 1.10 mT rms) was generated by Merritt-like coils in true exposure-sham experiments (blinded exposure and analysis conditions). The eggs were placed horizontally, and the calculated maximal E-field within the eggs was 1.8 V/m for the 1.1 mT exposure, which however does not reflect the true exposure of the embryo itself. No significant effects on any investigated parameter was seen after the experiments (performed in triplicate), at any of the investigated flux densities. In addition, embryos treated with retinoic acid (a known teratogenic agent) responded as expected with embryonic death and developmental abnormalities in 40-60% of sham exposed embryos, which was similar to the outcome in the MF treated specimens. The same group employed Crl:CD(SD) rats in two subsequent studies, where effects of 20 kHz or 60 kHz MF (sine wave) on embryonic organogenesis (Nishimura et al. 2011) and fertility and early embryogenesis (Nishimura et al. 2012). In the first of these studies, pregnant rats were exposed to either a 20 kHz (0.2 mT rms) or 60 kHz (0.1 mT rms) vertical MF for 22 h/day (gestation day 7 to 17). The dams were sacrificed on day 20, whereafter maternal toxicity, reproductive performance and prenatal mortality, litter viability, weight, and abnormalities were investigated. The experiments were performed twice for both types of MF. The occasional end-point differed between exposed and sham in single experiments, but this was not repeated. This includes a skeletal variation which was significantly increased in one of the two 20 kHz experiments, and an increased foetus sex ratio (more females than males) in

the second of the two 60 kHz experiments. No other end-points differed between foetuses from exposed or sham conditions.

The most recent of the studies from this group (Nishimura et al. 2012) employed the same MF exposure, but with the important difference that exposure was confined to both male and female animals 14 days prior to and during mating. Pregnant females were furthermore exposed until gestation day 7 and subsequently sacrificed. A large number of parameters regarding fertility, maternal and paternal toxicity, and early embryonic development were investigated. The only significant differences between exposed and sham were seen in one of two 60 kHz experiments, where the body weight in pregnant mice was lower in exposed animals. One group exposed to 20 kHz had lower body weight than their unexposed counterparts. However, no effects on reproductive outcome were documented in this study.

Three *in vitro* studies emanating from concern for negative health effects from exposure to IF of the type coming from induction hobs were published in the investigated period. The first study exposed cultured hamster CHO-K1 cells to a 23 kHz MF (6.05 mT rms; 2 h) and investigated genotoxicity (cell growth; comet assay – bot neutral and alkaline; micronucleus formation; HPRT gene mutation) (Sakurai et al. 2009). Cells were seeded, cultured for 16 h, and exposed to MF, sham or an appropriate positive control for 2 h, followed by further culture for up to 5 days. The MF exposure did not cause any different effects than sham exposure, whereas the positive controls gave expected results. Stress responses (expression levels of hsp27, hsp70, hsp105, phosphorylation of hsp27 and its nuclear translocation) were investigated in A172 human glioblastoma cells. Here, heat treatment (42.5 or 43 ^{o}C) served as positive control. No MF exposure-related effects were seen.

In subsequent studies, the same group investigated a more subtle end-point, i.e. global gene expression. A human astroglia cell line was used and exposed to the 23 kHz field, but at 100 μT rms (2, 4, or 6 h) after which cell cycle analysis and microarray analysis of gene expression was performed. Results were compared to the positive control (heat 43 ^{o}C, 2 h). No effects from exposure on either cell cycle distribution or gene expression were seen (Sakurai et al. 2012). Moreover, an absence of alteration in gene expression profile of human-foetus- derived astroglial cells (SVGp12) exposed to a magnetic field at 23 kHz for 2, 4 and 6 h (2 mT rms) was also reported (Sakurai et al., 2013).

In view of the expected increase of occupational exposure to IF, studies on biomarkers and health outcomes in workers, which are based on reasonably sized groups with well-characterized exposure, would be informative. This could be supplemented with experimental studies.

3.7.3. Conclusions on health effects from IF fields

There are few new studies on health effects from IF exposures in general, and no epidemiological studies have been conducted in particular. Some *in vivo* studies report on the absence of effects on reproduction and development of IF fields up to 0.2 mT in a frequency range of 20-60 kHz.

As in the previous SCENIHR Opinion, there are still too few studies available, and furthermore no epidemiological studies have been conducted. In view of the expected increase of occupational exposure to IF, studies on biomarkers and health outcomes in workers are recommended. This could be supplemented with experimental studies.

3.8. Health effects from ELF fields

3.8.1. Neoplastic diseases

3.8.1.1. Epidemiological studies

What was already known on this subject?

The previous SCENIHR statement endorsed the IARC assessment of classifying ELF magnetic fields as possibly carcinogenic to humans due to consistently observed increased childhood leukaemia risk in epidemiological studies (SCENIHR, 2009); the latter stems mainly from two pooled analyses based on studies completed before the year 2000, showing a two-fold risk increase with ELF magnetic fields above 0.3-0.4 µT (time-weighted average) but raising concerns about shortcomings of those studies preventing a causal interpretation (Ahlbom et al., 2000; Greenland et al., 2000).

What has been achieved since then?

Childhood cancers

Several studies on childhood cancers were completed later and not included in the pooled analyses by Ahlbom et al. (2000) or Greenland et al. (2000), some of them reviewed in the SCENIHR 2009 statement, but another pooled analysis of the more recent studies became just available in 2010 (Kheifets et al., 2010a). Of the included studies in the new pooled analysis, four were conducted in Europe (Germany, UK, 2 from Italy), and one each was conducted in Japan, Brazil and Australia. There were a total of 10,865 cases and 12,853 controls; however, total numbers in the high-exposure categories were small, even for this large data set. In the pooled analysis, combined ORs increased with increasing exposure, with non-significant ORs for exposure categories of 0.1–0.2 µT, 0.2–0.3 µT and 0.3+ µT, compared with ≤0.1 µT, being 1.07 (CI 0.81–1.41), 1.16 (CI 0.69–1.93) and 1.44 (CI 0.88–2.36), respectively. For 0.4+ µT compared <0.1 µT, the combined OR was 1.46 (CI 0.80 –2.68). The combined OR increased when Brazil was omitted and was 2.02 (CI 0.87–4.69) for 0.4+ µT, very similar to the doubling in risk in the pooled analysis of earlier studies (Ahlbom et al., 2000). No other individual study made such an impact on the overall result; the concern about the Brazilian study was their choice of controls. Individual studies used in the pooled analysis but not in the last SCENIHR statement were the ones from the UK (Kroll et al., 2010), Brazil (Wünsch-Filho et al., 2011), and one of the two from Italy (Malagoli et al., 2010). A study in the US (California) was published after the conduct of the new pooled analysis, with indoor and outdoor contact voltage and ELF magnetic field measurements collected for 245 cases and 269 controls (Does et al., 2011). For magnetic fields, no association with childhood leukaemia risk was seen (>0.20 µT: OR= 0.76, CI: 0.30-1.93). In addition, no statistically significant associations were seen between childhood leukaemia and elevated indoor contact voltage levels (OR= 0.83, CI: 0.45-1.54) or elevated outdoor contact voltage levels (OR= 0.89, CI: 0.48-1.63), providing little evidence that contact currents represent a plausible mechanism to explain the association between ELF exposure and childhood leukaemia risk. There was also some renewed interest in investigating childhood leukaemia risk in relation to distance to the closest high-voltage power line, which may be interpreted as a proxy of exposure to magnetic fields, although with several limitations (Maslanyj et al., 2009). In a study in France involving 2779 cases and 30,000 population controls, the OR living within 50 m of a 225-400 kV power line was 1.7 (CI: 0.9-3.6) (Sermage-Faure et al., 2013); there was no association beyond this distance. Pedersen et al. (2014a), using 1698 cases and 3396 controls in a case-control study in Denmark, found no association living within 200 m of a nearby high-voltage (132-400 kV) power line (OR 0.76; CI: 0.40-1.45). The same group also reported that while there was no interaction between distance to power line and exposure to air pollution, there was one with radon exposure, albeit based on very small numbers and possibly a chance finding (Pedersen et al., 2014b). In the UK, based on 16,630 cases diagnosed between 1962-2008 and 20,429 controls, no association was seen within the 200 m corridor to the nearest high-voltage (275-400 kV) power line, with an OR of 1.00 (CI: 0.75-1.34) (Bunch et al., 2014). There were also no associations with any other cancers.

Kheifets et al. (2010b) carried out a pooled analysis of studies on ELF magnetic fields and risk of childhood brain tumours following the analytical approach of the pooled analyses for childhood leukaemia described above, including 10 individual epidemiological studies. The ORs for childhood brain tumours compared to a reference category of up to 0.1 µT

were 0.95 (CI: 0.65-1.41), 0.70 (CI: 0.40-1.22), and 1.14 (CI: 0.61-2.13), for exposures of 0.1-0.2 µT, 0.2-0.4 µT, and 0.4+ µT. A Japanese study (Saito et al., 2010) reported a very high OR with wide CI for exposures of 0.4+ µT (10.9, CI: 1.05-113) based on 3 cases, but was included in the pooled analysis finding no effect.

A population-based case-control study in Germany investigated if children whose parents were exposed preconceptionally at work to ELF magnetic fields had an increased risk of developing cancer (Hug et al., 2009). The analysis included 2,382 controls and 2,049 cases (among them 846 children with acute leukaemia and 444 children with central nervous system tumours). No increased cancer risks in children whose fathers were occupationally exposed to ELF magnetic fields above 0.2 µT, or even above 1 µT were observed. In a meta-analysis provided in this paper combining all previous studies on this topic for leukaemia, a pooled risk estimate of 1.35 (CI: 0.95-1.91) was observed; given the high degree of heterogeneity in the reported results and the suggestion of publication bias as identified by this meta-analysis. Other uncertainties that may influence the differences between studies include selection bias and information bias. According to the authors, their quantitative summarization has, therefore, to be interpreted with caution.

In an Australian case-control study on childhood acute lymphocytic leukaemia published later than this meta-analysis, 379 case and 854 control mothers and 328 case and 748 control fathers completed an occupational history questionnaire (Reid et al., 2011). There was no association between maternal (OR=0.96; CI: 0.74-1.25) or paternal (OR=0.78; CI: 0.56-1.09) exposure to ELF any time before the birth and risk of leukaemia. In a UK register-based case-control study including 16,764 cases, OR were 1.1 (CI: 0.98-1.23) for lymphoid leukaemia, 0.82 (CI: 0.64–1.06) for acute myeloid leukaemia, and 1.64 (CI: 1.14–2.38) for other leukaemias; exposure was based on an assessment of occupational groups by an occupational hygienist (Keegan et al. 2012). Maternal ELF exposure and risk of childhood brain tumours was addressed in a Canadian case-control study (Li et al., 2009). A total of 548 incident cases and 760 healthy controls were included in this study and quantitative occupational ELF exposure in µT units was estimated using individual exposure estimations or a job exposure matrix. Using the average exposure metric measured before conception, an increased risk was observed for astroglial tumours (OR=1.5, CI: 1.0-2.4). During the entire pregnancy period, a significantly increased risk was observed for astroglial tumours as well as for all childhood brain tumours and significantly increased risks were specifically observed among sewing machine operators.

Under the hypothesis that ELF magnetic fields may promote growth of leukaemia cells, investigators have studied the relationship with length of remission and overall survival after childhood acute lymphoblastic leukaemia (ALL). Previous studies in the US and Germany reported poorer survival in children with ALL exposed to ELF magnetic fields above 0.2/0.3+ µT, but the number of exposed children was small (SCENIHR, 2009). A pooling study reported results obtained from over 3000 children with ALL with ELF magnetic field exposure data from Canada, Denmark, Germany, Japan, the UK, and the US, who were followed for up for 10 years for relapse, second neoplasm, and survival (Schüz et al., 2012). The hazard ratios by 0.1 µT increases were 1.00 (CI: 0.93-1.07) for event-free survival analysis and 1.04 (CI: 0.97-1.11) for overall survival. ALL cases exposed to 0.3+ µT did not have an increased risk of relapse or of dying, with hazard ratios of 0.76 (CI: 0.44–1.33) for event-free survival and of 0.96 (CI: 0.49–1.89) for overall survival (Schüz et al. 2012).

There is little new data available on the association between quantitatively assessed ELF magnetic fields and the risk of childhood leukaemia; meta-analysis of studies published 2000-2009, however, confirms an approximately two-fold increased risk at average magnetic field levels above 0.3/0.4 µT. Concerns remain that the association may be inflated or even entirely explained by methodological shortcomings of the epidemiological studies. A large study on ELF magnetic field exposure and survival after childhood leukaemia did not provide support for an effect on the leukaemia prognosis. No

association has been observed for the risk of childhood brain tumours. The possible association between preconceptional parental occupational exposure to ELF magnetic fields and risk of cancer in their offspring has also been studied, but most studies provide no support for an effect of ELF magnetic fields. In conclusion, the new epidemiological data do not alter the assessment that ELF magnetic field exposure is a possible carcinogen based on the reported association with childhood leukaemia risk.

Adult cancers

Elliott et al. (2013) conducted a register-based case-control study on adult cancers in relation to distance from high-voltage power lines in England and Wales. They compared 7823 leukaemia cases, 6781 brain and central nervous system tumour cases, 9153 malignant melanoma cases, and 29,202 female breast cancer cases with a control group consisting of other cancers (n=79,507). For distances closest to the power lines, ORs ranged from 0.82 (CI: 0.61-1.11) for melanoma to 1.22 (CI: 0.88-1.69) for brain and central nervous system tumours, hence, providing no evidence of an association. They also estimated the magnetic field strength in relation to the power lines, and for calculated fields exceeding 1 μT compared to <0.1 μT, ORs ranged from 0.68 (CI: 0.39-1.17) for melanoma to 1.08 (CI: 0.77-1.51) for female breast cancer, again showing no evidence of any association. Albeit the large study sample, one caveat is that the control group is also patients with cancer, assuming that those cancers included in that group were not related to magnetic field exposure.

Adult cancers were also investigated in a large number of studies in occupational settings, reviewed in IARC (IARC, 2002). The vast majority however lacks individual quantitative exposure assessment, and no consistent picture emerges. More recently, job-exposure-matrices (JEM) were constructed to better inform about exposure, and applying such a JEM to a multinational case-control study on brain tumours (subset of the Interphone study (Interphone, 2010)) showed no overall association between cumulative exposure to ELF (in μT-years), with ORs of 0.80 (CI: 0.63-1.00) and 0.89 (CI: 0.70-1.12) for glioma and meningioma, respectively, in the highest exposure category (Turner et al., 2014). For the shortest latency of 1-4 years, however, the OR for glioma was statistically significant elevated (OR 1.67, CI: 1.36-2.07), raising the question of an effect of ELF on tumour promotion. In a previous case-control study in France (Baldi et al., 2011), an association with ELF exposures was seen for meningioma, both occupational and residential (OR 2.99; CI: 0.86-10.40 for residential exposure; OR 3.02; CI: 0.92-2.51 for occupational ELF MF exposure).

Discussion on epidemiological studies

Pooled analyses of the more recent studies on ELF magnetic fields and childhood leukaemia confirm those of earlier studies, however, the new generation of studies shows little methodological advancement compared to the ones conducted before 2000. Therefore it remains difficult to judge whether the apparently quite robust empirical association is likely to be causal or a result of methodological shortcomings of the studies such as information bias, selection bias and confounding. In particular, low response rates among controls remain a concern. Identification of alternative explanations made little progress as well as finding further evidence for biological plausibility. In particular, a large study investigating childhood leukaemia survival in relation to ELF magnetic field exposure did not observe an association, adding no support to the hypothesis that ELF magnetic field may promote pre-leukemic clones both related to the risk of developing leukaemia as well as the risk of a relapse of leukaemia after successful treatment. Studies on other childhood cancers or adult cancers show no consistent associations.

It is important to note a common misunderstanding when interpreting the μT exposure levels used in the epidemiological studies. In all the childhood cancer studies mentioned above, the μT levels reflect average exposure measured over longer durations of up to several days, but not instantaneous exposure.

Conclusions on epidemiological studies

The previous assessment of the 2009 SCENIHR Opinion on a possible association between long-term exposure to ELF magnetic fields and an increased risk of childhood leukaemia remains valid. A positive association has been observed in multiple studies in different settings at different points in time. Little progress has been made in explaining the finding, neither in terms of a plausible mechanism for a causal relationship with magnetic field nor in identifying alternative explanations.

3.8.1.2. *In vivo* studies

What was known on this subject?

Overall, most animal studies do not suggest that magnetic fields can cause tumours or enhance the growth of implanted tumours. Nevertheless, one group has published several studies showing accelerated development of chemically-induced mammary tumours in Sprague-Dawley rats. The previous Opinion described a further study from that group showing comparable effects in Fischer 344 (F344) rats. Also described was a study using Wister rats that reported cytogenetic changes in bone marrow cells following long-term exposure to magnetic fields at 1 mT.

What has been achieved since then?

Previously, Löscher and co-workers have reported that rat (sub-) strains show different sensitivities to the effects of magnetic fields on the development of mammary tumours, and they suggested that genetic background plays a pivotal role in these responses. Fedrowitz and Löscher (2012) have explored this further, by comparing gene expression in the mammary glands of female F344 rats (which are considered to be a magnetic field-susceptible strain) and female Lewis rats (which are considered to be non-susceptible). Following continuous exposure to a horizontally-polarised, 50 Hz magnetic field at 100 µT for 14 days, the RNA samples from the mammary glands of 5 animals in each treatment group were pooled and analysed using a whole genome microarray. Only fold changes of 2.5 or more were considered of significance. Overall, the expression of 21 transcripts was found to be regulated by exposure: 9 were increased in Lewis rats, while 8 were increased and 6 decreased in F344 rats. Of these the most striking were the 832-fold decrease in α-amylase, 662-fold decrease in parotid secretory protein and 39-fold decrease in carbonic anhydrase 6 expression found in F344, but not in Lewis rats. The precise role of these transcripts in mammary tissues is largely unknown.

Fedrowitz et al. (2013) described a series of animal experiments performed over a four year period in which the activity and expression of α-amylase protein were determined in mammary tissues following exposure to 50 Hz magnetic fields at 100 µT for up to 28 days. For this analysis, the mammary glands were dissected into two samples, the cranial and caudal gland complexes. The first set of experiments found that exposure for 14 and 28 days resulted in a significant increase in amylase activity in the cranial mammary gland complex but not in the caudal complex in F344 rats. A significant increase was also seen in Lewis rats exposed for 14 days, but only in the cranial complex. A second experiment did not replicate these effects, and found that 14-day exposure of F344 rats resulted in no change in amylase activity in the cranial complex of F334 rats, and a significant increase in the activity in the caudal complex. For Lewis rats, exposure resulted in no changes in either gland complex. Protein expression of α-amylase, measured in one of these experiments, was significantly elevated in the caudal but not the cranial complex. These differences in activity between sets of experiments could not be explained, although it was noted that magnetic fields had increased α-amylase activity in both, not decreased it. In another set of experiments, no changes were found on α-amylase enzyme activity in serum of F344 rats following magnetic field exposures of 1, 7 or 14 days compared to sham-exposed controls. It was concluded that α-amylase might be a possible biomarker for magnetic fields effects, although it was acknowledged that it would be a difficult marker to use in animals because of its sensitivity to stress.

A few studies have used circularly polarised fields that are similar to the fields produced in the environment by some types of overhead powerlines. Negishi et al. (2008) investigated the effects of long-term exposure to magnetic fields on the incidence of chemically-induced malignant lymphoma/lymphatic leukaemia in mice. CD-1 mice were injected with 7,12-dimethylbenz(a)anthracene (60 μg/mouse) within 24 h of birth, and at 4 weeks of age were randomly allocated to a treatment group (each consisting of 50 males and 50 females). Animals were exposed in a dedicated exposure facility to 50 Hz, circularly polarized fields for 22 h/day, 7 days/week for 30 weeks at 7, 70 or 350 μT, and another group was sham exposed. The animals were checked daily for behaviour and clinical signs of morbidity, and any animal that died during exposure underwent an extensive histopathological examination, as did the remaining animals at the end of the exposure period. The experiment was repeated twice. For both experiments, whether examined separately or pooled, the cumulative proportions of exposed mice with malignant lymphoma/lymphatic leukaemia were not significantly different from those in the sham exposed groups, indicating that magnetic fields had not promoted chemically-induced lymphoma/leukaemia.

Two studies from the same laboratory report that long-term exposure of rodents to 60 Hz circularly polarised magnetic fields has no significant co-promoting effect on either chemically-induced tumours or spontaneous tumours in predisposed animals. In the first study, Chung et al. (2008) treated pregnant F334 rats on day 18 of gestation with ethylnitrosourea (ENU) (10 mg/kg) to induce brain tumours in the offspring. These animals were exposed to magnetic fields of up to 500 μT from age of 4 weeks for 21 h/day for up to 38 weeks. No consistent field-dependent changes were seen on survival rate, body weight, or haematology and no significant differences in tumour incidence were seen between the sham-exposed group and the 3 exposed groups. In the second study, Chung et al. (2010) exposed female AKR mice to magnetic fields of up to 500 μT for 21 h/day from 4-6 weeks of age for up to 42 weeks. Exposure was without consistent effect on any of the measured outcomes, including mean survival time, body weight, micronucleus assay, haematology values, or lymphoma incidence. Sporadic positive effects were noted in both studies but these were discounted due to a lack consistency.

Bernard et al. (2008) investigated the effects of 50 Hz magnetic fields on leukaemia using an animal model of childhood B-acute lymphoblastic leukaemia. Beginning when they were 3 months old, male WKAH/Hkm rats were given n-butylnitrosourea (BNU) in their drinking water 5 days a week for 24 weeks to initiate leukaemia. Animals were exposed in four replicate experiments to 50 Hz magnetic fields, both without and with harmonics at 150, 250 and 350 Hz, at 100 μT for 18 h /day, 7 days/week for 52 weeks. Another group of animals used a positive control were pre-treated with γ radiation before BNU treatment. To detect leukaemia, a range of haematological parameters and differential blood cell counts were measured, and immunophenotyping was performed to define the leukaemia phenotype. It was found that exposure both with and without harmonics had no effect on any of the other measured parameters, including survival, loss of body weight, cumulative incidence or type of leukaemia, but significant changes were obtained in the positive control group.

In order to gain insight into potential mechanisms whereby magnetic fields could affect the development of childhood leukaemia, Kabacik et al. (2013) investigated the effects of exposure to magnetic fields on bone marrow in young mice using three sensitive transcription methods. Juvenile animals (21 day old) were exposed for 2 h to a 50 Hz magnetic field at 100 μT and changes in gene expression in bone marrow were assayed 4 h after exposure using High Coverage Expression Profiling (HiCEP), Illumina arrays or quantitative real-time polymerase chain reaction (QRT-PCR). Four transcripts were identified using HiCEP as showing significantly different expression between exposed and sham-exposed mice: two of these (AK157520 and F10-NED) had no known function although one (Picalm) may be rearranged in human lymphoid and myeloid leukaemia. However, these differences were not confirmed using two different QRT-PCR assays or the microarrays, and it was concluded that no robust field-dependent changes had been seen. The authors commented on the difficulties of demonstrating small changes in gene

expression that may occur following *in vivo* exposure to magnetic fields that are due to inherent variability of biological responses and the technical limitations in the sensitivity of existing technologies.

Rageh et al. (2012) also investigated potential mechanisms into increased cancer risk and reported that continuous, sub-chronic exposure of young rats to 50 Hz magnetic fields could induce genotoxic and cytogenetic changes. In this study, rats were exposed at 0.5 mT using a solenoid-based exposure system for 30 days beginning when they were 10 days old. It was found that exposure significantly increased DNA damage in brain cells as measured by alkaline single cell gel electrophoresis and also increased micronucleus induction as measured by the numbers of polychromatic erythrocytes and micronucleated polychromatic erythrocytes in bone marrow. In addition, the mitotic index of bone marrow cells was increased, as were the levels of malondialdehyde and superoxide dismutase in brain cells suggesting exposure had increased lipid peroxidation and oxidative stress.

Discussion on *in vivo* studies

Motivated by the observed increased leukaemia risk in children, experimental studies have investigated the carcinogenic potential of magnetic fields using animals. These studies have tended to use traditional rodent models and do not support the epidemiological findings. However, these experimental studies suffer from a number of limitations (Lagroye et al., 2011). Firstly, the absence of appropriate animal models for childhood leukaemia is of concern. Most studies have not used directly relevant models, although one recent study did use a rat model of B-cell acute lymphoblastic leukaemia and this did not find any field-dependent effects on leukaemia. However, mouse models of acute lymphoblastic leukaemia are now becoming available, such as the ETV6-RUNX1 (TEL-AML1) model (e.g. Schlindler et al., 2009; van der Weyden et al., 2011) and it is expected that improved models should become accessible in the near future (Ziegelberger et al., 2011). Studies with these models should be a research priority. In addition, few studies have also been carried out with exposures during gestation, when the initial events are considered to occur in acute lymphoblastic leukaemia, so future studies should include this possibility. Further, it is possible that the exposure conditions used in experimental studies were far from optimal to reveal effects, because the biologically-relevant factor(s) not been identified, although many studies have used fields well in excess of values commonly found in the everyday environment. Finally, the possibility of strain-specific increases in sensitivity to magnetic fields is intriguing, and could lead to the identification of biomarkers, and this should be investigated further. All experiments should be of sufficient size and sensitivity to adequately detect an effect of a predefined size to avoid the possibility of type II errors.

Conclusions on *in vivo* studies

Previously SCENIHR (2009) concluded that animal studies did not provide evidence that exposure to magnetic fields alone caused tumours or enhanced the growth of implanted tumours. The inclusion of more recent studies does not alter that assessment. In addition, these studies do not provide further insight into how magnetic fields could contribute to an increased risk of childhood leukaemia.

3.8.1.3. *In vitro studies*

What was already known on this subject?

The previous SCENIHR Opinion observed that some studies indicated that ELF magnetic fields alone and in combination with carcinogens induce both genotoxic and other biological effects *in vitro* at flux densities of 100 µT and higher. It was further noted that there is a need for independent replication of certain studies suggesting genotoxic effects and for better understanding of combined effects of ELF magnetic fields with other agents and their effects on free radical homeostasis.

What has been achieved since then?

In vitro studies may be relevant for assessment of ELF MF effects on neoplastic diseases, depending on the cell type used, endpoints investigated, and the exposure. Although there are a substantial number of *in vitro* studies published in the scientific literature, only a fraction is relevant for the present Opinion. Relevant study endpoints include genotoxicity (genetic damage), cell proliferation, cell survival and death, cell differentiation and transformation, signal transduction events, acute effects on ion homeostasis (especially Ca2+), and radical homeostasis. Not all of these endpoints are represented in the literature that was used for the present assessment.

Vijayalaxmi and Prihoda (2009) published a meta-analysis on genetic damage in mammalian somatic cells exposed to ELF MF. Their analysis included data from 87 separate original publications (from 1999-2007). The studies included *in vitro* as well as *in vivo* animal studies, and also data from human occupational studies. Since all data were pooled in the paper, it is not possible to specifically analyse the contributions from *in vitro* studies to the meta-analysis, but the author´s descriptions of the material suggest that the majority of the studies were *in vitro* studies. The meta-analysis considered the ELF MF-related exposure characteristics (frequency, flux density, occupational exposure) and four genotoxicity endpoints (DNA single and double-strand breaks; chromosomal aberrations; micronuclei; sister-chromatid exchanges). The most commonly employed frequency was 50 Hz and fields with a flux density of 1 mT were predominantly used in the studies. Most of the studies investigated only one endpoint. The meta-analysis revealed that a small but statistically significant difference was present between MF-exposed and control cells, with an increase in genetic damage at certain exposure conditions. Mean indices for chromosomal aberrations and micronuclei for both exposed and control cells were similar to the levels seen in a historical database. The authors also concluded that publication bias (underreporting of negative findings) was evident in the studied material.

A study by Kim et al. (2010) documented that an early marker of DNA double-strand breaks (phosphorylated H2AX) and the down-stream effector Chk2 (checkpoint for DNA damage during progression of the cell cycle) both were induced by a 30-min exposure to a 60 Hz MF (6 mT) in human IMR90 lung fibroblasts and HeLa cells. A repeated exposure (30 min during each of three consecutive days) also led to induction of apoptosis (Caspase-3 activation) in both cell types. The study lacks a proper sham exposure and other essential information regarding exposure is lacking. Significantly better described is the study from Focke et al. (2010) where they replicated an earlier study by Ivancsits et al. (2003). The main finding in the older study was that intermittent exposure to 50 Hz MF increased DNA strand breaks in primary human fibroblasts. Focke and co-workers used the alkaline Comet assay to detect DNA strand breaks in normal human fibroblasts from three different donors and in HeLa cells. Exposure consisted of a 50 Hz MF (1 mT) for 15 h. Importantly, the exposure was either continuous for 15 h, or intermittent (5 min on, 10 min off) during the 15 h period. A small but statistically significant increase in DNA damage was seen after the intermittent exposure, but only in the fibroblasts. Furthermore, the authors provide evidence that suggests that the effect is from the MF, and not from any induced E-field. In addition, the response to MF was different than the one obtained after H2O2 treatment, suggesting that the primary effect on DNA is not coming from increased levels of oxygen radical species. The study also indicates that the noted DNA damage is due to an MF-dependent induction of apoptosis in a subpopulation of cells.

These recent studies on genotoxicity suggest that exposures to ELF MF at 1 mT or higher exert at least modest DNA-damaging activity in cultured human cells.

A series of studies regarding effects of weak 50 Hz MF on proliferation have recently been published by a Spanish group. Thus, Trillo et al. (2012) studied proliferation, DNA synthesis and DNA and protein content in two human cell lines (neuroblastoma NB69 and hepatocarcinoma HepG2 cells) exposed to a vertical 50 Hz MF. Exposure was intermittent (3 h on and 3 h off) for 42 h, in the presence or absence of all-trans-retinol. MF alone (0.10 mT) enhanced proliferation in both cell lines, whereas MF and retinol together

caused different (opposing) effects in the two cell lines. The authors followed up this study in 2013 (Trillo et al. 2013) where proliferation and proliferation markers were investigated in the NB69 cells. The previous effects of a 0.10 mT MF exposure (intermittent; 3 h on and 3 h off; 42 h) were confirmed. Also a weaker MF (0.01 mT) caused similar responses on cell number (increase 12.5 and 14.8 % compared to sham controls for 0.01 and 0.10 mT respectively). The MF effects were again counteracted by retinol addition. The importance of intermittency for proliferation effects in this cell line was documented in another study (Martinez et al. 2012). The NB69 cells were here exposed for 63 h to a 0.10 mT MF, either continuously or intermittently (5 min on and 10 min off; or 3 h on and 3 h off). Only intermittent exposure caused significant increases (10-15% increase compared to sham control) in percent of cells in the S-phase of the cell cycle and increased cell number. Finally, the group also published a study where the effects of a 0.01 mT MF (intermittent exposure; 3 h on and 3 h off; 24, 42, or 90 h) on proliferation in HepG2 cells were investigated. Also this lower flux density increased proliferation, and decreased levels of differentiation markers. However, the MF effects were prevented by melatonin in physiological concentrations (10 nM).

In summary, the studies from this group have documented proliferation stimulating effects of intermittent, but not continuous, exposure to 50 Hz MF at low flux densities (0.01 and 0.10 mT respectively).

Another proliferation related study has been published by Basile et al. (2011), who found that a 50 Hz MF (30 A/m; no flux density value was given) for 6 h did not influence ROS levels, hsp70 protein levels, or apoptosis, but did increase the levels of the anti-apoptotic protein BAG in two human melanoma cell lines.

Zhang and co-workers also evaluate the effect of MF exposure on cell proliferation and phenotype. They exposed human epidermal stem cells (hESC) to a 5 mT at 1, 10, and 50 Hz for 30 min/day for 3, 5, or 7 days. At the third, fifth, and seventh day of exposure cell proliferation resulted significantly enhanced in a frequency-dependent manner, with the highest cell proliferation rate at 50 Hz. Such an increase was due to an increased percentage of cells in the S phase of the cell cycle, with a decrease in the percentage of cells in the G1 phase, examined seven days after exposure by flow cytometry. Moreover, 7 days exposure did not modify cell morphology and cell-surface antigens, suggesting lack of effects on cell phenotype (Zhang et al., 2013).
Marcantonio et al. (2010) focused on all-trans-retinol-induced neuroblastoma BE(2) cells. Retinol treatment caused increased levels of several differentiation markers (neurite outgrowth; expression levels of the genes for p21, cdk5, cyp19). These effects were enhanced by simultaneous exposure to a 50 Hz MF (1.0 mT; 24-72 h). The MF exposure also caused a decrease in cell number, due to an increased proportion of the cells in the G0/G1 phase.

The mouse fibroblast cell line NIH3T3 is a useful tool for investigation of carcinogenic effects of chemicals and physical agents since a possible transformation of the cells cause an increase in colony formation, which can be quantified. Lee and co-authors (2011) exposed these cells to a 60 Hz MF (1.0 mT) for 4 h. MF exposure was either alone or in combination with ionizing radiation, H2O2, or c-Myc overexpression. The transformation potential of none of these agents was influenced by the MF exposure.

The question of MF-effects on differentiation and gene expression was also addressed by Sulpizio et al. (2011). The authors exposed human SH-SY5Y neuroblastoma cells for 5, 10, or 15 days to a 50 Hz, 1.0 mT sinusoidal MF. Besides analysis of cell number, viability and proliferation (which all increased in exposed compared to control cells), the main endpoint was a proteome analysis. A number of common protein spots were found, of which 86 unique proteins were identified and classified. Proteins belonging to the group of cellular organization and proliferation, and the group of cellular defence mechanism underwent the largest changes (increase) in cells exposed for 15 days. Regarding individual proteins, 3 new proteins appeared in cells exposed for 10 days, and an additional 6 new proteins were detected in samples exposed for 15 days. These altogether 9 new proteins belong to the groups of cellular organization and proliferation,

and to cellular defence mechanism. The authors argue that the protein changes correspond to the changes in proliferative potential seen after exposure, and that this reflects a phenotypic shift towards a more undifferentiated state.

The pineal hormone melatonin exerts anti-proliferative effects on estradiol-stimulated breast cancer cells *in vitro*. This observation was previously used in several studies where the effect of weak sine-wave 50 or 60 Hz MF was investigated (see SCENIHR 2007, 2009). Even at very low flux densities (1.2 µT), it was shown that the melatonin-inhibition of proliferation was counteracted by MF exposure. Recently, Girgert and co-workers (Girgert et al. 2009) have extended these studies. Here the authors employ a variant of MCF-7 breast cancer cells that are transfected with the gene for the melatonin-receptor MT1, and thus very sensitive to melatonin-treatment. In estradiol-treated cells, melatonin decreased binding of the transcription factor CREB to the promoter of BCRA-1, and also decreased mRNA levels of BCRA-1, p53, p21WAF and c-myc. Exposure to a 50 Hz MF (1.2 µT; various exposure times dependent on end-point investigated) counteracted these melatonin-effects and also the proliferation inhibition exerted by melatonin. Thus in several studies, this group reported significant effects at flux density levels far below those used by most other authors.

Discussion on *in vitro* studies

In summary, a number of *in vitro* studies published over the past years are relevant for the question of ELF MF exposure and neoplastic disease. However, the studies are too few and too scattered in scope and approach to provide any foundation for a conclusion on the possible neoplastic effects of ELF MF exposure. Furthermore, the studies do not provide any conclusions regarding mode of action for effects of ELF MF.

However, some studies provide interesting findings that justify additional research efforts. Thus, there are indications that DNA damage occurs in cultured human cells during certain exposure conditions. Effects are primarily noted at a flux density of 1 mT or higher. Even at lower flux densities (0.10 mT and below), MF exposure has been shown to stimulate proliferation. The effect can possibly be related to effects on signal transduction and gene expression.

An intriguing observation is that certain studies report exposure effects due to intermittent, but not due to continuous exposure. The area has not received much attention, but can be an opener of studies into mechanisms.

Conclusions on *in vitro* studies

As concluded in the previous SCENIHR Opinion, data suggest that ELF MF may induce both genotoxic and other biological effects *in vitro* at flux densities of 100 µT and higher. The mechanisms are not established and the relevance for a connection between ELF MF exposure and childhood leukaemia is unclear.

3.8.1.4. Conclusions on neoplastic diseases

The new epidemiological studies are consistent with earlier findings of an increased risk of childhood leukaemia with estimated daily average exposures above 0.3 to 0.4 µT. As stated in the previous Opinions, no mechanisms have been identified and no support is existing from experimental studies that could explain these findings, which, together with shortcomings of the epidemiological studies prevent a causal interpretation.

3.8.2. Nervous system effects and neurobehavioral disorders

3.8.2.1. Epidemiological studies

What was already known on this subject?

The previous SCENHIR Opinion indicated a possible increase in Alzheimer's disease arising from exposure to ELF, stressing the need for further epidemiological and laboratory investigations.

What has been achieved since then?

A cohort study found no indication of an increased mortality from motor neuron disease related to employment in electronic work (Parlett et al. 2011). The U.S. National Longitudinal Mortality Survey with 300,000 people followed up from the early 1980's was analysed. Exposure assessment relied on job titles at baseline, with further classification based on a previously constructed job-exposure matrix. Causes of death were obtained from the National Death Index. Information on several potential confounders including race/ethnicity, education and income was available. Despite the large cohort size, only 40 deaths from motoneuron disease occurred during an average of 8.8 years of follow-up. The crude hazard ratio was somewhat above unity, but after adjustment it indicated no excess among the quartile of population with the highest potential for ELF exposure (HR 0.98, 95% CI 0.39-2.50). No gradient across exposure strata was found. The study was limited by the relatively crude exposure assessment, and low statistical power due to small number of events.

A Danish registry-based case-control showed no association between residential exposure to power lines and risk of Alzheimer or Parkinson disease (Frei, et al. 2013). The cases (nearly 2000 with motor neuron disease, 8000 with multiple sclerosis, 16,000 with Parkinson disease and 20,000 with Alzheimer) were identified from the nationwide hospital discharge registry and matched controls population registry. Residential history was constructed for the past 20 years and distance from high-voltage power lines was calculated using geographical information system for about 90% of the subjects. Information was also available on marital status, education and income (the latter two at small area unit level). No indication of increased risks were found for ever having lived <50 m from a high-voltage power line, nor for duration of such residency. Only in a sub-group analysis of Alzheimer disease in the age group 65-74 years, an association was reported (HR 1.92, 96% CI 0.95-3.87). The results did not confirm the findings of the Swiss cohort study reporting increased risks of Alzheimer disease for living 15 years within 50 m of a power line.

A meta-analysis of 17 studies on occupational ELF exposure and amyotrophic lateral sclerosis found some evidence for an increased risk, but the findings were not consistent and indications of publication bias were detected (Zhou et al. 2012). The summary analysis showed elevated risk in case-control studies (OR 1.39, 95% CI 1.05-1.84), but not cohort studies (RR 1.16, 95% CI 0.80-1.69). Similarly, increased risk was indicated in studies using clinical diagnosis of ALS, but not in those relying on death certificates. Asymmetric funnel plots and Egger's test indicated an excess of small studies with increased risks, suggesting publication bias. The Swedish twin study with 216 cases (2/3 classified as Alzheimer's disease) showed elevated risks for occupational exposures exceeding 0.12 µT only for the subgroups with age of onset less or equal to 75 years and for manual workers but not the entire study population (Andel et al., 2010). Furthermore, there was no exposure-effect gradient, i.e. the findings suggest a protective effect of low exposure.

A meta-analysis covered 42 studies on occupational ELF exposure and neurodegenerative disease (Vergara JOEM 2013). Only PubMed was searched, no other databases and only publications in English language were included. Of the 27 case-control and 15 cohort studies, only three employed measurements of ELF, five used classification by an industrial hygienist and 14 JEM or similar exposure tabulations. Twelve of the studies reported selectively only some of the findings. Total number of cases for various outcomes was not reported. Overall, occupational ELF showed associations with motoneuron disease and Alzheimer's disease (pooled summary ORs 1.26-1.27), but not with Parkinson's disease, multiple sclerosis or all dementias. Studies with disease prevalence as outcome showed stronger associations than those addressing incidence or mortality. Mortality studies showed also significant though smaller associations, while

incidence studies showed no excess risk of AD and a non-significant increase in MND. Studies with assessment of level of ELF exposure showed less evidence of an effect than those relying on job titles alone for motoneuron disease, while for Alzheimer's disease no such difference was observed. The few studies with measured ELF fields showed some elevation in risk for both AD and MND, though not significant. In multiple metaregression, only prevalence studies showed significant association after adjustment for various study features (exposure assessment, source of outcome data, selective reporting, source of funding). There was evidence of selection bias with smaller studies showing larger effects, in particular for Alzheimer's disease. The authors interpreted the findings providing only limited support for an association of ELF with neurodegenerative diseases. The results were not consistent and some effect of publication bias was shown. In most analyses, studies with the most robust features of design or conduct did not show more evidence of effect than less reliable studies. Overall, there seemed to be more evidence for an association of ELF with AD than MND (cohort studies, studies without indication of selective reporting and studies assessing level of ELF), but this could be partly attributable to publication bias. Even if they do not provide definitive evidence, the findings by no means demonstrate the lack of an association.

A case-control study assessed the relationship between occupational ELF exposure and cognitive dysfunction (Davanipour et al. 2014). The subjects were 3050 Mexican Americans aged 65 or older interviewed in their homes in five US states in 1993-1994 as the baseline survey of H-EPESE study. Mini-Mental state exam was used to evaluate cognitive performance, with score <10 classified as severe cognitive dysfunction. This resulted in low prevalence of dementia (1.6%). Exposure was assessed based on a single job title only, no occupational histories were available. Also, any job title could be reported, as it was not defined as the most recent or the longest held occupation. Five occupations were classified as involving high exposure (average >10 mG or peak >100 mG, 3.5% of the subjects), but practically all subjects in the high exposure group were seamstresses (87 out of 105) or welders (14 subjects).

The prevalence of severe cognitive dysfunction was 2% in the low exposure group, 1% in the intermediate and 5% in the high exposure group. The crude odds ratio for dementia related to high occupational exposure was 3.4 (95% CI 1.3-8.9). The results were based on five exposed cases only in the "high" exposure category. No meaningful adjustment could be performed due to the small number of observations.

No analyses were presented for mild or moderate dementia. Different types of dementia (Alzheimer and vascular dementia) were not considered separately. No information was obtained on some risk factors and potential confounders such as family history of dementia, or medical history including diabetes, depression, cholesterol, blood pressure or obesity. Heavy alcohol use predisposes to dementia and the question regarding "ever consumption" of alcohol is unlikely to capture the relevant information. No association was found with education (<12 vs. 12 or more years), which is surprising.

Conclusions on epidemiological studies

Only few new studies have been published since the previous Opinion. Although the new studies in some cases have methodological weaknesses, they do not provide support for the previous conclusion that ELF MF exposure increases the risk for Alzheimer´s disease.

3.8.2.2. Neurophysiological studies

What was already known on this subject?

The previous Opinion summarizes from animal studies that there is some evidence for effects on the nervous system from ELF magnetic fields above about 0.1-1.0 mT. It is stated that there are still inconsistencies in the data, and no definite conclusions can be drawn concerning human health effects.

What has been achieved since then?

Since the last Opinion, 13 papers on effects of extremely low frequency magnetic fields (ELF MF) on human brain function (EEG, functional imaging and behavioural outcomes) have been published. Four of these studies (Perentos et al. 2008, Shafiei et al. 2012, Amirifalah et al. 2013, Shafiei et al. 2014) were noted but not considered for the present Opinion due to insufficient information on exposure and/or dosimetry.

Since mobile phones, in addition to RF, also emit ELF MF of varying spectra depending on the operating mode, several groups investigated effects of various real or simulated magnetic fields as generated by the circuitry of GSM handsets while transmitting. Schmid et al. (2012b) and Tommaso et al. (2009) investigated both RF and ELF effects on the sleep EEG and event related potentials during wake, respectively (see 3.6.2.2). Here studies are reported in which only effects of ELF signals are studied.

The effect of short-term ELF MF exposure (2 min) with varying ELF frequencies (50, 16.66, 13, 10, 8.33 and 4 Hz) was analysed (Cvetkovic and Cosic 2009) based on 1-min recordings with regard to stimulation specific outcome frequency bands: stimulation with 16.33 Hz: beta2 (15.5-17.5 Hz), stimulation with 13 Hz: beta1 (12-14 Hz), stimulation with 10 Hz: alpha2 (9-11 Hz), stimulation with 8.33 Hz: alpha1 (7.5-9.5 Hz) and stimulation with 4 Hz: theta (3-5 Hz). The magnetic flux density generated by Helmholtz coils was 20 µT. The sample comprised 33 healthy subjects (24 males and 9 females) in the age range 20-59 years. The study was double-blind with a cross-over design (sessions performed consecutively at the same day separated by a 30 min break). Out of 320 post-hoc t-tests (16 electrodes, five bands/stimulation, two exposure conditions and two test sessions) none was significant after Bonferroni adjustment of the alpha level. A Bonferroni adjustment to 80 tests (16 EEG recording sites* five paired stimulation frequencies and outcome EEG frequency bands, tests of interaction terms were not considered in this adjustment) lead to five statistically significant results, which the authors discuss in the context of exposure. However, none of these results reflected an exposure effect. One of the results indicated a pre-/post difference independent from exposure and four were related to interaction effects of condition and session. Exposure as such did not lead to a statistically significant result.

Legros et al. (2012) analysed the effect of an exposure to a 60 Hz 1.8 mT ELF MF for the duration of one hour as compared to sham in 73 subjects (46 males and 27 females, 28 ± 9 years) using a double blind counterbalanced cross-over design with test sessions on separate days. The magnetic fields were generated by Helmholtz-like coils of 1.6 m diameter. Each test session lasted 105 min and was composed of four 15 min test sessions (two under exposure, one before and one following exposure) separated by 15 min rest. The test battery included resting EEG analysed at 8 sites (2 min eyes open, 2 min eyes closed), postural tremor assessment (1 min eyes open, 1 min eyes closed), voluntary hand movements and postural oscillations (30 sec eyes open, 30 sec eyes closed). None of the EEG variables, the tremor variables and the voluntary alternating hand movement variables showed a significant effect of exposure as main factor. After restricting the frequency range of oscillations from 2 – 20 Hz to 7 – 12 Hz a significant session*block interaction was observed, suggesting an increase of tremor power in this frequency band after 45 min of MF exposure. The results of a repeated measures ANOVA showed a significant session*block*eyes effect for three out of seven variables characterizing standing balance data. Sway velocity was lower under ELF MF exposure in the eyes closed condition only as compared to sham. These results, which need replication, suggest the possibility of a MF effect leading to a small increase in tremor amplitude in a restricted frequency range and to a small decrease in standing balance oscillations when eyes are closed.

The same group (Corbacio et al. 2011) investigated a possible effect of a 60 Hz 3mT exposure (30 min duration) on 15 outcome parameters of 10 psychometric tests (see Table 7) in a sample of 99 subjects (60 females and 39 males, 18-49 years) assigned randomly to one of three exposure conditions: sham/sham; sham/MF exposure, and MF exposure/sham (parallel-group design). The homogeneous magnetic field generated by Helmholtz like coils was perpendicular to the sagittal plane. A double blind design was

used (personal communication). A statistically significant ($p = 0.01$) interaction effect was seen for one out of the 15 variables. The score of the digit span forward test did not show a practice related improvement (which was seen under sham exposure and which was observed for 11 out of the 15 variables) under both exposure conditions.

Capone et al. (2009) investigated the effect of a 45 min ELF pulsed magnetic field exposure on brain function in 22 subjects (9 males and 13 females, 27.6 ± 9 years). 14 of these subjects underwent a single-blind true or sham exposure in a randomized cross-over design. Eight subjects only received the true exposure. The ELF magnetic fields were delivered by a thin ring-shaped coil positioned horizontally around the head. The coil was driven by rectangular voltage pulses of 1.3 ms at 75 Hz resulting in a peak flux density of 1.8 mT. Cortical excitability was measured using transcranial magnetic stimulation. The observed effect (increase of intracortical facilitation –ICF- after true exposure) is not warranted since they used a wrong statistical analysis paradigm not taking into account the paired nature of the data. They compared sham exposure results from a subsample of 14 subjects to true exposure results all 22 subjects.

Robertson et al. (2010) used functional brain imaging to investigate a possible effect of low-intensity low-frequency magnetic fields on neuroprocessing. In a parallel group design 31 subjects in the age range of 18 – 60 years were included in the study and either assigned to a sham group (17) or to a true-exposure group (14). A complex sequence of ELF magnetic bursts with varying time intervals resulting in a spectrum containing frequencies from DC up to 300 Hz was used. Magnetic fields were generated utilizing the Z-axis gradient coil of the MRI scanner. The flux density (or its gradient) was set in order to reach the 200 µT amplitude as used in previous studies at the level of the subject’s eye brow. These fields are much lower than ELF MF fields generated during the fMRI measurement. Subjects received acute thermal pain stimuli at the hypothenar region of the right hand. Significant interactions have been observed between pre- and post-exposure activation between sham and true exposure for several brain areas, indicating that ELF MF might induce neuromodulation.

<u>Other human studies</u>

There are two studies from the same group investigating effects of a 1800-µT, 60 Hz exposure (McNamee et al. 2010) and a 200-µT, 60 Hz exposure (McNamee et al. 2011) on skin blood perfusion, skin temperature, heart rate and heart rate variability. McNamee et al. (2010) recruited 58 self-reported healthy volunteers (19 females and 39 males, mean age: 27 ± 8.5 years) between 18 and 55 years. Two sessions (sham and real exposure) were applied consecutively in a double-blind, counterbalanced cross-over design. Each session lasted 105 min and consisted of four 15-min measuring blocks, which were interspaced by three 15-min “rest” periods. In the real exposure condition, exposure was turned on after the first measurement block for 1 hour, the last measurement block in the real exposure situation was made 15 min after the exposure had been switched-off. Exposure was delivered by two coils (diameter: 1.6 m; distance between coils 1.2 m). They were enclosed in water pipes and temperature was stabilized. Subjects were seated between the coils with their head in the centre of the quasi-homogeneous field. Field distribution was calculated and measured. The cardiovascular parameters were not affected by the 1800-µT, 60 Hz MF in this study.

In a pilot study with 10 subjects between 18 and 55 years (mean age: 24.0 ± 3.0 years) McNamee et al. (2011) investigated possible effects of a 200-µT, 60 Hz exposure on skin blood perfusion and heart rate. This study was single-blinded. Sham and real exposure were applied counterbalanced on separate days. The exposure system was the same as described in the McNamee et al. (2010) study (see above). Each exposure session lasted for 86 min and comprised four periods of measurement, each lasting 3 min. After 5 min of seated rest a first baseline measurement was performed, than the exposure was started for the next 60 min. After 15 and 45 min measurements were made for a 3-min period. During these measurement periods the MF was switched off. A final measurement

was made 15 min after the end of exposure. The MF used in this study did not affect skin blood perfusion, heart rate, and mean arterial pressure.

In a double-blind randomized cross-over study Kim et al. (2013) investigated possible effects of a 32 min exposure of the head to a 60 Hz 12.5 μT magnetic field on perception, eight subjective symptoms and physiological changes. The sample comprised two groups: a) 30 adults (15 females and 15 males, 27.9 ± 5.9 years), and b) 30 teenagers (16 females and 14 males, 14.8 ± 1.4 years). The magnetic field was applied to the head by a pair of coils (distance 50 cm between the coils) specially constructed for this study. Background fields were controlled. Assessment of heart rate, respiration rate and heart rate variability was performed for a duration of 5 min at four times: pre-exposure, after 11 and 27 min of exposure, and post exposure. Data analysis is based on comparison of changes from baseline (pre-exposure measure) by exposure separately for the two age groups. Physiological parameters and subjective symptoms were not affected in either group, and neither group correctly perceived the magnetic fields.

Another study which aimed at analysing resting blood pressure was noted but not considered due to insufficient dosimetric information is Rikk et al. (2013).

Conclusion on neurophysiological studies

The approaches to investigate possible effects of exposure on the power spectra of the waking EEG are quite heterogeneous with regard to applied fields, duration of exposure, number of considered leads, and statistical methods. Therefore, these studies are not useful for drawing meaningful conclusions. The same is true for the results concerning behavioural outcomes and cortical excitability.

3.8.2.3. *In vivo* studies

What was already known on this subject?

The previous Opinion of 2009 described further studies that suggested that the long-term exposure of rodents to 50 Hz magnetic fields may have an effect on memory and anxiety, and may affect the antioxidant defence system of the brain. The direction of the behavioural effects appears to depend on the characteristics of the applied field, but the important parameters are still poorly defined. Another study reported magnetic field exposure was without effect on a mouse model of ALS.

What has been achieved since then?

Studies have continued to use behavioural methods to investigate the effects of magnetic fields on memory and anxiety in animals: other studies have investigated the use of target-specific treatments. There are an increasing number of isolated studies using novel methodologies which present a greater challenge to synthesize the results into a coherent picture.

Jadidi et al. (2007) reported acute exposure to 50 Hz magnetic fields impaired consolidation of spatial memory in rats. Rats were given two blocks of training trials on the same day on the standard (spatial memory) version of the water maze task or in a cued version (where the location of the escape platform was indicated by a visible ball) and a probe trial was conducted two days later to measure memory. The animals were immobilized during field exposures and their heads placed within a small coil electromagnet for 20 min. For the spatial task, it was found that exposure at 8 mT, but not 2 mT immediately after training impaired performance in the probe trial, whereas exposure at 8 mT immediately before the probe trial had no effect, suggesting exposure had not impaired retrieval. For the cued task, exposure at 8 mT immediately after training had no effect on performance in the probe trial. None of the exposure had any effect on motor performance of the task.

Cui et al. (2012) reported that exposure of mice to 50 Hz magnetic field at 1 mT for 4 h/day for 12 weeks did not cause any changes in behaviour in an open field, but resulted in significant impairments in learning in both the spatial version of the water maze task

and in a cued version. Training occurred over four days, as is standard, but a probe trial (without the escape platform being present) was not performed to measure spatial memory. In addition, exposure was reported to affect markers of oxidative stress in the hippocampus and striatum (the activities of catalase and glutathione peroxidise (GPx) were decreased, and the concentration of malondialdehyde (MDA) was increased). Exposure at 0.1 mT was without any significant effect.

Li et al. (2014) investigated the effects on long-term exposure to a magnetic field on spatial memory in Sprague-Dawley rats. Continuous exposure to a 50 Hz magnetic field at 100 μT for 90 days had no effect on the acquisition of the task in terms of either escape latencies or swimming distances, nor did this exposure have any effect on task performance during a second training session. During the probe trial conducted at the end of each training session, exposure had no significant effects on behaviour, with all groups showing comparable memory. This suggests that long-term exposure had no significant effects on either acquisition or retention of the task.

Wang et al. (2013) explored the effects of 50 magnetic fields on spatial memory in juvenile outbred albino Kunming mice. Animals were exposed at 2 mT for 60 min/day from postnatal day 23 to 35 and their behaviour was measured using a Y-maze and water maze. It was found that exposure had no significant effect in the Y-maze, but acquisition of the water maze was significantly improved as was retention of the location of the escape platform measured in a probe trial. However, as was acknowledged by the authors, the sham-exposed animals showed poor retention in the probe trial, at no better than chance levels. Exposure no significant effect on body weight, or on swim speed in the water maze.

Duan et al. (2013) investigated the effects of intense magnetic fields on water maze behaviour in ICR mice. Animals were exposed using a pair of Helmholtz coils to a 50 Hz field at 8 mT for 4 h/day for 28 days. Temperature in the exposure cage was maintained at 23°C and the cage itself was decoupled from the coils. The exposed animals took significantly longer to escape from the water, and during the probe trial spent less time in the target quadrant and swam a greater distance than controls, indicating impairments in learning and memory for the task. Exposure was also associated with increased oxidative stress in the hippocampus, including increased reactive oxygen species (ROS) production and decreased superoxide dismutase (SOD) and GPx activities. In addition, the morphology of the hippocampus was changed in exposed animals with a decrease in numbers of neurons. Exposure gradually reduced body weight, becoming significant after 14 days. All these effects were attenuated by repeated, daily administration by gavage of a solution of procyanidin compounds extracted from lotus seedpods. This was attributed to the antioxidant and free radical scavenging abilities of the extract.

Effects on consolidation of a non-spatial, passive avoidance step-down task were reported by Foroozandeh et al. (2013). Adult male and female mice were conditioned using mild electric foot shock to avoid stepping off a small platform. Immediately after this conditioning trial, animals were exposed to a 50 Hz magnetic field at 8 mT for 4 h using a water-cooled electromagnet with forced ventilation. A retention test was performed 24 h later, when it was reported that exposed animals showed significantly decreased step-down latencies compared to sham exposed controls suggesting exposure had impaired the long-term memory of the task. However, this conclusion seems premature. The data are only presented as mean values with no indication of variation, but the mean step down latency of the control mice appears very short compared to published values for this test, and is only around 1 s longer than the value in the conditioning trial. Similarly, the mean step down latency of the exposed animals following treatment is shorter than that in the conditioning trial, which also seems unusual, suggesting exposure had affected more than just consolidation processes. The reason why the controls and exposed animals did not behave as expected was not discussed. The animals were sham-exposed by being placed in the inactive electromagnet for 4 h and although this produced similar results to untreated controls, no comparisons between treatment groups were performed.

Two studies from the same group have studied effects of magnetic fields on anxiety and stress. Balassa et al. (2009) investigated whether single, acute exposure to a 500 µT field for 20 min had effects on behavioural anxiety and social interaction in adult, male Sprague-Dawley rats. Behaviour was measured in an elevated-plus maze (EPM) immediately after exposure, while the exploration of a novel object placed in the home cage was measured for 10 min immediately after exposure (groups of 10 different animals were used in each test). Differences in behaviour were recorded in the maze, with the exposed animals moving less than controls, plus the number of open arm entries and time spent in the open arms were significantly decreased. Differences were also seen with the novel object, with exposed animals approaching and exploring the object less than controls. Two tests of social interactions were also carried out, and no differences in behaviour were seen: in one test, the animals were placed in a neutral environment with an unfamiliar rat for 10 min each day for 5 days; in the other, a rat was placed in the cage of a larger male rat for 10 min. In the second study, Szemerszky et al. (2010) investigated the effects of repeated, short-term and continuous long-term exposures. Animals were exposed to a 50 Hz magnetic field at 500 µT for 8 h/day for 5 days or for 24h/day for 4-6 weeks. Neither short- nor long-term exposure produced significant changes in behaviour in the EPM measured 48 h after terminal of short-term exposure, or 48 h before the end of long-term exposure. After 4 weeks of continuous exposure, animals were tested in a forced swim test, and exposed animals spent significantly longer floating (as opposed to swimming or struggling to escape), suggesting enhanced depression-like behaviour. No effects were found on body weight or on the weight of the thymus or adrenal glands, nor did either exposure produce differences in haematocrit levels. Blood glucose levels were unaffected by short-term exposure, although they were significantly elevated after 6 weeks exposure. Plasma levels of ACTH and corticosterone were determined along with pre-proopiomelanocortin (POMC) mRNA levels in the adrenal gland to measure the activation of the hypothalamic-pituitary-adrenal (HPA) axis: only POMC mRNA levels were significantly elevated after 6 weeks exposure. It was concluded that long-term exposure may be a mild stress to rats because it had produced a few signs of chronic stress; however, many markers were unaffected. For both studies, the exposure system consisted of a pair of Helmholtz coils, but the noises or vibrations produced are not described.

He et al. (2011) reported magnetic field effects on behavioural anxiety and spatial memory in rats that depended on the length of exposure each day. Sprague-Dawley rats were exposed to 50 Hz magnetic fields at 2 mT for 1 h or 4 h per day; they were tested in an open field and an EPM after 3 weeks, and in a water maze after 4 weeks. In all tasks, it was found that exposure for 1 h per day was without significant effect, but exposure for 4 h caused increased levels of behavioural anxiety in both tests, and reduced the latency to find the platform in the water maze and improved retention in the probe trial. The noise and vibration levels from the exposure coils were not reported and it is possible they could have an influence on the measured outcomes.

Korpinar et al. (2012) reported that long-term, continuous exposure of Wistar rats to a 50 Hz field at 10 mT resulted in a significant increase in behavioural anxiety, as measured in an EPM, but there was no effect on activity and exploration, as measured using a hole board. Animals were exposed for 21 days using a series of solenoid coils placed beneath the holding cages. An air gap was used to uncouple the cages from the coils, and wooden plates were used to insulate the cages from the heat generated by the coils, although the success of these measures was not recorded.

The effects of long-term exposure to magnetic fields on stress and emotional behaviour in mice were examined by Kitaoka et al. (2014). Groups of four week old, male ICR mice were exposed using a cylindrical coil apparatus to a 50 Hz magnetic field at 1.5 mT (corrected by authors in Erratum) for 5 h/day for 25 days. Four behavioural tests were conducted during the exposure period, and no differences were found in the open field or EPM, but a significantly increased latency to enter the light compartment during a light-dark test was found, with an increased immobility time and decreased distance moved in the forced swim test. Plasma corticosterone levels (obtained after 1 week of exposure)

were increased in exposed mice but plasma ATCH and the expression of genes related to stress response did not change. It was concluded by the authors that exposure had induced anxiety and depression-like behaviour due to increased corticosterone secretion that was independent of the HPA axis. The results are interesting, but it is not clear when the behavioural tests were carried out, and the animals appear to have been tested after varying lengths of exposure to the magnetic field, confounding the results.

Salunke et al. (2013) also investigated the effects of long-term exposure to magnetic fields on anxiety using three behavioural tests. Groups of adult Swiss mice were exposed to a 50 Hz magnetic field at 1 mT for 8 h/day using a pair of Helmholtz coils, and anxiety was measured after 7, 30, 60 or 90 days of exposure; each animal was tested only once. It was found using an EPM, an open field arena and a social interaction test that exposure periods for 30 days or more significantly increased anxiety without affecting locomotion. There was a significant elevation of both GABA and glutamate levels in the hippocampus and hypothalamus of mice exposed for 120 days. In order to investigate the role of GABA and glutamate further, other mice that had been exposed (or sham-exposed) for 120 days were injected at sub-effective doses with a GABA receptor agonist or antagonist (muscimol or bicuculline) or a glutamate receptor agonist or antagonist (NMDA or MK-801) and anxiety levels measured in the three tasks. It was found that the glutamate analogs modulated field-induced anxiety (with NMDA increasing and MK-801 decreasing measures of anxiety) and behaviour in all tasks was not affected by the GABA analogs. Overall, the authors concluded that magnetic fields can produce an anxiogenic effect in rodents, and that this might be related to activation of NMDA glutamate receptors.

Janać et al. (2012) reported that exposure to 50 Hz magnetic fields for 7 days resulted in age-related changes in the motor behaviour of Mongolian gerbils. Groups of animals were housed between 20 and 40 cm from an electromagnet producing a gradient 50 Hz field, such that the field in the centre of the cages was 0.1, 0.25 or 0.5 mT. The behaviour of the gerbils was monitored in an open field arena (away from the electromagnet) for 60 min using a video tracking and analysis system at four intervals during the exposure period, and 3 days after exposure. Treatment group sizes were relatively modest, consisting of 5 - 7 animals. The data were analysed in two 30 min periods (although the reasons for this decision were not explicitly given). For 3 month old gerbils, significant increases in distances moved, average speed and stereotypic movements of the head, and significant decreases in immobility time were reported after 1 day of exposure, and only in the first 30 min of each assessment period, but no dose response was apparent; no consistent effects were observed 3 days after exposure. For 10 month old gerbils, the changes in behaviour were less consistent and significant changes were mostly seen again after 1 day of exposure and only in the first 30 min of each assessment period, although exposure at 0.5 mT provided some evidence of causing effects throughout exposure; significant changes in all behaviours were observed 3 days after exposure. The results were attributed to differential effects on neurotransmitters in the brain structures that control exploratory activity in young and adult gerbils. Previously this group had reported that continuous exposure of Wistar rats for 7 days to a 0.5 mT field affected serotonergic transmission in the prefrontal cortex (the affinity of serotonin 5-HT_{2A} receptors was decreased and their density was increased) although no effects were seen on dopamine D_1 and D_2 receptors in the striatum (Janać et al., 2009).

Prato et al. (2013) examined the sensitivity of adult male CD-1 mice to 30 Hz magnetic fields. Analgesia (anti-nociception) could be gradually induced in mice by placing them for 1 h per day for 5 days in mu-metal cages that attenuated the ambient magnetic fields more than 50-fold. Each cage also contained 4 coils in a Merritt-like configuration to produce a magnetic field. It was found that exposure of animals in the cages to 30 Hz fields at 65 or 33 nT reduced the magnitude of the analgesic response induced immediately after exposure. Analgesia was measured using a hot plate test to quantify latency of foot withdrawal to a thermal stimulus. No effects were seen using a stainless steel cage, suggesting the non-involvement of electric fields. In addition, exposure to a

horizontal static field (0 Hz) at 44 μT reduced analgesia by approximately half the value produced by time–varying (30 Hz) fields.

Compared to the situation with magnetic fields, very few behavioural studies have been conducted using electric fields. Hawakawa et al. (2007) studied the effects 50 Hz electric fields at 16 kV/m (rms, unperturbed) on place aversion conditioning in Wistar rats. Whereas unexposed animals were conditioned to avoid the white half of a shuttle box apparatus over 6 daily trials using light as the unconditioned stimulus, this aversion response was not shown by animals exposed to the electric field, and they still preferred to spend more time in the white half of the apparatus. However, the exposed animals initially had a greater preference for the white compartment than the sham-exposed animals, and the effects of noise and vibration from the exposure system were not considered. The field used was also above the perception threshold range of rats (2-10 kV/m).

Mechanistic studies

Studies have continued to investigate potential interaction mechanisms and a number of possible mechanisms have been suggested, including effects on neural plasticity, and changes in oxidative stress.

Akdag et al. (2010) investigated the effects of long-term exposure on apoptosis and oxidative stress in rat brain tissues. Male Sprague-Dawley rats were exposed to 50 Hz fields at either 100 or 500 μT for 2 h every day for 7 months. As an indication of cell death at the end of exposure, active caspase-3 expression was analysed subjectively by two investigators using immunohistochemistry: no field-dependent effects were seen. A number of markers for oxidative stress were examined, and apart from a significant decrease in catalase levels at both intensities, significant changes in MDA levels, total antioxidant capacity and total oxidative stress were only seen in the group exposed to the higher field intensity. There was no effect on myeloperoxidise levels. Taken together, it was concluded that long-term exposure had increased oxidative stress through an increase in radical oxygen species production.

The effects of acute exposure to 60 Hz fields on the antioxidant systems in rat brain were investigated by Martinez-Samano et al. (2012). Immobilised or freely moving male Wistar rats were exposed for 2 h at 2.4 mT using a Helmholtz coil system. Compared to unexposed and unrestrained controls, exposure of freely moving animals produced lower values for SOD and for catalase activity, whereas restraint plus exposure also produced significant changes in glutathione content and NO levels.

Selaković et al. (2013) investigated the effects of exposure to a gradient 50 Hz magnetic field on oxidative stress in brain tissues of 3 and 10-month old Mongolian gerbils. Small groups of animals were placed in their home cages at set distances from an electromagnet to produce average fields of 0.1, 0.25 or 0.5 mT in the cages. Animals were continuously exposed for 7 days, and oxidative stress was measured immediately or 3 days after cessation of exposure. All exposures produced a field-dependent increase in NO levels, superoxide production, SOD activity and MDA levels in all brain areas examined, in both 3 and 10 month old gerbils. These changes persisted in 10-month-old gerbils measured 3 days after exposure, although they were reduced or absent in 3 month old animals at that time point. It was concluded that magnetic field exposure had significantly increased production of free-radical species. However, treatment group sizes are fairly modest, and as mentioned above (Janać et al. 2012), since the field strength depends on the distance from the magnet, animals in neighbouring cages could have been exposed to a range of overlapping field strengths. Akdag et al. (2013) reported intensity-dependent changes on oxidative stress in brain tissues of adult, male Wistar rats. Animals were exposed to 50 Hz fields at 100 or 500 μT for 2 h/day for 10 months using a Helmholtz coil apparatus within a Faraday cage. At the end of exposure, β-amyloid protein levels in homogenized brain samples remained unchanged by exposure at both field strengths, but MDA and protein carbonyl levels both increased significantly with exposures at 500 μT producing significantly larger effects.

Manikonda et al. (2014) also reported effects on oxidative stress in rat tissues following long-term exposure to power frequency magnetic fields. Wistar rats were housed individually in cages covered by a stainless steel mesh and exposed to 50 Hz fields at 50 or 100 μT for 90 days continuously using a system off coils wound on wooden bobbins; sham exposure was performed using a similar set of non-energised wooden bobbins. Significant effects were seen that depended on the region of the brain and field intensity: MDA levels and glutathione peroxidase activity were increased in the hippocampus and cerebellum at both 50 μT and 100 μT, but only at 100 μT in the cortex; superoxide dismutase activity was increased in all brain areas at 100 μT only; and the level of reduced glutathione and oxidized glutathione (GSH/GSSG ratio) was decreased in all brain areas at 100 μT and decreased in the cerebellum only at 50 μT. Exposure had no significant effect on body weight. Overall, the authors concluded that long-term exposure to the magnetic field had induced oxidative stress in the rat brain.

In a brief communication, Chu et al. (2011) also reported that acute exposure to 60 Hz fields affected lipid peroxidation and antioxidant defence mechanisms. Exposure at 2.3 mT for 3 h was found to significantly increase MDA and production of hydroxyl radicals in the cerebellum of male Balb/C mice, as well as increase SOD and decrease ascorbic acid levels. There was no significant change in glutathione or GPx. In what otherwise appears to be a well-conducted study, no information was provided on the exposure system nor on metrology or dosimetry, although other studies from the same group suggest they may have used a Helmholtz coil system. But without this information it is impossible to assess any contribution to the observed effect from potential stress associated with the generation of the field.

Frilot et al. (2009, 2011) reported increases in localised glucose utilization in the brain following exposure to magnetic fields. Female Sprague-Dawley rats were exposed to either a continuous or intermittent (2 s on, 2 s off) 60 Hz field at 250 μT for 45 min. The animals were either restrained during exposure (to ensure the angle between the field and the body axis of the animals was kept constant), or they were free to move. Noise and vibration produced by the exposure system were minimised, and it was reported that animals did not respond behaviourally to the presence of the field. Neuronal activity was measured by positron emission tomography using fluorodeoxyglucose (FDG). Intermittent field exposure was associated with significantly increased FDG uptake in the mid-sagittal region of the hindbrain (possibly in the medulla or cerebellum due to uncertainties in localization) only in animals held in a fixed orientation to the field; continuous exposure produced far smaller changes in uptake. It was proposed that the induced electric fields had exerted a force on oligosaccharide side chains bound to ion-channel gates in a membrane, so opening those gates and increasing neuronal activity. It was reasoned that randomizing the direction of the field would reduce FDG uptake by mitigating the cumulative effect of the field on the ion channel gates. Frilot et al. (2009) reported that exposure to an intermittent magnetic field (2s on, 5s off) produced evoked potentials with a latency of about 500 ms of field onset, when analysed by a novel technique called recurrence analysis (which is capable of detecting nonlinear relationships) although not when analysed by traditional time-averaging techniques.

Reyes-Guerrero et al. (2010) reported that exposure of female Wistar rats to a 60 Hz magnetic field caused biphasic changes in estrogen receptor-beta (ERβ) gene expression that depended on the phase of the estrous cycle: exposure significantly decreased expression during oestrus and significantly increased expression during diestrous. No changes were seen in proestrous or metestrous, or in males and ovariectomised rats; nor in ERα expression in any treatment group. Unrestrained rats were exposed at 1 mT using a Helmholtz coil system for 2 h/day for 9 days, and mRNA levels in the olfactory bulb were analysed using RT-PCR with a GAPDH control.

Using Western blots, Strasák et al. (2009) investigated the effects of a 50 Hz magnetic field on the protein level of c-Jun and c-Fos in the brains of young ICR mice. The level of c-Fos was found to be unaffected by exposure at 2 mT for 4 days in both male and female mice, but c–Jun was significantly decreased in the olfactory lobes and the right

hemisphere in both sexes. However, the statistical analysis is not presented, and the numbers of animals in each treatment group are not given: group sizes could be fairly modest because they were taken from just one litter.

The effects of magnetic fields on NO signalling in the brain have been studied by Cho et al. (2012). Male Sprague-Dawley rats were exposed to a 60 Hz field at 2 mT for 5 days using a pair of Helmholtz coils. It was found that NO levels in the cortex, hippocampus and striatum were significantly increased following exposure, which correlated with an increase in the numbers of neurons expressing neuronal NO synthase activity. Conventional and electron microscopy did not reveal any changes in the morphology or number of neurons, suggesting the increased production of NO had not induced pathology. Nevertheless, given the emerging importance of NO as a signalling molecule in the brain, the finding that magnetic fields may increase NO production could have important consequences for health and well-being.

The expression on hsp70 mRNA in the brain tissues of CD1 mice exposed to 50 magnetic fields was investigated by Villarini et al. (2013). Animals were exposed to 0.1, 0.2, 1 or 2 mT for 15h/day for 7 days using a pair of rectangular coils in a Helmholtz-like configuration. No consistent effects on hsp70 expression were seen using real-time RT-PCR (expression was only significantly elevated in the hippocampus at 0.1 mT) and no effect on hsp70 protein was seen using Western blot analysis. Comet assays indicated that exposure at 1 mT temporarily increased the amount of DNA strand breaks in the hippocampus and cerebellum, while exposure at 2 mT also temporarily increased DNA damage in the cortex-striatum. In all cases, any field-induced damage had been repaired after 24 h. The capillaries in the circumventricular organs (CVOs) that include the pineal gland and the area postrema exhibit increased vascular permeability due to their cellular morphology. The effect of magnetic fields on permeability in the CVOs was investigated by Gutiérrez-Mercado et al. (2013). Male Wistar rats were exposed to 120 Hz magnetic fields at 0.66 mT using a pair of Helmholtz coils for 2 h/day for 7 days. Animals remained in their home cages within the coils at all times, and were habituated to the system for 3 days before exposure started. Animals were injected with tracer (colloidal carbon) immediately after the final exposure, and their brains examined using light and electron microscopy. Compared to sham-exposed animals that were housed within non-energised coils for 10 days, it was found that exposure significantly increased permeability in the CVOs and their vascular area was increased. The permeability of the blood-brain barrier in the hippocampus and cortex was also increased by exposure.

Balassa et al. (2013) reported that prenatal and early exposure to magnetic fields caused long-lasting changes in neuronal functions and plasticity in the brains of Wistar rats. Using a Helmholtz coil apparatus, foetal animals were exposed to 50 Hz fields at 0.5 mT for 7 days continuously starting in the second week of gestation or young males were exposed to 3 mT for 7 days starting on postnatal day 3 were made. Electrophysiological recordings were made using brain slice preparations of the cortex and hippocampus at 2 months of age. It was found that foetal (not postnatal) exposures significantly increased basic synaptic excitability in the hippocampus and cortex (measured by amplitude of evoked field potentials). Foetal exposure also affected synaptic plasticity (exposure significantly inhibited paired-pulse depression, and decreased long-term potentiation) in the cortex but not in the hippocampus: no effects were seen following postnatal exposure. Overall, it was suggested that exposure had caused a long-lasting increase in the excitatory state of the neurons in the cortex and hippocampus.

Fournier et al. (2012) also reported long-lasting changes in adult hippocampal microstructure and function in rats following intermittent (10 s on, 40 s off) exposure to low intensity, complex magnetic fields throughout gestation. Exposure was achieved using a pair of Helmholtz coils at end of the home cage, with distance from the coils used to produce different intensities. It was found that exposure at 30-50 nT resulted in impairments in the learning of a fear-conditioning paradigm along with reductions in overall hippocampal size with subtle changes in morphology: exposure to weaker or stronger fields did not produce these effects. The result is biologically intriguing, but the

complex and unusual nature of the field, plus the narrow intensity window for the effects may limit the relevance of these studies.

Xiong et al. (2013) found that the morphology of cortical neurons was affected by long-term exposure to magnetic fields. Adult rats were exposed to a 50 Hz magnetic field at 0.5 mT for 4h/day for either 14 or 28 days, and cell morphology was assessed in Golgi-stained sections of the medial endorhinal cortex using image analysis techniques. It was found that exposure at both time periods induced a decrease in spine density of the dendrites of stellate and pyramidal neurons. Exposure also caused changes in spine shape that depended on the specific type of dendritic spine and the length of exposure. Overall the author suggested that exposure-induced changes in dendritic spines might provide some explanation for the field-related changes that have been observed in cognitive function.

Very few recent studies have investigated the effects of electric fields, possibly due to the difficulties of avoiding perceptual effects of the field by the animals. Akpinar et al. (2012) found that repeated, acute exposure of female rats to 50 Hz electric fields resulted in prolongation of the components of the visual evoked potential, as well as in increases in total oxidant status and lipid peroxidation in the brain and retina. Electric fields were generated using a pair of copper plates connected to a transformer, and groups of 5 animals were exposed to fields of 12 or 18 kV/m (unperturbed values) for 1 h per day for 14 days. Effects were generally larger at the higher field intensity. The possibility of perceptual effects cannot be ruled out using these field values, however. The authors suggested that the plastic cages holding the animals would have provided some shielding, although the rats themselves and their behaviour could lead to local enhancements of the field.

Potential therapeutic applications

Some studies suggest that low intensity magnetic fields may provide novel therapeutic benefits, possibly by increasing neurogenesis and so deliver protection against neurodegenerative disease and by restoring function following injury to nervous tissues.

Shin et al. (2011) reported that repeated exposure of C57BL/6 mice to 60 Hz magnetic fields at 0.3 or 2.4 mT 1 h/day for 14 days resulted in intensity-dependent increases in locomotory activity as measured using an automatic video tracking system. This hyperactivity was largest immediately after the last exposure and diminished with time thereafter it remained significantly elevated 1 day after exposure using 0.3 mT, and 1 week after exposure using 2.4 mT; activity was not elevated at 3 months after either exposure. Numbers of cells showing fos-related antigen (FRA) expression in the striatum and nucleus accumbens were significantly increased 2 h after the last exposure, and these remained significantly elevated for 1 year. Exposure at 2.4 mT produced larger effects than 0.3 mT. Injection of the mice with SCH 233390, a dopaminergic D1 receptor antagonist, but not with sulpiride, a D2 receptor antagonist, 30 min before each exposure resulted in an attenuation of the effects on activity and FRA-positive cells, suggesting these effects were mediated by stimulation of D1 receptors. The authors acknowledged that the role and physiological significance of the long-term changes observed require further clarification, but they suggested that magnetic fields could be of benefit in improving Parkinson’s symptoms.

Grassi and co-workers have investigated the effects of magnetic fields on neurogenesis in hippocampus of mice of juvenile and adult mice. Cuccurazzu et al. (2010) investigated the effects of 50 Hz magnetic fields on neurogenesis in the hippocampus of adult mice. Adult C57BL/6 mice were exposed at 1 mT using a solenoid for 1-7 h/day for 4 or 7 days. It was found that exposure significantly increased numbers of immature neurons in the dentate gyrus, with a trend for longer daily exposures to have larger effects. Exposure also significantly increased the expression of three genes involved in neuronal commitment and differentiation, *Hes1*, *Mash1* and *NeuroD,* and genes encoding a voltage-gated Ca channel (α_{1C} subunits of $Ca_v1.2$). Electrophysiological recordings indicated that the newly generated neurons became functionally integrated in to the

hippocampus, resulting in enhanced synaptic plasticity. Overall, the authors suggested that magnetic fields may have a role to play as a treatment for neurodegenerative disease. A similar suggestion was reached by Leone et al. (2014). This study exposed young adult C57BL/6 mice to 50 Hz magnetic fields at 1 mT for 3.5 h/day for 12 days, A solenoid was used to produce the field, and the behaviour of the animals was assessed using a water maze and a novel object recognition task 30 days after exposure finished. It was found that exposure significantly improved performance in both tasks compared to controls: animals in the water maze showed decreased latencies to find the escape platform and spent more time in the target quadrant during the probe trial; and animals in the object recognition task spent more time exploring the novel object and less time exploring the familiar object. Other animals were injected i.p. with BrdU (100 mg/kg) immediately before each exposure, and the numbers of proliferating and immature neurons in the dentate gyrus of the hippocampus were assessed *ex vivo* 24 h or 40 days after exposure using immunohistochemistry. Exposure was found to have significantly increased neuronal numbers at both time points. Podda et al. (2014) also performed a similar experiment investigating neurogenesis in juvenile mice. In this study, mice were first injected with BrdU (100 mg/kg) for 3 consecutive days, then 9 days later were exposed to the magnetic field for 3.5 hours/per day for 6 days; mice were killed 3 or 9 days after exposure. It was found that exposure significantly increased cell proliferation, significantly increased protein expression of NeuN a neural marker suggesting an increase in neuronal maturity, and significantly decreased apoptosis. The performance of mice in a recognition memory task (tested on day 4, 5 and 6 of exposure) and a water maze task (on day 9 to 13 after the exposure) was also significantly improved by exposure. The authors concluded that magnetic fields had increased the survival of newly developed hippocampal cells.

Tasset et al. (2012) reported a protective effect of magnetic fields in a rat model of Huntingdon's disease in which animals were injected with 3-nitroprorionic acid (3NP) to induce neurological and behaviour changes. Male Wistar rats were exposed to a 60 Hz magnetic field at 0.7 mT for 2 h in the morning and 2 h in the afternoon for 21 days. The animals were held immobile in plastic cylinders, and their heads placed between a pair of horizontal Helmholtz coils. Animals were injected i.p. with 3NP (20 mg/kg) on 4 consecutive days immediately before exposure to the magnetic field. 3NP alone caused a significant decrease in dopamine levels (measured in a homogenised half brain), and decreased locomotion in an open field test and increased immobility time in a forced swim test. These effects were reduced in animals also given exposure to the magnetic field, although dopamine levels were lower compared to controls and immobility time was very much reduced. Exposure to magnetic fields alone had no significant effects. Compared to controls, levels of brain- and glial-derived neurotrophic factors were significantly increased in all treatment groups, including animals just exposed to the magnetic field. Histological examination of the brains revealed that 3NP had increased neurodegeneration and neuronal cell loss in the striatum that were largely reversed by the magnetic field, as were 3NP-induced effects on caspase-3 and lactate dehydrogenase activity. Similarly, exposure to the magnetic field reversed the 3NP-induced changes in glutathione, lipid-peroxidation products and in 8-hydroxy-2'-deoxyguanosine levels. In addition, Tasset et al. (2013) found that exposure to the magnetic field modulated expression of NF-E2-related factor 2 (Nrf2) a transcription factor that is responsible for regulating a battery of antioxidant and cellular protective genes in response to oxidative stress. Using Western blot analysis, it was found that 3NP treatment alone decreased Nrf2 protein levels in the cytoplasm and nucleus of brain cells, and Nrf2 levels in 3NP-treated rats were significantly increased (although they were still reduced compared to control values). This suggests a possible mechanism whereby magnetic fields were able to reverse the neurotoxic effects induced by 3NP treatment. In order to assess whether magnetic fields increased obsessive compulsive disorder, Salunke et al. (2014) investigated the effects of magnetic field on marble-burying behaviour in adult, male Swiss mice. Animals were exposed to 50 Hz fields at 1 mT for 8 h/day for up to 120 days using Helmholtz coils. All exposures increased the numbers of marbles buried in a 30 min period in an arena containing 20 marbles, with longer exposures producing larger effects.

Locomotion was not affected. Exposure for 120 days had no effect on dopamine or serotonin levels in the cortex, hippocampus or hypothalamus, but NO levels were significantly increased. Marble-burying behaviour was increased by daily i.p. injection with NO precursors and nitric oxide synthase (NOS) inhibitors attenuated the response. Thus the increase in marble-burying behaviour was attributed to increased NO levels, particularly in the cortex. In order to help to develop prevention strategies against potential field-induced neurotoxicity, Celik et al. (2013) investigated the effects of magnetic fields exposure on manganese (Mn) accumulation in Sprague-Dawley rats. Animals were exposed to 50 Hz fields at 1.5 mT for 4 h/day. 5 days/week over 45 days using a Helmholtz coil apparatus within a Faraday cage. Rats also received Mn solution (3.75, 15 or 60 mg/kg body weight) every other day by gavage. Mn levels were determined immediately after exposure in brain, kidney and liver tissues by spectrometry. It was found that Mn ingestion alone increased Mn levels in the brain and other tissues, and these levels were further significantly increased with magnetic field exposure. However, the numbers of animals used was small, with five animals in each treatment group.

Das et al. (2013) reported that intermittent exposure to low intensity magnetic fields can provide a significant recovery of function caused by spinal cord hemisection. One day after surgery to cut the left hand side of the spinal cord at T13, rats were exposed to a 50 Hz magnetic field at 17.96 µT for 2h/day for 6 weeks. Using a battery of tests to assess behaviour, nociception and neurophysiology, it was found that exposure caused a significantly improved pattern of locomotion in an open field throughout the six weeks of the study, along with increased recovery from pain, and faster restoration of bladder function. However, there were no sham-surgery or sham-exposure groups. A further study (Kumar et al. 2013) investigated the effects that magnetic field exposure (at 17.96 µT for 2 h/day for 8 weeks) had on pain behaviour in rats following complete spinal cord transection at T11. It was found that locomotion in an open field was significantly increased in exposed animals, but remained well below values in sham animals (whose treatment was not defined). At the end of the exposure period, behavioural pain ratings were subjectively scored for 60 min following injection of algogen (5% formalin solution) into the left dorsal hindpaw. Transection alone gave significantly reduced pain ratings compared to sham controls, whereas transection with exposure gave values between the sham and transected alone groups. In addition, magnetic field exposure reversed an observed decrease in 5-HT in the cortex, and also reversed the increase in norepinephrine and GABA in the brain stem seen with transection alone. These are interesting results but only a single magnetic field intensity was used in both experiments, and why the particular value was chosen was not explained. The lack of an appropriate sham control group in one experiment is also of concern.

Conclusions on *in vivo* studies

Animal studies have continued to investigate the effect of magnetic fields on neurobiology using various models and exposure conditions. While generally these studies are of good quality, many have used single field strengths, sometimes well in excess of exposure guideline values. Also, the possibilities of noise or vibration produced by Helmholtz coil-based exposure systems have not always been addressed adequately, and solenoid-based systems, where an animal's behaviour in the cage will affect its exposure, are not ideal. Largely consistent with earlier results, recent studies have reported that exposure to ELF magnetic fields has no effect on activity or locomotion. There is some evidence from animal studies that exposure to ELF MF may affect the performance of spatial memory tasks (both deficits and improvements have been reported) and engender subtle increases in behavioural anxiety and stress. Other studies have investigated potential molecular and cellular mechanisms, and despite a number of studies continue to report candidate mechanisms, particularly regarding effects on reactive oxygen species, none has been firmly identified that operates at exposure levels found in the everyday environment.. Several studies have tried to reveal subtle effects of magnetic fields through their interactions with drugs or other interventions but these have not produced any compelling evidence of field-induced changes on nervous system

function. Some, studies have suggested that magnetic fields may offer potential therapy against neurodegenerative diseases, although these results require confirmation and clarification. Finally, no additional insights regarding the effects of electric fields are possible, due to the almost complete absence of new data.

3.8.2.4. *In vitro* studies

What was already known on this subject?

It was stated in the previous SCENIHR Opinion that "Very few recent *in vitro* studies have investigated effects from ELF fields on diseases other than cancer and those available have very little relevance for understanding any disease connection. There is a need for hypothesis-based in vitro studies to examine specific diseases".

What has been achieved since then?

There are few suitable *in vitro* model systems for nervous system effects and disorders such as NDD. Therefore, it is appropriate to evaluate *in vitro* studies that are using nerve cells or glial cells, in combination with relevant experimental end-points (cell survival and death; cell differentiation, radical homeostasis, expression of inflammatory markers; synaptic transmission, functionality of the blood-brain barrier).

Previously it was noted that a few studies have focused on differentiation into the neuronal phenotype of undifferentiated or lowly differentiated precursors of nerve cells. Since the last Opinion, a study by Saito et al. (2009) used P19 embryonal carcinoma cells. The cells were induced to differentiate if exposed to 10 mT (50 Hz; 21 days exposure), but not at a lower flux density (1 mT). The expression of MAP2 and spike frequencies increased, whereas the glial marker GFAP decreased. In another study, primary cultures of newborn mouse cortical neuronal stem cells were stimulated to increase their differentiation rate after continuous 50 Hz MF exposure (1.0 mT) for up to twelve days (Piacentini et al. 2008). The differentiation was seen as enhanced expression of neuronal markers and enhanced Ca_v-channel expression and activity.

In a study by di Loreto et al. (2009) primary cultures of embryonal rat cortical neurons were used. The cells were exposed for seven days to a 50 Hz MF (0.1 or 1.0 mT). The higher exposure level had stronger effects, if effects occurred at all. The 1.0 mT exposure caused increased vitality and decreased apoptosis, possibly due to the enhancement of neurotrophic support. This seems to be independent of radical homeostasis disturbances, since redox status, MDA levels, and enzymatic activities were unaffected by exposure. The study did not include any positive control(s).

Synaptosomes (isolated synaptic terminals from neurons) from one adult male sheep was used by Afrasiabi et al. (2014) in a study where they investigated sine wave ELF MF effects on acetylcholine esterase (AChE) activity. The authors generated the MF in a set of Helmholtz coils in the frequency range 50-230 Hz at flux densities from 0.3-1.7 mT. Exposure duration was from 15-120 min. The results indicated a biphasic response to the ELF MF. Flux densities ranging from 0.8-1.2 mT at frequencies of 150-230 Hz (all investigated exposure durations) as well as 0.3-0.6 mT at 50-90 Hz increased AChE activity. In contrast, exposure at 1.2-1.7 mT at frequencies ranging from 50-90 Hz decreased enzyme activity. Obviously, the study needs to be replicated before the potentially interesting findings can be used for any assessment of health effects. Furthermore, there is no information regarding background EMF and blinding procedures in the article.

The role of voltage-gated T-type Ca^{2+}-channels in responses to ELF MF was investigated by Cui et al. (2013). The authors transfected T-type channels (Cav3.1, Cav3.2, Cav3.3) and measured the electrophysiological responses to sine wave 50 Hz MF (0.2 mT; 0.5-3 h exposure duration). The exposure caused inhibition of preferentially the Cav3.2 channel type, which also was seen in experiments where native channels (in mouse primary cultures of mouse cortical neurons) were investigated. By using various types of receptor antagonists, the authors could show the probable involvement of increased arachidonic

acid and subsequent increased leukotriene E4 levels in the MF response. The study seems to have been performed without proper sham conditions.

Embryonic neural stem cells have the potential to differentiate to mature neurons upon proper stimulation. It has been suggested that MF exposure can influence neuronal differentiation. This is the topic in the study by Ma et al. (2014) who obtained embryonic neural stem cells from the telencephala of embryonic day 13.5 BALB/c mice. The cell cultures were exposed to 50 Hz sinusoidal MF (either 0.5, 1 or 2 mT for 3 days; or 2 mT for 1, 2 or 3 days intermittently (5 min on/10 min off); double-blind conditions; sham and exposure randomly selected by computer). Cells were cultured under conditions that either promoted proliferation or differentiation, and a number of biological end-points were studied (cell viability, DNA synthesis, neurosphere diameter, cell cycle progression, specific protein and mRNA expression). In short, exposures had no effect on any of the end-points, with exception of some of the gene expression profiles during intermittent exposure. Thus, some genes that influence early stages of neural differentiation were up-regulated, although this had no effect on the phenotype of the cells. This nevertheless leaves the possibility open that intermittent exposures can have other effects than continuous exposure.

Differentiation to nerve cells from stem cells was also investigated in an interesting study from Seong et al. (2014). Both human bone marrow-mesenchymal stem cells and mouse embryonic stem cells were possible to induce to neuronal differentiation by means of a 50 Hz 1 mT MF exposure (8 and 6 days respectively). Neuronal molecular markers as well as morphology and electrophysiological properties supported that MF exposure led to neuronal differentiation. The authors then ambitiously undertook a transcriptome analysis, comparing the transcriptional profiles of MF-induced neuronal cells with those of sham-treated cells. Data showed that 57 genes expressed significant changes ($\geq$1.5 fold) due to exposure. Most of these genes were transcription factors, and in many cases associated to expression of the Egr1 gene. Elegantly, the authors then showed that Egr1 is necessary (by gene knockdown, but not sufficient (overexpression) for neuronal differentiation. Further studies in the same direction have been published by Bai et al. (2013), Cho et al. (2012), and Kim et al. (2013). All studies employed the same cell culture model and could demonstrate induction of neuronal differentiation (as determined by presence of various neuronal markers), appearance of neurons specific cellular structures and also electrophysiological properties.

Discussion on *in vitro* studies

The *in vitro* studies are mostly acute or short-term (with exposures ranging from minutes to a few days) and also limited by the fact that they almost always only include one cell type, primary cultures of neuronal precursors or established cell lines. The studies do not allow any conclusions regarding a possible effect of ELF MF exposure on, for example, development of neurodegenerative diseases, but offer some results that are interesting and possibly worthwhile following up, including the noted positive effects on differentiation. Especially the possible effects of ELF MF exposure on embryonic neuronal stem cells offer interesting possibilities for future studies. Besides these mentioned studies, there are no *in vitro* findings documenting effects on disease markers or transmitter systems.

Conclusions on *in vitro* studies

Like in the previous Opinion, the few available *in vitro* studies do not provide any support for drawing conclusions on the possible effects of ELF on the nervous system and neurobehavioral disorders.

3.8.2.5. Conclusions on nervous system effects and neurobehavioral disorders

Only a few new epidemiological studies on neurodegenerative diseases have been published since the previous Opinion. They do not provide support for the previous conclusion that ELF magnetic field exposure could increase the risk for Alzheimer's disease or any other neurodegenerative diseases or dementia.

Regarding neurophysiological studies, due to methodological weaknesses, these studies are not useful for drawing meaningful conclusions. The same is true for the results concerning behavioural outcomes and cortical excitability.

Largely consistent with earlier results, recent *in vivo* studies have reported that exposure to magnetic fields has no effect on activity or locomotion, but may affect the performance of spatial memory tasks (both deficits and improvements have been reported) and engender subtle increases in behavioural anxiety and stress. There is some evidence that these effects may be greater with higher intensity fields and with longer durations of exposure, but the magnitude or direction of effect cannot be defined with accuracy. *In vivo* studies that have investigated potential molecular and cellular mechanisms have not identified any mechanism that operates at levels of exposure found in the everyday environment. Animal studies that have suggested that magnetic fields may offer potential therapy against neurodegenerative diseases require confirmation and clarification. No additional insights regarding the effects of electric fields are possible, due to the almost complete absence of new data.

As in the previous Opinion, the few available *in vitro* studies do not provide any support for drawing conclusions on the possible effects of ELF on the nervous system and neurobehavioral disorders.

3.8.3. Other effects of ELF fields exposure

3.8.3.1. Symptoms

What was already known on this subject?

As with RF exposure, exposure to ELF fields has been suggested to cause symptoms, with some people describing themselves as being particularly sensitive to ELF exposure. This reported sensitivity falls within the broad definition of IEI-EMF. The 2009 Opinion concluded that no consistent relationship had been demonstrated between ELF exposure and symptoms, neither in the general public nor in people with IEI-EMF.

What has been achieved since then?

Since the 2009 Opinion, ten experimental provocation studies have tested whether exposure to ELF affects symptoms, well-being or other subjective outcomes, or whether participants can discriminate between real and sham ELF exposure.

In a study described in detail in section 3.8.2.2, Legros et al. (2012) analysed the effect of an exposure to a 60 Hz 1.8 mT ELF MF for the duration of one hour as compared to sham in 73 healthy participants (46 males and 27 females, 28 ± 9 years) using a double blind, counterbalanced cross-over design with test sessions on separate days. The magnetic fields were generated by Helmholtz-like coils of 1.6 m diameter. Each test session lasted 105 min. Participants completed a field detection questionnaire after each exposure. No evidence was found that they were able to discriminate between the conditions.

McNamee et al. (2011) tested the effects of 60 Hz MF exposure at 200 µT for one hour in 10 healthy volunteers, using a double-blind, counterbalanced cross-over design. Physiological variables including heart rate (HR), heart rate variability (HRV), skin surface temperature and blood pressure were assessed. Participants were also asked to complete a questionnaire relating to field detection. The exposure was delivered using the same apparatus as in Legros et al. (2012). This study identified no significant effects on HR, HRV or skin surface temperature. No effects on blood pressure were observed either. Participants were unable to accurately detect the exposure condition.

McNamee et al. (2010) tested 58 volunteers (mean age 27 +/-8.5 years) in a double-blinded protocol involving MF exposure at 60 Hz, 1800 µT (1 hour of exposure, real or sham). Once again, exposure was delivered using the same apparatus as that in Legros et al. (2012) and participants were asked to complete a field detection questionnaire. Participants were unable to accurately detect the MF exposure condition.

Corbacio et al. (2011) investigated a possible effect of a 60 Hz 3mT exposure (one hour duration) on 15 outcome parameters from 10 psychometric tests (see section 3.8.2.2) in a sample of 99 participants (60 females and 39 males, 18-49 years) who were assigned randomly to one of three exposure conditions: sham/sham; sham/MF exposure, and MF exposure/sham (a parallel-group design). Participants completed a field detection questionnaire at the end of each exposure. No evidence was found that they could discriminate between the conditions.

Kim et al. (2012) assessed the effects of real or sham exposure to a 60 Hz magnetic field (12.5 μT) generated above the heads of 15 IEI-EMF participants and 16 control participants. Each participant received one real and one sham exposure under double-blind conditions. Out of the eight symptoms that were measured, the only significant effect was a presumably spurious increase in perceived warmth among control participants during the sham condition. There was no evidence that either group could discriminate between the conditions.

Kim et al. (2013) exposed 30 adults (mean age 27.9 plus or minus 5.9 years; 15 female) and 30 teenagers (mean age 14.8 plus or minus 1.4 years) in a double-blind, randomised experiment to a magnetic field (12.5μT at 60Hz, generated using coils positioned either side of the participant's head) or a sham condition. Conditions occurred on separate days and lasted for 32 minutes. During and after each condition, participants were asked about the presence of eight symptoms and whether they could perceive any exposure. No effects of exposure were observed on these outcomes.

McCarty et al. (2011) exposed a single participant with IEI-EMF to ten 100-second conditions involving a 60 Hz electric field (300 V/m around the head) and to ten sham conditions. The participant was asked to describe any symptoms that she experienced, which were subsequently coded as 'none,' 'mild' or 'more than mild.' In a second study, the same participant received sham, continuous or pulsed (10 Hz) field exposures (five of each, lasting 100 seconds) and was again asked to describe her symptoms. The authors reported that in the first study, the participant experienced more symptoms in the real condition than the sham condition. In the second study, she experienced more symptoms in the pulsed condition than the sham condition. However, it has subsequently been suggested that analysing the data according to whether symptoms were present or absent would have resulted in a different set of findings (Rubin et al., 2012; Marino et al., 2012). A third study involving this participant observed that she was unable to discriminate sham exposure from exposure to carrier frequencies of between 60 Hz to 500 kHz during a series of 300 two-second trials (McCarty et al. 2011). According to how the exposure was done, it is not possible to determine if transients at the on/off were present and thus part of the total exposure. Robertson et al. (2010) exposed 47 healthy participants under single-blind conditions to magnetic fields of either 100 μT (n=6), 200 μT (n=14), 1000 μT (n=10) or a sham condition (n=17) for 15 minutes. Exposures were preceded and followed by a functional MRI investigation. Reduced activation in the anterior cingulated and insula regions of the brain in response to a painful thermal stimulation of the hand was observed following the 1000 μT and 200 μT conditions, suggesting reduced processing of pain stimuli. Participants in the 1000 μT condition were also significantly more likely to believe they were genuinely being exposed than those in the sham condition.

Landgrebe et al. (2008) assessed the ability of 89 people with and 107 people without IEI-EMF to detect transcranial magnetic stimulation by using a series of sham exposures and real exposures of intensities ranging from 0% to 57% of the maximum output of their stimulator (1.8 T). Perception thresholds for the real magnetic pulses were comparable in the two groups.

Maestu et al. (2013) assessed the impact of very low-intensity transcranial magnetic stimulation in a double-blind randomised controlled trial. Twenty-eight patients with fibromyalgia were randomised to receive exposure delivered through a magnetic stimulator consisting of an EEG cap with 33 stimulation coils evenly distributed across the head. Each coil produced a magnetic field of approximately 43 nT at a distance of 1cm

and 0.9nT at a distance of 4cm. A low frequency (8Hz) square function was used. Exposure occurred for one session per week over the course of eight weeks. Twenty-six patients were randomly allocated to receive an equivalent sham exposure. Median pressure pain threshold across 18 points on each patient's body was measured as the primary outcome. The stimulation group experienced a significantly improved pain threshold compared to the sham group over the eight weeks of the study. Some secondary outcomes (ability to perform daily activities, sleep quality, perceived pain) were also improved.

Koteles et al. (2013) tested whether 29 people with and 41 people without IEI-EMF were able to detect the presence of a 50 Hz, 500 μT magnetic field applied over their right hand. The field was applied ten times per participant, in 60-second trials. Ten sham conditions were also applied. The control group was found to be no better than chance at detecting the exposure. The IEI-EMF group, however, were significantly better than chance. The ratio of hits to false alarms was 1.22 in the IEI-EMF group, while it was 1.14 in the control group. In addition, one member of the control was able to detect the magnetic field "almost perfectly" and replicated his performance in a second testing session. The researchers noted that additional testing of this participant is planned, and that replication of the study as a whole is warranted.

Finally, in a double-blind provocation study focusing on the neurophysiological and behavioural effects of exposure for one hour to a 60 Hz, 1,800 μT magnetic field, Legros et al. (2012) found that their 73 participants were unable to accurately assess whether the field was present or not.

Aside from these experimental provocation studies, several observational studies have also assessed the possible association between exposure to sources of ELF fields and symptoms or other subjective effects. Zamanian et al. (2010) compared the mental health of three groups of workers: those exposed to electromagnetic fields and noise during their work at a power station, power station workers exposed to noise only, and administrative staff from a telecommunications company exposed to neither noise nor electromagnetic fields. The authors noted worse mental health in the group exposed to EMF. However, no attempt was made to control for any differences in work patterns or culture between these groups. It is unlikely that the remarkably high prevalence of mental disorder (78.2%) and social dysfunction (94.5%) identified within the noise and EMF group could be due solely to the effects of EMF.

Monazzam et al. (2014) assessed subjective sleep quality and levels of distress in 40 employees of a petrochemical complex. Participants were categorised on whether they had higher or lower occupational exposures to EMF (based on the location of the jobs: high voltage substations versus the control and engineering rooms). Those categorised as higher exposure were significantly more likely to suffer from poor sleep and psychological distress. However, the authors also measured EMF throughout the workplaces. Although details are scarce in the paper, they reported finding no significant relationship between exposure level and either outcome.

Korpinen and Paakkonen observed significant associations between use of a desktop computer and psychological symptoms in their sample of 6121 Finns (Korpinen & Paakkonen, 2009; see Section 3.6.3 for details). However, no attempt was made to control for other, non-EMF related differences between users and non-users of desktop computers. A subsequent analysis of this dataset demonstrated that, amongst respondents who experienced neck pain, the occurrence of various other musculoskeletal symptoms was associated with the use of desktop or laptop computers, presumably as a result of poor ergonomics (Korpinen, Paakkonen and Gobba, 2013).

Milde-Busch et al. (2010, see Section 3.6.3) observed some associations between use of electronic devices and headaches among their sample or 1025 adolescents, but cautioned that the inconsistency in their findings made it unlikely that these findings were valid.

Kucer et al. (2014) assessed the association between self-reported computer use and symptoms in a sample of 350 people from Turkey, finding associations between higher

usage and headache, vertigo/dizziness and tension-anxiety, but without attempting to control for any potential confounding variables.3.14.5

Finally, Baliatsas et al. (2011) assessed whether the distance from a powerline to a participant's house was associated with reports of symptoms in their sample of 3611 residents of the Netherlands. Although perceived proximity was associated with symptom reports, objective proximity was not.

Conclusions on symptoms

The studies published since the 2009 Opinion show discordant results. However, observational studies suffered from weaknesses and do not provide convincing evidence of an effect of ELF exposure on symptoms in the general population and most experimental evidence also points to the absence of any causal effect.

3.8.3.2. Reproductive effects

The relationship of residential ELF-EMF exposure from powerlines to pregnancy outcomes was evaluated in two reports of a Canadian cohort study. The material consisted of more than 700,000 live births in Montréal and Québec City during 1990-2004 (Auger et al. 2011). Exposure assessment was based on distance between residence and nearest powerline. The end-points evaluated included preterm birth, low birth weight and small for gestational age. Information on mother's age, parity, marital and socioeconomic status, and ethnicity was also available. More than 12,000 births were classified in the highest exposure category (<50 m from the powerline). No increased risks were found for any of the outcomes (adjusted ORs 0.99-1.04, with upper confidence limit of less than 1.10). Some uncertainty was due to the fact that the address was available only at time of birth, and lack of information on powerline voltage, earlier reproductive outcomes or lifestyle factors such as smoking. A similar analysis was conducted for stillbirths in 1998-2007 (Auger et al. 2012). The material consisted of 2033 stillbirths, also including pregnancy terminations due to foetal anomalies (foetal death with weight ≥500g regardless of gestational age) and more than 500,000 live births (singletons only for both categories). Non-significantly elevated odds ratio was found for distance <25 m from powerline (OR 1.4, 95% CI 0.9-2.4), without a clear trend by distance or increase in the second highest exposure category. Besides the above mentioned limitations, the analysis also had a small number of exposed stillbirths (16 in the highest exposure category), which hinders precise risk estimation.

An Italian case-control study assessed the relation between power lines and congenital anomalies (Malagoli et al. 2012). The material covered 228 congenital anomalies during 1998-2006 in Reggio Emilia (including livebirths, stillbirths and induced abortions), with a similar number of pregnancies as controls (matched by calendar year, hospital and maternal age). Magnetic flux density was estimated from distance from residence (during the first trimester) and average load of the line. The number of exposed subjects was too small to meaningfully evaluate the risk. Only one case and five controls had exposure levels exceeding 0.1 μT (OR 0.2, 95% CI 0-2.0).

A two-year prospective study in China enrolled pregnant women at 8 weeks of gestation and women planning pregnancy during 2010-2012 (Wang et al. 2013). The study area chosen was two towns with high prevalence of ELF exposure from power lines. Spot measurements of 50 Hz magnetic field were performed with Narda EFA-300 devices at the front door and on the street in front of the house. The median levels were 0.1μT and the exposed group was defined as those with a magnetic field above the median.

In analyses of the front door measurements, 8% of the pregnant women in the low exposure group had a miscarriage, while the proportion in the high-exposure group was 12%. When the high-exposure group was subdivided, no gradient was found for the average front door measurements, but a higher risk of miscarriage was seen in women with the highest maximal values of measured fields on the street in front of the house. Similarly, for continuous field strength measurements a significant increase was found only for the maximal values on the street, but not for the average front door

measurements. The women with miscarriage were slightly younger and had more commonly lower education and history of previous pregnancy problems or depression. Adjustment for these factors did not substantially alter the results.

Details of recruitment and participation of the subjects were not given. No measurements were performed within the home of the subjects, which reduces the validity of the results. The finding that miscarriage was associated with magnetic field on the street, but not at the front door seems counterintuitive as front door should be a better indicator of the residential exposure. It was unclear why the maximal values and not the average for the street were used in the analysis and this may be a post hoc decision suggesting exploratory data analysis. Only a third of the subjects had exposure levels above the median, but this could be due to a peaked exposure distribution (kurtosis).

A large cohort study covered 140,000 live singleton births in 2004-2008 in Northwest England (de Vocht et al. 2014). Residential proximity to power lines, cables, substations, or towers was used as the exposure indicator and it was estimated based on postal codes. The outcomes included preterm birth, low birth weight and small for gestational age and were obtained from a perinatal survey database covering 21 maternity units. Information was also available on maternal age ethnicity and parity. Complete data on all variables was available only for 53% of the births and information maternal smoking only for a third of the included births.

An alternative analysis using propensity score matching showed a similar result, with a lower birth weight for residence <50 m though of smaller size and borderline significance, but the findings were not consistent internally as a higher birth weight was found for distance <100 m (de Vocht et al. 2014).

The frequencies of various outcomes ranged 6-9% (7800-12,000 cases). Less than 1% resided within 200 m of the closest ELF-EMF source and distance was associated with deprivation index. No statistically significant differences in risk were found for proximity to a source of 50m or less, 100m or less or 200m or less compared to the reference distances for any of the selected birth outcomes. The highest exposure group (<50 m distance) had less than 10 events for all outcomes in the main analysis, with odds ratios ranging 1.3-1.7. Higher, but non-significant odds ratios were reported in the subset of the data from the most recent years with maternal data available. An analysis using the mean birth weight of all babies, instead of the proportion of markedly reduced birth weight, a significantly lower birth weight was found for residence <50 m from the closest EMF source based on 89 children (difference 125 g).

The main analysis did not reveal any significant association, and it is unclear if the analysis of mean birth weight was pre-specified or carried out post hoc. The validity of the exposure classification based on postal codes rather than house coordinates is uncertain.

In conclusion, recent results do not show an effect of the ELF fields on the reproductive function in humans.

3.8.3.3. Effects of foetal exposure to ELF on children's health

Maternal ELF exposure during pregnancy was associated with the risk of asthma in the offspring by age 12 (Li et al. 2011). A cohort of pregnant women was enrolled during the first trimester of pregnancy and a 24-hour measurement was carried out. An interview on socio-demographic and lifestyle factors was also carried out. Information on diseases in the offspring was obtained from the database of the health insurance provider. Of the original 1063 women, 626 were included in the analysis and a fifth of their children were later diagnosed with asthma. Compared with the lowest exposure decile, 10% with the highest exposure had a statically significant more than three-fold risk of asthma. The risk was also higher relative to the majority of the women in the cohort (with exposure between 10th and 90th percentiles), but the difference was not significant. Yet, the risk of asthma was also significantly associated with the mean field strength as a continuous variable. Subgroup analyses showed the risk mainly among first-born children and the

risk was also higher among mothers with a history of asthma. The results appear surprising, but the study has strengths including prospective setting with a measured exposure and information on several potential confounding factors. To some extent the findings may reflect a reduced risk in the subset with the lowest exposure levels and more detailed information on exposure-effect relation would be useful.

An analysis of childhood obesity from the same study suggested an association with residential EMF exposure (Li et al. 2012). The material was the same as above, with 733 mother-child pairs available for the analysis. Exposure classification was based on dividing the subjects into three groups based on the 90th percentile of the magnetic field level of the 24-hour measurement (cut-points at 0.15 and 0.25 µT). Obesity was defined as weight exceeding the 97.5th percentile of the CDC growth charts. However, as many as 12.9% of the children were considered to be obese. On average 11 (median 33) weight measurements were available per child. The age span covered was not reported, but 40% were followed up until at least age 11 years. For a subset of about 45%, information on activities and eating habits was also obtained at some point of time. The results indicated a significant association between measured magnetic field and childhood obesity (OR 1.84, 95% CI 1.05-3.22 for the highest exposure category). In this paper, no risk estimate for a continuous exposure indicator was reported. The association was even stronger for those followed up until age 11 years and for persistent obesity (defined as more than half of all weight measurements meeting the 97.5th percentile criterion). In an analysis using body mass index for children aged six years or older, a non-significant association was found for field strength >0.15 µT (OR 1.87, 95% CI 0.90-3.86). The strengths were similar to those mentioned above. Exposure classification was not consistent with the earlier paper and the definition of obesity was not state of the art (as ideally body fat should be measured) or even consistent with the standard definition of overweight or obesity (which is based on body mass index, rather than weight alone). The WHO recommendation is +2 SD in terms of BMI for overweight and +3 SD for obesity, while CDC uses cut-points at 85th and 95th percentiles. The motivation for adoption of non-standard definition in the paper is unclear.

In conclusion, recent results for the first time show an association between ELF fields and childhood obesity and asthma; however, these results need to be reproduced to evaluate their significance for risk assessment.

3.8.4. Conclusions on health effects from ELF fields

Neoplastic diseases

The new epidemiological studies are consistent with earlier findings of an increased risk of childhood leukaemia with estimated daily average exposures above 0.3 to 0.4 µT. As stated in the previous Opinions, no mechanisms have been identified and no support is existing from experimental studies that could explain these findings, which, together with shortcomings of the epidemiological studies prevent a causal interpretation.

Nervous system

Epidemiological studies do not provide convincing evidence of an increased risk of neurodegenerative diseases, including dementia, related to ELF MF exposure. Furthermore, they show no evidence for adverse pregnancy outcomes in relation to ELF MF. The studies concerning childhood health outcomes in relation to maternal residential ELF MF exposure during pregnancy involve some methodological issues that need to be addressed. They suggest implausible effects and need to be replicated independently, before they can be used for risk assessment.

Largely consistent with earlier results, recent studies have reported that exposure to ELF magnetic fields has no effect on activity or locomotion. There is some evidence from animal studies that exposure to ELF MF may affect the performance of spatial memory tasks (both deficits and improvements have been reported) and engender subtle increases in behavioural anxiety and stress. Other studies have investigated potential molecular and cellular mechanisms, and despite a number of studies continue to report

candidate mechanisms, particularly regarding effects on reactive oxygen species, none has been firmly identified that operates at exposure levels found in the everyday environment.

Symptoms

Overall, existing studies do not provide convincing evidence for a causal relationship between ELF MF exposure and self-reported symptoms.

Other effects

Recent results do not show an effect of the ELF fields on the reproductive function in humans.

3.9. Health effects from Static Fields including MRI exposure

3.9.1. Human studies

What was already known?

The previous SCENHIR looked at several studies performed where volunteers were exposed to either the static field of an MRI only, or to a diagnostic procedure which also includes exposure to low and high frequency fields.

The previous Opinion concluded that instantaneous effects on neuronal functioning of movement in particular, through a SMF or SMF gradient as used in clinical practice might be possible. However SCENHIR stressed the need for further confirmation of these studies.

What has been achieved since then?

In 2009 ICNIRP updated their guidelines for static magnetic field exposure, and in the paper (ICNIRP, 2009) a review of the scientific evidence is given, from what is known on the interaction mechanism(s) to epidemiological studies. The new values are 2 T for head and trunk, and for limbs 8 T can be allowed. The values are to be regarded as spatial peak exposure limits.

Since the previous SCENIHR report (2009) a few other studies have been published. A systematic review and meta-analysis of studies, which have assessed the health effects of static magnetic fields, identified four studies published between 1992 and 2007 which included sensory perceptions as an outcome (Heinrich et al., 2011). All four reported effects including dizziness and vertigo. Yamaguchi-Sekino et al. (2011) reviewed the properties of static and pulsed EMF that affect biological systems, and discussed the recent ICNIRP update.

Three further observational studies including subjective outcomes have appeared since the 2009 Opinion was published. These questioned MRI employees occupationally exposed to a 9.4 T MRI (Patel et al., 2008), and healthy volunteers or patients who underwent a 7 T or 1.5 T MRI (Theysohn et al., 2008; Heilmaier et al., 2011). Each study identified several symptoms attributed to the exposure, in particular vertigo. The studies by Theysohn et al. (2008) and Heilmaier et al. (2011) both suggested that 7 T is more likely to result in symptoms than 1.5 T, although these symptoms are seemingly still well tolerated by the majority of patients. Franco et al. (2008) published a review on health effects of exposure to the static magnetic field (SMF) in MRI. From cellular studies they did not find any specific effect as a consequence of exposure to SMF. Studies on volunteers showed that short-term exposure to SMF induces a variety of acute effects: (i) vertigo, nausea and a metallic taste in the mouth occur during body or head movement with SMF in T range, (ii) changes in blood pressure and heart rate within the range of physiological variability occur for exposures to SMF up to 8 T. These findings are in line with several publications on acute transient adverse effects (such as dizziness, nausea, headaches, a metallic taste and visual disturbances) related to exposure to the static and time-varying magnetic fields present in, but also surrounding MRI systems. Nor were effects on cognition measurable immediately after exposure had ended (Schlamann et al.

2010a). Van Nierop et al. (2012) showed that the neuro-cognitive functioning is modulated when human volunteers were exposed to movement in stray field from a 7 T MRI scanner. In a subsequent paper based on the same experiment, van Nierop et al. (2013) tested whether exposure to a sham, low and high static magnetic stray field from their 7 T MRI scanner affected body sway. Reduced postural stability was identified across several measures.

Data from a controlled trial using transcranial magnetic stimulation (TMS) does suggest a transient alteration in cortical excitability after undergoing an MRI investigation (Schlamann et al. 2010b). Although a threshold level seems to exist for at least some of the acute effects (Cavin et al. 2007), all effects could already be measured well below 2 Tesla and 6 T/s.

Heinrich et al. (2013) published a study on how cognitive functions in subjects undergoing MRI are acutely impaired by static magnetic fields. 41 healthy subjects underwent an extensive neuropsychological examination while in MR units of differing field strengths (1.5, 3.0, and 7.0 T), including a mock imager with no magnetic field as a control condition. The exposure was not found to have a significant effect on cognitive function at any field strength. However, sensory perceptions did vary according to field strength. Dizziness, nystagmus, phosphenes, and head ringing were related to the strength of the static magnetic field. Within the same study, also stress markers in blood and saliva were investigated, with a negative outcome (Gilles et al. 2013).

Field surveys of MR engineers (De Vocht et al. 2006b) and nurses (Wilén et al. 2010) routinely working with MRI scanners have further shown that they regularly experience adverse transient effects including dizziness/vertigo, nausea, concentration problems, memory loss, tiredness or drowsiness, illusions of movement, and ringing sensations in the head during their work as well as suffering from sleeping disorder. The frequency of occurrence of these symptoms seemed mainly to be associated to the strength of the MR systems, the time spent in their neighbourhood, and the speed with which workers move through these fields.

Recent experimental studies among volunteers who were placed inside the static magnetic stray field of MRI scanners suggest an effect of static field exposure on eye movement [Roberts et al., 2011; Mian et al., 2013]. Subjects developed nystagmus while lying in the bore of 3 T and 7 T MRI scanners. The authors propose an underlying mechanism based on Lorentz forces acting on the endolymph (i.e. the fluid in the labyrinth of the inner ear) [Roberts et al., 2011; Antunes et al., 2012; Mian et al., 2013; Glover et al., 2014, Ward et al., 2014]. More recently, Theysohn et al. (2014) recorded body axis rotation and sway path in 46 healthy volunteers in total. Participants were exposed to no MR exposure, to a 1.5T MRI exposure or to one of four 7T brain MRI scenarios (normal, with no RF excitation, with no gradients, or with a short duration of exposure achieved by moving participants into the bore and directly out again). Exposure for all sessions lasted 30 minutes, except for the short 'in and out' exposure which lasted about one minute. Sway path data showed significantly greater sway in participants exposed to the 30 minute 7T conditions at two minutes post exposure. This effect had resolved by 15 minutes post exposure. All 7T conditions were also associated with changes in a measure of body axis rotation, which persisted at 15 minutes post exposure.

Schaap et al. (2014) has recently conducted an observational study among more than 300 employees of clinical and research MRI facilities in the Netherlands. The study reports on the occurrence of acute transient symptoms among these workers, and assesses the association of these symptoms with static magnetic field exposure. The study shows an increased incidence of acute transient symptoms among health care and research staff working with closed-bore MRI scanners of 1.5 to 7 Tesla. Incidence of symptoms was positively associated with scanner magnet strength.

Noteworthy is also a recent study by Rauschenberg et al. (2014) on reported symptoms on individuals scanned in 7 and 9.2 T scanners and a study showing promising results

when using diphenhydramine in the prevention of vertigo and nausea among patients at 7 T MRI. Both studies however were on patients and therefore hardly relevant for employees exposed to the stray-fields of ultra-high field MRI-scanners.

The (long-term) health significance of these acute neurobehavioral effects and reported symptoms among employees who repetitively work near MRI systems is as yet unknown. Nevertheless, these dose-dependent effects (De Vocht et al. (2006b), De Vocht et al. (2007b), Wilén et al. (2010) could theoretically lead to an increased risk of accidents and errors by workers that are harmful for themselves or for patients under their care, for example during MRI-guided interventional procedures).

Möllerlökken et al. (2012) investigated if an acute high exposure to EMF could have possible adverse effects on male reproductive health. Twenty-four healthy male volunteers participated in a balanced cross-over study with exposure using a head scan in real MRI with whole-body transmitting coil and one set up for sham MRI exposure. Serum-blood samples of inhibin B, testosterone, prolactine, thyreotropine, luteinizing hormone, follicle stimulating hormone, sex-hormone binding globuline and estradiol were taken before and after the different scans. Neither immediately after, nor after 11 days were any differences observed in the hormone levels comparing real and sham MRI. The lack of effects of EMF on male reproductive hormones should be reassuring to the public and especially for men examined in MRI. Adverse effects on other endpoints than male reproduction or possible chronic effect of multiple MRI scans were not investigated in this study.

There is some evidence on genotoxic effects in patients undergoing MRI examination. Simi et al. (2008) studied the level of micronucleated lymphocytes in cultured lymphocytes of eight subjects before and after a cardiac MRI (CMR). Energy absorbed by the subjects was calculated to range from 19 to 306 J. An increase in micronucleus frequency, measured by the cytokinesis block method, was reported in lymphocyte cultures established immediately after the MRI in all individuals, with a 2-fold increase in mean micronucleus frequency in comparison with the samples collected before the examination. A statistically significant increase in micronuclei was still seen in samples obtained 24 h after the scan but not after 48 h, 72 h, 90 h or 120 h.

Fiechter et al. (2013) studied genotoxic effects in 20 prospectively enrolled patients who underwent 1.5 T CMR. A commercially available MR scanner equipped with a maximum gradient strength of 42 mT/m and a maximum gradient speed of 180 mT/m/ms was used and the mean CMR scan duration was 68 + 22 min with an average contrast media bolus of 15 + 4 ml. Peripheral mononuclear cells were studied for DNA damage using immunofluorescence microscopy of foci positive for phosphorylated gamma-H2AX in nuclear DNA, indicative of sites of DNA double strand break repair. The median and mean numbers of foci per mononuclear cell were, respectively, 0.066 and 0.143 in baseline samples (collected prior CMR scan) and 0.190 and 0.270 after the CMR scan; the difference (1.6-fold for median, 1.9-fold for mean) was statistically significant ($P<0.05$). In addition, gamma-H2AX-positive foci were quantified in CD3-positive T-lymphocytes by flow cytometry. The analysis revealed a statistically significant increase in geometric mean of fluorescence intensity (arbitrary units) of T-lymphocytes after the MRI (median 3232, 1.17-fold; mean 3395, 1.14-fold) as compared with samples collected before the scan (2758; mean 2989) which was statistically significant.

Discussion on human studies

The studies on effects on DNA integrity after an MRI scan are clearly of interest to follow up. However, it is not clear what part of the exposure in the scanner causes the effect: static, switched gradient field or the pulsed RF field. From other *in vivo* and *in vitro* studies it seems unlikely that the static field alone could cause this. Further studies on DNA integrity and MRI exposure are needed, and perhaps it is time to discuss cohort studies of patient undergoing scans.

Conclusion on human studies

Observational studies have shown that movement in strong static magnetic fields may cause subjective outcomes like vertigo and nausea. These are more likely to occur in field strengths above 2 T.

3.9.2. Animal studies

What was already known on this subject?

The previous SCENIHR Opinion pointed out that despite the fact that there are quite a few studies published, the data are still not adequate for a proper risk assessment, primarily because of many mixed and sometimes contradictory findings.

What has been achieved since then?

Several studies on animals have been published since the previous Opinion, covering work on nervous system effects and behaviour, embryonic development, and various physiological parameters and organ functions. In addition, there are also studies aimed at understanding more basic interaction mechanisms.

There are no studies that have directly investigated any relationship between SMF exposure and tumour development. However, one study (Strelczyk et al. 2009) investigated Syrian gold hamsters carrying syngenic A-Mel-3 melanomas implanted into the dorsal skin. Three days after tumour cell deposition, animals were exposed to a 586 mT SMF for 3 h. Subsequently, tumour angiogenesis and microcirculation as well as tumour development was followed for seven days. Compared to control animals, tumours in exposed animals were growing more slowly, and displayed impaired microcirculation (investigated with *in vivo* fluorescence microscopy). Additional histologic investigations suggested that the vessels in SMF-exposed tumours were fewer in number and with structural deficiencies.

<u>Reproduction and development</u>

The nematode *C. elegans* is a recognized and valuable model system for studies of many biological processes, especially on the molecular levels, including development and aging. The usability is due in part to the short life-span and a multitude of well characterized mutant strains that are available. In a study by Hung et al. (2010), both wild-type and mutant nematodes were exposed to SMF (up to 200 mT; continuously during the entire experiment). In wild-type nematodes, the maximal life-span was shortened from 31 to 24 days by a 200 mT exposure, and the median life span from 16 to 13 days. The expression of genes known to be associated with aging and development of *C. elegans* were investigated with quantitative real-time RT-PCR, showing that SMF exposure indeed affected expression of several genes (clk-1, lim-7, unc-3, age-1). In addition, mutant nematodes deficient in these genes did not respond to the SMF. The shortening of the life-span was in further experiments seen to be a function of accelerating through larval stages of development. Almost all significant effects were seen at 200 mT, whereas lower B-field strengths mostly were ineffective.

Another model organism was used by Savic et al. (2011) who followed development and viability in the fruitfly *D. melanogaster*, from egg to adult. In parallel, a closely related species, *D. hydei* was also studied. The specimens were exposed to a 60 mT SMF continuously during the investigated period. There was a small but statistically significant reduction of developmental time in *D. hydei*, and decreased viability (measured as percentage of eclosed adults) in both species. The eclosion was also faster in both species.

In a study on pregnant mice (C57BL/6) Laszlo et al. (2009) showed that a gradient SMF (2.8-476.7 mT whole body exposure) delayed preterm birth induced by the bacterial endotoxin LPS. The exposure occurred for 40 min on a daily basis, starting either on day 1 or day 14 of gestation. LPS was administered on day 15 and preterm birth was expected within 17 h. The group treated from day 1 had preterm birth delayed more than those that were treated the day before LPS injection.

Spermatogenesis in adult male albino rats was studied by Monfarad et al. (2009). The animals were exposed to a 1.5 T MF (exposure poorly described) for 30 min, with or without prior treatment to vitamin C and/or vitamin E (intraperitoneal injection 30 min before MF exposure) and sacrificed 16 or 29 days post exposure. On histological sections, germ cell number and seminiferous tubule diameter were investigated. Both end-points were reduced in the exposed animals, an effect which was counteracted by the vitamin injections.

Nervous system effects

The group of Houpt et al. have published a series of studies on the effects of strong static MF on behaviour. The studies have typically employed female adult Sprague-Dawley rats, which were exposed to a 14.1 T SMF, within the bore of a magnetic used for MRI (with the RF off). This field strength level is very high, and not very likely encountered. It has previously been reported that strong MF causes vertigo and furthermore circling behaviour, acquisition of a condition taste aversion (CTA) to saccharine, and induction of c-fos in the brain stem of rodents (Houpt et al. 2003). In a study by Cason et al. (2009), the hypothesis that such effects of SMF are dependent on the vestibular apparatus in the inner ear was tested. Chemically labyrinth-ectomized rats (by intratympanic injections of Na-arsanalite, which destroys the hair cells) as well as intact but sham-labyrinth-ectomized (saline injection instead of Na-arsanalite) rats were exposed (30 min) to the 14.1 T MF. Intact rats displayed expected behaviour (circling, saccharine avoidance) and increased c-fos expression, whereas ectomized rats showed no increase in circling, did not acquire a CTA, or display elevated c-fos levels. In another study from the same group (Houpt et al. 2010), the experimental paradigm was used to show that repeated treatment (2-3 times 30 min) to the 14.1 T MF causes habituation. Only momentary passages into and out of the MF was enough for CTA, whereas longer exposures were needed for circling to occur (Houpt et al. 2011), suggesting that substantial exposure time is needed for rats to display all behavioural effects of exposure. Finally, the most recent study (Houpt et al. 2012) shows that rats immediately tend to tilt their heads during exposure, in a direction opposite to the circling direction.

The group of Laszlo et al. have published several studies on SMF and pain reduction. The study by Antal and Laszlo (2009) showed that whole body exposure of adult male mice (Balb/c) for 30 min, once per day (14 days) to an inhomogeneous SMF (476 mT peak) alleviates allodynic pain in the hind paw. There was a modest effect if the exposure was applied on days 1-14 post operation, and a much stronger effect if the exposure took place on days 15-28 after surgery. Pain reduction was seen also in another experimental paradigm, where male CFLP mice were subjected to a writhing test (Laszlo and Gyires 2009). Pain was induced by i.p. injection of 0.6% acetic acid, whereafter the stretching and writhing movements of the animals were recorded. Animals were either exposed to a 0.1, 0.3, or 3 mT field outside an MRI magnet, or to a 3 T field inside the magnet bore. The exposure followed immediately upon acetic acid injection, and the animals´ reactions were followed for 30 min. The 3 T exposure reduced the writhing frequency compared to controls with 68%, which was significantly different from all other treatments. A different exposure system was used in another work (Laszlo et al. 2009), where the male CFLP mice were once again subjected to the writhing-inducing acetic acid injections. The exposure (inhomogeneous SMF, 2-754 mT) significantly reduced writhing 10, 20, or 30 min after exposure (also 10, 20 or 30 min). In order to see if behaviour characteristics were affected by the MF exposure, possible anxiogenic or anxiolytic effects were studied with the elevated plus maze test, and locomotor activity was investigated by means of the "Conducta System for behavioural and activity studies". No other effects than reduced writhing were seen after exposures. It is unclear from these articles if blinded conditions were used when possible.

A very comprehensive study was published by Hoyer et al. (2012) who exposed pregnant mice to a 7 T static MF daily (75 min per day) from day 1.5 to 18.5 post conception. This period allows for exposure to be present during implantation, early embryonic development, and organogenesis, all very sensitive stages in development. Pups were

subsequently investigated with a battery of behavioural tests, from an age of 10 weeks and onwards, which means that the animals were adult during testing. Both exposure and sham exposure (a mock MRI scanner) were performed. In addition, a sound recording was made from the MF exposure situation and played back to the sham exposed animals. Tests were performed on two cohorts (separated six months in time), that comprised both male and female offspring. In total, 26 male animals and 18 females were investigated. No differences in body weight between exposed and sham exposed were noticed, although gender differences were seen (males heavier in both cohorts). Exploration behaviour was investigated by Novel Cage, Open Field, and Novel Object Tests, with no documented exposure effects. Absence of exposure effects was also documented after motor coordination tests (Rotarod), thermal pain sensitivity (Hot Plate Test), anxiety like behaviour (Elevated O-Maze, Dark-Light-Box Test), associative learning (fear conditioning), and spatial working memory (T-Maze). There was a trend (statistically not significant) towards an effect of exposure for immobility latency in the Porsolt Forced Swim Test, which investigated depressive-like behaviour. This study, thus, indicates that repeated pre-natal exposure to a 7 T MF does not exert adverse effects on emotional and cognitive behaviour in the adult mouse.

Two additional studies from the same group regarding fertility, pregnancy and embryonic and post-embryonic development was subsequently published. In Zaun et al. (2013), mice were exposed daily in utero to different static magnetic field strengths at the bore entrance or in the isocenter of 1.5 T and 7 T MRI systems during the entire course of prenatal development. The reproductive organs and the fertility in adulthood of these animals were investigated. The only observed effect was reduced placental weight in the offspring of the in utero exposed female mice. Other parameters including fertility were not affected. In the study by Zahedi et al. (2013), pregnant mice were exposed for 75 minutes daily during the entire course of pregnancy at the bore entrance, representing the position of medical staff, and at the isocenter, representing the position of patients, of a 1.5 T and a 7 T human MRI scanner. The authors reported that no effect of static magnetic field strength was observed with regard to pregnancy rate, duration of pregnancy, litter size, still births, malformations, sex distribution, or postpartum death of offspring. During the first 8 weeks of postnatal development, mice exposed in utero to a magnetic field strength of 1.5 T or stronger showed a slight delay in weight gain and in time to eye opening compared to controls.

Another approach to see if behaviour is affected by SMF was seen in a paper by Lee et al. (2012) who performed experiments on the nematode *C. elegans*. Adult worms were exposed for up to eight days in SMF ranging from zero to 200 mT. The mobility end-points crawling speed and mobility (number of sine waves propagating per minute along the body axis) were recorded. A significant decline (ca 25-40%) in both end-points was seen from exposure for four days and longer, at field strengths of 150 and 200 mT (stronger effects at 200 mT). Gene expression analysis of 120 randomly selected genes revealed that certain genes involved in apoptosis and oxidative stress were upregulated by exposure. The importance of apoptotic pathways for the mobility decline increased by the SMF was then further strengthened by use of selected mutant nematode strains. Exposure to a 200 mT static MF did not cause mobility decline in these animals.

A combination of an *in vivo* and *in vitro study* was presented in the interesting paper by Nikolic and co-workers (2012) who employed the spontaneously active Br neuron from the brain-subesophageal ganglion of the snail *Helix pomatia*. Both the intact snail and the isolated Br neuron were exposed to a 10 mT SMF for 15 min. In the brain, exposure caused increases in the activity of the Na^+/K^+-ATPase (the "Na+/K+-pump"), in the activity of the Na^+/H^+-exchanger (leading to more alkaline cellular conditions,) and increased ATP consumption. Current clamp recording of the dissected neuron confirmed the increased activity of the Na^+/K^+-ATPase, leading to a hyperpolarization of the membrane resting potential. These effects were abolished if agents blocking phosphorylation/de-phosphorylation were administered during exposure, suggesting that this exposure primarily causes changes in phosphorylation status of membrane-

associated proteins in specific signal transduction pathways, which then lead to effects on the physiology of the cell.

Metabolism

Some studies on effects on *in vivo* metabolism have been published in recent years. A series of papers from the same group (Elferchichi et al. 2010a; 2010b; 2011; Jahbib et al. 2010) have repeatedly investigated a 128 mT SMF and its effect on glucose and lipid metabolism in 6-7 week old male Wistar rats. In one study (Elferchichi et al. 2010a), animals were exposed for 1 h/day during 15 days. At the end of the exposure period, a series of parameters were measured during post-prandial conditions. The exposed group (six animals) displayed increased levels of blood glucose, whereas the insulin levels were lowered. Furthermore, increased levels of glycerol, cholesterol, phospholipids and lactate were documented, whereas triglyceride levels did not deviate from those in control animals (n=6). A glucose tolerance test on fasted animals showed a significant increase in blood glucose among exposed, noticeable after 20 min. On the tissue level, glycogen depositions in skeletal muscle and liver were depleted (44% and 25% decrease compared to controls) in exposed rats. In another study (Elferchichi et al. 2011) the same group used the same experimental protocols in a comparison with Zucker rats (a diabetic strain). The outcome of the SMF exposure on the Wistar rats (hyperglycemia, low insulin, depleted glycogen reserves) overlapped with non-exposed Zucker rats. The conclusion is that the SMF exposure triggers a pre-diabetic state in normal rats. In Lahbib et al. (2010) the results of exposure is that if exposure is 15 days instead of five, effects on glucose and lipid metabolism are more pronounced. It is unclear if these three papers constitute separate studies, or if the results from one single experiment are used in separate papers. Furthermore, the numbers of animals are small, and it is not clear if the animals are from the same or separate litters.

A contradictory finding regarding effects on glucose metabolism is provided in a study from Laszlo et al. (2011) where CD1 mice are exposed to an inhomogeneous static MF (2.8-476.7 mT peak-to-peak). The authors investigate body weight (although only in another strain, CFLP), blood glucose and nociceptive temperature threshold (increasing temperature hot-plate test) in exposed and sham-exposed rats, as well as in rats made diabetic with streptozotocin (STZ). Exposure went on daily (30 min) for up to 12 weeks. MF exposure had no effects on the investigated end-points in normal rats, whereas in the group treated with the highest levels of STZ (and thus most diabetic), the exposure caused a significant glucose decrease. This outcome is opposite to the diabetogenic effects of SMF reported above. A major difference is naturally the different species (rats and mice respectively). Both research groups fail to report if blinded conditions were employed or not.

In yet another paper from Elferchichi et al. (2010b), the effects of SMF on ionic composition in the rat spinal cord were investigated. These are probably the same animals as those that were used in the other studies from this group. At the end of the five day exposure period, (128 mT; 1 h/day), samples from the cerebrospinal fluid (CSF) and from the blood serum were analysed with respect to calcium (increase in CSF after exposure, unchanged in serum), iron (increased in CSF, decreased in serum), magnesium (unchanged) and copper (unchanged).

Other effects

Lin and co-workers (2009) performed a study where 5-week-old Balb/c mice were injected with the bacterial endotoxin LPS (lipopolysaccharide) which causes sepsis. The LPS was injected intraperitoneally at 50 mg/kg which caused 90% mortality after 48 h. Animals were either controls, or treated with a 0.25 T static MF for 1 or 2 h before LPS administration, alternatively after the LPS injection. The survival rate was higher in SMF-treated animals than in unexposed, and highest (47%) in the group pre-treated for 2 h before LPS. Further studies suggested that the SMF may cause this protective effect by stimulating release of IL-1ra (interleukin-1 receptor agonist), which would counteract the pro-inflammatory actions of IL-1 that LPS causes.

Wang et al. (2009) employed a gradient SMF (0.2-0.4 T; 2.09 T/m; exposure 1-11 days) to investigate SMF effects on angiogenesis *in vivo* an *in vitro*. The *in vivo* model (the chicken choioallantoic membrane) displayed significantly lower vascular numbers, and also lower haemoglobin content than unexposed samples.

Wound healing in diabetic 3 month old Sprague-Dawley rats was improved by 180 mT SMF (Shen et al. 2010). Wound healing rate, gross healing time, and wound tensile strength were all positively influenced by exposure (5-19 days).

The use of SMF for blood pressure buffering during acute blood pressure rise was investigated in a study on adult male rabbits (Gmitrov 2010). Blood pressure was pharmacologically increased (successive injections of nitroprusside and phenylephrine), and the effects of a 300 mT SMF were compared to those of the calcium-channel blocker verapamil. The permanent magnets generating the MF were located at the level of the sinocarotid baroreceptors. Exposure for 40 min caused a significant buffering of the blood pressure increase, although at a level lower than the ones obtained with verapamil.

Exposure of male adult Wistar rats to a 128 mT SMF (1 h/day; 5 days) caused changes in radical homeostasis, specifically antioxidant enzymes (Ghodbane et al. 2011). Concomitantly, the exposure was seen to deplete selenium levels (kidney, muscle, brain), which was suggested by the authors to cause disturbances in the antioxidant systems.

Discussion on *in vivo* effects

A number of studies are reporting that effects occur with SMF exposures in animals, at B-field levels from mT – T. However, many of the findings are limited to single studies in the specific area, and need replications before any firm conclusions can be drawn.

Over the years, many studies report on effects on the nervous system. Several of the findings regarding nervous system effects reported here are contradictory. On one hand, studies that are reporting pain reduction are consistent and in line with what the group in question have reported previously. On the other hand, the studies where behaviour has been investigated, including at very high field strength levels, are not generating consistent effects. Mechanistic studies addressing basic effects on neurons would have the potential to resolve several of these inconsistencies.

Inconsistency is also obvious in the studies focussing on glucose and lipid metabolism. Similar exposure conditions are causing opposite effects, in rats and mice respectively.

Conclusions on *in vivo* effects

Taken together, the findings reported here do not provide any firmer foundation for a proper risk assessment of static MF exposure than what was available for the previous Opinion.

3.9.3. *In vitro* studies

What was already known on this subject?

Concerning *in vitro* studies the previous Opinion of 2009 stated that the results support the hypothesis that SMFs can affect the expression of specific genes in mammalian cells, although the effect is dependent on the exposure characteristics (duration, field gradient). Studies on genotoxicity, cell growth and apoptosis provided not univocal results.

What has been achieved since then?

Several endpoints have been investigated after exposure of different cell types to SMFs. The results are reported below and summarized in Table 17.

Gene expression and genotoxicity

Alteration of gene expression has been detected in several investigations carried out using primary mammalian cells as well as cell lines exposed to SMFs from few µTesla up to 10 T.

Changes in the expression of MACF-1, a gene encoding for cytoskeletal proteins, were detected in osteoblast-like cells exposed to large gradient high MF (magnetic force fields of -1360, 0 and 1312T^2/m). Different effects (up- or down-regulation) were found as a function of the exposure conditions (Qian et al., 2009).

Up-regulation of hematopoietic and cell cycle-related genes was found in human placental and umbilical cord blood cells exposed to 10 T for 16 h (Monzen et al., 2009).

By exposing HUVEC cells to 60 or 120 µT for 1 or 24 h Martino and co-workers found no changes of VEGF (vascular endothelial growth factor) gene expression, although an up-regulation of the eNOS (endothelial Nitric Oxide Synthase) was recorded after 24 h exposure (Martino et al., 2010).

Feng and co-workers (2013) exposed human A549 lung adenocarcinoma cells for 1-4 h to a 0.5 T, MF. Cell growth inhibition was detected in exposed cultures compared to controls (no sham exposed samples were included in the study). Moreover, microarray of cells exposed for 1 hour to MF showed that 19 cell cycle and apoptosis-related genes had 2-fold up-regulation and 40 genes had 2-fold down-regulation. MF also significantly arrested cells in G2 and M phases.

Politanski et al. (2013) investigated the effect of static magnetic fields on ROS formation in lymphocytes from male albino Wistar rats. The exposure was carried out at 0 mT (50 µT magnetic field induction opposite to the geomagnetic field) and 5 mT by placing cell cultures inside a pair of Helmholtz coils, which provided a highly homogenous field. ROS formation was measured after 15 min, 1 and 2 h of exposure to the SMF by using the fluorescent probe 2′,7′-dichlorofluorescin diacetate. The results indicated that a significant increase in ROS formation was induced by 5 mT exposure compared either to unexposed cultures and to cultures exposed to 0 mT. In this paper, the effect of co-exposures to SMF and x rays was also investigated (see section 3.11.2). Comparisons were carried out with respect to control cultures (no sham-exposed cultures were set up).

Gioia et al. (2013) evaluated the effect of chronic exposure to a 2mT SMF on primary cultures of swine granulosa cells (GCs). This cell model was selected due to its pivotal role in female reproduction; in fact, GCs surround and sustain the development of the female gamete, providing metabolic and regulatory factors for the oocyte. Moreover, they have an important endocrine activity concurring to the ovarian steroid hormones production.

No effect on cell viability was detected, but the doubling time was significantly reduced in exposed samples after 72 h of culture. At the same time, the cell length and thickness significantly changed, while the cell orientation was unaffected. Evident modifications were induced on actin and a-tubulin cytoskeleton after 3 days of exposure and, simultaneously, a change in intracellular Ca^{2+} concentration ([Ca^{2+}]i) and mitochondrial activity started to become evident. Moreover, exposure longer than 72 h determined a significant alteration of progesterone and estrogen production. In this study, no sham-exposed samples were set up. The results obtained in exposed cultures were compared to control cultures (incubator).

Nakamichi and co-workers exposed primary foetal rat brain progenitor cells to 100 mT from 2 up to12 days. A promotion of differentiation into neurons through over-expression of proneural genes was detected after 12 days exposure, but not for shorter exposure times (Nakamichi et al., 2009). Similar results were reported by Wang et al., who detected differentiation of human embryoid body derived cells after exposure to 0.25 T for several days (Wang et al., 2009). A transient up-regulation of several genes involved in cell division was also reported by Polidori et al. (2012) in HUVEC cells exposed for short (4 h) or long (24 h) periods to SMF of comparable intensity (0.3 T). The same

research group reported increased expression of one of the main genes related to mitochondrial biogenesis in the same experimental conditions (HUVEC cells exposed to 0.3 T for 24 h), together with an increase in ROS formation after 4 h, that reverted after 24 h exposure. Meanwhile, DNA damage was observed for exposure durations of 2, 4 and 24 h and unaffected for longer periods (48 and 72 h) (Potenza et al., 2010).

Expression of HSP70 was evaluated by Laramee and co-workers. They exposed rat primary fibroblast cells (RAT1), transfected with a HSP70 promoter-linked luciferase reporter, to static magnetic flux densities of 1 to 440 mT for 16, 24, and 48 h starting at both 24 and 48 h post transfection. HSP70 expression was followed for up to 96 h and showed a dependence on flux density, exposure duration, and start time post transfection. A nonlinear response in expression was observed for increasing flux density with a maximum of a 3.5-fold increase over control occurring at 48 h of exposure starting 48 h after transfection. Laramee et al., (2014)

DNA damage was also evaluated by other research groups. An increase in DNA migration was detected in human lymphocytes exposed for 1 h to inhomogeneous (0.3, 1.2 or 47.7 T/m) SMF or for 4 and 18 h to homogeneous (160 mT) SMF (Kubinyi et al., 2010). On the contrary, lower SMF (8.8 mT) did not induce alterations in DNA migration of human leukemic cells exposed for 12 h (Chen et al., 2010; Qi et al., 2011).

Exposure of human lymphoblastoid TK6 cells to 705 mT SMF led to a reduction in the level of both constitutive ɣH2AX phosphorylation and ATM activation (two parameters related to repair of constitutive and induced DNA damage). The effect was not cell cycle phase specific as the decrease was comparable across all phases of the cycle and was detected after 5 and 24 h exposure, although in the latter case an higher difference respect to unexposed cultures was recorded (Halicka et al., 2009). The authors stated that, since the constitutive DNA damage is one of the main causes of aging and predisposition to cancer, the effect detected can be regarded as protective.

Oxidative stress and membrane effects

Three papers reported transient increase in ROS production, consistent with the hypothesis that SMF can interfere with the cell redox status. A sharp increase was detected in human embryonic lung fibroblasts exposed for 18 h to a magnetic field ranging from 35 to 120 mT. The effect reverted after 5 days continuous exposure (Sullivan et al., 2011). Transient increase in ROS levels was also reported in HUVEC cells after exposure to 300 mT for 4 h, which reverted after 24 h exposure (Potenza et al., 2010). Zhao and co-workers reported an increase in ROS level in two human-hamster hybrid cells (A_L and ρ^0 A_L cells) and in Chinese Hamster ovary-derived cells (XRS-5) after three h exposure to 8.5 T SMF. Adenosine triphosphate (ATP) content was significantly decreased in AL cells exposed to 8.5 T but not to 1 or 4T SMF for either 3 or 5h. In addition, ATP content significantly decreased in the two deficient cell lines (ρ^0 AL and XLS-5) exposed to 8.5T SMF for 3h. With further incubation of 12 or 24h without SMF exposure, ATP content retrieved to the control level in the hybrid but not in the deficient cells (Zhao et al., 2011).

Changes in cell membrane ultrastructure (increase in cell membrane permeability) were reported in human leukemic cells exposed to 8.8 mT for 12 h by the group of Qi (Chen et al., 2010; Liu et al., 2011). Alteration of calcium flux was detected by Wang et al. in rat pheochromocytoma cells (PC12) exposed up to 3 h to a SMF ranging from 0.23 to 0.28 T. Moreover, increased ATP levels and reduced cAMP levels, Nitric Oxide production, p44/42 MAPK phosphorylation, together with a decrease in cell proliferation and iron uptake were also found. Since these effects are qualitatively similar to those obtained with a class of drugs candidates for treatment of Parkinson’s disease (PD), the authors suggest that SMF could be a promising non-invasive tool to treat PD and potentially other neurological disorders (Wang et al., 2010).

No membrane protrusion was observed in rat spinal cord astrocytes exposed to 2.1 T up to 72 h (Khodarahmi et al., 2010).

Cell growth, differentiation and viability

The results reported on cell growth and viability are not univocal. No effect was detected in HUVEC cells exposed from 4 up to 72 h to a 300 mT SMF (Potenza et al., 2010). Primary cultures of rat astroglial cells also resulted unaffected by higher SMF exposure (2.1 T) (Khodarahmi et al., 2010). Similar results were obtained in terms of cell cycle progression both by Zhao et al. (2011), who exposed human-hamster hybrid cells and CHO-derived cells for 3 h to 8.5 T, and by Sarvestani et al. (2010) on rat bone marrow stem cells exposed for 5 h to SMF of lower intensity (15 mT).

Dini and Panzarini (2010) reported that exposure to 6 mT of several cell types induced a decrease in phagocytosis and endocytosis and an increase in apoptotic rate. Such effects resulted dependent on the degree of macrophage differentiation.

In three papers, an increase in cell proliferation of HUVEC cells was noted. Polidori et al. (2012) reported a 25 % enhancement in cell proliferation after 4 h exposure to a 300 mT SMF, together with a transient up-regulation of several genes involved in cell growth and division. Martino et al. also found an increase in cell number either after 24 h (but not after 1 h) exposure to 60 or 120 µT SMF (Martino et al., 2010) and 48 h exposure (Martino et al., 2011). In the latter case, the resulting effect was suppressed by treatments with free-radical scavengers.

Different results were found by other authors. A reduction in cell proliferation was also detected in PC12 cells exposed for 3 days (Wang et al., 2010) and in human embryoid body derived (LVEC) cells exposed up to 6 days to a SMF ranging from 0.23 to 0.28 T (Wang et al., 2009). In the latter the authors also recorded case changes in gene expression related to signalling and differentiation and altered morphology. The effect resulted in cell type dependent since no variation with respect to unexposed cells was detected in human embryoid kidney (HEK AD293) cells. Feng and co-workers found a decrease in proliferation of human osteosarcoma cells, grown on a surface of poly-L-lactide (PLLA) substrate and exposed to 0.4 mT for 5 days. The effect was recorded after 1 and 3 days of exposure. In addition, cells showed a more differentiated phenotype after 1 day exposure (Feng et al., 2010). Similar results were detected in primary foetal rat brain progenitor cells that decreased cell proliferation and differentiated into neurons (over-expression of proneuronal genes) under 100 mT SMF for 12 days. Shorter exposure duration did not result in any effect (Nakamichi et al., 2009).

Up-regulation of hematopoietic- and cell cycle-related genes and increase in the number of hematopoietic progenitor cells was found in human placental and umbilical cord blood CD34 cells exposed to 10 T for 16 h. Also, in this case, shorter exposure duration did not exert any effect (Monzen et al., 2009).

Table 17. *In vitro* studies on static magnetic fields (SMF)

Reference	Cell type	SMF exposure conditions	Results
Qian et al., 2009	Human (MG-63) and murine (MC3T3-E1) osteoblastic cell lines	LG-HMF, -1360, 0, 1312 T^2/m<br>24 h	Decrease or increase in cell viability and changes in MACF1 expression as a function of the exposure conditions in G0/G1 phase
Monzen et al., 2009	Human placental and umbilical cord blood cells (CD34)	5 T (41.7 T/m); 10 T (0 T/m)<br>4-16 h	Increase in number of hematopoietic progenitor cells and in expression of either early hematopoietic- and cell cycle-related genes (10 T, 16 h)
Martino et al., 2010	Human umbilical vein endothelial cells (HUVECs)	60 or 120 µT<br>1-24 h	Increase in cell number and up-regulation of e-NOS. No changes of VEGF gene expression. No effects after 1 h exposure
Feng et al., 2013	human lung adenocarcinoma cells (A549)	0.5 T<br>1-4 h	Cell cycle and apoptosis-related genes up- or down-regulated.<br>Cells arrested in G2 and M phases

Politanski et al. (2013)	Lymphocytes from Wistar albino rats	0 mT (50 µT MF opposite to the geomagnetic field) and 5 mT 15 min, 1 and 2 h	Increase in ROS formation at 5 mT
Gioia et al. (2013)	Primary swine granulosa cells (GCs)	2 mT 72 h – 3 days	Reduction in doubling time, without effect on viability and alteration of progesterone and estrogen production for exposure longer than 72 h. Morphological, intracellular Ca^{2+} and mitochondrial activity modifications after 3-day exposure.
Nakamichi et al., 2009	Primary foetal rat brain progenitor cells	100 mT From 2 to 12 dd	Decrease in cell proliferation and promotion of differentiation into neurons trough over-expression of proneuronal genes. Effect after 12 dd exposure but not for shorter exposure times.
Wang et al., 2009	Human embryoid body derived cells (LVEC); Human embryoid kidney cells (HEK AD 293)	0.23-0.28 T 1h - 9dd	Changes in gene expression related to signalling and differentiation. Time-dependent regulation of IL-6 signalling
Polidori et al., 2012	Human umbilical vein endothelial cells (HUVECs)	300 mT 4, 24, h	Transient up-regulation of several genes involved in cell growth and division after 4 h exposure together with enhanced cell proliferation (25%)
Potenza et al., 2010	Human umbilical vein endothelial cells (HUVECs)	300 mT 4, 24, 48, 72 h	No effect on cell viability; reduction of mitochondrial content and increase in ROS production after 4 h exposure; enhancement of mitochondrial content after 24 h. No effects after 48 and 72 h exposure.
Laramee et a., 2014	Rat primary fibroblast cells (RAT1),	1 to 440 mT 16, 24, and 48 h	Nonlinear response in HSP70 expression upon increasing flux density
Kubinyi et al., 2010	Human peripheral blood leukocytes	Inhomogeneous SMF 0.3, 1.2, 47.7 T/m Homogeneous SMF 159.2 ± 13.4 mT 0.5 min – 24 h	Increase in DNA migration (comet) as a function of the experimental protocol adopted.
Chen et al., 2010	Human leukaemic cells (K562)	8.8 mT 12 h	Changes in cell surface ultrastructure (cell membrane permeability); no effect on DNA migration (comet).
Qi et al., 2011	Human leukemic cells (K562)	8.8 mT 12 h	No effects on metabolic activity.
Halicka et al., 2009	Human leukemic cells (TK6)	705 mT 5 and 24 h	Reduction in the level of constitutive ɣ H2AX phosphorylation and ATM activation
Sullivan et al., 2011	Human embryonic lung fibroblasts (WI-38); adult skin fibroblasts (AG11020); adult adipose stem cell line (SBMCO46); human melanoma (LIDRU80)	35-120 mT 18 h – 14 dd	Decreased cell attachment on the flask bottom and cell growth. Transitory sharp increase in ROS production as a function of cell type and exposure duration.
Zhao et al., 2011	Human-hamster hybrid cells (A_L and ρ^0 A_L); Chinese Hamster Ovary-derived cells (XRS-5)	1, 4, 8.5 T 3 or 5 h	Decrease in ATP content as a function of the cell type investigated. Increase in ROS production at 8.5 T for 3 h in all cell lines. No effect on cell cycle distribution and CD-59 mutation frequency.
Liu et al., 2011	Human leukemic cells (K562)	9 mT 12-24 h	Changes in cell surface ultrastructure.
Wang et al., 2010	Rat pheochromocytoma cells (PC12)	0.23-0.28 T 10 min - 3dd	Altered calcium flux, increased ATP levels, reduced cAMP levels, NO production, p44/42 MAPK phosphorylation, proliferation and iron uptake, reproducing the effect of ZM241385.
Khodarahmi et al., 2010	Primary cultures of rat astroglial cells	2.1 T 4-72 h	No effects on viability and morphological properties
Sarvestani et	Rat bone marrow stem	15 mT	No effects on cell cycle progression.

al., 2010	cells	5 h	
Dini and Panzarini, 2010	Human myeloid leukaemia promonocytes (U-937); Human Kupffer cells; Murine macrophages (RAW 264.7); TPA-differentiated monocytes (THP-1);	6 mT<br>1 - 4 h	Decrease in phagocytosis efficiency of apoptotic U-937 by several monocyte cell lines and increase in apoptotic rate. Greater effect at the late stage of the macrophage differentiation.
Martino, 2011	Human umbilical vein endothelial cells (HUVECs)	30 and 120 μT<br>48 h	Increased cell proliferation respect to unexposed cultures (0.2-1 μT); effect suppressed by free radical scavengers
Feng et al., 2010	Human osteosarcoma cells (MG63)	0.4 mT<br>5 dd	Decrease in cell proliferation. Increase in extracellular matrix production (more differentiated phenotype)

ATM: protein kinase mutated in ataxia-telangiectasia; e-NOS: endothelial-Nitric Oxide Synthase; IL-6: Interleukin 6; LG-HMF: Large Gradient High Magnetic Field; MACF1: Microtubule Actin Crosslinking Factor 1; MAPK: mitogen-activated protein kinase; ROS: reactive oxygen species; VEGF: Vascular endothelial growth factor.

Conclusions on *in vitro* effects

In most of the available studies, SMF induced effects in the cellular endpoints investigated, although in some cases the effects were transient. Gene expression was affected in all studies, with predominantly up-regulated outcomes. These new studies confirm the previous SCENIHR conclusions.

3.9.4. Conclusion on health effects from SMF exposure

In most of the available *in vitro* studies, SMF above 30 μT induced effects in the cellular endpoints investigated, although in some cases the effects were transient. Gene expression was affected in all studies, with predominantly up-regulated outcomes. These new studies are consistent with the results of previous studies.

A number of studies are reporting that effects of SMF exposures occur in animals, at levels ranging from mT to T. However, since many of the findings are limited to single studies, they do not provide any firm foundation for risk assessment.

Observational studies have shown that movement in strong SMF may cause effects, such as vertigo and nausea. These can be explained by established interaction mechanisms and are more likely to occur in fields above 2 T. The relevance of these effects for the health of personnel remains unclear.

3.10. Health effects from combined exposure to different EMF

What was already known on this subject?

In the previous Opinion of 2009 the topic related to combined exposures to more than one EMF frequency was not discussed.

What has been achieved since then?

3.10.1. *Combined exposure in Magnetic Resonance Imaging (MRI) environment*

In the MRI environment, workers and patients are exposed to high static magnetic fields, fast gradient magnetic fields and strong radiofrequency electromagnetic fields. Thus, exposure in MRI suite represents a particular case of combined exposure to different EMF.

Most of the investigations carried out on this topic refer to human studies. In particular, Schlamann et al. (2010) investigated possible cognitive effects of MRI examinations at 1.5 and 7 T by means of transcranial magnetic stimulation (TMS). In 12 healthy, right-handed male volunteers TMS was performed, first to specify the individual motor threshold, and then the cortical silent period (SP) was measured. Then, the volunteers

were exposed to the 1.5-T MRI scanner for 63 minutes using standard sequences. After the MRI examination another TMS session followed. Fifteen minutes later, TMS was repeated. Four weeks later, the complete setting was repeated using a 7 T scanner. Controls were lying in the 1.5 T scanner for 63 minutes without scanning and lying in a separate room for 63 minutes. TMS was performed in the same way in each case. Immediately after MRI exposure, the SP was highly significantly prolonged in all 12 subjects at 1.5 and 7 T. The motor threshold was significantly increased. Fifteen minutes after the examination, the measured value tended toward normal again. Control conditions revealed no significant differences. The transitory effects on human cortical excitability seen in the study do not seem to be caused by the static magnetic field, since no significant differences between the examinations at 1.5 and 7 T were detected. The radiofrequency pulses and/or the gradient fields seem to be responsible for the measured effects.

In an editorial, Bluemke (2010) commented on these results and asked if they had discovered a new physiological effect. However, he says that the answer is not clear since several controls in their study are lacking. The acoustic noise is very high during MRI scanning, and it is possible that the TMS parameters could be affected by brain exposure to high sound levels. The reproducibility and reliability of the TMS machine are unknown. Unfortunately, Schlamamann et al. (2010) used a wide variety of MRI pulse sequences, including both gradient-echo and spin-echo sequences. These sequences vary widely in their duty factors and energy deposition. And as pointed out by both the authors and in the editorial, further studies are necessary to explore the cause and possible clinical impact of these effects since the cellular, molecular, and apparently neurologic effects of these high–field strength MRI scanners are largely unknown and must continue to be investigated.

Gobba et al. (2012) reported that three female health operators with implanted copper IUDs, had developed menometrorrhagia (a condition in which prolonged or excessive uterine bleeding occurs irregularly and more frequently than normal) some months after an increase of the working time in a Magnetic Resonance Imaging (MRI) Unit (1.5 T), that progressively disappeared when the previous organization, involving discontinuous work shifts at MRI, was re-established. No known factors were evidenced in the 3 operators. A possible mechanism is suggested to be the low-frequency currents induced in the wires of the IUD during the movements of the operator inside the static magnetic field. The problem of possible interactions between copper IUDs and EMF induced by MRI has been considered in patients undergoing imaging, but the possible risk in MRI Unit operators has been largely neglected. Gobba et al. conclude that the possibility that MRI operators with implanted metallic IUDs should be included in the group of "workers at particular risk" according to the EU Directive 2004/40/EC.

Möllerlökken et al. (2012) investigated if an acute high exposure to EMF could have possible adverse effects on male reproductive health. Twenty-four healthy male volunteers participated in a balanced cross-over study with exposure using a head scan in real MRI with whole-body transmitting coil and one set up for sham MRI exposure. Serum-blood samples of inhibin B, testosterone, prolactine, thyreotropine, luteinizing hormone, follicle stimulating hormone, sex-hormone binding globuline and estradiol were taken before and after the different scans. Neither immediately after, nor after 11 days were any differences observed in the hormone levels comparing real and sham MRI. The lack of effects of EMF on male reproductive hormones should be reassuring to the public and especially for men examined in MRI. Adverse effects on other endpoints than male reproduction or possible chronic effect of multiple MRI scans were not investigated in this study.

There is some evidence on genotoxic effects in patients undergoing MRI examination. Simi et al. (2008) studied the level of micronucleated lymphocytes in cultured lymphocytes of eight subjects before and after a cardiac MRI (CMR). Energy absorbed by the subjects was calculated to range from 19 to 306 J. An increase in micronucleus frequency, measured by the cytokinesis block method, was reported in lymphocyte

cultures established immediately after the MRI in all individuals, with a 2-fold increase in mean micronucleus frequency in comparison with the samples collected before the examination. A statistically significant increase in micronuclei was still seen in samples obtained 24 h after the scan but not after 48 h, 72 h, 90 h or 120 h.

Fiechter et al. (2013) studied genotoxic effects in 20 prospectively enrolled patients who underwent 1.5 T CMR. A commercially available MR scanner equipped with a maximum gradient strength of 42 mT/m and a maximum gradient speed of 180 mT/m/ms was used and the mean CMR scan duration was 68 + 22 min with an average contrast media bolus of 15 + 4 ml. Peripheral mononuclear cells were studied for DNA damage using immunofluorescence microscopy of foci positive for phosphorylated gamma-H2AX in nuclear DNA, indicative of sites of DNA double strand break repair. The median and mean numbers of foci per mononuclear cell were, respectively, 0.066 and 0.143 in baseline samples (collected prior CMR scan) and 0.190 and 0.270 after the CMR scan; the difference (1.6-fold for median, 1.9-fold for mean) was statistically significant ($P<0.05$). In addition, gamma-H2AX-positive foci were quantified in CD3-positive T-lymphocytes by flow cytometry. The analysis revealed a statistically significant increase in geometric mean of fluorescence intensity (arbitrary units) of T-lymphocytes after the MRI (median 3232, 1.17-fold; mean 3395, 1.14-fold) as compared with samples collected before the scan (2758; mean 2989), which was statistically significant.

Two studies were carried out on lymphocyte cultures from healthy donors exposed *in vitro*. Lee and co-workers investigated the induction of genotoxic effects in human peripheral blood lymphocytes from one donor exposed from 22 to 89 min to a 3 T MRI scanner. An increase in the frequency of chromosomal aberration (CA) and micronuclei (MN) and in the extent of DNA migration (comet assay) was detected, although it resulted time-dependent in the case of CA and MN (Lee JW et al., 2011). Similar experimental conditions were applied by Szerencsi and co-workers, and different results were obtained. In particular, peripheral blood samples from three healthy donors were exposed to electromagnetic fields produced by 3T magnetic resonance imaging equipment for 0, 22, 45, 67, and 89 min during the scanning procedure. To evaluate DNA damage, blood samples from each donor were processed to apply the alkaline comet assay and the micronucleus assay. No effects were detected in exposed cultures while in positive controls, exposed to 4 Gy gamma rays, a significant increase in the comet parameters and in MN frequency was induced, as expected (Szerencsi et al., 2014).

3.10.2. *Combined exposure to RF*

Since the recent development and use of mobile electronic devices employ different frequencies of RF signals, humans are simultaneously exposed to more than one signal. A scanty number of papers is available on this topic and most of them are by a research group from the Korea Institute of Radiological and Medical Science (Seoul, Korea).

Most of the *in vivo* investigations have been carried out on rodents, and are summarized in table 18.

Lee et al (2009) evaluated teratogenicity in ICR mouse foetuses by exposing pregnant mice to combined CDMA and WCDMA signals at SAR of 4 W/kg (2 W/kg for each signal). Mice received two 45 minutes exposures separated by 15 min intervals daily through the entire gestational period. Animals were killed on the 18th day of gestation and foetuses were examined for mortality, growth retardation, changes in head size and other morphological abnormalities. No observable adverse effects on mouse foetuses were detected for all the experimental conditions adopted (Lee et al., 2009).

In subsequent studies, animals were simultaneously exposed to CDMA and WCDMA RF signals at SAR of 4 W/kg (2 W/kg for each signal). The exposure was 45 min per day and the total exposure duration varied on the basis of the endpoint investigated. In particular, testicular function was examined in male SD rats exposed for a total of 12 weeks. No differences between-RF exposed and sham-exposed animals were detected in sperm count, blood serum testosterone concentration, malondialdehyde concentration in

testis and epididymis, frequency of spermatogenesis stages and appearance of apoptotic cells in the testis. Moreover, apoptosis-related proteins in the testes (p53, bcl2, cyclin G1 and GADD45) also resulted unaffected by the RF exposure. Therefore, the authors concluded that simultaneous exposures had no effects on the rat reproductive system (Lee et al., 2012a). Lack of effects was also found on immunofunctions of male Sprague-Dawley rats exposed for up to 8 weeks, evaluated as subtype population of splenocytes and cytokine production or mRNA expressions, interleukin (IL)-6, tumour necrosis factor (TNF)-α, IL-1β, interferon (IFN)-γ and transforming growth factor (TGF)-β from the splenocytes or IL-6, TNF-α, and immunoglobulin (Ig) of IgG and IgM from blood serum (Jin et al., 2012a).

The authors also evaluated lymphoma development in AKR-mice, a suitable model of lymphoma, exposed for 42 weeks in the same experimental conditions reported above. No differences with respect to sham-exposed animals were detected in terms of body mass, lymphoma incidence, lymphoma malignancy or metastasis infiltration to the spleen, lung and liver. However, occurrence of metastasis infiltration to the brain was higher in exposed mice with respect to sham-exposed ones. The authors concluded that, due to the long exposure duration and the high SAR level, the results do not indicate a health hazard for neoplastic development and more advanced experiments are needed to elucidate the observed effect (Lee et al., 2011a).

In another paper, several parameters of the endocrine system were measured in Sprague-Dawley rats exposed up to 8 weeks. In this study the effect of CDMA signal alone was also evaluated (849 MHz, 4W/kg). Animals were divided into two groups and were sacrificed after 4 or 8 weeks of exposure. No alterations of serum levels of melatonin, thyroid stimulating hormone, triiodothyronine, thyroxin, adenocorticotropin and sex hormones (testosterone and estrogen) were detected for all the experimental conditions investigated (Jin et al., 2013).

Only one investigation has been carried out by an independent research group. They exposed adult male Sprague-Dawley rats for 1 hour to 900 MHz (2 W/kg), 2450 MHz (2 W/kg) or both (1 W/kg each; 2 W/kg in total). After 24 h animals were sacrificed. No differences in general cell morphology and apoptosis were recorded respect to negative controls, either after single and simultaneous exposures (Lopez et al., 2012).

Table 18. Combined exposures to RF: *in vivo* studies

Reference	Model	Combined exposure	Results
Lee et al., 2009	ICR pregnant mice	CDMA (837 MHz) + WCDMA (1950 MHz), 2 W/kg each<br>Two 45 min exposure/day through the entire gestational period	No effects on mortality and several morphological abnormalities on mouse foetuses
Lee et al., 2012a	Male S-D rats	CDMA+WCDMA, 2 W/kg each<br>45 min exposure/day for 12 weeks	No effects on reproductive system
Jin et al., 2012a	Male S-D rats	CDMA+WCDMA, 2 W/kg each<br>45 min exposure/day for up to 8 weeks	No effects on immune system
Lee et al., 2011b	AKR mice	CDMA+WCDMA, 2 W/kg each<br>45 min exposure/day for up to 42 weeks	No effects on lymphoma development
Jin et al., 2013	SD-rats	CDMA signal alone, 4 W/kg;<br>CDMA+WCDMA, 2 W/kg each<br>45 min exposure/day for up to 8 weeks	No alterations of several parameters of the endocrine system
Lopez et al., 2012	Male SD-rats	900 MHz, 2 W/kg;<br>2450 MHz, 2 W/kg;<br>900 MHz + 2450 MHz, 1 W/kg each;<br>1 h exposure	No effects on cell morphology and apoptosis

CDMA: Code Division Multiple Access; S-D rats: Sprague-Dawley rats; WCDMS: Wideband Code Division Multiple Access.

Concerning *in vitro* studies, the effect of single or combined exposures was investigated in human carcinoma cell lines in terms of DNA synthesis, cell cycle distribution and cell cycle regulatory proteins. MCF7 cell cultures were exposed either to the code division multiple access (CDMA, 837 MHz) signal alone or simultaneously to CDMA and wideband

CDMA (WCDMA, 1950 MHz) for 1 hour. The SAR was 4 W/kg for CDMA signal exposure alone and 2 W/kg each (4 W/kg in total) for combined CDMA plus WCDMA signals. Neither single nor combined RF radiation had any effect on the endpoints investigated (Lee et al., 2011b). The same research group also evaluated the induction of oxidative stress in human breast epithelial MCF10A cells exposed for two hours in the experimental conditions described above, but in this study the effect of the WCDMA signal alone was also tested. No statistically significant differences were found in the levels of ROS, in the antioxidant enzyme activity of superoxide dismutase and in the ratio of reduced/oxidized glutathione when exposed cultures were compared to sham-exposed ones (Hong et al., 2012). In another study the authors investigated the effect of longer exposure duration on the expression level and phosphorylation states of specific heat shock proteins (HSP90, HSP70, HSP60, HSP40) and mitogen-activated protein kinases (MAPKs). MCF10A cell cultures were exposed for four hours or for two hours on three consecutive days to CDMA signal alone (4 W/kg) or in combination with WCDMA (2 W/kg for each signal). Again, no significant differences were detected between RF exposed and sham-exposed samples (Kim et al., 2012). In a more recent study, the authors exposed three different neuronal cells such as U87, PC12 or SH-SY5Y to combined RF radiation (837 MHz CDMA plus 1950 MHz WCDMA) for 2 h at 2 W/kg SAR level to investigate the effects on intracellular reactive oxygen species (ROS) at different time points (1, 3, 6 and 12 h) after exposure. Results indicated absence of effects in all the cell types for all conditions tested, except for 6 h post exposure in U87 cells and 12 h post exposure in PC12 cells, in which a slight but statistically significant effect was detected. Measurement of ROS levels in neuronal cells after 2 h co-exposure to multiple RF signals and 100 μM H_2O_2 were also carried out at 1, 3, 6 and 12 h post exposure and no significant changes were detected. Absence of cooperative effects was also detected when co-exposures were carried out with 100 and 200 μM menadione and ROS analysed after 0.5, 1 and 3 h. (Kang et al., 2014).

The results of *in vitro* investigations are summarized in table 19.

Table 19. Combined exposures to RF: *in vitro* studies

Reference	Cell type	Combined exposure	Results
Lee et al., 2011b	Human breast cancer cells (MCF7)	CDMA (837 MHz), 4 W/kg; CDMA + WCDMA (1950 MHz), 2 W/kg each<br>1 h exposure	No effects on DNA synthesis, cell cycle distribution and cell cycle regulatory proteins
Hong et al., 2012	Human breast epithelial cells (MCF10A)	CDMA, 4 W/kg;<br>WCDMA, 4 W/kg;<br>CDMA + WCDMA, 2 W/kg each<br>2 h exposure	No induction of oxidative stress (ROS formation, SOD activity and GSH depletion)
Kim et al., 2012	Human breast epithelial cells (MCF10A)	CDMA, 4 W/kg;<br>CDMA + WCDMA, 2 W/kg each<br>4 h exposure or 2 h on three consecutive days	No variation in the expression level of HSPs and MAPKs
Kang et al., 2014	Neuronal cells (U87, PC12, SH-SY5Y)	CDMA (837 MHz) + WCDMA (1950 MHz), 2 W/kg each<br>2 h exposure	ROS formation evaluated 1, 3, 6 and 12 h after RF exposure.<br>ROS increase in U87 cells at 6 h and in PC12 cells at 12 h.<br>No effect in the other conditions.<br>No effect in cultures co-exposed to H2O2 or menadione

CDMA: Code Division Multiple Access; GSH: Reduced Glutathione; HSP: Heat shock proteins; MAPK: mitogen-activated protein kinase; ROS: reactive oxygen species; SOD: Superoxide dismutase; WCDMS: Wideband Code Division Multiple Access.

3.10.3. *Combined exposures to different EMFs*

Novikov and co-workers evaluated the effects of combined exposures to ELF and SMFs on BALB/c mice. The animals were intraperitoneally transplanted with Ehrlich ascites carcinoma (EAC) cells and then exposed one hour/day for 12 days to a combination of SMF (DC; 42 μT) and alternating MF (AC; 1, 4.4 and 16.5 Hz). For each frequency, several series of experiments have been performed with intensities ranging from 40 to

500 nT. Moreover, other experiments have been carried out at 16.5 Hz carrier frequency in the presence of a modulating frequency of 0.5 Hz. For each of the AC components the optimal intensity for survival of animals was adopted to perform a combined exposure (1 Hz, 300 nT; 4.4 Hz, 100 nT; 16.5 Hz, 150 nT). The results obtained showed that in the combined exposure the antitumor activity was higher than in the single frequency exposures. In animals without tumours no pathological deviation from the norm was detected, indicating lack of intrinsic toxicity of the combined exposures (Novikov et al., 2009).

Conclusions on health effects from combined exposures to different EMFs

The few available studies on combined exposure to different EMFs do not provide sufficient evidence for risk assessment.

The studies reporting on effects on DNA integrity after an MRI investigation are clearly of interest to follow up. However, it is not clear which component of the complex EMF exposure during scanning may cause the effect: SMF, switched gradient MF or the pulsed RF EMF. Further studies on DNA integrity and MRI exposure are needed, and the feasibility of cohort studies of MRI patients and occupationally exposed personnel should be discussed.

3.11. Health effects from co-exposure to environemtal stressors

3.11.1. Animal studies

What was already known?

In the previous Opinion of 2009 (SCENIHR 2009), the few studies available in the literature suggest that co-exposures with ELF fields may be co-carcinogenic, while no evidence was achieved in the case of RF fields.

What has been achieved since then?

ELF fields

Two co-carcinogenesis investigations have been carried out. Jimenez-Garcia et al. 2010 concurrently exposed Male Fischer-344 rats to 120 Hz, 4.5 mT, 50 min/d for 32 days and to N-Diethylnitrosamine (DEN) and 2-acetylaminofluorene (2AAF), two hepatocarcinogenesis-inducers. After 7 days from the start of co-exposure an inhibition of pre-neoplastic lesion induced by the chemical treatments was detected. In particular, a reduction in cell proliferation (decreased expression of Ki-67 and cyclin D1 proteins) was reported, not associated with apoptosis. However, this interesting result has been obtained on a small number of animals for each treated group (6) (Jimenez-Garcia et al., 2010). In contrast, no differences in 7, 12-dimethylbenz(a)anthracene (DMBA)-induced hematopoietic neoplasia was reported by Negishi et al. They co-exposed CD-1 mice to a 50 Hz magnetic field (7, 70 or 350 μT field intensity) for 22 h/d, 7 days/week for 30 weeks (Negishi et al., 2008).

Rajkovic et al. reported cooperative effects of MF (50 Hz, 100 and 300 μT 4h daily exposure) and the pesticide atrazine on male Wistar rats. They found an increased number of degranulated mast cells for all the co-exposure protocols applied, compared to atrazine treatment alone. It should be pointed out that the exposure duration is not clearly mentioned (Rajkovic et al., 2010). Wang and co-workers also reported cooperative effects of ELF fields and chemical treatments. They exposed Sprague-Dawley rats to a 20 Hz MF (14 mT) 1 h/day for 12 days and, after MF exposure morphine was administered. They found a decreased density of dopamine receptors upon morphine withdrawal respect to morphine treatment alone. The effect of combined treatment tended to normalize as morphine withdrawal days increased (Wang et al., 2008). Celik and co-workers (2013) employed Sprague Dawley rats to investigate the effect of ELF field exposure on the accumulation of manganese in various rat tissues. Manganese at

different doses was administered every two days for 45 days, while ELF exposure (50 Hz, 1.5 mT) was carried for 4 h/day, for 5 days/week during the same period of 9 weeks. Manganese treatment increased accumulation levels in kidney, liver and brain with respect to control rats. Moreover, ELF exposure increased this accumulation.

Some studies have tried to reveal subtle behavioural consequences of exposure to magnetic fields through their interactions with drugs or interventions that cause known biological effects. Canseven et al. (2007) investigated the effects of magnetic fields on drug-induced seizure activity in female Swiss albino mice. Seizures were induced in female mice by injection of pentylenetetrazole at a sub maximal dose (60 mg kg^{-1} in 0.1 ml saline); this dose induced a grand-mal seizure within a few minutes. Exposure to a 50 Hz magnetic field at 0.2 mT had no significant effects on seizure latency or duration, or on mortality. Animals were exposed using a pair of Helmholtz coils for either 1 h before and 30 min after injection, 1 h before and 30 min sham exposure after injection, or 1 h sham exposure before injection and 30 min of exposure after injection The coils used were not shielded against electric fields, but the measured electric fields were negligible.

Gulturk et al. (2010) investigated the effect of long-term exposure to magnetic fields on the permeability of the blood-brain barrier (BBB) in the streptozotocin (STZ)-induced diabetic rat model. Male Wistar rats were exposed within a solenoid that was producing a 50 Hz field at 5 mT, for 30 min on /15 min off, for 165 min/day for 30 days; sham exposed animals were placed within the solenoid without the field being generated. The magnitudes of any noise, vibration or heat from the solenoid when energised were not described. BBB permeability was assessed using Evans Blue extravasation. It was found that BBB permeability was significantly increased by treatment with STZ or magnetic field alone (and by the same amount), and in combination they caused an even greater increase in permeability; daily injection with insulin reduced these effects, although permeability remained well above values for sham exposed animals. STZ reduced weight gain but exposure to magnetic fields had no effect alone and no additive effects with STZ. Compared to their own baseline values, STZ significantly increased blood glucose levels four-fold, whereas magnetic fields caused a small but significant decrease, and together the resultant increase in blood pressure was significantly less than that caused by STZ alone, but still around a three-fold increase over baseline, and was similar to the effects of treatment of these animals with insulin. Finally, STZ increased mean arterial blood pressure, but the magnetic field had no significant effect either alone or in combination with STZ. Overall, the authors concluded that exposure to magnetic fields increases the vulnerability of the BBB in diabetes, but treatment with insulin reversed this sensitivity.

Lei et al. (2013) also employed the STZ-induced diabetic rat model to study the effects of pulsed magnetic fields in relieving behavioural signs of diabetic peripheral neuropathy (DNP). Male Sprague-Dawley rats were exposed to pulsed 15 Hz fields for 8 /day, 6 days/week for 7 weeks using a modified Helmholtz coil exposure system (consisting of 3 coils of 800 mm diameter, spaced 304 mm apart) connected to a pulsed generator producing a train of 0.2 ms pulses for 5 ms every 60 ms. The peak intensity of the field was 1.6 mT. Compared to sham exposed animals with induced diabetes, exposure significantly increased the thresholds for withdrawal of the hind paws to tactile stimuli (mechanical allodynia) and to noxious heat stimuli (thermal hyperalgesia). Exposure also attenuated the development of neuropathogical changes associated with diabetes. It was suggested that pulsed fields might prove an effective therapy for the treatment of DNP in humans.

Rauš et al. (2012) reported that magnetic fields inhibited hyperactivity induced by transient global cerebral ischemia. Following surgical occlusion of both common carotid arteries for 10 min, adult Mongolian gerbils were exposed continuously to a gradient 50 Hz field from an electromagnet for 7 days; animals were housed 20 cm from the electromagnet, so that the field in the centre of the cages was 0.5 mT, with a range of 0.2 – 2 mT; the exposure of each animal would be uncontrolled and depend on their location in the cage. The behaviour of the animals in an open field arena was analysed

using a video tracking system for 60 min on four days during exposure and on day 7 after exposure. It was found that transient ischemia induced significant increases in distance moved, stereotypic head movements and body rotations for the first 4 days. However, exposure to the magnetic field significantly reduced these effects for the first 2 days, and thereafter the reductions were not significant. The authors speculated that the magnetic field may have produced the changes in activity through an influence on the opioid system. Rauš et al. (2013) examined the histology of the hippocampus of these animals and found that exposure to the magnetic field helped to overcome the damage caused by ischemia. It was reported that ischemia significantly affected cell morphology and increased cell death, magnetic field exposure alone caused no consistent effects, but exposure combined with ischemia resulted in a reduction in numbers of dying neurons (at 7 days after termination of field exposure) compared to ischemia alone, and increased numbers of GFAP-labelled astrocytes and microglial cells (immediately after exposure). A companion study (Rauš Balind et al., 2014) suggested that increases in oxidative stress could be transiently induced in the hippocampus, striatum or cortex by either global cerebral ischemia or by exposure to the magnetic field, but oxidative stress was significantly reduced following combined ischemia and magnetic field treatment. In this study, the activities of superoxide dismutase and malondialdehyde and production of nitric oxide and superoxide were measured at the end of field exposure (to assess immediate effects) or a further 7 days later (for delayed effects). Magnetic fields alone had a significantly greater effect than ischemia alone, and why combined treatment should reduce the magnitude of the immediate effects is not obvious. Generally, delayed effects were much reduced for all treatments except ischemia alone, with most measured parameters returning to control levels. However, a single control value for each of the measured parameters was provided at both assessment times without any indication of variability.

Zhang et al. (2013) found that exposure to magnetic fields had no influence on the pathogenesis of Alzheimer's disease-like changes induced by chronic aluminium (Al) treatment. Adult, male Sprague-Dawley rats were exposed to either 50 Hz magnetic fields at 100 μT using a pair of Helmholtz coils, given Al overload (AlCl3 solution, 2g/l, in their drinking water) or both treatments for 12 weeks. Compared to unexposed and untreated control animals, Al increased escape latency and total distance swum in a water maze during acquisition trials and impaired retention of the task during the probe trial; exposure to 50 Hz fields had no effect on performance; and combined exposure did not have an increased effect compared to Al alone. Al treatment with or without magnetic fields significantly increased levels of Al in the cortex, and increased Aβ concentration and neuronal losses in the cortex and hippocampus: magnetic fields alone did not affect these outcomes. Al significantly reduced water intake and body weight gain during the study period, possibly due to differences in palatability of the AlCl3 solution.

Deng et al. (2013) investigated whether memory impairments produced in mice by chronic Al treatment could be modulated by magnetic field exposure. It was found that water maze performance was impaired in Kunming mice that had been treated for 8 weeks with either 50 Hz fields at 2 mT for 4 h/day, 6 days/week, given daily Al loading (200 mg/kg) or given both magnetic fields and Al. In addition, in all three experimental groups there was neuronal cell loss and overexpression of phosphorylated tau protein in the hippocampus and cerebral cortex, plus superoxide dismutase activity was decreased and malondialdehyde was increased. It was concluded that there was no evidence that combined exposure had any caused any synergistic effect, although both Al loading and magnetic fields could produce significant effects on their own.

The results are summarized in Table 20.

Table 20. ***In vivo*** **studies on ELF & co-exposures**

Reference	MODEL	MF exposure	Co-exposure	Results
Jiménez-García et al., 2010	Male Fischer-344 rats	120 Hz, 4.5 mT 50 min/d for 32 dd	Hepatocarcinogenesis-inducers DEN and 2AAF after 7 dd from the start of MF exposure (concurrent)	Inhibition of pre-neoplastic lesion development induced by the hepatocarcinogenesis experimental protocol; reduction in cell proliferation (decreased expression of Ki-67 and cyclin D1 proteins); no induction of apoptosis
Negishi et al., 2008	CD-1 mice	50 Hz, 7, 70 or 350 µT 22h/d, 7dd/w; 30 ws	lymphoma/leukaemia inducer DMBA	No differences in DMBA-induced hematopoietic neoplasia
Rajkovic et al., 2010	Male Wistar rats	50 Hz, 100 and 300 µT 4 h daily exposure	Atrazine, 20 or 200 mg/kg bw	Increased number of degranulated mast cells for all the co-exposure protocols applied respect to atrazine treatment alone
Wang et al., 2008	Sprague–Dawley rats	20 Hz, 14 mT 1 h/d for 12 dd	Morphine (after MF exposure)	Decreased density of dopamine D2 receptors upon morphine withdrawal with respect to morphine treatment alone. The effect of combined treatment tended to normalize as morphine withdrawal days increased
Celik et al., 2013	Sprague–Dawley rats	50 Hz, 1.5 mT 4 h/d for 5 days/week for 9 weeks	Manganese every two days/9 weeks (concurrent)	ELF-exposure increased manganese accumulation in kidney, liver and brain
Canseven et al., 2007	Female Swiss albino mice	50 Hz, 0.2 mT 0.5, 1, 1.5 h	Pentylenetetrazole (before, concurrent, after MF)	No effect on seizure latency or duration or on mortality
Gulturk et al., 2010	Male Wistar rats	50 Hz, 5 mT 165 min/d for 30 dd (30 min on/15 min off)	STZ	Increased BBB permeability by STZ alone and MF alone. Greater increase by co-exposure No effect of co-exposure in STZ-reduced body weight ; reduction of STZ-induced glucose levels and blood pressure
Lei et al., 2013	Male Sprague–Dawley rats	15 Hz pulsed (0.2 ms pulses for 5 ms every 60 ms)	STZ	Increased threshold for mechanical and thermal sensitivity
Raus et al., 2012; 2013; 2014	Mongolia n gerbils	50 Hz, 0.5 mT (0.2-2 mT) 7 days	Surgical induced ischemia	Transient reduction of ischemia-induced increase in body movements; reduced number of dying neurons and increased number of astrocytes and microglial cells. Reduction of ischemia-induced oxidative stress.
Zhang et al., 2013	Male Sprague–Dawley rats	50 Hz, 0.1 mT 12 weeks	AlCl3 (before or concurrent)	No effects on behaviour (water maze)
Deng et al., 2013	Kunmig mice	50 Hz, 2 mT 4 h/day for 6 days for 8 weeks	AlCl3 (before or concurrent)	No effect on memory impairment induced by Al or MF treatment. No effect of co-exposure on Al and MF-induced neuronal cell loss; overexpression of phosphorylate tau protein and oxidative stress.

2AAF: 2-acetylaminofluorene; ; Al: aluminium; BBB: Blood Brain Barrier; bw: body weight; DEN: N-Diethylnitrosamine; DMBA: 7,12-dimethylbenz(a)anthracene, STZ: Streptozotocin.

RF fields

For RF fields two co-carcinogenesis investigations have also been carried out. Tillmann et al. exposed female B6C3F1 mice to 1966 MHz, UMTS signal, 4.8 W/m^2 20h/day from gestational day 6 up to 24 months and to n-ethylnitrosourea (ENU) on gestational day 14. They found increased malignancy and multiplicity of lung carcinomas in co-exposed animals respect to animals exposed to ENU alone (Tillmann et al., 2010).

In a second investigation, carried out by Paulraj and Behari, no effects of co-exposures were detected. They exposed Swiss albino mice to 112 MHz modulated at 16 Hz, 0.1 W/m^2 or at 2450 MHz, 0.034 W/m^2 (calculated SAR 0.75 W/kg and 0.1 W/kg, respectively). Two co-exposure protocols were applied: a) exposure of 2 h/day, 3 days/week for 16 weeks and treatments with DMBA, and b) 14 days exposure and intraperitoneal injection of ascites carcinoma cells. In all cases RF was given after treatments. For all the experimental conditions tested the authors reported no increase in tumour growth and development respect to carcinogenic treatments alone (Paulraj and Behari, 2011).

Behavioural and neurochemical deficits were the focus of a study by Maaroufi and co-workers (2014). They exposed Wistar rats to 900 MHz EMFs in absence and in presence of ferrous sulfate to test the hypothesis of a possible link between iron overload in the brain and neurodegenerative disorders. Rats were exposed 1h/day during 21 consecutive days at SAR of 0.05 and 0.18 W/kg, depending on the position of the rat in the exposure chamber, and, in the case of co-exposures, were concurrently daily subjected to injection of ferrous sulfate. Rats exposed to RF field resulted impaired in the exploratory activity but not in the navigation and working memory tasks. Alteration in monoamine content was also detected mainly in the hippocampus area. Combined treatments did not potentiate behavioural and neurochemical deficits with respect to EMF alone exposed rats.

In several investigations a protective effect of RF pre-exposure against exposure to ionizing radiation was reported. Four papers have been published by Dr. Cao's research group. In a first study male Kunming mice were exposed to 900 MHz (1.2 W/m^2) 1h/day for 14 days and then treated with 5 Gy gamma-rays. Less severe hematopoietic pathological alterations (cell reduction, hematopoietic tissue volume, decreased edema) were detected in co-exposed animals respect to those exposed to gamma ray alone (Cao et al., 2010). In a second investigation the authors pre-exposed male Kunming mice to 900 MHz, 0.12, 1.2 and 12 W/m^2 (calculated SARs 0.00548, 0.0548 and 0.548 W/kg) 1 h/day for 14 days and then the animals were treated with gamma-rays (8 or 5 Gy). A significant increase in survival time (8 Gy) and a significant reduction in hematopoietic tissue damage (5 Gy) was detected (Cao et al., 2011). In a third study pre-exposure of male ICR mice to RF in the same experimental condition but 4h/day for 1, 3, 5, 7 or 14 days, followed by 3Gy gamma-rays, resulted in a decreased DNA migration (comet assay) respect to mice exposed to gamma-rays alone, except for RF exposure of 1 day (Jiang et al., 2012). In a more recent investigation, the authors pre-exposed adult male ICR mice to 900 MHz at an SAR of 0.548 W/kg for 4h/day for 7 days and then the animals were subjected to an acute whole body dose of 3 Gy gamma rays to investigate the chromosomal damage in peripheral blood (PB) and bone marrow (BM) tissues. Combined treatments resulted in a significant decrease in micronucleus indices compared to gamma rays alone, in both PB and BM tissues (Jiang et al., 2013).

The authors suggested an adaptive response induced by pre-exposure to RF field. The four studies were carried out with the same exposure system, as reported in Cao et al., 2011 and Jiang et al., 2012 and 2013.

The results described above are summarized in Table 21.

Table 21. ***In vivo* studies on RF & co-exposures**

Reference	MODEL	MF exposure	Co-exposure	Results
Tillmann et al., 2010	female B6C3F1 mice	1966 MHz, UMTS<br>4.8 or 48 W/m^2 (peak SAR calculated 5 W/kg)<br>20 h/d from gestational day 6 up to 24 months	ENU on gestational day 14 in animals exposed to 4.8 W/m^2	Increased malignancy and multiplicity of lung carcinomas in animals exposed to both ENU and RF.<br>No effect of RF exposure alone.
Paulraj and Behari, 2011	Swiss albino mice	112 MHz modulated at 16 Hz 0.1W/m^2 (0.75 W/kg);<br>2450 MHz, 0.034 W/m^2 (0.1 W/kg)<br>Protocol A: 2 h/d, 3 dd/week for 16 weeks<br>Protocol B: 14 days	Protocol A: 7,12-DMBA<br>Protocol B: ascites carcinoma cells<br>Chemicals given before RF	No increase in tumour growth and development respect to carcinogenic treatments alone
Maarofui et al., 2014	Wistar rats	900 MHz<br>0.05 or 0.18 W/kg<br>1h/d, 21 days	Ferrous sulfate (concurrent)	Impairment in explanatory activity after RF exposure. No effect on navigation and working memory<br>No effects of combined treatments
Cao et al., 2010	male Kunming mice	900 MHz, CW<br>1.2 W/m2<br>1 h/d for 14 dd	gamma-rays (5 Gy) after 14 dd RF	Less severe hematopoietic pathological alterations (cell reduction, hematopoietic tissue volume, decreased edema) in co-exposed animals respect to those exposed to gamma ray alone.
Cao et al., 2011	male Kunming mice	900 MHz, CW<br>0.12, 1.2 and 12 W/m2 (SARs 0.00548, 0.0548 and 0.548 W/kg)<br>1 h/day for 14 days	gamma-rays (8 or 5 Gy) after RF	Significant increase in survival time (8 Gy) and significant reduction in hematopoietic tissue damage (5 Gy)
Jiang et al., 2012	male ICR mice	900 MHz, CW<br>0.12 W/m2 (calculated SAR 0.548 W/kg)<br>4 h/d for 1, 3, 5, 7 and 14 days	gamma-rays (3 Gy) after RF	Decreased DNA migration (comet assay) in mice pre-exposed to RF for 3, 5, 7 and 14 days respect to mice exposed to gamma rays alone.
Jiang et al., 2013	male ICR mice	900 MHz, CW<br>0.548 W/kg for 4h/d for 7 days	gamma-rays (3 Gy) after RF	Decreased MN frequency in mice pre-exposed to RF respect to mice exposed to gamma rays alone

CW: continuous wave; DMBA: dimethylbenzen(a)anthracene; ENU*:* n-ethylnitrosourea; MN:micronucleus

Discussion and conclusions on *in vivo* studies

From the results reported above it seems that exposure to ELF or RF interacts with several chemical or physical agents by exhibiting an increase or a decrease in the effects of the latter. Nevertheless, due to the small number of investigations available and the large variety of protocols adopted (different chemical or physical treatments and different EMF exposure conditions), it is not possible to draw concrete conclusions. Further investigations should be carried out to clarify the role of EMFs in increasing/decreasing the effect of other treatments.

3.11.2. *In vitro* studies

What was already known?

In the previous Opinion, the studies on cooperative effects of ELF fields resulted all positive: the co-exposure induced enhancement or decrease of the effect induced by chemical or physical agents. Co-exposures with RF fields were also reported, but the results were conflicting.

What has been achieved since then?

A large number of *in vitro* investigations have been carried out on a variety of biological targets and by applying different co-exposure protocols.

Static Fields – Eight papers have been devoted to investigate the combined effects of SMF and chemical or physical agents, as reported in Table 22. In six studies the results indicated an enhancement of the effects induced by chemical/physical treatment alone. Moreover, in two studies an increase or a decrease was recorded, on the basis of the experimental conditions investigated.

The research group of Professor Qi reported an increased killing effect of several drugs currently used for chemotherapy when human leukemic cells K562 were concurrently exposed to a SMF of 8.8 mT. In particular, Chen et al. detected an increased cell membrane permeability after 12 h exposure; moreover, co-exposure with Cisplatin (DDP) induced a more pronounced decrease in cell proliferation and an arrest at the S phase of the cell cycle, together with an altered DMA migration pattern (alkaline comet assay) respect to DDP treatment alone. The extent of the effects resulted dependent on the DDP dose used for combined exposures. The authors suggested that SMF is able to alter the cell surface ultrastructure (Chen et al., 2010). Similar results were obtained when co-exposures were carried out with Adriamycin (Qi et al., 2011). In a third investigation the authors confirmed that cell killing induced by different anticancer drugs was enhanced by co-exposures. The effect of SMF combined with taxol or cyclophosphamide resulted additive, while it was synergistic with DPP or doxorubicin (Liu et al., 2011).

Poniedzialek et al. (2013) evaluated the effect of gradient static magnetic field (SMF) on ROS production in peripheral blood human neutrophils *in vitro*. Blood samples were exposed in an inhomogeneous SMF (in a south or north pole of the field) for 15, 30 or 45 minutes. The maximum value of magnetic flux density (B_{max}) amounted to <60 mT. Phorbol 12-myristate 13-acetate (PMA) was employed as respiratory burst stimulator. A statistically significant change in ROS production was induced in unstimulated and PMA-stimulated neutrophils and the effects were highly correlated with the exposure time and depended on the orientation of the field. In particular, 15 min exposure induced a decrease, while 45 min resulted in an increase in ROS formation. In this study no sham-exposed samples were set up and the results of exposed cultures were compared to control cultures (incubator). Moreover, the number of donors involved in the study is not reported.

Concerning combined treatments with physical agents, human peripheral blood leukocytes were exposed from 0.5 to 24 h to inhomogeneous (0.3, 1.2, 47.7 T/m) or homogeneous (159.2 ± 13.4 mT) SMF, given alone or with gamma rays (4 Gy). Several co-exposure schedules were applied (SMF before or after ɣ-rays). The results showed an increase in DNA migration (comet assay) as a function of the SMF characteristics either when SMF was given alone and after gamma irradiation. No cooperative effects were found if SMF preceded ɣ irradiation (Kubinyi et al., 2010). On the contrary, Sarvestani et al. reported enhancement of X-ray induced arrest in G2/M phase of the cell cycle in rat bone marrow stem (BMSC) cells with SMF (15 mT for 5h) provided after 0.5 Gy X-ray, although co-exposures with SMF before X-ray have not been performed. In this case no effects of SMF alone were detected (Sarvestani et al., 2010).

In an investigation carried out by Feng and co-workers (2013) human A549 lung adenocarcinoma cells were exposed to a 0.5 T MF, alone or in combination with ionizing radiation (4 Gy x rays given after MF exposure). Cell growth inhibition and up- or down-regulation of genes involved in cell cycle and apoptosis resulted increase in cells exposed to MF and x rays, compared to cells exposed to x rays alone. In this study, no information is given on the employed MF exposure system. Politanski et al. (2013) also investigated the effect of static magnetic fields given in combination with x rays. They evaluated ROS formation in lymphocytes from male albino Wistar rats. The SMF exposure was carried out at 0 mT (50 µT magnetic field induction opposite to the geomagnetic field) and 5 mT by placing cell cultures inside a pair of Helmholtz coils, which provided a highly homogenous field. ROS formation was measured after 15 min, 1 and 2 h. The

results indicated that a significant increase in ROS formation was induced in cultures co-exposed to X rays and 5 mT compared to cultures exposed to x ray alone, while a reduction in ROS formation was recorded in samples co-exposed to x ray and 0mT, with respect to samples exposed to x ray alone. In this study sham-exposures were not performed.

Table 22. *In vitro* studies on co-exposures to SMF

Reference	Cell type	SMF exposure conditions	Results
Chen et al., 2010	Human leukemic cells (K562)	8.8 mT<br>12 h with or w/o DDP<br>(concurrent exposures)	Changes in cell surface ultrastructure (cell membrane permeability); no effect on DNA migration (comet); combined exposures enhances the killing effect of DDP and DNA damage as a function of DDP concentration.
Qi et al., 2011	Human leukemic cells (K562)	8.8 mT<br>12 h with or w/o ADM	No effects of SMF or ADM on metabolic activity when given alone. Combined treatments resulted in inhibition of metabolic activity, DNA damage and arrest of the cell cycle.
Liu et al., 2011	Human leukemic cells (K562)	9 mT<br>12-24 h with or w/o Taxol, Doxorubicin, DDP and cyclophosphamide<br>Concurrent exposures	Changes in cell surface ultrastructure;<br>combined exposures enhances the killing effect of drugs as a function of the experimental protocol (exposure duration, drug concentration).
Poniedzialek et al., 2013	Human peripheral blood neutrophils	Inhomogeneous SMF, Bmax <60 mT<br>15-45 min with or w/o PMA.	Decrease in ROS formation after 15 min exposure; increase in ROS formation after 45 min exposure.
Kubinyi et al., 2010	Human peripheral blood leukocytes	Inhomogeneous SMF 0.3, 1.2, 47.7 T/m<br>Homogeneous SMF 159.2 ± 13.4 mT<br>0.5 min – 24 h with or w/o ɣ-radiation given before or after MF.	Increase in DNA migration (comet) as a function of the experimental protocol when SMF was given alone or after ɣ-radiation. No effects for SMF given before ɣ-radiation.
Sarvestani et al., 2010	Rat bone marrow stem cells	15 mT<br>5 h<br>X-ray before SMF	No effect of SMF alone on cell cycle progression. Enhancement of X-ray arrest in G2/M phase.
Feng et al., 2013	Human lung adenocarcinoma cells (A549)	0.5 T<br>1-4 h<br>4 Gy x rays after SMF	Increase in x ray-induced cell growth inhibition and up- or down-regulation of genes involved in cell cycle and apoptosis.
Politanski et al., 2013	Albino Wistar rat lymphocytes	0 mT (50 µT MF opposite to the geomagnetic field) and 5 mT<br>15 min, 1 and 2 h<br>3 Gy x rays after SMF	Increase or decrease in ROS formation in cultures co-exposed at 5 mT and at 0 mT, respectively.

ADM: Adriamycin; DDP: Cisplatin; PMA: Phorbol 12-myristate 13-acetate

ELF fields

Gene expression was investigated by Marcantonio et al., 2010. The authors exposed human neuroblastoma cell line BE(2)C to 50 Hz MF, 1 mT, for 24-72 h in presence or absence of all-trans-retinoic acid (ATRA), a neuronal differentiating agent. Co-exposed cells showed a significant increase of mRNA levels of $p21^{WAF1/CIP1}$ and cdK5 genes, both involved in neural differentiation and a more differentiated morphological traits (a higher

neurite number/cell, and an increased neurite length). They also evaluated the expression of cyp19 gene, involved both in neuronal differentiation and stress response: it resulted enhanced by ATRA treatment and significantly enhanced further by MF-co-exposure. In addition, decreased cell proliferation and increased proportion of cells in G0/G1 stage was also detected following co-exposures. The authors suggested that MF-concurrent treatments of neuroblastoma cells with MF and ATRA can strengthen the effect of ATRA alone (Marcantonio et al., 2010).

Garip and Akan exposed K562 human leukaemia cells concurrently to a 50 Hz MF (1 mT) and H_2O_2. Three hours exposure resulted in a statistically significant increase in the number of apoptotic cells, compared to cells treated with H_2O_2 alone. ROS formation and expression of heat-shock protein 70 (hsp-70) also were enhanced co-exposed cultures, although statistically not significant. Since exposure to MF alone was found to decrease the number of apoptotic cells and to increase the HSP levels and ROS formation, the authors concluded that the effect of MF on biological systems strictly depends on the status of the cell (Garip and Akan, 2010).

Exposure of human hepatoma cells to a 100 Hz MF at 0.7 mT carried out before or after x-ray irradiation also was found to enhance x-ray induced apoptosis, as assessed by Annexin V assay. MF exposure was delivered for two cycles (30 min on/12 h off) with doses of x-ray from 2 to 10 Gy or for six cycles with 2 Gy. The effect became more pronounced if ELF MF exposure was given for six cycles and before the X-ray exposure (Jian et al. 2009).

A time-dependent increase in cell proliferation and in protein oxidation was reported by Eleuteri et al. in human colon adenocarcinoma CaCo 2 cells exposed for 24, 48 and 72 h to a 50 Hz MF (1 mT) in presence of 12-O-tetradecanoylphorbol-13-acetate (TPA), a tumour promoter able to activate protein kinase C, with respect to cells treated with TPA alone (Eleuteri et al., 2009). However, in this paper the authors do not discuss the induced E field, current or the effect due to magnetic field.

Genotoxicity was investigated in five papers. Luukkonen et al. reported that 24 h exposure of human neuroblastoma SHSY5Y cells to a 50 Hz MF (100 µT) immediately followed by 3 h treatment with Menadione resulted in an enhancement of Menadione-induced DNA damage, DNA repair rate and MN formation. The authors found similar results when co-exposures were carried out with methyl-metane sulfonate for 3 h, although the increase was not found to be statistically significant (Luukkonen et al. 2011). In a more recent investigation the same research group observed that human SH-SY5Y neuroblastoma cells exposed to a 50-Hz, 100-µT MF for 24 h showed a slight increase (<2-fold in comparison with sham treated control) in MN measured flow cytometrically 11 and 18 days after the end of the treatment. The results came from a co-treatment experiment where the MF exposure was followed by a 3-h treatment with menadione (at 0, 1, and 20 µM). The effects of MF (and of menadione) were statistically significant in a 3-way ANOVA where MF and menadione were fixed factors and replicate a random factor. Statistics were not provided separately for MF at 0 dose menadione, but MN induction by MF appeared to be similar regardless of menadione. The authors interpreted this effect as induced genomic instability that has previously been described, e.g., for ionizing radiation. Immediately after the exposure, MF was reported to induce ROS (DCFH-DA assay) and superoxide in mitochondria, and to decrease glutathione in ANOVA of the whole series, but the influence of MF alone seemed to be non-existent or low (superoxide) without menadione (which decreased both ROS and glutathione and increased superoxide) and was also low in co-exposure with menadione. Superoxide in cytosol and mitochondrial activity were affected by menadione but not by MF. 8 days after the end of the treatment, MF increased mitochondrial activity without menadione but did not affect the levels of ROS, glutathione, lipid peroxidation, or superoxide. 15 days after the exposure, the only parameters reported to be significantly affected by MF were lipid peroxidation (apparent also without menadione) and ROS production (obvious only at 20 µM menadione). According to the authors, the results suggest that changes in oxidant/antioxidant balance induced by an initial effect of MF can lead to genomic

instability. Even if the effect on MN frequency seemed to be rather small, a prolonged increase in the level of chromosome damage may be expected to have significance with respect to carcinogenesis (Luukkonen et al., 2014).

Cho et al. (2014) employed PHA stimulated human peripheral blood lymphocytes from healthy donors to investigate the effects of ELF-EMF generated by MRI scanner on gadolinium (a contrast agent for enhanced magnetic resonance imaging) toxicity. Genotoxicity (strand breaks and MN induction) and cytotoxicity (cell viability, ROS and apoptosis) were investigated. Exposures/sham exposures (60 Hz, 0.8 mT) of different duration up to 48 h, based on the biological parameter investigated, were carried out concurrently with several doses of gadolinium (0.2-1.2 mM). The results indicated that EMF exposure was able to enhance the gadolinium induced cytotoxicity and genotoxicity.

Different results were reported by Buldak et al.: they exposed AT478 murine carcinoma cells to a 50 Hz MF, 1 mT, for 16 minutes and to cisplatin for 24 h, given concurrently or immediately after MF. A decrease in cisplatin-induced DNA migration was detected in co-exposed cultures, together with a decrease in ROS formation and antioxidant enzyme activities (SOD, GSH-Px) as well as malondialdehyde concentration, compared to treatments with cisplatin alone (Buldak et al., 2012).

Negative results were reported by Jin et al. (2012) who co-exposed mouse fibroblasts or human lung fibroblasts for 4 h to a 60 Hz MF (field intensity of 0.01, 0.5 and 1 mT) and hydrogen peroxide, ionizing radiation or c-Myc activation. In all cases no variation in MN frequency was detected respect to treatments with genotoxic agents alone in both cell types, although no clear information is reported on the co-exposure protocol adopted.

Cellular transformation was evaluated by Lee et al. in NIH3T3 mouse fibroblasts exposed to a 60 Hz MF (1 mT) for 4 h in combination with several stress factors (ionizing radiation, hydrogen peroxide or myelocytomatosis oncogene (c-Myc) activation). No combined effects were detected for all the experimental conditions tested (Lee et al., 2012).

The possibility that MF could modify biological responses to UV radiation by causing an overall change in oxidative reactions was investigated by Markkanen et al. Murine L929 fibroblasts were exposed to 50 Hz MF of 100 or 300 μT during 1 h UV exposure (240 J/m^2) or for 24 h before it. No significant effects of MF on oxidative reactions were detected, as assessed by measuring ultra weak chemiluminescence. The authors concluded that in the experimental conditions tested, MF is not able to modify the biological response of UV radiation (Markkanen et al., 2010).

The results reported above are summarized in Table 23.

Table 23. ***In vitro* studies on ELF & co-exposures**

Reference	Cell type	MF exposure	Co-exposure	Combined effects
Marcantonio et al., 2010	Human neuroblastoma cell line (BE(2)C)	50 Hz, 1 mT<br>24-72 h	Neuronal differentiating agent ATRA<br>(concurrent)	Decreased cell proliferation and increased proportion of cells in G0/G1 phase;<br>More differentiated morphological traits and increase in expression of genes involved in differentiation and stress response
Garip and Akan, 2010	Human leukaemia cells (K562)	50 Hz, 1 mT<br>3 h	H_2O_2<br>(concurrent)	Increase in H_2O_2-induced apoptosis; No statistically significant increase in hsp70 and ROS levels. Decrease in cell viability
Jian et al., 2009	Human liver cancer cells (BEL-7402)	100 Hz,<br>0,7 mT<br>2 or 6 cycles<br>0.5 h on/12 h off	X-rays 2-10 Gy (before or after MF)	Increase in X-ray induced apoptosis; Highest response at 4 and 6 Gy; increased effect with more MF cycles
Eleuteri et al., 2009	Human colon adenocarcinoma cell line (CaCo 2)	50 Hz, 1 mT<br>24, 48, 72 h	TPA<br>(concurrent)	Time-dependent increase in cell growth and protein oxidation
Luukkonen et al., 2011	Human neuroblastoma (SH-SY5Y)	50 Hz, 100 µT<br>24 h	Menadione for 3 h<br>MMS for 3 h<br>(immediately after MF)	Enhancement of Menadione-induced DNA damage, DNA repair rate and MN formation;<br>Similar results with MMS, but not statistically significant
Luukkonen et al., 2014	Human neuroblastoma (SH-SY5Y)	50 Hz, 100 µT<br>24 h	Menadione for 3 h<br>(after MF)	Slight increase in MN frequency 11 and 18 days after MF exposure;<br>Increase in ROS formation and decrease in GSH levels immediately after MF exposure.
Cho et al. (2014)	Human peripheral blood lymphocytes	60 Hz, 0.8 mT<br>Up to 48 h	Gadolinium<br>(concurrent)	Increase in gadolinium-induced strand breaks, MN, ROS and apoptosis and decrease in cell viability.
Buldak et al., 2012	Murine carcinoma cells (AT478)	50 Hz, 1 mT<br>16 min	Cisplatin<br>(concurrent or after MF)	Decrease in cisplatin-induced ROS formation, antioxidant enzyme activity, MDA concentration and DNA damage (comet)
Jin et al., 2012	Mouse fibroblasts (NIH-3T3)<br>Human lung fibroblasts (WI-38)	60 Hz, 0.01, 0.5 and 1 mT<br>4h	H2O2, IR, c-Myc activation<br>(not clear co-exposure protocol)	No effects on MN induction
Lee et al.,-2012	Mouse fibroblasts (NIH3T3)	60 Hz, 1 mT<br>4h	2 Gy γ-rays (before MF);<br>H_2O_2 (concurrent)	No effects on transformation activity
Markkanen et al., 2010	Murine fibroblasts (L929)	50 Hz, 100 or 300 µT<br>1 h and 24 h	UV radiation for 1 h (concurrent or after 24 h MF)	No effects on UV-induced chemiluminescence

ATRATRA: all-trans-retinoid acid; GSH: reduced glutathione; MDA: malondialdeyde; MMS: methyl-metane sulfonate; MN: micronuclei; ROS: Reactive Oxygen Species; TPA: 12-O-tetradecanoylphorbol-13-acetate

<u>RF fields</u>

As reported in Table 24, most of the investigations deal with DNA damage on human cells.

Luukkonen et al. detected an increased DNA migration (comet assay) in human neuroblastoma SH-SY5Y cells co-exposed to 872 MHz, continuous wave, (5 W/kg for 1 h) and menadione with respect to menadione-treated alone cells. This increase was not detected when a GSM signal was employed (Luukkonen et al., 2009).

Zhijian et al. exposed human lymphoblastoid B-cells to 1800 MHz (SAR of 2.0 W/kg) and Doxorubicin (DOX). RF was given intermittently (5 min on/10 min off) for two hours, and several co-exposure protocols were tested. The authors detected influence on repair of DNA damage induced by DOX as a function of the exposure schedule (Zhijian et al., 2010), although in a previous paper the same research group reported that 24 h RF-

exposure in the same experimental conditions, followed by X-rays (0.25 – 2 Gy) did not induce variation in DNA damage (comet assay) induced by X-rays in human white blood cells (Zhijian et al., 2009).

Manti and co-workers exposed human peripheral blood lymphocytes to 4 Gy X-rays followed by 24 h exposure to 1950 MHz, UMTS (SAR 0.5 and 2 W/kg). The RF field did not exacerbate the yield of X-rays-induced aberrant cells, as assessed by chromosomal aberrations, although the frequency of exchanges per cell in X-ray irradiated cells resulted increased, especially at 2 W/kg (Manti et al., 2008).

Four papers were published by the same research group, showing that 20 h pre-exposure of human peripheral blood lymphocytes to RF fields are able to reduce the genotoxic effects induced by chemical or physical mutagens, as assessed by the evaluation of MN frequency. Such an effect was detected in cultures pre-exposed either to 900 MHz, GSM signal (Sannino et al., 2009a) or to 1950 MHz, UMTS, and treated with mitomycin-C. In the latter case a SAR-dependent effect was also detected (Zeni et al., 2012). The authors further evidenced that cells were required to be exposed to RF in the S-phase of the cell cycle to exhibit the reduced DNA damage (Sannino et al., 2011). In a more recent investigation, the authors also demonstrated the ability of 20 h RF exposure (1950 MHz, UMTS, 0.3 W/kg SAR) to induce protection towards X-ray induced chromosomal damage in human peripheral blood lymphocyte cultures (Sannino et al., 2014).

They stated that taken together, their results indicate the ability of RF exposure to induce adaptive response (AR).

Gajski and Garaj-Vrhovac reported an increase in DNA migration, evaluated by means of the alkaline comet assay, in rat blood lymphocytes exposed for 30 minutes to 915 MHz (GSM) 2.4 W/m^2 (calculated SAR of 0.6 W/kg); treatments with honeybee venom given 4 hours before or immediately before RF resulted able to protect against RF-induced DNA damage (Gajski and Garaj-Vrhovac, 2009).

Other studies reported absence of combined effects in terms of genotoxicity. Sannino et al., exposed human fibroblasts from healthy donors and subjects affected by Turner’s syndrome for 24 h to 900 MHz RF field (GSM signal, SAR of 1 W/kg) followed by 1 h treatment with 3-Chloro-4-(dichloromethyl)-5-Hydroxy-2(5h)-furanone (MX), a carcinogen produced during chlorination of drinking water. No increase in MX-induced DNA migration was detected in co-exposed cultures (Sannino et al., 2009b).

Luukkonen and co-workers also failed to find enhancement of DNA migration in human neuroblastoma SH-SY5Y cells concurrently exposed to 872 MHz, continuous wave and GSM, (5 W/kg for 3 h) and ferrous chloride plus Diethyl maleate. Lack of cooperative effects was also detected in terms of ROS production and viability when cells were co-exposed to Ferrous chloride for 1 h (Luukkonen et al., 2010).

Absence of variation in ferrous ions-induced ROS and cell viability was also reported by Brescia et al. (2009) in human lymphoblastoid T cells (Jurkat) co-exposed to 1950 MHz, UMTS signal, irrespective of SAR values (0.5 and 2 W/kg), exposure duration (5-60 min or 24 h) and co-exposure schedule (ferrous ions treatment concurrent or after RF exposure).

On the contrary, Del Vecchio and co-workers reported an increase in some parameters related to oxidative stress following co-exposures to 900 MHz. They co-exposed SN56 cholinergic mice neurons and primary cortical rat neurons to RF (GSM signal, 1 W/kg) and well-known neurotoxic challenges: hydrogen peroxide, glutamate or 25-35 beta-amyloid fragments. Cell death due to oxidative stress induced by hydrogen peroxide was increased by RF co-exposure in SN56 cells but not in primary neurons, while combined treatments with a 25-35 beta-amyloid fragment did not affect cell viability in either cell types (Del Vecchio et al., 2009).

The paper by Canseven et al. (2014) deals with the effect of exposure to 1800 MHz, GSM signal on apoptosis and viability of Burkitt’s lymphoma (Raji) cells with or without Gemcitabine, an inhibition of DNA synthesis and apoptosis inducer. Cell cultures were

exposed to RF for 24 h at a SAR value of 0.35 W/kg. For co-exposure experiments, Gemcitabine was given for 24 h before RF exposure. Apoptosis, measured using Annexin V-FITC and propidium iodide staining, resulted significantly increased, with a decrease in viability, in cells exposed to RF alone, compared to sham exposed cultures. Co-exposures showed significant increase in apoptotic cells and decrease in viability compared to cultures exposed to RF alone. In this investigation, cultures treated with Gemcitabine alone have not been included in the study design.

Only one paper deals with malignant transformation that resulted unaffected in mouse embryonic BALB/3T3 fibroblasts initiated with 3-methylcholanthrene (MCA) and co-exposed to 2142 MHz, W-CDMA RF fields at SARs of 0.08 or 0.8 W/kg and 12-Otetradecanoylphorbol-13-acetate (TPA) (Hirose et al., 2008).

Further research is needed in order to clarify the relevance of these exposures to cancer in humans under real life exposure conditions as well as to explore the potentially beneficial (protective) effects.

Table 24. ***In vitro*** **studies on RF & co-exposures**

Reference	Cell type	RF exposure	Co-exposure	Results
Luukkonen et al., 2009	Human neuroblastoma (SH-SY5Y)	872 MHz, CW and GSM, 5 W/kg<br>1 h	menadione	Increased DNA migration (comet assay) and ROS production in co-exposed cultures with CW respect to cell menadione-treated alone. No effect of co-exposures with GSM signal
Zhijian et al., 2010	Human lymphoblastoid B-cells (HMy2.CIR)	1800 MHz, GSM,2 W/kg<br>2 h intermittent exposure (5 min on, 10 min off) with several exposure schedules	Doxorubicin before, after or concurrent to RF	influence on repair of DNA damage induced by doxorubicin as a function of the exposure schedule
Zhijian et al., 2009	Human white blood cells	1800 MHz, GSM, 2 W/kg<br>24 h intermittent exposure (5 min on, 10 min off)	X-rays after RF exposure (0.25, 0.5, 1.0 and 2.0 Gy)	No cooperative effects (Comet assay at 0, 15, 45, 90, 150 and 240 min after exposure to X-rays)
Manti et al., 2008	Human peripheral blood lymphocytes	1950 MHz, UMTS,<br>0.5 & 2 W/kg<br>24 h	X-rays (4 Gy) immediately before RF	No effects on chromosomal aberrations. Slight increase in the frequency of exchange/cell in cultures co-exposed at 2 W/kg
Sannino et al., 2009a	Human peripheral blood lymphocytes	900 MHz, GSM, 1.25 W/kg mean SAR<br>20 h (from 24 to 44h after PHA)	MMC after 48 h of growth	significant decrease of MN induced by MMC in RF pre-exposed cultures compared to those not pre-exposed to RF
Zeni et al., 2012	Human peripheral blood lymphocytes	1950 MHz, UMTS, 1.25, 0.6, 0.3 and 0.15 W/kg<br>20 h (from 24 to 44h after PHA)	MMC after 48 h of growth	significant decrease of MN induced by MMC only in cultures pre-exposed to RF at SAR of 0.3 W/kg compared to those not pre-exposed to RF
Sannino et al., 2011	Human peripheral blood lymphocytes	900 MHz, GSM, 1.25 W/kg mean SAR<br>20 h in several stages of the cell cycle	MMC after 48 h of growth	significant decrease of MN induced by MMC only in cultures pre-exposed to RF in S phase compared to those not pre-exposed to RF
Sannino et al., 2014	Human peripheral blood lymphocytes	1950 MHz, UMTS,<br>0.3 W/kg<br>20 h (from 24 to 44h after PHA)	1.5 Gy X-rays after 48 h of growth	significant decrease of MN induced by X-rays in RF pre-exposed cultures compared to those not pre-exposed to RF
Gajski and Garaj-Vrhovac,	rat blood lymphocytes	915 MHz, GSM, 2.4 W/m^2<br>(calculated SAR	honeybee venom<br>4 h prior to	Bee venom resulted able to protect against RF-induced DNA damage, as assessed by the alkaline comet

2009		0.6 W/kg) 30 min	and immediately before RF	assay and Fpg-modified comet assay
Sannino et al., 2009b	Human fibroblasts from healthy (ES-1) and Turner's syndrome (TS) donors	900 MHz, GSM, 1 W/kg mean SAR 24 h	MX for 1 h immediately after RF	No enhancement of the MX-induced DNA damage. TS fibroblasts co-exposed to RF for 24 h showed higher but statistically non-significant increases in DNA migration (comet assay) compared to MX-exposed cultures
Luukkonen et al., 2010	Human neuroblastoma (SH-SY5Y)	872 MHz, CW and GSM, 5W/kg 1 h (ROS) or 3 h (DNA migration)	$FeCl_2$ (ROS) or $FeCl_2$ + DEM (DNA migration) Concurrent to RF	No cooperative effects in terms of ROS production, DNA damage and cell viability for all the experimental conditions tested
Brescia et al., 2009	Human lymphoblastoid T cells (Jurkat)	1950 MHz, UMTS, 0.5 and 2 W/kg 5-60 min, 24 h	Ferrous ions ($FeSO_4$) Concurrent or after RF	No cooperative effects in terms of ROS production and cell viability for all the experimental conditions tested
Del Vecchio et al., 2009b	Rat primary cortical neurons; Murine SN56 cholinergic neurons	900 MHz GSM; 1 W/ kg 24 and 144 h	hydrogen peroxide, glutamate or 25-35AA beta-amyloid	No effect of RF alone on viability, proliferation, apoptosis, oxidative stress. Increased hydrogen peroxide-induced oxidative stress in SN56 cells
Canseven et al., (2014)	Burkitt's lymphoma cells (Raji)	1800 MHz, GSM; 0.35 W/kg 24 h	Gemcitabine (before RF)	Increased Gemcitabine-induced apoptosis and decreased cell viability
Hirose et al., 2008	Embryonic mouse fibroblasts BALB/3T3	2142 MHz, W-CDMA; 0.08 and 0.8 W/kg 6 weeks	TPA or MCA + TPA	Neither malignant cell transformation nor tumour promotion with MCA. No tumour co-promotion after co-exposures with TPA

CW: Continuous wave; DEM: Diethyl Maleate; $FeCl_2$: Ferrous Chloride; MCA: 3-methylcholanthrene; MMC: Mitomycin-C; MN: micronuclei; MX: 3-chloro-4-(dichloromethyl)-5-hydroxy-2(5H)-furanone; ROS: Reactive Oxygen Species; TPA: 12-O-tetradecanoylphorbol-13-acetate.

3.11.3. Conclusions on health effects from co-exposure to environmental stressors

Experimental results reported since the previous opinion indicate that co-exposures of environmental stressors (such as physical or chemical agents) with ELF or RF lack consistency. Under the same conditions, effects might be increased, decreased or not influenced at all and are not linked to specific experimental protocols. Due to the small number of available investigations and the large variety of protocols used (different chemical or physical treatments and different EMF exposure conditions), it is not possible to draw definitive conclusions. Therefore, the relevance of co-exposures of environmental stressors (such as physical or chemical agents) with ELF or RF to human health under real-life exposure conditions remains unclear.

3.12. EMF effects on implanted medical devices

It is known that people with implanted active and passive medical devices belong to a group that needs special attention when doing risk assessment for exposure to electromagnetic fields. Medical electronic devices—such as pacemakers, and passive metallic implants (orthopaedic prostheses)—implanted in people of working age are increasingly used. EMF, if sufficiently intense, may interfere with electronic medical devices causing malfunction and subsequent injury or illness. Potential interactions include electromagnetic interference, static magnetic fields which may cause displacement of ferromagnetic implants, and time-varying EMFs which may cause electrostimulation or heating of adjacent tissue, depending on the device or implant and the frequency of the fields. Hocking and Hansson Mild (2008) have published a guidance

note providing generic advice in risk identification, risk assessment and risk control for managements of workers with medical implants exposed to EMF.

There have been some recent studies on the effect of EMF on active and passive implants. Tiikkaja et al. (2012a,b; and 2013) have performed thorough analyses of how pacemakers and implantable cardioverter-defibrillators (ICDs) may be affected by an external ELF magnetic field. They first made an experimental study where they exposed pacemakers (Tiikkaja et al. 2012a) and ICDs (Tiikkaja et al. 2012b) to magnetic fields (2 - 1000 Hz, sinusoidal, pulse, ramp, and square waveforms) created in a Helmholtz coil and with the devices immersed in physiological saline solution in a plastic box. It was observed that pacemaker malfunction occurred in six of the 16 pacemakers, starting almost immediately upon exposure to the strong MF. At some frequencies when using ramp or square waveforms, interference even occurred at levels below public exposure limits. For the ICDs, malfunctions occurred in 11 of the 17 specimens tested. In most cases, no interference occurred at magnetic field levels below the occupational safety limits (ICNIRP 2010).

Tiikkaja et al. (2013) followed up the experimental studies with a study on eleven volunteers with pacemakers and 13 with implantable cardioverter-defibrillators (ICDs). The effect of ELF magnetic fields (sine, pulse, ramp, and square waveform) with flux densities up to 0.3 mT was investigated. Bipolar settings caused no interference, but three of the devices tested in unipolar sensing mode were affected by the highest fields. One was also affected by an EAS gate and a welding cable. The authors conclude that in most cases, employees can return to work after implantation of a bipolar pacemaker or an ICD, but require an appropriate risk assessment. However, pacemakers programmed to unipolar working mode can cause danger to their users in environments with high electromagnetic fields.

Other studies of interest regarding compatibility problems with active implants are for instance Barbouri et al. (2009), Joosten et al. (2009) Korpinen et al. (2012), Souques et at (2011) and Seidman et al. (2010).

The interference with medical devices is a well-known phenomenon in MRI investigations and there are several publications dealing with the heating of the implant and adjacent tissue. However, related risk assessment goes beyond the mandate for this Opinion.

3.13. Research recommendations

Research to date has not been able to identify with any certainty any adverse health effect resulting from exposure to EMFs at any frequency or intensity typically found in the workplace or everyday environment. Epidemiological studies have reported associations between EMF exposure and certain diseases, most notably for an increased risk of childhood leukaemia with exposure to low frequency magnetic fields, but none of these associations can be considered causal, primarily because of shortcomings of those studies, the lack of support from laboratory studies, and an inability to identify biophysical interactions mechanisms. However, not all areas have been studied to the same extent, and research with some frequencies or modulations is very limited, and this is particularly true regarding new and emerging technologies.

A number of areas were identified where the information regarding health effects is either absent or insufficient, or is too discordant to allow science-based assessment of the possibility of health effects. It is recommended that steps are taken to fill these gaps in knowledge, as outlined in the following list of research recommendations. These recommendations are organised by frequency, starting with static fields and rising through the spectrum to THz fields. In addition, recommendations are made for research on combined exposures to various frequencies and co-exposures with other stressors. The previous Opinion from SCENIHR (2009a) also made research recommendations that were enlarged in a second Opinion on research needs and methodology (SCENIHR 2009b).

3.13.1. Static fields including MRI exposure

With regard to static electric fields, there is little information from representative population based samples on thresholds for perception, annoyance, and other effects, especially in the presence of varying ion concentrations in the air. There is a need to collect such data with high priority in view of the upcoming construction of high-voltage DC overhead powerlines. [R1].

There is very little information regarding the health effects of occupational exposure to MRI fields. Therefore, long-term prospective or retrospective cohort studies on workers that are exposed to high stray fields from the construction or operation of MRI devices are recommended as a high priority [R2]. These studies could be used to investigate long-term risk of disease, but also use potential biomarkers for cancer risk and neurological disease as intermediate end-points.

As noted in the previous Opinion, MRI is also increasingly used in paediatric imaging diagnosis. A cohort study into the effects of MRI exposure on children is recommended as a high priority [R3] provided that the feasibility of such a study can be shown in a pilot phase. A retrospective study would have the advantage of allowing future extension of follow-up and incorporation of additional endpoints. Internal comparison between patients with different levels of exposure (number of examinations, body areas examined) would be the most appropriate design. These recommendations are made in order to improve knowledge about possible risks associated with MRI: they are not motivated by existing evidence of adverse effects.

It has been reported that DNA integrity in patients may be affected after an MRI investigation, although the animal and mechanistic data do not suggest that static magnetic fields alone are responsible. Therefore further studies investigating genotoxic effects following MRI investigations in either patients or volunteers are recommended as a medium priority [R4].

As members of staff are increasingly working in the immediate vicinity of MRI equipment, studies investigating possible cognitive effects of exposure to magnetic gradient fields are recommended as a medium priority in humans and animals [R5].

Mechanistic studies with static magnetic fields that address basic neurophysiological effects on neurons are recommended as a low priority [R6]. These have the potential to resolve inconsistencies in the data relating to effects on nervous system.

Further studies on potential developmental effects in animals [R7], and studies with volunteers exploring effects of exposure at 3 T and above on the cardiovascular system [R8] are recommended as a low priority.

In all the available *in vitro* studies with static magnetic fields, gene expression resulted in alterations. Studies on gene expression and epigenetic studies are recommended with medium priority [R9].

3.13.2. ELF fields

Epidemiological studies indicate an increased risk of leukaemia in children exposed to magnetic fields, although there is a lack of support for such an effect from laboratory studies. Further studies using recently-developed mouse models of acute lymphoblastic leukaemia are recommended as a high priority [R10]. These should include exposures during gestation when the initiating events are considered to occur.

The possibility of strain-specific increases in sensitivity to magnetic fields is recommended as a medium priority [R11], since this could lead to the identification of biomarkers. These experiments should be of sufficient size and sensitivity to reject the possibility of false positives.

Whether exposure to magnetic fields may affect the development or progression of Alzheimer's and other neurodegenerative diseases remains unclear and further epidemiological and experimental studies are required. A cohort or register-based case-

control study on magnetic field exposure Alzheimer's disease incidence or mortality is recommended as a high priority [R12]. Laboratory studies are also necessary to gain insight into possible mechanisms, and studies using validated models of Alzheimer's disease are recommended as a high priority [R13]. Of particular interest would be the identification of potential biomarkers.

A recent study suggests an association between maternal magnetic field exposure during pregnancy and asthma and childhood obesity in offspring. These intriguing results require independent confirmation and study using a cohort of pregnant women with measured field exposures, detailed information on potential confounding factors and using standard definitions of obesity is recommended as a medium priority [R14].

Two provocation studies have identified single participants (out of the many who have been tested in this way across the literature) who seemed to react consistently to the presence of electric or magnetic fields (McCarty et al., 2011; Koteles et al., 2013). Independent replication of the ability of the specific participants tested in these studies to react to ELF fields is therefore recommended as a high priority [R15]. These studies should use best practice methods, including the prior registration of a protocol.

3.13.3. IF fields

Research in this area remains very limited and there are very few data regarding health outcomes. The previous Opinion focused on the risks on pregnancy outcome from anti-theft devices in shops because of the exposed area of the body, exposures that may exceed reference levels, and the numbers of young women working in these jobs.

In the absence of new epidemiological data, this study remains a high priority [R16], provided reasonably-sized occupational groups with sufficient exposure can be identified and their exposures can be well-characterized. These studies should also investigate potential biomarkers of exposure, provided appropriate control groups can be chosen. This work should be supplemented with experimental studies using a wider range of exposures and such studies are recommended with a medium priority [R17].

3.13.4. RF fields

Although there is little evidence that moderate use of mobile phones is associated with any cancer in the head and neck region, a prospective cohort study in adults investigating long-term effects of RF fields associated with use of mobile phones is recommended with a high priority [R18]. The study should be of sufficient size and duration to allow the evaluation of realistic effect sizes. The study should reflect the latest developments in exposure assessment, and additional outcomes could include cerebrovascular and neurodegenerative disease.

While the only available study found no increased risk, whether children show an increased tumour risk to RF fields remains unclear. Further studies of the effects of RF fields associated with mobile phone use and brain tumours in children are recommended as a high priority [R19]. These should include children of a younger age than those that have been studied to date, and be of sufficient duration to include assessments of cancer risk later in life. Where practical, other sources that produce significant RF exposure of the brain should be included in assessing exposure.

No further studies investigating the genotoxic or carcinogenic potential of RF fields in animal models are recommended. However, this recommendation should be reconsidered following the publication of the US National Toxicology Program study that is nearing completion.

Several *in vitro* studies have reported effects on non-fixed DNA damage following RF exposure. Further studies on DNA migration, spindle disturbance and foci formation are recommended with a medium priority [R20] to provide additional data and clarification.

The available evidence regarding mobile use on development, cognitive function and behaviour in children does not suggest that adverse effects occur, but the data are

limited and further studies are recommended with a medium priority [R21]. These studies should include characterisation of exposure patterns in (mothers) children and adolescents, and validated exposure assessment. Experimental studies with immature animals can address some of the questions relating to effects on early development of the brain and behaviour.

Most neurophysiological studies on possible effects of RF exposure on brain function in volunteers have been performed with young and predominately male subjects. Since brain structure and brain physiology changes with age possible RF EMF effects may also show age dependencies. It is not known whether effects may change with age, and further studies using elderly and children and adolescent subjects are recommended as a medium high priority on sleep and sleep EEG power [R22], waking EEG [R23], and a medium priority on cognition [R24]. In particular, every study assessing EEG during exposure must ensure that the RF signal does not affect the acquisition of the EEG. If the device used to record the EEG does not offer an adequate resistance against electromagnetic interference, either detectable artefacts in the EEG signal or subtle changes of the electrical properties of the recording system might occur and bias the results. Future studies should report that they have considered this problem.

Studies on possible effects on cognition must pay attention to numerous other factors that can affect the test results. These include exposure design (cross-over vs. parallel group design, exposure before or during testing, avoidance of carryover effects), selection of test subjects (age, sex, inclusion and exclusion criteria), consumption of caffeinated beverages and alcohol, motivation, test sequence and duration, and time of day. For example, a study of 30 young men (Sauter et al. 2011) showed that after correcting for multiple testing, the time of day was the only factor that affected the results of cognitive tests: exposure had no effect.

Overall, there is a high priority research need for (preferably multicentre) neurophysiological studies in volunteers with pre-defined effect sizes, based on *a priori* considerations of power and sample size (type I and type II errors and adequate sample size for the statistical test(s) to be used) for data analysis according to a predefined analysis protocol [R25]. There are a few studies indicating that women are more affected than men, exposure effects vary with age, and that patient populations could be more affected than healthy subjects. Hence, proposed studies should cover a wide range of ages, look at data for females and males separately and, if possible, include patient populations, e.g. insomniacs in sleep studies or patients with neurological disorders including neurodegenerative diseases.

Although most studies have suggested that RF fields are unlikely to be the cause of the symptoms that are attributed to them, it is clear that these symptoms can have a major detrimental impact on quality of life. Additional research on RF mechanisms of these symptoms is recommended as a low priority [R26]. Research recommendations relating to symptoms that currently have no adequate medical explanation are outside of the remit for this Opinion. However, other expert bodies should consider whether the evidence base for treating patients who attribute symptoms to EMF is currently adequate. The evidence suggesting that RF fields affect male fertility is weak and the existing ex vivo studies reporting positive effects have methodological problems. Cohort studies are recommended only if a study design is available that can overcome potential confounding and recall bias regarding phone use and the study has appropriate exposure assessment.

An animal study investigating effects on reactive oxygen species activity in field-exposed sperm is recommended as a low priority [R27] provided the study has sufficient power to detect subtle changes (reported effect sizes are modest) and employs detailed computational methods to characterise the absorbed power in the testes.

3.13.5. THz technologies

Considering the expected increase in the use of THz technologies, experimental research related to possible adverse effects on the skin and the cornea is recommended as a high

priority [R28]. In particular, human and animal studies should focus on the effects of long-term, low-level exposure on the skin, and on the effects of high-intensity, short-term exposure on the cornea. Studies to date have used a relatively narrow frequency range (0.1-1 THz) so future studies should also use higher frequencies.

Monitoring of occupationally-exposed groups for skin and eye changes and disorders is recommended as medium priority [R29], provided suitably-sized groups with sufficient and well-characterised exposure can be identified with an appropriately matched control group.

3.13.6. Combined exposures to EMF

Although few studies have examined this possibility, the available data suggest that combined exposures to different fields or signals do not cause significant effects with total exposures below international guideline values.

Further laboratory studies investigating effects of combined exposures on genotoxicity, cancer, development and neurobehaviour are recommended as a medium priority [R30]. In particular, since people are exposed to a variety of frequencies in the everyday environment, the effects of combined exposures to low and high frequencies should be examined.

3.13.7. Co-exposure with other agents

Further animal studies are recommended as medium priority to clarify the role of co-exposure to magnetic fields as a co-carcinogen [R31] and the apparent protective effects of RF fields against the ionizing radiation [R32].

Further *in vitro* research is needed to clarify the relevance of combined exposures to human carcinogenicity under real life conditions and to explore the potentially beneficial (protective) effects of such exposures on humans. These studies are recommended with a medium priority [R33] provided that justification can be provided for the chosen model (for both EMF exposure and co-treatment).

3.13.8. Exposure assessment

Microdosimetry aims at the quantitative investigation of the interaction of electromagnetic fields at the microscopic level, i.e. at cellular or subcellular levels. With the emergence of THz technology and nanosecond pulses applications this area of exposure assessment needs to be strengthened both experimentally (e.g. single cell exposure setups) as well as the theoretically, since it may result in the elucidation of underlying biophysical mechanisms that are still missing. This research subject [R34] can be considered of medium priority.

The dielectric properties of tissues are of utmost importance in the exposure assessment with numerical techniques, both for medical applications as well as experiments in bioelectromagnetics. There is a scarcity of data and systematic studies in the literature for these properties at static fields and the lower ELF and THz ranges, introducing a high degree of uncertainty in the evaluated electromagnetic field distributions. Dielectric spectroscopy measurements of - preferably - human tissues from subjects of different ages, gender or physiological conditions [R35] are of high priority.

In prospective epidemiological studies it is useful to be able to characterize personal exposure with several types of metrics both for the general public and the workers. The instrumentation that is available currently is either detailed and expensive, making itself prohibitive to be used for large samples, or cheap and prone to large uncertainties and exposure misclassification. It is necessary, but at a medium priority, to continue the research in the manufacturing of new affordable instrumentation or the improvement of existing specialized exposure meters [R36]. It is equally important to launch new methodologies in collecting exposure data at a personal or an environmental level with

the use of simple everyday equipment, like mobile electronic devices, and techniques like crowd-sensing [R37].

Table 25. Research recommendations by type of field and priority

Type of field	High priority	Medium priority	Low priority
SMF inc MRI	R1, R2, R3	R4, R5,R9	R6, R7, R8
ELF	R10, R12, R13, R15	R11, R14	
IF	R16	R17	
RF	R18, R19, R22, R23, R24,	R30, R21, R24	R26, R27
THz	R28	R29	
Combined		R30	
Co-exposure		R31, R32, 33	
Exposure assessment	R35	R34, R36, R37	

3.14. Guidance on research methods

As mentioned in section 3.2, there are a number of limitations and practical difficulties common to all lines of scientific research dealing with the study of the biological and possible health effects of EMF. These limitations have often resulted in data that are unsuitable or unusable for the purposes of risk assessment. In this section, several recommendations are made to researchers which are intended to function as a guide to improve experimental design and to offer some minimum requirements to ensure the quality of the data that are collected can be used for risk assessment.

Because of the large number of different endpoints and protocols that are used in bioelectromagnetics research, it is not possible to produce a single, multipurpose exposure setup that is applicable to all types of study. Nevertheless, a generic design algorithm for the development of experimental setups in this area was published more than ten years ago by Kuster and Schönborn (2000). This document described the minimal requirements necessary to achieve the appropriate quality of data for risk assessment. It was the intention of the authors that those guidelines "might be of benefit not only as a yardstick for setup designers, but also for reviewers and bodies evaluating programs and studies". Unfortunately, this objective has only been partially accomplished, because studies have continued to be published which do not comply with several critical requirements of the document.

Recently, more detailed guidance has become available on experimental design for *in vitro* experiments using RF fields. Although it is still not possible to specify a single exposure system, it is possible to specify some priorities in design to ensure that the appropriate exposure system is identified and used (Paffi et al., 2010). Among the most important priorities to be met in the procedure of designing or choosing an *in vitro* exposure system is the ability to accurately determine the electric and magnetic fields in the exposed samples and to ensure there are experimental conditions optimal for cell growth. Controlled conditions are also required for biological materials that are not limited to *in vitro* experiments using RF fields. The appropriate cell model has to be chosen for specific experimental approaches, and the standardization of cell culture is achieved by controlling the materials, such as cells and culture medium, that interact and determine the properties of the whole system. More than one endpoint has to be investigated for each cellular target in order to also balance mechanistic vs. toxicity studies. Thus, a combination of techniques, confirming and/or complementing each other, is recommended for the reliable detection of effects. A general requirement for the biological assay in a well-designed *in vitro* experiment is the high sensitivity, and particular care must be devoted to set up accurate experimental control samples. Negative and positive controls provide evidence for controlled experimental conditions, while sham exposed samples, and blind exposure conditions are also necessary. Finally,

the procedures established in preliminary experiments have to be recorded in writing and strictly followed throughout the subsequent experiments in a Good Laboratory Practices (GLP)-like approach. These have to allow understanding of what was done, and why it was done, and to allow the biological relevance of the study to be independently scrutinized and the reliability and validity of the findings to be assessed. There should always be enough information in publications to allow the experiments to be repeated by independent laboratories (Zeni and Scarfi, 2012).

Exposure assessment in all biological experiments should be as accurate as possible. However, the evaluation of electric and magnetic field distributions is not trivial, especially when dealing with humans and laboratory animals, since the field distributions depend not only on physical factors such as wavelength, but also on biological factors such as body size and body shape, and on variables such as body posture. Nevertheless, calculations and measurements of the absorbed energy within the organism are important to determine not only how much energy was absorbed, but also where absorption actually occurred in the body (Paffi et al., 2013). Indeed, organ-specific dosimetry is considered necessary to help to establish causality. The methodology for dosimetry in animal experiments with a special emphasis on uncertainty calculations and both intra- and inter-animal variation is given by Kuster et al. (2006). In addition, Paffi et al. (2013) provide a systematic review and classification of *in vivo* microwave exposure systems used for bioelectromagnetics research in the last decade. The main features of each system's typology are presented and discussed for different types of experiments. This review of the strengths and weaknesses of each exposure system is useful for identifying the features necessary for new studies.

While the majority of recent human provocation studies have been of reasonably good quality, scope remains for researchers to improve the future methodological rigour of this field still further. In particular, the quality of reporting in many papers can sometimes make it difficult to assess exactly what was done, how it was done or even why it was done. Particular issues currently exist in terms of the details provided as to which areas of the brain were exposed, how double-blinding was achieved, how the sample size for the study was determined, how the issue of conducting multiple statistical analyses was treated, and, in case of not statistically significant results, a power consideration should be addressed. With respect to exposure of the brain, a guidance for the design of respective exposure setups already exists in the literature (Kuster et al., 2004) as well as comparisons between various setups (Boutry et al., 2008) and exemplary studies of thorough dosimetric analysis (Schmid et al., 2012; Murbach et al., 2012) at different frequency ranges. In particular, for provocation studies with cognitive performance as the investigated parameter, several aspects of experimentation and corresponding recommendations were given by Regel and Achermann (2011).

It is apparent that the large majority of human provocation studies in this field fail to lodge their experimental protocols with a publically accessible repository before starting their data collection. Publishing a detailed protocol has become a common practice for "any research study that prospectively assigns human participants or groups of humans to one or more health-related interventions to evaluate the effects on health outcomes" (Laine et al. 2007) and is now recommended or required by many mainstream medical journals, the World Health Organization and the Declaration of Helsinki (WMA General Assembly, 2008). Registration guards against publication bias for studies as a whole, and selective reporting of outcomes or analyses within specific studies. It is disappointing that registration has not, as yet, been adopted as standard practice among researchers investigating effects of EMF. Benefactors, researchers and journal editors within this field should consider how registration can be encouraged.

These methodological problems also apply to epidemiological studies. A very good introduction in such problems, although specific to mobile phones and cancer, is the work by Auvinen et al. (2006), which can help researchers identify and eliminate potential limitations of their own study designs.

4.OPINION

As part of its mandate, the SCENIHR is asked to continuously monitor new information that may influence the assessment of risks to human health in the area of electromagnetic fields (EMF) and to provide regular updates on the scientific evidence base to the Commission.

A sufficient number of new scientific publications have appeared since the last Opinion of 2009 to warrant a new analysis of the scientific evidence on possible effects on human health of exposure to EMF. In addition, the development of novel technologies using THz fields calls for new assessments also in this frequency range.

Held on 16-17 November 2011, the International Conference on EMF and Health, organized by the European Commission under the auspices of the SCENIHR, provided an overview of the most recent scientific developments in this area as a first preparation for a future scientific Opinion.

Consequently, the SCENIHR is being asked to examine this new scientific evidence and to address in particular the four major questions listed in the Terms of Reference.

1. To update its Opinion of 2009 in the light of newly available information.

In most of the sections of the Scientific Rationale in the current Opinion, reports appearing in the literature after 2009, i.e. after the publication of the previous Opinion, have been considered. Therefore, the present Opinion covers studies that were published (at least electronically) between 2009 and June 30, 2014. However, certain sections of the Scientific Rationale were not covered in the previous Opinions. In such cases, reports published before 2009 have also been taken into account for the risk assessment.

2. To give particular attention to issues affected by important gaps in knowledge in the previous Opinions, especially:

2a. the potential adverse effects of EMF on the nervous system, including neuro-behavioural disorders and on the risk of neo-plastic diseases;

RF fields

Previous studies suggesting that RF exposure may affect brain activities as reflected by changes in the EEG during wake and sleep are confirmed by results of more recent studies. However, given the variety of applied fields, duration of exposure, number of considered leads, and statistical methods it is difficult to derive firm conclusions. For event-related potentials and slow brain oscillations results are inconsistent. Likewise, studies on cognitive functions in humans lack consistency. The biological relevance of reported small physiological EEG changes remains unclear, and mechanistic explanation is still lacking.

A reasonable body of experimental evidence now suggests that exposure to RF does not trigger symptoms, at least in the short-term. While additional observational studies are required to assess whether longer-term exposure could be associated with symptoms, the evidence to date weighs against a causal effect.

Human studies on neurological diseases and symptoms show no clear effect, but the evidence is limited. Human studies on child development and behavioural problems suffer from conflicting results and methodological limitations. Therefore, the evidence of an effect is weak. Effects of exposure on foetuses from mother's mobile phone use during pregnancy are not plausible owing to extremely low foetal exposure.

Overall, the epidemiological studies on RF EMF exposure do not show an increased risk of brain tumours. Furthermore, they do not indicate an increased risk for other cancers of the head and neck region. Some studies raised questions regarding an increased risk of glioma and acoustic neuroma in heavy users of mobile phones. The results of cohort and incidence time trend studies do not support an increased risk for glioma while the

possibility of an association with acoustic neuroma remains open. Epidemiological studies do not indicate increased risk for other malignant diseases including childhood cancer.

A considerable number of well-performed *in vivo* studies using a wide variety of animal models have been mostly negative in outcome. These studies are considered to provide evidence for the absence of a carcinogenic effect.

A large number of *in vitro* studies pertaining to genotoxic as well as non-genotoxic end-points have been published since the last Opinion. In most of the studies, no effects of exposure at levels below exposure limits were recorded, although in some cases DNA strand breaks and spindle disturbances were observed.

IF fields

This part of the frequency spectrum remains poorly investigated with respect to potential health effects resulting from exposure to EMF.

ELF fields

Studies investigating possible effects of ELF MF exposure on the power spectra of the waking EEG of volunteers are too heterogeneous with regard to applied fields, duration of exposure, number of considered leads, and statistical methods to draw any sound conclusion. The same applies for the results concerning behavioural outcomes and cortical excitability.

Only a few new epidemiological studies on neurodegenerative diseases have been published since the previous Opinion. They do not provide support for the previous conclusion that ELF magnetic field exposure could increase the risk for Alzheimer's disease or any other neurodegenerative diseases or dementia. Animal studies that have suggested beneficial effects of strong magnetic fields require confirmation.

The evidence with respect to self-reported symptoms is discordant. While most studies have not found an effect of exposure, two experimental studies have identified individual participants who may reliably react to magnetic fields. However, replication of these findings is essential before weight is given to these results.

The new epidemiological studies are consistent with earlier findings of an increased risk of childhood leukaemia with estimated daily average exposures above 0.3 to 0.4 µT. As stated in the previous Opinions, no mechanisms have been identified and no support from experimental studies could explain these findings, which, together with shortcomings of the epidemiological studies prevent a causal interpretation.

2b. the understanding of biophysical mechanisms that could explain observed biological effects and epidemiological associations;

Despite a number of studies continuing to report candidate mechanisms, particularly regarding effects on reactive oxygen species, lipid peroxidation and antioxidant defence, no mechanism that operates at levels of exposure found in the everyday environment has been firmly identified and experimentally validated. It is important to stress here the difficulties of demonstrating small changes in gene expression that may occur following *in vivo* exposure to EMF which are due to inherent variability of biological responses and the technical limitations in the sensitivity of existing technologies.

2c. the potential role of co-exposures with other environmental stressors in biological effects attributed to EMF.

Experimental results reported since the previous opinion indicate that co-exposures of environmental stressors (such as physical or chemical agents) with ELF or RF lack consistency. Under the same conditions, effects might be increased, decreased or not influenced at all and are not linked to specific experimental protocols. Due to the small number of available investigations and the large variety of protocols used (different chemical or physical treatments and different EMF exposure conditions), it is not possible to draw definitive conclusions. Therefore, the relevance of co-exposures of environmental

stressors (such as physical or chemical agents) with ELF or RF to human health under real-life exposure conditions remains unclear.

3. To review the scientific evidence available to understand the potential adverse health effects of EMF in the THz range.

The number of studies investigating potential biological, non-thermal effects of THz fields is small, but has been increasing over recent years, due to the availability of adequate sources and detectors.

In vivo studies indicate mainly beneficial effects on disorders of intravascular components of microcirculation in rats under immobilization stress, but do not address acute and chronic toxicity or carcinogenesis. *In vitro* studies on mammalian cells differ greatly with respect to irradiation conditions and endpoints under investigation. Studies suggesting effects of exposure have not been replicated in independent laboratories. Some theoretical mechanisms have been proposed, but no conclusive experimental support is available.

4. To develop a set of prioritized research recommendations updating previous efforts in this area (in particular by the SCENIHR and the WHO). These recommendations should include methodological guidance on the experimental design and minimum requirements to ensure data quality and usability for risk assessment.

A set of prioritized research recommendations and methodological guidance on the experimental design and minimum requirements to ensure data quality and usability for risk assessment are provided in chapters 3.14 and 3.15 of the Opinion.

5. MINORITY OPINION

None

6. CONSIDERATION OF THE RESPONSES RECEIVED DURING THE CONSULTATION PROCESS

A public consultation on this Opinion was opened on the website of the Scientific Committees from 4 February to 16 April 2014. A public hearing was also held in Athens on 27 March 2014.

Information about the public consultation and the hearing was broadly communicated to national authorities, international organisations and other stakeholders.

The aim of these open consultations were to present the preliminary opinion and gathering specific comments, suggestions, explanations or contributions on the scientific basis of the opinion, as well as any other scientific information regarding the questions addressed.

57 organisations and individuals participated in the public consultation providing 186 comments to different chapters and section of the opinion. Each submission was carefully considered by the SCENIHR and the scientific opinion has been revised to take account of relevant comments. The cut-off date for the literature review was extended and the literature has been accordingly updated with relevant publications. The scientific rationale and the opinion section were clarified and strengthened.

The SCENIHR thanks all contributors for their comments and for references sent during the public consultation.

The text of the comments received and the response provided by the SCENIHR are available here:

http://ec.europa.eu/health/scientific_committees/consultations/public_consultations/scenihr_consultation_19_en.htm

7. GLOSSARY

This section includes technical terms and definitions used within the document. The definitions are given in alphabetical order.

Alpha-band/waves: A specific frequency range (8-13 Hz) of the human EEG activity which is associated with relaxed wakefulness.

Conductivity: A property of a material that determines the magnitude of the electric current density when an electric field is impressed on the material.

Confounding factor (confounder): A confounding factor in an epidemiological study is a variable which is related to one or more of the variables defined in a study. The confounder may mask an actual association or falsely demonstrate an apparent association between the study variables where no real association between them exists. If confounding factors are not measured and considered, bias may result in the conclusion of the study.

Contralateral: On the opposite from another structure.

Contralateral use of mobile phone: Preferred side of the head during mobile phone use corresponds to the side of the head opposite to the tumour.

Crossover design: A cross over design is a special situation where a separate comparison group is not present. Instead, each subject receives both treatments or is exposed to both sham and active exposure and the outcomes under the two conditions are compared within the same subjects. Thus, the subject serves as his/her own control. Ideally in a crossover design, a subject is randomly assigned to a specific treatment/exposure order.

Dielectric properties: In the context of this document the properties of a materials conductivity and permeability.

Double-blind (study): Blinding is used to prevent conscious as well as subconscious bias (e.g. by expectations) in research. In a double-blinded study the participants as well as the researchers are unaware of (blind to) the nature of the treatment (e.g. a new drug or placebo) or the exposure condition (e.g. the exposure under study or sham) that the participants receive in the study.

Ecological studies: An ecological or correlational study is one in which the unit of analysis is an aggregate of individuals and information is collected on this group rather than on individual members. The association between a summary measure of disease and a summary measure of exposure is studied. An error of reasoning occurs when conclusions are drawn about individuals from data that are associated with groups, as relationships observed for groups may not necessarily hold for individuals.

Electric field strength (E): The magnitude of a field vector at a point that represents the force (F) on a charge (q). E is defined as $E = F/q$ and is expressed in units of Volt per meter (V/m).

Electroencephalogram (EEG): Extracellular recording of the electrical activity of the cerebral cortex.

Electromagnetic field: Electromagnetic phenomena expressed in vector functions of space and time.

Electromagnetic radiation: The propagation of energy in the form of electromagnetic waves through space.

EMF: Electromagnetic field.

Exposure: Exposure occurs wherever a person is subjected to electric, magnetic or electromagnetic fields or contact currents other than those originating from physiological processes in the body.

Extremely low frequency (ELF): Extremely low frequency fields include, in this document, electromagnetic fields from 1 to 300 Hz.

Far field: The far field of an antenna or other source of an electromagnetic field is the field that is at a distance away which is far exceeding the wavelength of the field.

Frequency modulation (FM): Frequency Modulation is a type of modulation representing information as variations in the frequency of a carrier wave. FM is often used at VHF frequencies (30 to 300 MHz) for broadcasting music and speech.

Frequency (Hz): The number of cycles of a repetitive waveform per second.

Intermediate frequencies (IF): Intermediate frequencies are, in the frame of this report, defined as frequencies between 300 Hz and 100 kHz.

Ipsilateral: On the same side as another structure.

Ipsilateral use of mobile phone: Preferred side of the head during mobile phone use corresponds to the side of the head where the tumour is located.

Magnetic flux density (B): The magnitude of a field vector at a point that results in a force (F) on a charge (q) moving with the velocity (v). The force F is defined by $F = q*(v \times B)$ and is expressed in units of Tesla (T).

Magnetic field strength (H): The magnitude of a field vector that is equal to the magnetic flux density (B) divided by the permeability (μ) of the medium. H is defined as $H = B/\mu$ and is expressed in units of Ampere per metre (A/m).

Microwaves: Microwaves are defined in the frame of this expertise as electromagnetic waves with wavelengths of approximately 30 cm (1 GHz) to 1 mm (300 GHz).

Milliwatt (mW): A unit of power equal to 10^{-3} Watt.

Nanowatt (nW): A unit of power equal to 10^{-9} Watt.

Near field: The near field of an antenna or other source of an electromagnetic field is the field in the close vicinity of the source, much less than the wavelength of the field.

Nocebo: A nocebo effect is an adverse, non-specific effect caused by expectation or belief that something is harmful.

Non – thermal effects (or athermal effects): An effect which can only be explained in terms of mechanisms other than increased molecular motion (i.e. heating), or occurs at absorbed power levels so low that a thermal mechanism seems unlikely, or displays such an unexpected dependence upon an experimental variable that it is difficult to see how heating could be the cause.

Permeability (μ): A property of a material that indicates how much polarisation occurs when an electric field is applied.

Power density (S): Power per unit area normal to the direction of propagation, usually expressed in watt per square meter (W/m^2).

Radio frequency (RF): The frequencies between 100 kHz and 300 GHz of the electromagnetic spectrum.

Sham exposure: A control condition used to simulate the environmental conditions of the exposure under study, but in absence of exposure (Similar to Placebo-controlled, which is a term used to de scribe a method of research in which an inactive substance (a placebo) is given to one group of participants, while the treatment (usually a drug or a vaccine) being tested is given to another group. The results obtained in the two groups are then compared to see if the investigative treatment is more effective (or has more negative effects) than placebo. Both treatments may also be given in succession to the same subjects, see crossover design.)

Specific energy absorption rate (SAR): A measure of the rate of energy absorbed by or dissipated in an incremental mass contained in a volume element of dielectric

materials such as biological tissues. SAR is usually expressed in terms of watts per kilogram (W/kg).

Static electric field: Static fields produced by fixed potential differences.

Static magnetic fields (SMF): Static fields established by permanent magnets and by steady currents.

VDU: Video display units for computers, videos, TV and some measurement devices using cathode ray tubes.

8. REFERENCES

References marked in italics were added after the public consultation.

Aboul Ezz HS, Khadrawy YA, Ahmed NA, Radwan NM, El Bakry MM (2013). The effect of pulsed electromagnetic radiation from mobile phone on the levels of monoamine neurotransmitters in four different areas of rat brain. Eur Rev Med Pharmacol Sci, 17(13), 1782-8.

Aldad TS, Gan G, Gao XB, Taylor HS (2012). Fetal radiofrequency radiation exposure from 800-1900 MH-rated cellular telephones affects neurodevelopment and behavior in mice. Sci Rep, 2, 312.

Adams JA, Galloway TS, Mondal D, Esteves SC and Mathews F (2014). Effect of mobile telephones on sperm quality: A systematic review and meta-analysis. Environ Int. 70, 106-12.

Adang D, Remacle C, Vorst A V (2009). Results of a long-term low-level microwave exposure of rats. IEEE Trans Microwave Theory Techniques, 5, 2488-97.

Aerts S, Plets D, Verloock L, Martens L, Joseph W (2013). Assessment and comparison of total RF-EMF exposure in femtocell and macrocell base station scenarios. Radiat Prot Dosimetry, 162(3), 236-43.

Ahlbom A, Day N, Feychting M, Roman E, Skinner J, Dockerty J, Linet M, McBride M, Michaelis J, Olsen JH, Tynes T, Verkasalo PK (2000). A pooled analysis of magnetic fields and childhood leukaemia. Br J Cancer, 83(5), 692-8.

Ahlers MT, Ammermüller J (2013). No influence of acute RF exposure (GSM-900, GSM-1800, and UMTS) on mouse retinal ganglion cell responses under constant temperature conditions. Bioelectromagnetics, 35 (1), 16-29.

Aït-Aïssa S, Billaudel B, Poulletier de Gannes F (2012). In utero and early-life exposure of rats to a Wi-Fi signal: screening of immune markers in sera and gestational outcome. Bioelectromagnetics, 33 (5) 410 - 20.

Akdag MZ, Dasdag S, Ulukaya E, Uzunlar AK, Kurt MA, Taşkin A (2010). Effects of extremely low-frequency magnetic field on caspase activities and oxidative stress values in rat brain. Biol Trace Elem Res, 138(1-3), 238-49.

Akdag MZ, Dasdag S, Cakir DU, Yokus B, Kizil G and Kizil M (2013). Do 100- and 500-μT ELF magnetic fields alter beta-amyloid protein, protein carbonyl and malondialdehyde in rat brains? Electromagn Biol Med, 32(3), 363-72.

Akpinar D, Ozturk N, Ozen S, Agar A, Yargicoglu P (2012). The effect of different strengths of extremely low-frequency electric fields on antioxidant status, lipid peroxidation, and visual evoked potentials. Electromagn Biol Med, 31(4), 436-48.

Al-Ali BM, Patzak J, Fischereder K, Pummer K and Shamloul R (2013). Cell phone usage and erectile function. Cent European J Urol, 66(1), 75-7.

Alanko T, Puranen L, Hietanen M (2011). Assessment of exposure to intermediate frequency electric fields and contact currents from a plasma ball. Bioelectromagnetics 32(8), 644-51.

Alexandrov BS, Gelev V, Bishop AR, Usheva A, Rasmussen KO (2010). DNA breathing dynamics in the presence of a terahertz field. Physics Letters A, 374(10), 1214–7.

Alexandrov BS, Rasmussen KØ, Bishop AR, Usheva A, Alexandrov LB, Chong S, Dagon Y, Booshehri LG, Mielke CH, Phipps ML, Martinez JS, Chen HT, Rodriguez G (2011). Non-thermal effects of terahertz radiation on gene expression in mouse stem cells, Biomed Opt Express, 2(9), 2679-89.

Alhekail Z O I. (2001). Electromagnetic radiation from microwave ovens. Journal of Radiological Protection, 21(3), 251-8.

Amirifalah Z, Firoozabadi SM, Shafiei SA (2013). Local exposure of brain central areas to a pulsed ELF magnetic field for a purposeful change in EEG. Clin EEG Neurosci, 44(1), 44-52.

Andel R, Crowe M, Feychting M, et al. (2010). Work-related exposure to extremely low-frequency magnetic fields and dementia: results from the population-based study of dementia in Swedish twins. J Gerontol A Biol Sci Med Sci, 65(11), 1220-7.

Anger G. (2010). Low frequency magnetic fields in different modes of transport: a study based on measurements from the years 1993 to 2010. Swedish Radiation Safety Agency Rapportnummer: 2010:20 Juni 2010 (In Swedish with English Summary).

Antunes A, Glover PM, Li Y, Mian OS, Day BL (2012). Magnetic field effects on the vestibular system: calculation of the pressure on the cupula due to ionic current-induced Lorentz force. Phys Med Biol, 57, 4477-87.

Arendash GW, Mori T, Dorsey M, Gonzalez R, Tajiri N, Borlongan C (2012). Electromagnetic treatment to old Alzheimer's mice reverses β-amyloid deposition, modifies cerebral blood flow, and provides selected cognitive benefit. PLoS One, 7(4), e35751.

Arendash GW, Sanchez-Ramosa J, Mori T, Mamcarz M, Lin X, Runfeldt M, Wang L, Zhang G, Sava V, Tan J, Cao C (2010). Electromagnetic field treatment protects against and reverses cognitive impairment in Alzheimer's disease mice. J Alzheimer's Disease, 19, 191-210.

Auger N, Joseph D, Goneau M, Daniel M (2011). The relationship between residential proximity to extremely low frequency power transmission lines and adverse birth outcomes. J Epidemiol Community Health, 65(1), 83-5.

Auger N, Park AL, Yacouba S, et al. (2012). Stillbirth and residential proximity to extremely low frequency power transmission lines: a retrospective cohort study. Occup Environ Med, 69(2), 147-9.

Augner C, Hacker GW (2009). Are people living next to mobile phone base stations more strained? Relationship of health concerns, self-estimated distance to base station, and psychological parameters. Indian J of Occup and Environ Med, 13, 141-5.

Augner C, Gnambs T, Winker R, Barth A (2012). Acute effects of electromagnetic fields emitted by GSM mobile phones on subjective well-being and physiological reactions: a meta-analysis. Sci Total Environ, 424, 11-5.

Auvinen A, Toivo T, Tokola K (2006). Epidemiological risk assessment of mobile phones and cancer: where can we improve? Eur J Cancer Prev, 15(6), 516-23.

Avendaño C, Mata A, Sanchez Sarmiento CA, Doncel GF (2012). Use of laptop computers connected to internet through Wi-Fi decreases human sperm motility and increases sperm DNA fragmentation. Fertil Steril, 97(1), 39-45.

Aydin D, Feychting M, Schüz J, Andersen TV, Poulsen AH, Prochazka M, Klæboe L, Kuehni CE, Tynes T, Röösli M (2011). Predictors and overestimation of recalled mobile phone use among children and adolescents. Prog Biophys Mol Biol, 107(3), 356-61.

Aydin D, Feychting M, Schüz J, Röösli M, CEFALO study team (2012). Childhood brain tumours and use of mobile phones: comparison of a case-control study with incidence data. Environ Health, 11, 35.

Baan R, Grosse Y, Lauby-Secretan B, El Ghissassi F, Bouvard V, Benbrahim-Tallaa L, Guha N, Islami F, Galichet L, Straif K,WHO International Agency for Research on Cancer Monograph Working Group (2011). Carcinogenicity of radiofrequency electromagnetic fields. Lancet Oncol, 12(7), 624-6.

Babouri A, Hedjeidj A, Guendouz L (2009). Experimental and theoretical investigation of implantable cardiac pacemaker exposed to low frequency magnetic field. Journal of Clinical Monitoring and Computing, 23, 63–73.

Bahr A, Dorn H, Bolz T (2006). Dosimetric assessment of an exposure system for simulating GSM and WCDMA mobile phone usage. Bioelectromagnetics, 27, 320-7.

Bahr A, Adami C, Bolz T, Dorn H, Ruttiger L (2007). Exposure setups for laboratory animals and volunteer studies using body-mounted antennas. Radiat Prot Dosimetry, 124, 31-4.

Bai WF, Xu WC, Feng Y, Huang H, Li XP, Deng CY, Zhang MS (2013). Fifty-Hertz electromagnetic fields facilitate the induction of rat bone mesenchymal stromal cells to differentiate into functional neurons. Cytotherapy, 15(8), 961-70.

Balassa T, Szemerszky R, Bárdos G (2009). Effect of short-term 50 Hz electromagnetic field exposure on the behavior of rats. Acta Physiol Hung, 96(4), 437-48.

Balassa T, Varró P, Elek S, Drozdovszky O, Szemerszky R, Világi I and Bárdos G (2013). Changes in synaptic efficacy in rat brain slices following extremely low-frequency magnetic field exposure at embryonic and early postnatal age. Int J Dev Neurosci, 31(8), 724-30.

Baldi I, Coureau G, Jaffré A, Gruber A, Ducamp S, Provost D, Lebailly P, Vital A, Loiseau H, Salamon R (2011). Occupational and residential exposure to electromagnetic fields and risk of brain tumors in adults: a case-control study in Gironde, France. Int J Cancer, 129(6), 1477-84.

Baliatsas C, Van Kamp I, Kelfkens G, Schipper M, Bolte J, Yzermans J, Lebret E (2011). Non-specific physical symptoms in relation to actual and perceived proximity to mobile phone base stations and powerlines. BMC Public Health, 11, 421.

Baliatsas C, Van Kamp I, Bolte J, Schipper M, Yzermans J, Lebret E (2012). Non-specific physical symptoms and electromagnetic field exposure in the general population: Can we get more specific? A systematic review. Environment International, 41, 15-28.

Ballardin M, Tusa I, Fontana N, Monorchio A, Pelletti C, Rogovich A, Barale R, Scarpato R (2011). Non-thermal effects of 2.45 GHz microwaves on spindle assembly, mitotic cells and viability of Chinese hamster V-79 cells. Mutation Research, 716, 1– 9.

Barth A, Winker R, Ponocny-Seliger E, Mayrhofer W, Ponocny I, Sauter C, Vana N A (2008). Meta-analysis for neurobehavioural effects due to electromagnetic field exposure emitted by GSM mobile phones. Occup Environ Med, 65, 342-46.

Barth A, Ponocny I, Gnambs T, Winker R (2012). No effect of short-term exposure to mobile phone electromagnetic fields on human cognitive performance: a meta-analysis. Bioelectromagnetics, 33, 159-65.

Bartsch H, Küpper H, Scheurlen U, Deerberg F, Seebald E, Dietz K, Mecke D, Probst H, Stehle T, Bartsch C (2010). Effect of chronic exposure to a GSM-like signal (mobile phone) on survival of female Sprague-Dawley rats: modulatory effects by month of birth and possibly stage of the solar cycle. Neuro Endocrinol Lett, 31(4), 457-73.

Bas O, Odaci E, Mollaoglu H, Ucok K, Kaplan S (2009b). Chronic prenatal exposure to the 900 megahertz electromagnetic field induces pyramidal cell loss in the hippocampus of newborn rats. Toxicol Ind Health, 25(6), 377-84.

Baste V, Hansson Mild K, Moen BE (2010). Radiofrequency exposure on fast patrol boats in the Royal Norwegian Navy-an approach to a dose assessment. Bioelectromagnetics, 31(5), 350-60.

Baste V, Moen BE, Oftedal G, Strand LA, Bjørge L, Mild KH (2012). Pregnancy outcomes after paternal radiofrequency field exposure aboard fast patrol boats. J Occup Environ Med, 54(4), 431-8.

Belenky I, Margulis A, Elman M, Bar-Yosef U, & Paun S D (2012). Exploring Channeling Optimized Radiofrequency Energy: a Review of Radiofrequency History and Applications in Esthetic Fields. Advances in Therapy, 29(3), 249-66.

Belyaev IY, Markova E, Hillert L, Malmgren LOG, Persson BRR (2009). Microwaves From UMTS/GSM Mobile Phones Induce Long-Lasting Inhibition of 53BP1/g-H2AX DNA Repair Foci in Human Lymphocytes. Bioelectromagnetics, 30, 129-41.

Beneduci A (2009). Evaluation of the potential in vitro antiproliferative effects of millimeter waves at some therapeutic frequencies on RPMI 7932 human skin malignant melanoma cells. Cell biochemistry and biophysics, 55, 25-32.

Benson VS, Pirie K, Schüz J et al. (2013). Mobile phone use and risk of brain neoplasms and other cancers: prospective study. Int J Epid, 42(3), 792-802.

Berg-Beckhoff G, Blettner M, Kowall B, Breckenkamp J, Schlehofer B, Schmiedel S, Bornkessel C, Reiss U, Potthoff P, Schuz J (2009). Mobile phone base stations and adverse health effects: phase 2 of a cross-sectional study with measured radio frequency electromagnetic fields. Occupational and Environmental Medicine, 66, 124-30.

Bernard N, Alberdi AJ, Tanguy ML, Brugere H, Helissey P, Hubert C, Gendrey N, Guillosson JJ, Nafziger J (2008). Assessing the potential leukemogenic effects of 50 Hz magnetic fields and their harmonics using an animal leukemia model. J Radiat Res, 49(6), 565-77.

Berns MW, Bewley W (1987). Inhibition of nucleic acid synthesis in cells exposed to 200 micrometers radiation from the free electron laser, Photochem Photobiol, 46, 65-7.

Berns M W, Bewley W., Sun CH, Templin P (1990). "Free electron laser irradiation at 200 microns affects DNA synthesis in living cells," Proc. Natl. Acad. Sci, 87(7), 2810–2.

Berns M W, Bewley W, Sun CH, Templin Karn AP (1994). Free electron laser irradiation at 200 microns inhibits DNA synthesis in living cells. Journal of Laser Applications, 6(7), 165–9.

Billaudel B, Taxile M, Ruffie G, Veyret B, Lagroye I (2009a). Effects of exposure to DAMPS and GSM signals on ornithine decarboxylase (ODC) activity: I. L-929 mouse fibroblasts. Int J Radiat Biol, 85(6), 510-8.

Billaudel B, Taxile M, Poulletier de Gannes F, Ruffie G, Lagroye I, Veyret B (2009b). Effects of exposure to DAMPS and GSM signals on ornithine decarboxylase (ODC) activity: II SH-SY5Y human neuroblastoma cells. Int J Radiat Biol, 85 (6), 519-22.

Blettner M, Schlehofer B, Breckenkamp J, Kowall B, Schmiedel S, Reis U, Potthoff P, Schuz J, Berg-Beckhoff G (2009). Mobile phone base stations and adverse health effects: phase 1 of a population-based, cross-sectional study in Germany. Occupational and Environmental Medicine, 66, 118-23.

Bock J, Fukuyo Y, Kang S, Phipps ML, Alexandrov LB, Rasmussen KØ, Bishop AR, Rosen ED, Martinez JS, Chen HT, Rodriguez G, Alexandrov BS, Usheva A (2010). Mammalian stem cells reprogramming in response to terahertz radiation. PLoS One, 5(12), e15806.

Bondar, NP, Kovalenko IL, Avgustinovich DF, Khamoyan, AG, Kudryavtseva NN (2008). Behavioral effect of terahertz waves in male mice. Bull Exp Biol Med, 145(4), 401–5.

Bortkiewicz A, Gadzicka E, Szyjkowska A, Politaski P, Mamrot P, Szymczak W, Zmyslony M (2012). Subjective complaints of people living near mobile phone base stations in Poland. International Journal of Occupational Medicine and Environmental Health 2012, 25, 31-40.

Bortkiewicz A, Gadzicka E, Szymczak W, Zmyślony M. (2012). Heart rate variability (HRV) analysis in radio and TV broadcasting stations workers. Int J Occup Med Environ Health, 25(4), 446-55.

Bouji M, Lecomte A, Hode Y, de Seze R, Villégier AS (2012). Effects of 900 MHz radiofrequency on corticosterone, emotional memory and neuroinflammation in middle-aged rats. Exp Gerontol. 47(6), 444-51.

Bourne N, Clothier RH, D'Arienzo M, Harrison P (2008). The effects of terahertz radiation on human keratinocyte primary cultures and neural cell cultures. Altern Lab Anim, 36(6), 667-84.

Boursianis A, Pantelis Vanias and Theodoros Samaras (2012).Measurements for assessing the exposure from 3G femtocells. Radiat Prot Dosimetry, 150 (2), 158-67.

Bourthoumieu S, Joubert V, Marin B, Collin A, Leveque P, Terro F, Yardin C (2010). Cytogenetic studies in human cells exposed in vitro to GSM-900 MHz radiofrequency radiation using R-banded karyotyping. Radiat Res, 174(6), 712-8.

Bourthoumieu S, Terro F, Leveque P, Collin A, Joubert V, Yardin C (2011). Aneuploidy studies in human cells exposed in vitro to GSM-900 MHz radiofrequency radiation using FISH., 87(4), 400-8.

Bourthoumieu S, Magnaudeix A, Terro F, Leveque P, Collin A, Yardin C (2013). Study of p53 Expression and Post-Transcriptional Modifications After GSM-900 Radiofrequency Exposure of Human Amniotic Cells. Bioelectromagnetics, 34, 52-60.

Boutry, CM, Kuehn S, Achermann P, Romann A, Keshvari J, Kuster N (2008). Dosimetric evaluation and comparison of different RF exposure apparatuses used in human volunteer studies. Bioelectromagnetics, 29(1), 11-9.

Brescia F, Sarti M, Massa R, Calabrese M, Sannino A, Scarfì MR (2009). Reactive oxygen species formation is not enhanced by exposure to UMTS 1950 MHz radiation and co-exposure to ferrous ions in Jurkat cells. Bioelectromagnetics, 30, 525-35.

Buckelmüller J, Landolt HP, Stassen HH, Achermann P (2006). Trait-like individual differences in the human sleep electroencephalogram. Neuroscience, 138(1), 351-6.

Buldak RJ, Polaniak R, Buldak L, Zwirska-Korczala K, Skonieczna M, Monsiol A, Kukla M, Dulawa-Buldak A, Birkner E (2012). Short-Term Exposure to 50 Hz ELF-EMF Alters the Cisplatin-Induced Oxidative Response in AT478 Murine Squamous Cell Carcinoma Cells. Bioelectromagnetics, 33 (8), 641–51.

Bunch KJ, Keegan TJ, Swanson J, Vincent TJ, Murphy MF (2014). Residential distance at birth from overhead high-voltage powerlines: childhood cancer risk in Britain 1962-2008. Br J Cancer, 110(5), 1402-8.

Byun YH, Ha M, Kwon HJ, Hong YC, Leem JH, Sakong J, Kim SY, Lee CG, Kang D, Choi HD, Kim N. (2013). Mobile phone use, blood lead levels, and attention deficit hyperactivity symptoms in children: a longitudinal study. PLoS One,8(3), e59742.

Campisi A, Gulino M, Acquaviva R, Bellia P, Raciti G, Grasso R, Musumeci F, Vanella A, Triglia A (2010) Reactive oxygen species levels and DNA fragmentation on astrocytes in primary culture after acute exposure to low intensity microwave electromagnetic field. , 473(1), 52-5.

Canseven AG, Keskil ZA, Keskil S, Seyhan N (2007). Pentylenetetrazol-induced seizures are not altered by pre- or post-drug exposure to a 50 Hz magnetic field. Int J Radiat Biol, 83(4), 231-5.

Canseven AG, Esmekaya MA, Kayhan H, Tuysuz MZ, Seyhan N (2014). Effects of microwave exposure and Gemcitabine treatment on apoptotic activity in Burkitt's lymphoma (Raji) cells. Electromagn Biol Med, [Epub ahead of print].

Cao Y, Zhang W, Lu MX, Xu Q, Meng QQ, Nie JH, Tong J. (2009). 900-MHz microwave radiation enhances gamma-ray adverse effects on SHG44 cells, 72(11-12), 727-32.

Cao Y, Xu Q, Jin Z-D, Zhang J, Lu M-X, Nie J, Tong J (2010). Effects of 900-MHz Microwave radiation on gamma-ray-induced damage to mouse hematopoietic system. J Toxicol Environ Health A, 73, 507–513.

Cao Y, Xu Q, Jin Z-D, Zhou Z, Nie J, Tong J (2011). Induction of adaptive response: pre-exposure of mice to 900 MHz radiofrequency fields reduces hematopoietic damage caused by subsequent exposure to ionizing radiation, Int. J. Radiat. Biol, 87, 720–8.

Capone F, Dileone M, Profice P, Pilato F, Musumeci G, Minicuci G, Ranieri F, Cadossi R, Setti S, Tonali PA, Di Lazarro V (2009). Does exposure to extremely low frequency magnetic fields produce functional changes in human brain? J Neural Transm, 116, 257-265.

Capstick M, McRobbie D, Hand J, Christ A, Kühn S, Hansson Mild K, Cabot E, Li Y, Melzer A, Papadaki A, Prüssmann K, Quest R, Rea M, Ryf S, Oberle M, Kuster N (2008). An investigation into occupational exposure to electromagnetic fields for personnel working with and around medical magnetic resonance imaging equipment. Report on Project VT/2007/017 of the European Commission, DG Employment, Social Affairs and Equal Opportunities http://www.myesr.org/html/img/pool/VT2007017FinalReportv04.pdf; http://www.myesr.org/html/img/pool/VT2007017FinalReportv04.pdf

Cardis E, Varsier N, Bowman J D, Deltour I, Figuerola J, Mann S, Moissonnier M, Taki M, Vecchia P, Villegas R, Vrijheid M, Wake K, Wiart J (2011a). Estimation of RF energy absorbed in the brain from mobile phones in the Interphone Study. Occup Environ Med, 68(9), 686-69

Cardis E, Armstrong BK, Bowman JD, Giles GG, Hours M, Krewski D, McBride M, Parent ME, Sadetzki S, Woodward A, Brown J, Chetrit A, Figuerola J, Hoffmann C, Jarus-Hakak A, Montestruq L, Nadon L, Richardson L, Villegas R, Vrijheid M. (2011b). Risk of brain tumours in relation to estimated RF dose from mobile phones: results from five Interphone countries. Occup Environ Med, 68(9), 631-40.

Carlberg M, Söderqvist F, Hansson Mild K, Hardell L (2013). Meningioma patients diagnosed 2007-2009 and the association with use of mobile and cordless phones: a case-control study. Environ Health, 12(1), 60.

Cavin ID, Glover PM, Bowtell RW, Gowland PA (2007). Thresholds for perceiving metallic taste at high magnetic field. J Magn Reson Imaging. 26(5), 1357-61.

Celik MS, Guven K, Akpolat V, Akdag MZ, Naziroglu M, Gul-Guven R, Celik MY, Erdogan S (2013). Extremely low-frequency magnetic field induces manganese accumulation in brain, kidney and liver of rats. Toxicol Ind Health, [Epub ahead of print].

Cervellati F, Franceschetti G, Lunghi L, Franzellitti S, Valbonesi P, Fabbri E, Biondi C, Vesce F (2009). Effect of high-frequency electromagnetic fields on trophoblastic connexins. Reproductive Toxicology, 28, 59–65.

Çetin H, Nazıroğlu M, Celik O, Yüksel M, Pastacı N and Ozkaya MO (2014). Liver antioxidant stores protect the brain from electromagnetic radiation (900 and 1800 MHz)-induced oxidative stress in rats during pregnancy and the development of offspring. J Matern Fetal Neonatal Med, 27(18), 1915-21.

Chahal H, Fung C, Kuhle S, Veugelers PJ (2013). Availability and night-time use of electronic entertainment and communication devices are associated with short sleep duration and obesity among Canadian children. Pediatr Obes, 8(1), 42-51.

Chakeres DW, Bornstein R, Kangarlu A (2003). Randomized comparison of cognitive function in humans at 0 and 8 Tesla. J Magn Reson Imaging, 18(3), 342-5.

Chaturvedi CM, Singh VP, P. Singh P, Basu P, Singaravel M (2011). 2.45GHz (CW) microwave irradiation alters circadian organization, spatial memory, DNA structure in the brain cells and blood cell counts of male mice, Mus musculus. Prog Electromagnetics Res B, 29, 23-42.

Chen WF, Qi H, Sun RG, Liu Y, Zhang K, Liu JQ (2010). Static magnetic fields enhanced the potency of cisplatin on k562 cells. Cancer Biother Radiopharm, 25(4), 401-8.

Chiampi M, Zilberti L (2011). Induction of electric field in human bodies moving near MRI: an efficient BEM computational procedure. IEEE Trans Biomed Eng, 58(10), 2787-93.

Chitanvis SM (2006). Can low-power electromagnetic radiation disrupt hydrogen bonds in dsDNA? J. Polym Sci: Part B: Polym Phis, 44, 2740-7.

Cho H, Seo YK, Yoon HH, Kim SC, Kim SM, Song KY, Park JK (2012). Neural stimulation on human bone marrow-derived mesenchymal stem cells by extremely low frequency electromagnetic fields (ELF-EMFs). Biotechnol Prog, 28(5), 1329-35.

Cho S, Lee Y, Lee S, Choi YJ, Chung HW (2014). Enhanced cytotoxic and genotoxic effects of gadolinium following ELF-EMF irradiation in human lymphocytes. Drug Chem Toxicol, 37, 440-7.

Choi SB, Kwon MK, Chung JW, Park JS, Chung K, Kim DW (2014). Effects of short-term radiation emitted by WCDMA mobile phones on teenagers and adults. BMC Public Health, 14, 438.

Cho SI, Nam YS, Chu LY, Lee JH, Bang JS, Kim HR, Kim HC, Lee YJ, Kim HD, Sul JD, Kim D, Chung YH, Jeong JH (2102). Extremely low-frequency magnetic fields modulate nitric oxide signaling in rat brain. Bioelectromagnetics, 33(7), 568-74.

Chou CK, Guy AW, Galambos R (1982). Auditory perception of radio-frequency electromagnetic fields. J of Acoustic Society of America, 71(6), 1321-34.

Choy JT, Brannigan RE (2012). Words of wisdom. Re: Use of laptop computers connected to Internet through Wi-Fi decreases human sperm motility and increases sperm DNA fragmentation. Eur Urol, 62(6), 1196-7.

Christ A, Guldimann R, Bühlmann B, Zefferer M, Bakker J F, van Rhoon G C, Kuster N (2012). Exposure of the Human Body to Professional and Domestic Induction Cooktops Compared to the Basic Restrictions. Bioelectromagnetics, 33(8), 695-705.

Christ A, Gosselin MC, Christopoulou M, Kuhn S, Kuster N (2010). Age-dependent tissue-specific exposure of cell phone users. Phys Med Biol, 55, 1767–83.

Christ A, Kainz W, Hahn EG, Honegger K, Zeffer M, Neufeld E, Rasher W, et al. (2010). The Virtual Family – Development of surface-based anatomical models of two adults and two children for dosimetric simulations. Phys Med Biol, 55, 23-38.

Chu LY, Lee JH, Nam YS, Lee YJ, Park WH, Lee BC, Kim D, Chung YH, Jeong JH (2011). Extremely low frequency magnetic field induces oxidative stress in mouse cerebellum. Gen Physiol Biophys, 30(4), 415-21.

Chung MK, Kim YB, Ha CS, Myung SH (2008). Lack of a co-promotion effect of 60 Hz rotating magnetic fields on N-ethyl-N-nitrosourea induced neurogenic tumors in F344 rats. Bioelectromagnetics, 29(7), 539-48.

Chung MK, Yu WJ, Kim YB, Myung SH (2010). Lack of a co-promotion effect of 60 Hz circularly polarized magnetic fields on spontaneous development of lymphoma in AKR mice. Bioelectromagnetics, 31(2), 130-9.

Clothier RH and Bourne N (2003). Effects of THz exposure on human primary keratinocyte differentiation and viability. Journal of Biological Physics, 29, 179–85.

Cohen SA, Klevens AI (2011). Use of capsule endoscopy in diagnosis and management of pediatric patients, based on meta-analysis. Clinical Gastroenterology and Hepatology, 9(6), 490-6.

Col-Araz N (2013). Evaluation of factors affecting birth weight and preterm birth in southern Turkey. J Pak Med Assoc, 63(4), 459-62.

Colletti V, Mandsala M, Manganotti P, Ramat S, Sacchetto l, Colletti L (2011). Intraoperative observation of changes in cochlear nerve action potentials durino exposure to electromagnetic fields generated by mobile phones. J Neurol Neurosurg Psychiatry, 82, 766-71.

Contessa GM, Falsaperla R, Brugaletta V, Rossi P (2010). Exposure to magnetic fields of railway engine drivers: a case study in Italy. Radiat Prot Dosimetry, 142(2-4), 160-7.

Consales C, Merla C, Marino C, Benassi B (2012). Electromagnetic fields, oxidative stress, and neurodegeneration. Int J Cell Biol, 683897.

Cooke R, Laing S, Swerdlow AJ (2010). A case-control study of risk of leukaemia in relation to mobile phone use. Br J Cancer, 103(11), 1729-35.

Cooper TG (2012). Comment on the morphology of spermatozoa in air-dried seminal smears. Int J Androl, 35(1), 105-6.

Corbacio M, Brown S, Dubois S, Goulet D, Prato FS, Thomas AW, Legros AS (2009). Human Cognitive Performance in a 3mT power-line frequency magnetic field. J Neural Transm, 116, 257-65.

Corbacio M, Brown S, Dubois S, Goulet D, Prato FS, Thomas AW, Legros AG (2011). Human Cognitive Performance in a 3 mT power-line frequency magnetic field. Bioelectromagnetics, 32(8), 620-33.

Coureau G, Bouvier G, Lebailly P, Fabbro-Peray P, Gruber A, Leffondre K, Guillamo JS, Loiseau H, Mathoulin-Pélissier S, Salamon R, Baldi I (2014). Mobile phone use and brain tumours in the CERENAT case-control study. Occup Environ Med, 71(7), 514-22.

Croft RJ, Leung S, McKenzie RJ, Loughran SP, Iskra S, Hamblin DL, Cooper NR (2010). Effects of 2G and 3G mobile phones on human alpha rhythms: resting EEG in adolescents, young adults and the elderly. Bioelectromagnetics, 31, 434-44.

Cuccurazzu B, Leone L, Podda MV, Piacentini R, Riccardi E, Ripoli C, Azzena GB, Grassi C (2010). Exposure to extremely low-frequency (50 Hz) electromagnetic fields enhances adult hippocampal neurogenesis in C57BL/6 mice. Exp Neurol, 226(1), 173-82.

Cui Y, Ge Z, Rizak JD, Zhai C, Zhou Z, Gong S, Che Y (2012). Deficits in water maze performance and oxidative stress in the hippocampus and striatum induced by extremely low frequency magnetic field exposure. PLoS One, 7(5), e32196.

Curcio G, Ferrara M, Limongi T, Tempesta D, Di Sante G, De Gennaro L, Quaresima V, Ferrari M. Acute mobile phones exposure affects frontal cortex hemodynamics as evidenced by functional near-infrared spectroscopy. Journal of Cerebral Blood Flow and Metabolism 2009; 29:903-910.

Curcio G, Nardo D, Perrucci MG, Pasqualetti P, Chen TL, Del Gratta C, Romani GL, Rossini PM (2012). Effects of mobile phone signals over BOLD response while performing a cognitive task. Clin Neurophysiol, 123(1), 129-36.

Cvetkovic D, Cosic I (2009). Alterations of human electroencephalographic activity caused by multiple extremely low frequency magnetic field exposures. Med Biol Eng Comput, 4, 1063-73.

Czerninski R, Zini A, Sgan-Cohen HD (2011). Risk of parotid malignant tumors in Israel (1970-2006). Epidemiology, 22(1), 130-1.

Danker-Hopfe H, Dorn H, Bornkessel C, Sauter C (2010). Do mobile phone base stations affect sleep of residents? Results from an experimental double-blind sham-controlled field study. American Journal of Human Biology, 22, 613-8.

Danker-Hopfe H, Dorn H, Bahr A, Anderer P, Sauter C (2011). Effects of electromagnetic fields emitted by mobile phones (GSM 900 and WCDMA/UMTS) on the macrostructure of sleep. J Sleep Res, 20, 73-81.

Das S, Kumar S, Jain S, Avelev VD, Mathur R (2012). Exposure to ELF- magnetic field promotes restoration of sensori-motor functions in adult rats with hemisection of thoracic spinal cord. Electromagn Biol Med, 31(3), 180-94.

Dasdag S, Taş M, Akdag MZ, Yegin K (2014). Effect of long-term exposure of 2.4□GHz radiofrequency radiation emitted from Wi-Fi equipment on testes functions. Electromagn Biol Med, [Epub ahead of print].

Dasdag S, Akdag MZ, Kizil G, Kizil M, Cakir DU, Yokus B (2012). Effect of 900 MHz radio frequency radiation on beta amyloid protein, protein carbonyl, and malondialdehyde in the brain. Electromagn Biol Med, 31(1), 67-74.

Davanipour Z, Tseng CC, Lee PJ, Markides KS, Sobel E (2014). Severe Cognitive Dysfunction and Occupational Extremely Low Frequency Magnetic Field Exposure among Elderly Mexican Americans. Br J Med Med Res, 4, 1641-62.

D'Arienzo M, Lee HJ, Harrison P, Pack JK (2008). The effects of terahertz radiation on human keratinocyte primary cultures and neural cell cultures. Altern Lab Anim.Gimm YM, 36(6), 667-84.

De Iuliis GN, Newey RJ, King BV, Aitken RJ (2009). Mobile Phone Radiation Induces Reactive Oxygen Species Production and DNA Damage in Human Spermatozoa In Vitro. PLoS ONE, 4(7), e6446.

De Marco M, Maggi S (2006). Evaluation of stray radiofrequency radiation emitted by electrosurgical devices. Physics in medicine and biology, 51(14), 3347.

De Tommaso M, Rossi P, Falsaperla R, Francesco Vde V, Santoro R, Federici A (2009). Mobile phones exposure induces changes of contingent negative variation. Neurosci Let; 464, 79-83.

De Vocht F, van-Wendel-de-Joode B, Engels H, Kromhout H (2003). Neurobehavioral effects among subjects exposed to high static and gradient magnetic fields from a 1.5 Tesla magnetic resonance imaging system--a case-crossover pilot study. Magn Reson Med, 50(4), 670-4.

De Vocht F, Stevens T, van Wendel-de-Joode B, Engels H, Kromhout H (2006a). Acute neurobehavioral effects of exposure to static magnetic fields: analyses of exposure-response relations. J Magn Reson Imaging, 23(3), 291-7.

De Vocht F, van Drooge H, Engels H, Kromhout H (2006b). Exposure, health complaints and cognitive performance among employees of an MRI scanners manufacturing department. J Magn Reson Imaging, 23(2), 197-204.

De Vocht F, Stevens T, Glover P, Sunderland A, Gowland P, Kromhout H (2007a) Cognitive effects of head-movements in stray fields generated by a 7 Tesla whole-body MRI magnet. Bioelectromagnetics, 28(4), 247-55.

De Vocht F, Glover P, Engels H, Kromhout H (2007b). Pooled analyses of effects on visual and visuomotor performance from exposure to magnetic stray fields from MRI scanners: application of the Bayesian framework. J Magn Reson Imaging, 26(5), 1255-60.

De Vocht F, Muller F, Engels H, Kromhout H (2009). Personal exposure to static and time-varying magnetic fields during MRI system test procedures. J Magn Reson Imaging, 30(5), 1223-8.

De Vocht F, Burstyn I, Cherrie JW (2011). Time trends (1998-2007) in brain cancer incidence rates in relation to mobile phone use in England. Bioelectromagnetics, 32(5), 334-9.

De Vocht F, Hannam K, Buchan I (2012). Environmental risk factors for cancer of the brain and nervous system. Occup Environ Med, 70, 349-356.

De Vocht F, Lee B (2014). Residential proximity to electromagnetic field sources and birth weight: Minimizing residual confounding using multiple imputation and propensity score matching. Environ Int, 69, 51-7.

De Vocht F, Hannam K, Baker P, Agius R (2014). Maternal residential proximity to sources of extremely low frequency electromagnetic fields and adverse birth outcomes in a UK cohort. Bioelectromagnetics, 35, 201-209.

Del Vecchio G, Giuliani A, Fernandez M, Mesirca P, Bersani F, Pinto R, Ardoino L, Lovisolo, GA, Giardino L, Calzà L (2009a). Continuous exposure to 900 MHz GSM-modulated EMF alters morphological maturation of neural cells. Neurosci Lett, 455(3), 173-7.

Del Vecchio G, Giuliani A, Fernandez M, Mesirca P, Bersan F, Pinto R, Ardoino L, Lovisolo GA, Giardino L, Calza L (2009b). Effect of radiofrequency electromagnetic field exposure on in vitro models of neurodegenerative disease. Bioelectromagnetics, 30(7), 564-72.

Deltour I, Wiart J, Taki M, Wake K, Varsier N, Mann S, Schüz J, Cardis E (2011). Analysis of three-dimensional SAR distributions emitted by mobile phones in an epidemiological perspective. Bioelectromagnetics, 32(8), 634-43.

Deltour I, Auvinen A, Feychting M, Johansen C, Klaeboe L, Sankila R, Schüz J (2012). Mobile Phone Use and Incidence of Glioma in the Nordic Countries 1979-2008: Consistency Check. Epidemiology, 23(2), 301–7.

Deng Y, Zhang Y, Jia S, Liu J, Liu Y, Xu W, Liu L (2013). Effects of aluminum and extremely low frequency electromagnetic radiation on oxidative stress and memory in brain of mice. Biol Trace Elem Res, 156(1-3), 243-52.

Di Loreto S, Falone S, Caracciolo V, Sebastiani P, D'Alessandro A, Mirabilio A, Zimmitti V, Amicarelli F (2009). Fifty hertz extremely low-frequency magnetic field exposure elicits redox and trophic response in rat-cortical neurons. J Cell Physiol, 219(2), 334-43.

Dimbylow P, Khalid M, Mann S (2003). Assessment of specific energy absorption rate (SAR) in the head from a TETRA handset. Physics in Medicine and Biology, 48(23), 3911-26.

Dini L, Panzarini E (2010). The influence of a 6 mT static magnetic field on apoptotic cell phagocytosis depends on monocyte/macrophage differentiation. Exp Biol Med, 235(12), 1432-41.

Divan HA, Kheifets L, Olsen J (2011). Prenatal cell phone use and developmental milestone delays among infants. Scand J Work Environ Health, 37(4), 341-8.

Divan HA, Kheifets L, Obel C, Olsen J. (2012). Prenatal cell phone use and developmental milestone delays among infants. J Epidemiol Community Health, 66(6), 524-9.

Dobroiu A, Otani C, and Kawase K (2006). Terahertz-wave sources and imaging applications. Measurement Science and Technology, 17(11), 161–74.

Does M, Scélo G, Metayer C, Selvin S, Kavet R, Buffler P. (2011). Exposure to electrical contact currents and the risk of childhood leukemia. Radiat Res, 175(3), 390-6.

Doré JF, Chignol MC (2012). Laptop computers with Wi-Fi decrease human sperm motility and increase sperm DNA fragmentation. Fertil Steril, 97(4); and author reply e13.

Dröge W (2002). Free radicals in the physiological control of cell function. Physiol Rev, 82, 47-95.

Duan Y, Zhang HZ, Bu RF (2011). Correlation between cellular phone use and epithelial parotid gland malignancies. Int J Oral Maxillofac Surg, 40(9), 966-72.

Duan Y, Wang Z, Zhang H, He Y, Lu R, Zhang R, Sun G and Sun X (2013). The preventive effect of lotus seedpod procyanidins on cognitive impairment and oxidative damage induced by extremely low frequency electromagnetic field exposure. Food Funct, 4(8), 1252-62.

Dwan K, Altman DG, Arnaiz JA, et al. (2008). Systematic review of the empirical evidence of study publication bias and outcome reporting bias. PLoS One, 3, e3081.

Editorial (2010). Should protocols for observational research be registered? Lancet, 375, 348.

Eger H, Jahn M (2010). Spezifische symptome und mobilfunkstrahlung in Selbitz (Bayern) – Evidenz fur eine dosiswirkungsbeziehung. Umwelt Medizin Gesellschaft. 23, 130-9.

Elder JA and Chou CK (2003). Auditory response to pulsed radiofrequency energy. Bioelectromagnetics, 24, 162–173.

Eleuteri AM, Amici M, Bonfili L, Cecarini V, Cucciolini M, Grimaldi S, Giuliani L, Angeletti, M, Fioretti E (2009). 50Hz Extremely Low Frequency Electromagnetic Fields Enhance Protein Carbonyl Groups Content in Cancer Cells: Effects on Proteasomal Systems. J Biomed Biotechnol.

Elliott P, Toledano MB, Bennett J, et al. (2010). Mobile phone base stations and early childhood cancers: case-control study. BMJ, 340, 3077.

Elliott P, Shaddick G, Douglass M, de Hoogh K, Briggs DJ, Toledano MB (2013). Adult cancers near high-voltage overhead power lines. Epidemiology, 24(2), 184-90.

Eltiti S, Wallace D, Ridgewell A, Zougkou K, Russo R, Sepulveda F, Fox E (2009). Short-term exposure to mobile phone base station signlas does not affect cognitive functioning or physiological measures in individuals who report sensitivity to electromagnetic fields and controls. Bioelectromagnetics, 30, 556-63.

EPRI (Electric Power Research Institute) 2010 Technical Report "An Investigation of Radiofrequency Fields Associated with the Itron Smart Meter".

Eser O, Songur A, Aktas C, Karavelioglu E, Caglar V, Aylak F, Ozguner F, Kanter M (2013). The effect of electromagnetic radiation on the rat brain: an experimental study. Turk Neurosurg, 23(6), 707-15.

Eshraghi AA, Gupta C, Ozdamar O, Balkany TJ, Truy E, Nazarian R (2012). Biomedical engineering principles of modern cochlear implants and recent surgical innovations. The Anatomical Record, 295(11), 1957-1966.

European Committee for Electrotechnical Standardization (CENELEC) (2008). Measurement methods for electromagnetic fields of household appliances and similar apparatus with regard to human exposure (Standard No. EN 62233:2008). Brussels, Belgium: CENELEC.

European Union. Directive 2008/46/EC of the European Parliament and of theCouncil of 23 April 2008. Amending Directive 2004/40/EC on minimum health and safety requirements regarding the exposure of workers to the risks arising from physical agents (electromagnetic fields) (18th individual Directive within the meaning of Article 16 (1) of Directive 89/391/EEC). Official Journal of the European Union 2008, L114/88.

Falzone N, Huyser C, Fourie F, Toivo T, Leszczynski D, Franken D (2008). In vitro effect of pulsed 900 MHz GSM radiation on mitochondrial membrane potential and motility of human spermatozoa. Bioelectromagnetics, 29(4), 268-76.

Falzone N, Huyser C, Becker P, Leszczynski D, Franken DR (2010a). The effect of pulsed 900-MHz GSM mobile phone radiation on the acrosome reaction, head morphometry and zona binding of human spermatozoa. Int J Androl, 34(1), 20-6.

Falzone N, Huyser C, Franken DR, Leszczynski D (2010b). Mobile phone radiation does not induce pro-apoptosis effects in human spermatozoa. Radiat Res, 174(2), 169-76.

Fang, Q (2010). Body EMF Absorption: A Design Issue for Implantable Medical Electronics. International Journal of Bioelectromagnetism, 12(1), 7-11.

Federici J F, Schulkin B, Huang F, Gary D, Barat R, Oliveira F, Zimdars D (2005). THz imaging and sensing for security applications—explosives, weapons and drugs. Semiconductor Science and Technology, 20(7), 266-80.

Federici J, and Moeller L (2010). Review of terahertz and subterahertz wireless communications. Journal of Applied Physics, 107(11), 111101.

Fedrowitz M, Hass R, Löscher W (2012). Effects of 50 Hz magnetic field exposure on the stress marker α-amylase in the rat mammary gland. Int J Radiat Biol, 88(7), 556-64.

Fedrowitz M, Löscher W (2012). Gene expression in the mammary gland tissue of female Fischer 344 and Lewis rats after magnetic field exposure (50 Hz, 100 μT) for 2 weeks. Int J Radiat Biol, 88(5), 425-9.

Feng J, Sheng H, Zhu C, Jiang H, Ma S (2013). Effect of adjuvant magnetic fields in radiotherapy on non-small-cell lung cancer cells in vitro. Biomed Res Int.

Feng SW, Lo YJ, Chang WJ, Lin CT, Lee SY, Abiko Y, Huang HM (2010). Static magnetic field exposure promotes differentiation of osteoblastic cells grown on the surface of a poly-L-lactide substrate. Med Biol Eng Comput, 48(8), 793-8.

Ferreri F, Curcio G, Pasqualetti P, De Gennaro L, Fini R, Rossini PM. Mobile phone emissions and human brain excitability. Ann Neurol, 60(2), 188-96.

Fiechter M, Stehli J, Fuchs TA, Dougoud S, Gaemperli O, Kaufmann PA. Impact of magnetic resonance imaging on human lymphocyte DNA integrity. Eur Heart J 2013;34, 2340–2345.

Figueroa Karlström E, Lundström R, Stensson O, Hansson Mild K (2006). Therapeutic staff exposure to magnetic field pulses during TMS/rTMS treatments. Bioelectromagnetics, 27(2), 156-8.

Findlay R P, Dimbylow P J (2010). SAR in a child voxel phantom from exposure to wireless computer networks (Wi-Fi). Physics in medicine and biology, 55(15), 405.

Fiocchi S, Markakis IA, Ravazzani P, Samaras T (2013). SAR exposure from UHF RFID reader in adult, child, pregnant woman, and fetus anatomical models. Bioelectromagnetics, 34(6), 443–52.

Fleming TR, DeMets DL. Surrogate end points in clinical trials: are we being misled? Annals of Internal Medicine 1996, 125(7), 605-613

Foroozandeh E, Derakhshan-Barjoei P, Jadidi M (2013). Toxic effects of 50 Hz electromagnetic field on memory consolidation in male and female mice. Toxicol Ind Health, 29(3), 293-9.

Foster KR (2007). Radiofrequency exposure from wireless LANs utilizing Wi-Fi technology. Health physics, 92(3), 280-9.

Foster KR, Tell RA (2013). Radiofrequency energy exposure from the Trilliant smart meter. Health Phys, 105(2), 177-86.

Fournier NM, Mach QH, Whissell PD, Persinger MA (2012). Neurodevelopmental anomalies of the hippocampus in rats exposed to weak intensity complex magnetic fields throughout gestation. Int J Dev Neurosci, 30, 427-33.

Fouquet NC, Hawken MB, Elliott P, Burgess AP (2013). TETRA mobile radios interfere with electroencephalography recording equipment. Med Eng Phys, 35(11), 1688-91.

Franco G, Perduri R, Murolo A (2008). Health effects of occupational exposure to static magnetic fields used in magnetic resonance imaging: a review. (Article in Italian), 99(1), 16-28.

Franzellitti, S., Valbonesi, P., Contin, A., Biondi, C., Fabbri, E (2008). HSP70 Expression in human trophoblast cells exposed to different 1.8 GHz mobile phone signals. Radiat Res, 170, 488–97.

Franzellitti S, Valbonesi P, Ciancaglini N, Biondi C, Contin A, Bersani F, Fabbri E. (2010). Transient DNA damage induced by high-frequency electromagnetic fields (GSM 1.8 GHz) in the human trophoblast HTR-8/SVneo cell line evaluated with the alkaline comet assay. Mutation Research, 683, 35–42.

Frei P, Poulsen AH, Johansen C, Olsen JH, Steding-Jessen M, Schüz J (2011). Use of mobile phones and risk of brain tumours: update of Danish cohort study. BMJ, 343, d6387.

Frei P, Mohler E, Braun-Fahrlander C, Frohlich J, Neubauer G, Roosli M (2012). Cohort study on the effects of everyday life radio frequency electromagnetic field exposure on non-specific symptoms and tinnitus. Environmental International, 38, 29-36.

Frei P, Poulsen AH, Mezei G, et al. (2013). Residential distance to high-voltage power lines and risk of neurodegenerative diseases: a Danish population-based case-control study. Am J Epidemiol, 177 (9), 970-8.

Freour T, Barriere P (2012). Wi-Fi decreases human sperm motility and increases sperm DNA fragmentation. Fertil Steril, 97(4), e14; and author reply e15.

Frilot C 2nd, Carrubba S, Marino AA (2009). Magnetosensory function in rats: localization using positron emission tomography. Synapse, 63(5), 421-8.

Frilot C 2nd, Carrubba S, Marino AA (2011). Transient and steady-state magnetic fields induce increased fluorodeoxyglucose uptake in the rat hindbrain. Synapse, 65(7), 617-23.

Fröhlich H (1968). Long range coherence and energy storage in biological systems Int J Quantum Chem, 2, 641-9.

Fröhlich H (1975). Extraordinary dielectric properties of biological materials and action of enzymes. Proc.Natl Acad Sci USA, 72, 4211-5.

Gajski G, Garaj-Vrhovac V (2009). Radioprotective effects of honeybee venom (Apis mellifera) against 915-MHz microwave radiation-induced DNA damage in wistar rat lymphocytes: in vitro study. Int J Toxicol, 28(2), 88-98.

Garip AI, Akan Z (2010). Effect of ELF-EMF on number of apoptotic cells; correlation with reactive oxygen species and hsp. Acta Biol Hung, 61, 158-67.

Gati A, Hadjem A, Wong M-F, Wiart J (2009). Exposure induced by WCDMA mobiles phones in operating networks. Wireless Communications. IEEE Transactions on, 8(12), 5723-7.

Gerner C, Haudek V, Schandl U, Bayer E, Gundacker N, Hutter HP, Mosgoeller W. (2010). Increased protein synthesis by cells exposed to a 1,800-MHz radio-frequency mobile phone electromagnetic field, detected by proteome profiling. Int Arch Occup Environ Health, 83(6), 691-702.

Ghanbari M, Mortazavi SB, Khavanin A and Khazaei M (2013). The Effects of Cell Phone Waves (900 MHz-GSM Band) on Sperm Parameters and Total Antioxidant Capacity in Rats. Int J Fertil Steril, 7(1), 21-8.

Ghosn R, Thuróczy G, Loos N, Brenet-Dufour V, Liabeuf S, de Seze R, Selmaoui B(2012). Effects of GSM 900 MHz on middle cerebral artery blood flow assessed by transcranial Doppler sonography. Radiat Res, 178(6), 543-50.

Ghovanloo M, Najafi K (2007). A wireless implantable multichannel microstimulating system-on-a-chip with modular architecture. Neural Systems and Rehabilitation Engineering, IEEE Transactions on, 15(3), 449-57.

Gilles M, Paslakis G, Heinrich A, Szostek A, Meyer P, Nees F, Rauschenberg J, Gröbner J, Krumm B, Semmler W, Flor H, Meyer-Lindenberg A, Deuschle M (2013) A cross-over study of effects on the hypothalamus-pituitary-adrenal (HPA) axis and the

sympathoadrenergic system in magnetic field strength exposure from 0 to 7 T. Stress, 16(2), 172-80.

Gioia L, Saponaro I, Bernabò N, Tettamanti E, Mattioli M, Barboni B (2013). Chronic exposure to a 2 mT static magnetic field affects the morphology, the metabolism and the function of in vitro cultured swine granulosa cells. Electromagn Biol Med, 32(4), 536-50.

Glover PM, Cavin I, Qian W, Bowtell R, Gowland PA (2007). Magnetic-field-induced vertigo: a theoretical and experimental investigation. Bioelectromagnetics, 28(5), 349-61.

Glover PM (2009). Interaction of MRI field gradients with the human body. Phys Med Biol, 54, 99-115.

Glover PM, Li Y, Antunes A, Mian OS, Day BL (2014). A dynamic model of the eye nystagmus response to high magnetic fields. Phys Med Biol, 59, 631-45.

Gobba F, Bianchi N, Verga P, Contessa GM, Rossi P (2012). Menometrorrhagia in magnetic resonance imaging operators with copper intrauterine contraceptive devices (IUDS): a case report. Int J Occup Med Environ Health. 25(1), 97-102.

Gomez-Perretta C, Navarro EA, Segura J, Portoles M (2013). Subjective symptoms related to GSM radiation from mobile phone base stations: A cross-sectional study. BMJ Open, 2, e003836.

Greenland S, Sheppard AR, Kaune WT, Poole C, Kelsh MA (2000). A pooled analysis of magnetic fields, wire codes, and childhood leukemia. Childhood Leukemia-EMF Study Group. Epidemiology, 11(6), 624-34.

Guidelines on Limits of Exposure to Laser Radiation of Wavelengths between 180 nm and 1 mm. (1996) Health Physics, 71 (5), 804-19.

Gulturk S, Demirkazik A, Kosar I, Cetin A, Dökmetas HS, Demir T (2010). Effect of exposure to 50 Hz magnetic field with or without insulin on blood-brain barrier permeability in streptozotocin-induced diabetic rats. Bioelectromagnetics, 31(4), 262-9.

Gutiérrez-Mercado YK, Cañedo-Dorantes L, Gómez-Pinedo U, Serrano-Luna G, Bañuelos-Pineda J and Feria-Velasco A (2013). Increased vascular permeability in the circumventricular organs of adult rat brain due to stimulation by extremely low frequency magnetic fields. Bioelectromagnetics, 34(2), 145-55.

Gutschi T, Mohamad Al-Ali B, Shamloul R, Pummer K, Trummer H (2011). Impact of cell phone use on men's semen parameters. Andrologia, 43(5), 312-6.

Guxens M, van Eijsden M, Vermeulen R, et al. (2013) Maternal cell phone and cordless phone use during pregnancy and behaviour problems in 5-year-old children. J Epidemiol Community Health, 67(5), 432-8.

Hadjem A, Conil E, Gati A, Wong MF and Wiart J (2010). Analysis of power absorbed by children's head as a result of new usages of mobile phones. IEEE Trans Electromagn Compat, 52(4), 812–9.

Halgamuge MN, Abeyrathne CD, Mendis P (2010). Measurement and analysis of electromagnetic fields from trams, trains and hybrid cars. Radiation Protection Dosimetry, 141(3), 255–68.

Halicka HD, Darzynkiewicz Z, Teodori L (2009). Attenuation of constitutive AT M activation and H2AX phosphorylation in human leukemic TK6 cells by their exposure to static magnetic field. Cell Cycle 8, 3238-40.

Hamnerius Y, Atefi S, Eslami A, Hopeson M,Khan A, Silva G, Estenberg J (2011). Distribution of ELF magnetic fields in Swedish dwellings. Proc. 2011 XXXth URSI General Assembly and Scientific Symposium Istanbul, 13-20 Aug 2011, doi: 10.1109/URSIGASS.2011.6051313.

Hansson Mild K (1981). Radiofrequency electromagnetic fields in Swedish radio stations and tall FM/TV towers. Bioelectromagnetics, 2, 61–9.

Hansson Mild K, Carlberg M, Wilén J, Hardell L (2005). How to combine the use of different mobile and cordles telephones in epidemiological studies on brain tumours? European Journal of Cancer prevention, 14, 285-8.

Hansson Mild K, Wilén J, Mattsson MO, Simko M (2009). Background ELF magnetic fields in incubators: A factor of importance in cell culture work. Cell Biology International, 33 (7), 755-7.

Hansson Mild K, Alanko T, Decat G, Falsaperla R, Gryz K, Hietanen M, Karpowicz J, Rossi P, Sandström M (2009). Exposure of workers to electromagnetic fields. A review of open questions on exposure assessment techniques. International Journal of Occupational Saferty and Ergonomics, 15, 3-33.

Hansson Mild K, Andersen JB, Pedersen GF (2012). Is there any exposure from a mobile phone in stand-by mode? Electromagn Biol Med, 31(1), 52-6.

Hansson Mild K, Hand J, Hietanen M, Gowland P, Karpowicz J, Keevil S, Lagroye I, van Rongen E, Scarfi MR, Wilén J (2012). Exposure classification of MRI workers in epidemiological studies. Bioelectromagnetics, 34(1), 81-4.

Hansteen IL, Clausen KO, Haugan V, Svendsen M, Svendsen MV, Eriksen JG, Skiaker R, Hauger E, Lågeide L, Vistnes AI, Kure EH (2009a). Cytogenetic effects of exposure to 2.3 GHz radiofrequency radiation on human lymphocytes in vitro. Anticancer Res, 29(11), 4323-30.

Hansteen IL, Lageidel, Clausen KO, Haugan V, Svendsen M, Eriksen JG, Skiaker R, Hauger E, Vistnes AI, Kure EH (2009b). Cytogenetic Effects of 18.0 and 16.5 GHz Microwave Radiation on Human Lymphocytes In Vitro. Anticancer Res, 29, 2885-92.

Hao D, Yang L, Chen S, Tong J, Tian Y, Su B, Wu S, Zeng Y (2012). Effects of long-term electromagnetic field exposure on spatial learning and memory in rats. Neurol Sci, 34(2), 157-64.

Hao Y, Yang X, Chen C, Yuan-Wang, Wang X, Li M, Yu Z (2010). STAT3 signalling pathway is involved in the activation of microglia induced by 245 GHz electromagnetic fields. Int J Radiat Biol, 86 (1), 27-36.

Harakawa S, Nedachi T, Hori T, Takahashi K, Tochio K, Inoue N (2008). Effect of electric field in conditioned aversion response. J Vet Med Sci, 70(6), 611-3.

Hardell L, Söderqvist F, Carlberg M, Zetterberg H, Hansson Mild K (2010). Exposure to wireless phone emissions and serum ß-trace protein. Int J Mol Med, 26, 301-6.

Hardell L, Carlberg M, Hansson Mild K, Eriksson M (2011). Case-control study on the use of mobile and cordless phones and the risk for malignant melanoma in the head and neck region. Pathophysiology, 18(4), 325-33.

Hardell L, Carlberg M (2012). Re: Mobile phone use and glioma risk: comparison of epidemiological study results with incidence trends in the United States. http://www.bmj.com/content/344/bmj.e1147/rr/578564.

Hardell L, Carlberg M (2013). Use of mobile and cordless phones and survival of patients with glioma. Neuroepidemiology, 40, 101–8.

Hardell L, Carlberg M, Hansson Mild K (2013). Use of mobile phones and cordless phones is associated with increased risk for glioma and acoustic neuroma. Pathophysiology; 20, 85-110.

Hardell L, Carlberg M, Söderqvist F, Mild KH (2013). Case-control study of the association between malignant brain tumours diagnosed between 2007 and 2009 and mobile and cordless phone use. Int J Oncol, 43(6), 1833-45.

Hardell L, Carlberg M, Söderqvist F, Mild KH (2013). Pooled analysis of case-control studies on acoustic neuroma diagnosed 1997-2003 and 2007-2009 and use of mobile and cordless phones. Int J Oncol, 43(4), 1036-44.

Hareuveny R, Eliyaho I, Luria R, Meiran N, Margaliot M (2011). Cognitive effects of cellular phones: a possible role of non-radiofrequency radiation factors. Bioelectromagnetics, 32, 585-8.

Hareuveny R, Kandel S, Yitzhak N M, Kheifets L, Mezei G (2011). Exposure to 50 Hz magnetic fields in apartment buildings with indoor transformer stations in Israel. Journal of exposure science & environmental epidemiology, 21(4), 365-71.

Hauri DD, Spycher B, Huss A, Zimmermann F, Grotzer M, von der Weid N, Spoerri A, Kuehni CE, Röösli M (2014). Swiss National Cohort; Swiss Paediatric Oncology Group. Exposure to radio-frequency electromagnetic fields from broadcast transmitters and risk of childhood cancer: a census-based cohort study. Am J Epidemiol, 179(7), 843-51.

Havas M, Marrongelle J (2013). Replication of heart rate variability provocation study with 2.4-GHz cordless phone confirms original findings. Electromagn Biol Med, 32(2), 253-66.

Havas M, Marrongelle J, Pollner B, Kelley E, Rees C, Tully L (2010). Provocation study using heart rate variability shows microwave radiation from 2.4 GHz cordless phone affects autonomic nervous system. Eur. J. Oncol. Library Vol. 5, 273-98.

He LH, Shi HM, Liu TT, Xu YC, Ye KP, Wang S (2011). Effects of extremely low frequency magnetic field on anxiety level and spatial memory of adult rats. Chin Med J, 124(20), 3362-6.

He YL, Liu DD, Fang YJ, Zhan XQ, Yao JJ, Mei YA (2013). Exposure to extremely low-frequency electromagnetic fields modulates Na+ currents in rat cerebellar granule cells through increase of AA/PGE2 and EP receptor-mediated cAMP/PKA pathway. PLoS One, 8(1), e54376.

Heilmayer C, Theysohn JM, Maderwald S, Kraff O, Ladd ME, Ladd SC (2011). A large-scale study on subjective perception of discomfort during 7 and 1.5 T MRI examinations. Bioelectromagetics, 32, 610-9.

Heinrich A, Szostek A, Meyer P, Nees F, Rauschenberg J, Gröbner J, Gilles M, Paslakis G, Deuschle M, Semmler W, Flor H (2013). Cognition and sensation in ultra-high static magnetic fields—a randomized case-crossover study with several scanners up to 7 Tesla. Radiology, 266(1), 236-45.

Heinrich S, Thomas S, Heumann C, von Kries R, Radon K (2010). Association between exposure to radiofrequency electromagnetic fields assessed by dosimetry and acute symptoms in children and adolescents: a population based cross-sectional study. Environmental Health, 9, 75.

Heinrich S, Thomas S, Heumann C, von Kries R, Radon K (2011). The impact of exposure to radio frequency electromagnetic fields on chronic well-being in young people – A cross-sectional study based on personal dosimetry. Environment International, 37, 26-30.

Hillert L (2004). Report on characterization, diagnosis and treatment In Hansson Mild K, Repacholi M, van Deventer E, Ravazzani P. Electromagnetic hypersensitivity: proceedings of an international workshop on EMF hypersensitivity. World Health Organization: Prague, Czech Republic.

Hillert L, Åkerstedt T, Lowden A, Wiholm C, Kuster N, Ebert S, Boutry C, Moffat SD, Berg M, Arnetz BB (2008). The effects of 884MHz GSM wireless communication signals on headache and other symptoms: An experimental provocation study. Bioelectromagnetics, 29, 185-196.

Hintzsche H, Jastrow C, Kleine-Ostmann T, Stopper H, Schmid E, Schrader T (2011). Terahertz radiation induces spindle disturbances in human-hamster hybrid cells, Radiat Res, 175(5), 569-74.

Hintzsche H, Jastrow C, Kleine-Ostmann T, Kärst U, Schrader T, Stopper H (2012). Terahertz electromagnetic fields (0.106 THz) do not induce manifest genomic damage in vitro, PLoS One, 7(9), e46397.

Hintzsche H, Jastrow C, Heinen B, Baaske K, Kleine-Ostmann T, Schwerdtfeger M, Shakfa MK, Kärst U, Koch M, Schrader T, Stopper H (2013). Terahertz Radiation at 0.380 THz and 2.520 THz Does Not Lead to DNA Damage in Skin Cells In Vitro. Radiat Res, 179(1), 38-45.

Hirata A, Kojima M, Kawai H, Yamashiro Y, Watanabe S, Sasaki H,Fujiwara O (2010). Acute Dosimetry and Estimation of Threshold-Inducing Behavioral Signs of Thermal Stress in Rabbits at 2.45-GHz Microwave Exposure. IEEE Transactions on Biomedical Engineering, 57(5),1234-42.

Hirose H, Sasaki A, Kaji N, Sakuma N, Sekijima M, Nojima T, Miyakoshi J (2008). Mobile phone base station radiation does not affect neoplastic transformation in BALB/3T3 cells. Bioelectromagnetics, 29, 55-64.

Hirose H, Sasaki A, Ishii N, Sekijima M, Iyama T, Nojima T, Ugawa Y (2010). 1950 MHz IMT-2000 field does not activate microglial cells in vitro. Bioelectromagnetics, 31(2), 104-12.

Hocking B, Hansson Mild K (2008). Guidance Note: Risk Management of Workers With Medical Electronic Devices and Metallic Implants in Electromagnetic Fields. International Journal of Occupational Safety and Ergonomics, 14 (2), 217–22.

Hong MN, Kim BC, Ko YG, Lee YS, Hong SC, Kim T, Pack JK, Choi HD, Kim N, Lee JS (2012). Effects of 837 and 1950 MHz radiofrequency radiation exposure alone or combined on oxidative stress in MCF10A cells. Bioelectromagnetics, 33, 604-11.

Hopewell S, Loudon K, Clarke MJ, Oxman AD, Dickersin K (2009). Publication bias in clinical trials due to statistical significance or direction of trial results. Cochrane Database Syst Rev, (1), MR000006.

Hountala CD, Maganioti AE, Papageorgiou CC, Nanou ED, Kyprianou MA, Tsiafakis VG, Rabavilas AD, Capsalis CN (2008). The spectral power coherence of the EEG under different EMF#mF conditions. Neuroscience Letters, 441, 188-92.

HPA, RCE-20 Health Effects from Radiofrequency Electromagnetic Fields: Report of the independent Advisory Group on Non-ionising Radiation, April 2012

Hug K, Grize L, Seidler A, Kaatsch P, Schüz J (2010). Parental occupational exposure to extremely low frequency magnetic fields and childhood cancer: a German case-control study. Am J Epidemiol, 171(1), 27-35.

Hutter H-P, Moshammer H, Wallner P, Cartellieri M, Denk-Linnet D-M, Katzinger M, Ehrenberger K, Kundi M (2010). Tinnitus and mobile phone use. Occup Environ Med, 67(12), 804-8.

Hwang Y, Ahn J Mun J Bae S Uk Jeong Y,Vinokurov NA and Kim P. In vivo analysis of THz wave irradiation induced acute inflammatory response in skin by laser scanning confocal microscopy. Optic Express 2014, 22 (10), 11465.

ICNIRP (International Commission on Non-Ionizing Radiation Protection) (1998). Guidelines for limiting exposure to time-vaying electric, magnetic, and electromagnetic fields (up to 300 GHz). Health Physics, 74(4), 494-522.

ICNIRP (International Commission on Non-Ionizing Radiation Protection) (2009). Guidelines on Limits of Exposure to Static Magnetic Fields. Health Physics, 96(4), 504-4.

ICNIRP (International Commission on Non-ionizing Radiation Protection) (2010). Guidelines for limiting exposure to time varying electric and magnetic fields (1 Hz to 100 kHz). Health Physics, 2010, 99(6), 818-36.

IEEE (Institute of Electrical and Electronics Engineers) (2002). IEEE standard for safety levels with respect to human exposure to electromagnetic fields, 0–3 kHz (Standard No. C.95.6:2002). New York, NY, USA.

Ilonen K., Markkanen A., Mezei G., and Juutilainen J (2008). Indoor Transformer Stations as Predictors of Residential ELF Magnetic Field Exposure. Bioelectromagnetics, 29(3), 213-8.

Ilvonen S, Sihvonen A-P, Kärkkäinen K, Sarvas J. (2005). Numerical assessment of induced ELF Currents in the human head due to the battery current of a digital mobile phone. Bioelectromagnetics, 26(8), 648-56.

Imai N, Kawabe M, Hikage T, Nojima T, Takahashi S, Shirai T (2011). Effects on rat testis of 1.95-GHz W-CDMA for IMT-2000 cellular phones. Syst Biol Reprod Med, 57(4), 204-9.

International Agency for Research on Cancer (2002). IARC Monographs on the Evaluation of Carcinogenic Risks to Humans. Volume 80. Non-Ionizing Radiation, Part 1: Static and Extremely Low-Frequency (ELF) Electric and Magnetic Fields. IARCpress, Lyon, France.

International Agency for Research on Cancer (2013) IARC Monographs on the Evaluation of Carcinogenic Risks to Humans. Volume 102. Radiofrequency Electromagnetic Fields. IARCpress, Lyon, France.

INTERPHONE Study Group (2010). Brain tumour risk in relation to mobile telephone use: results of the INTERPHONE international case-control study. Int J Epidemiol, 39(3), 675-94. Erratum in: Int J Epidemiol, 2012, 41(1), 328.

Ioannidis JP (2008). Why most discovered true associations are inflated? Epidemiology, 19, 640-8.

Israel M, Ivanova P, Ivanova M (2011). Electromagnetic fields and other physical factors around wind power generators (pilot study). The Environmentalist, 31(2), 161-8.

Jadidi M, Firoozabadi SM, Rashidy-Pour A, Sajadi AA, Sadeghi H, Taherian AA (2007). Acute exposure to a 50 Hz magnetic field impairs consolidation of spatial memory in rats. Neurobiol Learn Mem, 88(4), 387-92.

Janać B, Tovilović G, Tomić M, Prolić Z, Radenović L (2009). Effect of continuous exposure to alternating magnetic field (50 Hz, 0.5 mT) on serotonin and dopamine receptors activity in rat brain. Gen Physiol Biophys, Special Issue, 28, 41-6.

Janać B, Selaković V, Rauš S, Radenović L, Zrnić M, Prolić Z (2012). Temporal patterns of extremely low frequency magnetic field-induced motor behavior changes in Mongolian gerbils of different age. Int J Radiat Biol, 88(4), 359-66.

Jansen C, Wietzke S, Peters O, Scheller M, Vieweg N, Salhi M, Krumbholz N, Jördens C, Hochrein T, and Koch M (2010). Terahertz imaging: applications and perspectives. Applied Optics, 49(19), 48-59.

Jennings C, Chang G, Bell K (1993). PV EMF. Photovoltaic Specialists Conference, Conference Record of the Twenty Third IEEE , 1308-13.

Jennings C, Chang GJ, Reyes AB, Whitaker CM (1997). AC photovoltaic module magnetic fields. Photovoltaic Specialists Conference, Conference Record of the Twenty-Sixth IEEE, 1097-1100.

Jezek P, Hlavatá L (2005). Mitochondria in homeostasis of reactive oxygen species in cell, tissues, and organism. Int J Biochem Cell Biol, 37(12), 2478-503.

Jian W, Wei Z, Zhiqiang C, Zheng F (2009). X-Ray-Induced Apoptosis of BEL-7402 Cell Line Enhanced by Extremely Low Frequency Electromagnetic Field In Vitro. Bioelectromagnetics, 30, 163-5.

Jiang B, Nie J, Zhou Z, Zhang J, Tong J, Cao Y (2012). Adaptive Response in Mice Exposed to 900 MHz Radiofrequency Fields: Primary DNA Damage. PLoS ONE, 7(2), e32040.

Jiménez-García MN, Arellanes-Robledo J, Aparicio-Bautista DI, Rodríguez-Segura MA, Villa-Treviño S, Godina-Nava JJ (2010). Anti-proliferative effect of extremely low frequency electromagnetic field on preneoplastic lesions formation in the rat liver. BMC Cancer, 24(10), 159.

Jin YB, Choi HD, Kim BC, Pack JK, Kim N, Lee JS (2013). Effects of simultaneous combined exposure to CDMA and WCDMA electromagnetic fields on serum hormone levels in rats. J Radiat Res, 54(3), 430–7.

Jin YB, Lee HJ, Seon Lee J, Pack JK, Kim N, Lee YS (2011). One-year, simultaneous combined exposure of CDMA and WCDMA radiofrequency electromagnetic fields to rats. Int J Radiat Biol, 87(4), 416-23.

Jin YB, Kang GY, Lee JS, Choi J, Lee J-W, Hong S-C, Myung SH, Lee Y-S (2012). Effects on Micronuclei Formation of 60-Hz Electromagnetic Field Exposure with Ionizing Radiation, Hydrogen Peroxide, or c-Myc Overexpression. Int J Radiat Biol, 88, 374-80.

Jing J, Yuhua Z, Xiao-qian Y, Rongping J, Dong-mei G, Xi C (2012). The influence of microwave radiation from cellular phone on fetal rat brain. Electromagn Biol Med, 31(1), 57-66.

Jokela K, Puranen L, Sihvonen A-P. (2004). Assessment of the Magnetic Field Exposure Due to the Battery Current of Digital Mobile Phones. Health Physics, 86(1), 56-66.

Joosten S, Pammler K, Silny J (2009). The influence of anatomical and physiological parameters on the interference voltage at the input of unipolar cardiac pacemaker in low frequency electric fileds. Phys Med Biol, 54(3), 591-609.

Joseph P, Gaetano B, Bonato P (2008). Implantable Electronics. IEEE Pervasive Computing, 7(1), 12–3.

Joseph W, Verloock L, Martens L (2009). General Public Exposure by ELF Fields of 150–36/11-kV Substations in Urban Environment. IEEE Transactions on Power Delivery, 24(2), 642-9.

Joseph W, Frei P, Roösli M, Thuróczy G, Gajsek P, Trcek T, Bolte J, Vermeeren G, Mohler E, Juhász P, Finta V, Martens L (2010a). Comparison of personal radio frequency electromagnetic field exposure in different urban areas across Europe. Environmental Research, 110(7), 658-63.

Joseph W, Verloock L, Goeminne F, Vermeeren G, Martens L (2010b). Assessment of general public exposure to LTE and RF sources present in an urban environment. Bioelectromagnetics, 31(7), 576-9.

Joseph W, Verloock L, Goeminne F, Vermeeren G, Martens L (2012). Assessment of RF exposures from emerging wireless communication technologies in different environments. Health Physics, 102(2), 161.

Joseph W, Vermeeren G, Verloock L, Goeminne F (2012). In situ magnetic field exposure and ICNIRP-based safety distances for electronic article surveillance systems. Radiation Protection Dosimetry, 148(4), 420-7.

Joubert V, Bourthoumieu S, Leveque P, Yardin C (2008). Apoptosis is Induced by Radiofrequency Fields through the Caspase-Independent Mitochondrial Pathway in Cortical Neurons. Radiation Research, 169(1), 38-45.

Júnior LC, Guimarães ED, Musso CM, Stabler CT, Garcia RM, Mourão-Júnior CA, Andreazzi AE (2014). Behavior and memory evaluation of Wistar rats exposed to 1•8 GHz radiofrequency electromagnetic radiation. Neurol Res, 36(9), 800-3.

Jurewicz J, Radwan M, Sobala W, Ligocka D, Radwan P, Bochenek M and Hanke W (2014). Lifestyle and semen quality: role of modifiable risk factors. Syst Biol Reprod Med, 60(1), 43-51.

Juutilainen J. Bourne N (2005). Developmental effects of electromagnetic fields. Bioelectromagnetics, Suppl 7, 107-15. Review.

Kabacik S, Kirschenlohr H, Raffy C, Whitehill K, Coster M, Abe M, Brindle K, Badie C, Sienkiewicz Z, Bouffler S (2013). Investigation of transcriptional responses of juvenile mouse bone marrow to power frequency magnetic fields. Mutat Res, 745-746, 40-5.

Kahya MC, Nazıroğlu M and Çiğ B (2014). Selenium Reduces Mobile Phone (900 MHz)-Induced Oxidative Stress, Mitochondrial Function, and Apoptosis in Breast Cancer Cells. Biol Trace Elem Res, 160, 285-93.

Kane MJ, Breen P P, Quondamatteo F, ÓLaighin G (2011). BION microstimulators: A case study in the engineering of an electronic implantable medical device. Med Eng Phys, 33(1), 7-16.

Kang KA, Lee HC, Lee JJ, Hong MN, Park MJ, Lee YS, Choi HD, Kim N, Ko YG, Lee JS, (2014). Effects of combined radiofrequency radiation exposure on levels of reactive oxygen species in neuronal cells. J Radiat Res, 55(2), 265-76.

Karpowicz J, Gryz K, Politański P, Zmyślony M (2009). [Exposure to static magnetic field and health hazards during the operation of magnetic resonance scanners]. [Article in Polish] Med Pr, 62(3), 309-21.

Kaufman DW, Anderson TE, Issaragrisil S (2009). Risk factors for leukemia in Thailand. Ann Hematol, 88(11), 1079-88.

Kaune WT (2005). Comment on „designing EMF experiments: What is required to characterize 'exposure'?"Bioelectromagnetics, 16(6), 402–4.

Keegan TJ, Bunch KJ, Vincent TJ, King JC, O'Neill KA, Kendall GM, MacCarthy A, Fear NT, Murphy MF (2012). Case-control study of paternal occupation and childhood leukaemia in Great Britain, 1962-2006. Br J Cancer, 107(9), 1652-9.

Keikko T., Seesvuori R., and Valkealahti S. (2006) Exposure to magnetic field harmonics in the vicinity of indoor distribution substations. Health Physics 91(6): 574-581.

Kesari KK, Behari J (2010). Microwave exposure affecting reproductive system in male rats. Appl Biochem Biotechnol, 162(2), 416-28.

Khalid M, Mee T, Peyman A, Addison D, Calderon C, Maslanyj M, Mann S (2011). Exposure to radio frequency electromagnetic fields from wireless computer networks: Duty factors of Wi-Fi devices operating in schools. Progress in Biophysics and Molecular Biology, 107(3), 412-20.

Khan MM (2008). Adverse effects of excessive mobile phone use. Int J Occup Med Environ Health, 21, 289-93.

Kheifets L, Ahlbom A, Crespi CM, Draper G, Hagihara J, Lowenthal RM, Mezei G, Oksuzyan S, Schüz J, Swanson J, Tittarelli A, Vinceti M, Wunsch Filho V (2010a). Pooled analysis of recent studies on magnetic fields and childhood leukaemia. Br J Cancer, 103(7), 1128-35.

Kheifets L, Ahlbom A, Crespi CM, Feychting M, Johansen C, Monroe J, Murphy MF, Oksuzyan S, Preston-Martin S, Roman E, Saito T, Savitz D, Schüz J, Simpson J, Swanson J, Tynes T, Verkasalo P, Mezei G (2010b). A pooled analysis of extremely low-frequency magnetic fields and childhood brain tumors. Am J Epidemiol, 172(7), 752-61.

Khodarahmi I, Mobasheri H, Firouzi M (2010). The effect of 2.1 T static magnetic field on astrocyte viability and morphology. Magn Reson Imaging, 28(6), 903-9.

Kim DW, Choi JL, Nam KC, Yang DI, Kwon MK (2012). Origin of electromagnetic hypersensitivity to 60 Hz magnetic fields: A provocation study. Bioelectromagnetics, 33, 326-33.

Kim HJ, Jung J, Park JH, Kim JH, Ko KN, Kim CW (2013). Extremely low-frequency electromagnetic fields induce neural differentiation in bone marrow derived mesenchymal stem cells. Exp Biol Med (Maywood), 238(8), 923-31.

Kim HN, Han NK, Hong MN, Chi SG, Lee YS, Kim T, Pack JK, Choi HD, Kim N, Lee JS (2012). Analysis of the cellular stress response in MCF10A cells exposed to combined radio frequency radiation. J Radiat Res, 253(2), 176-83.

Kim HS, An YS, Paik MJ, Lee YS, Choi HD, Kim BC, Pack JK, Kim N, Ahn YH (2013). The effects of exposure to 915 MHz radiofrequency identification on cerebral glucose metabolism in rat: A [F-18] FDG micro-PET study. Int J Radiat Biol, 89(9), 750-5.

Kim JY, Hong SY, Lee YM, Yu SA, Koh WS, Hong JR, Son T, Chang SK, Lee M (2008). In vitro assessment of clastogenicity of mobile-phone radiation (835 MHz) using the alkaline comet assay and chromosomal aberration test. Environ Toxicol, 23, 319–27.

Kim KB, Byun HO, Han NK, Ko YG, Choi HD, Kim N, Pack JK, Lee JS (2010). Two-dimensional electrophoretic analysis of radio-frequency radiation-exposed MCF7 breast cancer cells. J Radiat Res, 51(2), 205-13.

Kim S, Im W (2010). Static magnetic fields inhibit proliferation and disperse subcellular localization of gamma complex protein3 in cultured C2C12 myoblast cells. Cell Biochem Biophys, 57(1), 1-8.

Kim SH, Song JE, Kim SR, Oh H, Gimm YM, Yoo DS, Pack JK, Lee YS (2004). Clothier RH. Teratological studies of prenatal exposure of mice to a 20 kHz sawtooth magnetic field., Bioelectromagnetics, 25(2), 114-7.

Kim SK, Choi JL, Kwon MK, Choi JY, Kim DW (2013). Effects of 60Hz magnetic fields on teenagers and adults. Environ Health, 12, 42.

Kirichuck VF, Ivanov AN, Antipova ON, Krenickiy AP, Mayborodin AV, Tupikin VD (2008). Sex-specific differences in changes of disturbed functional activity of platelets in albino rats under the effect of terahertz electromagnetic radiation at nitric oxide frequencies. Bull Exp Biol Med, 145(1), 75–7.

Kirichuk VF, Tsymbal AA (2009). Effects of Terahertz Irradiation at Nitric Oxide Frequencies on Intensity of Lipoperoxidation and Antioxidant Properties of the Blood under Stress Conditions. Bull Exp Biol Med, 148(2), 200–3.

Kirichuk VF, EfimovaN, Andronov E (2009). Effect of High Power Terahertz Irradiation on Platelet Aggregation and Behavioral Reactions of Albino Rats. Bull Exp Biol Med, 48(5), 746–9.

Kirichuk VF, Tsymbal AA (2009). Use of Terahertz Electromagnetic Waves for Correcting Hemostasis Functions. Biomedical Engineering, 44(1), 11–14.

Kirichuk VF, Ivanov AN, Kirijazi TS (2011). Correction of Microcirculatory Disturbances with THz electromagnetic radiation at nitric oxide frequencies in albino rates under conditions of acute stress. Bull Exp Biol Med, 151(3), 288-91.

Kirichuk VF, Velikanov VV, Velikanova TS, Antipova ON, Andronov EV, Ivanov AN, Parshina SS, Babichenko NE, Kiriyazi TS, Ponukalina EV, Smyshlyaeva IV, Tokaeva LK, Tsymbal AA (2012). Hemodynamic Changes Caused by Exposure of Animals with Acute Immobilization Stress to Continuous Terahertz Radiation with Frequencies equal to Absorption and Emission Frequencies of Nitrogen Oxide and Atmospheric Oxygen ISSN 2304-3415, Russian Open Medical Journal, 1, 0303.

Kirichuk VF, Tsymbal AA (2012) Effects of Terahertz Radiation at Atmospheric Oxygen Frequency of 129 GHz on blood nitrite concentrations under condition of different types

of stress against the background of administration of nonselective inhibitor of constitutive NO-syntases. Bull Exp Biol Med, 152(4), 435-8.

Kitaoka K, Kitamura M, Aoi S, Shimizu N and Yoshizaki K (2013). Chronic exposure to an extremely low-frequency magnetic field induces depression-like behavior and corticosterone secretion without enhancement of the hypothalamic-pituitary-adrenal axis in mice. Bioelectromagnetics, 34(1), 43-51. Plus Erratum in Bioelectromagnetics, 2013, 34(7), 562.

Kiyokawa T, Kikuchi K, Miyakoshi J (2009). Intermediate frequency magnetic fields generated by an induction heating (IH) cooktop do not affect genotoxicities and expression of heat shock proteins. Int J Radiat Biol, 85(10), 883-90.

Kjellström B, Linde C, Bennett T, Ohlsson Å, Ryden L (2004). Six years follow-up of an implanted SvO2 sensor in the right ventricle. European journal of heart failure, 6(5), 627-34.

Klueha U, Dorskya DI, Kreutzera DL (2005). Enhancement of implantable glucose sensor function in vivo using gene transfer-induced neovascularization. Biomaterials, 26(10), 1155–63.

Kooperberg C, Aragaki A, Strand AD, Olson JM (2005). Significance testing for small microarray experiments. Stat Med, 24, 2281-98.

Korenstein-Ilan A, Barbul A, Hasin P, Eliran A, Gover A, Korenstein R (2008). Terahertz radiation increases genomic instability in human lymphocytes, Radiat Res, 170(2), 224-34.

Korpinar MA, Kalkan MT, Tuncel H (2012). The 50 Hz (10 mT) sinusoidal magnetic field: effects on stress-related behavior of rats. Bratisl Lek Listy, 113(9), 521-4.

Korpinen L, Paakkonen R (2009). Mental symptoms and the use of new technical equipment. International Journal of Occupational Safety and Ergonomics, 15, 385-400.

Korpinen L, Kuisti H, Elovaara J, Virtanen V (2012). Cardiac pacemakers in electric and magnetic fields of 400-kV power lines. Pacing Clin Electrophysiol, 35(4), 422-30.

Korpinen L, Paakkonen R, Gobba F (2013). Self-reported neck symptoms and use of personal computers, laptops and cell phones among Finns aged 18-65. Ergonomics; 56(7), 1134-46.

Koteles F, Szemersky R, Gubanyi M, Kormendi J, Szekrenyesi C, Lloyd R, Molnar L, Drozdovsky O, Bardos G (2013). Idiopathic environmental intolerance attributed to electromagnetic fields (IEI-EMF) and electrosensibility (ES) – Are they connected? International Journal of Hygiene and Environment, 216, 362-70.

Kroll ME, Swanson J, Vincent TJ, Draper GJ (2010). Childhood cancer and magnetic fields from high-voltage power lines in England and Wales: a case-control study. Br J Cancer, 103(7), 1122-7.

Kubinyi G, Zeitler Z, Thuróczy G, Juhász P, Bakos J, Sinay H, László J (2010). Effects of homogeneous and inhomogeneous static magnetic fields combined with gamma radiation on DNA and DNA repair. Bioelectromagnetics, 31(6), 488-94.

Kucer N, Pamukcu T (2014). Self-reported symptoms associated with exposure to electromagnetic fields: A questionnaire study. Electromagn Biol Med, 33(1), 15-7.

Kühn S, Axel Kramer A, Urs Lott U, and Niels Kuster N (2007). Assessment of Human Exposure to Electromagnetic Radiation from Wireless Devices in Home and Office Environment. In M. Repacholi, E. van Deventer and P. Ravazzani (eds), Base Stations and Wireless Networks: Exposures and Health Consequences, World Health Organization, ISBN 978 92 4 159561 2.

Kühn S, Cabot E, Christ A, Capstick M, Kuster N (2009). Assessment of the radio-frequency electromagnetic fields induced in the human body from mobile phones used with hands-free kits. Physics in Medicine and Biology, 54(18), 5493-508.

Kühn S, Kelsh MA, Kuster N, Sheppard AR, Shum M (2013). Analysis of mobile phone design features affecting radiofrequency power absorbed in a human head phantom. Bioelectromagnetics, 34(6), 479-88.

Kuhnlein A, Heumann C, Thomas S, Heinrich S, Radon K (2009). Personal exposure to mobile communication networks and well-being in children – a statistical analysis based on a functional approach. Bioelectromagnetics, 30, 261-9.

Kumar G, Wood AW, Anderson V, McIntosh RL, Chen YY, McKenzie RJ (2011). Evaluation of hematopoietic system effects after in vitro radiofrequency radiation exposure in rats. Int J Radiat Biol, 87(2), 231-40.

Kumar S, Kesari KK, Behari J (2011a). Influence of microwave exposure on fertility of male rats. Fertil Steril, 95(4), 1500-2.

Kumar S, Kesari KK, Behari J (2011b). The therapeutic effect of a pulsed electromagnetic field on the reproductive patterns of male Wistar rats exposed to a 2.45-GHz microwave field, Clinics (Sao Paulo) 66(7), 1237-45.

Kumar S, Behari J, Sisodia R (2012). Impact of microwave at X-band in the aetiology of male infertility. Electromagn Biol Med, 31(3), 223-32.

Kumar S, Behari J and Sisodia R (2013). Influence of electromagnetic fields on reproductive system of male rats. Int J Radiat Biol, 89(3), 147-54.

Kumar S, Jain S, Velpandian T, Petrovich Gerasimenko Y, D Avelev V, Behari J, Behari M, Mathur R (2013). Exposure to extremely low-frequency magnetic field restores spinal cord injury-induced tonic pain and its related neurotransmitter concentration in the brain. Electromagn Biol Med, 32(4), 471-483.

Kunz SN, Grove N, Fischer F (2012). Acute pathophysiological influences of conducted electrical weapons in humans: A review of current literature. Forensic Science International, 221(1), 1-4.

Kuster N, Schönborn F (2000). Recommended minimal requirements and development guidelines for exposure setups of bio-experiments addressing the health risk concern of wireless communications. Bioelectromagnetics, 21, 508–14.

Kuster N, Schuderer J, Christ A, Futter P, Ebert S (2004). Guidance for exposure design of human studies addressing health risk evaluations of mobile phones. Bioelectromagnetics, 25(7), 524–9.

Kuster N, Torres VB, Nikoloski N, Frauscher M, Kainz W (2006). Methodology of detailed dosimetry and treatment of uncertainty and variations for in vivo studies; Bioelectromagnetics, 27(5), 378-91.

Kwon MK, Choi JY, Kim SK, Yoo TK, Kim DW (2012). Effects of radiation emitted by WCDMA mobile phones on electromagnetic hypersensitive subjects. Environmental Health, 11, 69.

Kwon MS, Koivisto M, Laine M, Hamalainen H (2008). Perception of the electromagnetic field emitted by a mobile phone. Bioelectromagnetics, 29(2), 154-9.

Kwon MS, Kujala T, Huotilainen M, Shestakova A, Näätänen R, Hämäläinen H (2009). Preattentive auditory information processing under exposure to the 920 NHz GSM mobile phone electromagnetic field: A mismatch negativity (MMN) stdy. Bioelectromagnetics, 30, 241-8.

Kwon MS, Kujala T, Huotilainen M, Shestakova A, Kujala T, Näätänen R, Hämäläinen H. (2010a). No effects of mobile phone use on cortical auditory change-detection in children: An ERP study. Bioelectromagnetics, 31, 191-9.

Kwon MS, Jääskeläinen SK, Toilvo T, Hämäläinen H (2010b). No effects of mobile phone electromagnetic field auditory brainstem response. Bioelectromagnetics, 31, 48-55.

Kwon MS, Vorobyev V, Kännälä S, Laine M, Rinne JO, Toivonen T, Johansson J, Teräs M, Lindholm H, Alanko T, Hämäläinen H (2011). GSM mobile phone radiation suppresses brain glucose metabolism. J Cereb Blood Flow Metab, 31(12), 2293-301.

Kwon MS, Vorobyev V, Kännälä S, Laine M, Rinne JO, Toivonen T, Johansson J, Teräs M, Joutsa J, Tuominen L, Lindholm H, Alanko T, Hämäläinen H (2012). No effects of short-term GSM mobile phone radiation on cerebral blood flow measured using positron emission tomography. Bioelectromagnetics,33(3), 247-56.

Lagroye I, Percherancier Y, Juutilainen J, De Gannes FP, Veyret B (2011). ELF magnetic fields: animal studies, mechanisms of action. Prog Biophys Mol Biol, 107, 369-73.

Laine C, Horton R, DeAngelis CD, Godlee F, Drazen JM, Frizelle FA, Haug C, Hebert PC, Kotzin S, Marusic A, Sahni P, Schroeder TV, Sox HC, Van Der Weyden MB, Verheugt FWA (2007). Clinical trial registration: Looking back and moving ahead. http://www.icmje.org/update_june07.html (Accessed 26 November 2012).

Lancet. Editorial. Should protocols for observational research be registered? Lancet 2010, 375, 348.

Landgrebe M, Frick U, Hauser S, Langguth B, Rosner R, Hajak G, Eichhammer P. (2008). Cognitive and neurobiological alterations in electromagnetic hypersensitive patients: results of a case-control study. Psychological Medicine, 38, 1781-91.

Larjavaara S, Schüz J, Swerdlow A, Feychting M, Johansen C, Lagorio S, Tynes T, Klaeboe L, Tonjer SR, Blettner M, Berg-Beckhoff G, Schlehofer B, Schoemaker M, Britton J, Mäntylä R, Lönn S, Ahlbom A, Flodmark O, Lilja A, Martini S, Rastelli E, Vidiri A, Kähärä V, Raitanen J, Heinävaara S, Auvinen A (2011). Location of gliomas in relation to mobile telephone use: a case-case and case-specular analysis. Am J Epidemiol, 174(1), 2-11.

Laudisi F, Sambucci M, Nasta F, Pinto R, Lodato R, Altavista P, Lovisolo GA, Marino C, Pioli C (2012). Prenatal exposure to radiofrequencies: effects of WiFi signals on thymocyte development and peripheral T cell compartment in an animal model. Bioelectromagnetics, 33(8), 652-61.

Lauer O, Frei P, Gosselin MC, Joseph W, Röösli M, Fröhlich J (2013). Combining near- and far-field exposure for an organ-specific and whole-body RF-EMF proxy for epidemiological research: a reference case. Bioelectromagnetics, 34(5), 366-74.

Le Quement C, Nicolaz CN, Zhadobov M, Desmots F, Sauleau R, Aubry M, Michel D, Le Drean Y (2012). Whole-Genome Expression Analysis in Primary Human Keratinocyte Cell Cultures Exposed to 60 GHz Radiation. Bioelectromagnetics, 33, 147-58.

Lee HJ, Lee JS, Pack JK, Choi HD, Kim N, Kim SH, Lee YS (2009). Lack of teratogenicity after combined exposure of pregnant mice to CDMA and WCDMA radiofrequency electromagnetic fields. Radiat Res, 172(5), 648-5.

Lee HJ, Pack JK, Gimm YM, Choi HD, Kim N, Kim SH, Lee YS (2009). Teratological evaluation of mouse foetuses exposed to a 20 kHz EMF. Bioelectromagnetics, 30(4), 330-3.

Lee HJ, Pack JK, Kim TH, Kim N, Choi SY, Lee JS, Kim SH, Lee YS (2010) The lack of histological changes of CDMA cellular phone-based radio frequency on rat testis. Bioelectromagnetics, 31(7), 528-34.

Lee HJ, Jin YB, Lee JS, Choi SY, Kim TH, Pack JK, Choi HD, Kim N, Lee YS (2011b) Lymphoma Development of Simultaneously Combined Exposureto Two Radiofrequency Signals in AKR/JMice. Bioelectromagnetics, 32, 485-92.

Lee HJ, Jin YB, Lee JS, Choi J, Lee JW, Myung SH, Lee YS (2012). Combined Effects of 60 Hz Electromagnetic Field Exposure With Various Stress Factors on Cellular Transformation in NIH3T3 Cells. Bioelectromagnetics, 33, 207-14.

Lee HJ, Jin HB, Kim TH, Pack JK, Kim N, Choi HD, Lee JS and Lee YS (2012). The Effects of Simultaneous Combined Exposure to CDMA and WCDMA Electromagnetic Fields on Rat Testicular Function. Bioelectromagnetics, 33, 356-64.

Lee JW, Kim MS, Kim YJ, Choi YJ, Lee Y, Chung HW (2011). Genotoxic Effects of 3 T Magnetic Resonance Imaging in Cultured Human Lymphocytes. Bioelectromagnetics, 32, 535-42.

Lee KJ, Kim BC, Han NK, Lee YS, Kim T, Yun JH, Kim N, Pack JK and Lee JS (2011a). Effects of Combined Radiofrequency Radiation Exposure on the Cell Cycle and Its Regulatory Proteins. Bioelectromagnetics, 32, 169-78.

Legros A, Corbacio M, Beuter A, Modolo J, Goulet D, Prato FS, Thomas AW (2011). Neurophysiological and behavioral effects of a 60Hz, 1,800 μT magnetic field in humans. European Journal of Applied Physiology, 112, 1751-62.

Lei T, Jing D, Xie K, Jiang M, Li F, Cai J, Wu X, Tang C, Xu Q, Liu J, Guo W, Shen G and Luo E (2013). Therapeutic effects of 15 Hz pulsed electromagnetic field on diabetic peripheral neuropathy in streptozotocin-treated rats. PLoS One, 8(4), e61414.

Leitgeb N, Cech R, Schröttner J, Lehofer P, Schmidpeter U, Rampetsreiter M (2008). Magnetic emission ranking of electrical appliances. A comprehensive market survey. Radiation Protection Dosimetry, 129(4), 439-45.

Leitgeb N, Schrottner J, Cech R, Kerbl R (2008). EMF-protection sleep study near mobile phone base stations. Somnologie, 12, 234-43.

Leitgeb N, Niedermayr F, Neubauer R, Loos G (2010). Numerically simulated cardiac exposure to electriccurrent densities induced by TASER X-26 pulses in adult men. Physics in Medicine and Biology, 55(20), 6187-95.

Leitgeb N, Omerspahic A, Niedermayr F (2010). Exposure of non-target tissues in medical diathermy. Bioelectromagnetics, 31(1), 12-9.

Leone L, Fusco S, Mastrodonato A, Piacentini R, Barbati SA, Zaffina S, Pani G, Podda MV and Grassi C (2014). Epigenetic modulation of adult hippocampal neurogenesis by extremely low-frequency electromagnetic fields. Mol Neurobiol, 49(3), 1472-86.

Lerchl A (2009). Comments on "Radiofrequency electromagnetic Welds (UMTS, 1,950 MHz) induce genotoxic effects in vitro in human fibroblasts but not in lymphocytes" by Schwarz et al. (Int Arch Occup Environ Health 2008: doi: 10.1007/s00420-008-0305-5). Int Arch Occup Environ Health, 82, 275–8.

Lerchl A, Wilhelm AFX (2010). Critical comments on DNA breakage by mobile-phone electromagnetic fields [Diem et al., Mutat Res, 583 (2005) 178–83]. Mutat Res, 697, 60–5.

Leung S, Croft RJ, McKenzie RJ, Iskra S, Silber B, Cooper N R, O'Neill B, Cropley V, Diaz-Trujillo A, Hamblin D, Simpson D (2011). Effects of 2G and 3G mobile phones on performance and electrophysiology in adolescents, young adults and older adults. Clinical Neurophysiology, 122(11), 2203-16.

Li CY, Liu CC, Chang YH, et al. (2012). A population-based case-control study of radiofrequency exposure in relation to childhood neoplasm. Sci Total Environ. 2012; 435, 472-8.

Li DK, Chen H, Odouli R (2011). Maternal exposure to magnetic fields during pregnancy in relation to the risk of asthma in offspring. Arch Pediatr Adolesc Med, 165(10), 945-50.

Li DK, Ferber JR, Odouli R, Quesenberry CP Jr (2012). A prospective study of in-utero exposure to magnetic fields and the risk of childhood obesity. Sci Rep, 2, 540.

Li P, McLaughlin J, Infante-Rivard C (2009). Maternal occupational exposure to extremely low frequency magnetic fields and the risk of brain cancer in the offspring. Cancer Causes Control, 20(6), 945-55.

Li Y, Zhang C, Song T (2014). Disturbance of the magnetic field did not affect spatial memory. Physiol Res, 63, 377-385.

Liao Z, Gao R, Xu C, Li ZS (2010). Indications and detection, completion, and retention rates of small-bowel capsule endoscopy: a systematic review. Gastrointestinal endoscopy, 71(2), 280-6.

Liljestrand B, Sandström M, Hansson Mild K (2003). RF exposure during use of electrosurgical units. Electromagnetic Biology and Medicine, 22(2-3), 127-32.

Lindholm H, Alanko T, Rintamäki H, Kännälä S, Toivonen T, Sistonen H, Tiikkaja M, Halonen J, Mäkinen T, Hietanen M (2011). Thermal effects of mobile phone RF fields on children: A provocation study. Prog Biophys Mol Biol, 107(3), 399-403.

Little MP, Rajaraman P, Curtis RE, Devesa SS, Inskip PD, Check DP, Linet MS (2012). Mobile phone use and glioma risk: comparison of epidemiological study results with incidence trends in the United States. BMJ, 344, e1147.

Liu K, Li Y, Zhang G, Liu J, Cao J, Ao L and Zhang S (2014). Association between mobile phone use and semen quality: a systemic review and meta-analysis. Andrology, 2(4), 491-501.

Liu K, Zhang G, Wang Z, Liu Y, Dong J, Dong X, Liu J, Cao J, Ao L, Zhang S (2014). The protective effect of autophagy on mouse spermatocyte derived cells exposure to 1800MHz radiofrequency electromagnetic radiation. Toxicol Lett, 228(3), 216-24.

Liu Y, Qi H, Sun RG, Chen WF (2011). An investigation into the combined effect of static magnetic fields and different anticancer drugs on K562 cell membranes. Tumori, 97(3), 386-92.

Liu YX, Tai JL, Li GQ, et al. (2012). Exposure to 1950-MHz TD-SCDMA electromagnetic fields affects the apoptosis of astrocytes via caspase-3-dependent pathway. PloS one, 7, e42332

Liu C, Duan W, Xu S, Chen C, He M, Zhang L, Yu Z, Zhou Z (2013). Exposure to 1800 MHz radiofrequency electromagnetic radiation induces oxidative DNA base damage in a mouse spermatocyte-derived cell line. Toxicol Lett, 218, 2-9.

Lolis MS, Goldberg DJ (2012) Radiofrequency in Cosmetic Dermatology: A Review. Dermatologic Surgery, 38(11), 1765–76.

Loos N, Thuróczy G, Ghosn R, Brenet-Dufour V, Liabeuf S, Selmaoui B, Libert JP, Bach V, Diouf M, de Seze R (2013). Is the effect of mobile phone radiofrequency waves on human skin perfusion non-thermal? Microcirculation, 20(7), 629-36.

Lopez Furelos A, Minana Maiques MM, Leiro JM, Rodriguez-Gonzalez JA, Ares-Pena FJ, Lopez-Martin E (2012). An experimental multi-frequency system for studying dosimetry and acute effects on cell and nuclear morphology in rat tissues. Progress In Electromagnetics Research, 129, 541-58.

Loughran SP, Wood A, Barton JM, Croft RJ, Thompson B, Stough C (2005). The effect of electromagnetic fields emitted by mobile phones on human sleep. Neuroreport, 16, 1973-6.

Loughran SP, McKenzie RJ, Jackson ML, Howard ME, Croft RJ (2012). Individual differences in the effects of mobile phone exposure on human sleep: Rethinking the problem. Bioelectromagnetics, 33, 86-93.

Loughran SP, Benz DC, Schmid MR, Murbach M, Kuster N, Achermann P (2013). No increased sensitivity in brain activity of adolescents exposed to mobile phone-like emissions. Clinical Neurophysiology, 124(7), 1303-8.

Lowden A, Åkerstedt T, Ingre M, Wiholm C, Hillert L, Kuster N, Nilsson JP, Arnetz B (2011). Sleep after mobile phone exposure in subjects with mobile phone-related symptoms. Bioelectromagnetics, 32, 4-14.

Lu M, Ueno S (2010). Dosimetry of typical transcranial magnetic stimulation devices. Journal of Applied Physics, 107, 09B316.

Lu Y, Xu S, He M, Chen C, Zhang L, Liu C, Chu F, Yu Z, Zhou Z, Zhong M. (2012). Glucose administration attenuates spatial memory deficits induced by chronic low-power-density microwave exposure. Physiol Behav, 106(5), 631-7.

Luria R, Eliyahu I, Hareuveny R, Margaliot M, Meiran N (2009). Cognitive effects of radiation emitted by cellular phones: the influence of exposure side and time. Bioelectromagnetics,30, 198-204.

Lustenberger C, Murbach M, Dürr R, Schmid MR, Kuster N, Achermann P, Huber R (2013). Stimulation of the brain with radiofrequency electromagnetic field pulses affects sleep-dependent performance improvement. Brain Stimul, 6(5), 805-11.

Luukanen A, Appleby R, Kemp M, and Salmon N (2013). Millimeter-Wave and Terahertz Imaging in Security Applications; 491-520. In K.-E. Peiponen et al. (eds.), Terahertz Spectroscopy and Imaging. Springer-Verlag.

Luukkonen J, Hakulinen P, Maki-Paakkanen J, Juutilainen J, Naarala J (2009). Enhancement of chemically induced reactive oxygen species production and DNA damage in human SH-SY5Y neuroblastoma cells by 872 MHz radiofrequency radiation, Mutat Res, 662, 54–8.

Luukkonen J, Juutilainen J, Naarala J. (2010). Combined effects of 872 MHz radiofrequency radiation and ferrous chloride on reactive oxygen species production and DNA damage in human SH-SY5Y neuroblastoma cells, Bioelectromagnetics, 31(6), 417-24.

Luukkonen J, Liimatainen A, Hoyto A, Juutilainen J, Naarala J (2011). Pre-Exposure to 50 Hz Magnetic Fields Modifies Menadione-Induced Genotoxic Effects in Human SH-SY5Y Neuroblastoma Cells. PLoS ONE, 6(3): e18021.

Luukkonen J, Liimatainen A, Juutilainen J, Naarala J (2014). Induction of genomic instability, oxidative processes, and mitochondrial activity by 50Hz magnetic fields in human SH-SY5Y neuroblastoma cells. Mutat Res Fundam Mol Mech Mutagen, 760, 33-41.

Lv B, Chen Z, Wu T, Shao Q, Yan D, Ma L5, Lu K, Xie Y (2014). The alteration of spontaneous low frequency oscillations caused by acute electromagnetic fields exposure Clin Neurophysiol, 125(2), 277-86.

Maaroufi K, Had-Aissouni L, Melon C, Sakly M, Abdelmelek H, Poucet B, Save E (2014). Spatial learning, monoamines and oxidative stress in rats exposed to 900 MHz electromagnetic field in combination with iron overload. Behav Brain Res, 258, 80– 89.

Maccà I, Scapellato ML, Carrieri M, di Bisceglie AP, Saia B, Bartolucci GB (2008). Occupational exposure to electromagnetic fields in physiotherapy departments. Radiation protection dosimetry, 128(2), 180-90.

Maestru C, Blanco M, Nevado A, Romero J, Rodriguez-Rubio P, Galindo J, Lorite JB, de las Morenas F, Fernandez-Arguelles P (2013). Reduction of pain thresholds in fibromyalgia after very low-intensity magnetic stimulation: A double-blinded, randomized placebo-controlled clinical trial. Pain Res Manag, 18(6), e101-6.

Maganioti AE, Hountala CD, Papageorgiou CC, Kyprianou MA, Rabavilas AD, Capsalis CN. (2010). Principal component analysis of the P600 waveform: RF and gender effects. Neurosci Lett, 478(1), 19-23.

Malagoli C, Crespi CM, Rodolfi R, et al. (2012). Maternal exposure to magnetic fields from high-voltage power lines and the risk of birth defects. Bioelectromagnetics, 33(5), 405-9.

Mandalà M, Colletti V, Sacchetto L, Manganotti P, Ramat S, Marcocci A, Colletti L (2014). Effect of Bluetooth headset and mobile phone electromagnetic fields on the human auditory nerve. Laryngoscope, 124(1), 255-9.

Manikonda PK, Rajendra P, Devendranath D, Gunasekaran B, Channakeshava, Aradhya SR, Sashidhar RB and Subramanyam C (2014). Extremely low frequency magnetic fields induce oxidative stress in rat brain. Gen Physiol Biophys, 33(1), 81-90.

Manti L, Braselmann H, Calabrese ML, Massa R, Pugliese M, Scampoli P, Sicignano G, Grossi G (2008). Effects of modulated microwave radiation at cellular telephone frequency (1.95 GHz) on X-ray-induced chromosome aberrations in human lymphocytes in vitro. Radiat Res, 169, 575–83.

Mantiply ED, Pohl KR, Poppell SW, Murph JA (1997). Summary of Measured Radiofrequency Electric and Magnetic Fields (10 kHz to 30 GHz) in the General and Work Environment. Bioelectromagnetics, 18, 563–77.

Marcantonio P, Del Re B, Franceschini A, Capri M, Lukas S, Bersani F, Giorgi G (2010). Synergic effect of retinoic acid and extremely low frequency magnetic field exposure on human neuroblastoma cell line BE(2)C. Bioelectromagnetics, 31(6), 425-33.

Marino AA, Carrubba S, McCarty DE (2012). Response to letter to the editor concerning "electromagnetic hypersensitivity: evidence for a novel neurological syndrome. International Journal of Neuroscience, 122, 402-3.

Marino C, Lagroye I, Scarfì MR, Sienkiewicz Z (2011). Are the young more sensitive than adults to the effects of radiofrequency fields? An examination of relevant data from cellular and animal studies. Progress in Biophysics & Molecular Biology, 107, 374-85.

Markkanen A; Naarala J; Juutilainen J (2010). A Study on the Effects of 50 Hz Magnetic Fields on UV-induced Radical Reactions in Murine Fibroblasts. J. Radiat. Res, 51, 609-13.

Markovà E, Malmgren LOG, Belyaev IY (2010). Microwaves from Mobile Phones Inhibit 53BP1 Focus Formation in Human Stem Cells More Strongly Than in Differentiated Cells: Possible Mechanistic Link to Cancer Risk. Environmental Health Perspectives, 118(3), 394-9.

Martin CJ, McCallum HM, Heaton B (1990). An evaluation of radiofrequency exposure from therapeutic diathermy equipment in the light of current recommendations. Clin Phys Physiol Meas, 11, 53-63.

Martínez MA, Úbeda A, Cid MA, Trillo MÁ (2012). The proliferative response of NB69 human neuroblastoma cells to a 50 Hz magnetic field is mediated by ERK1/2 signaling. Cell Physiol Biochem, 29(5-6), 675-86.

Martínez-Búrdalo M, Martin A, Sanchis A, Villar R (2009). FDTD assessment of human exposure to electromagnetic fields from WiFi and bluetooth devices in some operating situations. Bioelectromagnetics, 30(2), 142-51.

Martínez-Búrdalo M, Sanchis A, Martín A, Villar R (2010). Comparison of SAR and induced current densities in adults and children exposed to electromagnetic fields from electronic article surveillance devices. Physics in Medicine and Biology, 55(4), 1041-55.

Martínez-Sámano J, Torres-Durán PV, Juárez-Oropeza MA, Verdugo-Díaz L (2012). Effect of acute extremely low frequency electromagnetic field exposure on the antioxidant status and lipid levels in rat brain. Arch Med Res, 43(3), 183-9.

Martino CF, Perea H, Hopfner U, Ferguson VL, Wintermantel E (2010). Effects of weak static magnetic fields on endothelial cells. Bioelectromagnetics, 31(4), 296-301.

Martino CF (2011). Static magnetic field sensitivity of endothelial cells. Bioelectromagnetics, 32(6), 506-8.

Maskey D, Kim HJ, Kim HG, Kim MJ (2012). Calcium-binding proteins and GFAP immunoreactivity alterations in murine hippocampus after 1 month of exposure to 835 MHz radiofrequency at SAR values of 1.6 and 4.0 W/kg. Neurosci Lett, 506(2), 292-6.

Maskey D, Kim MJ (2014). Immunohistochemical Localization of Brain-derived Neurotrophic Factor and Glial Cell Line-derived Neurotrophic Factor in the Superior Olivary Complex of Mice after Radiofrequency Exposure. Neurosci Lett, 564, 78-82.

Maslanyj M, Simpson J, Roman E, Schüz J (2009). Power frequency magnetic fields and risk of childhood leukaemia: misclassification of exposure from the use of the 'distance from power line' exposure surrogate. Bioelectromagnetics, 30(3), 183-8.

Matthes R (1992). Radiation emission from microwave ovens. Journal of Radiological Protection, 12(3), 167-72.

Mattsson MO, Simkó M (2014). Grouping of Experimental Conditions as an Approach to Evaluate Effects of Extremely Low-Frequency Magnetic Fields on Oxidative Response in in vitro Studies. Front Public Health, 2, 132.

McCallum LC, Whitfield Aslund ML, Knopper LD, Ferguson GM, Ollson CA (2014). Measuring electromagnetic fields (EMF) around wind turbines in Canada: is there a human health concern? Environmental Health, 13, 9.

McCarty DE, Carrubba S, Chesson AL, Frilot C, Gonzalez-Toledo E, Marino A. (2011). Electromagnetic hypersensitivity: evidence for a novel neurological syndrome. International Journal of Neuroscience, 121, 670-6.

McNamee DA, Corbacio M, Weller JK, Brown S, Prato FS, Thomas AW, Legros AG (2010). The cardiovascular response to an acute 1800-microT, 60-Hz magnetic field exposure in humans. Int Arch Occup Environ Health, 83(4), 441-54.

McNamee DA, Corbacio M, Weller JK, Brown S, Stodilka RZ, Prato FS, Bureau Y, Thomas AW, Legros AG (2011). The response of the human circulatory system to an acute 200-μT, 60-Hz magnetic field exposure. Int Arch Occup Environ Health, 84, 267-77.

McRobbie DW (2012). Occupational exposure in MRI. Br J Radiol, 85(1012), 293-312.

Meng E, Hoang T (2012). MEMS-enabled implantable drug infusion pumps for laboratory animal research, preclinical, and clinical applications. Advanced Drug Delivery Reviews, 64(14), 1628–38.

Megha K, Deshmukh PS, Banerjee BD, Tripathi AK, Abegaonkar MP (2012). Microwave radiation induced oxidative stress, cognitive impairment and inflammation in brain of Fischer rats. Indian J Exp Biol, 50(12), 889-96.

Mian OS, Li Y, Antunes A, Glover PM, Day BL (2013). On the vertigo due to static magnetic fields. PLoS ONE, 8, e78748.

Milde-Busch A, von Kries R, Thomas S, Heinrich S, Straube A, Radon K (2010). The association between use of electronic media and prevalence of headaches in adolescents: results from a population-based cross-sectional study. BMC Neurology, 10, 12.

Milham S, Hatfield JB, Tell R (1999). Magnetic fields from steel-belted radial tires: implications for epidemiologic studies. Bioelectromagnetics, 20(7), 440-5.

Minagawa Y, Saito Y (2014). An analysis of the impact of cell phone use on depressive symptoms among Japanese elders. Gerontology, 60(6), 539-47.

Mizuno Y, Moriguchi Y, Hikage T, Terao Y, Ohnishi T, Nojima T, Ugawa Y (2009). Effects of W-CDMA 1950 MHz EMF emitted by mobile phones on regional cerebral blood flow in humans. Bioelectromagnetics, 30, 536-44.

Mohler E, Frei P, Braun-Fahrlander C, Frohlich J, Neubauer G, Roosli M (2010). Effects of everyday radiofrequency electromagnetic field exposure on sleep quality: A cross-sectional study. Radiation Research, 174, 347-56.

Mohler E, Frei P, Frohlich J, Braun-Fahrlander C, Roosli M (2012). Exposure to radiofrequency electromagnetic fields and sleep quality: A prospective cohort study. PLoS One, 7, e37455.

Møllerløkken OJ, Moen BE, Baste V, Magerøy N, Oftedal G, Neto E, Ersland L, Bjørge L, Torjesen PA, Hansson Mild K (2012). No effects of MRI scan on male reproduction hormones. Reprod Toxicol, 34(1), 133-9.

Monazzam HR, Hosseini M, Matin LF, Aghaei HA, Khosroabadi H, Hesami A (2014). Sleep quality and general health status of employees exposed to extremely low frequency magnetic fields in a petrochemical complex. J Environ Health Sci Eng, 12, 78.

Monzen S, Takahashi K, Toki T, Ito E, Sakurai T, Miyakoshi J, Kashiwakura I (2009). Exposure to a MRI-Type High-Strength Static Magnetic Field Stimulates Megakaryocytic/Erythroid Hematopoiesis in CD34+ Cells From Human Placental and Umbilical Cord Blood. Bioelectromagnetics, 30, 280-5.

Moquet J, Ainsbury E, Bouffler S, Lloyd D (2008). Exposure to low level GSM 935 MHZ radiofrequency fields does not induce apoptosis in proliferating or differentiated murine neuroblastoma cells. Radiat Prot Dosimetry, 131(3), 287-96.

Moretti D, Garenne A, Haro E, Poulletier de Gannes F, Lagroye I, Lévêque P, Veyret B, Lewis N (2013). In-vitro exposure of neuronal networks to the GSM-1800 signal. Bioelectromagnetics, 34(8), 571-8.

Mortazavi SM, Mahbudi A, Atefi M, Bagheri S, Bahaedini N, Besharati A (2011). An old issue and a new look: electromagnetic hypersensitivity caused by radiations emitted by GSM mobile phones. Technol Health Care, 19(6), 435-43.

Mueller ER (2003). Terahertz Radiation: Applications and Sources. The Industrial Physicist, 27-9, August/September 2003.

Murbach M, Christopoulou M, Crespo-Valero P, Achermann P, Kuster N (2012). Exposure system to study hypotheses of ELF and RF electromagnetic field interactions of mobile phones with the central nervous system. Bioelectromagnetics, 33, 527–33.

Murbach M, Neufeld E, Christopoulou M, Achermann P, Kuster N (2014). Modeling of EEG electrode artifacts and thermal ripples in human radiofrequency exposure studies. Bioelectromagnetics, 35(4), 273-83.

Nakamichi N, Ishioka Y, Hirai T, Ozawa S, Tachibana M, Nakamura N, Takarada T, Yoneda Y (2009). Possible Promotion of Neuronal Differentiation in Fetal Rat Brain Neural Progenitor Cells After Sustained Exposure to Static Magnetism. Journal of Neuroscience Research, 87, 2406–17.

Nakatani-Enomoto S, Furubayashi T, Ushiyama A, Groiss SJ, Ueshima K, Sokejima S, Simba AY, Wake K, Watanabe S, Nishikawa M, Miyawaki K, Taki M, Ugawa Y (2013). Effects of electromagnetic fields emitted from W-CDMA-like mobile phones on sleep in humans. Bioelectromagnetics, 34(8), 589-98.

Nam KC, Lee JH, Noh HW, Cha EJ, Kim NH, Kim DW (2009). Hypersensitivity to RF fields emitted from CDMA cellular phones: A provocation study. Bioelectromagnetics, 30, 641-50.

Navarro EA, Segura J, Portolés M, et al. (2003). The microwave syndrome: a preliminary study in Spain. Electromagn Biol Med;22, 161–9.

Nazıroğlu M, Çelik Ö, Özgül C, Çiğ B, Doğan S, Bal R, Gümral N, Rodríguez AB, Pariente JA (2012). Melatonin modulates wireless (2.45 GHz)-induced oxidative injury through TRPM2 and voltage gated Ca(2+) channels in brain and dorsal root ganglion in rat. Physiol Behav, 105(3), 683-92.

Negishi T, Imai S, Shibuya K, Nishimura I, Shigemitsu T. (2008). Lack of promotion effects of 50 Hz magnetic fields on 7,12-dimethylbenz(a)anthracene-induced malignant lymphoma/lymphatic leukemia in mice. Bioelectromagnetics, 29(1), 29-38.

Ni S, Yu Y, Zhang Y, Wu W, Lai K, Yao K. (2013) Study of oxidative stress in human lens epithelial cells exposed to 1.8 GHz radiofrequency fields. PLoS One 2013 Aug 26;8(8):e72370. doi: 10.1371/journal.pone.0072370

Nicolaz CN, Zhadobov M, Desmots F, Ansart A, Sauleau R, Thouroude D, Michel D, Le Drean Y (2009). Study of narrow band millimeter-wave potential interactions with endoplasmic reticulum stress sensor genes. Bioelectromagnetics, 30(5), 365-73.

Nieto-Hernandez R, Williams J, Cleare AJ, Landau S, Wessely S, Rubin GJ (2011). Can exposure to a terrestrial trunked radio (TETRA)-like signal cause symptoms? A randomised double-blind provocation study. Occup Environ Med, 68, 339-44.

Nishimura I, Imai S, Negishi T (2009). Lack of chick embryotoxicity after 20 kHz, 1.1 mT magnetic field exposure. Bioelectromagnetics, 30(7), 573-82.

Nishimura I, Oshima A, Shibuya K, Negishi T (2011). Lack of teratological effects in rats exposed to 20 or 60 kHz magnetic fields. Birth Defects Res B Dev Reprod Toxicol, 92(5), 469-77.

Nishimura I, Oshima A, Shibuya K, Mitani T, Negishi T (2012). Absence of reproductive and developmental toxicity in rats following exposure to a 20-kHz or 60-kHzmagnetic field. Regul Toxicol Pharmacol, 64(3), 394-401.

Nordenson I, Hansson Mild K, Järventaus H, Hirvonen A, Sandström M, Wilén J, Blix N, Norppa H. Chromosomal aberrations in peripheral lymphocytes of train engine drivers. Bioelectromagnetics. 2001 Jul;22(5):306-15.

Novikov VV, Novikov GV, Fesenko EE (2009). Effect of Weak Combined Static and Extremely Low-frequency Alternating Magnetic Fields on Tumor Growth in Mice Inoculated With the Ehrlich Ascites Carcinoma. Bioelectromagnetics, 30, 343-51.

Ntzouni MP, Skouroliakou A, Kostomitsopoulos N, Margaritis LH (2013). Transient and cumulative memory impairments induced by GSM 1.8 GHz cell phone signal in a mouse model. Electromagn Biol Med, 32(1), 95-120.

Nylund R, Tammio H, Kuster N, Leszczynski D (2009). Proteomic analysis of the response of human endothelial cell line EA.hy926 to 1800 GSM mobile phone radiation. DOI: 10.4172/jpb.1000105.

Nylund R, Kuster N, Leszczynski D (2010). Analysis of proteome response to the mobile phone radiation in two types of human primary endothelial cells. Proteome Science, 8, 52.

Ogawa K, Nabae K, Wang J, Wake K, Watanabe S, Kawabe M, Fujiwara O, Takahashi S, Ichihara T, Tamano S, Shirai T (2009). Effects of gestational exposure to 1.95-GHz W-CDMA signals for IMT-2000 cellular phones: Lack of embryotoxicity and teratogenicity in rats. Bioelectromagnetics, 30(3), 205-12.

O'Handley RC, Huang JK, Bono DC, Simon J (2008). Improved Wireless, Transcutaneous Power Transmission for In Vivo Applications. Sensors Journal, IEEE, 8(1), 57-62.

Ong JM, da Cruz L (2011). The bionic eye: a review. Clinical & Experimental Ophthalmology, 40(1), 6-17.

Osseiran A, Boccardi F, Braun V, Kusume K, Marsch P, Maternia M et al. (2014). Scenarios for 5G mobile and wireless communications: the vision of the METIS project. IEEE Communications Magazine, 52(5), 26-35.

Ostrovskiy NV, et al. (2005) Application of the terahertz waves in therapy of burn wounds. in Infrared and Millimeter Waves and 13th International Conference on Terahertz Electronics, IRMMW-THz 2005.

Ozgur E, Guler G, Kismali G, Seyhan N (2014). Mobile Phone Radiation Alters Proliferation of Hepatocarcinoma Cells. Cell Biochem Biophys, 70(2), 983-91.

Ozlem Nisbet H, Nisbet C, Akar A, Cevik M, Karayigit MO (2012). Effects of exposure to electromagnetic field (1.8/0.9 GHz) on testicular function and structure in growing rats. Res Vet Sci, 93(2), 1001-5.

Özorak A, Nazıroğlu M, Çelik Ö, Yüksel M, Özçelik D, Özkaya MO, Çetin H, Kahya MC and Kose SA (2013). Wi-Fi (2.45 GHz) - and mobile phone (900 and 1800 MHz)-induced risks on oxidative stress and elements in kidney and testis of rats during pregnancy and the development of offspring. Biol Trace Elem Res, 156(1-3), 221-9.

Paffi A, Apollonio F, Lovisolo GA, Marino C, Pinto R, Repacholi M, Liberti M (2010). Considerations for Developing an RF Exposure System: A Review for in vitro Biological Experiments. Microwave Theory and Techniques, IEEE Transactions on, 58(10), 2702 - 14.

Paffi A, Merla C, Pinto R, Lovisolo GA, Liberti M, Marino C, Repacholi M, Apollonio F (2013). Microwave Exposure Systems for In Vivo Biological Experiments: A Systematic Review. Microwave Theory and Techniques, IEEE Transactions on, 61(5), 1980-93.

Pall ML (2013). Electromagnetic fields act via activation of voltage-gated calcium channels to produce beneficial or adverse effects. J Cell Mol Med, 17(8), 958-65.

Palumbo R, Brescia F, Capasso D, Sannino A, Sarti M, Capri M, Grassilli E, Scarfì MR (2008). Exposure to 900 MHz radiofrequency radiation induces caspase-3 activation in proliferating human lymphocytes. Radiat Res, 170, 327-34.

Papageorgiou CC, Hountala CD, Maganioti AE, Kyprianou MA, Rabavilas AD, Papadimitriou GN, Capsalis CN (2011). Effects of Wi-Fi signals on the P300 component of event-related potentials during an auditory Hayling task. J Integr Neurosci 10, 189-202.

Paradiso J, Gaetano B, Bonato P (2008). Implantable Electronics, IEEE Pervasive Computing, 7(1), 12–13.

Parazzini M, Ravazzani P, Tognola G, Thuróczy G, Molnar FB, Sacchettini A, Ardesi G, Mainardi LT (2007). Electromagnetic fields produced by GSM cellular phones and heart rate variability. Bioelectromagnetics, 28(2), 122-9.

Parazzini M, Lutman ME, Moulin A, Barnel C, Sliwinska-Kowalska M, Zmyslony M, Hernadi I, Stefanics G, Thuroczy G, Ravazzani P (2010). Absence of short-term effects of UMTS exposure on the human auditory system. Radiat Res, 173(1), 91-7.

Parazzini M, Ravazzani P, Thuroczy G, Molnar FB, Ardesi G, Sacchettini A, Mainardi LT. (2013). Nonlinear heart rate variability measures under electromagnetic fields produced by GSM cellular phones. Electromagn Biol Med, 32(2), 173-81.

Parlett LE, Bowman JD, van Wijngaarden E (2011). Evaluation of occupational exposure to magnetic fields and motor neuron disease mortality in a population-based cohort. J Occup Environ Med, 53(12), 1447-51.

Patel M, Williamsom RA, Dorevitch S, Buchanan S (2008). Pilot study investigating the effect of the static magnetic field from a 9.4T MRI on the vestibular system. J Occup Environ Med, 50, 576-83.

Paulraj R, Behari J (2011). Effects of low level microwave radiation on carcinogenesis in Swiss Albino mice. Mol Cell Biochem, 348, 191–7.

Pedersen C, Bräuner EV, Rod NH, Albieri V, Andersen CE, Ulbak K, Hertel O, Johansen C, Schüz J, Raaschou-Nielsen O (2014). Distance to high-voltage power lines and risk of childhood leukemia - an analysis of confounding by and interaction with other potential risk factors. PLoS One, 9(9), e107096.

Pedersen C, Raaschou-Nielsen O, Rod NH, Frei P, Poulsen AH, Johansen C, Schüz J (2014). Distance from residence to power line and risk of childhood leukemia: a population-based case-control study in Denmark. Cancer Causes Control, 25(2), 171-7.

Pelletier A, Delanaud S, Décima P, Thuroczy G, de Seze R, Cerri M, Bach V, Libert JP, Loos N (2013). Effects of chronic exposure to radiofrequency electromagnetic fields on energy balance in developing rats. Environ Sci Pollut Res Int, 20(5), 2735-46.

Pelletier A, Delanaud S, de Seze R, Bach V, Libert JP, Loos N (2014). Does exposure to a radiofrequency electromagnetic field modify thermal preference in juvenile rats? PLoS One, 9(6), e99007.

Perentos N, Iskra S, McKenzie R, Cosic I (2007). Characterization of pulsed ELF magnetic fields generated by GSM mobile phone handsets. IFMBE Proceedings, 14, 2706-9.

Perentos N, Croft RJ, McKenzie RJ, Cvetkovic D, Cosic I (2008). The effect of GSM-like ELF radiation on the alpha band of the human resting EEG. Conf Proc IEEE, Eng Med Biol Soc, 5680-3.

Perentos N, Croft RJ, McKenzie RJ, Cosic I (2013). The alpha band of the resting electroencephalogram under pulsed and continuous radio frequency exposures. IEEE Trans Biomed Eng, 60(6), 1702-10.

Perez FP, Zhou X, Morisaki J, Jurivich D (2008). Electromagnetic field therapy delays cellular senescence and death by enhancement of the heat shock response. Exp Gerontol, 43(4), 307-16.

Persson T, Törnevik C, Larsson LE, Lovén J (2012). Output power distributions of terminals in a 3G mobile communication network, Bioelectromagnetics, 33(4) 320-5.

Pettersson D, Mathiesen T, Prochazka M, Bergenheim T, Florentzson R, Harder H, Nyberg G, Siesjö P, Feychting M (2014). Long-term mobile phone use and acoustic neuroma risk. Epidemiology, 25, 233-41.

Peyman A, Khalid M, Calderon C, Addison D, Mee T, Maslanyj M, Mann S (2010). Assessment of exposure to electromagnetic fields from wireless computer networks (wi-fi) in schools; results of laboratory measurements. Health Phys, 100(6), 594-612.

Piacentini R, Ripoli C, Mezzogori D, Azzena GB, Grassi C (2008). Extremely low-frequency electromagnetic fields promote in vitro neurogenesis via upregulation of Ca(v)1-channel activity. J Cell Physiol, 215(1), 129-39.

Podda MV, Leone L, Barbati SA, Mastrodonato A, Li Puma DD, Piacentini R, Grassi C (2014). Extremely low-frequency electromagnetic fields enhance the survival of newborn neurons in the mouse hippocampus. Eur J Neurosci, 39(6), 893-903.

Polidori E, Zeppa S, Potenza L, Martinelli C, Colombo E, Casadei L, Agostini D, Sestili P, Stocchi V (2012). Gene expression profile in cultured human umbilical vein endothelial cells exposed to a 300μmT static magnetic field. Bioelectromagnetics, 33(1), 65-74.

Politański P, Rajkowska E, Brodecki M, Bednarek A, Zmyślony M (2013). Combined effect of X-ray radiation and static magnetic fields on reactive oxygen species in rat lymphocytes in vitro. Bioelectromagnetics, 34(4), 333-6.

Poniedziałek B, Rzymski P, Karczewski J, Jaroszyk F, Wiktorowicz K (2013). Reactive oxygen species (ROS) production in human peripheral blood neutrophils exposed in vitro to static magnetic fields. Electromagn Biol Med, 32(4), 560-8.

Portelli LA, Schomay TE, Barnes FS (2013). Inhomogeneous background magnetic field in biological incubators is a potential confounder for experimental variability and reproducibility. Bioelectromagnetics, Jul;34(5), 337-48.

Potenza L, Martinelli C, Polidori E, Zeppa S, Calcabrini C, Stocchi L, Sestili P, Stocchi V (2010). Effects of a 300 mT static magnetic field on human umbilical vein endothelial cells. Bioelectromagnetics, 31(8), 630-9.

Poulletier de Gannes F, Haro E, Hurtier A, Taxile M, Ruffié G, Billaudel B, Veyret B, Lagroye I (2011). Effect of exposure to the EDGE signal on oxidative stress in brain cell model. Radiat Res, 175, 225–30.

Poulletier de Gannes F, Haro E, Hurtier A, Taxile M, Athane A, Ait-Aissa S, Masuda H, Percherncier Y, Ruffié G, Billaudel B, Dufour P, Veyret B, Lagroye I (2012). Effect of in

utero wi-fi exposure on the pre- and postnatal development of rats. Birth Defects Research (Part B), 95(2), 130-6.

Poulletier de Gannes F, Billaudel B, Haro E, Taxile M, Le Montagner L, Hurtier A, Ait Aissa S, Masuda H, Percherancier Y, Ruffié G, Dufour P, Veyret B and Lagroye I (2013). Rat fertility and embryo fetal development: influence of exposure to the Wi-Fi signal. Reprod Toxicol, 36, 1-5.

Poulsen AH, Stenager E, Johansen C, et al. (2012). Mobile phones and multiple sclerosis--a nationwide cohort study in Denmark. PLoS One, 7(4), e34453.

Poulsen AH, Friis S, Johansen C, et al. (2013). Mobile phone use and the risk of skin cancer: a nationwide cohort study in Denmark. Am J Epidemiol. 178(2), 190-7.

Prato FS, Desjardins-Holmes D, Keenliside LD, DeMoor JM, Robertson JA and Thomas AW (2013). Magnetoreception in laboratory mice: sensitivity to extremely low-frequency fields exceeds 33 nT at 30 Hz. J R Soc Interface, 10(81), 20121046.

Preece A W, Kaune W, Grainger P, Preece S, and J Golding (1997). Magnetic fields from domestic appliances in the UK. Physics in Medicine and Biology, 42(1), 67-76.

Prochnow N, Gebing T, Ladage K, Krause-Finkeldey, El Quardi A, Bitz A, Streckert J, Hansen V, Dermietzel R (2011). Electromagnetic field effect or simply stress? Effects of UMTS exposure on hippocampal longterm plasticity in the context of procedure related hormone release. PLOS One, 6, e19437.

Qi H, Wenfang C, Xia A, Qiang W, Ying L, Kun Z, Runguang S (2011). Effects of a moderate-intensity static magnetic field and adriamycin on K562 cells. Bioelectromagnetics, 32(3), 191-9.

Qian AR, Hu LF, Gao X, Zhang W, Di SM, Tian ZC, Yang PF, Yin DC, Weng YY, Shang P (2009). Large Gradient High Magnetic Field Affects the Association of MACF1 With Actin and Microtubule Cytoskeleton. Bioelectromagnetics, 30, 545-55.

Qin F, Zhang J, Cao H, Guo W, Chen L, Shen O, Sun J, Yi C, Li J, Wang J, Tong J (2014). Circadian alterations of reproductive functional markers in male rats exposed to 1800 MHz radiofrequency field. Chronobiol Int, 31(1), 123-33.

Rageh MM, El-Gebaly RH, El-Bialy NS (2012). Assessment of genotoxic and cytotoxic hazards in brain and bone marrow cells of newborn rats exposed to extremely low-frequency magnetic field. J Biomed Biotechnol, 2012, 716023.

Rago R, Salacone P, Caponecchia L, Sebastianelli A, Marcucci I, Calogero AE, Condorelli R, Vicari E, Morgia G, Favilla V, Cimino S, Arcoria AF, La Vignera S (2013). The semen quality of the mobile phone users. J Endocrinol Invest, 36(11), 970-4.

Rajkovic V, Matavulj M, Johansson O (2010). Combined exposure of peripubertal male rats to the endocrine-disrupting compound atrazine and power-frequency electromagnetic fields causes degranulation of cutaneous mast cells: a new toxic environmental hazard? Arch Environ Contam Toxicol, 59(2), 334-41.

Ramundo Orlando A, Gallerano GP (2009). Terahertz Radiation Effects and Biological Applications. Journal of Infrared, Millimeter, and Terahertz Waves, 30(12), 1308-18.

Rao VS, Titushkin IA, Moros EG, Pickard WF, Thatte HS, Cho MR (2008). Nonthermal effects of radiofrequency-field exposure on calcium dynamics in stem cell-derived neuronal cells: elucidation of calcium pathways. Radiat Res, 169(3), 319-29.

Rauš S, Selaković V, Radenović L, Prolić Z, Janać B (2012). Extremely low frequency magnetic field induced changes in motor behaviour of gerbils submitted to global cerebral ischemia. Behav Brain Res, 228(2), 241-6.

Rauš S, Selaković V, Manojlović-Stojanoski M, Radenović L, Prolić Z, Janać B (2013). Response of Hippocampal Neurons and Glial Cells to Alternating Magnetic Field in Gerbils Submitted to Global Cerebral Ischemia. Neurotox Res, 23(1), 79-91.

Rauš Balind S, Selaković V, Radenović L, Prolić Z, Janać B (2014). Extremely Low Frequency Magnetic Field (50 Hz, 0.5 mT) Reduces Oxidative Stress in the Brain of Gerbils Submitted to Global Cerebral Ischemia. PLoS One, 9(2), e88921.

Rauschenberg J, Nagel AM, Ladd SC, Theysohn JM, Ladd ME, Möller HE, Trampel R, Turner R, Pohmann R, Scheffler K, Brechmann A, Stadler J, Felder J, Shah NJ, Semmler W (2014). Multicenter study of subjective acceptance during magnetic resonance imaging at 7 and 9.4 T. Invest Radiol, 49(5), 249-59.

Redmayne M, Smith E, Abramson MJ (2013). The relationship between adolescents' well-being and their wireless phone use: a cross-sectional study. Environmental Health, 12(1), 90.

Regel SJ, Achermann P (2011). Cognitive performance measures in bioelectromagnetic research – critical evaluation and recommendations. Environmental Health, 10(1), 10.

Reid A, Glass DC, Bailey HD, Milne E, de Klerk NH, Downie P, Fritschi L, Aus-ALL Consortium (2011). Risk of childhood acute lymphoblastic leukaemia following parental occupational exposure to extremely low frequency electromagnetic fields. Br J Cancer, 105(9), 1409-13.

Reilly JP (1998). Applied bioelectricity. From electrical stimulation to electropathology. New York, NY, USA: Springer-Verlag.

Reilly JP, Diamant AM, Comeaux J (2009). Dosimetry considerations for electrical stun devices. Physics in Medicine and Biology, 54(5), 1319-35.

Repacholi MH, Basten A, Gebski V, Noonan D, Finnie J, Harris AW (1997). Lymphomas in E mu-Pim1 transgenic mice exposed to pulsed 900 MHZ electromagnetic fields. Radiat Res, 147(5), 631-40.

Repacholi MH, Lerchl A, Röösli M, Sienkiewicz Z, Auvinen A, Breckenkamp J, d'Inzeo G, Elliott P, Frei P, Heinrich S, Lagroye I, Lahkola A, McCormick DL, Thomas S, Vecchia P (2012). Systematic review of wireless phone use and brain cancer and other head tumors. Bioelectromagnetics, 33(3), 187-206.

Reyes-Guerrero G, Guzmán C, García DE, Camacho-Arroyo I, Vázquez-García M (2010). Extremely low-frequency electromagnetic fields differentially regulate estrogen receptor-alpha and -beta expression in the rat olfactory bulb. Neurosci Lett, 471(2), 109-13.

Rice TK, Schork NJ, Rao DC (2008). Methods for handling multiple testing. Adv Genet, 60, 293-308.

Riddervold IS, Kjaergaard SK, Pedersen GF, Andersen NT, Franek O, Pedersen AD, Sigsgaard T, Zachariae R, Molhave L, Andersen JB (2010). No effect of hand portable transmission signals on human cognitive function and symptoms. Bioelectromagnetics, 31, 380-90.

Rikk J, Finn KJ, Liziczai I, Radák Z, Bori Z, Ihász F (2013). Influence of pulsing electromagnetic field therapy on resting blood pressure in aging adults. Electromagn Biol Med, 32(2), 165-72.

Roberts DC, Marcelli V, Gillen JS, Carey JP, della Santina CC, Zee DS (2011). MRI magnetic field stimulates rotational sensors of the brain. Current Biology, 21, 1-6.

Robertson JA, Juen N, Theberge J, Weller J, Drost DJ, Prato FS, Thomas AW (2010). Evidence for a dose-dependent effect of pulsed magnetic fields on pain processing. Neuroscience Letters, 482, 160-2.

Robertson JA, Théberge J, Weller J, Drost DJ, Prato FS, Thomas AW (2010). Low-frequency pulsed electromagnetic field exposure can alter neuroprocessing in humans. J R Soc Interface, 7(44), 467–73.

Röösli M, Lörtscher M, Egger M, Pfluger D, Schreier N, Lörtscher E, Locher P, Spoerri A, Minder C (2007). Leukaemia, brain tumours and exposure to extremely low frequency

magnetic fields: cohort study of Swiss railway employees. Occup Environ Med, 64(8), 553-9.

Röösli M, Frei P, Mohler E, Hug K (2010). Systematic review on the health effects of exposure to radiofrequency electromagnetic fields from mobile phone base stations. Bulletin of the World Health Organization, 88(12), 887-896.

Röösli M, Jenni D, Kheifets L, Mezei G (2011). Extremely low frequency magnetic field measurements in buildings with transformer stations in Switzerland. Science of the Total Environment, 409(18), 3364-9.

Rossi S, Hallett M, Rossini PM, Pascual-Leone A, The Safety of TMS Consensus Group (2009). Safety, ethical considerations, and application guidelines for the use of transcranial magnetic stimulation in clinical practice and research. Clinical Neurophysiology, 120(12), 2008-39.

Roux D, Girard S, Paladian F, Bonnet P, Lallechere S,Gendraud M, Davies E, Vian A (2010). Human Keratinocytesin Culture Exhibit No Response When Exposed to Short Duration, Low Amplitude, High Frequency (900 MHz) Electromagnetic Fields in a Reverberation Chamber. Bioelectromagnetics, 32, 302-11.

Rowley JT, Joyner, Ken H (2012). Comparative international analysis of radiofrequency exposure surveys of mobile communication radio base stations. J Expos Sci Environ Epidemiol, 22(3), 304-15.

Rubin GJ, Das Munshi J, Wessely S (2005). Electromagnetic hypersensitivity: A systematic review of provocation studies. Psychosomatic Medicine, 67, 224-32.

Rubin GJ, Nieto-Hernandez R, Wessely S (2010). Idiopathic environmental intolerance attributed to electromagnetic fields (formerly ‘electromagnetic hypersensitivity’): An updated systematic review of provocation studies. Bioelectromagnetics, 31, 1-11.

Rubin GJ, Cleare AJ, Wessely S (2012). Letter to the editor: Electromagnetic hypersensitivity. International Journal of Neuroscience, 122, 401.

Sadick NS, Makino Y (2004). Selective Electro-Thermolysis in Aesthetic Medicine: A Review. Lasers in Surgery and Medicine, 34(2), 91–7.

Saito A, Takayama Y, Moriguchi H, Kotani K, Jimbo Y (2013). Developmental effects of low frequency magnetic fields on P19-derived neuronal cells. Conf Proc IEEE Eng Med Biol Soc, 2009, 5942-5.

Saito T, Nitta H, Kubo O, Yamamoto S, Yamaguchi N, Akiba S, Honda Y, Hagihara J, Isaka K, Ojima T, Nakamura Y, Mizoue T, Ito S, Eboshida A, Yamazaki S, Sokejima S, Kurokawa Y, Kabuto M (2010). Power-frequency magnetic fields and childhood brain tumors: a case-control study in Japan. *J Epidemiol, 20(1), 54-61.*

Sakurai T, Kiyokawa T, Kikuchi K, Miyakoshi J (2009). Intermediate frequency magnetic fields generated by an induction heating (IH) cooktop do not affect genotoxicities and expression of heat shock proteins. Int J Radiat Biol, 85(10), 883-90.

Sakurai T, Kiyokawa T, Narita E, Suzuki Y, Taki M, Miyakoshi J (2011). Analysis of gene expression in a human-derived glial cell line exposed to 245 GHz continuous radiofrequency electromagnetic fields. J Radiat Res, 52(2), 185-92.

Sakurai T, Narita E, Shinohara N, Miyakoshi J (2012). Intermediate frequency magnetic field at 23 kHz does not modify gene expression in human fetus-derived astroglia cells. Bioelectromagnetics, 33(8), 662-9.

Sakurai T, Narita E, Shinohara N, Miyakoshi J (2013). Alteration of gene expression by exposure to a magnetic field at 23 kHz is not detected in astroglia cells. J Radiat Res, 54, 1005–9.

Salunke BP, Umathe SN, Chavan JG (2013). Involvement of NMDA receptor in low-frequency magnetic field-induced anxiety in mice. Electromagn Biol Med, 33(4), 312-26.

Salunke BP, Umathe SN and Chavan JG (2014). Experimental evidence for involvement of nitric oxide in low frequency magnetic field induced obsessive compulsive disorder-like behavior. Pharmacol Biochem Behav, 122, 273-8.

Sambucci M, Laudisi F, Nasta F, Pinto R, Lodato R, Altavista P, Lovisolo GA, Marino C, Pioli C (2010). Prenatal exposure to non-ionizing radiation: effects of WiFi signals on pregnancy outcome, peripheral B-cell compartment and antibody production. Radiat Res, 174(6), 732-40.

Sambucci M, Laudisi F, Nasta F, Pinto R, Lodato R, Lopresto V, Altavista P, Marino C, Pioli C (2011). Early life exposure to 2.45GHz WiFi-like signals: effects on development and maturation of the immune system.Prog Biophys Mol Biol, 107(3), 393-8.

Sangun O, Dundar B, Darici H, Comlekci S, Doguc DK, Celik S (2014). The effects of long-term exposure to a 2450 MHz electromagnetic field on growth and pubertal development in female Wistar rats. Electromagn Biol Med, Early Online, 1-9, Jan 24.

Sannino A, Di Costanzo G, Brescia F, Sarti M, Zeni O, Juutilainen J, Scarfì MR (2009a). Human fibroblasts and 900 MHz radiofrequency radiation: evaluation of DNA damage after exposure and co-exposure to 3-chloro-4-(dichloromethyl)-5-hydroxy-2(5 h)-furanone (MX). Radiat Res, 171, 743–51.

Sannino A, Sarti M, Reddy SB, Prihoda TJ, Vijayalaxmi, Scarfi MR (2009b). Induction of adaptive response in human blood lymphocytes exposed to radiofrequency radiation. Radiat Res, 171(6), 735–42.

Sannino A, Zeni O, Sarti M, Romeo S, Reddy SB, Belisario A, Prihoda TJ, Vijayalaxmi, Scarfì MR (2011). Characterization of Radiofrequency-induced Adaptive Response in human peripheral blood lymphocytes: cell cycle effects. Int J Radiat Biol, 87(7), 1–8.

Sannino A, Zeni O, Romeo S, Massa R, Gialanella G, Grossi G, Manti L, Vijayalaxmi, Scarfì MR (2014). Adaptive response in human blood lymphocytes exposed to non-ionizing radiofrequency fields: resistance to ionizing radiation-induced damage. J Radiat Res. 55(2), 210-217

Sarvestani AS, Abdolmaleki P, Mowla SJ, Ghanati F, Heshmati E, Tavasoli Z, Jahromi AM (2010). Static magnetic fields aggravate the effects of ionizing radiation on cell cycle progression in bone marrow stem cells. Micron, 41(2), 101-4.

Sauter C, Dorn H, Bahr A, Hansen ML, Peter A, Bajbouj M, Danker-Hopfe H (2012). Effects of exposure to electromagnetic fields emitted by GSM 900 and WCDMA mobile phones on cognitive function in young male subjects. Bioelectromagnetics, 32, 179-90.

Scanlon WG, Evans NE, Burns JB (1999). FDTD analysis of close-coupled 418 MHz radiating devices for human biotelemetry. Physics in Medicine and Biology, 44(2), 335.

SCENIHR (Scientific Committee on Emerging and Newly Identified Health Risks) (2009) Health Effects of Exposure to EMF. European Commission.

SCENIHR (Scientific Committee on Emerging and Newly Identified Health Risks) (2009). Research needs and methodology to address the remaining knowledge gaps on the potential health effects of EMF. European Commission.

SCENIHR (Scientific Committee on Emerging and Newly Identified Health Risks) (2012). Memorandum on the use of the scientific literature for human health risk assessment purposes – weighing of evidence and expression of uncertainty. European Commission.

Schaap K, Christopher-De Vries Y, Mason CM, De Vocht F, Portenten L, Kromhout H (2014). Occupational exposure of health care and research staff to static magnetic stray fields from 1.5 - 7 Tesla MRI scanners is associated with reporting of transient symptoms. Occup Environ Med, 71(6), 423-9.

Schindler JW, Van Buren D, Foudi A, Krejci O, Qin J, Orkin SH, Hock H (2009). TEL-AML1 corrupts hematopoietic stem cells to persist in the bone marrow and initiate leukemia. Cell Stem Cell, 5, 43-53.

Schlamann M, Voigt MA, Maderwald S, Bitz AK, Kraff O, Ladd SC, Ladd ME, Forsting M, Wilhelm HJ (2010a). Exposure to high-field MRI does not affect cognitive function. Magn Reson Imaging, 31(5), 1061-6.

Schlamann M, Yoon MS, Maderwald S, Pietrzyk T, Bitz AK, Gerwig M, Forsting M, Ladd SC, Ladd ME, Kastrup O (2010b). Short term effects of magnetic resonance imaging on excitability of the motor cortex at 1.5T and 7T. Acad Radiol, 17(3), 277-81.

Schmid G, Lager D, Preiner P, Überbacher R, Cecil S (2007). Exposure caused by wireless technologies used for short-range indoor communication in homes and offices. Radiat Prot Dosimetry, 124 (1), 58-62.

Schmid G, Überbacher R, Samaras T, Tschabitscher M, Mazal PR (2007). The dielectric properties of human pineal gland tissue and RF absorption due to wireless communication devices in the frequency range 400–1850 MHz. Physics in Medicine and Biology, 52(17), 5457.

Schmid G, Bolz T, Überbacher R, Escorihuela-Navarro A, Bahr A, Dorn H, Sauter C, Eggert T, Danker-Hopfe H (2012). Design and dosimetric analysis of a 385 MHz TETRA head exposure system for use in human provocation studies Bioelectromagnetics, 33(7), 594-603.

Schmid M, Loughran S, Regel SJ, Murbach M, Bratic Grunauer A, Rusterholz T, Bersagliere A, Kuster N, Achermann P (2012a). Sleep EEG alterations: effects of different pulse-modulated radio-frequency electromagnetic fields. J Sleep Res, 21, 50-8.

Schmid M, Murbach M, Lustenberger C, Maire M, Kuster N, Achermann P, Loughran S (2012b). Sleep EEG alterations: effects of pulsed magnetic fields versus pulse-modulated radio-frequency electromagnetic fields. J Sleep Res, 21(6), 620-9.

Schrader T, Munter K, Kleine-Ostmann T, Schmid E (2008). Spindle Disturbances in Human-Hamster Hybrid (AL) Cells Induced by Mobile Communication Frequency Range Signals. Bioelectromagnetics, 29, 626-39.

Schrader T, Kleine-Ostmann T,Munter K, Jastrow C, Schmid E (2011). Spindle Disturbances in Human Hamster Hybrid (AL) Cells Induced by the Electrical Component of the Mobile Communication Frequency Range Signal. Bioelectromagnetics, 32, 291-301.

Schubert M, Bornkessel C, Wuschek M and Schimdt P (2007). Exposure of the general public to digital broadcast transmitters compared to analogue ones. Radiat Prot Dosim, 124(1), 53–7.

Schüz J, Mann S (2000). A discussion of potential exposure metrics for use in epidemiological studies on human exposure to radiowaves from mobile phone base stations. Journal of Exposure Analysis and Environmental Epidemiology, 10(6 Pt 1), 600-5.

Schüz J, Waldemar G, Olsen JH, Johansen C (2009). Risks for central nervous system diseases among mobile phone subscribers: a Danish retrospective cohort study.PLoS One, 4(2), e4389.

Schüz J, Elliott P, Auvinen A, Kromhout H, Poulsen AH, Johansen C, Olsen JH, Hillert L, Feychting M, Fremling K, Toledano M, Heinävaara S, Slottje P, Vermeulen R, Ahlbom A (2011). An international prospective cohort study of mobile phone users and health (Cosmos): design considerations and enrolment. Cancer Epidemiol, 35(1), 37-43.

Schüz J, Grell K, Kinsey S, Linet MS, Link MP, Mezei G, Pollock BH, Roman E, Zhang Y, McBride ML, Johansen C, Spix C, Hagihara J, Saito AM, Simpson J, Robison LL, Dockerty JD, Feychting M, Kheifets L, Frederiksen K (2012). Extremely low-frequency magnetic fields and survival from childhood acute lymphoblastic leukemia: an international follow-up study. Blood Cancer J, 2, e98.

Schwarz C, Kratochvil E, Pilger A, Kuster N, Adlkofer F, Rüdiger HW (2008). Radiofrequency electromagnetic fields (UMTS, 1,950 MHz) induce genotoxic effects in

vitro in human fibroblasts but not in lymphocytes. Int Arch Occup Environ Health, 81(6), 755-67.

Sefidbakht Y, Hosseinkhani S, Mortazavi M, Tavakkolnia I, Khellat MR, Shakiba-Herfeh M, Saviz M, Faraji-Dana R, Saboury AA, Sheibani N, Moosavi-Movahedi AA (2013). Effects of 940 Hz EMF on luciferase solution: structure, function, and dielectric studies. Bioelectromagnetics, 34(6), 489-98.

Seidman SJ, Brockman R, Lewis BM, Guag J, Shein MJ, Clement WJ, et al (2010). In vitro tests reveal sample radiofrequency identification readers inducing clinically significant electromagnetic interference to implantable pacemakers and implantable cardioverter-defibrillators. Heart Rhythm, 7(1), 99-107.

Sekijima M, Takeda H, Yasunaga K, Sakuma N, Hirose H, Nojima T, Miyakoshi J (2010). 2-GHz band CW and W-CDMA modulated radiofrequency fields have no significant effect on cell proliferation and gene expression profile in human cells. J Radiat Res, 51(3), 277-84.

Selaković V, Rauš Balind S, Radenović L, Prolić Z and Janać B (2013). Age-dependent effects of ELF-MF on oxidative stress in the brain of Mongolian gerbils. Cell Biochem Biophys, 66(3), 513-21.

Sermage-Faure C, Demoury C, Rudant J, Goujon-Bellec S, Guyot-Goubin A, Deschamps F, Hemon D, Clavel J (2013). Childhood leukaemia close to high-voltage power lines--the Geocap study, 2002-2007. Br J Cancer 2013, 108(9), 1899-906.

Shafiei SA, Firoozabadi SM, Rasoulzadeh Tabatabaie K, Ghabaee M (2012). Study of the frequency parameters of EEG influenced by zone-dependent local ELF-MF exposure on the human head. Electromagn Bio Med, 31, 112-21.

Shafiei SA, Firoozabadi SM, Tabatabaie KR, Ghabaee M (2014). Investigation of EEG changes during exposure to extremely low-frequencymagnetic field to conduct brain signals. Neurol Sci, 35(11), 1715-21.

Shahbazi-Gahrouei D, Karbalae M, Moradi HA, Baradaran-Ghahfarokhi M (2014). Health effects of living near mobile phone base transceiver station (BTS) antennae: A report from Isfahan, Iran. Electromagnetic Biology and Medicine, 33(2), 206-10.

Shahin S, Mishra V, Singh SP and Chaturvedi CM (2014). 2.45-GHz microwave irradiation adversely affects reproductive function in male mouse, Mus musculus by inducing oxidative and nitrosative stress. Free Radical Res, 48(5), 511-25.

Sharma A, Sisodia R, Bhatnagar D, Saxena VK (2014). Spatial memory and learning performance and its relationship to protein synthesis of Swiss albino mice exposed to 10 GHz microwaves. Int J Radiat Biol, 90(1), 29-35.

Shiba K, Koshiji K, Tatsumi E, Taenaka Y, Takano H (2002). Analysis of specific absorption rate in biological tissue surrounding transcutaneous transformer for an artificial heart. Journal of Artificial Organs, 5(2), 91-6.

Shiba K, Nagato T, Tsuji T, Koshiji K (2008). Energy Transmission Transformer for a Wireless Capsule Endoscope: Analysis of Specific Absorption Rate and Current Density in Biological Tissue. IEEE Transactions on Biomedical Engineering, 55(7), 1864-71.

Shields N, O'Hare N, Gormley J (2004). An evaluation of safety guidelines to restrict exposure to stray radiofrequency radiation from short-wave diathermy units. Physics in Medicine and Biology, 49(13), 2999.

Shin EJ, Nguyen XK, Nguyen TT, Pham DT, Kim HC (2011). Exposure to extremely low frequency magnetic fields induces fos-related antigen-immunoreactivity via activation of dopaminergic d1 receptor. Exp Neurobiol, 20(3), 130-6.

Shu X, Ahlbom A, Feychting M (2012). Incidence trends of malignant parotid gland tumors in Swedish and nordic adults 1970 to 2009. Epidemiology, 23(5), 766-7.

Shumyatsky P, Alfano R R (2011). Terahertz sources. Journal of Biomedical Optics, 16(3), 033001.

Simi S, Ballardina M, Casella M, De Marchi D, Hartwig V, Giovannetti G, Vanello N, Gabbriellini S, Landini L, Lombardi M (2008). Is the genotoxic effect of magnetic resonance negligible? Low persistence of micronucleus frequency in lymphocytes of individuals after cardiac scan. Mutat Res, 645, 39–43.

Simkó M, Mattsson MO (2004). Extremely low frequency electromagnetic fields as effectors of cellular responses in vitro: possible immune cell activation. J Cell Biochem, 93(1), 83-92.

Simon D, Daubos A, Pain C, Fitoussi R, Vie´K, Taieb A, de Benetti L, Cario-Andre'M (2013). Exposure to acute electromagnetic radiation of mobile phone exposure range alters transiently skin homeostasis of a model of pigmented reconstructed epidermis. Int J Cosmetic Sci, 35, 27–34.

Singh V, Qusba A, Roy A, Castro RA, McClure K, Dai R, Lazzi G (2009). Specific absorption rate and current densities in the human eye and head induced by the telemetry link of an epiretinal prosthesis. Antennas and Propagation, IEEE Transactions on, 57(10), 3110-8.

Söderqvist F, Carlberg M, Hardell L (2009a). Use of wireless telephones and serum S100B levels: a descriptive cross-sectional study among healthy Swedish adults aged 18-65 years. Sci Total Environ 407, 798-805.

Söderqvist F, Carlberg M, Hardell L (2009b). Mobile and cordless telephones, serum transthyretin and the blood-cerebrospinal fluid barrier: a cross-sectional study. Environ Health 8, 19.

Söderqvist F, Carlberg M, Hansson Mild K, Hardell L (2009c). Exposure to an 890-MHz mobile phone-like signal and serum levels of S100B and transthyretin in volunteers. Toxicol Lett 189, 63-6.

Söderqvist F, Carlberg M, Hardell L (2012a). Use of wireless phones and the risk of salivary gland tumours: a case-control study. Eur J Cancer Prev, 21(6), 576-9.

Söderqvist F, Carlberg M, Zetterberg H, Hardell L (2012b). Use of wireless phones and serum b-trace protein in randomly recruited persons aged 18–65 years: a cross-sectional study. Electromagnetic Biology and Medicine, 31(4): 416–24.

Sommer AM, Grote K, Reinhardt T, Streckert J, Hansen V, Lerchl A (2009). Effects of radiofrequency electromagnetic fields (UMTS) on reproduction and development of mice: a multi-generation study. Radiat Res, 171(1), 89-95.

Souques M, Magne I, Lambrozo J (2011). Implantable cardioverter defibrillator and 50-Hz electric and magnetic fields exposure in the workplace. Int Arch Occup Environ Health, 84, 1-6.

Spichtig S, Scholkmann F, Chin L, Lehmann H, Wolf M (2012). Assessment of intermittent UMTS electromagnetic field effects on blood circulation in the huan auditory region using a near-infrared system. Bioelectromagnetics, 33, 40-54.

SSM:s Scientific Council on Electromagnetic Fields (2013): 2013:19 Eighth report from SSM:s Scientific Council on Electromagnetic Fields.

Stang A, Anastassiou G, Ahrens W, et al (2001). The possible role of radiofrequency radiation in the development of uveal melanoma. Epidemiology, 12(1), 7-12.

Stang A, Schmidt-Pokrzywniak A, Lash TL, et al (2009). Mobile phone use and risk of uveal melanoma: results of the risk factors for uveal melanoma case-control study. J Natl Cancer Inst, 101(2), 120-3.

Strasák L, Bártová E, Krejci J, Fojt L, Vetterl V (2009). Effects of ELF-EMF on brain proteins in mice. Electromagn Biol Med, 28(1), 96-104.

Sudan M, Kheifets L, Arah O, Olsen J, Zeltzer L (2012). Prenatal and postnatal cell phone exposures and headaches in children. Open Pediatric Medicine Journal, 6, 46-52.

Sudan M, Kheifets L, Arah OA, Olsen J (2013). Cell phone exposures and hearing loss in children in the Danish National Birth Cohort. Paediatr Perinat Epidemiol, 27(3), 247-57.

Suhhova A1, Bachmann M, Karai D, Lass J, Hinrikus H (2013). Effect of microwave radiation on human EEG at two different levels of exposure. Bioelectromagnetics, 34(4), 264-74.

Sullivan K, Balin AK, Allen RG (2011). Effects of static magnetic fields on the growth of various types of human cells. Bioelectromagnetics, 32(2), 140-7.

Sun W, Shen X, Lu D, Fu Y, Lu D, Chiang H (2012). A 18-GHz radiofrequency radiation induces EGF receptor clustering and phosphorylation in cultured human amniotic (FL) cells. Int J Radiat Biol, 88(3), 239-44.

Swaen GM, Carmichael N, Doe J (2011). Strengthening the reliability and credibility of observational epidemiology studies by creating an Observational Studies Register. J Clin Epidemiol, 64, 481-86.

Swanson ES (2011). Modeling DNA response to THz radiation, Phys Rev E Stat Nonlin Soft Matter Phys, 83(4 Pt 1), 040901.

Szabo J, Gabor Janossy G, Thuroczy G (2007). Survey of Residential 50 Hz EMF Exposure from Transformer Stations. Bioelectromagnetics, 28(1), 48-52.

Szerencsi Á, Kubinyi G, Váliczkó É, Juhász P, Rudas G, Mester Á, Jánossy G, Bakos J, Thuróczy G. (2013) DNA integrity of human leukocytes after magnetic resonance imaging. Int J Radiat Biol, 89(10), 870-6.

Szemerszky R, Zelena D, Barna I, Bárdos G (2010). Stress-related endocrinological and psychopathological effects of short- and long-term 50Hz electromagnetic field exposure in rats Brain Res Bull, 81(1), 92-9.

Szyjkowska A, Gadzicka E, Szymczak W, Bortkiewicz A (2014). The risk of subjective symptoms in mobile phone users in Poland – an epidemiological study. International J Occup Med Environ Health, 27(2), 293-303.

Takahashi S, Imai N, Nabae K, Wake K, Kawai H, Wang J, Watanabe S, Kawabe M, Fujiwara O, Ogawa K, Tamano S, Shirai T (2010). Lack of adverse effects of whole-body exposure to a mobile telecommunication electromagnetic field on the rat fetus. Radiat Res, 173(3), 362-72.

Tanenbaum A. (2002). Computer Networks, 101. Prentice Hall.

Tas M, Dasdag S, Akdag MZ, Cirit U, Yegin K, Seker U, Ozmen MF and Eren LB (2013). Long-term effects of 900 MHz radiofrequency radiation emitted from mobile phone on testicular tissue and epididymal semen quality. Electromagn Biol Med, 33(3), 216-22.

Tasset I, Medina FJ, Jimena I, Agüera E, Gascón F, Feijóo M, Sánchez-López F, Luque E, Peña J, Drucker-Colín R, Túnez I (2012). Neuroprotective effects of extremely low-frequency electromagnetic fields on a Huntington's disease rat model: effects on neurotrophic factors and neuronal density. Neuroscience, 209, 54-63.

Tasset I, Pérez-Herrera A, Medina FJ, Arias-Carrión O, Drucker-Colín R, Túnez I (2013). Extremely low-frequency electromagnetic fields activate the antioxidant pathway Nrf2 in a Huntington's disease-like rat model. Brain Stimul, 6, 84-6.

Taylor ZD, Singh RS, Culjat MO, Suen JY, Grundfest WS, Lee H, Brown E R (2008). Reflective terahertz imaging of porcine skin burns. Optics Letters, 33(11), 1258-1260.

Tell RA and Mantiply ED (1980). Population exposure to VHF and UHF broadcast radiation in the United States. Proc IEEE, 68(1), 6–12.

Tell RA, Sias GG, Vazquez A, Sahl J, Turman JP, Kavet RI, Mezei G (2012). Radiofrequency fields associated with the Itron smart meter. Radiation protection dosimetry, 151(1), 17-29.

Tell RA, Sias G, Smith J, Sahl J, Kavet R (2013). ELF magnetic fields in electric and gasoline-powered vehicles. Bioelectromagnetics, 34(2), 156-61.

Tell RA, Kavet R (2014). A survey of the urban radiofrequency (RF) environment. Radiat Prot Dosimetry, 162(4), 499-507.

Terro F, Magnaudeix A, Crochetet M, et al (2012). GSM-900MHz at low dose temperature-dependently downregulates alpha-synuclein in cultured cerebral cells independently of chaperone-mediated-autophagy. Toxicology, 292, 136-44.

Theysohn J, Maderwald S, Kraff O, Moenninghoff C, Ladd ME, Ladd SC (2008). Subjective acceptance of 7 Tesla MRI for human imaging. Magnetic Resonance Material Physics, 21, 63-72.

Theysohn JM, Kraff O, Eilers K, Andrade D, Gerwig M, Timmann D, Schmitt F, Ladd ME, Ladd SC, Bitz AK (2014). Vestibular effects of a 7 Tesla MRI examination compared to 1.5T and 0 T in healthy volunteers. PLOS One, 9(3), e92104.

Thomas S, Heinrich S, von Kries R, Radon K (2010). Exposure to radio-frequency electromagnetic fields and behavioural problems in Bavarian children and adolescents. European Journal of Epidemiology, 25, 135-141.

Thomee S, Härenstam A, Hagberg M (2011). Mobile phone use and stress, sleep disturbances, and symptoms of depression among young adults – a prospective cohort study. BMC Public Health, 11, 66.

Thormann M, Amthauer H, Adolf D, Wollrab A, Ricke J, Speck O (2013). Efficacy of diphenhydramine in the prevention of vertigo and nausea at 7 T MRI. Eur J Radiol, 82(5), 768-72.

Tiikkaja M, Alanko T, Lindholm H, Hietanen M, Hartikainen J, Toivonen L (2012a). Experimental study on malfunction of pacemakers due to exposure to different external magnetic fields. J Interv Card Electrophysiol, 34(1), 19-27.

Tiikkaja M, Alanko T, Lindholm H, Hietanen M, Toivonen L, Hartikainen J (2012b). Interference of low frequency magnetic fields with implantable cardioverter-defibrillators. Scand Cardiovasc J, 46(5), 308-14.

Tiikkaja M, Aro AL, Alanko T, Lindholm H, Sistonen H, Hartikainen JE, Toivonen L, Juutilainen J, Hietanen M (2013). Electromagnetic interference with cardiac pacemakers and implantable cardioverter-defibrillators from low-frequency electromagnetic fields in vivo. Europace, 15(3), 388-94.

Tillmann T, Ernst H, Streckert J, Zhou Y, Taugner F, Hansen V, Dasenbrock C (2010). Indication of cocarcinogenic potential of chronic UMTS-modulated radiofrequency exposure in an ethylnitrosourea mouse model. Int J Radiat Biol, 86 (7), 529-541.

Titova LV, Ayesheshim AK, Golubov A, Fogen D, Rodriguez-Juarez R, Hegmann FA, Kovalchuk O. (2013a). Intense THz pulses cause H2AX phosphorylation and activate DNA damage response in human skin tissue. Biomed Opt Express, 4(4), 559-68.

Tiwari R, Lakshmi NK, Surender V, Rajesh AD, Bhargava SC, Ahuja YR (2008). Combinative exposure effect of radiofrequency signals from CDMA mobile phones and aphidicolin on DNA integrity. Electromagn Biol Med, 27, 418–25.

Tombini M, Pellegrino G, Pasqualetti P, Assenza G, Benvenga A, Fabrizio E, Rossini PM (2013). Mobile phone emissions modulate brain excitability in patients with focal epilepsy. Brain Stimul, 6(3), 448-54.

Trillo MA, Cid MA, Martinez MA, Page JE, Esteban J, Ubeda A (2011). Cytostatic response of NB69 cells to weak pulse-modulated 2.2 GHz radar-like signals. Bioelectromagnetics, 32, 340-50.

Trillo MA, Martínez MA, Cid MA, Leal J, Úbeda A (2012). Influence of a 50 Hz magnetic field and of all-trans-retinol on the proliferation of human cancer cell lines. Int J Oncol, 40(5), 1405-13.

Trillo MÁ, Martínez MA, Cid MA, Úbeda A (2013). Retinoic acid inhibits the cytoproliferative response to weak 50-Hz magnetic fields in neuroblastoma cells. Oncol Rep, 29(3), 885-94.

Trošić I, Mataušić-Pišl M, Pavičić I and Marjanović AM (2013). Histological and cytological examination of rat reproductive tissue after short-time intermittent radiofrequency exposure. Arh Hig Rada Toksikol, 64(4), 513-9.

Trulsson J, Anger G, Estenberg U (2007). Assessment of magnetic fields surrounding electronic article surveillance systems in Sweden. Bioelectromagnetics, 28(8), 664-6.

Trunk A, Stefanics G, Zentai N, Kovács-Bálint Z, Thuróczy G, Hernádi I (2012). No effects of a single 3G UMTS mobile phone exposure on spontaneous EEG activity, ERP correlates, and automatic deviance detection. Bioelectromagnetics, 34, 31-42.

Trunk A, Stefanics G, Zentai N, Bacskay I, Felinger A, Thuróczy G, Hernádi I (2014). Lack of interaction between concurrent caffeine and mobile phone exposure on visual target detection: An ERP study. Pharmacol Biochem Behav, 124, 412-20.

Turner MC, Benke G, Bowman JD, Figuerola J, Fleming S, Hours M, Kincl L, Krewski D, McLean D, Parent ME, Richardson L, Sadetzki S, Schlaefer K, Schlehofer B, Schüz J, Siemiatycki J, van Tongeren M, Cardis E (2014). Occupational exposure to extremely low-frequency magnetic fields and brain tumor risks in the INTEROCC study. Cancer Epidemiol Biomarkers Prev, 23(9), 1863-72.

Urbinello D, Röösli M (2013). Impact of one's own mobile phone in stand-by mode on personal radiofrequency electromagnetic field exposure. J Expos Sci Environ Epidemiol, 23(5), 545-8.

Valberg PA (1995). Designing EMF experiments: What is required to characterize "exposure"? Bioelectromagnetics, 16(6), 396–401.

Valentini E, Ferrara M, Presaghi F, de Gennaro L, Curcio G (2010). Systematic review and meta-analysis of psychomotor effects of mobile phone electromagnetic fields. Occup Environ Med, 67, 708-16.

Valentini E, Ferrara M, Presaghi F, de Gennaro L, Curcio G (2011). Republished review: Systematic review and meta-analysis of psychomotor effects of mobile phone electromagnetic fields. Postgrad Med, 87, 643-51.

Van der Weyden L, Giotopoulos G, Rust AG, Matheson LS, van Delft FW, Kong J, Corcoran AE, Greaves MF, Mullighan CG, Huntly BJ, Adams DJ (2011). Modeling the evolution of ETV6-RUNX1-induced B-cell precursor acute lymphoblastic leukemia in mice. Blood, 118, 1041-51.

Van Nierop LE, Slottje P, van Zandvoort MJ, de Vocht F, Kromhout H (2012). Effects of magnetic stray fields from a 7 Tesla MRI scanner on neurocognition: a double-blind randomised crossover study. Occup Environ Med, 69(10), 759-66.

Van Nierop LE, Slottje P, Kingma H, Kromhout H (2013). MRI-related static magnetic stray fields and postural body sway: A double-blind randomized crossover study. Magn Reson Med, 232-40.

Vecchio F, Babiloni C, Ferreri F, Buffo P, Cibelli G, Curcio G, van Dijkman S, Melgari JM, Giambattistelli F, Rossini PM (2010). Mobile phone emission modulates inter-hemisperic functional coupling of EEG alpha rhythms in elderly compared to young subjects. Clin Neurophys, 121, 163-71.

Vecchio F, Tombini M, Buffo P, Assenza G, Pellegrino G, Benvenga A, Babiloni C, Rossini PM (2012a). Monile phone emission increases inter-hemispheric functional coupling of electroencephalographic alpha rhythms in epileptic patients. Int J Psychophysiol, 84, 164-71.

Vecchio F, Buffo P, Sergio S, Iacoviello D, Rossini PM, Babiloni C (2012b). Mobile phone emission modulates event-related desynchronozation of alpha rhythms and cognitive-motor performance in healthy humans. Clin Neurophys, 123, 121-28.

Vecsei Z, Csathó Á, Thuróczy G, Hernádi I (2013). Effect of a single 30 min UMTS mobile phone-like exposure on the thermal pain threshold of young healthy volunteers. Bioelectromagnetics, 34(7), 530-41.

Vedholm K, Hamnerius Y (1997). Personal exposure from low frequency electromagnetic fields in automobiles. Abstract Book, Second World Congress for Electricity and Magnetism in Biology and Medicine, June 8-13, 116-7.

Vergara X, Kheifets L, Greenland S, Oksuzyan S, Cho YS, Mezei G (2013). Occupational exposure to extremely low-frequency magnetic fields and neurodegenerative disease: a meta-analysis. J Occup Environ Med, 55(2), 135-46.

Verschaeve L, Juutilainen J, Lagroye I, Miyakoshi J, Saunders R, de Seze R, Tenforde T, van Rongen E, Veyret B, Xu Z (2010). In vitro and in vivo genotoxicity of radiofrequency fields. Mutat Res, 705, 252-68.

Vijayalaxmi, Prihoda TJ (2012). Genetic damage in human cells exposed to non-ionizing radiofrequency fields: a meta-analysis of the data from 88 publications (1990-2011). Mutat Res, 749(1-2), 1-16.

Vijayalaxmi, Reddy AB, McKenzie RJ, McIntosh RL, Prihoda TJ, Wood AW *(2013). Incidence of micronuclei in human peripheral blood lymphocytes exposed to modulated and unmodulated 2450 MHz radiofrequency fields. Bioelectromagnetics, 34(7), 542-8.*

Vijver MG, Bolte JF, Evans TR, Tamis WL, Peijnenburg WJ, Musters CJ and de Snoo GR (2014). Investigating short-term exposure to electromagnetic fields on reproductive capacity of invertebrates in the field situation. Electromagn Biol Med, 33(1), 21-8.

Villarini M, Ambrosini MV, Moretti M, Dominici L, Taha E, Piobbico D, Gambelunghe C and Mariucci G (2013). Brain hsp70 expression and DNA damage in mice exposed to extremely low frequency magnetic fields: a dose-response study. Int J Radiat Biol, 89(7), 562-70.

Volkow ND, Tomasi D, Wang GJ, Vaska P, Fowler JS, Telang F, Alexoff D, Logan J, Wong C (2011). Effects of Cell Phone Radiofrequency Signal Exposure on Brain Glucose Metabolism. JAMA, 305(8), 808-13.

Vrijheid M, S Mann, P Vecchia, J Wiart, M Taki, L Ardoino, B K Armstrong, A Auvinen, D Bédard, G Berg-Beckhoff, J Brown, A Chetrit, H Collatz-Christensen, E Combalot, A Cook, I Deltour, M Feychting, G G Giles, S J Hepworth, M Hours, I Iavarone, C Johansen, D Krewski, P Kurttio, S Lagorio, S Lönn, M McBride, L Montestrucq, R C Parslow, S Sadetzki, J Schüz, T Tynes, A Woodward, E Cardis (2009). Determinants of mobile phone output power in a multinational study: implications for exposure assessment. Occup Environ Med, 66(10), 664-71.

Vrijheid M, Martinez D, Forns J, et al (2010). Prenatal exposure to cell phone use and neurodevelopment at 14 months. Epidemiology, 21(2), 259-62.

Wacholder S, Chanock S, Garcia-Closas M, El Ghormli L, Rothman N (2004). Assessing the probability that a positive report is false: an approach for molecular epidemiology studies. J Natl Cancer Inst, 1996, 434-42.

Wainwright PR (2007). Computational modelling of temperature rises in the eye in the near field of radiofrequency sources at 380, 900 and 1800 MHz. Physics in Medicine and Biology, 52(12), 3335-50.

Waldmann P, Bohnenberger S, Greinert R, Hermann-Then B, Heselich A, Klug SJ, Koenig J, Kuhr K, Kuster N, Merker M, Murbach M, Pollet D, Schadenboeck W, Scheidemann-Wesp U, Schwab B, Volkmer B, Weyer V, Blettner M (2013). Influence of GSM signals on human peripheral lymphocytes: study of genotoxicity. Radiat Res, 179(2), 243-53.

Wallace D, Eltiti S, Ridgewell A, Garner K, Russo R, Sepulveda F, Walker S, Quinlan T, Dudley S, Maung S, Deeble R, Fox E (2010). Do TETRA (Airwave) base station signals have a short-term impact on health and well-being? A randomized double-blind provocation study. Environmental Health Perspectives, 118, 735-41.

Wallace D, Eltiti S, Ridgewell A, Garner K, Russo R, Sepulveda F, Walker S, Quinlan T, Dudley S, Maung S, Deeble R, Fox E (2012). Cognitive and physiological responses in humans exposed to a TETRA base station signal in relation to perceived electromagnetic hypersensitivity. Bioelectromagnetics, 33(1) 23-39.

Wang H, Peng R, Zhou H, Wang S, Gao Y, Wang L, Yong Z, Zuo H, Zhao L, Dong J, Xu X, Su Z (2013). Impairment of long-term potentiation induction is essential for the disruption of spatial memory after microwave exposure. Int J Radiat Biol, 89(12), 1100-7.

Wang Q, Cao Z, Qu Y, Peng X, Guo S, Chen L (2013). Residential exposure to 50 Hz magnetic fields and the association with miscarriage risk: a 2-year prospective cohort study. PLoS One, 8, e82113.

Wang W, Bottauscio O, Chiampi M, Giordano D, Zilberti L (2012). A procedure to estimate the electric field induced in human body exposed to unknown magnetic sources. Radiat Prot Dosimetry, 154, 157-63.

Wang X, Liu Y, Lei Y, Zhou D, Fu Y, Che Y, Xu R, Yu H, Hu X, Ma Y (2008). Extremely low-frequency electromagnetic field exposure during chronic morphine treatment strengthens downregulation of dopamine D2 receptors in rat dorsal hippocampus after morphine withdrawal. Neurosci Lett, 433(3), 178-82.

Wang X, Zhao K, Wang D, Adams W, Fu Y, Sun H, Liu X, Yu H and Ma Y (2013). Effects of exposure to a 50 Hz sinusoidal magnetic field during the early adolescent period on spatial memory in mice. Bioelectromagnetics, 34(4), 275-84.

Wang Z, Sarje A, Che PL, Yarema KJ (2009). Moderate strength (0.23-0.28 T) static magnetic fields (SMF) modulate signaling and differentiation in human embryonic cells. BMC Genomics, 4, 356.

Wang Z, Che PL, Du J, Ha B, Yarema KJ (2010). Static magnetic field exposure reproduces cellular effects of the Parkinson's disease drug candidate ZM241385. PLoS One, 5(11), e13883.

Ward BK, Roberts DC, Della Santina CC, Carey JP, Zee DS (2014). Magnetic vestibular stimulation in subjects with unilateral labyrinthine disorders. Front Neurol, 5, 28.

Watilliaux A, Edeline JM, Lévêque P, Jay TM, Mallat M (2011). Effect of exposure to 1,800 MHz electromagnetic fields on heat shock proteins and glial cells in the brain of developing rats. Neurotox Res, 20(2), 109-19.

Weinberg IN, Stepanov PY, Fricke ST, Probst R, Urdaneta M, Warnow D, Sanders H,Glidden SC, McMillan A, Starewicz PM, Reilly JP (2012). Increasing the oscillation frequency of strong magnetic fields above 101 kHz significantly raises peripheral nerve excitation thresholds. Med Phys, 39(5), 2578-83.

Wiholm C, Lowden A, Kuster N, Hillert L, Arnetz BB, Akerstedt T, Moffat SC (2009). Mobile phone exposure and spatial memory. Bioelectromagnetics, 30, 59-65.

Wilén J, Sandström M, Hansson Mild K (2003). Subjective symptoms among mobile phone users--a consequence of absorption of radiofrequency fields? Bioelectromagnetics, 24(3), 152-9.

Wilén J, Hörnsten R, Sandström M, Bjerle P, Wiklund U, Stensson O, Lyskov E, and Hansson Mild K (2004). Electromagnetic Field Exposure and Health Among RF Plastic Sealer Operators. Bioelectromagnetics, 25(1), 5-15.

Wilén J, Hauksson J, Hansson Mild K (2010). Modification of pulse sequences reduces occupational exposure from MRI switched gradient fields: Preliminary results. Bioelectromagnetics, 31(1), 85-7.

Wilén J, De Vocht F (2011). Health complaints among nurses working near MRI scanners - A descriptive pilot study. Eur J Radiol, 80(2), 510-3.

Williams R, Schofield A, Holder G, Downes J, Edgar D, Harrison P, Siggel-King M, Surman M, Dunning D, Hill S, Holder D, Jackson F, Jones J, McKenzie J, Saveliev Y, Thomsen N, Williams P, Weightman P (2013). The influence of high intensity terahertz radiation on mammalian cell adhesion, proliferation and differentiation. Phys Med Biol, 58(2), 373-91.

Wilmink G, , Roth CC, Ibey BL, Payne JA, Gundt JE, Peralta X, Mixon DG, Roach WP (2011a). In vitro investigation of the biological effects associated with human dermal fibroblasts exposed to 2.52 THz radiation. Lasers Surg Med, 43(2), 152-63.

Wilmink G and Grundt JE (2011b). Current State of Research on Biological Effects of Terahertz Radiation. J Infrared Milli Terahz Waves, 32, 1074–122.

Woodward RM, Wallace VP, Arnone DD, Linfield EH, Pepper M (2003). Terahertz Pulsed Imaging of Skin Cancer in the Time and Frequency Domain. Journal of Biological Physics, 29(2-3), 257-9.

Woodward RM, Wallace PV, Pye RJ, Cole BE, Arnone DD, lin EH, Pepper M (2003). Terahertz pulse imaging of ex vivo basal cell carcinoma. J Invest Dermatol, 120(1), 72–8.

World Health Organization. About Trial Registration. http://www.who.int/ictrp/trial_reg/en/index2.html (Accessed 26 November 2012)

World Medical Association (2008). Declaration of Helsinki. http://www.wma.net/en/30publications/10policies/b3/ (Accessed 26 November 2012)

Wünsch-Filho V, Pelissari DM, Barbieri FE, Sant'Anna L, de Oliveira CT, de Mata JF, Tone LG, Lee ML, de Andréa ML, Bruniera P, Epelman S, Filho VO, Kheifets L. (2011) Exposure to magnetic fields and childhood acute lymphocytic leukemia in São Paulo, Brazil. Cancer Epidemiol, 35(6), 534-9.

Xiong J, He C, Li C, Tan G, Li J, Yu Z, Hu Z, Chen F (2013). Changes of dendritic spine density and morphology in the superficial layers of the medial entorhinal cortex induced by extremely low-frequency magnetic field exposure. PLoS One, 8(12), e83561.

Xu S, Zhou Z, Zhang L, Yu Z, Zhang W, Wang Y, Wang X, Li M, Chen Y, Chen C, He M, Zhang G, Zhong M (2010). Exposure to 1800 MHz radiofrequency radiation induces oxidative damage to mitochondrial DNA in primary cultured neurons. Brain Res, 1311, 189-96.

Xu S, Chen G, Chen C, Sun C, Zhang D, Murbach M, Kuster N, Zeng Q, Xu Z (2013). Cell Type-Dependent Induction of DNA Damage by 1800 MHz Radiofrequency Electromagnetic Fields Does Not Result in Significant Cellular Dysfunctions. PLoS One, 8(1), e54906.

Xu L, Meng MH, Ren H, Chan Y (2009). Radiation characteristics of ingestible wireless devices in human intestine following radio frequency exposure at 430, 800, 1200, and 2400 MHz. Antennas and Propagation, IEEE Transactions, 57(8), 2418-28.

Yamaguchi-Sekino S, Sekino M, Ueno S (2011). Biological effects of electromagnetic fields and recently updated safety guidelines for strong static magnetic fields. Magn Reson Med Sci, 10(1), 1-10.

Yang L, Hao D, Wang M, Zeng Y, Wu S, Zeng Y (2012). Cellular neoplastic transformation induced by 916 MHz microwave radiation. Cellular and molecular neurobiology, 32, 1039-46.

Yang X, He G, Hao Y, Chen C, Li M, Wang Y, Zhang G, Yu Z (2010). The role of the JAK2-STAT3 pathway in pro-inflammatory responses of EMF-stimulated N9 microglial cells. J Neuroinflammation, 7, 54.

Yang XS, He GL, Hao YT, Xiao Y, Chen CH, Zhang GB, Yu ZP (2012). Exposure to 2.45 GHz electromagnetic fields elicits an HSP-related stress response in rat hippocampus. Brain Res Bull, 88(4),371-8.

Yoon SY, Kim KT, Jo SJ, Cho AR, Jeon SI, Choi HD, Kim KH, Park GS, Pack JK, Kwon OS, Park WY (2011). Induction of hair growth by insulin-like growth factor-1 in 1,763 MHz radiofrequency-irradiated hair follicle cells. PLoS One, 6 (12), e28474.

Zahedi Y, Zaun G, Maderwald S, Orzada S, Pütter C, Scherag A, Winterhager E, Ladd ME, Grümmer R (2014). Impact of repetitive exposure to strong static magnetic fields on pregnancy and embryonic development of mice. J Magn Reson Imaging, 39(3), 691-9.

Zamanian Z, Gharepoor S, Dehghani M (2009). Effects of electromagnetic fields on mental health of staff employed in gas power plants, Shiraz. Pakistan Journal of Biological Science, 13, 956-60.

Zan P, Yang BH, Shao Y, Yan GZ, Liu H (2010). Electromagnetic effects on the biological tissue surrounding a transcutaneous transformer for an artificial anal sphincter system. Journal of Zhejiang University SCIENCE B, 11(12), 931-6.

Zarikoff B, Malone D (2013). A comparison of RF exposure in macro- and femtocells. Health Physics, 105(1), 39–48.

Zaryabova V, Shalamanova T, Israel M (2013). Pilot study of extremely low frequency magnetic fields emitted by transformers in dwellings. Social aspects. Electromagnetic Biology and Medicine, 32(2), 209-17.

Zaun G, Zahedi Y, Maderwald S, Orzada S, Pütter C, Scherag A, Winterhager E, Ladd ME, Grümmer R (2014). Repetitive exposure of mice to strong static magnetic fields in utero does not impair fertility in adulthood but may affect placental weight of offspring. J Magn Reson Imaging, 39(3), 683-90.

Zeni O, Gallerano GP, Perrotta A, Romanò M, Sannino A, Sarti M, D'Arienzo M, Doria A, Giovenale E, Lai A, Messina G, Scarfì MR (2007). Cytogenetic observations in human peripheral blood leukocytes following in vitro exposure to THz radiation: a pilot study. Health Phys, 92(4), 349-57.

Zeni O, Scarfi MR (2012). Experimental Requirements for in vitro Studies Aimed to Evaluate the Biological Effects of Radiofrequency Radiation, Microwave Materials Characterization, Prof. Sandra Costanzo (Ed.), ISBN: 978-953-51-0848-1, InTech, DOI: 10.5772/51421. Available from: http://www.intechopen.com/books/microwave-materials-characterization/experimental-requirements-for-in-vitro-studies-aimed-to-evaluate-the-biological-effects-of-radiofreq

Zeni O, Sannino A, Romeo S, Massa R, Sarti M, Reddy AB, Prihoda TJ, Vijayalaxmi, Scarfì MR (2012). Induction of an adaptive response in human blood lymphocytes exposed to radiofrequency fields: Influence of the universal mobile telecommunication system (UMTS) signal and the specific absorption rate. Mutat Res - Genet Toxicol Environ Mutagen, 747(1), 29-35.

Zhang C, Li Y, Wang C, Lv R, Song T (2013). Extremely low-frequency magnetic exposure appears to have no effect on pathogenesis of Alzheimer's disease in aluminum-overloaded rat. PLoS One, 8(8), e71087.

Zhang M, Li X, Bai L, Uchida K, Bai W, Wu B, Xu W, Zhu H, Huang H (2013). Effects of low frequency electromagnetic field on proliferation of human epidermal stem cells: An in vitro study. Bioelectromagnetics, 34(1), 74-80.

Zhao G, Chen S, Wang L, Zhao Y, Wang J, Wang X, Zhang W, Wu R, Wu L, Wu Y, Xu A (2011). Cellular ATP content was decreased by a homogeneous 8.5 T static magnetic field exposure: role of reactive oxygen species. Bioelectromagnetics, 32(2), 94-101.

Zhijian C, Xiaoxue L, Yezhen L, Deqiang L, Shijie C, Lifen J, Jianlin L, Jiliang H (2009). Influence of 1.8-GHz (GSM) radiofrequency radiation (RFR) on DNA damage and repair induced by X-rays in human leukocytes in vitro. Mutat Res, 677, 100–4.

Zhijian C, Xiaoxue L, Yezhen L, Shijie C, Lifen J, Jianlin L, Deqiang L, Jiliang H (2010). Impact of 1.8-GHz radiofrequency radiation (RFR) on DNA damage and repair induced by doxorubicin in human B-cell lymphoblastoid cells. Mutat Res, 695, 16–21.

Zhou H, Chen G, Chen C (2012). Association between extremely low-frequency electromagnetic fields occupations and amyotrophic lateral sclerosis: a meta-analysis. PLoS One, 7(11), e48354

Zhou L, Schneider JB (2012). A study of RF dosimetry from exposure to an AMI smart meter. IEEE Ant Propagat Magazine, 54(6), 69-80.

Ziegelberger G, Dehos A, Grosche B, Hornhardt S, Jung T, Weiss W (2011). Childhood leukemia - risk factors and the need for an interdisciplinary research agenda. Prog Biophys Mol Biol, 107, 312-314.

Zimmerman JW, Pennison MJ, Brezovich I, Yi N, Yang CT, Ramaker R, Absher D, Myers RM, Kuster N, Costa FP, Barbault A, Pasche B (2012). Cancer cell proliferation is inhibited by specific modulation frequencies. Br J Cancer, 106(2), 307-13.

Zollner S, Pritchard JK (2007). Overcoming the winner's curse: estimating penetrance parameters from case-control data. Am J Hum Genet, 80, 605-15.

Zradziński P (2013). The properties of human body phantoms used in calculations of electromagnetic fields exposure by wireless communication handsets or hand-operated industrial devices. Electromagn Biol Med., 32(2), 226-35.

Literature identified but not cited

Agarwal A, Desai NR, Makker K, Varghese A, Mouradi R, Sabanegh E, Sharma R (2009).Effects of radiofrequency electromagnetic waves (RF-EMW) from cellular phones on human ejaculated semen: an in vitro pilot study. Fertil Steril, 92(4), 1318-25.

Al-Damegh MA (2012). Rat testicular impairment induced by electromagnetic radiation from a conventional cellular telephone and the protective effects of the antioxidants vitamins C and E. Clinics (Sao Paulo), 67(7), 785-92.

Alsanosi AA, Al-Momani MO, Hagr AA, Almomani FM, Shami IM, Al-Habeeb SF (2013). The acute auditory effects of exposure for 60 minutes to mobile`s electromagnetic field. Saudi Med J, 34(2), 142-6.

Atasoy HI, Gunal MY, Atasoy P, Elgun S, Bugdayci G (2012). Immunohistopathologic demonstration of deleterious effects on growing rat testes of radiofrequency waves emitted from conventional Wi-Fi devices. J Pediatr Urol, 9, 223-9 [insufficient dosimetry]

Avendaño C, Mata A, Sanchez Sarmiento CA, Doncel GF (2012). Use of laptop computers connected to internet through Wi-Fi decreases human sperm motility and increases sperm DNA fragmentation. Fertil Steril, 97(1), 39-45.

Azanza MJ, del Moral A, Calvo AC, Pérez-Bruzón RN, Junquera C (2013). Synchronization dynamics induced on pairs of neurons under applied weak alternating magnetic fields Comp Biochem Physiol A Mol Integr Physiol, 166(4), 603-18.

Baliatsas C, van Kamp I, Hooiveld M, Yzermans J, Lebret E (2014). Comparing non-specific physical symptoms in environmentally sensitive patients: Prevalence, duration, functional status and illness behaviour. J Psychosom Res, 76(5), 405-13.

Barchana M, Margaliot M, Liphshitz I (2012). Changes in brain glioma incidence and laterality correlates with use of mobile phones--a nationwide population based study in Israel. Asian Pac J Cancer Prev, 13(11), 5857-63.

Baş O, Sönmez OF, Aslan A, İkinci A, Hancı H, Yıldırım M, Kaya H, Akça M, Odacı E (2013). Pyramidal Cell Loss in the Cornu Ammonis of 32-day-old Female Rats Following Exposure to a 900 Megahertz Electromagnetic Field During Prenatal Days 13–21. NeuroQuantology, 11, 591-9.

Berg-Beckhoff G, Heyer K, Kowall B, Breckenkamp J, Razum O (2010). The views of primary care physicians on health risks from electromagnetic fields. Dtsch Aztebl Int, 107(46), 817-23.

Boase J, Ling R (2013). Measuring Mobile Phone Use: Self-Report Versus Log Data. Journal of Computer-Mediated Communication, 18(4), 508-19.

Bodera P, Stankiewicz W, Antkowiak B, Paluch M, Kieliszek J, Sobiech J, Zdanowski R, Wojdas A, Siwicki AK, Skopińska-Rózewska E (2012). Suppressive effect of electromagnetic field on analgesic activity of tramadol in rats. Pol J Vet Sci, 15(1), 95-100.

Bokkon I, Erdofi-Szabo A, Till A, Lukacs T, Erdofi-Nagy E (2014). EMOST: Elimination of chronic constipation and diarrhea by low-frequency and intensity electromagnetic fields. Electromagnetic Biology and Medicine, 33(1), 68-74.

Buchner K, Eger H (2011). Changes of clinically important neurotransmitters under the influence of modulated RF fields – A long-term study under real-life conditions. Umwelt Medizin Gesellschaft, 24(1), 44-57.

Calabrò E, Condello S, Currò M, Ferlazzo N, Caccamo D, Magazù S, Ientile R (2012). Modulation of heat shock protein response in SH-SY5Y by mobile phone microwaves. World J Biol Chem, 3(2), 34-40.

Calabrò E, Condello S, Currò M, Ferlazzo N, Vecchio M, Caccamo D, Magazù S, Ientile R (2013). 50 Hz Electromagnetic Field Produced Changes in FTIR Spectroscopy Associated with Mitochondrial Transmembrane Potential Reduction in Neuronal-Like SH-SY5Y Cells. Oxid Med Cell Longev, 2013, 414393.

Cammaerts MC, De Doncker P, Patris X, Bellens F, Rachidi Z, Cammaerts D (2012). GSM 900 MHz radiation inhibits ants' association between food sites and encountered cues. Electromagn Biol Med, 31(2), 151-65.

Cammaerts MC, Rachidi Z, Bellens F, De Doncker P (2013). Food collection and response to pheromones in an ant species exposed to electromagnetic radiation. Electromagn Biol Med, 32(3), 315-32.

Cao Y, Zhang W, Lu MX, Xu Q, Meng QQ, Nie JH, Tong J (2009). 900-MHz microwave radiation enhances gamma-ray adverse effects on SHG44 cells. J Toxicol Environ Health A, 72(11-12), 727-32.

Celik S, Aridogan IA, Izol V, Erdogan S, Polat S, Doran S (2012). An evaluation of the effects of long-term cell phone use on the testes via light and electron microscope analysis. Urology, 79, 346-50.

Chu MK, Song HG, Kim C, Lee BC (2011). Clinical features of headache associated with mobile phone use: a cross-sectional study in university students. BMC Neurol, 11, 115.

Cid MA, Ubeda A, Hernández-Bule ML, Martínez MA, Trillo MÁ (2012). Antagonistic effects of a 50 Hz magnetic field and melatonin in the proliferation and differentiation of hepatocarcinoma cells. Cell Physiol Biochem, 30(6), 1502-16.

Cousin ME, Siegrist M (2011). Cell phones and health concerns: impact of knowledge and voluntary precautionary recommendations. Risk Anal, 31(2), 301-11.

Dahmen N, Ghezel-Ahmadi D, Engel A (2009). Blood laboratory findings in patients suffering from self-perceived electromagnetic hypersensitivity (EHS). Bioelectromagnetics, 30(4), 299-306.

Dehghan N, Taeb S (2013). Adverse health effects of occupational exposure to radiofrequency radiation in airport surveillance radar operators. Indian J Occup Environ Med, 17(1), 7-11. Retracted in Indian J Occup Environ Med, 17(2), 40.

Deshmukh PS, Banerjee BD, Abegaonkar MP, Megha K, Ahmed RS, Tripathi AK, Mediratta PK (2013). Effect of low level microwave radiation exposure on cognitive function and oxidative stress in rats. Indian J Biochem Biophys, 50(2), 114-9.

Dimitrijević D, Savić T, Anđelković M, Prolić Z, Janać B (2014). Extremely low frequency magnetic field (50 Hz, 0.5 mT) modifies fitness components and locomotor activity of Drosophila subobscura. Int J Radiat Biol, 90(5), 337-43.

Dogan M, Turtay MG, Oguzturk H, Samdanci E, Turkoz Y, Tasdemir S, Alkan A, Bakir S (2012). Effects of electromagnetic radiation produced by 3G mobile phones on rat brains: magnetic resonance spectroscopy, biochemical, and histopathological evaluation. Hum Exp Toxicol, 31(6), 557-64.

European Economic and Social Committee Opinion (EESC) on electromagnetic hypersensitivity, 2015: http://www.eesc.europa.eu/resources/docs/eesc-2014-05117-01-01-amp-tra-en-2.doc

El Gohary MI, Salama AA, El Saeid AA, El Sayed TM, Kotb HSh (2013). Influence of magnetic field on brain activity during administration of caffeine. Cell Biochem Biophys, 67(3), 929-33. [frequency of ELF-MF unspecified; no details of exposure system]

El-Sayed A, Badr HS, Yahia R, Salem SM, and Kandil AM (2011). Effects of thirty minute mobile phone irradiation on morphological and physiological parameters and gene expression in pregnant rats and their fetuses. African J Biotechnol, 10, 19670–80.

Esmekaya MA, , Aytekin E, Ozgur E, Güler G, Ergun MA, Omeroğlu S, Seyhan N (2011). Mutagenic and morphologic impacts of 1.8 GHz radiofrequency radiation on human peripheral blood lymphocytes (hPBLs) and possible protective role of pre-treatment with Ginkgo biloba (EGb 761). Science of The Total Environment, 410–411, 59–64.

Esmekaya MA, Acar SI, Kıran F, Canseven AG, Osmanagaoglu O, Seyhan N (2013). Effects of ELF magnetic field in combination with Iron(III) chloride (FeCl3) on cellular growth and surface morphology of Escherichia coli (E. coli). Appl Biochem Biotechnol, 169(8), 2341-9.

Fortune JA, Wu BI, Klibanov AM (2010). Radio frequency radiation causes no nonthermal damage in enzymes and living cells. Biotechnology progress, 26, 1772-6.

Fragopoulou AF, Koussoulakos SL, Margaritis LH (2010). Cranial and postcranial skeletal variations induced in mouse embryos by mobile phone radiation. Pathophysiology, 17, 169-77.

Gathiram P, Kistnasamy B and Lalloo U (2009). Effects of a unique electromagnetic field system on the fertility of rats. Arch Environ Occup Health, 64, 93-100.

Gavoçi E, Zironi I, Remondini D, Virelli A, Castellani G, Del Re B, Giorgi G, Aicardi G, Bersani F (2013). ELF magnetic fields tuned to ion parametric resonance conditions do not affect TEA-sensitive voltage-dependent outward K(+) currents in a human neural cell line. Bioelectromagnetics, 34(8),579-88.

Gorpinchenko I, Nikitin O, Banyra O and Shulyak A (2014). The influence of direct mobile phone radiation on sperm quality. Cent European J Urol, 67(1), 65-71. [used a mobile phone as a source of exposure without dosimetry]

Gul A, Celebi H and Uğraş S (2009). The effects of microwave emitted by cellular phones on ovarian follicles in rats. Arch Gynecol Obstet, 280(5), 729-33.

Haghani M, Shabani M and Moazzami K (2013). Maternal mobile phone exposure adversely affects the electrophysiological properties of Purkinje neurons in rat offspring. Neuroscience, 250, 588-98.

Hagstrom M, Auranen J, Ekman R (2013). Electromagnetic hypersensitive Finns: symptoms, perceived sources and treatments, a questionnaire study. Pathophysiology, 20(2), 117-22.

Hao Y, Yang X, Chen C, Yuan-Wang, Wang X, Li M, Yu Z (2010). STAT3 signalling pathway is involved in the activation of microglia induced by 245 GHz electromagnetic fields. Int J Radiat Biol, 86(1), 27-36.

Hassoy H, Durusoy R, Karababa AO (2013). Adolescents' risk perceptions on mobile phones and their base stations, their trust to authorities and incivility in using mobile phones: A cross-sectional survey on 2240 high school students in Izmir, Turkey. Environ Health, 12, 10.

Huang J, Tang T, Hu G, Zheng J, Wang Y, Wang Q, Su J, Zou Y, Peng X (2013). Association between exposure to electromagnetic fields from high voltage transmission lines and neurobehavioral function in children, 8(7), e67284.

İkinci A, Odacı E, Yıldırım M, Kaya H, Akça M, Hancı H, Aslan A, Sönmez OF, Baş O (2013). The Effects of Prenatal Exposure to a 900 Megahertz Electromagnetic Field on Hippocampus Morphology and Learning Behavior in Rat Pups. NeuroQuantology, 11(4), 582-90.

Johansson A, Nordin S, Heiden M, Sandtrom M (2010). Symptoms, personality traits, and stress in people with mobile phone-related symptoms and electromagetnic hypersensitivity. J Psychosom Res, 68, 37-45.

Karaca E, Durmaz B, Aktug H, Yildiz T, Guducu C, Irgi M, Koksal MG, Ozkinay F, Gunduz C, Cogulu O (2012). The genotoxic effect of radiofrequency waves on mouse brain. J Neurooncol, 106(1), 53-8.

Kesari KK, Kumar S, Behari J (2010). Mobile phone usage and male infertility in Wistar rats. Indian J Exp Biol, 48(10), 987-92.

Kesari KK, Kumar S, Behari J (2011). Effects of radiofrequency electromagnetic wave exposure from cellular phones on the reproductive pattern in male Wistar rats. Appl Biochem Biotechnol, 164(4), 546-59.

Kesari KK, Behari J (2012). Evidence for mobile phone radiation exposure effects on reproductive pattern of male rats: role of ROS. Electromagn Biol Med, 31(3), 213-22.

Kesari KK, Kumar S, Nirala J, Siddiqui MH and Behari J (2013). Biophysical evaluation of radiofrequency electromagnetic field effects on male reproductive pattern. Cell Biochem Biophys, 65(2), 85-96.

Khullar S, Sood A, Sood S (2013). Auditory Brainstem Responses and EMFs Generated by Mobile Phones. Indian J Otolaryngol Head Neck Surg, 65(Suppl 3), 645-9.

Kim HJ, Jung J, Park JH, Kim JH, Ko KN, Kim CW (2013). Extremely low-frequency electromagnetic fields induce neural differentiation in bone marrow derived mesenchymal stem cells. Exp Biol Med, 238(8), 923-31.

Kim S, Im W (2010). Static magnetic fields inhibit proliferation and disperse subcellular localization of gamma complex protein3 in cultured C2C12 myoblast cells. Cell Biochem Biophys, 57(1), 1-8.

Köktürk S, Yardimoglu M, Celikozlu SD, Dolanbay EG and Cimbiz A (2013). Effect of Lycopersicon esculentum extract on apoptosis in the rat cerebellum, following prenatal and postnatal exposure to an electromagnetic field. Exp Ther Med, 6(1), 52-56.

Korpinen LH, Paakkonen RJ (2009). Self-report of physical symptoms associated with using mobile phones and other electrical devices. Bioelectromagnetics, 30(6), 431-7.

Kumar N, Sharma VP, Mathur N, Khan MY, Khan RA (2009). Prevalence of headache among extensive and normal cellular phone users. J Neurochem, 110 (supplement 2), 228.

Landegrebe M, Frick U, Hauser S, Hajak G, Langguth B (2009). Association of tinnitus and electromagnetic hypersensitivity: hints for a shared pathophysiology? PLoS One, 4(3), e5026.

Li L, Xiong DF, Liu JW, Li ZX, Zeng GC, Li HL (2014). No effects of power line frequency extremely low frequency electromagnetic field exposure on selected neurobehavior tests of workers inspecting transformers and distribution line stations versus controls. Australas Phys Eng Sci Med, 37(1), 37-44.

Li Y, Héroux P (2014). Extra-low-frequency magnetic fields alter cancer cells through metabolic restriction. Electromagn Biol Med*, 33(4), 264-75.*

Li SS, Zhang ZY, Yang CJ, Lian HY, Cai P (2013). Gene expression and reproductive abilities of male Drosophila melanogaster subjected to ELF-EMF exposure. Mutat Res, 758(1-2), 95-103.

Liu C, Gao P, Xu SC, Wang Y, Chen CH, He MD, Yu ZP, Zhang L, Zhou Z (2013). Mobile phone radiation induces mode-dependent DNA damage in a mouse spermatocyte-derived cell line: a protective role of melatonin. Int J Radiat Biol, 89 (11), 993-1001.

Liu C, Yu J, Yang Y, Tang X, Zhao D, Zhao W, Wu H (2013). Effect of 1 mT sinusoidal electromagnetic fields on proliferation and osteogenic differentiation of rat bone marrow mesenchymal stromal cells. Bioelectromagnetics, 34(6), 453-64.

Liu ML, Wen JQ, Fan YB (2011). Potential protection of green tea polyphenols against 1800 MHz electromagnetic radiation-induced injury on rat cortical neurons. Neurotoxicity research, 20, 270-6.

Liu TT, Wang S, He LH, Ye KP, Xu YC, Zhang FR (2010). Effects of chronic exposure of power frequency magnetic field on neurobehavior in rats. Beijing Da Xue Xue Bao, 42(3), 351-5.

Liu Y, Wang ML, Zhong RG, Ma XM, Wang Q, Zeng Y (2013). The induction of Epstein-Barr Virus early antigen expression in Raji cells by GSM mobile phone radiation. Biomed Environ Sci, 26(1), 76-8.

Lu YS, Huang BT, Huang YX (2012). Reactive oxygen species formation and apoptosis in human peripheral blood mononuclear cell induced by 900 MHz mobile phone radiation. Oxidative medicine and cellular longevity, 2012 (2012), 1-8.

Lukac N, Massanyi P, Roychoudhury S, Capcarova M, Tvrda E, Knazicka Z, Kolesarova A, Danko J (2011). In vitro effects of radiofrequency electromagnetic waves on bovine spermatozoa motility. Journal of Environmental Science and Health, 46(12), 1417-23.

Luo Q, Jiang Y, Jin M, Xu J and Huang HF (2013). Proteomic analysis on the alteration of protein expression in the early-stage placental villous tissue of electromagnetic fields associated with cell phone exposure. Reprod Sci, 20(9), 1055-61.

Mailankot M, Kunnath AP, Jayalekshmi H, Koduru B, Valsalan R (2009). Radio frequency electromagnetic radiation (RF-EMR) from GSM (0.9/1.8GHz) mobile phones induces oxidative stress and reduces sperm motility in rats. Clinics, 64(6), 561-5.

Manjhi J, Kumar S, Behari J, Mathur R (2013). Effect of extremely low frequency magnetic field in prevention of spinal cord injury-induced osteoporosis. J Rehabil Res Dev, 50(1), 17-30.

Margaritis LH, Manta AK, Kokkaliaris KD, Kokkaliaris CD, Schiza D, Alimisis K, Barkas G, Georgiou E, Giannakopoulou O, Kollia I, Kontogianni G, Kourouzidou A, Myari A,

Roumelioti F, Skouroliakou A, Sykioti V, Varda G, Xenos K and Ziomas K (2014). Drosophila oogenesis as a bio-marker responding to EMF sources. Electromagn Biol Med, 33(3), 165-89.

Meg Tseng MC, Lin YP, Cheng TJ (2011). Prevalence and psychiatric comorbidity of self-reported electromagnetic field sensitivity in Taiwan: A population-based study. J Formos Med Assoc, 110(10), 634-41.

Meo SA, Al-Drees AM, Husain S, Khan MM, Imran MB (2010). Effects of mobile phone radiation on serum testosterone in Wistar albino rats. Saudi Med J, 31 (8), 869-73.

Minagawa Y, Saito Y (2014). An analysis of the impact of cell phone use on depressive symptoms among Japanese elders. Gerontology, 60(6), 539-47.

Moen BE, Mollerlokken OJ, Bull N, Oftedal G, Hannson Mild K (2013). Accidental exposure to electromagnetic fields from the radar of a naval ship: A descriptive study. Int Marit health, 64(4), 177-82.

Mohammed HS, Fahmy HM, Radwah NM, Elsayed AA (2013). Non-thermal continuous and modulated electromagnetic radiation fields effects on sleep EEG of rats. J Adv Res, 4(2), 181-7.

Mortazavi SMJ, Mosleh-Shirazi MA, Tavassoli AR, Taheri M, Bagheri Z, Ghalandari R, Bonyadi S, Shafie M, Haghani M (2011). A comparative study on the increased radioresistance to lethal doses of gamma rays after exposure to microwave radiation and oral intake of flaxseed oil, Iran. J Radiat Res, 9, 9–14.

Mortazavi SMJ, Mosleh-Shirazi MA, Tavassoli AR,Taheri M, Mehdizadeh AR, Namazi SAS, Jamali A, Ghalandari R, Bonyadi S, Haghani M and Shafie M (2012). Increased radioresistance to lethal doses of gamma rays in mice and rats after exposure to microwave radiation emitted by a GSM mobile phone simulator. Dose-Response, 11, 281–92.

Mortazavi SMJ, Shirazi KR and Mortazavi G (2013). The study of the effects of ionizing and non-ionizing radiations on birth weight of newborns to exposed mothers. J Nat Sci Biol Med, 4(1), 213-7 [no description of exposure to RF fields].Mortazavi S, Parsanezhad M, Kazempour M, Ghahramani P, Mortazavi A and Davari M (2013). Male reproductive health under threat: Short term exposure to radiofrequency radiations emitted by common mobile jammers. J Hum Reprod Sci, 6(2), 124-8.

Nazıroğlu M, Yüksel M, Köse SA and Özkaya MO (2013). Recent reports of Wi-Fi and mobile phone-induced radiation on oxidative stress and reproductive signaling pathways in females and males. J Membr Biol, 246(12), 869-75.

Narayanan SN, Kumar RS, Paval J, Kedage V, Bhat MS, Nayak S, Bhat PG (2013). Analysis of emotionality and locomotion in radio-frequency electromagnetic radiation exposed rats. Neurol Sci, 34(7), 1117-24.

Nielsen JB, Elstein A, Gyrd-Hansen D, Kildemoes HW, Kristiansen IS, Stovring H (2010). Effects of alternative styles of risk information on EMF risk perception. Bioelectromagnetics, 31(7), 504-12.

Nittby H, Brun A, Strömblad S, Moghadam MK, Sun W, Malmgren L, Eberhardt J, Persson BR, Salford LG (2011). Nonthermal GSM RF and ELF EMF effects upon rat BBB permeability. Environmentalist, 31(2), 140-8.

Nittby H, Moghadam MK, Sun W, Malmgren L, Eberhardt J, Persson BR, Salford LG (2012). Analgetic effects of non-thermal GSM-1900 radiofrequency electromagnetic fields in the land snail Helix pomatia. Int J Radiat Biol, 88(3), 245-52.

Nordin S, Palmquist E, Claeson AS, Stenberg B (2013). The environmental hypersensitivity inventory: metric properties and normative data from a population-based study. Arch Public Health, 71(1), 18.

Nordin S, Palmquist E, Claeson AS (2013). Metric properties and normative data for brief noise and electromagnetic field sensitivity scales. Scand J Public Health 41(3), 293-301.

Odacı E, İkinci A, Yıldırım M, Kaya H, Akça M, Hancı H, Sönmez OF, Aslan A, Okuyan M, Baş O (2013). The Effects of 900 Megahertz Electromagnetic Field Applied in the Prenatal Period on Spinal Cord Morphology and Motor Behavior in Female Rat Pups. NeuroQuantology, 11, 573-81.

Otitoloju AA, Obe IA, Adewale OA, Otubanjo OA, Osunkalu VO (2010). Preliminary study on the induction of sperm head abnormalities in mice, *Mus musculus*, exposed to radiofrequency radiations from global system for mobile communication base stations. Bull Environ Contam Toxicol, 84(1), 51-4.

Palmquist E, Claeson AS, Neely G, Stenberg B, Nordin S (2014). Overlap in prevalence between various types of environmental intolerance. Int J Hyg Environ Health, 217(4-5), 427-34.

Panagopoulos D. J. (2012). Effect of microwave exposure on the ovarian development of Drosophila Melanogaster. Cell Biochem Biophys, 63, 121-32.

Panagopoulos DJ, Karabarbounis A, Lioliousis C (2013). ELF alternating magnetic field decreases reproduction by DNA damage induction. Cell Biochem Biophys, 67(2), 703-16.

Perez FP, Zhou X, Morisaki J, Jurivich D (2008). Electromagnetic field therapy delays cellular senescence and death by enhancement of the heat shock response. Exp Gerontol, 43(4), 307-16.

Pesnya DS, Romanovsky AV (2013). Comparison of cytotoxic and genotoxic effects of plutonium-239 alpha particles and mobile phone GSM 900 radiation in the Allium cepa test. Mutation Research, 750, 27–33.

Razavinasab M, Moazzami K, Shabani M (2014). Maternal mobile phone exposure alters intrinsic electrophysiological properties of CA1 pyramidal neurons in rat offspring. Toxicol Ind Health. 2014 Mar 6. [Epub ahead of print]

Ribeiro EP, Rhoden EL, Horn MM, Rhoden C, Lima LP, Toniolo L (2007). Effects of subchronic exposure to radio frequency from a conventional cellular telephone on testicular function in adult rats. J Urol, 177(1), 395-9.

Salama N, Kishimoto T, Kanayama HO and Kagawa S (2009). The mobile phone decreases fructose but not citrate in rabbit semen: a longitudinal study. Syst Biol Reprod Med, 55(5-6), 181-7.

Salama N, Kishimoto T and Kanayama HO (2010a). Effects of exposure to a mobile phone on testicular function and structure in adult rabbit. Int J Androl, 33(1), 88-94.

Salama N, Kishimoto T, Kanayama HO and Kagawa S (2010b). Effects of exposure to a mobile phone on sexual behavior in adult male rabbit: an observational study. Int J Impot Res, 22(2), 127-33.

Sarapultseva EI, Igolkina JV, Tikhonov VN, Dubrova YE (2013).The in vivo effects of low-intensity radiofrequency fields on the motor activity of protozoa. Int J Radiat Biol, 90(3), 262-7.

Shamsi Mahmoudabadi F, Ziaei S, Firoozabadi M, Kazemnejad A (2013). Exposure to Extremely Low Frequency Electromagnetic Fields during Pregnancy and the Risk of Spontaneous Abortion: A Case-Control Study. J Res Health Sci, 13(2), 131-4.

Sokolovic D, Djordjevic B, Kocic G, Babovic P, Ristic G, Stanojkovic Z, Sokolovic DM, Veljkovic A, Jankovic A, Radovanovic Z (2012). The effect of melatonin on body mass and behaviour of rats during an exposure to microwave radiation from mobile phone. Bratisl Lek Listy, 113(5),265-9.

Song XL, Wang CH, Hu HY, Yu C, Bai C (2011). Microwave induces apoptosis in A549 human lung carcinoma cell line. Chinese medical journal, 124,1193-8.

Speit G, Gminski R, Tauber R (2013). Genotoxic effects of exposure to radiofrequency electromagnetic fields (RF-EMF) in HL-60 cells are not reproducible. Mutat Res, 755, *163-6.*

Su XJ, Yuan W, Tan H, Liu XY, Li D, Li DK, Huang GY, Zhang LW, Miao MH (2014). Correlation between exposure to magnetic fields and embryonic development in the first trimester, PLoS One, 9(6), e101050.

Szemersky R, Koteles F, Lihi R, Bardos G (2010). Polluted places of polluted minds? An experimental sham-exposure study on background psychological factors of symptom formation in 'idiopathic environmental intolerance attributed to electromagnetic fields.' Int J Hyg Environ Health, 213(5), 387-94.

Tiwari R, Lakshmi N, Surender V, Rajesh A, Bhargava S, Ahuja Y (2008). Combinative exposure effect of radiofrequency signals from CDMA mobile phones and aphidicolin on DNA integrity. Electromagn Biol Med, 27, 418–25.

Todorović D, Marković T, Prolić Z, Mihajlović S, Rauš S, Nikolić L, Janać B (2012). The influence of static magnetic field (50 mT) on development and motor behaviour of Tenebrio (Insecta, Coleoptera). Int J Radiat Biol, 89(1), 44-50.

Tseng MC, Lin YP, Hu FC, Cheng TJ (2013). Risks perception of electromagnetic fields in Taiwan: The influence of psychopathology and the degree of sensitivity to electromagnetic fields. Risk Anal, 33(11), 2002-12.

Tsybulin O, Sidorik E, Brieieva O, Buchynska L, Kyrylenko S, Henshel D and Yakymenko I (2013). GSM 900 MHz cellular phone radiation can either stimulate or depress early embryogenesis in Japanese quails depending on the duration of exposure. Int J Radiat Biol, 89(9), 756-63.

Tuengler A, von Klitzing L (2013). Hypothesis on how to measure electromagnetic hypersensitivity. Electromagn Biol Med, 32(3), 281-90.

Tumkaya L, Kalkan Y, Bas O and Yilmaz A (2013). Mobile phone radiation during pubertal development has no effect on testicular histology in rats. Toxicol Ind Health, 2013 Oct 9. [Epub ahead of print]

Umur AS, Yaldiz C, Bursali A, Umur N, Kara B, Barutcuoglu M, Vatansever S, Selcuki D and Selcuki M (2013). Evaluation of the effects of mobile phones on the neural tube development of chick embryos. Turk Neurosurg, 23(6), 742-52.

Velayutham P, Govindasamy GK, Raman R, Prepageran N, Ng KH (2014). High-frequency hearing loss among mobile phone users. Indian J Otolaryngol Head Neck Surg, 66(Suppl 1),169-72.

Vijver MG, Bolte JF, Evans TR, Tamis WL, Peijnenburg WJ, Musters CJ and de Snoo GR (2014). Investigating short-term exposure to electromagnetic fields on reproductive capacity of invertebrates in the field situation. Electromagn Biol Med, 33(1), 21-8.

Volkow, Nora D, Tomasi D, Wang GJ, Vaska P, Fowler JS, Telang F, Alexoff D, Logan J, Wong C (2011). JAMA, 305(8), 808-13.

Wiedemann PM, Schuetz H, Boerner F, Clauberg M, Croft R, Shukla R, Kikkawa T, Kemp R, Gutteling JM, de Villiers B, da Silva Medeiros FN, Barnett J (2013). When precaution creates misunderstandings: The unintended effects of precautionary information on perceived risks, the EMF case. Risk Anal, 33(10), 1788-801.

Wiedemann PM, Boerner FU, Repacholi MH (2014). Do people understand IARC's 2B categorization of RF fields from cell phones? Bioelectromagnetics, 35(5), 373-8.

Witthoft M, Rubin GJ (2013). Are media warnings about the adverse health effects of modern life self-fulfilling? An experimental study on idiopathic environmental intolerance attributed to electromagnetic fields (IEI-EMF). J Psychosom Res, 74(3), 206-12.

Wu H, Wang D, Shu Z, Zhou H (2012). Cytokines produced by microwave-radiated Sertoli cells interfere with spermatogenesis in rat testis. Andrologia, 44, 590-9.

Yang X, He G, Hao Y, Chen C, Li M, Wang Y, Zhang G, Yu Z (2010). The role of the JAK2-STAT3 pathway in pro-inflammatory responses of EMF-stimulated N9 microglial cells. J Neuroinflammation, 7, 54.

Yi G, Wang J, Wei X, Deng B, Tsang KM, Chan WL, Han C (2014). Effects of extremely low-frequency magnetic fields on the response of a conductance-based neuron model. Int J Neural Syst, 24(1),1450007.

Yilmaz F, Dasdag S, Akdag MZ, Kilinc N (2008). Whole-body exposure of radiation emitted from 900 MHz mobile phones does not seem to affect the levels of anti-apoptotic bcl-2 protein. Electromagn Biol Med, 27(1), 65-72.

Zareen N, Khan MY, Ali Minhas L (2009). Derangement of chick embryo retinal differentiation caused by radiofrequency electromagnetic fields. Congenit Anom (Kyoto), 49(1), 15-9.

Zhang Y, She F, Li L, Chen C, Xu S, Luo X, Li M, He M, Yu Z (2013). p25/CDK5 is partially involved in neuronal injury induced by radiofrequency electromagnetic field exposure. Int J Radiat Biol, 89(11), 976-84.
